# Mouse mammary tumor virus-based vector transduces non-dividing cells, enters the nucleus via a TNPO3-independent pathway and integrates in a less biased fashion than other retroviruses

**DOI:** 10.1186/1742-4690-11-34

**Published:** 2014-04-30

**Authors:** Constantine James Konstantoulas, Stanislav Indik

**Affiliations:** 1 Institute of Virology, University of Veterinary Medicine Vienna, Veterinaerplatz 1, Vienna 1210, Austria

**Keywords:** Betaretrovirus, MMTV, Non-dividing cells, TNPO3, Integrase, Random integration

## Abstract

**Background:**

Mouse mammary tumor virus (MMTV) is a complex, milk-born betaretrovirus, which preferentially infects dendritic cells (DC) in the gastrointestinal tract and then spreads to T and B lymphocytes and finally to the mammary gland. It is not clear how the prototypic betaretrovirus infects mucosal DCs and naïve lymphocytes as these cells are considered to be non-proliferative. Studies of MMTV biology have been hampered by the difficulty of obtaining sufficient virus/vector titers after transfection of a molecular clone in cultured cells. To surmount this barrier we developed a novel MMTV-based vector system with a split genome design containing potent posttranscriptional regulatory functions.

**Results:**

Using this system, vector particles were produced to markedly greater titers (>1000-fold) than those obtained previously. The titers (>10^6^ transduction units /ml) were comparable to those achieved with lentiviral or gammaretroviral vectors. Importantly, the vector transduced the enhanced green fluorescence protein gene into the chromosomes of non-dividing cells, such as cells arrested at the G_2_/M phase of the cell cycle and unstimulated hematopoietic progenitor cells, at an efficiency similar to that obtained with the HIV-1-based vector. In contrast to HIV-1, MMTV transductions were not affected by knocking down the expression of a factor involved in nuclear import of the HIV-1 pre-integration complexes, TNPO3. In contrast to HIV-1, the MMTV-based vector did not preferentially integrate in transcription units. Additionally, no preference for integration near transcription start sites, the regions preferentially targeted by gammaretroviral vectors, was observed. The vector derived from MMTV exhibits a random integration pattern.

**Conclusions:**

Overall, the betaretroviral vector system should facilitate molecular virology studies of the prototypic betaretrovirus as well as studies attempting to elucidate fundamental cellular processes such as nuclear import pathways. Random integration in cycling and non-cycling cells may be applicable in unbiased gene delivery.

## Background

Mouse mammary tumor virus (MMTV) is a milk-born betaretrovirus associated with mammary adenocarcinomas in mice [1]. Unlike other retroviruses, MMTV displays a high degree of tissue specificity *in vivo*. Expression of MMTV is mainly limited to the mammary gland, where the virus replicates extensively, particularly during lactation when the levels of glucocorticoid hormone rise. Replication of MMTV during lactation ensures efficient transmission of the virus from mother to offspring. In the infected animals, the virus preferentially infects dendritic cells, naïve B and T lymphocytes and is subsequently transmitted to the mammary gland where it ultimately infects mammary epithelial cells [2,3]. The cell types targeted at the initial stages of infection are reminiscent of the cell types infected by the prototypic lentivirus, human immunodeficiency virus type 1 (HIV-1). Macrophages and dendritic cells at the mucosal surface are the first immune cells targeted by HIV-1. Although these cells are terminally differentiated and as such non-proliferative, they are efficiently productively infected by HIV-1. MMTV, like HIV-1, initiates infection of the host organism by infecting the mucosal dendritic cells. Nevertheless, the possibility that it is capable of infecting non-proliferating cells has not been experimentally tested. Studies of the prototypic betaretrovirus have been hampered due to a low virus production from natural virus producers (such as cell lines derived from mammary tumors), as well as insufficient MMTV-based vector production after transfection of molecular clones into cultured cells. This is a result of the complex regulation of virus gene expression, which is dependent not only on the glucocorticoid hormone, stimulating the MMTV promoter, but also on a functional analogue of the HIV-1 Rev protein, Rem. [4-6]. Rem, like Rev, binds to its responsive element on viral RNA, the ribonucleoprotein complex is then recognized by a cellular factor CRM1, recruited to the nuclear pore complex and exported to the cytoplasm [7-10].

Understanding of the regulation of gene expression enabled us to design and construct a novel MMTV-based vector system analogous to the third generation lentiviral vectors. Replication-incompetent vectors, pseudotyped with the G protein of the vesicular stomatitis virus (VSV-G), were produced to ~1000 fold greater titers than those obtained previously. The high titer vector production allowed us to address the question of whether MMTV is capable of infecting non-mitotic cells such as γ-irradiated cell lines and un-stimulated hematopoietic stem cells. Here we provide the first direct evidence that MMTV-based vector transduces non-proliferating cells, thereby explaining its ability to infect cells such as mucosal dendritic cells and naïve B lymphocytes. The prototypic betaretrovirus can thus be added to the small group of retroviruses that are capable of infecting cells independently of cell cycle progression. In contrast to other retroviruses, however, the MMTV pre-integration complex does not seem to require a cellular factor TNPO3 for nuclear import. Additionally, the high titer MMTV-based vector exhibits, regardless of whether cycling or non-cycling cells were transduced, a random integration pattern reminiscent of the unbiased integration profile previously demonstrated for the wild type MMTV [11]. This property distinguishes MMTV-based vector from other vectors derived from gammaretroviruses and lentiviruses, which preferentially integrate near transcriptional start sites and within genes, respectively [12,13].

## Results

### Description of MMTV-based vector with a split genome design

**Figure 1:**
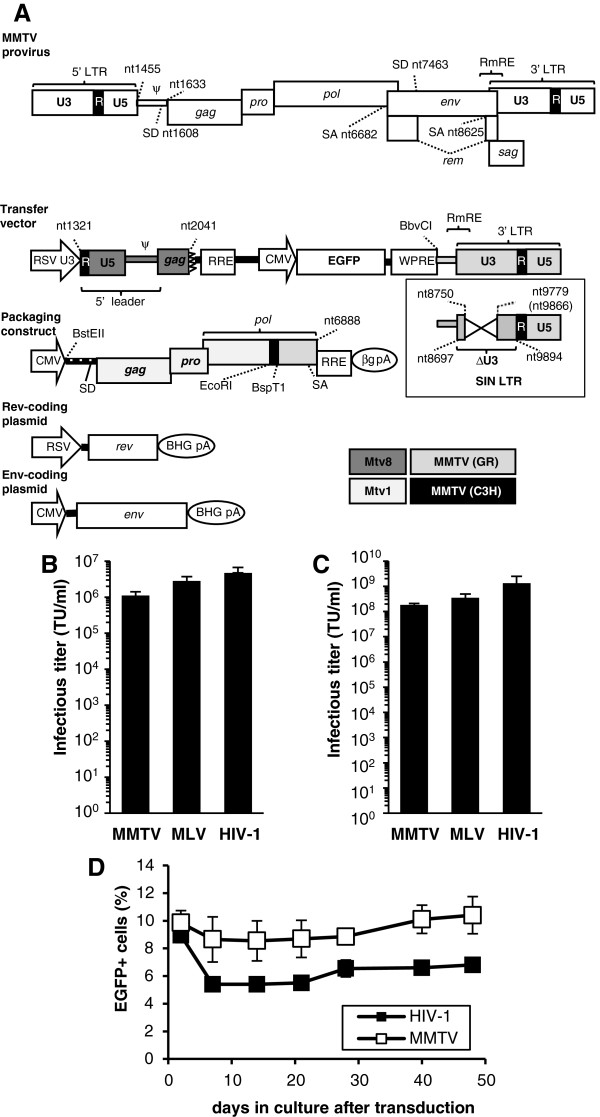
**MMTV-based vector system. (A)** Schematic representation of the MMTV provirus and the four-plasmid expression system used for generating MMTV-based vectors. For the MMTV provirus the regions encoding structural, enzymatic and accessory proteins are shown. The splice donor (SD) and acceptor (SA) sites with their nucleotide coordinates, the long terminal repeat (LTR), the Rem responsive element (RmRE) and the packaging signal are also indicated. In the transfer vector, pRRpCeGFPWPRE25, the truncated gag gene is followed by the Rev responsive element (RRE) from HIV-1 and the internal CMV promoter that drives the expression of enhanced green fluorescent protein (EGFP). Woodchuck hepatitis virus posttranscriptional element (WPRE) upstream of the 3′ LTR is also indicated. In the packaging construct pCMgpRRE17, the restriction sites used for the construction of this chimera, SD, SA, RRE and the beta globin polyadenylation signal (βg pA) are shown. The Rev-encoding plasmid (pRSV-Rev) and the VSV-G envelope protein-encoding plasmid (pHCMV-G) have been previously described [14,22]. **(B)** and **(C)** Infectious titers of VSV-G-pseudotyped vectors (transduction units [TU]/ ml) determined by flow cytometric analysis on transduced HeLa cells three days post transduction using un-concentrated **(B)** or concentrated **(C)** supernatant from transfected 293 T cells. The mean values from three infection replicates are shown, together with the standard deviations (SD). **(D)** Time-course analysis of EGFP positivity after single exposure of HeLa cells to the MMTV- or HIV-1-based vectors. The cells were analysed by FACS at various time points (days 3-48) after transduction. Transductions were performed in triplicate. All values are means ± SD. The nucleotide coordinates are based on the prototypic MMTV strain BR6 (GenBank Acc. no.15122).

To overcome the difficulty to produce MMTV vector to high titers that would enable in-depth studies of molecular virology of the prototypic betaretrovirus, we developed a novel betaretrovirus vector production system. Analogously to the third generation of lentiviral vector system, four-plasmids were used to generate MMTV-derived retroviral vector particles by transient transfection [14]. The conditional packaging construct, pCMVgpRRE17, contains the human cytomegalovirus immediate early promoter (CMV), which drives the expression of the structural and enzymatic components of MMTV. The plasmid contains the *Mtv-1* sequences upstream of the *EcoRI* site in *pol* and a sequence derived from the exogenous virus from GR mice [MMTV(GR)] downstream of this site (Figure 1A, the integrase-coding portion of *pol* is derived from the MMTV(GR)). The packaging signal (ψ) was deleted from the 5′leader region [15] and the plasmid is defective for the production of the viral envelope protein. A bovine growth hormone polyadenylation signal (BGH pA), cloned in place of the 3′LTR, serves as transcriptional termination signal. Analogously to other complex retroviruses, efficient expression of the MMTV *gag* and *pol* genes requires the presence of a *cis*-acting posttranscriptional regulatory element, which allows binding of a viral RNA export factor and thereby facilitates the accumulation of RNA in the cytoplasm [7,8]. Therefore, the HIV-1 Rev responsive element (RRE) was inserted downstream of the *pol* gene and the resulting plasmid was cotransfected with a Rev expression vector into HEK293T cells (Figure 1A) [14]. To broaden vector’s tropism and enhance its stability and titer, we used a plasmid encoding a heterologous envelope protein, derived from vesicular stomatitis virus (VSV-G), for pseudotyping the particles generated by pCMVgpRRE17 [16,17]. A chimeric transfer vector, pRRpCeGFPWPRE25, derived from the MMTV molecular clone pGR102 [18] contains all the *cis*-acting sequences of the virus genome required for packaging of the vector RNA and for its reverse transcription and integration into the host genome. The 5′ leader sequence [containing the primer binding site (PBS)], ~ 400 base pairs of *gag*, the 3′end of *env*, [carrying the polypurine tract sequence (ppt)] and the 3′LTR were included in the pRRpCeGFPWPRE25 vector. To ensure efficient and hormone-independent transcription, the U3 region of the Rous sarcoma virus (RSV) LTR, containing the enhancer/promoter, was substituted for the U3 region of the MMTV 5′LTR. The substitution was made in such a manner as to preserve the transcription initiation site of MMTV. The posttranscriptional regulatory element of woodchuck hepatitis virus (WPRE) and RRE sequences were inserted into the transducing vector to stabilize RNA and allow efficient nuclear export of full-length vector transcripts in the presence of the regulatory protein expressed *in trans*[19,20]. The Rem responsive element (RmRE), which spans the *env*-U3 junction, is also present in the construct containing the complete 3′LTR [9,21]. The enhanced green fluorescent protein (EGFP) gene, driven by the CMV promoter, was used as a reporter (Figure 1A).

### High-titer vector production upon co-transfection of the four-plasmids into HEK 293 T cells

Replication-defective MMTV-based vector particles were generated by transient co-transfection of the four plasmids into HEK293T cells. As controls, third generation HIV-1-derived packaging and transducing vectors and stable MLV vector producers were used [14,23,24]. VSV-G pseudotyped vector particles from various transfectants were filtered and assayed for transduction efficiency on HeLa cells. MMTV-based EGFP-carrying vectors yielded titers of 1.3 × 10^6^ ± 3.5 × 10^5^ transduction units (TU) per milliliter (ml) of unconcentrated supernatant and mean fluorescence intensity (MFI) of 560 ± 157 (Additional file 1: Figure S1). This titer was comparable to that obtained with both the HIV-1-based vector produced by the same method (4.6 × 10^6^ ± 2.0 × 10^6^ TU/ml; MFI = 1585 ± 275) and the stably produced MLV-based vector (2.8 × 10^6^ ± 9.2 × 10^5^ TU/ml; MFI = 324 ± 26) (Figure 1B). Infectious MMTV vector titers above 1 × 10^8^ TU/ml were obtained after concentration by ultracentrifugation (Figure 1C). Furthermore, the pseudobetaretrovirus vector-transduced cells remained EGFP positive for at least seven weeks after transduction, suggesting that the vector was stably integrated into the host genome (Figure 1D).

### SIN design of MMTV-based vector does not decrease vector titer

To reduce genotoxic potential of the MMTV vector harboring a potent enhancer element and to avoid promoter interference, we also generated two self-inactivating (SIN) variants of the transfer vector by leaving out most of the 1.2 kb 3′ U3 region (Figure 1A, inner panel). In both SIN variants the attachment site, required for the interaction with the integrase, was maintained (~50 bp). In the first deletion construct (pRRpCeGFPWPRE25_1293), the complete promoter/enhancer region, including the TATA box, was removed, leaving only the core elements of pre-mRNA processing signals (cleavage and polyadenylation specificity factor [CPSF] and cleavage stimulating factor [CstF] recognition motifs). The second SIN vector (pRRpCeGFPWPRE25_1218) contained, in addition to the CPSF and the CstF recognition motifs, additional *cis*-acting elements including the U-rich region and CF I_m_ recognition motif, involved in the regulation of mRNA polyadenylation [25,26]. When the SIN vector with the longer deletion, pRRpCeGFPWPRE25_1293, was used for transduction, the titers determined were approximately 20% of those obtained with the parental vector carrying an intact 3′LTR. This vector also exhibited a moderately decreased MFI when compared to the parental vector. In contrast, the vector pRRpCeGFPWPRE25_1218, yielded titers slightly greater than the parental vector (Additional file 1: Figure S2A and S2B). As expected, absence of the enhancer abolished hormone responsiveness and no increase in the vector RNA levels could be detected in transduced cells upon dexamethasone stimulation (Additional file 1: Figure S3).

### MMTV-based vector transduces cells arrested in the cell cycle

In contrast to HIV-1 and other lentiviruses, most oncoretroviruses require cell division to enter the host cell genome and integrate viral DNA in the chromosomes [27-33]. Little is known about MMTV’s ability to infect non-dividing cells. In vivo, the milk-transmitted MMTV crosses the gut and preferentially infects naïve B lymphocytes and dendritic cells in the gastrointestinal tract, which are generally non-proliferative [3,34]. This suggests that MMTV has the capacity to infect non-cycling cells.

**Figure 2:**
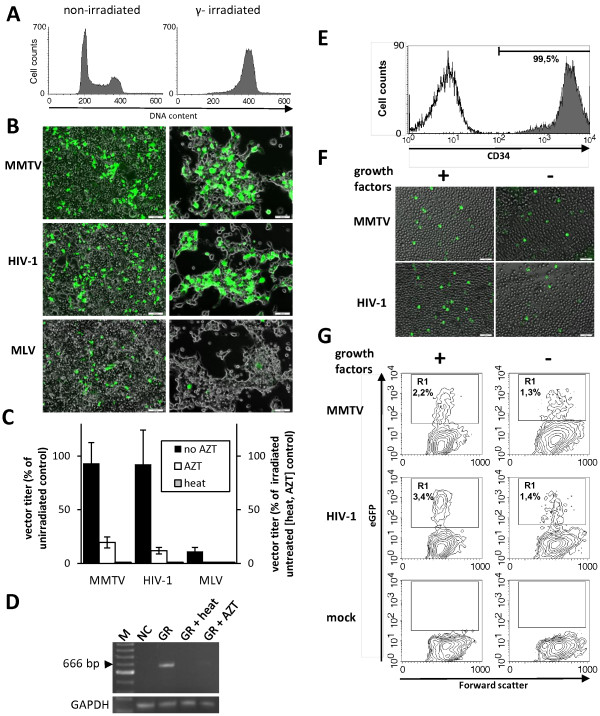
**Transduction of non-dividing cells. (A)** 293 cells were exposed to ionizing radiation (50 Gy) and 24 h later (at the time of transduction) stained with propidium iodide and analysed by flow cytometry. Non-irradiated cells used as a control showed a well defined G_1_ peak. Exposure to radiation resulted in an accumulation of virtually all cells in the G_2_/M stage of the cell cycle. **(B)** The irradiated and non-irradiated cells were transduced with MMTV-based vector. As positive and negative controls, for transduction of non-dividing cells, vectors derived from HIV-1 and MLV were used. The transduced cells were analysed by UV microscopy and FACS analysis 3 days post transduction. **(C)** Viral titers expressed as percentages of titers obtained on dividing cells (black columns). The cells of one set of transduced irradiated cells were exposed to AZT (10 μM, white columns). Another control is represented by a heat-treated vector (65°C, 10 min, grey columns). For the controls, the titer is given as a percentage of titers observed on untreated cells. Error bars indicate standard deviation from the mean of three independent transduction experiments. **(D)** The irradiated cells were infected either with MMTV(GR) or the same virus treated with heat (65°C, 10 min). The infected cells were cultured in the absence or presence of AZT (GR + AZT) for 7 days. MMTV-specific PCR was used to detect the presence of MMTV provirus in the infected cells. GAPDH-specific PCR with equivalent DNA amounts was used as control. **(E)** The purity of bone marrow-derived CD34+ preparations was verified by flow cytometry using an anti-CD34-PE antibody. **(F)**+ **(G)** The hematopoietic progenitor cells (1 × 10^6^ cell/ml) transduced with the MMTV-or HIV-based vector (1 × 10^7^ TU/ml) were cultured in the presence or absence of growth factors for 16 days and analysed by UV microscopy and FACS. Representative figures of microscopic **(G)** and flow cytometric **(F)** analysis of transduced cells are shown.

To assess whether MMTV is able to infect non-dividing cells we used the high-titer betaretrovirus vector and we first assessed its ability to transduce cells arrested in the cell cycle by γ-irradiation. HEK 293 cells exposed to 50 Gy from a linear accelerator source were arrested in the G_2_/M phase (Figure 2A). The irradiated cells and their non-irradiated counterparts were exposed to MMTV(SIN) vector 24 h after the treatment. Parallel transductions with HIV-1 and MLV vectors served as positive and negative controls, respectively. As expected, transduction of G_2_/M-arrested cells with MLV vector was reduced to 11.1 ± 3.5% of the level detected in proliferating cells. In contrast, the HIV-1-based vector efficiently transduced non-proliferating cells, giving rise to titers of 92.4 ± 31.6% of those detected on proliferating cells (Figure 2B and C; 3dpi). Interestingly, the phenotype of MMTV(SIN) was similar to that obtained with the HIV-1 vector. Only a moderate reduction in infectious titers was detected on arrested cells relative to cycling cells (93.2 ± 19.2%) (Figure 2B and C; 3dpi). Importantly, heat treatment of vectors abolished transduction and the presence of 3′-azido-3′-deoxythymidine (AZT) profoundly reduced the number of EGFP-positive cells (Figure 2C). Together, these results indicated that the presence of EGFP cannot be attributed to pseudotransduction events resulting from a passive transfer of EGFP protein or plasmid DNA.

To ascertain whether the wild-type virus can also infect non-proliferating cells, we tested the infectivity of the GR strain of MMTV [MMTV(GR)] on the γ-irradiated cells. As shown in Figure 2D, an MMTV(GR)-specific PCR product was detected in G_2_/M-arrested cells infected with the virus but not in cells infected with a heat-treated virus or in cells cultured in the presence of AZT. Collectively, the results indicate that both the wild-type virus and the MMTV-derived vector are able to infect non-proliferating cells.

### Transduction of hematopoietic stem cells

As the MMTV(SIN) vector transduces G_2_/M-arrested cells, we next investigated whether the vector can also transduce quiescent hematopoietic stem cells (HSC). These cells, which represent attractive targets for gene therapy applications, do not divide unless stimulated with cytokines. Bone marrow-derived human hematopoietic stem cells (CD34+) (Figure 2E) were cultured in StemSpan serum-free medium (StemCell Technologies) either without supplements or supplemented with a cocktail of early-acting cytokines (rhIL-6, rhSCF, rhFlt-3 ligand and rhTPO). The primary CD34+ cells were seeded at a concentration of 1.5 × 10^6^ cells/ml and MMTV(SIN) vector or HIV-1 vector (1.5 × 10^7^ TU/ml) was applied. A direct comparison of the betaretroviral and lentiviral vectors, performed by UV microscopy and flow cytometry on day 16 post-transduction, showed that the transduction rates of the two vectors did not differ markedly. As expected, the HIV-1-based vector transduced the CD34+ cells regardless of whether the cells were stimulated with cytokines. Only a slightly reduced transduction rate was detected when the cells were grown in the absence of cytokines. Importantly, the MMTV-based vectors also transduced the human primary HSC grown without cytokines and the transduction efficiency of the betaretrovirus-based vector was comparable to that of the lentivirus-derived control vector (Figure 2F and G).

Similar results were obtained when the performance of the HIV-1 and MMTV vectors was directly compared in cultured lineage negative HSC derived from the murine bone marrow. Both vectors transduced the lineage negative cells cultured in the absence or the presence of the cytokine cocktail comprising of rmIL-3, rmSCF, rmFlt-3 ligand and rmTPO. Although the transduction rate of the MMTV vector was reduced in the absence of cytokines (by about three-fold), under these conditions the MMTV vector was more efficient than the lentiviral vector (Additional file 1: Figure S4). These results demonstrate the potency of the newly designed betaretroviral split packaging system for transducing human and murine HSC in the absence of cell division.

### TNPO3 depletion does not lead to a reduction of MMTV infectivity

**Figure 3:**
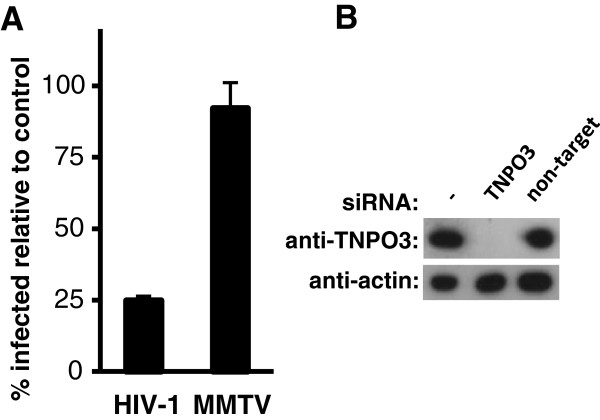
**Effect of TNPO3 knockdown on transduction efficiency of MMTV and HIV-1 vectors. (A)** Hela cells transfected with the TNPO3-specific or non-silencing siRNA were transduced with HIV-1 and MMTV vectors 48 h after transfection. At 48 h post transduction cells were harvested and the percentage of EGFP-positive cells was determined by flow cytometry. Transduction efficiency in TNPO3-specific siRNA-treated cells relative to control siRNA-treated cells is shown. Results represent mean values ± standard deviation of three independent experiments. **(B)** The expression levels of TNPO3 at the time of transduction (48 h post transfection) were determined by immunoblotting with an anti-TNPO3-specific antibody. Equivalent loading and blotting was confirmed by membrane re-probing using an anti-actin-specific antibody.

Infection of non-dividing cells, which have an intact nuclear membrane, requires the active import of a viral nucleoprotein complex [pre-integration complex (PIC)] through the nuclear membrane pores. The mechanisms exploited by lentiviruses to infect non-cycling cells have been a subject of debate. It appears that lentiviruses use several nuclear import pathways to gain access to the host chromosomes. One of the pathways includes a member of the nuclear pore complex Nup358/RanBP2 and a member of the karyopherin β superfamily of proteins,TNPO3/Transportin 3 [35-37]. These proteins associate with the HIV-1 capsid (CA) protein, which, at least to some extent, remains in the pre-integration complex (PICs) and promote nuclear import [38]. To ascertain whether the nuclear entry of MMTV PICs is also dependent on TNPO3, we knocked down the expression of the karyopherin in HeLa cells and followed the transduction levels in these cells. As expected the depletion of TNPO3 resulted in a reduction of the HIV-1 vector infectivity (Figure 3A and B). In contrast, transduction levels of the MMTV vector were not affected by the siRNA treatment (Figure 3A). Thus, the results suggest that MMTV exploits a nuclear import pathway that is distinct from the TNPO3/RanBP2-dependent pathway.

### Neutral integration of MMTV-based vector

**Figure 4:**
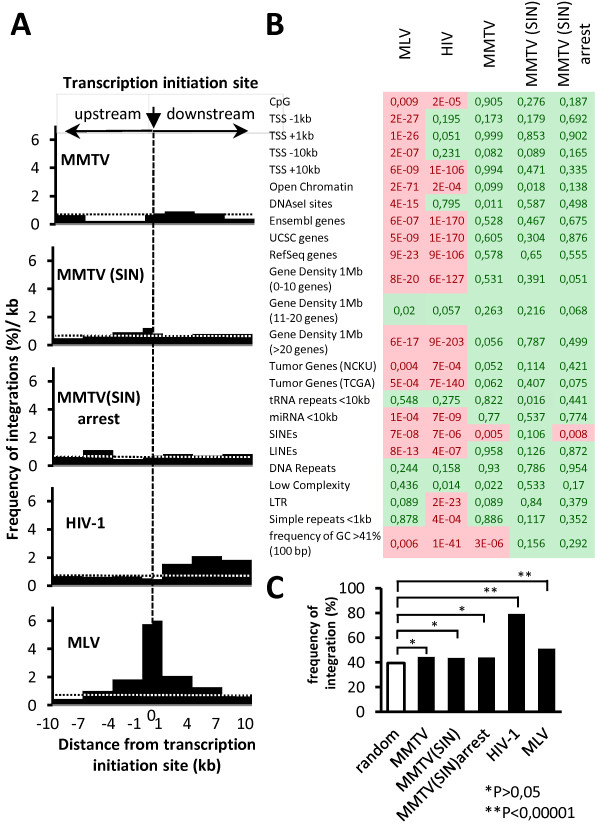
**Distribution of integration sites. (A)** Distribution of MMTV (n = 254), MMTV(SIN) (n = 585), MMTV(SIN)arrest (n = 671), HIV-1 (n = 3246) and MLV (n = 885) integration sites within a 20 kb window centered on Transcription Start Sites (TSS). The vertical dashed line shows TSS (0 kb). The integration sites were binned to 1 kb or 3 kb sequence windows and plotted as the percentage of all integrations per kb. The distribution of a set of 100 000 computationally-generated random integration sites is represented by the dotted horizontal line. **(B)** Integration site preferences of retroviral vectors relative to random control. Tile color code indicates whether the vector integrations departure (red) or not (green) from random control. The numbers represent the Chi-square p-values. P < 0.01 was considered as a significant difference from random dataset. **(C)** Distribution of integration sites within RefSeq genes (hg 18). Differences between virus integrations and random control were analyzed by a Chi-square test.

To investigate the genomic distribution of the integrated vectors, we mapped the location of integration sites in the transduced HeLa cells [MMTV and MMTV(SIN)] and γ-irradiated 293 cells [MMTV(SIN)arrest] (3 dpt) by a ligation-mediated polymerase chain reaction (LM-PCR) followed by 454-pyrosequencing. After a stringent quality control and dereplication, 254 (MMTV), 585 [MMTV(SIN)] and 671 [MMTV(SIN)arrest] unique integration sites unambiguously mapped to the human genome draft hg18. As a first step to analyze the determined integration sites we assessed the frequency of integrations within the Reference Sequence genes (RefSeq, National Center for Biotechnology Information) and regions proximal to transcription start sites (TSS), the features displaying marked integration-targeting differences among retroviruses [12,13,39-42]. The random, computationally generated, integration frequencies (n = 100 000) and previously identified integration sites for HIV-1 and MLV vectors [12,13,39] were used as controls. The integration frequencies of the MMTV vectors in the proximity (±2 kb) of TSS was not increased relative to random control [MMTV(SIN) = 3.7%, [Χ^2^, P = 0.29]; MMTV = 2.6%, [Χ^2^, P = 0.73]; MMTV(SIN)arrest = 2.8%; [Χ^2^, P = 0.81]; random = 3.0%] (Figure 4A). This strongly contrasted with the integration pattern of MLV vector, which preferentially targets TSS (MLV = 15.7%, [Χ^2^, P < 0.0001]) [12].

A significantly lower MMTV vector integration frequencies relative to HIV-1 and MLV integration frequencies were observed when targeting within RefSeqs was analyzed. In contrast to HIV-1 and MLV, MMTV vectors integrated in RefSeq genes with the frequency indistinguishable from that of random placements [MMTV(SIN) (43.6%, [Χ^2^, P = 0.65]); MMTV (44.1%, [Χ^2^, P = 0.58]; MMTV(SIN) arrest = 43.9%, [Χ^2^, P = 0.56]; random = 40.3%) (Figure 4B and C). Similar picture was obtained when we analyzed the distribution of integration sites as a function of gene density. Significanty less MMTV integrants than HIV-1 and MLV integrants were found in the regions with a high gene density (Figure 4B, Additional file 2: Figure S7). The MMTV vectors also integrated less frequently within cancer-related genes (Additional file 2: Figure S7, Additional file 3: Tables S1 and Additional file 4: Table S2). Further, analysis of integration frequency relative to a collection of other genomic features showed that MMTV vectors do not specifically target any of these regions (Figure 4B, Additional file 2: Figure S5 and S6). The only significant difference between random dataset and MMTV-vector integration sites [MMTV; MMTV(SIN)arrest] was detected for integrants mapped to the short interspersed nuclear elements (SINEs) (Figure 4B and Additional file 2: Figure S6).

Together, these results showed that MMTV vectors, like the wild type virus [11], do not preferentially target TSS, genes and most of the other genomic features analyzed. The semi-random integration phenotype strongly contrasts with the phenotype of γ-retroviruses and lentiviruses, which preferentially target TSS and genes, respectively.

## Discussion

Mouse mammary tumor virus (MMTV) has served as an important model in breast cancer research. The glucocorticoid hormone-responsive promoter has been exploited for conditional gene expression in cell culture as well as for preferential expression in organs such as the mammary gland. However, development of an efficient MMTV-derived vector has been hampered by the low vector titers obtained after transfection into cultured cells [4-6]. The failure to produce a high titer vector could result from low cytoplasmic levels of vector RNA attributable to insufficient expression of a functional homologue of the HIV-1 Rev protein, termed Rem. Rem interacts with a Rem-responsive element (RmRE) in viral RNAs and facilitates the cytoplamic accumulation of viral transcripts, which are subsequently either translated into the structural and enzymatic components of the virus or serve as the viral genomic RNA [9,21]. To circumvent the difficulty with low vector titers we produced MMTV-based transducing particles in HEK293T cells using a four-plasmid expression system. The first two plasmids, packaging construct and transfer vector, contain an element from HIV-1 analogous to RmRE (RRE), which in the presence of HIV-1 Rev (supplied *in trans* from the third plasmid) ensures efficient nuclear export of vector transcripts in the human kidney cell line (Rev/RRE was used because Rem/RmRE functioned less efficiently in these cells). The infectious vector titer obtained with this production system (>10^6^ TU/ml) markedly exceeded (~1000-fold) the titers previously reported for MMTV-based vectors [4,5] and was comparable to titers obtained with the third generation of lentivirus-based vector systems and stably produced MLV-derived vectors [14,24]. The MMTV-derived transducing particles could be pseudotyped with VSV-G envelope protein and the pseudoparticles were resistant to high speed centrifugation, allowing further concentration to > 10^8^ TU/ml.

Comparison of integration specificities of MMTV-based vectors with other integrating vectors showed that the high titer MMTV vector does not have a preference for integrating within genes or near promoters, the loci targeted by HIV-1 and MLV vectors, respectively. In contrast, irrespective of whether the complete U3 region is maintained, the vector integrates with a specificity indistinguishable from random integration placements. The random integration pattern is consistent with the reported integration profile of wild-type MMTV [11] and suggests that the MMTV integrase (IN) does not interact with the LEDGF/p75 transcription factor, which is responsible for targeting HIV-1 into active genes [43]. It remains to be elucidated whether the MMTV IN associates with another cellular factor(s), which leads to the random integration profile, or whether it is itself the principal determinant of integration site selection with low-specificity binding to host DNA. Regardless of the mechanism, our results suggest that MMTV-based vectors may be suitable for applications in gene therapy, as they would decrease the risk of mutating and/or activating cellular genes including protooncogenes and tumor suppressor genes. Generation of self inactivating variants of retroviral vectors (SIN), represent another step towards increased biosafety of viral vectors. We also generated a self inactivating (SIN) variant that was as infectious as the non-SIN vector. The SIN vector lacks the potent enhancer element, reducing the risk of enhancer-mediated activation of neighboring genes. Whether the use of the randomly integrating betaretrovirus-based SIN vectors *in vivo* would represent a significant safety improvement over other retroviral vectors remains to be elucidated. MMTV is transmitted via the milk and first infects the mucosal dendritic cells before spreading to T and B lymphocytes in the Peyer’s patches of the gut [2,3]. MMTV encodes an enzyme deoxyuridine triphosphate nucleotidohydrolase (dUTPase) [44]. Although the role of this enzyme in the life cycle of MMTV is unknown, dUTPases of non-primate lentiviruses are thought to facilitate viral replication in non-dividing cells. The enzyme limits dUTPs pools in infected cells and thereby minimizes the misincorporation of uracils into viral DNA [45]. The fact that MMTV encodes dUTPase and is able to infect dendritic cells, which are largely non-proliferative, suggests that the virus may be able to infect cells regardless of cell cycle progression. Retroviruses differ widely in their requirements for mitosis for productive infection of host cells. Some of them are nearly totally dependent on cell cycle progression (MLV, spleen necrosis virus), while others show an intermediate phonotype (avian sarcoma virus) or seem almost completely independent of cell proliferation (lentiviruses) [27-33].

Our novel high-titer MMTV vector production system enabled us to test whether MMTV is capable of infecting non-mitotic cells. We found that the MMTV-based vector transduced cell cycle-arrested cells as well as unstimulated hematopoietic stem cells with the same efficiency as the HIV-1-based vector. The results are unlikely to stem from a leaky cell division of target cells as transduction with the control cell cycle-dependent MLV-based vector was markedly reduced. Pseudotransduction events can also be ruled out as EGFP expression was either not detected or was markedly reduced after transduction with heat-treated vector or when AZT was added to culture medium. The vector cDNA was integrated into the host genome as confirmed by LM-PCR, which identified 671 unique vector-host junctions. Thus, we conclude that reporter gene expression is the result of legitimate, reverse transcription-dependent, transduction of non-cycling cells.

The ability of lentiviruses to cross the intact nuclear membrane of a host cell accounts for their capacity to efficiently replicate in a mitosis-independent fashion. The mechanism by which nuclear entry is achieved is not completely understood.

According to one model the lentiviral capsid protein (CA) is the major determinant of lentivirus’ ability to infect non-mitotic cells [46]. Presumably, an interaction between cellular factor(s) and lentiviral CA affects virus uncoating, thereby influencing downstream events such as nuclear import and integration [35,36,38]. Genome-wide screens using siRNA technology identified a plethora of cellular factors that, when depleted from target cells, reduced the susceptibility of cells to HIV-1 [37,47]. One of them is TNPO3, a karyopherin known to be involved in the nuclear import of serine/arginine-rich splicing factors. TNPO3 has been proposed to promote also the nuclear import of CA-containing PICs via a pathway that involves a large cyclophilin-related nuclear pore protein RanBP2 [37]. We found that TNPO3 is dispensable for infections with MMTV. In contrast to the HIV-1-based vector, transductions with the MMTV vector were not reduced when TNPO3 was depleted from target cells. The result suggests that MMTV uses an alternative, TNOP3-independent nuclear entry pathway. This pathway may be the same as that employed by feline immunodeficiency virus (FIV) and the HIV-1 carrying the N47D substitution in CA [35,38]. Recently, it has been demonstrated that the MMTV CA localizes within the nucleus, further corroborating the importance of the protein for nuclear localization of the MMTV PICs [48].

## Conclusions

We have demonstrated that MMTV may be used as a basis for the construction of a high-titer betaretroviral vector for unbiased delivery of transgenes into host genomes. Comparable efficiency of both the SIN and non-SIN constructs in transferring EGFP offers various possibilities for using MMTV-based vectors. The non-SIN vector may be used as a hormone-regulated retroviral vector, while the SIN vector, lacking the enhancer, may be used for the relatively safe delivery of genes into target cells.

The present study describes for the first time several aspects of the betaretroviral life cycle. We provide evidence explaining MMTV’s natural tropism for infection of mucosal dendritic cells and naïve B-cells. The pathway employed to access the nucleus does not involve TNPO3, a karyopherin used by HIV-1 to reach the host chromosomes. The differences in the nuclear entry pathway may account for the marked differences in integration specificity detected between the prototypic betaretro- and lenti-virus. The described vector should facilitate future betaretrovirology research as well as studies delineating fundamental cellular processes such as transport across the nuclear pore complex.

## Methods

### Cell culture, vector preparations and transductions

HEK293T, HEK 293, HeLa and NMuMG cells were grown in DMEM supplemented with 10% heat inactivated fetal calf serum (FCS). HEK 293 T cells were used for production of MMTV-and HIV-1-based vectors by transient transfection as described previously [14]. Briefly, 5 × 10^6^ HEK 293 T cells were seeded in a 10 cm dish the day before transfection. For the MMTV vector, 5 μg of the packaging construct (pCMgpRRE17), 6 μg of the vector pRRpCeGFPWPRE25 or derivatives, 1.1 μg of the VSV-G Env-encoding plasmid (pHCMV-G) and 3.6 μg of the Rev-encoding plasmid pLP2 (Invitrogen) were co-transfected using calcium phosphate transfection protocol. Cell culture medium was replaced 24 h after transfection with a serum-reduced medium, Opti-MEM (Life Technologies). To harvest vector particles, supernatants were collected 24-36 h later, filtered through a 0.45 μm filter and either stored at−80°C or used for concentration by ultracentrifugation (75 000 × g, 2 h, 4°C). The HIV-1 vector was prepared using the lentiviral packaging (pLP1, Invirogen) and vector (pRRL-cPPT-EGFP; [23]) constructs. The MLV-based vector was obtained from 2GP19Talf packaging cells stably transfected with pCEWmCMV (293Alf-ST-pCEWmCMV; [24]). For transductions, cells were seeded the day before transduction at a density of 2 × 10^5^ cells per well (6 well plate). Viral vectors were added to cells together with polybrene (8 μg/ml) and after 3 h replaced with fresh medium.

### Human and murine haematopoietic progenitors

Human bone marrow-derived hematopoietic CD34+ stem cells (Lonza) were plated at a density of 1.5 × 10 ^6^ cells/ml and cultivated overnight in a serum free StemSpan SFEM medium (StemCell Technologies) with or without a cytokine cocktail composed of 100 ng/ml recombinant human stem cell factor (rhSCF), 20 ng/ml thrombopoietin (rhTPO), 100 ng/ml rhFlt3-L and 20 ng/ml interleukin 6 (rhIL6) (all from Peprotech). 1.5 × 10 ^7^ transduction units of MMTV(SIN) or HIV-1 vectors were added to the cells. After the transduction period (20 h), vector-containing medium was replaced with the original medium (with or without cytokines) and cells were cultured for five days. Then, the cells were resuspended and maintained in the cytokine-containing medium. UV microscopy and FACS analysis were performed two weeks after transduction.

Murine bone marrow cells were flushed from the femurs and tibias of eight-weeks-old Balb/c mice and the lineage negative (Lin^−^) cells were isolated using Lineage Cell Depletion kit (Miltenyi Biotech), following the manufacturer’s instructions. The day before transduction, the Lin^−^ cells were resuspended at a density of 1 × 10^6^ cells/ml in StemSpan SFEM medium with or without cytokines consisting of 100 ng/ml recombinant murine stem cell factor (rmSCF), 100 ng/ml thrombopoietin (rmTPO), 100 ng/ml rmFlt3-L and 20 ng/ml interleukin 3 (rmIL3) (all from PeproTech). The betaretroviral or lentiviral vectors concentrated to 1.8 × 10^7^ TU/ml were added to cells and after 24 h the transduced cells were cultured in the original medium. After five days the original medium was replaced with the medium containing cytokine cocktail and cells were cultured for an additional 10 days.

### Irradiation of cells

HEK 293 cells were seeded at a density of 2 × 10^5^ cells/well (6 well plate) 24 h before exposure to a single radiation dose of 50 Gy using a linear accelerator (Siemens Primus, Munich, Germany). The γ-irradiated cells were used for transductions as well as cell cycle analysis by flow cytometry 24 h after the irradiation.

### Construction of plasmids

The packaging construct pCMgpRRE17, derived from multiple fragments, was assembled in a series of subcloning steps. pCMMTV molecular clone that was used as a base for construction of the packaging construct was described elsewhere [9]. Due to a delayed processing of the Gag-Pro-Pol polyprotein, the *gag-pro-pol*-coding region in the pCMMTV was replaced with the corresponding region from a Mtv-1/MMTV(C3H) hybrid molecular clone [6]. The Mtv-1/MMTV(C3H) fragment was amplified using the hybrid molecular clone as a template and primers 1321FHind 5′-AAA AAA *AAG CTT* GCA ACA GTC CTA ACA TTC AC-3′and 6980R 5′-CTC CTC CGC TTC GGA GAT-3′(HindIII in italics), digested with HindIII (second HindIII site is in *pol*) and cloned into the 9.1 kb HinIII-HindIII backbone from pCMMTV. The resulting chimera pCMMTV1/2 was digested with XcmI and RsrII and ligated with the 2.1 kb XcmI-RsrII fragment obtained from pCMVrem (a Rem expression plasmid constructed by inserting the *rem* ORF into pcDNA3). The intermediate, *env*-3′LTR lacking, product (pCM1/2envΔ) was then processed using MluI and BstEII to delete the packaging signal (pCM1/2envΔΨΔ). The digested vector was linked to a PCR product amplified using primers CMVdelF 5′-ACA ACA AGG CAA GGC TTG AC -3′and CMV3endR 5′-AAA AA*G GTG ACC* GCT AGC AAT ATC GAT AAG CCA GTA AG-3′ (BstEII in italics; MluI is downstream of CMVdelF) and digested with the same restriction enzymes. In the final cloning step the integrase (IN)-coding region from the MMTV(GR) strain was substituted for the IN-coding region present in the pCM1/2envΔΨΔ and the HIV-1 RRE region was inserted . The substitution was performed as follows. The pCM1/2envΔΨΔ was cut with SnaBI and BspTI and ligated with the 4.5 kb SnaBI-BspTI backbone from an HIV-1-based packaging construct containing the MMTV IN coding region in place of the HIV-1 IN coding region (pLP1mIN). The pLP1mIN is derived from pLP1 (Invitrogen). To replace the IN-coding regions, the MMTV IN was amplified using primers CfHIV/MMTV 5′-GGA ATC AGG AAA GTA CTA ACC GCT TTA GAG TCA GCT CA-3′and Dr 5′-AAA AAA CAC CTG C*TC CGG A* TT AAG AAC CTC CTC CGC TTC GGA GAT-3′(AccIII in italics). In a second PCR the HIV-1 RT-coding region was amplified using primers AfHIVpol 5′-GTT CCC TTA GAT AAA GAC TTC-3′ (EcoRV site is downstream of AfHIVpol)and BrMMTV/HIV 5′-TGA GCT GAC TCT AAA GCG GTT AGT ACT TTC CTG ATT CC-3′. The two PCR fragments were fused using an overlap extension PCR strategy with primers AfHIVpol and Dr. The fused product was digested with EcoRV and AccIII and ligated with EcoRV/AccIII-digested pLP1. The resulting packaging construct was named pCMgpRRE17. To construct the vector, the RSV U3 region amplified using RSVf_MluI (5′-TCG CGA *ACG CGT* ATG TAG TCT TAT GCA ATA CTC-3′; MluI in italics) and RSVr (5′-GTG AAT GTT AGG ACT GTT GCC GTT TAT TGT ATC GAG CTA G-3′) was fused to the 1 kb R-U5-leader fragment derived from a molecular clone pGR102 [18] (1321f: 5′-GCA ACA GTC CTA ACA TTC AC-3′; 2041R: 5′-GCT TAG C*CC TCA GC* G AAT TCG GTA CCG ATA TCA AGC TT*C AAT TG* C ATA TGC TGT TCC CCT AGT C-3′; BbvCI and MfeI are italics). The resulting overlap extension PCR product was digested with MluI and BbvCI and ligated to the 6.9 kb MluI-BbvCI fragment derived from the pCMMTV plasmid. In the next step, a cytomegalovirus promoter-driven green fluorescent protein (EGFP) expression cassette with the postrtanscriptional regulatory elements from woodchuck hepatitis virus (WPRE) and HIV-1 (RRE) (excised by MfeI/EcoRI digestion from the 3rd generation lentiviral vector RRL-cPPT-GFP [23]) was inserted to the intermediate vector digested with the same restriction enzymes. The resulting vector was named pRRpCeGFPWPRE25. To construct SIN vectors, a long template PCR using the reverse primer 8750rKpnI (5′-AAA AAA *GGT ACC* CTA AGT GTA GGA CAC TCT CGG-3′) in combination with either the 1218fKpnI (5′-AAA GCT AGC *GGT ACC* TCT GAT CTG AGC TCT TAG TG-3′) or 1293fKpnI (5′-AAA GCT AGC *GGT ACC* AAA GAG TGC TGA TTT TTT GAG-3′) (KpnI in italics) forward primer and the vector as a template was performed (Expand Long Template PCRR System; Roche). The PCR product was digested with KpnI and religated. This resulted in SIN vectors, pRRpCeGFPWPRE25_1218 and pRRpCeGFPWPRE25_1293 having a short (nt8750–nt9779) and long (nt8750–nt9866) deletion in the 3′LTR, respectively (nt coordinates are according to GenBank # M15122). All amplifications, unless stated otherwise, were performed using Phusion High Fidelity polymerase (New England Biolabs).

### Downmodulation of gene expression

For gene knockdown, cells were seeded at a density of 2 × 10^5^ cells/well (6 well plate and transfected using DharmaFECT (Thermo Scientific) with 25 nM siRNA (TNPO3 SMART pool; Thermo Scientific). As a negative control a non-targeting control siRNA (siCONTROL Non-Targeting siRNA #2, Thermo Scientific) was used. After 48 h of siRNA-mediated gene knockdown the medium was removed and viral vectors at an MOI of 0.1 in 0.6 ml DMEM with 10% FCS and 8 μg/ml of polybrene (Sigma) were added. The EGFP-positive cells were counted 72 h later by flow cytometry.

### Flow cytometry

Transduced cells were inspected by fluorescence microscopy and transduction efficiency was analysed by fluorescence-activated cell sorter (FACS with 488 nm argon-ion laser fluorescence excitation source) analysis and CellQuest software (50 000 events; FACSCalibur, BD Biosciences). The cells were harvested, washed with PBS and resuspended in PBS containing 0.5% FCS. Dead cells and debris were excluded from analysis using forward scatter (FSC) vs side scatter (SSC) plot. EGFP-expressing cells were identified by the shift of fluorescence intensity in the FL-1 channel. For antibody staining of hematopoietic stem cells, the cell preparations were resuspended in PBS containing 0.5% BSA and 2 mM EDTA (PBE) and incubated with anti-CD34 (human cells) or anti-CD117 (murine cells) antibody (both Miltenyi Biotech) conjugated with phycoerythrin (PE) for 10 min. Following washing in PBE, the cells were analyzed by FACS. Shift of fluorescence intensity in the FL-2 channel was used for quantification of PE positive cells.

For cell cycle analysis, γ-irradiated and non-irradiated cells were stained with propidium iodide (PI) as described in [49]. Briefly, cells were harvested by trypsinization, collected by centrifugation (200 × g, room temperature, 6 min) and resuspended at a density of 2 × 10^6^ cells per ml of PBS. 0.5 ml of cellular suspension was transferred drop-wise to 4.5 ml of cold ethanol (70%) and incubated overnight on ice. Following centrifugation, the cells were resuspended in PI-staining solution consisting of PBS with 0.1% Triton ×-100, 0.2 g/l DNase-free RNase A and 20 mg/l PI (15 min, 37°C) and analyzed by flow cytometry. Cellular debris and aggregates were excluded from analysis using pulse-width/pulse-area signal (FL2-W vs. FL2-A) and DNA content of single cells was determined (FL2-A vs. counts).

### Detection and mapping of integration sites

Integration sites were recovered as described previously with modifications [12]. Genomic DNA was purified, digested with MseI (37°C, overnight) and ligated to MseI linker (16°C, overnight). Seminested PCR was then performed as decribed previously [50]. PCR products, smaller than the internal control product (437 bp), were gel excised, purified and sequenced on a Genome Sequencer FLX + Titanum (Roche 454 Life Sciences). The obtained reads contained the vector and linker sequences as well as unknown human sequences. The MMTV vector sequences were terminated by the canonical CA dinucleotide, whereas the MMTV(SIN) vector sequences were, due to an aberrant termination of transcription, often terminated before or after the CA dinucleotide at the end of the 3′LTR. These sequences were not derived from non-integrated proviral or plasmid DNA contaminations as we have not detected the 5′LTR virus fused to human genome DNA. Redundant 454 sequencing reads were removed, aligned with the pRRpCeGFPWPRE25_1218 and manually trimmed. Only sequences that matched the human genome within five base pairs after pRRpCeGFPWPRE25_1218 sequence and showed 95% or greater identity to the human genome were considered as true integration sites. Mapping to the human genome (hg18) was performed using BLAT (UCSC Genome Bioinformatics) [51]. The coordinates of RefSeq genes and other genomic features were downloaded from the UCSC Table Browser. The Cancer Genome Atlas (TCGA) (http://cbio.mskcc.org/tcga-generanker/sources.jsp) and Tumor Associated Gene database [52] were used to identify cancer-relevant genes hosting vector integration sites. A Galaxy platform, PERL and Visual Basic scripts were used for comparisons of localizations of integration sites with various chromosomal features [53]. HIV-1 (n = 3246) and MLV (n = 885) integration sites were processed as above for comparison (CL528773–CL529767; BH609398–BH609877; CL799519–CL800849; AY516894.1–AY517469.1; AY515855–AY516880) [12,13,39]. The distribution of vector integration sites was compared to a set of 100,000 random integration coordinates generated by computer. Random coordinates that mapped to un-annotated human genome sequences were removed from the random dataset. Statistical significance of differences in distribution of integration sites was determined using χ^2^ test. P values < 0.05 were considered significant. Sequences were submitted to NCBI.

### RNA extraction and RT-PCR

RNA was extracted from transduced cells using RNeasy Mini kit (Qiagen), resuspended in nuclease free water and treated with Turbo DNase (Ambion). The DNA-free RNA was reverse transcribed into cDNA using SuperScript III First-Strand Synthesis System and random primers, following the manufacturer’s instructions (Invitrogen). A semi-quantitative PCR (Platinum Taq; Invitrogen) was then performed using the primers 1321 F (5′-GCA ACA GTC CTA ACA TTC AC-3′) and 1637R (5′-CCC AGT TCC AAT GGC TCA CCG TAA-3′) under the following cycling conditions: initial denaturation at 94°C for 2 min followed by 25 cycles of 94°C for 15 s, 55°C for 30s and 72°C for 40 s and a final extension at 72°C for 7 min. Equivalent loading the quality of extracted RNA was verified using GAPDH-specific primers GAPDHf (5′-ACA ACG AAT TTG GCT ACA GCA ACA-3′) and GAPDHr (5′-GGT TGA GCA CAG GGT ACT TTA-3′) under the same cycling conditions.

### Western blot analysis

Whole-cell extracts were prepared by cell lysis, equivalent protein content (20 μg; measured using DC Protein Assay; Bio-Rad) boiled in SDS sample buffer, resolved on SDS/PAGE, transferred onto a Hybond-P PVDF membrane (GE Healthcare) and probed with anti-TNPO3 antibody (ab54353; Abcam) followed by secondary antibody conjugated with horse radish peroxidase (DAKO). Equivalent protein loading was demonstrated using anti-actin antibody (A2066; Sigma).

## Competing interests

The authors declare that they have no competing interest.

## Authors’ contributions

SI. designed experiments and wrote the manuscript. SI. and CJK. performed the experiments. Both authors read and approved the final manuscript.

## Supplementary Material

Additional file 1: Figure S1 Mean fluorescence intensity of cells transduced with retroviral vectors. The cells were analyzed by flow cytometry 3 days post transduction. The mean values ± SD from three experiments are shown. **Figure S2.(A)** Infectious titers of the MMTV(SIN) vectors (black columns) relative to the non-SIN vector (white columns). **(B)** Comparison of the mean fluorescence intensities (MFI) of SIN (black columns) and non-SIN vectors (white columns). The mean values ± SD from three experiments are shown. **Figure S3.** Transcription from MMTV (pRRpCeGFPWPRE25) and MMTV(SIN) (pRRpCeGFPWPRE25_1218) provirus. HeLa cells were transduced with either MMTV or MMTV(SIN) vectors and cultured for two weeks. 24 h prior to RNA extraction the cells were stimulated with dexamethasone (DEX+) or mock stimulated (DEX-). Extracted RNA was treated with DNAseI and subjected to reverse transcription followed by PCR with the MMTV-specific primers. Control experiments, in which the RT step was omitted (RT-), served as controls for the genomic DNA contamination. The same cDNA preparations were used for amplification of the GAPDH mRNA. As a positive control 100 pg of plasmid DNA (pGR102; [18]) was used. Mock: mock transduced cells; NTC: no template control. **Figure S4.** Ex vivo transduction of hematopoietic lineage negative (Lin^−^) cells derived from murine bone marrow. Bone marrow cells were enriched for the population of Lin^−^ cells using the Lineage Cell Depletion kit (Miltenyi Biotech). The enriched cells were analysed by FACS using anti-CD117-PE antibody (Miltenyi Biotech)(upper panel). The cells were cultured overnight in the StemSpan medium either containing or lacking a murine cytokine cocktail consisting of 100 ng/ml rmSCF, 100 ng/ml rmTPO, 100 ng/ml rmFlt3-L and 20 ng/ml IL3. Following the 16 h pre-incubation period, the cells (1 × 10^6^ cells/ml) were transduced with MMTV-or HIV-1-based vectors (1.8 × 10^7^ TU/ml). 24 h after transduction, the vector-containing supernatant was replaced with StemSpan medium with or without the murine cytokines. Five days after transduction the cytokine-containing medium was added to cells and they were cultured for 10 days. FACS analysis was used to detect the expression of EGFP in the Lin^−^ cells. Click here for file

Additional file 2: Figure S5 Comparison of integration site distribution within genes, tumor associated genes, CpG islands and open chromatin regions. Nucleotide coordinates for the genomic features were obtained from the USCS Genome Bioinformatics Table Browser using NCBI36/hg18 assembly of the human genome. For the tumor associated genes, The Cancer Gene Atlas (TCGA) Gene Ranker and Tumor Associated Gene Database (NCKU Bioinformatics center) were used to obtain a list of genes. A Chi-square test was used to test differences between random dataset and vectors. **Figure S6.** Comparison of integration site distribution within or near (simple repeats: ± 1 kb; tRNA and miRNA: ± 10 kb) various genomic features. Nucleotide coordinates and statistical evaluation of differences was performed us described above. The GC content in a 100 nt window surrounding the integration sites was computed and the frequency of integrations in the loci with GC content exceeding 41% (average GC content for the human genome) was plotted for vectors and random dataset. **Figure S7.** Gene density around integration sites. RefSeq genes found in the vicinity (1 Mb window) of integration sites were counted. The average and mean number of genes for vectors and random sites are shown. The middle plots show the frequency of integrations in the regions with a gene density of 0-10 genes, 11-20 genes and >20 genes within the 1 Mb window. A Chi-square test was used to test differences between random dataset and vectors. Bottom plots are histograms displaying the distribution of integration sites relative to gene density within a 1 Mb window surrounding integration sites. Click here for file

Additional file 3: Table S1 Vector integration sites within the tumor-associated genes (NCKU Bioinformatics Center). Integrants within the tumor-associated genes are shown. The cell color indicates whether a single or multiple integration events were detected within genes. A single integration within a gene is shown uncolored (name of integrant, position, strand). Two integration events of the same vector in the same gene are in yellow. Two integrations of different vectors are in orange. Red color indicates three different vectors found within the same gene. Click here for file

Additional file 4: Table S2 Vector integrations and the tumor associated genes listed in The Cancer Gene Atlas. The same color coding as above was used. Click here for file
