# Supplementary figures and images for: Mouse mammary tumor virus-based vector transduces non-dividing cells, enters the nucleus via a TNPO3-independent pathway and integrates in a less biased fashion than other retroviruses
Source: Retrovirology. 2014 Apr 30;11:34. doi: 10.1186/1742-4690-11-34 (PMC4098793; doi:10.1186/1742-4690-11-34)

Additional files

Figure S1

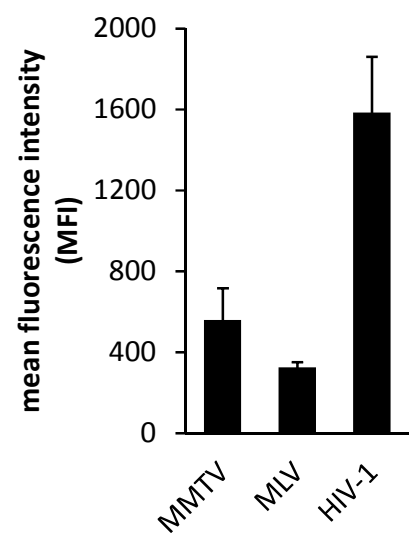

Figure S2

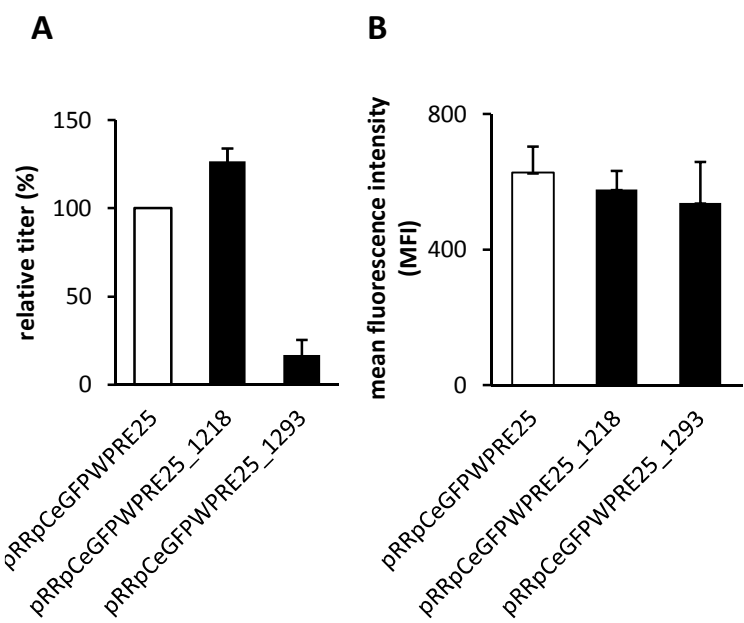

Figure S3

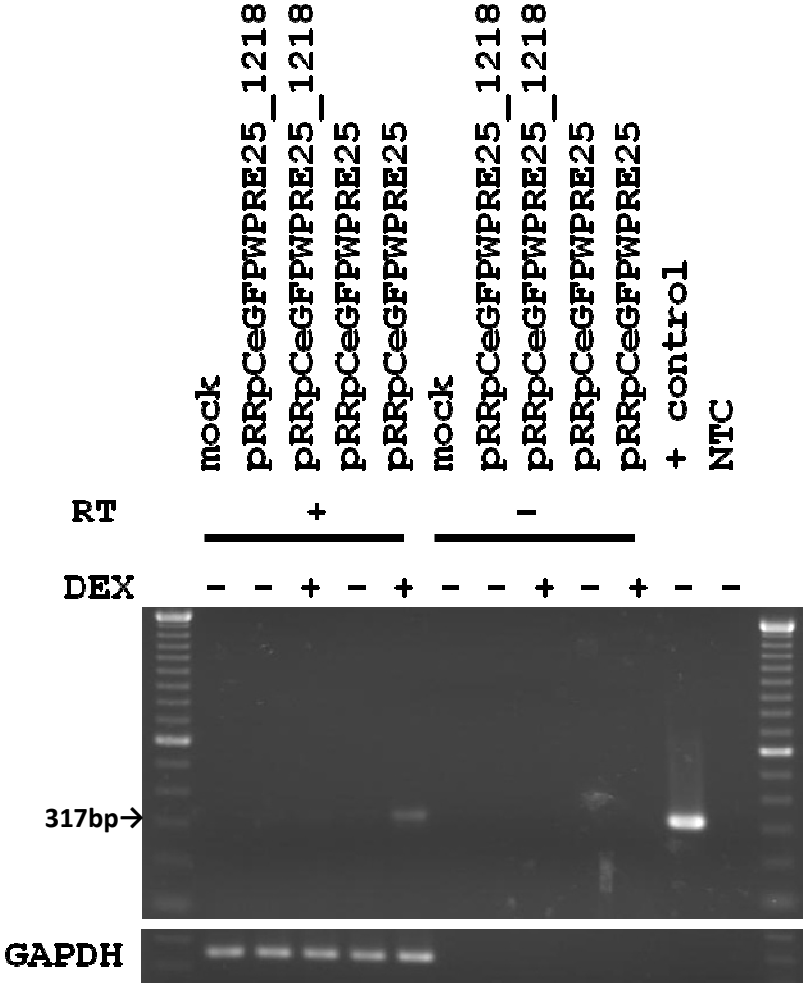

Figure S4

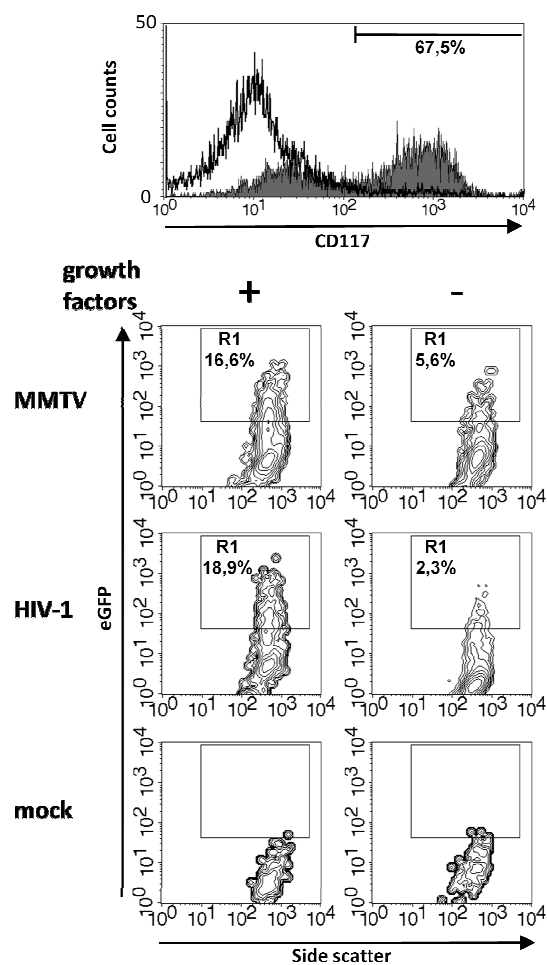

Supplement: Additional file 1: Figure S1 — Mean fluorescence intensity of cells transduced with retroviral vectors. The cells were analyzed by flow cytometry 3 days post transduction. The mean values ± SD from three experiments are shown. Figure S2.(A) Infectious titers of the MMTV(SIN) vectors (black columns) relative to the non-SIN vector (white columns). (B) Comparison of the mean fluorescence intensities (MFI) of SIN (black columns) and non-SIN vectors (white columns). The mean values ± SD from three experiments are shown. Figure S3. Transcription from MMTV (pRRpCeGFPWPRE25) and MMTV(SIN) (pRRpCeGFPWPRE25_1218) provirus. HeLa cells were transduced with either MMTV or MMTV(SIN) vectors and cultured for two weeks. 24 h prior to RNA extraction the cells were stimulated with dexamethasone (DEX+) or mock stimulated (DEX-). Extracted RNA was treated with DNAseI and subjected to reverse transcription followed by PCR with the MMTV-specific primers. Control experiments, in which the RT step was omitted (RT-), served as controls for the genomic DNA contamination. The same cDNA preparations were used for amplification of the GAPDH mRNA. As a positive control 100 pg of plasmid DNA (pGR102; [18]) was used. Mock: mock transduced cells; NTC: no template control. Figure S4. Ex vivo transduction of hematopoietic lineage negative (Lin−) cells derived from murine bone marrow. Bone marrow cells were enriched for the population of Lin− cells using the Lineage Cell Depletion kit (Miltenyi Biotech). The enriched cells were analysed by FACS using anti-CD117-PE antibody (Miltenyi Biotech)(upper panel). The cells were cultured overnight in the StemSpan medium either containing or lacking a murine cytokine cocktail consisting of 100 ng/ml rmSCF, 100 ng/ml rmTPO, 100 ng/ml rmFlt3-L and 20 ng/ml IL3. Following the 16 h pre-incubation period, the cells (1 × 10^6^ cells/ml) were transduced with MMTV-or HIV-1-based vectors (1.8 × 10^7^ TU/ml). 24 h after transduction, the vector-containing supernatant was replaced with StemSpan medium with [file 1742-4690-11-34-S1.pdf]

Figure S5

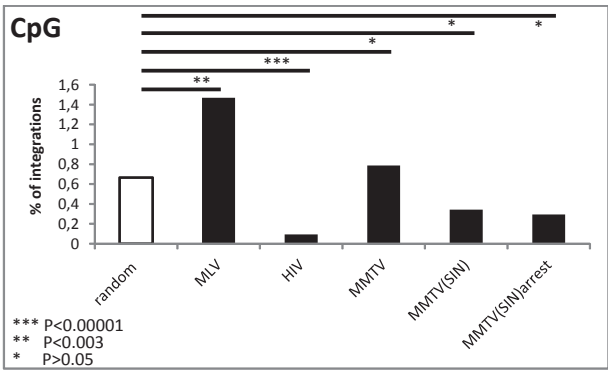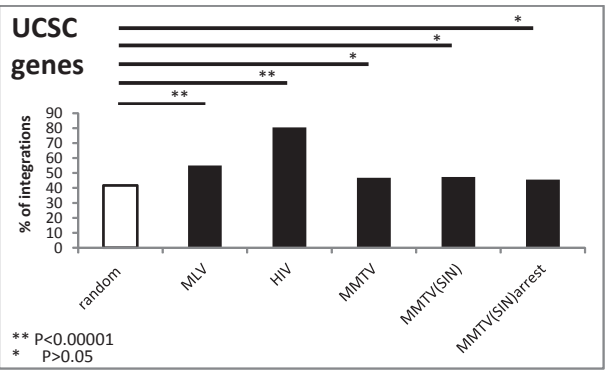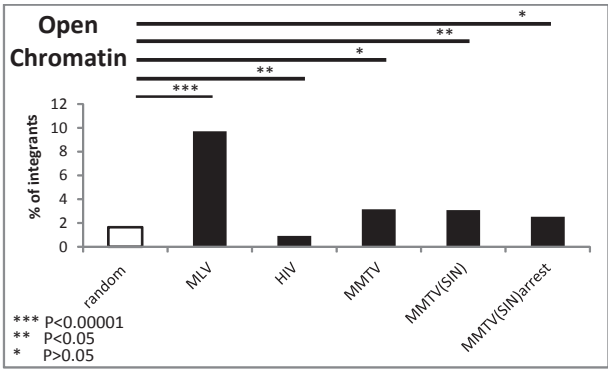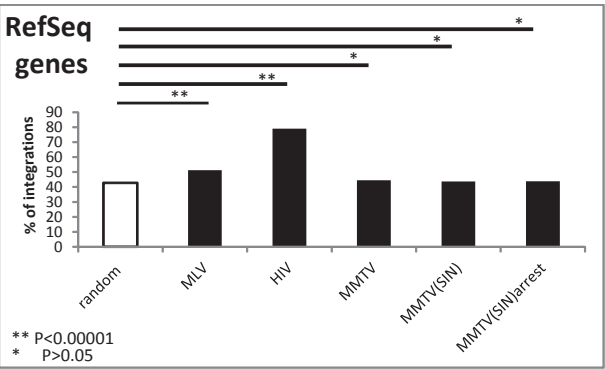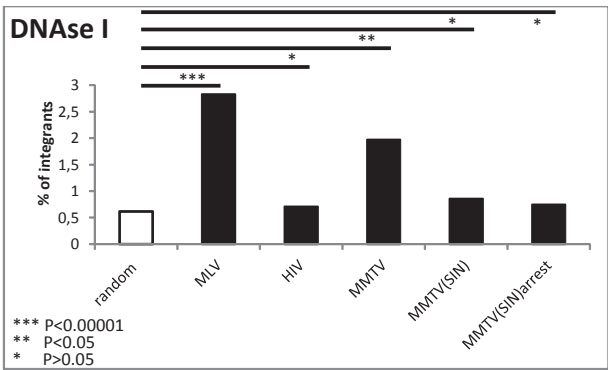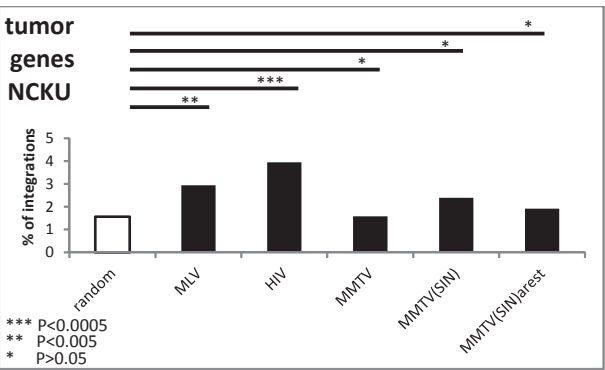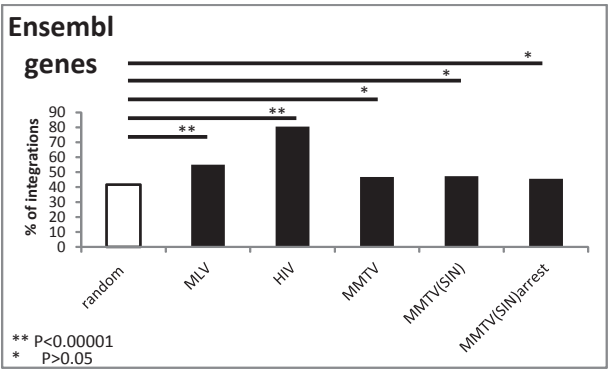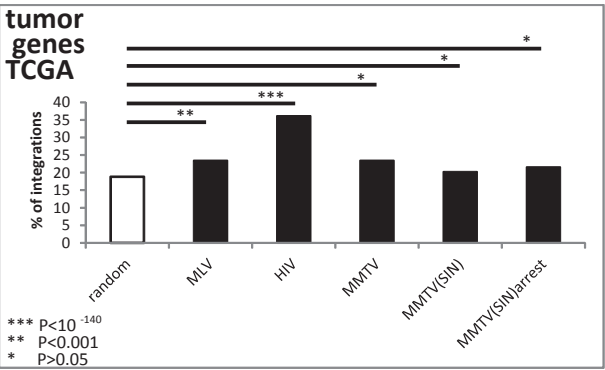

Figure S6

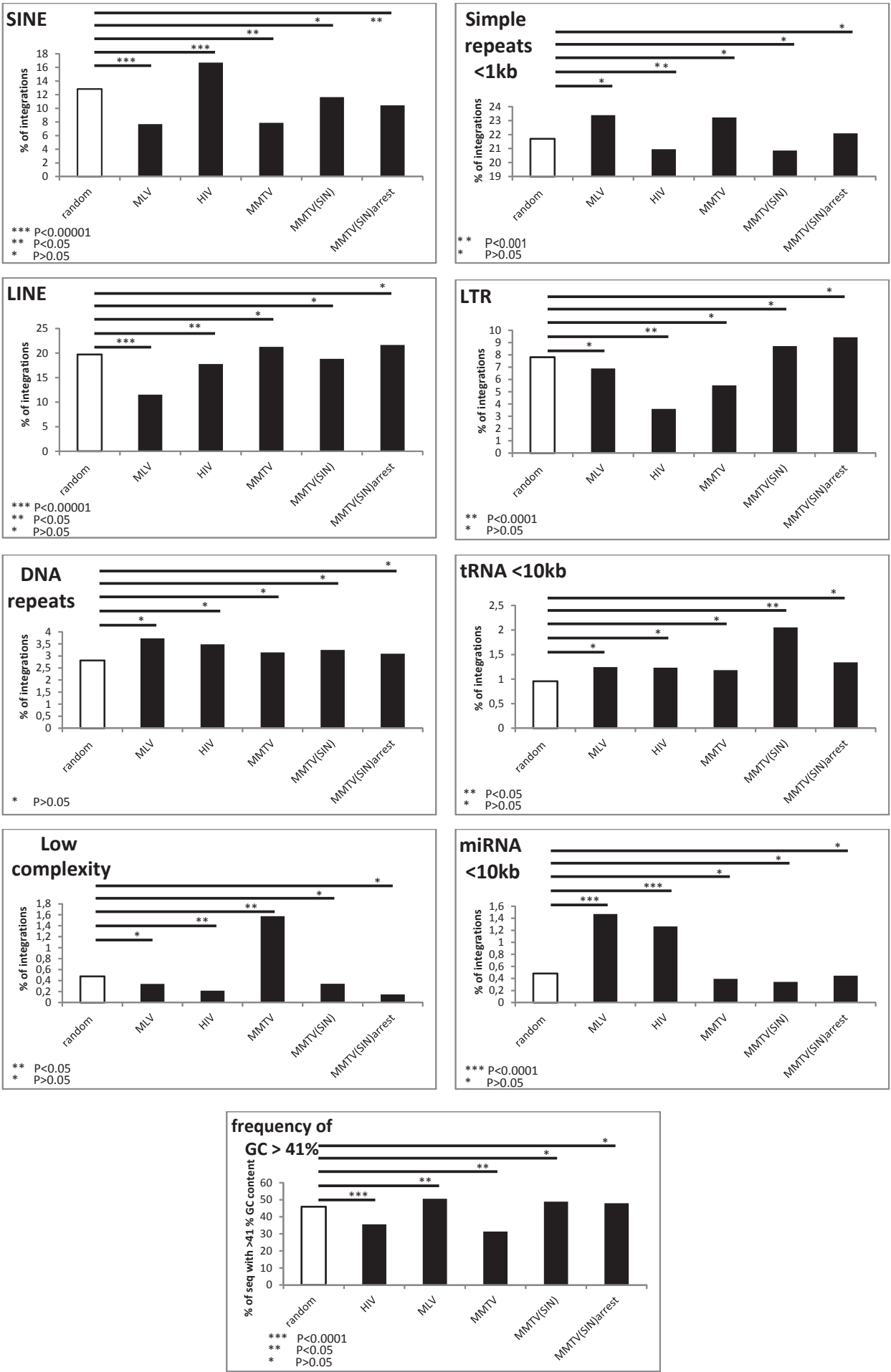

Figure S7

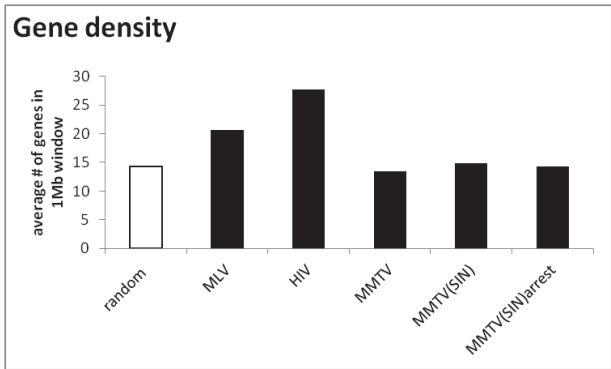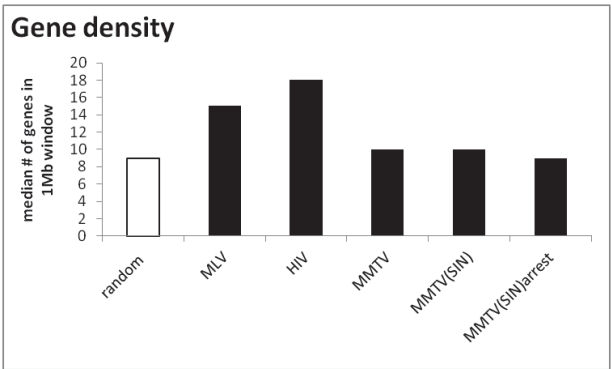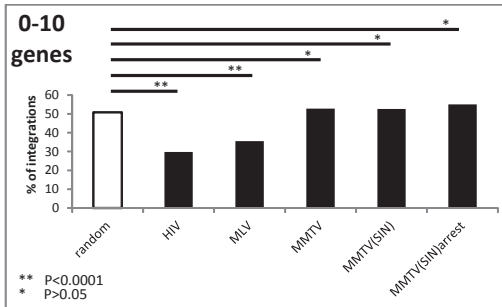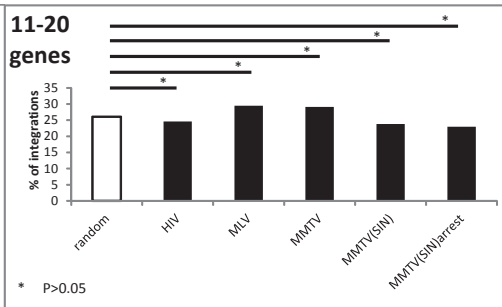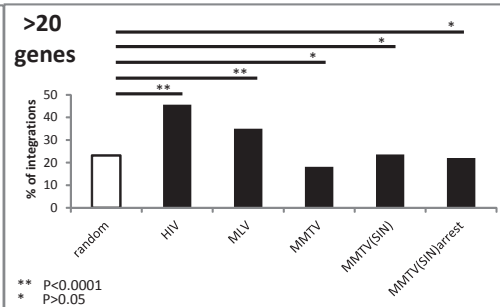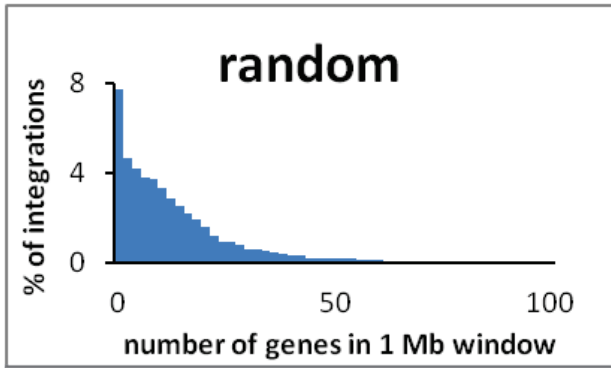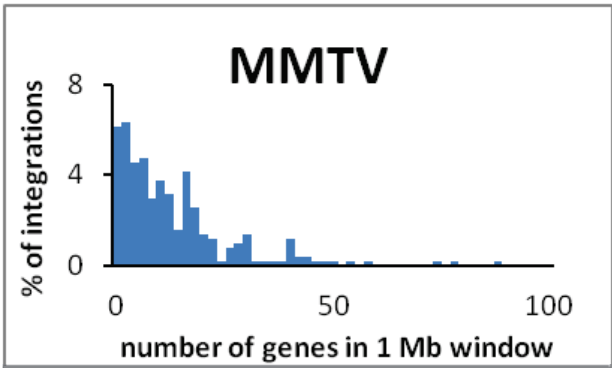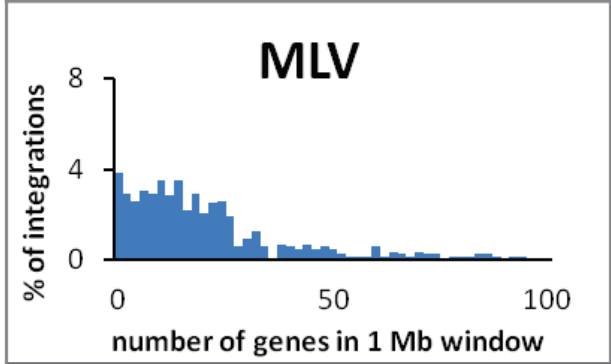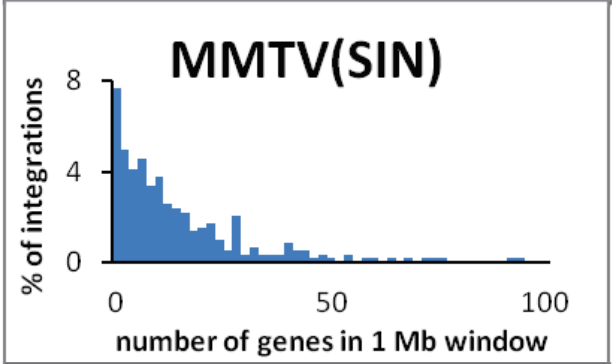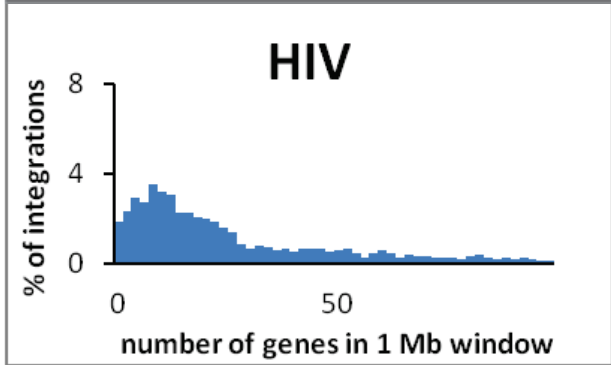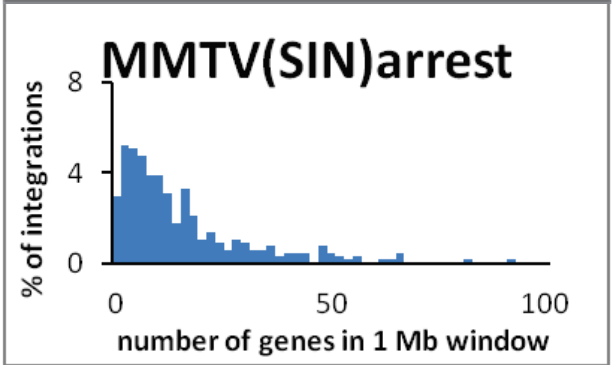

Supplement: Additional file 2: Figure S5 — Comparison of integration site distribution within genes, tumor associated genes, CpG islands and open chromatin regions. Nucleotide coordinates for the genomic features were obtained from the USCS Genome Bioinformatics Table Browser using NCBI36/hg18 assembly of the human genome. For the tumor associated genes, The Cancer Gene Atlas (TCGA) Gene Ranker and Tumor Associated Gene Database (NCKU Bioinformatics center) were used to obtain a list of genes. A Chi-square test was used to test differences between random dataset and vectors. Figure S6. Comparison of integration site distribution within or near (simple repeats: ± 1 kb; tRNA and miRNA: ± 10 kb) various genomic features. Nucleotide coordinates and statistical evaluation of differences was performed us described above. The GC content in a 100 nt window surrounding the integration sites was computed and the frequency of integrations in the loci with GC content exceeding 41% (average GC content for the human genome) was plotted for vectors and random dataset. Figure S7. Gene density around integration sites. RefSeq genes found in the vicinity (1 Mb window) of integration sites were counted. The average and mean number of genes for vectors and random sites are shown. The middle plots show the frequency of integrations in the regions with a gene density of 0-10 genes, 11-20 genes and >20 genes within the 1 Mb window. A Chi-square test was used to test differences between random dataset and vectors. Bottom plots are histograms displaying the distribution of integration sites relative to gene density within a 1 Mb window surrounding integration sites. [file 1742-4690-11-34-S2.pdf]
