# Supplementary material for: Mouse mammary tumor virus-based vector transduces non-dividing cells, enters the nucleus via a TNPO3-independent pathway and integrates in a less biased fashion than other retroviruses
Source: Retrovirology. 2014 Apr 30;11:34. doi: 10.1186/1742-4690-11-34 (PMC4098793; doi:10.1186/1742-4690-11-34)
Supplement: Additional file 3: Table S1 — Vector integration sites within the tumor-associated genes (NCKU Bioinformatics Center). Integrants within the tumor-associated genes are shown. The cell color indicates whether a single or multiple integration events were detected within genes. A single integration within a gene is shown uncolored (name of integrant, position, strand). Two integration events of the same vector in the same gene are in yellow. Two integrations of different vectors are in orange. Red color indicates three different vectors found within the same gene. [file 1742-4690-11-34-S3.pdf]

Table S1

tumor associated genes								HIV			MLV			MMTV			MMTV(SIN)			MMTV(SIN)arrest		
RefSeq	gene symbol	Chr om	txStart	txEnd	s tr a	Categ ory	Gene name	position	integrant	str an d	position	integrant	st ra n	positiopn	integrant	st ra n	position	integrant	st ra n	position	integrant	st ra n
NM_014372	RNF11	chr1	51474532	51511707	+	Other	ring finger protein 11				51498028	AY515884	+									
NM_007262	PARK7	chr1	7944300	7967929	+	Onc	Parkinson disease (autosomal recessive, early onset) 7															
NM_003036	SKI	chr1	2149993	2231512	+	Onc	v-ski sarcoma viral oncogene homolog (avian)															
NM_013945	PAX7	chr1	18830086	18935219	+	Other	paired box gene 7															
NM_001042747	FGR	chr1	27811387	27825338	-	Onc	Gardner-Rasheed feline sarcoma viral (v-fgr) oncogene homolog															
NM_001242884	ZBTB17	chr1	16140950	16175214	-	Other	zinc finger and BTB domain containing 17															
NM_001428	ENO1	chr1	8843645	8861738	-	--	enolase 1, (alpha) ENO1: Approved															
NM_182744	NBL1	chr1	19842309	19857536	+	Tum Sup	neuroblastoma, suppression of tumorigenicity 1															
NM_024674	LIN28A	chr1	26609855	26628806	+	--	lin-28 homolog A (C. elegans) (Previous name: lin-28 homolog (C. elegans)) LIN28A: Approved															
NM_017449	EPHB2	chr1	22909917	23114410	+	Tum Sup	EPH receptor B2 (Previous name: EphB2)															
NM_004455	EXTL1	chr1	26220857	26235541	+	Tum Sup	exostoses (multiple)-like 1															
NM_033489	CDC2L1	chr1	1560963	1645635	-	Tum Sup	cell division cycle 2-like 1 (PITSLRE proteins)															
NM_004350	RUNX3	chr1	25098588	25129357	-	Tum Sup	runt-related transcription factor 3															
NM_006015	ARID1A	chr1	26895108	26981188	+	Tum Sup	AT rich interactive domain 1A (SWI-like) (Previous names: SWI/SNF related, matrix associated, actin															
NM_003689	AKR7A2	chr1	19501788	19511227	-	Other	aldo-keto reductase family 7, member A2 (aflatoxin aldehyde reductase)															
NM_001171940	FNDC5	chr1	33100455	33109001	-	--	fibronectin type III domain containing 5 FNDC5: Approved															
NM_001703	BAI2	chr1	31965304	32002235	-	Tum Sup	brain-specific angiogenesis inhibitor 2															

Table S1

tumor associated genes								HIV			MLV			MMTV			MMTV(SIN)			MMTV(SIN)arrest		
RefSeq	gene symbol	Chrom	txStart	txEnd	stra	Category	Gene name	position	integrant	strand	position	integrant	stran	positiopn	integrant	stran	position	integrant	stran	position	integrant	stran
NM_000300	PLA2G2A	chr1	20174510	20179519	-	Tum Sup	phospholipase A2, group IIA (platelets, synovial fluid)															
NM_001042771	LCK	chr1	32512298	32524353	+	Onc	lymphocyte-specific protein tyrosine kinase															
NM_001033081	MYCL1	chr1	40133682	40140274	-	Onc	v-myc myelocytomatosis viral oncogene homolog 1, lung carcinoma derived (avian)															
NM_005373	MPL	chr1	43576061	43592722	+	Onc	myeloproliferative leukemia virus oncogene															
NM_130440	PTPRF	chr1	43769133	43861930	+	Tum Sup	protein tyrosine phosphatase, receptor type, F															
NM_003738	PTCH2	chr1	45060524	45081203	-	Tum Sup	patched homolog 2 (Drosophila)															
NM_004102	FABP3	chr1	31610686	31618510	-	Tum Sup	fatty acid binding protein 3, muscle and heart (mammary-derived growth inhibitor)															
NR_036634	TGFBR3	chr1	91918487	92124424	-	Tum Sup	transforming growth factor, beta receptor III (Previous names: transforming growth factor, beta rece				92070642	AY516332	-									
NM_002228	JUN	chr1	59019050	59022373	-	Onc	v-jun sarcoma virus 17 oncogene homolog (avian)															
NM_001262	CDKN2C	chr1	51206954	51212894	+	Tum Sup	cyclin-dependent kinase inhibitor 2C (p18, inhibits CDK4)															
NM_001159969	EPS15	chr1	51592522	51660381	-	Other	epidermal growth factor receptor pathway substrate 15															
NM_003189	TAL1	chr1	47454549	47468030	-	Onc	T-cell acute lymphocytic leukemia 1															
NM_001258215	IL12RB2	chr1	67545634	67635171	+	Tum Sup	interleukin 12 receptor, beta 2															
NM_004623	TTC4	chr1	54954082	54980916	+	Tum Sup	tetratricopeptide repeat domain 4															
NM_007034	DNAJB4	chr1	78243223	78255583	+	Tum Sup	DnaJ (Hsp40) homolog, subfamily B, member 4															
NM_004675	DIRAS3	chr1	68284232	68289048	-	Tum Sup	DIRAS family, GTP-binding RAS-like 3															
NM_004815	ARHGAP2[illegible]	chr1	94407050	94475895	-	Tum Sup	Rho GTPase activating protein 29															
NM_003921	BCL10	chr1	85504047	85515175	-	Tum Sup	B-cell CLL/lymphoma 10															

Table S1

tumor associated genes								HIV			MLV			MMTV			MMTV(SIN)			MMTV(SIN)arrest		
RefSeq	gene symbol	Chrom	txStart	txEnd	stra	Category	Gene name	position	integrant	strand	position	integrant	stran	positiopn	integrant	stran	position	integrant	stran	position	integrant	stran
NM_001127216	GFI1	chr1	92712905	92724216	-	Onc	growth factor independent 1															
NM_001261441	EXTL2	chr1	101110515	101133323	-	Tum Sup	exostoses (multiple)-like 2															
NM_000561	GSTM1	chr1	110031940	110037890	+	Tum Sup	glutathione S-transferase M1															
NM_006113	VAV3	chr1	107915304	108309068	-	Onc	vav 3 oncogene															
NM_002524	NRAS	chr1	115048607	115061038	-	Onc	neuroblastoma RAS viral (v-ras) oncogene homolog															
NM_004185	WNT2B	chr1	112811562	112865433	+	Other	wingless-type MMTV integration site family, member 2B															
NR_046070	CHD1L	chr1	145180914	145234071	+	Onc	chromodomain helicase DNA binding protein 1-like															
NM_004326	BCL9	chr1	145479805	145564639	+	Other	B-cell CLL/lymphoma 9															
NM_006694	JTB	chr1	152213368	152217075	-	Other	jumping translocation breakpoint															
NM_006818	MLLT11	chr1	149298774	149307597	+	Onc	myeloid/lymphoid or mixed-lineage leukemia (trithorax homolog, Drosophila); translocated to, 11															
NM_006099	PIAS3	chr1	144287344	144297903	+	Other	protein inhibitor of activated STAT, 3															
NM_001030	RPS27	chr1	152229862	152231255	+	Other	ribosomal protein S27 (metallopanstimulin 1)															
NM_005620	S100A11	chr1	150271605	150276135	-	Other	S100 calcium binding protein A11 (calgizzarin)															
NM_005978	S100A2	chr1	151800208	151804930	-	Tum Sup	S100 calcium binding protein A2 (Previous name: S100 calcium-binding protein A2)															
NM_001243491	SETDB1	chr1	149165438	149184420	+	Other	SET domain, bifurcated 1															
NM_001202859	SHC1	chr1	153201397	153213583	-	Other	SHC (Src homology 2 domain containing) transforming protein 1															
NM_001007792	NTRK1	chr1	155052165	155118266	+	Onc	neurotrophic tyrosine kinase, receptor, type 1															
NM_001145312	ETV3	chr1	155361082	155375007	-	Onc	ets variant gene 3															
AB033071	KIAA1245	chr1	144022460	144076031	+	Onc	KIAA1245															
NM_004833	AIM2	chr1	157298898	157313271	-	Tum Sup	absent in melanoma 2															
NM_016371	HSD17B7	chr1	161027119	161049232	+	Tum Sup	hydroxysteroid (17-beta) dehydrogenase 7															
NM_002585	PBX1	chr1	162795220	163087684	+	Onc	pre-B-cell leukemia transcription factor 1				162983636	AY516655	+									

Table S1

tumor associated genes								HIV			MLV			MMTV			MMTV(SIN)			MMTV(SIN)arrest		
RefSeq	gene symbol	Chrom	txStart	txEnd	stra	Category	Gene name	position	integrant	strand	position	integrant	stran	positiopn	integrant	stran	position	integrant	stran	position	integrant	stran
NM_006182	DDR2	chr1	160868851	161016871	+	--	discoidin domain receptor tyrosine kinase 2 (Previous names: discoidin domain receptor family, membe	160916390	CL528818	+							161011594	1250_176	+			
NM_001136001	ABL2	chr1	177346338	177465442	-	Onc	v-abl Abelson murine leukemia viral oncogene homolog 2 (arg, Abelson-related gene)															
NM_005562	LAMC2	chr1	181421796	181480885	+	Onc	laminin, gamma 2															
NM_002923	RGS2	chr1	191044791	191048030	+	Other	regulator of G-protein signalling 2, 24kDa															
NM_006763	BTG2	chr1	201541286	201545352	+	Tum Sup	BTG family, member 2															
NM_001185158	IL24	chr1	205137410	205144107	+	Tum Sup	interleukin 24															
NM_006618	JARID1B	chr1	200963155	201044172	-	Other	Jumonji, AT rich interactive domain 1B (RBP2-like)															
NR_029626	MIR181A1	chr1	197094795	197094905	-	--	microRNA 181a-1 (Previous name: microRNA 213) MIR181A1: Approved															
NM_004433	ELF3	chr1	200246312	200252938	+	Onc	E74-like factor 3 (ets domain transcription factor, epithelial-specific)															
NM_001270616	PROX1	chr1	212227900	212281470	+	Tum Sup	prospero homeobox 1 (Previous name: prospero-related homeobox 1)															
NM_003238	TGFB2	chr1	216585298	216684584	+	--	transforming growth factor, beta 2 TGFB2: Approved				216623164	AY516051	+									
NM_003238	TGFB2	chr1	216585298	216684584	+	--	transforming growth factor, beta 2 TGFB2: Approved				216621665	AY516388	+									
NM_000740	CHRM3	chr1	237858995	238139340	+	Other	cholinergic receptor, muscarinic 3															
NM_006499	LGALS8	chr1	234753361	234782902	+	Other	lectin, galactoside-binding, soluble, 8 (galectin 8)															
NM_000081	LYST	chr1	233890968	234096843	-	Other	lysosomal trafficking regulator	233997585	CL528836	-												
NM_000081	LYST	chr1	233890968	234096843	-	Other	lysosomal trafficking regulator	233956663	BH609444	-												
NM_000081	LYST	chr1	233890968	234096843	-	Other	lysosomal trafficking regulator	233991757	BH609445	-												
NM_000081	LYST	chr1	233890968	234096843	-	Other	lysosomal trafficking regulator	234030985	BH609446	-												

Table S1

tumor associated genes								HIV			MLV			MMTV			MMTV(SIN)			MMTV(SIN)arrest		
RefSeq	gene symbol	Chrom	txStart	txEnd	stra	Category	Gene name	position	integrant	strand	position	integrant	strand	positiopn	integrant	strand	position	integrant	strand	position	integrant	strand
NM_020247	ADCK3	chr1	225194560	225241869	+	--	aarF domain containing kinase 3 (Previous names: chaperone-ABC1 (activity of bc1 complex, S.pombe)-l															
NM_175876	EXOC8	chr1	229535104	229540201	-	--	exocyst complex component 8 EXOC8: Approved															
NM_181690	AKT3	chr1	241718157	242073207	-	Onc	v-akt murine thymoma viral oncogene homolog 3 (protein kinase B, gamma)				241753682	AY516765	-									
NM_005378	MYCN	chr2	15998133	16004580	+	Onc	v-myc myelocytomatosis viral related oncogene, neuroblastoma derived (avian)															
NR_026766	MYCNOS	chr2	15997470	15999296	-	Other	v-myc myelocytomatosis viral related oncogene, neuroblastoma derived (avian) opposite strand															
NM_004939	DDX1	chr2	15649195	15688686	+	Other	DEAD (Asp-Glu-Ala-Asp) box polypeptide 1															
NM_015909	NAG	chr2	15224483	15618905	-	Other	neuroblastoma-amplified protein															
NM_004040	RHOB	chr2	20510315	20512682	+	Onc	ras homolog gene family, member B															
NM_007046	EMILIN1	chr2	27154938	27162769	+	Other	elastin microfibril interfacer 1															
NM_006887	ZFP36L2	chr2	43303044	43307249	-	Other	zinc finger protein 36, C3H type-like 2															
NM_001135652	EIF2AK2	chr2	37185786	37228469	-	Tum Sup	eukaryotic translation initiation factor 2-alpha kinase 2															
NM_000251	MSH2	chr2	47483709	47563871	+	Tum Sup	mutS homolog 2, colon cancer, nonpolyposis type 1 (E. coli)															
NM_138559	BCL11A	chr2	60531805	60634137	-	Onc	B-cell CLL/lymphoma 11A (zinc finger protein)															
NM_000179	MSH6	chr2	47863724	47887596	+	Other	mutS homolog 6 (E. coli)															
NM_002398	MEIS1	chr2	66516035	66653395	+	Onc	Meis1, myeloid ecotropic viral integration site 1 homolog (mouse)															
NM_014755	SERTAD2	chr2	64712258	64734550	-	Onc	SERTA domain containing 2															

Table S1

tumor associated genes								HIV			MLV			MMTV			MMTV(SIN)			MMTV(SIN)arrest		
RefSeq	gene symbol	Chrom	txStart	txEnd	s tr a	Category	Gene name	position	integrant	str an d	position	integrant	st ra n	positiopn	integrant	st ra n	position	integrant	st ra n	position	integrant	st ra n
NM_002908	REL	chr2	60962255	61003682	+	Onc	v-rel reticuloendotheliosis viral oncogene homolog (avian)															
NM_001025108	LAF4	chr2	99530148	100088477	-	Onc	AF4/FMR2 family, member 3															
NM_001039492	FHL2	chr2	105343714	105382007	-	Other	four and a half LIM domains 2				105359151	AY516497	-									
NM_139347	BIN1	chr2	127522068	127581373	-	Tum Sup	bridging integrator 1															
NM_004336	BUB1	chr2	111111880	111152155	-	Other	BUB1 budding uninhibited by benzimidazoles 1 homolog (yeast)															
NM_000576	IL1B	chr2	113303807	113310827	-	--	interleukin 1, beta IL1B: Approved															
NM_006343	MERTK	chr2	112372661	112503416	+	Onc	c-mer proto-oncogene tyrosine kinase															
NM_002881	RALB	chr2	120726883	120768756	+	Onc	v-ral simian leukemia viral oncogene homolog B (ras related; GTP binding protein)															
NM_018234	STEAP3	chr2	119697853	119739697	+	Tum Sup	STEAP family member 3															
NM_006186	NR4A2	chr2	156889189	156897533	-	--	nuclear receptor subfamily 4, group A, member 2 NR4A2: Approved															
NM_001256850	TTN	chr2	179098963	179380395	-	--	titin (Previous names: cardiomyopathy, dilated 1G (autosomal dominant)) TTN: Approved										179110944	9_1_2118_	-			
NM_000534	PMS1	chr2	190357055	190450600	+	Other	PMS1 postmeiotic segregation increased 1 (S. cerevisiae)	190374673	CL800743	-												
NM_000534	PMS1	chr2	190357055	190450600	+	Other	PMS1 postmeiotic segregation increased 1 (S. cerevisiae)	190409035	BH609465	+												
NM_194247	HNRPA3	chr2	177785668	177796931	+	Other	heterogeneous nuclear ribonucleoprotein A3															
NM_001256310	GLS	chr2	191453791	191508260	+	--	glutaminase GLS: Approved															
NM_005759	ABI2	chr2	203901247	204005137	+	Tum Sup	abl interactor 2															
NM_003507	FZD7	chr2	202607554	202611405	+	Other	frizzled homolog 7 (Drosophila)															

Table S1

tumor associated genes								HIV			MLV			MMTV			MMTV(SIN)			MMTV(SIN)arrest		
RefSeq	gene symbol	Chrom	txStart	txEnd	stra	Category	Gene name	position	integrant	strand	position	integrant	stran	positiopn	integrant	stran	position	integrant	stran	position	integrant	stran
NM_001142355	NIF3L1	chr2	201462294	201476900	+	--	NIF3 NGG1 interacting factor 3-like 1 (S. cerevisiae) (Previous names: NIF3 (Ngg1 interacting factor															
NM_001042599	ERBB4	chr2	211948686	213111597	-	Onc	v-erb-a erythroblastic leukemia viral oncogene homolog 4 (avian)															
NM_000465	BARD1	chr2	215301519	215382673	-	Tum Sup	BRCA1 associated RING domain 1	215317107	CL528871	-												
NM_000465	BARD1	chr2	215301519	215382673	-	Tum Sup	BRCA1 associated RING domain 1	215325388	CL529698	+												
NM_181458	PAX3	chr2	222772849	222871959	-	Other	paired box gene 3 (Waardenburg syndrome 1)															
NM_005689	ABCB6	chr2	219782731	219791956	-	Other	ATP-binding cassette, sub-family B (MDR/TAP), member 6															
NM_000091	COL4A3	chr2	227737524	227887752	+	Tum Sup	collagen, type IV, alpha 3 (Goodpasture antigen)															
NM_001257198	CUL3	chr2	225043110	225142786	-	Tum Sup	cullin 3															
NM_170712	RASSF1	chr3	50342220	50350668	-	Tum Sup	Ras association (RalGDS/AF-6) domain family member 1															
NM_005778	RBM5	chr3	50101344	50131401	+	Tum Sup	RNA binding motif protein 5	50121835	CL800686	-												
NM_005778	RBM5	chr3	50101344	50131401	+	Tum Sup	RNA binding motif protein 5	50113941	CL800600	+												
NM_001167582	RBM6	chr3	49952480	50089689	+	Tum Sup	RNA binding motif protein 6	49963465	CL800557	+												
NM_001167582	RBM6	chr3	49952480	50089689	+	Tum Sup	RNA binding motif protein 6	50072625	CL528884	+												
NM_001167582	RBM6	chr3	49952480	50089689	+	Tum Sup	RNA binding motif protein 6	50056717	CL800375	+												
NM_001664	RHOA	chr3	49371582	49424530	-	Other	ras homolog gene family, member A															
NM_001251877	USP4	chr3	49324221	49352540	-	Onc	ubiquitin specific peptidase 4 (proto-oncogene)															
NM_006010	ARMET	chr3	51397743	51401815	+	Other	arginine-rich, mutated in early stage tumors															
NM_002841	PTPRG	chr3	61522282	62255613	+	Tum Sup	protein tyrosine phosphatase, receptor type, G															
NM_001256105	WNT5A	chr3	55474782	55490466	-	Tum Sup	wingless-type MMTV integration site family, member 5A															

Table S1

tumor associated genes								HIV			MLV			MMTV			MMTV(SIN)			MMTV(SIN)arrest		
RefSeq	gene symbol	Chrom	txStart	txEnd	stra	Category	Gene name	position	integrant	strand	position	integrant	stran	positiopn	integrant	stran	position	integrant	stran	position	integrant	stran
NM_001098209	CTNNB1	chr3	41215945	41256943	+	Onc	catenin (cadherin-associated protein), beta 1, 88kDa															
NM_002070	GNAI2	chr3	50248650	50271790	+	Other	guanine nucleotide binding protein (G protein), alpha inhibiting activity polypeptide 2															
NM_022171	TCTA	chr3	49424642	49428913	+	Tum Sup	T-cell leukemia translocation altered gene															
NM_182920	ADAMTS9	chr3	64476370	64648405	-	Tum Sup	ADAM metallopeptidase with thrombospondin type 1 motif, 9															
NM_001166243	FHIT	chr3	59710075	61212173	-	Tum Sup	fragile histidine triad gene	60272158	BH609473	-							60703533	2382_297	-			
NM_001166243	FHIT	chr3	59710075	61212173	-	Tum Sup	fragile histidine triad gene	60729652	CL800041	+												
NM_013270	TSP50	chr3	46728610	46734800	-	Onc	testes-specific protease 50															
NM_001007565	TFG	chr3	101910823	101950501	+	Onc	TRK-fused gene															
NM_198793	CD47	chr3	109244630	109292625	-	Other	CD47 antigen (Rh-related antigen, integrin-associated signal transducer)															
NM_170662	CBLB	chr3	106859798	107070577	-	Other	Cas-Br-M (murine) ecotropic retroviral transforming sequence b	107048744	CL529618	+												
NM_170662	CBLB	chr3	106859798	107070577	-	Other	Cas-Br-M (murine) ecotropic retroviral transforming sequence b	106896467	AY517375.	-												
NM_002338	LSAMP	chr3	117003899	117647075	-	Tum Sup	limbic system-associated membrane protein										117486060	4_1_1120_	-			
NM_007354	C3orf27	chr3	129773532	129777619	-	Other	chromosome 3 open reading frame 27															
NM_002958	RYK	chr3	135358667	135452276	-	Onc	RYK receptor-like tyrosine kinase															
NM_001252093	MRAS	chr3	139550197	139607067	+	Onc	muscle RAS oncogene homolog															
NM_006286	TFDP2	chr3	143145959	143230197	-	Other	transcription factor Dp-2 (E2F dimerization partner 2)															
NM_001105077	EVI1	chr3	170283981	170346761	-	Onc	ecotropic viral integration site 1															
NM_001195432	MLF1	chr3	159771646	159806943	+	Onc	myeloid leukemia factor 1															

Table S1

tumor associated genes								HIV			MLV			MMTV			MMTV(SIN)			MMTV(SIN)arrest		
RefSeq	gene symbol	Chrom	txStart	txEnd	stra	Category	Gene name	position	integrant	strand	position	integrant	stran	positiopn	integrant	stran	position	integrant	stran	position	integrant	stran
NM_000902	MME	chr3	156280129	156384212	+	Onc	membrane metallo-endopeptidase (neutral endopeptidase, enkephalinase, CALLA, CD10)															
NM_002888	RARRES1	chr3	159905133	159932969	-	Tum Sup	retinoic acid receptor responder (tazarotene induced) 1															
NM_001145097	SKIL	chr3	171560104	171597331	+	Onc	SKI-like													171594141	435_104_1	+
NM_016820	OGG1	chr3	9766627	9774089	+	--	8-oxoguanine DNA glycosylase OGG1: Approved															
NM_198156	VHL	chr3	10158318	10170354	+	Tum Sup	von Hippel-Lindau tumor suppressor															
NM_001018115	FANCD2	chr3	10043112	10118614	+	Tum Sup	Fanconi anemia, complementation group D2															
NM_002880	RAF1	chr3	12600099	12680700	-	Onc	v-raf-1 murine leukemia viral oncogene homolog 1															
NM_133625	SYN2	chr3	12020861	12208532	+	--	synapsin II SYN2: Approved										12112559	2159_73	+			
NM_001128176	THRB	chr3	24133648	24511317	-	Onc	thyroid hormone receptor, beta (erythroblastic leukemia viral (v-erb-a) oncogene homolog 2, avian)															
NM_016152	RARB	chr3	25444757	25614426	+	Tum Sup	retinoic acid receptor, beta															
NM_002295	RPSA	chr3	39423207	39429036	+	Other	ribosomal protein SA															
NM_007335	DLEC1	chr3	38055699	38139232	+	Tum Sup	deleted in lung and esophageal cancer 1															
NR_024071	PLCD1	chr3	38023990	38041282	-	Tum Sup	phospholipase C, delta 1															
NM_003242	TGFBR2	chr3	30622997	30710637	+	Tum Sup	transforming growth factor, beta receptor II (70/80kDa)															
NM_004656	BAP1	chr3	52410064	52419049	-	Tum Sup	BRCA1 associated protein-1 (ubiquitin carboxy-terminal hydrolase)															
NM_014240	LIMD1	chr3	45611326	45697759	+	Tum Sup	LIM domains containing 1				45693385	AY516750	+									
NM_001167617	MLH1	chr3	37010271	37067341	+	Tum Sup	mutL homolog 1, colon cancer, nonpolyposis type 2 (E. coli)															
NR_001566	TERC	chr3	170965091	170965542	-	Onc	telomerase RNA component															

Table S1

tumor associated genes								HIV			MLV			MMTV			MMTV(SIN)			MMTV(SIN)arrest		
RefSeq	gene symbol	Chrom	txStart	txEnd	stra	Category	Gene name	position	integrant	strand	position	integrant	stran	positiopn	integrant	stran	position	integrant	stran	position	integrant	stran
NM_006217	SERPINI2	chr3	168642416	168674512	-	Tum Sup	serpin peptidase inhibitor, clade I (pancpin), member 2															
NM_020390	EIF5A2	chr3	172088897	172109120	-	Onc	eukaryotic translation initiation factor 5A2															
NM_020640	DCUN1D1	chr3	184143252	184181020	-	Onc	DCN1, defective in cullin neddylation 1, domain containing 1 (S. cerevisiae)															
NM_006218	PIK3CA	chr3	180349004	180435191	+	Onc	phosphoinositide-3-kinase, catalytic, alpha polypeptide															
NM_002740	PRKCI	chr3	171422913	171506464	+	Onc	protein kinase C, iota															
NM_014398	LAMP3	chr3	184322696	184363361	-	Other	lysosomal-associated membrane protein 3															
NM_003106	SOX2	chr3	182912405	182914917	+	--	SRY (sex determining region Y)-box 2 SOX2: Approved															
NM_001134738	BCL6	chr3	188921858	188935389	-	Onc	B-cell CLL/lymphoma 6 (zinc finger protein 51)															
NM_001177598	THPO	chr3	185572466	185578626	-	Onc	thrombopoietin (myeloproliferative leukemia virus oncogene ligand, megakaryocyte growth and developm															
NM_001204388	DLG1	chr3	198253827	198395399	-	Tum Sup	discs, large homolog 1 (Drosophila)															
NM_022965	FGFR3	chr4	1764836	1780397	+	Onc	fibroblast growth factor receptor 3 (achondroplasia, thanatophoric dwarfism)															
NM_006342	TACC3	chr4	1693014	1716703	+	Other	transforming, acidic coiled-coil containing protein 3															
NM_133335	WHSC1	chr4	1864306	1953732	+	Onc	Wolf-Hirschhorn syndrome candidate 1															
NM_001130087	ABLIM2	chr4	8017936	8211459	-	Tum Sup	actin binding LIM protein family, member 2 (Previous name: actin binding LIM protein 2)															
NM_001328	CTBP1	chr4	1195227	1232908	-	Tum Sup	C-terminal binding protein 1															
NM_004787	SLIT2	chr4	19864332	20229886	+	Tum Sup	slit homolog 2 (Drosophila) (Previous name: slit (Drosophila) homolog 2)													20209426	[illegible]2511_217_[illegible]	+
NM_004310	RHOH	chr4	39874921	39922676	+	Other	ras homolog gene family, member H															

Table S1

tumor associated genes								HIV			MLV			MMTV			MMTV(SIN)			MMTV(SIN)arrest		
RefSeq	gene symbol	Chrom	txStart	txEnd	stra	Category	Gene name	position	integrant	strand	position	integrant	stran	positiopn	integrant	stran	position	integrant	stran	position	integrant	stran
NM_003215	TEC	chr4	47832556	47966571	-	Onc	tec protein tyrosine kinase															
NM_001093772	KIT	chr4	55218851	55301638	+	Onc	v-kit Hardy-Zuckerman 4 feline sarcoma viral oncogene homolog															
NM_001553	IGFBP7	chr4	57591993	57671308	-	Tum Sup	insulin-like growth factor binding protein 7															
NM_006206	PDGFRA	chr4	54790020	54859169	+	Onc	platelet-derived growth factor receptor, alpha polypeptide															
NM_001511	CXCL1	chr4	74953972	74955883	+	Onc	chemokine (C-X-C motif) ligand 1 (melanoma growth stimulating activity, alpha)															
NM_001079911	DMP1	chr4	88790477	88804536	+	Tum Sup	dentin matrix acidic phosphoprotein 1 (Previous name: dentin matrix acidic phosphoprotein)															
NM_004464	FGF5	chr4	81406765	81431195	+	Onc	fibroblast growth factor 5															
NM_005935	MLLT2	chr4	88147177	88281215	+	Onc	AF4/FMR2 family, member 1															
NM_001098540	HPSE	chr4	84432637	84475058	-	Other	heparanase															
NM_080683	PTPN13	chr4	87734491	87955352	+	Tum Sup	protein tyrosine phosphatase, non-receptor type 13 (APO-1/CD95 (Fas)-associated phosphatase)							87909221	_174_1_15	+						
NM_001237	CCNA2	chr4	122957048	122964538	-	Onc	cyclin A2															
NM_002006	FGF2	chr4	123967312	124038840	+	Onc	fibroblast growth factor 2 (basic)															
NM_005651	TDO2	chr4	157044294	157061008	+	--	tryptophan 2,3-dioxygenase TDO2: Approved							157056546	_293_1_18	+						
NM_001257069	FBXW7	chr4	153551761	153675843	-	Tum Sup	F-box and WD repeat domain containing 7 (Previous names: F-box and WD-40 domain protein 7 (archipela															
NM_012403	ANP32C	chr4	165337608	165338313	-	Other	acidic (leucine-rich) nuclear phosphoprotein 32 family, member C															
NM_002199	IRF2	chr4	185545869	185632720	-	Onc	interferon regulatory factor 2															
NM_005245	FAT	chr4	187745931	187881981	-	Tum Sup	FAT tumor suppressor homolog 1 (Drosophila)															

Table S1

tumor associated genes								HIV			MLV			MMTV			MMTV(SIN)			MMTV(SIN)arrest		
RefSeq	gene symbol	Chrom	txStart	txEnd	stra	Category	Gene name	position	integrant	strand	position	integrant	stran	positiopn	integrant	stran	position	integrant	stran	position	integrant	stran
NM_001564	ING2	chr4	184663213	184669243	+	Tum Sup	inhibitor of growth family, member 2 (Previous names: inhibitor of growth family, member 1-like)															
NM_007118	TRIO	chr5	14196828	14562458	+	Onc	triple functional domain (PTPRF interacting)															
NM_001244871	DAB2	chr5	39407532	39461092	-	Tum Sup	disabled homolog 2, mitogen-responsive phosphoprotein (Drosophila)															
NM_001243120	SKP2	chr5	36187901	36219899	+	Other	S-phase kinase-associated protein 2 (p45)															
NM_003711	PPAP2A	chr5	54756439	54866630	-	Tum Sup	phosphatidic acid phosphatase type 2A															
NM_002439	MSH3	chr5	79986222	80208390	+	Other	mutS homolog 3 (E. coli)				80204900	AY516272	+							80022750	540_109_1	+
NM_018695	ERBB2IP	chr5	65258137	65412607	+	Other	erbb2 interacting protein															
NR_046318	ENC1	chr5	73958986	73973005	-	Tum Sup	ectodermal-neural cortex 1 (with BTB-like domain)															
NM_001270	CHD1	chr5	98218807	98290138	-	--	chromodomain helicase DNA binding protein 1 CHD1: Approved	98250537	CL529541	-												
NM_001270	CHD1	chr5	98218807	98290138	-	--	chromodomain helicase DNA binding protein 1 CHD1: Approved	98219425	CL800066	-												
NM_005246	FER	chr5	108111421	108551272	+	Onc	fer (fps/fes related) tyrosine kinase (phosphoprotein NCP94)															
NM_002387	MCC	chr5	112385694	112658511	-	& -- & Tum Sup	mutated in colorectal cancers MCC: Approved															
NM_000038	APC	chr5	112101454	112209835	+	Tum Sup	adenomatosis polyposis coli															
NM_003135	SRP19	chr5	112224892	112231503	+	Other	signal recognition particle 19kDa															
NM_002084	GPX3	chr5	150380191	150388747	+	Tum Sup	glutathione peroxidase 3 (plasma)															
NM_001178102	LOX	chr5	121426788	121440817	-	Tum Sup	lysyl oxidase															
NM_014423	AFF4	chr5	132238969	132327253	-	Other	AF4/FMR2 family, member 4															
NM_000358	TGFBI	chr5	135392482	135427406	+	Tum Sup	transforming growth factor, beta-induced, 68kDa															

Table S1

tumor associated genes								HIV			MLV			MMTV			MMTV(SIN)			MMTV(SIN)arrest		
RefSeq	gene symbol	Chrom	txStart	txEnd	stra	Category	Gene name	position	integrant	strand	position	integrant	stran	positiopn	integrant	stran	position	integrant	stran	position	integrant	stran
NM_001964	EGR1	chr5	137829079	137832903	+	Tum Sup	early growth response 1															
NM_002198	IRF1	chr5	131845199	131854364	-	Tum Sup	interferon regulatory factor 1															
NM_001131027	PDLIM4	chr5	131621249	131637046	+	Tum Sup	PDZ and LIM domain 4															
NM_002715	PPP2CA	chr5	133560046	133589849	-	Other	protein phosphatase 2 (formerly 2A), catalytic subunit, alpha isoform															
NR_024610	HINT1	chr5	130522773	130528933	-	Tum Sup	histidine triad nucleotide binding protein 1 (Previous name: histidine triad nucleotide-binding prot															
NM_032420	PCDH1	chr5	141212856	141238128	-	--	protocadherin 1 (Previous name: protocadherin 1 (cadherin-like 1)) PCDH1: Approved															
NM_014945	ABLIM3	chr5	148501246	148620192	+	Tum Sup	actin binding LIM protein family, member 3															
NM_005211	CSF1R	chr5	149413046	149473128	-	Onc	colony stimulating factor 1 receptor, formerly McDonough feline sarcoma viral (v-fms) oncogene homol															
NM_002609	PDGFRB	chr5	149473594	149515615	-	Onc	platelet-derived growth factor receptor, beta polypeptide															
NM_001142557	HMMR	chr5	162820094	162851530	+	Onc	hyaluronan-mediated motility receptor (RHAMM)															
NM_003900	SQSTM1	chr5	179180447	179197683	+	--	sequestosome 1 (Previous names: Paget disease of bone 3, oxidative stress induced like) SQSTM1: App															
NM_004219	PTTG1	chr5	159781442	159788324	+	Onc	pituitary tumor-transforming 1															
NM_020185	DUSP22	chr6	237100	296355	+	Tum Sup	dual specificity phosphatase 22															
NM_001195286	IRF4	chr6	336738	356443	+	Onc	interferon regulatory factor 4															
NM_001032280	TFAP2A	chr6	10504901	10520593	-	Other	transcription factor AP-2 alpha (activating enhancer binding protein 2 alpha)															
NM_001134709	DEK	chr6	18332378	18372778	-	Onc	DEK oncogene (DNA binding)															
NM_006510	TRIM27	chr6	28978758	28999747	-	Onc	ret finger protein															

Table S1

tumor associated genes								HIV			MLV			MMTV			MMTV(SIN)			MMTV(SIN)arrest		
RefSeq	gene symbol	Chrom	txStart	txEnd	stra	Category	Gene name	position	integrant	strand	position	integrant	stran	positiopn	integrant	stran	position	integrant	stran	position	integrant	stran
NM_001202522	DDR1	chr6	30964443	30975912	+	--	discoidin domain receptor tyrosine kinase 1 (Previous names: discoidin domain receptor family, membe															
NM_001164239	DHX16	chr6	30728874	30748809	-	Other	DEAH (Asp-Glu-Ala-His) box polypeptide 16															
NM_000594	TNF	chr6	31651322	31654091	+	--	tumor necrosis factor (Previous names: tumor necrosis factor (TNF superfamily, member 2)) TNF: Appr															
NM_001220778	CDKN1A	chr6	36754464	36763095	+	Tum Sup	cyclin-dependent kinase inhibitor 1A (p21, Cip1)															
NM_002648	PIM1	chr6	37245899	37251182	+	Onc	pim-1 oncogene															
NM_014780	CUL7	chr6	43113332	43129661	-	Onc	cullin 7 (Previous name: KIAA0076)															
NM_001253	CDC5L	chr6	44463228	44526139	+	Onc	CDC5 cell division cycle 5-like (S. pombe) (Previous names: CDC5 (cell division cycle 5, S. pombe, h															
NM_001024630	RUNX2	chr6	45404031	45626797	+	Onc	runt-related transcription factor 2	45414391	CL800133	-	45523382	AY515890	+									
NM_006929	SKIV2L	chr6	32034559	32045511	+	Other	superkiller viralicidic activity 2-like (S. cerevisiae)															
NM_001171624	VEGFA	chr6	43845923	43862201	+	Onc	vascular endothelial growth factor A (Previous name: vascular endothelial growth factor)															
NM_016277	RAB23	chr6	57161540	57195037	-	Onc	RAB23, member RAS oncogene family															
NM_001704	BAI3	chr6	69402352	70156124	+	Tum Sup	brain-specific angiogenesis inhibitor 3										69766805	330_391	+			
NM_003463	PTP4A1	chr6	64339878	64351448	+	Other	protein tyrosine phosphatase type IVA, member 1															
NM_001170794	BACH2	chr6	90692967	91063348	-	Tum Sup	BTB and CNC homology 1, basic leucine zipper transcription factor 2															
NM_020771	HACE1	chr6	105282660	105414487	-	Tum Sup	HECT domain and ankyrin repeat containing, E3 ubiquitin protein ligase 1															
NM_002037	FYN	chr6	112088227	112301348	-	Onc	FYN oncogene related to SRC, FGR, YES	112242335	AY516916.[illegible]	+	112184413	AY516769	-									

Table S1

tumor associated genes								HIV			MLV			MMTV			MMTV(SIN)			MMTV(SIN)arrest		
RefSeq	gene symbol	Chrom	txStart	txEnd	stra	Category	Gene name	position	integrant	strand	position	integrant	stran	positiopn	integrant	stran	position	integrant	stran	position	integrant	stran
NM_002037	FYN	chr6	112088227	112301348	-	Onc	FYN oncogene related to SRC, FGR, YES	112279291	AY517356.1	-												
NM_002912	REV3L	chr6	111726926	111911107	-	Tum Sup	REV3-like, catalytic subunit of DNA polymerase zeta (yeast) (Previous names: REV3 (yeast homolog)-li															
NM_198239	WISP3	chr6	112482063	112497580	+	Onc	WNT1 inducible signaling pathway protein 3															
NM_001198	PRDM1	chr6	106640887	106664507	+	Tum Sup	PR domain containing 1, with ZNF domain															
NM_002944	ROS1	chr6	117716222	117853711	-	Onc	v-ros UR2 sarcoma virus oncogene homolog 1 (avian)										117852470	686_128	-			
NM_001161659	MYB	chr6	135544145	135582004	+	Onc	v-myb myeloblastosis viral oncogene homolog (avian)	135553836	AY516946.1	-										135547863	606_112_1	+
NM_001003395	TPD52L1	chr6	125517118	125626343	+	Other	tumor protein D52-like 1															
NM_001135648	PTPRK	chr6	128331616	128883563	-	Tum Sup	protein tyrosine phosphatase, receptor type, K	128453385	BH609871	+	128335745	AY515943	-				128741180	2051_116	-			
NM_005923	MAP3K5	chr6	136919879	137155349	-	--	mitogen-activated protein kinase kinase kinase 5 MAP3K5: Approved															
NM_001901	CTGF	chr6	132311009	132314211	-	Onc	connective tissue growth factor															
NM_001134830	AHI1	chr6	135646802	135860596	-	Onc	Abelson helper integration site 1 (Previous name: Abelson helper integration site)	135794109	AY516959.1	+										135849823	824_167_	-
NM_005100	AKAP12	chr6	151602826	151721390	+	Tum Sup	A kinase (PRKA) anchor protein 12 (Previous name: A kinase (PRKA) anchor protein (gravin) 12)															
NM_001080954	PLAGL1	chr6	144303129	144371234	-	Tum Sup	pleiomorphic adenoma gene-like 1															
NR_073033	LATS1	chr6	150020981	150079254	-	Tum Sup	LATS, large tumor suppressor, homolog 1 (Drosophila)															
NM_001122741	ESR1	chr6	152168500	152466101	+	Onc	estrogen receptor 1															
NM_002377	MAS1	chr6	160247963	160249097	+	Onc	MAS1 oncogene															
NM_000876	IGF2R	chr6	160310120	160447573	+	Tum Sup	insulin-like growth factor 2 receptor															
NM_007045	FGFR1OP	chr6	167332805	167374056	+	Onc	FGFR1 oncogene partner	167348509	CL800162	+												

Table S1

tumor associated genes								HIV			MLV			MMTV			MMTV(SIN)			MMTV(SIN)arrest		
RefSeq	gene symbol	Chrom	txStart	txEnd	stra	Category	Gene name	position	integrant	strand	position	integrant	stran	positiopn	integrant	stran	position	integrant	stran	position	integrant	stran
NM_007045	FGFR1OP	chr6	167332805	167374056	+	Onc	FGFR1 oncogene partner	167346459	CL528995	+												
NM_001040000	MLLT4	chr6	167970519	168108642	+	Other	myeloid/lymphoid or mixed-lineage leukemia (trithorax homolog, Drosophila); translocated to, 4															
NM_007353	GNA12	chr7	2734266	2850485	-	Onc	guanine nucleotide binding protein (G protein) alpha 12															
NR_003085	PMS2	chr7	5979395	6015263	-	Other	PMS2 postmeiotic segregation increased 2 (S. cerevisiae)															
NM_001163152	ETV1	chr7	13897380	13992664	-	Onc	ets variant gene 1															
NM_000474	TWIST1	chr7	19121615	19123820	-	Onc	twist homolog 1 (Drosophila) (Previous names: blepharophimosis, epicanthus inversus and ptosis 3, ac															
NM_014399	TSPAN13	chr7	16759875	16790686	+	Tum Sup	tetraspanin 13 (Previous name: transmembrane 4 superfamily member 13)															
NR_037939	HOXA10	chr7	27176734	27186405	-	Onc	homeobox A10															
NM_152739	HOXA9	chr7	27168581	27171674	-	Other	homeobox A9															
NM_005402	RALA	chr7	39629676	39714248	+	Onc	v-ral simian leukemia viral oncogene homolog A (ras related)															
NM_001127454	DFNA5	chr7	24704498	24764164	-	Tum Sup	deafness, autosomal dominant 5															
NM_003014	SFRP4	chr7	37912059	37923050	-	Other	secreted frizzled-related protein 4															
NM_005228	EGFR	chr7	55054218	55242525	+	Onc	epidermal growth factor receptor (erythroblastic leukemia viral (v-erb-b) oncogene homolog, avian)				55109025	AY516339	+									
NM_001131009	PTPN12	chr7	77005287	77107324	+	Tum Sup	protein tyrosine phosphatase, non-receptor type 12															
NM_194455	KRIT1	chr7	91666218	91713350	-	Tum Sup	KRIT1, ankyrin repeat containing															
NM_001202855	CYP3A4	chr7	99192518	99219747	-	Other	cytochrome P450, family 3, subfamily A, polypeptide 4															

Table S1

tumor associated genes								HIV			MLV			MMTV			MMTV(SIN)			MMTV(SIN)arrest		
RefSeq	gene symbol	Chrom	txStart	txEnd	stra	Category	Gene name	position	integrant	strand	position	integrant	stran	positiopn	integrant	stran	position	integrant	stran	position	integrant	stran
NM_000601	HGF	chr7	81169379	81237388	-	Onc	hepatocyte growth factor (hepapoietin A; scatter factor) (Previous names: deafness, autosomal recess	81225868	CL529008	-	81172672	AY516177	-									
NM_012431	SEMA3E	chr7	82831157	83116415	-	Other	sema domain, immunoglobulin domain (Ig), short basic domain, secreted, (semaphorin) 3E															
NM_003496	TRRAP	chr7	98314048	98448802	+	Other	transformation/transcription domain-associated protein															
NM_145032	FBXL13	chr7	102240543	102502251	-	Tum Sup	F-box and leucine-rich repeat protein 13							102395361	_GVJ7CIC(	-						
NM_005824	LRRC17	chr7	102340579	102372792	+	Other	leucine rich repeat containing 17															
NM_203397	MBLAC1	chr7	99562255	99564057	+	--	metallo-beta-lactamase domain containing 1 MBLAC1: Approved															
NM_001122838	NAPEPLD	chr7	102527259	102576805	-	Tum Sup	N-acyl-phosphatidylethanolamine-hydrolyzing phospholipase D															
NM_031905	ARMC10	chr7	102502564	102527441	+	Tum Sup	SVH protein															
NM_019071	ING3	chr7	120378052	120402947	+	Tum Sup	inhibitor of growth family, member 3															
NM_001127500	MET	chr7	116099694	116225676	+	Onc	met proto-oncogene (hepatocyte growth factor receptor)															
NM_000111	SLC26A3	chr7	107193147	107230914	-	Tum Sup	solute carrier family 26, member 3															
NM_001172895	CAV1	chr7	115952298	115988475	+	& -- & Tum Sup	caveolin 1, caveolae protein, 22kDa															
NM_001206748	CAV2	chr7	115927333	115935831	+	Tum Sup	caveolin 2															
NM_001193584	NRCAM	chr7	107575306	107884077	-	Tum Sup	neuronal cell adhesion molecule													107864478	3336_390_	-
NM_005631	SMO	chr7	128615948	128640621	+	Tum Sup	smoothened homolog (Drosophila)															
NM_005435	ARHGEF5	chr7	143683421	143708658	+	Onc	Rho guanine nucleotide exchange factor (GEF) 5															
NM_004333	BRAF	chr7	140080281	140271033	-	Onc	v-raf murine sarcoma viral oncogene homolog B1															
NM_000193	SHH	chr7	155288318	155297728	-	Other	sonic hedgehog homolog (Drosophila)															

Table S1

tumor associated genes								HIV			MLV			MMTV			MMTV(SIN)			MMTV(SIN)arrest		
RefSeq	gene symbol	Chrom	txStart	txEnd	stra	Category	Gene name	position	integrant	strand	position	integrant	stran	positiopn	integrant	stran	position	integrant	stran	position	integrant	stran
NM_003592	CUL1	chr7	148026865	148129135	+	Tum Sup	cullin 1															
NM_033225	CSMD1	chr8	2780281	4839736	-	Tum Sup	CUB and Sushi multiple domains 1													3414644	513_107_1	-
NM_002052	GATA4	chr8	11599125	11654918	+	Onc	GATA binding protein 4															
NM_019851	FGF20	chr8	16894704	16904045	-	Onc	fibroblast growth factor 20															
NM_006207	PDGFRL	chr8	17478214	17544922	+	Tum Sup	platelet-derived growth factor receptor-like															
NM_024767	DLC1	chr8	13206486	13416800	-	Tum Sup	deleted in liver cancer 1															
NM_000237	LPL	chr8	19840861	19869050	+	Tum Sup	lipoprotein lipase															
NM_001001924	MTUS1	chr8	17545582	17702706	-	Tum Sup	microtubule associated tumor suppressor 1 (Previous name: mitochondrial tumor suppressor 1)															
NM_006765	TUSC3	chr8	15441966	15668529	+	Tum Sup	tumor suppressor candidate 3															
NM_001160037	RHOBTB2	chr8	22909305	22933655	+	Tum Sup	Rho-related BTB domain containing 2															
NM_004331	BNIP3L	chr8	26296439	26326561	+	Tum Sup	BCL2/adenovirus E1B 19kDa interacting protein 3-like															
NM_001440	EXTL3	chr8	28615071	28667121	+	Tum Sup	exostoses (multiple)-like 3															
NM_006167	NKX3-1	chr8	23592150	23596395	-	Other	NK3 transcription factor related, locus 1 (Drosophila)															
NM_182826	SCARA3	chr8	27547495	27590205	+	Tum Sup	scavenger receptor class A, member 3															
NM_024025	DUSP26	chr8	33568392	33576981	-	Onc	dual specificity phosphatase 26 (putative)															
NM_005195	CEBPD	chr8	48812028	48813279	-	Tum Sup	CCAAT/enhancer binding protein (C/EBP), delta															
NM_023105	FGFR1	chr8	38387812	38445509	-	Onc	fibroblast growth factor receptor 1 (fms-related tyrosine kinase 2, Pfeiffer syndrome)															
NM_006283	TACC1	chr8	38763878	38829703	+	Other	transforming, acidic coiled-coil containing protein 1															
NM_005372	MOS	chr8	57188054	57189095	-	Onc	v-mos Moloney murine sarcoma viral oncogene homolog															

Table S1

tumor associated genes								HIV			MLV			MMTV			MMTV(SIN)			MMTV(SIN)arrest		
RefSeq	gene symbol	Chrom	txStart	txEnd	stra	Category	Gene name	position	integrant	strand	position	integrant	stran	positiopn	integrant	stran	position	integrant	stran	position	integrant	stran
NM_001111097	LYN	chr8	56954939	57087560	+	Onc	v-yes-1 Yamaguchi sarcoma viral related oncogene homolog															
NM_006837	COPS5	chr8	68117868	68137116	-	Onc	COP9 constitutive photomorphogenic homolog subunit 5 (Arabidopsis) (Previous names: COP9 (constituti															
NM_001025253	TPD52	chr8	81109659	81246391	-	Onc	tumor protein D52															
NM_001205263	RAD54B	chr8	95453363	95518356	-	Other	RAD54 homolog B (S. cerevisiae)															
NM_001080416	MYBL1	chr8	67636963	67688034	-	Onc	v-myb myeloblastosis viral oncogene homolog (avian)-like 1															
NM_175635	RUNX1T1	chr8	93036370	93176666	-	Onc	runt-related transcription factor 1; translocated to, 1 (cyclin D-related)															
NM_033285	TP53INP1	chr8	96007375	96030791	-	Tum Sup	tumor protein p53 inducible nuclear protein 1															
NM_057749	CCNE2	chr8	95961628	95976658	-	Other	cyclin E2															
NR_003367	PVT1	chr8	128972055	129182681	+	Onc	Pvt1 oncogene homolog, MYC activator (mouse)															
NM_002514	NOV	chr8	120497732	120505859	+	Onc	nephroblastoma overexpressed gene															
NM_003882	WISP1	chr8	134272463	134313114	+	Onc	WNT1 inducible signaling pathway protein 1															
NM_000127	EXT1	chr8	118880782	119193239	-	Tum Sup	exostoses (multiple) 1	119170173	CL529513	+	119098953	AY516196	-									
NM_002467	MYC	chr8	128817496	128822862	+	Onc	v-myc myelocytomatosis viral oncogene homolog (avian)															
NM_016601	KCNK9	chr8	140693985	140784481	-	Onc	potassium channel, subfamily K, member 9															
NM_001258433	NDRG1	chr8	134318595	134378729	-	Tum Sup	N-myc downstream regulated gene 1															
NM_130393	PTPRD	chr9	8304245	8723946	-	Tum Sup	protein tyrosine phosphatase, receptor type, D															
NM_024013	IFNA1	chr9	21430452	21431315	+	Tum Sup	interferon, alpha 1															
NM_004529	MLLT3	chr9	20334967	20612514	-	Onc	myeloid/lymphoid or mixed-lineage leukemia (trithorax homolog, Drosophila); translocated to, 3				20598051	AY516371	-				20485907	s1701_266	-			

Table S1

tumor associated genes								HIV			MLV			MMTV			MMTV(SIN)			MMTV(SIN)arrest		
RefSeq	gene symbol	Chrom	txStart	txEnd	stra	Category	Gene name	position	integrant	strand	position	integrant	strand	positiopn	integrant	strand	position	integrant	strand	position	integrant	strand
NM_004529	MLLT3	chr9	20334967	20612514	-	Onc	myeloid/lymphoid or mixed-lineage leukemia (trithorax homolog, Drosophila); translocated to, 3				20610185	AY516195	-									
NM_001195132	CDKN2A	chr9	21957750	21965132	-	Tum Sup	cyclin-dependent kinase inhibitor 2A (melanoma, p16, inhibits CDK4)															
NM_078487	CDKN2B	chr9	21992901	21999312	-	Tum Sup	cyclin-dependent kinase inhibitor 2B (p15, inhibits CDK4)															
NM_004629	FANCG	chr9	35063834	35070013	-	Tum Sup	Fanconi anemia, complementation group G															
NM_016734	PAX5	chr9	36828530	37024476	-	Onc	paired box gene 5 (B-cell lineage specific activator)															
NM_000264chr9	PTCH	chr9	97245085	97310652	-	Tum Sup	patched homolog (Drosophila)															
NM_006401	ANP32B	chr9	99785309	99818045	+	Other	acidic (leucine-rich) nuclear phosphoprotein 32 family, member B															
NM_004235	KLF4	chr9	109286953	109291868	-	Onc	Kruppel-like factor 4 (gut)															
NM_002829	PTPN3	chr9	111177794	111300414	-	Tum Sup	protein tyrosine phosphatase, non-receptor type 3															
NM_017418	DEC1	chr9	116943917	117204744	+	Tum Sup	deleted in esophageal cancer 1															
NM_005421	TAL2	chr9	107464558	107465206	+	Other	T-cell acute lymphocytic leukemia 2															
NM_006195	PBX3	chr9	127549437	127769476	+	Other	pre-B-cell leukemia transcription factor 3															
NM_004789	LHX2	chr9	125813709	125835263	+	Other	LIM homeobox 2															
NM_012210	TRIM32	chr9	118489401	118503400	+	Onc	tripartite motif-containing 32 (Previous name: limb girdle muscular dystrophy 2H (autosomal recessiv															
NM_005564	LCN2	chr9	129951552	129955555	+	Onc	lipocalin 2 (oncogene 24p3)															
NM_003011	SET	chr9	130491329	130498496	+	Onc	SET translocation (myeloid leukemia-associated)															
NM_021138	TRAF2	chr9	138900785	138940888	+	--	TNF receptor-associated factor 2 TRAF2: Approved															
NM_001162427	TSC1	chr9	134756555	134809841	-	Tum Sup	tuberous sclerosis 1															

Table S1

tumor associated genes								HIV			MLV			MMTV			MMTV(SIN)			MMTV(SIN)arrest		
RefSeq	gene symbol	Chrom	txStart	txEnd	stra	Category	Gene name	position	integrant	strand	position	integrant	stran	positiopn	integrant	stran	position	integrant	stran	position	integrant	stran
NM_007313	ABL1	chr9	132579088	132752883	+	Onc	v-abl Abelson murine leukemia viral oncogene homolog 1															
NM_004938	DAPK1	chr9	89302575	89513369	+	Tum Sup	death-associated protein kinase 1															
NM_005085	NUP214	chr9	132990801	133098912	+	Other	nucleoporin 214kDa										133046238	9_1_474_1	+			
NM_003371	VAV2	chr9	135616836	135847267	-	Onc	vav 2 oncogene															
NM_004188	GFI1B	chr9	134843918	134856905	+	Other	growth factor independent 1B (potential regulator of CDKN1A, translocated in CML)															
NM_001144026	NDOR1	chr9	139219939	139233634	+	Other	NADPH dependent diflavin oxidoreductase 1															
NM_001160124	KLF6	chr10	3808187	3817473	-	Tum Sup	Kruppel-like factor 6 (Previous name: core promoter element binding protein)															
NM_001047160	NET1	chr10	5444513	5491019	+	Onc	neuroepithelial cell transforming gene 1															
NM_012425	RSU1	chr10	16672622	16899459	-	Other	Ras suppressor protein 1															
NM_012342	BAMBI	chr10	29006429	29011874	+	Other	BMP and activin membrane-bound inhibitor homolog (Xenopus laevis)															
NM_005204	MAP3K8	chr10	30762955	30790768	+	Onc	mitogen-activated protein kinase kinase kinase 8															
NM_005180	PCGF4	chr10	22645305	22660192	+	Onc	polycomb group ring finger 4															
NM_003591	CUL2	chr10	35337484	35419317	-	Tum Sup	cullin 2															
NM_001040084	ANXA8	chr10	46577989	46594128	-	Other	annexin A8															
NM_001145260	NCOA4	chr10	51235113	51260740	+	Other	nuclear receptor coactivator 4															
NM_020630	RET	chr10	42892522	42942958	+	Onc	ret proto-oncogene (multiple endocrine neoplasia and medullary thyroid carcinoma 1, Hirschsprung dis															
NM_002750	MAPK8	chr10	49279692	49313189	+	Onc	mitogen-activated protein kinase 8															
NM_014000	VCL	chr10	75427877	75549920	+	Tum Sup	vinculin															
NM_005398	PPP1R3C	chr10	93378176	93382838	-	Tum Sup	protein phosphatase 1, regulatory (inhibitor) subunit 3C															

Table S1

tumor associated genes								HIV			MLV			MMTV			MMTV(SIN)			MMTV(SIN)arrest		
RefSeq	gene symbol	Chrom	txStart	txEnd	stra	Category	Gene name	position	integrant	strand	position	integrant	stran	positiopn	integrant	stran	position	integrant	stran	position	integrant	stran
NM_003087	SNCG	chr10	88708267	88712997	+	Other	synuclein, gamma (breast cancer-specific protein 1)															
NM_000314	PTEN	chr10	89613174	89718512	+	Tum Sup	phosphatase and tensin homolog (mutated in multiple advanced cancers 1)															
NM_018131	CEP55	chr10	95246378	95278839	+	Onc	centrosomal protein 55kDa (Previous name: chromosome 10 open reading frame 3)															
NM_033164	FGF8	chr10	103519876	103525749	-	Onc	fibroblast growth factor 8 (androgen-induced)															
NM_005097	LGI1	chr10	95507555	95547906	+	Tum Sup	leucine-rich, glioma inactivated 1															
NM_032429	LZTS2	chr10	102746853	102757583	+	Tum Sup	leucine zipper, putative tumor suppressor 2															
NM_002502	NFKB2	chr10	104145416	104152276	+	Onc	nuclear factor of kappa light polypeptide gene enhancer in B-cells 2 (p49/p100)															
NM_003988	PAX2	chr10	102495457	102579688	+	Onc	paired box gene 2										102521038	5_1_2636_[illegible]	+			
NM_005521	TLX1	chr10	102881050	102887536	+	Onc	T-cell leukemia homeobox 1															
NM_130439	MXI1	chr10	111957352	112037113	+	Tum Sup	MAX interactor 1															
NM_005479	FRAT1	chr10	99069011	99071662	+	& -- & Onc	frequently rearranged in advanced T-cell lymphomas															
NM_012083	FRAT2	chr10	99082243	99084448	-	Onc	frequently rearranged in advanced T-cell lymphomas 2															
NM_003015	SFRP5	chr10	99516497	99521746	-	Tum Sup	secreted frizzled-related protein 5															
NM_005029	PITX3	chr10	103979935	103991221	-	--	paired-like homeodomain 3 (Previous name: paired-like homeodomain transcription factor 3) PITX3: Ap															
NM_005445	CSPG6	chr10	112317439	112354382	+	Other	chondroitin sulfate proteoglycan 6 (bamacan)															
NM_004210	NEURL	chr10	105243724	105342299	+	Tum Sup	neuralized-like (Drosophila)															
NM_017579	DMBT1	chr10	124310170	124393242	+	Tum Sup	deleted in malignant brain tumors 1															

Table S1

tumor associated genes								HIV			MLV			MMTV			MMTV(SIN)			MMTV(SIN)arrest		
RefSeq	gene symbol	Chrom	txStart	txEnd	stra	Category	Gene name	position	integrant	strand	position	integrant	stran	positiopn	integrant	stran	position	integrant	stran	position	integrant	stran
NM_022970	FGFR2	chr10	123227833	123347962	-	Onc	fibroblast growth factor receptor 2 (bacteria-expressed kinase, keratinocyte growth factor receptor,															
NM_206860	TACC2	chr10	123913094	124004047	+	Other	transforming, acidic coiled-coil containing protein 2															
NM_022802	CTBP2	chr10	126666407	126706443	-	& -- & Tum Sup	C-terminal binding protein 2															
NM_000076	CDKN1C	chr11	2861023	2863571	-	Tum Sup	cyclin-dependent kinase inhibitor 1C (p57, Kip2)															
NM_176795	HRAS	chr11	522241	525550	-	Onc	v-Ha-ras Harvey rat sarcoma viral oncogene homolog															
NM_002458	MUC5B	chr11	1200870	1239982	+	--	mucin 5B, oligomeric mucus/gel-forming (Previous names: mucin 5, subtype B, tracheobronchial) MUC5B															
NM_139131	NUP98	chr11	3689634	3775598	-	Other	nucleoporin 98kDa													3745576	1738_162_	-
NM_002555	SLC22A18	chr11	2880087	2903052	+	Tum Sup	solute carrier family 22 (organic cation transporter), member 18															
NM_139022	TSPAN32	chr11	2279818	2296006	+	Tum Sup	tetraspanin 32															
NM_005706	TSSC4	chr11	2380098	2381682	+	Tum Sup	tumor suppressing subtransferable candidate 4															
NM_145040	PRKCDBP	chr11	6296751	6298316	-	Tum Sup	protein kinase C, delta binding protein															
NM_021961	TEAD1	chr11	12652544	12922860	+	Onc	TEA domain family member 1 (SV40 transcriptional enhancer factor)							12686687	_351_1_21	+						
NM_004179	TPH1	chr11	17998659	18018911	-	--	tryptophan hydroxylase 1 (Previous name: tryptophan hydroxylase (tryptophan 5-monooxygenase)) TPH1:															
NM_013253	DKK3	chr11	11941118	11987493	-	Tum Sup	dickkopf homolog 3 (Xenopus laevis) (Previous name: dickkopf (Xenopus laevis) homolog 3)															

Table S1

tumor associated genes								HIV			MLV			MMTV			MMTV(SIN)			MMTV(SIN)arrest		
RefSeq	gene symbol	Chrom	txStart	txEnd	stra	Category	Gene name	position	integrant	strand	position	integrant	strand	positiopn	integrant	strand	position	integrant	strand	position	integrant	strand
NM_001177314	RRAS2	chr11	14256041	14342628	-	Other	related RAS viral (r-ras) oncogene homolog 2													14284166	[illegible]763_163_[illegible]	-
NM_006410	HTATIP2	chr11	20341806	20361905	+	Tum Sup	HIV-1 Tat interactive protein 2, 30kDa															
NM_001270428	LMO1	chr11	8202426	8246758	-	Onc	LIM domain only 1 (rhombotin 1)															
NM_130385	MRVI1	chr11	10551213	10672111	-	Tum Sup	murine retrovirus integration site 1 homolog															
NM_139157	ST5	chr11	8671474	8789458	-	Tum Sup	suppression of tumorigenicity 5															
NM_006292	TSG101	chr11	18458433	18505079	-	Tum Sup	tumor susceptibility gene 101															
NM_001142316	LMO2	chr11	33836698	33847947	-	Onc	LIM domain only 2 (rhombotin-like 1)															
NM_000378	WT1	chr11	32365897	32413657	-	Tum Sup	Wilms tumor 1															
NM_207122	EXT2	chr11	44073674	44223556	+	Tum Sup	exostoses (multiple) 2															
NM_001206615	EHF	chr11	34599163	34641410	+	Tum Sup	ets homologous factor															
NM_001198810	SLC43A1	chr11	57008579	57038934	-	Other	solute carrier family 43, member 1															
NM_001024844	CD82	chr11	44543716	44597891	+	Tum Sup	CD82 antigen															
NM_003120	SPI1	chr11	47332984	47356703	-	Onc	spleen focus forming virus (SFFV) proviral integration oncogene spi1															
NM_053056	CCND1	chr11	69165053	69178423	+	Onc	cyclin D1															
NM_005851	CDK2AP2	chr11	67030543	67032678	-	Tum Sup	CDK2-associated protein 2															
NM_000738	CHRM1	chr11	62432726	62445588	-	Other	cholinergic receptor, muscarinic 1															
NM_005231	CTTN	chr11	69922259	69960338	+	Onc	cortactin															
NM_005247	FGF3	chr11	69333916	69343129	-	Onc	fibroblast growth factor 3 (murine mammary tumor virus integration site (v-int-2) oncogene homolog)															
NM_005438	FOSL1	chr11	65416267	65424573	-	Onc	FOS-like antigen 1															
NM_130800	MEN1	chr11	64327561	64334611	-	Tum Sup	multiple endocrine neoplasia I															
NM_006185	NUMA1	chr11	71391558	71469221	-	Other	nuclear mitotic apparatus protein 1															
NM_000932	PLCB3	chr11	63775570	63793500	+	Tum Sup	phospholipase C, beta 3 (phosphatidylinositol-specific)															

Table S1

tumor associated genes								HIV			MLV			MMTV			MMTV(SIN)			MMTV(SIN)arrest		
RefSeq	gene symbol	Chrom	txStart	txEnd	stra	Category	Gene name	position	integrant	strand	position	integrant	stran	positiopn	integrant	stran	position	integrant	stran	position	integrant	stran
NM_206873	PPP1CA	chr11	66922227	66925952	-	Tum Sup	protein phosphatase 1, catalytic subunit, alpha isoform															
NM_003860	BANF1	chr11	65526125	65528193	+	Onc	barrier to autointegration factor 1															
NM_212469	CHKA	chr11	67576901	67645434	-	Onc	choline kinase alpha (Previous name: choline kinase)															
NM_003824	FADD	chr11	69726916	69731156	+	Other	Fas (TNFRSF6)-associated via death domain															
NM_002007	FGF4	chr11	69296977	69299352	-	Onc	fibroblast growth factor 4 (heparin secretory transforming protein 1, Kaposi sarcoma oncogene)															
NM_153451	ORAOV1	chr11	69189512	69199346	-	Onc	oral cancer overexpressed 1															
NM_001256802	RPS3	chr11	74788182	74810993	+	Other	ribosomal protein S3															
NM_014786	ARHGEF17	chr11	72697310	72758073	+	Tum Sup	Rho guanine nucleotide exchange factor (GEF) 17				72723878	AY516567	+									
NM_000051	ATM	chr11	107598768	107745036	+	--	ataxia telangiectasia mutated (Previous names: ataxia telangiectasia mutated (includes complementati															
NM_003478	CUL5	chr11	107384617	107483698	+	Tum Sup	cullin 5															
NM_014622	LOH11CR2A	chr11	123491321	123522828	+	Tum Sup	loss of heterozygosity, 11, chromosomal region 2, gene A															
NM_005933	MLL	chr11	117812414	117902749	+	Onc	myeloid/lymphoid or mixed-lineage leukemia (trithorax homolog, Drosophila)															
NM_004585	RARRES3	chr11	63060848	63070506	+	Tum Sup	retinoic acid receptor responder (tazarotene induced) 3															
NM_016952	CDON	chr11	125331922	125438397	-	Onc	Cdon homolog (mouse)															
NM_014333	IGSF4	chr11	114549555	114880451	-	Tum Sup	immunoglobulin superfamily, member 4															
NM_181700	PPP2R1B	chr11	111102841	111142379	-	Tum Sup	protein phosphatase 2 (formerly 2A), regulatory subunit A (PR 65), beta isoform															
NM_005188	CBL	chr11	118582195	118684069	+	Onc	Cas-Br-M (murine) ecotropic retroviral transforming sequence	118605969	BH609666	+												

Table S1

tumor associated genes								HIV			MLV			MMTV			MMTV(SIN)			MMTV(SIN)arrest		
RefSeq	gene symbol	Chrom	txStart	txEnd	stra	Category	Gene name	position	integrant	strand	position	integrant	stran	positiopn	integrant	stran	position	integrant	stran	position	integrant	stran
NM_005188	CBL	chr11	118582195	118684069	+	Onc	Cas-Br-M (murine) ecotropic retroviral transforming sequence	118606108	CL800684	+												
NM_004397	DDX6	chr11	118123682	118167182	-	Other	DEAD (Asp-Glu-Ala-Asp) box polypeptide 6															
NM_001143820	ETS1	chr11	127833865	127962663	-	& Onc & Tum Sup	v-ets erythroblastosis virus E26 oncogene homolog 1 (avian)															
NR_045205	CHEK1	chr11	125001333	125032252	+	Tum Sup	CHK1 checkpoint homolog (S. pombe)															
NM_001167681	FLI1	chr11	128069020	128188372	+	Onc	Friend leukemia virus integration 1															
NM_001127582	ING4	chr12	6629964	6642569	-	Tum Sup	inhibitor of growth family, member 4															
NM_001190839	MGP	chr12	14925381	14930120	-	Other	matrix Gla protein															
NM_004064	CDKN1B	chr12	12761568	12766572	+	Tum Sup	cyclin-dependent kinase inhibitor 1B (p27, Kip1)															
NM_003979	GPRC5A	chr12	12935222	12957867	+	Tum Sup	G protein-coupled receptor, family C, group 5, member A (Previous name: retinoic acid induced 3)															
NM_001759	CCND2	chr12	4253162	4284783	+	Onc	cyclin D2															
NM_001987	ETV6	chr12	11694054	11939592	+	Tum Sup	ets variant gene 6 (TEL oncogene)															
NM_020996	FGF6	chr12	4413568	4425041	-	Onc	fibroblast growth factor 6															
NM_001423	EMP1	chr12	13240868	13260975	+	Other	epithelial membrane protein 1															
NM_001164747	RASSF8	chr12	26017954	26117074	+	Tum Sup	Ras association (RalGDS/AF-6) domain family (N-terminal) member 8 (Previous name: chromosome 12 open															
NM_198965	PTHLH	chr12	28002283	28016183	-	Other	parathyroid hormone-like hormone															
NM_033360	KRAS	chr12	25249446	25295121	-	Onc	v-Ki-ras2 Kirsten rat sarcoma viral oncogene homolog															
NM_005086	SSPN	chr12	26239772	26278975	+	Other	sarcospan (Kras oncogene-associated gene)															
NM_001164595	PDZRN4	chr12	39868516	40254659	+	--	PDZ domain containing ring finger 4 PDZRN4: Approved													40005465	133_82_1	+
NM_014470	RND1	chr12	47537182	47545920	-	Onc	Rho family GTPase 1															

Table S1

tumor associated genes								HIV			MLV			MMTV			MMTV(SIN)			MMTV(SIN)arrest		
RefSeq	gene symbol	Chrom	txStart	txEnd	stra	Category	Gene name	position	integrant	strand	position	integrant	stran	positiopn	integrant	stran	position	integrant	stran	position	integrant	stran
NM_052827	CDK2	chr12	54646822	54652835	+	Other	cyclin-dependent kinase 2															
NM_001982	ERBB3	chr12	54760075	54783558	+	Onc	v-erb-b2 erythroblastic leukemia viral oncogene homolog 3 (avian)															
NM_033045	KRT84	chr12	51057862	51065684	-	--	keratin 84 (Previous names: keratin, hair, basic, 4) KRT84: Approved															
NM_005430	WNT1	chr12	47658502	47662663	+	Onc	wingless-type MMTV integration site family, member 1															
NM_003394	WNT10B	chr12	47645389	47651908	-	Other	wingless-type MMTV integration site family, member 10B															
NM_005730	CTDSP2	chr12	56499976	56527014	-	Other	CTD (carboxy-terminal domain, RNA polymerase II, polypeptide A) small phosphatase 2															
NM_006530	YEATS4	chr12	68039798	68070843	+	Other	YEATS domain containing 4															
NM_001195057	DDIT3	chr12	56196637	56200567	-	Onc	DNA-damage-inducible transcript 3															
NM_012404	ANP32D	chr12	47152714	47153110	+	Other	acidic (leucine-rich) nuclear phosphoprotein 32 family, member D															
NM_000424	KRT5	chr12	51194625	51200510	-	--	keratin 5 (Previous names: epidermolysis bullosa simplex 2 Dowling-Meara/Kobner/Weber-Cockayne types															
NM_001167609	GLI1	chr12	56140200	56152314	+	Onc	glioma-associated oncogene homolog 1 (zinc finger protein)															
NM_004503	HOXC6	chr12	52708460	52710874	+	Other	homeobox C6															
NM_000075	CDK4	chr12	56427776	56432497	-	Onc	cyclin-dependent kinase 4															
NM_002392	MDM2	chr12	67488237	67525587	+	Onc	Mdm2, transformed 3T3 cell double minute 2, p53 binding protein (mouse)													67511427	1312_139_	+
NM_003483	HMGA2	chr12	64504506	64646338	+	Onc	high mobility group AT-hook 2 (Previous name: high-mobility group (nonhistone chromosomal) protein i															
NM_002583	PAWR	chr12	78509875	78608921	-	Tum Sup	PRKC, apoptosis, WT1, regulator	78510912	CL529499	+												

Table S1

tumor associated genes								HIV			MLV			MMTV			MMTV(SIN)			MMTV(SIN)arrest		
RefSeq	gene symbol	Chrom	txStart	txEnd	stra	Category	Gene name	position	integrant	strand	position	integrant	stran	positiopn	integrant	stran	position	integrant	stran	position	integrant	stran
NM_002583	PAWR	chr12	78509875	78608921	-	Tum Sup	PRKC, apoptosis, WT1, regulator	78519186	CL800293	+												
NM_173353	TPH2	chr12	70618892	70712488	+	--	tryptophan hydroxylase 2 TPH2: Approved															
NM_004316	ASCL1	chr12	101875581	101878424	+	Other	achaete-scute complex-like 1 (Drosophila)															
NM_001160	APAF1	chr12	97563208	97653342	+	Tum Sup	apoptotic peptidase activating factor															
NM_012406	PRDM4	chr12	106650772	106679044	-	Tum Sup	PR domain containing 4															
NM_080601	PTPN11	chr12	111340918	111409110	+	Onc	protein tyrosine phosphatase, non-receptor type 11 (Previous name: Noonan syndrome 1)															
NM_001024808	BCL7A	chr12	120944243	120984333	+	Other	B-cell CLL/lymphoma 7A															
NM_001270434	CDK2AP1	chr12	122311469	122318754	-	Tum Sup	CDK2-associated protein 1															
NM_023012	RSRC2	chr12	121555142	121577513	-	Tum Sup	arginine/serine-rich coiled-coil 2															
NM_016529	ATP8A2	chr13	24844208	25493420	+	Tum Sup	ATPase, aminophospholipid transporter-like, Class I, type 8A, member 2										25219892	604_114	+	24893374	631_113_2	+
NM_015032	APRIN	chr13	32058592	32250157		Tum Sup	androgen-induced proliferation inhibitor															
NM_000059	BRCA2	chr13	31787616	31871809	+	Tum Sup	breast cancer 2, early onset															
NM_001265	CDX2	chr13	27434204	27441505	-	Other	caudal type homeobox transcription factor 2															
NM_004128	GTF2F2	chr13	44592630	44756239	+	--	general transcription factor IIF, polypeptide 2, 30kDa (Previous names: general transcription factor															
NM_001007278	RFP2	chr13	49469144	49490604	+	Tum Sup	ret finger protein 2															
NM_002015	FOXO1A	chr13	40027801	40138734	-	Tum Sup	forkhead box O1A (rhabdomyosarcoma)															
NM_014059	C13ORF15	chr13	40929542	40943013	+	& Other & Tum Sup	chromosome 13 open reading frame 15															
NM_138450	ARL11	chr13	49100435	49106009	+	Tum Sup	ADP-ribosylation factor-like 11															
NM_000321	RB1	chr13	47775883	47954027	+	Tum Sup	retinoblastoma 1 (including osteosarcoma)															

Table S1

tumor associated genes								HIV			MLV			MMTV			MMTV(SIN)			MMTV(SIN)arrest		
RefSeq	gene symbol	Chrom	txStart	txEnd	stra	Category	Gene name	position	integrant	strand	position	integrant	stran	positiopn	integrant	stran	position	integrant	stran	position	integrant	stran
NR_002605	DLEU1	chr13	49554414	49577434	+	Tum Sup	deleted in lymphocytic leukemia, 1															
NR_002612	DLEU2	chr13	49454688	49597678	-	Tum Sup	deleted in lymphocytic leukemia, 2															
NM_001008895	CUL4A	chr13	112911931	112967393	+	Tum Sup	cullin 4A															
NM_198219	ING1	chr13	110163610	110171422	+	Tum Sup	inhibitor of growth family, member 1															
NM_024979	MCF2L	chr13	112704028	112802054	+	Onc	MCF.2 cell line derived transforming sequence-like										112731957	s1004_163	+			
NM_004050	BCL2L2	chr14	22845810	22850808	+	Onc	BCL2-like 2				59056337	AY516170	-									
NM_001079668	NKX2-1	chr14	36055352	36059181	-	Onc	NK2 homeobox 1 (Previous names: benign chorea, thyroid transcription factor 1)															
NM_014360	NKX2-8	chr14	36118966	36121537	-	Other	NK2 transcription factor related, locus 8 (Drosophila)															
NM_145113	MAX	chr14	64611597	64638980	-	Other	MYC associated factor X															
NM_002083	GPX2	chr14	64475622	64479376	-	Onc	glutathione peroxidase 2 (gastrointestinal)															
NM_005252	FOS	chr14	74815233	74818690	+	Onc	v-fos FBJ murine osteosarcoma viral oncogene homolog															
NM_012245	SKIIP	chr14	77253697	77297250	-	Other	SNW domain containing 1															
NM_021966	TCL1A	chr14	95246056	95250286	-	Onc	T-cell leukemia/lymphoma 1A															
NM_004918	TCL1B	chr14	95222506	95228733	+	Onc	T-cell leukemia/lymphoma 1B															
NR_028288	TCL6	chr14	95187267	95209542	+	Onc	T-cell leukemia/lymphoma 6															
NM_005113	GOLGA5	chr14	92330402	92376057	+	Onc	golgi autoantigen, golgin subfamily a, 5															
NM_005163	AKT1	chr14	104306731	104330983	-	Onc	v-akt murine thymoma viral oncogene homolog 1															
NR_027514	CCNDBP1	chr15	41264757	41276667	+	Tum Sup	cyclin D-type binding-protein 1															
NM_170697	ALDH1A2	chr15	56032913	56093587	-	Tum Sup	aldehyde dehydrogenase 1 family, member A2															
NM_033240	PML	chr15	72074066	72115788	+	Tum Sup	promyelocytic leukemia	72079379	AY517169.1	-												
NM_033240	PML	chr15	72074066	72115788	+	Tum Sup	promyelocytic leukemia	72083622	CL529666	+												
NM_000499	CYP1A1	chr15	72798935	72804930	-	Other	cytochrome P450, family 1, subfamily A, polypeptide 1															

Table S1

tumor associated genes								HIV			MLV			MMTV			MMTV(SIN)			MMTV(SIN)arrest		
RefSeq	gene symbol	Chrom	txStart	txEnd	stra	Category	Gene name	position	integrant	strand	position	integrant	stran	positiopn	integrant	stran	position	integrant	stran	position	integrant	stran
NM_001018004	TPM1	chr15	61121890	61151166	+	Tum Sup	tropomyosin 1 (alpha) (Previous name: chromosome 15 open reading frame 13)															
NM_006305	ANP32A	chr15	66857928	66900315	-	Tum Sup	acidic (leucine-rich) nuclear phosphoprotein 32 family, member A															
NM_002499	NEO1	chr15	71131877	71384600	+	Tum Sup	neogenin homolog 1 (chicken)															
NM_001145102	SMAD3	chr15	65205107	65274587	+	Tum Sup	SMAD, mothers against DPP homolog 3 (Drosophila)															
NM_001897	CSPG4	chr15	73753717	73792244	-	Other	chondroitin sulfate proteoglycan 4 (melanoma-associated)															
NM_002902	RCN2	chr15	75011016	75029656	+	Tum Sup	reticulocalbin 2, EF-hand calcium binding domain															
NM_145204	SENP8	chr15	70197684	70220365	+	--	SUMO/sentrin specific peptidase family member 8 (Previous names: protease, cysteine, 2 (NEDD8 specif															
NM_004378	CRABP1	chr15	76419720	76427627	+	Tum Sup	cellular retinoic acid binding protein 1 (Previous name: cellular retinoic acid-binding protein 1)															
NM_006738	AKAP13	chr15	83724850	84093593	+	Onc	A kinase (PRKA) anchor protein 13	83760953	BH609727	+												
NM_006738	AKAP13	chr15	83724850	84093593	+	Onc	A kinase (PRKA) anchor protein 13	84066374	BH609728	-												
NM_006738	AKAP13	chr15	83724850	84093593	+	Onc	A kinase (PRKA) anchor protein 13	84045966	CL529607	-												
NM_001127190	CSK	chr15	72861477	72882592	+	Onc	c-src tyrosine kinase															
NM_001145358	SIN3A	chr15	73448772	73531140	-	Tum Sup	SIN3 homolog A, transcription regulator (yeast) (Previous names: SIN3 homolog A, transcriptional reg															
NM_005928	MFGE8	chr15	87242917	87257667	-	Other	milk fat globule-EGF factor 8 protein															
NM_012125	CHRM5	chr15	32048380	32144579	+	Other	cholinergic receptor, muscarinic 5															
NM_000057	BLM	chr15	89061582	89159690	+	Tum Sup	Bloom syndrome															
NM_001143785	FES	chr15	89228668	89240010	+	Onc	feline sarcoma oncogene															
NM_000296	PKD1	chr16	2078711	2125900	-	Tum Sup	polycystic kidney disease 1 (autosomal dominant)															

Table S1

tumor associated genes								HIV			MLV			MMTV			MMTV(SIN)			MMTV(SIN)arrest		
RefSeq	gene symbol	Chrom	txStart	txEnd	stra	Category	Gene name	position	integrant	strand	position	integrant	stran	positiopn	integrant	stran	position	integrant	stran	position	integrant	stran
NM_001114382	TSC2	chr16	2037990	2078714	+	Tum Sup	tuberous sclerosis 2															
NM_005030	PLK1	chr16	23597701	23609189	+	Other	polo-like kinase 1 (Drosophila)															
NM_001170634	FUS	chr16	31098931	31113693	+	Onc	fusion (involved in t(12;16) in malignant liposarcoma)															
NM_001042412	CYLD	chr16	49333529	49393347	+	Tum Sup	cylindromatosis (turban tumor syndrome)															
NM_005611	RBL2	chr16	52025851	52083061	+	Tum Sup	retinoblastoma-like 2 (p130)															
NM_001031804	MAF	chr16	78185245	78192123	-	Onc	v-maf musculoaponeurotic fibrosarcoma oncogene homolog (avian)															
NM_022845	CBFB	chr16	65620550	65692459	+	Other	core-binding factor, beta subunit	65680238	CL799604	-												
NM_022845	CBFB	chr16	65620550	65692459	+	Other	core-binding factor, beta subunit	65622828	BH609751	+												
NM_022845	CBFB	chr16	65620550	65692459	+	Other	core-binding factor, beta subunit	65630439	CL529504	-												
NM_022845	CBFB	chr16	65620550	65692459	+	Other	core-binding factor, beta subunit	65658377	AY517062.1	-												
NM_004360	CDH1	chr16	67328695	67426945	+	Tum Sup	cadherin 1, type 1, E-cadherin (epithelial)															
NM_016948	PARD6A	chr16	66252351	66254182	+	Other	par-6 partitioning defective 6 homolog alpha (C.elegans)															
NM_130791	WWOX	chr16	76690827	76870094	+	Tum Sup	WW domain containing oxidoreductase (Previous name: WW domain-containing oxidoreductase)	76721205	CL529169	+							76760157	7_1_2189_	+			
NM_001098533	CDK10	chr16	88280576	88290273	+	Other	cyclin-dependent kinase (CDC2-like) 10															
NM_002163	IRF8	chr16	84490274	84513712	+	Tum Sup	interferon regulatory factor 8 (Previous name: interferon consensus sequence binding protein 1)															
NM_001220488	CDH13	chr16	81217899	82387716	+	Tum Sup	cadherin 13, H-cadherin (heart)															
NM_001083314	CHMP1A	chr16	88238339	88251630	-	Tum Sup	chromatin modifying protein 1A (Previous name: procollagen (type III) N-endopeptidase)															
NM_014427	CPNE7	chr16	88169676	88191155	+	Tum Sup	copine VII															
NM_001140	ALOX15	chr17	4480962	4491709	-	Other	arachidonate 15-lipoxygenase															

Table S1

tumor associated genes								HIV			MLV			MMTV			MMTV(SIN)			MMTV(SIN)arrest		
RefSeq	gene symbol	Chrom	txStart	txEnd	stra	Category	Gene name	position	integrant	strand	position	integrant	stran	positiopn	integrant	stran	position	integrant	stran	position	integrant	stran
NM_005206	CRK	chr17	1271396	1306311	-	Onc	v-crk sarcoma virus CT10 oncogene homolog (avian)															
NM_006497	HIC1	chr17	1905142	1909731	+	Tum Sup	hypermethylated in cancer 1															
NM_001105538	MYBBP1A	chr17	4388939	4405430	-	Tum Sup	MYB binding protein (P160) 1a															
NM_006224	PITPNA	chr17	1368032	1412860	-	--	phosphatidylinositol transfer protein, alpha (Previous name: phosphotidylinositol transfer protein)															
NM_018289	VPS53	chr17	382282	564846	-	Tum Sup	vacuolar protein sorting 53 homolog (S. cerevisiae) (Previous name: vacuolar protein sorting 53 (yea	525285	CL799785	-	399912	AY515951	-									
NM_018289	VPS53	chr17	382282	564846	-	Tum Sup	vacuolar protein sorting 53 homolog (S. cerevisiae) (Previous name: vacuolar protein sorting 53 (yea	536999	CL800770	-	446741	AY516057	-									
NM_018289	VPS53	chr17	382282	564846	-	Tum Sup	vacuolar protein sorting 53 homolog (S. cerevisiae) (Previous name: vacuolar protein sorting 53 (yea	461907	CL799563	-												
NM_002616	PER1	chr17	7984512	7996478	-	Tum Sup	period homolog 1 (Drosophila) (Previous name: period (Drosophila) homolog 1)															
NM_001126116	TP53	chr17	7512444	7519536	-	& -- & Tum Sup	tumor protein p53 (Li-Fraumeni syndrome)															
NM_017523	XAF1	chr17	6599879	6619688	+	& Onc & Tum Sup	XIAP associated factor 1															
NM_004822	NTN1	chr17	8865583	9088042	+	Other	netrin 1															
NM_004505	USP6	chr17	4972410	5019048	+	Onc	ubiquitin specific peptidase 6 (Tre-2 oncogene)															
NM_139215	TAF15	chr17	31160571	31198359	+	Onc	TAF15 RNA polymerase II, TATA box binding protein (TBP)-associated factor, 68kDa (Previous names: TA															

Table S1

tumor associated genes								HIV			MLV			MMTV			MMTV(SIN)			MMTV(SIN)arrest		
RefSeq	gene symbol	Chrom	txStart	txEnd	stra	Category	Gene name	position	integrant	strand	position	integrant	stran	positiopn	integrant	stran	position	integrant	stran	position	integrant	stran
NM_006495	EVI2B	chr17	26654913	26665256	-	Other	ecotropic viral integration site 2B															
NM_001042492	NF1	chr17	26446070	26728821	+	Tum Sup	neurofibromin 1 (neurofibromatosis, von Recklinghausen disease, Watson disease)															
NM_001190919	THRA	chr17	35471971	35503646	+	Onc	thyroid hormone receptor, alpha (erythroblastic leukemia viral (v-erb-a) oncogene homolog, avian)															
NM_002982	CCL2	chr17	29606408	29608333	+	Other	chemokine (C-C motif) ligand 2															
NM_001005862	ERBB2	chr17	35097918	35138441	+	Onc	v-erb-b2 erythroblastic leukemia viral oncogene homolog 2, neuro/glioblastoma derived oncogene homol															
NM_001094	ACCN1	chr17	28364219	29507938	-	Tum Sup	amiloride-sensitive cation channel 1, neuronal															
	C17ORF37	chr17	35138935	35140314	-	Onc	chromosome 17 open reading frame 37															
NM_001030002	GRB7	chr17	35148101	35157064	+	Other	growth factor receptor-bound protein 7															
NM_005568	LHX1	chr17	32368884	32376028	+	Other	LIM homeobox 1															
NR_003562	MPP3	chr17	39233692	39266073	-	Tum Sup	membrane protein, palmitoylated 3 (MAGUK p55 subfamily member 3)															
NM_007299	BRCA1	chr17	38449837	38530994	-	Tum Sup	breast cancer 1, early onset															
NM_005937	MLLT6	chr17	34115398	34139582	+	Other	myeloid/lymphoid or mixed-lineage leukemia (trithorax homolog, Drosophila); translocated to, 6															
NM_002634	PHB	chr17	44836418	44847241	-	Tum Sup	prohibitin															
NM_001145302	RARA	chr17	35727998	35767421	+	Onc	retinoic acid receptor, alpha															
NM_005801	EIF1	chr17	37098652	37101424	+	Tum Sup	eukaryotic translation initiation factor 1															
NM_003150	STAT3	chr17	37718868	37794039	-	& -- & Onc	signal transducer and activator of transcription 3 (acute-phase response factor)	37761451	CL529622	-												

Table S1

tumor associated genes								HIV			MLV			MMTV			MMTV(SIN)			MMTV(SIN)arrest		
RefSeq	gene symbol	Chrom	txStart	txEnd	stra	Category	Gene name	position	integrant	strand	position	integrant	stran	positiopn	integrant	stran	position	integrant	stran	position	integrant	stran
NM_003150	STAT3	chr17	37718868	37794039	-	& -- & Onc	signal transducer and activator of transcription 3 (acute-phase response factor)	37761181	CL529621	+												
NM_001258	CDK3	chr17	71508581	71513675	+	Other	cyclin-dependent kinase 3															
NM_004655	AXIN2	chr17	60955144	60988202	-	Tum Sup	axin 2 (conductin, axil)															
NM_006572	GNA13	chr17	60435868	60483382	-	Onc	guanine nucleotide binding protein (G protein), alpha 13															
NM_001168	BIRC5	chr17	73721871	73733311	+	Other	baculoviral IAP repeat-containing 5 (survivin)															
NM_001005619	ITGB4	chr17	71232370	71265494	+	Other	integrin, beta 4															
NM_003004	SECTM1	chr17	77872188	77885210	-	Other	secreted and transmembrane 1															
NM_001142601	SPHK1	chr17	71892284	71895536	+	Onc	sphingosine kinase 1															
NM_032711	MAFG	chr17	77469436	77474737	-	Onc	v-maf musculoaponeurotic fibrosarcoma oncogene homolog G (avian)															
NM_001071	TYMS	chr18	647603	663499	+	--	thymidylate synthetase TYMS: Approved															
NM_005433	YES1	chr18	711591	802327	-	Onc	v-yes-1 Yamaguchi sarcoma viral oncogene homolog 1															
NM_005257	GATA6	chr18	18003401	18036489	+	& Onc & Tum Sup	GATA binding protein 6 (Previous name: GATA-binding protein 6)															
NM_001135937	SMAD2	chr18	43613463	43711515	-	Tum Sup	SMAD, mothers against DPP homolog 2 (Drosophila)	43678923	CL529185	-												
NM_001135937	SMAD2	chr18	43613463	43711515	-	Tum Sup	SMAD, mothers against DPP homolog 2 (Drosophila)	43677799	CL799875	+												
NM_001135937	SMAD2	chr18	43613463	43711515	-	Tum Sup	SMAD, mothers against DPP homolog 2 (Drosophila)	43662302	CL529663	+												
NM_001135937	SMAD2	chr18	43613463	43711515	-	Tum Sup	SMAD, mothers against DPP homolog 2 (Drosophila)	43698413	CL529741	-												
NM_005359	SMAD4	chr18	46810580	46865409	+	Tum Sup	SMAD, mothers against DPP homolog 4 (Drosophila)															
NM_005215	DCC	chr18	48120539	49316271	+	Tum Sup	deleted in colorectal carcinoma															

Table S1

tumor associated genes								HIV			MLV			MMTV			MMTV(SIN)			MMTV(SIN)arrest		
RefSeq	gene symbol	Chrom	txStart	txEnd	stra	Category	Gene name	position	integrant	strand	position	integrant	stran	positiopn	integrant	stran	position	integrant	stran	position	integrant	stran
NM_002035	FVT1	chr18	59145951	59185486	-	Other	follicular lymphoma variant translocation 1															
NM_002639	SERPINB5	chr18	59295123	59323298	+	Tum Sup	serpin peptidase inhibitor, clade B (ovalbumin), member 5															
NM_021127	PMAIP1	chr18	55718171	55722518	+	Other	phorbol-12-myristate-13-acetate-induced protein 1															
NM_000633	BCL2	chr18	58941558	59137593	-	Onc	B-cell CLL/lymphoma 2															
NM_005883	APC2	chr19	1401147	1424243	+	Tum Sup	adenomatosis polyposis coli 2															
NM_005224	ARID3A	chr19	877036	923803	+	Onc	AT rich interactive domain 3A (BRIGHT- like)	911064	CL799972	+												
NM_005224	ARID3A	chr19	877036	923803	+	Onc	AT rich interactive domain 3A (BRIGHT- like)	914555	CL800020	-												
NM_005755	EBI3	chr19	4180539	4188524	+	--	Epstein-Barr virus induced 3 EBI3: Approved															
NM_005934	MLLT1	chr19	6161391	6230959	-	Other	myeloid/lymphoid or mixed-lineage leukemia (trithorax homolog, Drosophila); translocated to, 1				6225234	AY516580	-									
NM_002229	JUNB	chr19	12763309	12765125	+	Onc	jun B proto-oncogene															
NM_005354	JUND	chr19	18251562	18253432	-	Onc	jun D proto-oncogene															
NM_005583	LYL1	chr19	13070841	13074974	-	Onc	lymphoblastic leukemia derived sequence 1															
NM_015965	NDUFA13	chr19	19488018	19500013	+	Tum Sup	NADH dehydrogenase (ubiquinone) 1 alpha subcomplex, 13															
NM_032152	PRAM1	chr19	8460939	8473538	-	Onc	PML-RARA regulated adaptor molecule 1															
NM_005428	VAV1	chr19	6723678	6808377	+	Onc	vav 1 oncogene				6782061	AY516518	+									
NM_006532	ELL	chr19	18414472	18493937	-	Onc	elongation factor RNA polymerase II															
NM_005370	RAB8A	chr19	16083489	16105445	+	Onc	RAB8A, member RAS oncogene family															
NM_006221	PIN1	chr19	9806882	9821365	+	Onc	peptidylprolyl cis/trans isomerase, NIMA-interacting 1 (Previous name: protein (peptidyl-prolyl cis/															
NM_001238	CCNE1	chr19	34994740	35007055	+	Onc	cyclin E1															
NM_006494	ERF	chr19	47443556	47451149	-	Tum Sup	Ets2 repressor factor				47449000	AY516670	-									
NM_006494	ERF	chr19	47443556	47451149	-	Tum Sup	Ets2 repressor factor				47448798	AY516219	-									

Table S1

tumor associated genes								HIV			MLV			MMTV			MMTV(SIN)			MMTV(SIN)arrest		
RefSeq	gene symbol	Chrom	txStart	txEnd	stra	Category	Gene name	position	integrant	strand	position	integrant	stran	positiopn	integrant	stran	position	integrant	stran	position	integrant	stran
NM_001077500	KLK10	chr19	56207811	56215243	-	Tum Sup	kallikrein-related peptidase 10 (Previous name: kallikrein 10)															
NM_001699	AXL	chr19	46416947	46459511	+	Onc	AXL receptor tyrosine kinase															
NM_021102	SPINT2	chr19	43446937	43475094	+	Tum Sup	serine peptidase inhibitor, Kunitz type, 2 (Previous names: serine protease inhibitor, Kunitz type,															
NM_007145	ZNF146	chr19	41397343	41421515	+	Onc	zinc finger protein 146															
NM_001243028	AKT2	chr19	45428063	45483142	-	Onc	v-akt murine thymoma viral oncogene homolog 2															
NM_005178	BCL3	chr19	49943817	49955141	+	Onc	B-cell CLL/lymphoma 3															
NM_013376	SERTAD1	chr19	45620248	45623772	-	Onc	SERTA domain containing 1															
NM_006890	CEACAM7	chr19	46869074	46883936	-	Tum Sup	carcinoembryonic antigen-related cell adhesion molecule 7															
NM_001022	RPS19	chr19	47055827	47067324	+	Other	ribosomal protein S19															
NM_000660	TGFB1	chr19	46528651	46551671	-	& -- & Onc	transforming growth factor, beta 1 (Camurati-Engelmann disease)															
NM_014475	DHDH	chr19	54128750	54140038	+	--	dihydrodiol dehydrogenase (dimeric) DHDH: Approved															
NM_015711	GLTSCR1	chr19	52803264	52898346	+	Tum Sup	glioma tumor suppressor candidate region gene 1															
NM_015710	GLTSCR2	chr19	52940604	52952135	+	Tum Sup	glioma tumor suppressor candidate region gene 2															
NM_138761	BAX	chr19	54149928	54156867	+	Other	BCL2-associated X protein															
NM_006270	RRAS	chr19	54830363	54835212	-	Onc	related RAS viral (r-ras) oncogene homolog															
NM_021872	CDC25B	chr20	3724400	3734761	+	Onc	cell division cycle 25B															
NM_003245	TGM3	chr20	2224612	2269725	+	Onc	transglutaminase 3 (E polypeptide, protein-glutamine-gamma-glutamyltransferase)															
NM_006606	RBBP9	chr20	18415187	18425887	-	Other	retinoblastoma binding protein 9															
NM_001172132	HCK	chr20	30103651	30153318	+	Onc	hemopoietic cell kinase															
NM_012325	MAPRE1	chr20	30871359	30901872	+	Other	microtubule-associated protein, RP/EB family, member 1															

Table S1

tumor associated genes								HIV			MLV			MMTV			MMTV(SIN)			MMTV(SIN)arrest		
RefSeq	gene symbol	Chr om	txStart	txEnd	s tr a	Categ ory	Gene name	position	integrant	str an d	position	integrant	st ra n	positiopn	integrant	st ra n	position	integrant	st ra n	position	integrant	st ra n
NM_006892	DNMT3B	chr20	30813851	30860823	+	Other	DNA (cytosine-5-)-methyltransferase 3 beta															
NM_005225	E2F1	chr20	31726952	31737871	-	Tum Sup	E2F transcription factor 1															
NM_005461	MAFB	chr20	38747930	38751290	-	Onc	v-maf musculoaponeurotic fibrosarcoma oncogene homolog B (avian)															
NM_001174088	NCOA3	chr20	45564007	45719028	+	Onc	nuclear receptor coactivator 3	45664614	CL529540	+												
NM_001174088	NCOA3	chr20	45564007	45719028	+	Onc	nuclear receptor coactivator 3	45639475	CL800590	+												
NM_005417	SRC	chr20	35406501	35467235	+	Onc	v-src sarcoma (Schmidt-Ruppin A-2) viral oncogene homolog (avian)															
NM_003881	WISP2	chr20	42777298	42789866	+	Onc	WNT1 inducible signaling pathway protein 2															
NM_001256135	CSE1L	chr20	47096189	47146904	+	Onc	CSE1 chromosome segregation 1-like (yeast)	47137911	CL800151	-												
NM_001256135	CSE1L	chr20	47096189	47146904	+	Onc	CSE1 chromosome segregation 1-like (yeast)	47125353	CL529206	-												
NM_001336	CTSZ	chr20	57003636	57015704	-	Other	cathepsin Z															
NM_002466	MYBL2	chr20	41729122	41778536	+	Onc	v-myb myeloblastosis viral oncogene homolog (avian)-like 2															
NM_020436	SALL4	chr20	49833989	49852455	-	Onc	sal-like 4 (Drosophila) (Previous name: sal (Drosophila)-like 4)															
NM_003222	TFAP2C	chr20	54637764	54647745	+	Other	transcription factor AP-2 gamma (activating enhancer binding protein 2 gamma)															
NM_006526	ZNF217	chr20	51617016	51633043	-	Onc	zinc finger protein 217															
NM_198436	AURKA	chr20	54377851	54400758	-	Onc	aurora kinase A															
NM_003657	BCAS1	chr20	51993485	52120711	-	Other	breast carcinoma amplified sequence 1															
NM_016592	GNAS	chr20	56848189	56919645	+	Onc	GNAS complex locus (Previous names: guanine nucleotide binding protein (G protein), alpha stimulatin															
NM_001256358	PTK6	chr20	61630219	61639167	-	& -- & Other	PTK6 protein tyrosine kinase 6															
NM_003823	TNFRSF6B	chr20	61798447	61800495	+	Onc	tumor necrosis factor receptor superfamily, member 6b, decoy															

Table S1

tumor associated genes								HIV			MLV			MMTV			MMTV(SIN)			MMTV(SIN)arrest		
RefSeq	gene symbol	Chrom	txStart	txEnd	stra	Category	Gene name	position	integrant	strand	position	integrant	stran	positiopn	integrant	stran	position	integrant	stran	position	integrant	stran
NM_031215	CABLES2	chr20	60397080	60415734	-	Tum Sup	Cdk5 and Abl enzyme substrate 2															
NM_003253	TIAM1	chr21	31412606	31853161	-	Other	T-cell lymphoma invasion and metastasis 1															
NM_130445	COL18A1	chr21	45649524	45758062	+	Tum Sup	collagen, type XVIII, alpha 1															
NM_182918	ERG	chr21	38673819	38792298	-	Onc	v-ets erythroblastosis virus E26 oncogene like (avian)															
NM_001001890	RUNX1	chr21	35081967	35182857	-	Other	runt-related transcription factor 1 (acute myeloid leukemia 1; aml1 oncogene)															
NM_001256295	ETS2	chr21	39099100	39118748	+	Onc	v-ets erythroblastosis virus E26 oncogene homolog 2 (avian)															
NM_000853	GSTT1	chr22	22706138	22714284	-	Tum Sup	glutathione S-transferase theta 1															
NM_003073	SMARCB1	chr22	22459149	22506705	+	Tum Sup	SWI/SNF related, matrix associated, actin dependent regulator of chromatin, subfamily b, member 1															
NM_005940	MMP11	chr22	22445035	22456503	+	Tum Sup	matrix metallopeptidase 11 (stromelysin 3)															
NM_005207	CRKL	chr22	19601713	19638037	+	Onc	v-crk sarcoma virus CT10 oncogene homolog (avian)-like													19636699	1570_154_[illegible]	+
NM_004327	BCR	chr22	21852551	21990224	+	Other	breakpoint cluster region															
NM_001257387	CHEK2	chr22	27413730	27467822	-	Tum Sup	CHK2 checkpoint homolog (S. pombe)				27438106	AY516248	-									
NR_033319	MIAT	chr22	25383445	25402440	+	--	myocardial infarction associated transcript (non-protein coding) (Previous name: chromosome 22 open															
NM_001002879	THOC5	chr22	28234156	28279644	-	Tum Sup	chromosome 22 open reading frame 19															
NM_005243	EWSR1	chr22	27993997	28026515	+	Onc	Ewing sarcoma breakpoint region 1															
NM_181830	NF2	chr22	28329544	28424589	+	Tum Sup	neurofibromin 2 (bilateral acoustic neuroma)															
NM_032052	PATZ1	chr22	30051789	30072249	-	Onc	POZ (BTB) and AT hook containing zinc finger 1 (Previous name: zinc finger protein 278)															
NM_001007279	RRP22	chr22	28038922	28041748	-	Other	RAS-related on chromosome 22															

Table S1

tumor associated genes								HIV			MLV			MMTV			MMTV(SIN)			MMTV(SIN)arrest		
RefSeq	gene symbol	Chrom	txStart	txEnd	stra	Category	Gene name	position	integrant	strand	position	integrant	stran	positiopn	integrant	stran	position	integrant	stran	position	integrant	stran
NM_033016	PDGFB	chr22	37949630	37966860	-	Onc	platelet-derived growth factor beta polypeptide (simian sarcoma viral (v-sis) oncogene homolog)															
NM_001051	SSTR3	chr22	35932190	35938299	-	Other	somatostatin receptor 3															
NM_014248	RBX1	chr22	39677296	39698965	+	Tum Sup	ring-box 1															
NM_003932	ST13	chr22	39550546	39582633	-	Tum Sup	suppression of tumorigenicity 13 (colon carcinoma) (Hsp70 interacting protein)															
NR_044991	LINC00229	chr22	43380871	43399963	-	--	long intergenic non-protein coding RNA 229 (Previous names: chromosome 22 open reading frame 10, non															
NM_001167890	EGFL6	chrX	13497614	13561615	+	Other	EGF-like-domain, multiple 6															
NM_001170752	SRPX	chrX	37893531	37965121	-	Tum Sup	sushi-repeat-containing protein, X-linked (Previous names: sushi-repeat-containing protein, X chromo															
NM_001114377	FOXP3	chrX	48993840	49008232	-	Tum Sup	forkhead box P3 (Previous names: immune dysregulation, polyendocrinopathy, enteropathy, X-linked)															
NM_006521	TFE3	chrX	48771185	48787934	-	Tum Sup	transcription factor binding to IGHM enhancer 3															
NM_005229	ELK1	chrX	47379862	47394947	-	Onc	ELK1, member of ETS oncogene family															
NM_006743	RBM3	chrX	48317684	48324497	+	Onc	RNA binding motif (RNP1, RRM) protein 3 (Previous name: RNA binding motif protein 3)															
NM_001242362	MAGED4B	chrX	51944658	51952104	+	--	melanoma antigen family D, 4B MAGED4B: Approved															
NM_005938	MLLT7	chrX	70232751	70240109	+	Other	myeloid/lymphoid or mixed-lineage leukemia (trithorax homolog, Drosophila); translocated to, 7															
NM_014467	SRPX2	chrX	99785818	99812952	+	Other	sushi-repeat-containing protein, X-linked 2															
NM_001137554	MCTS1	chrX	119622579	119639042	+	Onc	malignant T cell amplified sequence 1															

Table S1

tumor associated genes								HIV			MLV			MMTV			MMTV(SIN)			MMTV(SIN)arrest		
RefSeq	gene symbol	Chrom	txStart	txEnd	stra	Category	Gene name	position	integrant	strand	position	integrant	stran	positiopn	integrant	stran	position	integrant	stran	position	integrant	stran
NM_001727	BRS3	chrX	135397790	135402264	+	Other	bombesin-like receptor 3															
NM_005369	MCF2	chrX	138491595	138552666	-	Onc	MCF.2 cell line derived transforming sequence															
NM_004065	CDR1	chrX	139693090	139694389	-	Other	cerebellar degeneration-related protein 1, 34kDa															
NM_004909	CSAG2	chrX	151627398	151628403	-	Other	CSAG family, member 2															
NM_001018025	MTCP1	chrX	153945502	153952741	-	Onc	mature T-cell proliferation 1															
NM_001256577	RPL10	chrX	153279764	153283874	+	Tum Sup	ribosomal protein L10															
NR_024048	TAZ	chrX	153293070	153303257	+	Other	tafazzin (cardiomyopathy, dilated 3A (X-linked); endocardial fibroelastosis 2; Barth syndrome)															
NM_013230	CD24	chrY	19611913	19614093	-	Onc	CD24 molecule (Previous name: CD24 antigen (small cell lung carcinoma cluster 4 antigen))															
NM_001197242	TSPY1	chrY	9914563	9917358	+	Onc	testis specific protein, Y-linked 1															
