# Supplementary material for: Mouse mammary tumor virus-based vector transduces non-dividing cells, enters the nucleus via a TNPO3-independent pathway and integrates in a less biased fashion than other retroviruses
Source: Retrovirology. 2014 Apr 30;11:34. doi: 10.1186/1742-4690-11-34 (PMC4098793; doi:10.1186/1742-4690-11-34)
Supplement: Additional file 4: Table S2 — Vector integrations and the tumor associated genes listed in The Cancer Gene Atlas. The same color coding as above was used. [file 1742-4690-11-34-S4.pdf]

Table S2

tumor associated genes								HIV				MLV				MMTV				MMTV(SIN)				MMTV(SIN)arrest			
rank	score	gene symbol	RefSEq	chrom	txStart	txEnd	strand	integrant	chr	position	strand	integrant	chr	position	strand	integrant	chr	position	strand	integrant	chr	position	strand	integrant	chr	position	strand
4031	1	HES4	NM_021170	chr1	924204	925415	-	.	.	.	.	.	.	.	.	.	.	.	.	.	.	.	.	.	.	.	.
5510	1	TNFRSF4	NM_003327	chr1	1136568	1139411	-	.	.	.	.	.	.	.	.	.	.	.	.	.	.	.	.	.	.	.	.
3109	1	B3GALT6	NM_080605	chr1	1157491	1160283	+	.	.	.	.	.	.	.	.	.	.	.	.	.	.	.	.	.	.	.	.
3632	1	DVL1	NM_004421	chr1	1260520	1274355	-	.	.	.	.	.	.	.	.	.	.	.	.	.	.	.	.	.	.	.	.
3104	1	AURKAIP1	NM_001127230	chr1	1298973	1300425	-	.	.	.	.	.	.	.	.	.	.	.	.	.	.	.	.	.	.	.	.
901	2,5	NADK	NM_001198993	chr1	1672530	1700157	-	.	.	.	.	.	.	.	.	.	.	.	.	.	.	.	.	.	.	.	.
3920	1	GNB1	NM_002074	chr1	1706584	1812386	-	.	.	.	.	.	.	.	.	.	.	.	.	.	.	.	.	.	.	.	.
3833	1	GABRD	NM_000815	chr1	1940627	1952052	+	.	.	.	.	.	.	.	.	.	.	.	.	.	.	.	.	.	.	.	.
145	5	PRKCZ	NM_001242874	chr1	1995284	2106694	+	BH609430	chr1	2074228	+	.	.	.	.	.	.	.	.	.	.	.	.	2249_196_1	chr1	2081278	-
7548	0,25	SKI	NM_003036	chr1	2149993	2231512	+	.	.	.	.	.	.	.	.	.	.	.	.	.	.	.	.	.	.	.	.
1594	2	PANK4	NM_018216	chr1	2429834	2447895	-	.	.	.	.	.	.	.	.	.	.	.	.	.	.	.	.	.	.	.	.
4032	1	HES5	NM_001010926	chr1	2450043	2451544	-	.	.	.	.	.	.	.	.	.	.	.	.	.	.	.	.	.	.	.	.
935	2,5	PRDM16	NM_199454	chr1	2975601	3345045	+	.	.	.	.	.	.	.	.	.	.	.	.	.	.	.	.	.	.	.	.
5535	1	TPRG1L	NM_182752	chr1	3531415	3536554	+	.	.	.	.	.	.	.	.	.	.	.	.	.	.	.	.	.	.	.	.
234	4,25	TP73	NM_001204187	chr1	3558988	3642625	+	.	.	.	.	.	.	.	.	.	.	.	.	.	.	.	.	.	.	.	.
3563	1	DFFB	NM_004402	chr1	3763704	3791853	+	.	.	.	.	.	.	.	.	.	.	.	.	.	.	.	.	.	.	.	.
7158	0,25	AJAP1	NM_001042478	chr1	4614964	4737714	+	.	.	.	.	.	.	.	.	.	.	.	.	.	.	.	.	.	.	.	.
2697	1,25	NPHP4	NM_015102	chr1	5845456	5975120	-	.	.	.	.	.	.	.	.	.	.	.	.	.	.	.	.	.	.	.	.
5929	0,75	KCNAB2	NM_001199860	chr1	6016934	6083840	+	.	.	.	.	.	.	.	.	.	.	.	.	.	.	.	.	.	.	.	.
6173	0,5	CHD5	NM_015557	chr1	6084433	6162781	-	.	.	.	.	.	.	.	.	.	.	.	.	.	.	.	.	.	.	.	.
503	3,25	RPL22	NM_000983	chr1	6167666	6182266	-	.	.	.	.	.	.	.	.	.	.	.	.	.	.	.	.	.	.	.	.
7516	0,25	RNF207	NM_207396	chr1	6188775	6203946	+	.	.	.	.	.	.	.	.	.	.	.	.	.	.	.	.	.	.	.	.
2619	1,25	ICMT	NM_012405	chr1	6203839	6218631	-	.	.	.	.	.	.	.	.	.	.	.	.	.	.	.	.	.	.	.	.
7343	0,25	HES3	NM_001024598	chr1	6226838	6228225	+	.	.	.	.	.	.	.	.	.	.	.	.	.	.	.	.	.	.	.	.
7333	0,25	GPR153	NM_207370	chr1	6229992	6243622	-	.	.	.	.	.	.	.	.	.	.	.	.	.	.	.	.	.	.	.	.
7150	0,25	ACOT7	NM_181865	chr1	6246918	6343351	-	BH609428	chr1	6282223	+	AY516311	chr1	6359651	+	.	.	.	.	.	.	.	.	.	.	.	.
7342	0,25	HES2	NM_019089	chr1	6397880	6402566	-	.	.	.	.	.	.	.	.	.	.	.	.	.	.	.	.	.	.	.	.
7303	0,25	ESPN	NM_031475	chr1	6407434	6443591	+	.	.	.	.	.	.	.	.	.	.	.	.	.	.	.	.	.	.	.	.
5509	1	TNFRSF25	NM_003790	chr1	6443800	6448842	-	.	.	.	.	.	.	.	.	.	.	.	.	.	.	.	.	.	.	.	.
5946	0,75	PLEKHG5	NM_001265592	chr1	6448738	6479743	-	.	.	.	.	.	.	.	.	.	.	.	.	.	.	.	.	.	.	.	.
2696	1,25	NOL9	NM_024654	chr1	6503993	6537245	-	.	.	.	.	.	.	.	.	.	.	.	.	.	.	.	.	.	.	.	.
2432	1,5	TAS1R1	NM_177540	chr1	6537924	6562404	+	.	.	.	.	.	.	.	.	.	.	.	.	.	.	.	.	.	.	.	.
2836	1,25	ZBTB48	NM_005341	chr1	6562649	6571927	+	.	.	.	.	.	.	.	.	.	.	.	.	.	.	.	.	.	.	.	.
2224	1,5	KLHL21	NM_014851	chr1	6573370	6585516	-	.	.	.	.	.	.	.	.	.	.	.	.	.	.	.	.	.	.	.	.
2709	1,25	PHF13	NM_153812	chr1	6596342	6606680	+	.	.	.	.	.	.	.	.	.	.	.	.	.	.	.	.	.	.	.	.
2799	1,25	THAP3	NM_001195752	chr1	6607796	6616229	+	.	.	.	.	.	.	.	.	.	.	.	.	.	.	.	.	.	.	.	.
2569	1,25	DNAJC11	NM_018198	chr1	6616814	6684553	-	.	.	.	.	.	.	.	.	.	.	.	.	.	.	.	.	.	.	.	.
1287	2	CAMTA1	NM_001242701	chr1	6767970	6870848	+	CL800682	chr1	6810001	-	.	.	.	.	_408_1_251	chr1	7161006	-	.	.	.	.	.	.	.	.
5666	1	VAMP3	NM_004781	chr1	7753915	7764079	+	.	.	.	.	.	.	.	.	.	.	.	.	.	.	.	.	.	.	.	.
4754	1	PER3	NM_016831	chr1	7767349	7827824	+	CL799630	chr1	7824049	+	.	.	.	.	.	.	.	.	.	.	.	.	.	.	.	.
1815	2	TNFRSF9	NM_001561	chr1	7898517	7925812	-	.	.	.	.	.	.	.	.	.	.	.	.	.	.	.	.	.	.	.	.
2704	1,25	PARK7	NM_007262	chr1	7944300	7967929	+	.	.	.	.	.	.	.	.	.	.	.	.	.	.	.	.	.	.	.	.
372	3,5	ERRFI1	NM_018948	chr1	7994365	8008980	-	.	.	.	.	.	.	.	.	.	.	.	.	.	.	.	.	.	.	.	.
6918	0,5	SLC45A1	NM_001080397	chr1	8306976	8326814	+	.	.	.	.	.	.	.	.	.	.	.	.	.	.	.	.	.	.	.	.
2372	1,5	RERE	NM_001042681	chr1	8335050	8800286	-	BH609425	chr1	8589373	-	.	.	.	.	.	.	.	.	.	.	.	.	.	.	.	.
2372	1,5	RERE	NM_001042681	chr1	8335050	8800286	-	BH609426	chr1	8696157	-	.	.	.	.	.	.	.	.	.	.	.	.	.	.	.	.
2372	1,5	RERE	NM_001042681	chr1	8335050	8800286	-	CL799958	chr1	8742925	-	.	.	.	.	.	.	.	.	.	.	.	.	.	.	.	.
824	2,5	ENO1	NM_001201483	chr1	8843645	8854222	-	AY517132.1	chr1	8850705	-	.	.	.	.	.	.	.	.	.	.	.	.	.	.	.	.
2526	1,25	CA6	NM_001270502	chr1	8928479	8957735	+	.	.	.	.	.	.	.	.	.	.	.	.	.	.	.	.	.	.	.	.
7556	0,25	SLC2A7	NM_207420	chr1	8985945	9008991	-	.	.	.	.	.	.	.	.	.	.	.	.	.	.	.	.	.	.	.	.
6915	0,5	SLC2A5	NM_001135585	chr1	9024013	9052474	-	.	.	.	.	.	.	.	.	.	.	.	.	.	.	.	.	.	.	.	.

Table S2

tumor associated genes								HIV				MLV				MMTV				MMTV(SIN)				MMTV(SIN)arrest			
rank	score	gene symbol	RefSEq	chrom	txStart	txEnd	strand	integrant	chr	position	strand	integrant	chr	position	strand	integrant	chr	position	strand	integrant	chr	position	strand	integrant	chr	position	strand
2606	1,25	GPR157	NM_024980	chr1	9087062	9111816	-	.	.	.	.	.	.	.	.	.	.	.	.	.	.	.	.	.	.	.	.
7425	0,25	MIR34A	NR_029610	chr1	9134313	9134423	-	.	.	.	.	.	.	.	.	.	.	.	.	.	.	.	.	.	.	.	.
4002	1	H6PD	NM_004285	chr1	9217449	9253981	+	BH609427	chr1	9226186	-	.	.	.	.	.	.	.	.	.	.	.	.	.	.	.	.
223	4,25	PIK3CD	NM_005026	chr1	9634376	9711759	+	.	.	.	.	.	.	.	.	.	.	.	.	.	.	.	.	.	.	.	.
4592	1	NMNAT1	NM_022787	chr1	9926072	9968143	+	.	.	.	.	.	.	.	.	.	.	.	.	.	.	.	.	.	.	.	.
6530	0,5	KIF1B	NM_015074	chr1	10193350	10364248	+	BH609422	chr1	10240553	-	.	.	.	.	.	.	.	.	.	.	.	.	.	.	.	.
4770	1	PGD	NM_002631	chr1	10381671	10402788	+	.	.	.	.	.	.	.	.	.	.	.	.	.	.	.	.	.	.	.	.
3001	1	APITD1	NM_199294	chr1	10412745	10425459	+	.	.	.	.	.	.	.	.	.	.	.	.	.	.	.	.	.	.	.	.
3562	1	DFFA	NM_213566	chr1	10443189	10455200	-	.	.	.	.	.	.	.	.	.	.	.	.	.	.	.	.	.	.	.	.
5350	1	SRM	NM_003132	chr1	11037235	11042678	-	.	.	.	.	.	.	.	.	.	.	.	.	.	.	.	.	.	.	.	.
2137	1,5	FBXO2	NM_012168	chr1	11631004	11637475	-	.	.	.	.	.	.	.	.	.	.	.	.	.	.	.	.	.	.	.	.
3759	1	FBXO6	NM_018438	chr1	11646736	11656996	+	.	.	.	.	.	.	.	.	.	.	.	.	.	.	.	.	.	.	.	.
4325	1	MAD2L2	NM_001127325	chr1	11657123	11674265	-	.	.	.	.	.	.	.	.	.	.	.	.	.	.	.	.	.	.	.	.
1115	2,25	MTHFR	NM_005957	chr1	11768373	11788747	-	.	.	.	.	.	.	.	.	.	.	.	.	.	.	.	.	.	.	.	.
7448	0,25	NPPA	NM_006172	chr1	11828353	11830427	-	.	.	.	.	.	.	.	.	.	.	.	.	.	.	.	.	.	.	.	.
4845	1	PLOD1	NM_000302	chr1	11917310	11958186	+	.	.	.	.	.	.	.	.	.	.	.	.	.	.	.	.	.	.	.	.
973	2,5	TNFRSF8	NM_152942	chr1	12108544	12126851	+	.	.	.	.	.	.	.	.	.	.	.	.	.	.	.	.	.	.	.	.
694	3	TNFRSF1B	NM_001066	chr1	12149646	12191864	+	.	.	.	.	.	.	.	.	.	.	.	.	.	.	.	.	.	.	.	.
1355	2	DHRS3	NM_004753	chr1	12550525	12600407	-	.	.	.	.	.	.	.	.	.	.	.	.	.	.	.	.	.	.	.	.
1128	2,25	PDPN	NM_198389	chr1	13782838	13817039	+	.	.	.	.	.	.	.	.	.	.	.	.	.	.	.	.	.	.	.	.
4933	1	PRDM2	NM_012231	chr1	13903936	14024161	+	.	.	.	.	.	.	.	.	.	.	.	.	.	.	.	.	.	.	.	.
6299	0,5	EFHD2	NM_024329	chr1	15608977	15629426	+	.	.	.	.	.	.	.	.	.	.	.	.	.	.	.	.	.	.	.	.
2532	1,25	CASP9	NM_001229	chr1	15691355	15723527	-	.	.	.	.	.	.	.	.	.	.	.	.	.	.	.	.	.	.	.	.
2924	1	AGMAT	NM_024758	chr1	15770780	15784192	-	.	.	.	.	.	.	.	.	.	.	.	.	.	.	.	.	.	.	.	.
1638	2	PLEKHM2	NM_015164	chr1	15883413	15933851	+	.	.	.	.	.	.	.	.	.	.	.	.	.	.	.	.	.	.	.	.
6345	0,5	FBLIM1	NM_001024215	chr1	15963580	15974302	+	.	.	.	.	.	.	.	.	.	.	.	.	.	.	.	.	.	.	.	.
307	4	SPEN	NM_015001	chr1	16046945	16139537	+	CL800580	chr1	16112187	+	.	.	.	.	.	.	.	.	.	.	.	.	.	.	.	.
307	4	SPEN	NM_015001	chr1	16046945	16139537	+	CL528781	chr1	16108156	-	.	.	.	.	.	.	.	.	.	.	.	.	.	.	.	.
307	4	SPEN	NM_015001	chr1	16046945	16139537	+	CL800616	chr1	16126846	+	.	.	.	.	.	.	.	.	.	.	.	.	.	.	.	.
7113	0,5	ZBTB17	NM_003443	chr1	16140950	16175214	-	.	.	.	.	.	.	.	.	.	.	.	.	.	.	.	.	.	.	.	.
2039	1,5	C1orf64	NM_178840	chr1	16203317	16205771	+	.	.	.	.	.	.	.	.	.	.	.	.	.	.	.	.	.	.	.	.
7306	0,25	FAM131C	NM_182623	chr1	16256850	16272714	-	.	.	.	.	.	.	.	.	.	.	.	.	.	.	.	.	.	.	.	.
324	3,75	EPHA2	NM_004431	chr1	16323418	16355169	-	.	.	.	.	.	.	.	.	.	.	.	.	.	.	.	.	.	.	.	.
7418	0,25	MFAP2	NM_001135248	chr1	17173585	17179760	-	.	.	.	.	.	.	.	.	.	.	.	.	.	.	.	.	.	.	.	.
3064	1	ATP13A2	NM_022089	chr1	17185039	17211010	-	.	.	.	.	.	.	.	.	.	.	.	.	.	.	.	.	.	.	.	.
231	4,25	SDHB	NM_003000	chr1	17217811	17253252	-	.	.	.	.	AY516335	chr1	17252157	-	.	.	.	.	.	.	.	.	.	.	.	.
5081	1	RCC2	NM_018715	chr1	17605837	17638837	-	.	.	.	.	.	.	.	.	.	.	.	.	.	.	.	.	.	.	.	.
6469	0,5	IGSF21	NM_032880	chr1	18306826	18577564	+	.	.	.	.	.	.	.	.	.	.	.	.	.	.	.	.	.	.	.	.
1127	2,25	PAX7	NM_001135254	chr1	18830086	18947947	+	.	.	.	.	.	.	.	.	.	.	.	.	.	.	.	.	.	.	.	.
2951	1	ALDH4A1	NM_003748	chr1	19070510	19101880	-	.	.	.	.	.	.	.	.	.	.	.	.	.	.	.	.	.	.	.	.
6005	0,5	AKR7A3	NM_012067	chr1	19481643	19487867	-	.	.	.	.	.	.	.	.	.	.	.	.	.	.	.	.	.	.	.	.
3275	1	CAPZB	NM_004930	chr1	19537853	19684653	-	.	.	.	.	.	.	.	.	.	.	.	.	.	.	.	.	.	.	.	.
4500	1	NBL1	NM_001204084	chr1	19842794	19857536	+	.	.	.	.	.	.	.	.	.	.	.	.	.	.	.	.	.	.	.	.
4830	1	PLA2G2E	NM_014589	chr1	20119386	20122697	-	.	.	.	.	.	.	.	.	.	.	.	.	.	.	.	.	.	.	.	.
746	2,75	PLA2G2A	NM_000300	chr1	20174510	20179519	-	.	.	.	.	.	.	.	.	.	.	.	.	.	.	.	.	.	.	.	.
1133	2,25	PLA2G5	NM_000929	chr1	20269287	20290981	+	.	.	.	.	.	.	.	.	.	.	.	.	.	.	.	.	.	.	.	.
4829	1	PLA2G2D	NM_012400	chr1	20311020	20318595	-	.	.	.	.	.	.	.	.	.	.	.	.	.	.	.	.	.	.	.	.
4831	1	PLA2G2F	NM_022819	chr1	20338409	20349466	+	.	.	.	.	.	.	.	.	.	.	.	.	.	.	.	.	.	.	.	.
2049	1,5	CAMK2N1	NM_018584	chr1	20681470	20685315	-	.	.	.	.	.	.	.	.	.	.	.	.	.	.	.	.	.	.	.	.
3304	1	CDA	NM_001785	chr1	20788030	20817987	+	.	.	.	.	.	.	.	.	.	.	.	.	.	.	.	.	.	.	.	.
1625	2	PINK1	NM_032409	chr1	20832534	20850591	+	.	.	.	.	.	.	.	.	.	.	.	.	.	.	.	.	.	.	.	.

Table S2

tumor associated genes								HIV				MLV				MMTV				MMTV(SIN)				MMTV(SIN)arrest			
rank	score	gene symbol	RefSEq	chrom	txStart	txEnd	strand	integrant	chr	position	strand	integrant	chr	position	strand	integrant	chr	position	strand	integrant	chr	position	strand	integrant	chr	position	strand
3545	1	DDOST	NM_005216	chr1	20850846	20860624	-	.	.	.	.	.	.	.	.	.	.	.	.	.	.	.	.	.	.	.	.
6529	0,5	KIF17	NM_001122819	chr1	20863093	20916904	-	.	.	.	.	.	.	.	.	.	.	.	.	.	.	.	.	.	.	.	.
6885	0,5	SH2D5	NM_001103160	chr1	20918811	20931720	-	.	.	.	.	.	.	.	.	.	.	.	.	.	.	.	.	.	.	.	.
7296	0,25	ECE1	NM_001113347	chr1	21416326	21478770	-	.	.	.	.	.	.	.	.	.	.	.	.	.	.	.	.	.	.	.	.
2971	1	ALPL	NM_001127501	chr1	21708444	21777492	+	.	.	.	.	.	.	.	.	.	.	.	.	.	.	.	.	.	.	.	.
2363	1,5	RAP1GAP	NM_002885	chr1	21795294	21868443	-	.	.	.	.	.	.	.	.	.	.	.	.	.	.	.	.	.	.	.	.
4097	1	HSPG2	NM_005529	chr1	22021323	22136337	-	.	.	.	.	.	.	.	.	.	.	.	.	.	.	.	.	.	.	.	.
63	6,5	CDC42	NM_044472	chr1	22251706	22289883	+	.	.	.	.	.	.	.	.	.	.	.	.	.	.	.	.	.	.	.	.
1183	2,25	WNT4	NM_030761	chr1	22316384	22342106	-	.	.	.	.	.	.	.	.	.	.	.	.	.	.	.	.	.	.	.	.
252	4	EPHA8	NM_020526	chr1	22762590	22802674	+	.	.	.	.	.	.	.	.	.	.	.	.	.	.	.	.	.	.	.	.
6085	0,5	C1QA	NM_015991	chr1	22835704	22838762	+	.	.	.	.	.	.	.	.	.	.	.	.	.	.	.	.	.	.	.	.
253	4	EPHB2	NM_004442	chr1	22909917	23114410	+	.	.	.	.	.	.	.	.	.	.	.	.	.	.	.	.	.	.	.	.
7363	0,25	HTR1D	NM_000864	chr1	23390974	23393809	-	.	.	.	.	.	.	.	.	.	.	.	.	.	.	.	.	.	.	.	.
2117	1,5	E2F2	NM_004091	chr1	23705506	23730299	-	.	.	.	.	.	.	.	.	.	.	.	.	.	.	.	.	.	.	.	.
1923	1,75	ID3	NM_002167	chr1	23757007	23758872	-	.	.	.	.	.	.	.	.	.	.	.	.	.	.	.	.	.	.	.	.
4372	1	MDS2	NR_027042	chr1	23826410	23839643	+	AY517143.1	chr1	23831737	+	.	.	.	.	.	.	.	.	.	.	.	.	.	.	.	.
4322	1	LYPLA2	NM_007260	chr1	23990232	23994616	+	.	.	.	.	.	.	.	.	.	.	.	.	.	.	.	.	.	.	.	.
3839	1	GALE	NM_000403	chr1	23994675	23999621	-	.	.	.	.	.	.	.	.	.	.	.	.	.	.	.	.	.	.	.	.
4057	1	HMGCL	NM_001166059	chr1	24000953	24024536	-	.	.	.	.	.	.	.	.	.	.	.	.	.	.	.	.	.	.	.	.
3813	1	FUCA1	NM_000147	chr1	24044158	24067446	-	.	.	.	.	.	.	.	.	.	.	.	.	.	.	.	.	.	.	.	.
2281	1,5	NIPAL3	NM_020448	chr1	24614831	24672060	+	.	.	.	.	.	.	.	.	.	.	.	.	.	.	.	.	.	.	.	.
6591	0,5	MAN1C1	NM_020379	chr1	25816545	25983845	+	.	.	.	.	.	.	.	.	.	.	.	.	.	.	.	.	.	.	.	.
1970	1,75	STMN1	NM_001145454	chr1	26083263	26105580	-	.	.	.	.	.	.	.	.	.	.	.	.	.	.	.	.	.	.	.	.
4685	1	PAFAH2	NM_000437	chr1	26158844	26197235	-	.	.	.	.	.	.	.	.	.	.	.	.	.	.	.	.	.	.	.	.
2128	1,5	EXTL1	NM_004455	chr1	26220857	26235541	+	.	.	.	.	.	.	.	.	.	.	.	.	.	.	.	.	.	.	.	.
4745	1	PDIK1L	NM_152835	chr1	26310854	26324626	+	.	.	.	.	.	.	.	.	.	.	.	.	.	.	.	.	.	.	.	.
3404	1	CNKSR1	NR_023345	chr1	26376568	26388962	+	.	.	.	.	.	.	.	.	.	.	.	.	.	.	.	.	.	.	.	.
7352	0,25	HMGN2	NM_005517	chr1	26671488	26675720	+	.	.	.	.	.	.	.	.	.	.	.	.	.	.	.	.	.	.	.	.
300	4	RPS6KA1	NM_002953	chr1	26728835	26774107	+	.	.	.	.	.	.	.	.	.	.	.	.	.	.	.	.	.	.	.	.
4804	1	PIGV	NM_017837	chr1	26987040	26997481	+	.	.	.	.	.	.	.	.	.	.	.	.	.	.	.	.	.	.	.	.
7119	0,5	ZDHHC18	NM_032283	chr1	27025787	27054798	+	.	.	.	.	.	.	.	.	.	.	.	.	.	.	.	.	.	.	.	.
1163	2,25	SFN	NM_006142	chr1	27062219	27063534	+	.	.	.	.	.	.	.	.	.	.	.	.	.	.	.	.	.	.	.	.
3939	1	GPATCH3	NM_022078	chr1	27089565	27099549	-	.	.	.	.	.	.	.	.	.	.	.	.	.	.	.	.	.	.	.	.
1582	2	NR0B2	NM_021969	chr1	27110561	27113154	-	.	.	.	.	.	.	.	.	.	.	.	.	.	.	.	.	.	.	.	.
4642	1	NUDC	NM_006600	chr1	27120799	27145949	+	.	.	.	.	.	.	.	.	.	.	.	.	.	.	.	.	.	.	.	.
2240	1,5	MAP3K6	NM_004672	chr1	27554256	27565924	-	.	.	.	.	.	.	.	.	.	.	.	.	.	.	.	.	.	.	.	.
2587	1,25	FGR	NM_001042729	chr1	27811387	27825693	-	.	.	.	.	.	.	.	.	.	.	.	.	.	.	.	.	.	.	.	.
1669	2	PPP1R8	NM_138558	chr1	28029911	28050770	+	.	.	.	.	.	.	.	.	.	.	.	.	.	.	.	.	.	.	.	.
5155	1	RPA2	NM_002946	chr1	28090635	28113823	-	.	.	.	.	.	.	.	.	.	.	.	.	.	.	.	.	.	.	.	.
5212	1	SESN2	NM_031459	chr1	28458549	28481589	+	.	.	.	.	.	.	.	.	.	.	.	.	.	.	.	.	.	.	.	.
4778	1	PHACTR4	NM_001048183	chr1	28568679	28699468	+	CL799913	chr1	28633575	+	.	.	.	.	.	.	.	.	.	.	.	.	.	.	.	.
6310	0,5	EPB41	NM_203342	chr1	29086189	29319145	+	BH609416	chr1	29290129	+	.	.	.	.	.	.	.	.	.	.	.	.	.	.	.	.
5493	1	TMEM200B	NM_001171868	chr1	29318523	29321600	-	.	.	.	.	.	.	.	.	.	.	.	.	.	.	.	.	.	.	.	.
4374	1	MECR	NM_001024732	chr1	29391971	29430041	-	.	.	.	.	.	.	.	.	.	.	.	.	.	.	.	.	.	.	.	.
1703	2	PTPRU	NM_001195001	chr1	29435614	29525912	+	.	.	.	.	.	.	.	.	.	.	.	.	.	.	.	.	.	.	.	.
6547	0,5	LAPTM5	NM_006762	chr1	30977901	31003270	-	.	.	.	.	.	.	.	.	.	.	.	.	.	.	.	.	.	.	.	.
2390	1,5	SDC3	NM_014654	chr1	31114899	31154067	-	.	.	.	.	.	.	.	.	.	.	.	.	.	.	.	.	.	.	.	.
6672	0,5	NKAIN1	NM_024522	chr1	31425178	31485321	-	.	.	.	.	.	.	.	.	.	.	.	.	.	.	.	.	.	.	.	.
3712	1	FABP3	NM_004102	chr1	31610686	31618510	-	.	.	.	.	.	.	.	.	.	.	.	.	.	.	.	.	.	.	.	.
2765	1,25	SERINC2	NM_018565	chr1	31659246	31680114	+	.	.	.	.	.	.	.	.	.	.	.	.	.	.	.	.	.	.	.	.
6199	0,5	COL16A1	NM_001856	chr1	31890434	31942355	-	.	.	.	.	.	.	.	.	.	.	.	.	.	.	.	.	.	.	.	.

Table S2

tumor associated genes								HIV				MLV				MMTV				MMTV(SIN)				MMTV(SIN)arrest			
rank	score	gene symbol	RefSEq	chrom	txStart	txEnd	strand	integrant	chr	position	strand	integrant	chr	position	strand	integrant	chr	position	strand	integrant	chr	position	strand	integrant	chr	position	strand
2022	1,5	BAI2	NM_001703	chr1	31965304	32002235	-	.	.	.	.	.	.	.	.	.	.	.	.	.	.	.	.	.	.	.	.
2731	1,25	PTP4A2	NM_080391	chr1	32144608	32176575	-	.	.	.	.	.	.	.	.	.	.	.	.	.	.	.	.	.	.	.	.
392	3,5	LCK	NM_001042771	chr1	32512298	32524353	+	.	.	.	.	.	.	.	.	.	.	.	.	.	.	.	.	.	.	.	.
80	6,25	HDAC1	NM_004964	chr1	32530294	32571811	+	BH609415	chr1	32551320	+	.	.	.	.	.	.	.	.	.	.	.	.	.	.	.	.
6596	0,5	MARCKSL1	NM_023009	chr1	32572016	32574427	-	.	.	.	.	.	.	.	.	.	.	.	.	.	.	.	.	.	.	.	.
5576	1	TSSK3	NM_052841	chr1	32600448	32602511	+	.	.	.	.	.	.	.	.	.	.	.	.	.	.	.	.	.	.	.	.
5720	1	YARS	NM_003680	chr1	33013426	33056220	-	.	.	.	.	.	.	.	.	.	.	.	.	.	.	.	.	.	.	.	.
4075	1	HPCA	NM_002143	chr1	33124684	33132834	+	.	.	.	.	.	.	.	.	.	.	.	.	.	.	.	.	.	.	.	.
1231	2	AK2	NM_001199199	chr1	33246127	33275099	-	.	.	.	.	.	.	.	.	.	.	.	.	.	.	.	.	.	.	.	.
2906	1	ADC	NM_052998	chr1	33319300	33358582	+	.	.	.	.	.	.	.	.	.	.	.	.	.	.	.	.	.	.	.	.
6738	0,5	PHC2	NM_198040	chr1	33561810	33613781	-	.	.	.	.	.	.	.	.	.	.	.	.	.	.	.	.	.	.	.	.
7329	0,25	GJA4	NM_002060	chr1	35031185	35033935	+	.	.	.	.	.	.	.	.	.	.	.	.	.	.	.	.	.	.	.	.
1750	2	SFPQ	NM_005066	chr1	35421787	35431330	-	.	.	.	.	.	.	.	.	.	.	.	.	.	.	.	.	.	.	.	.
2837	1,25	ZMYM4	NM_005095	chr1	35507154	35660132	+	CL800333	chr1	35564297	-	.	.	.	.	.	.	.	.	.	.	.	.	.	.	.	.
4506	1	NCDN	NM_014284	chr1	35795979	35804967	+	.	.	.	.	.	.	.	.	.	.	.	.	.	.	.	.	.	.	.	.
1314	2	CLSPN	NM_001190481	chr1	35970299	36008138	-	.	.	.	.	.	.	.	.	.	.	.	.	.	.	.	.	.	.	.	.
6204	0,5	COL8A2	NM_005202	chr1	36333429	36338437	-	.	.	.	.	.	.	.	.	.	.	.	.	.	.	.	.	.	.	.	.
1808	2	THRAP3	NM_005119	chr1	36462603	36543544	+	CL528787	chr1	36471033	-	.	.	.	.	.	.	.	.	.	.	.	.	.	.	.	.
1808	2	THRAP3	NM_005119	chr1	36462603	36543544	+	BH609413	chr1	36476686	+	.	.	.	.	.	.	.	.	.	.	.	.	.	.	.	.
5390	1	STK40	NM_032017	chr1	36577811	36624072	-	.	.	.	.	.	.	.	.	.	.	.	.	.	.	.	.	.	.	.	.
4440	1	MRPS15	NM_031280	chr1	36693948	36702627	-	.	.	.	.	.	.	.	.	.	.	.	.	.	.	.	.	.	.	.	.
1326	2	CSF3R	NM_156039	chr1	36704230	36721502	-	.	.	.	.	.	.	.	.	.	.	.	.	.	.	.	.	.	.	.	.
7561	0,25	SNIP1	NM_024700	chr1	37772636	37792532	-	.	.	.	.	.	.	.	.	.	.	.	.	.	.	.	.	.	.	.	.
2167	1,5	GNL2	NM_013285	chr1	37804999	37834173	-	CL529720	chr1	37818110	+	.	.	.	.	.	.	.	.	.	.	.	.	.	.	.	.
2167	1,5	GNL2	NM_013285	chr1	37804999	37834173	-	CL528788	chr1	37825577	+	.	.	.	.	.	.	.	.	.	.	.	.	.	.	.	.
3308	1	CDCA8	NM_018101	chr1	37930659	37947978	+	.	.	.	.	.	.	.	.	.	.	.	.	.	.	.	.	.	.	.	.
723	2,75	EPHA10	NM_173641	chr1	37998531	38003411	-	.	.	.	.	.	.	.	.	.	.	.	.	.	.	.	.	.	.	.	.
602	3	INPP5B	NM_005540	chr1	38098955	38185316	-	.	.	.	.	.	.	.	.	.	.	.	.	.	.	.	.	.	.	.	.
5167	1	RRAGC	NM_022157	chr1	39077605	39097927	-	.	.	.	.	.	.	.	.	.	.	.	.	.	.	.	.	.	.	.	.
4549	1	NDUFS5	NM_004552	chr1	39264553	39272895	+	.	.	.	.	.	.	.	.	.	.	.	.	.	.	.	.	.	.	.	.
1446	2	HEYL	NM_014571	chr1	39861689	39877935	-	.	.	.	.	.	.	.	.	.	.	.	.	.	.	.	.	2236_195_1	chr1	39867384	-
4632	1	NT5C1A	NM_032526	chr1	39897379	39910297	-	.	.	.	.	.	.	.	.	.	.	.	.	.	.	.	.	.	.	.	.
2191	1,5	HPCAL4	NM_016257	chr1	39917231	39929676	-	.	.	.	.	.	.	.	.	.	.	.	.	.	.	.	.	.	.	.	.
7488	0,25	PPIE	NM_203456	chr1	39977103	40002173	+	.	.	.	.	.	.	.	.	.	.	.	.	.	.	.	.	.	.	.	.
4677	1	OXCT2	NM_022120	chr1	40007783	40009607	-	.	.	.	.	.	.	.	.	.	.	.	.	.	.	.	.	.	.	.	.
7604	0,25	TRIT1	NM_017646	chr1	40079292	40121764	-	.	.	.	.	.	.	.	.	.	.	.	.	.	.	.	.	.	.	.	.
220	4,25	MYCL1	NM_005376	chr1	40137870	40140274	-	.	.	.	.	.	.	.	.	.	.	.	.	.	.	.	.	.	.	.	.
4929	1	PPT1	NM_001142604	chr1	40310968	40335729	-	.	.	.	.	.	.	.	.	.	.	.	.	.	.	.	.	.	.	.	.
7515	0,25	RLF	NM_012421	chr1	40399627	40479180	+	.	.	.	.	.	.	.	.	.	.	.	.	.	.	.	.	.	.	.	.
7139	0,5	ZNF643	NM_023070	chr1	40688923	40701977	+	.	.	.	.	.	.	.	.	.	.	.	.	.	.	.	.	.	.	.	.
3557	1	DEM1	NM_022774	chr1	40747019	40754801	+	.	.	.	.	.	.	.	.	.	.	.	.	.	.	.	.	.	.	.	.
6820	0,5	RIMS3	NM_014747	chr1	40858938	40903911	-	.	.	.	.	.	.	.	.	.	.	.	.	.	.	.	.	.	.	.	.
3374	1	CITED4	NM_133467	chr1	41099314	41100605	-	.	.	.	.	.	.	.	.	.	.	.	.	.	.	.	.	.	.	.	.
3646	1	EDN2	NM_001956	chr1	41717032	41722931	-	.	.	.	.	.	.	.	.	.	.	.	.	.	.	.	.	.	.	.	.
4050	1	HIVEP3	NM_024503	chr1	41744622	42156965	-	BH609412	chr1	41892072	+	.	.	.	.	.	.	.	.	.	.	.	.	2919_263_1	chr1	42267193	-
6417	0,5	GUCA2B	NM_007102	chr1	42391678	42394082	+	.	.	.	.	.	.	.	.	.	.	.	.	.	.	.	.	.	.	.	.
1665	2	PPCS	NM_024664	chr1	42694759	42698673	+	.	.	.	.	.	.	.	.	.	.	.	.	.	.	.	.	.	.	.	.
7108	0,5	YBX1	NM_004559	chr1	42920652	42940607	+	.	.	.	.	.	.	.	.	.	.	.	.	.	.	.	.	.	.	.	.
505	3,25	SLC2A1	NM_006516	chr1	43163632	43197434	-	.	.	.	.	.	.	.	.	.	.	.	.	.	.	.	.	.	.	.	.
7029	0,5	TMEM125	NM_144626	chr1	43508251	43512260	+	.	.	.	.	.	.	.	.	.	.	.	.	.	.	.	.	.	.	.	.
761	2,75	TIE1	NM_005424	chr1	43539152	43561368	+	.	.	.	.	.	.	.	.	.	.	.	.	.	.	.	.	.	.	.	.

Table S2

tumor associated genes								HIV				MLV				MMTV				MMTV(SIN)				MMTV(SIN)arrest			
rank	score	gene symbol	RefSEq	chrom	txStart	txEnd	strand	integrant	chr	position	strand	integrant	chr	position	strand	integrant	chr	position	strand	integrant	chr	position	strand	integrant	chr	position	strand
218	4,25	MPL	NM_005373	chr1	43576061	43592722	+	.	.	.	.	.	.	.	.	.	.	.	.	.	.	.	.	.	.	.	.
1889	1,75	CDC20	NM_001255	chr1	43597212	43601460	+	.	.	.	.	.	.	.	.	.	.	.	.	.	.	.	.	.	.	.	.
4103	1	HYI	NM_031207	chr1	43689260	43692247	-	.	.	.	.	.	.	.	.	.	.	.	.	.	.	.	.	.	.	.	.
343	3,75	PTPRF	NM_130440	chr1	43769133	43861930	+	.	.	.	.	.	.	.	.	.	.	.	.	.	.	.	.	.	.	.	.
4208	1	KDM4A	NM_014663	chr1	43888383	43943776	+	.	.	.	.	.	.	.	.	.	.	.	.	.	.	.	.	.	.	.	.
2788	1,25	ST3GAL3	NR_073021	chr1	43945790	44169424	+	.	.	.	.	.	.	.	.	.	.	.	.	.	.	.	.	.	.	.	.
4156	1	IPO13	NM_014652	chr1	44185064	44206281	+	.	.	.	.	.	.	.	.	.	.	.	.	.	.	.	.	.	.	.	.
3084	1	ATP6V0B	NM_004047	chr1	44213188	44216559	+	.	.	.	.	.	.	.	.	.	.	.	.	.	.	.	.	.	.	.	.
3118	1	B4GALT2	NM_001005417	chr1	44218763	44229430	+	.	.	.	.	.	.	.	.	.	.	.	.	.	.	.	.	.	.	.	.
6924	0,5	SLC6A9	NM_201649	chr1	44234741	44255599	-	.	.	.	.	.	.	.	.	.	.	.	.	.	.	.	.	.	.	.	.
6269	0,5	DMAP1	NM_001034024	chr1	44451711	44458938	+	.	.	.	.	.	.	.	.	.	.	.	.	.	.	.	.	.	.	.	.
874	2,5	KIF2C	NM_006845	chr1	44978076	45006025	+	.	.	.	.	.	.	.	.	.	.	.	.	.	.	.	.	.	.	.	.
748	2,75	PLK3	NM_004073	chr1	45038622	45044254	+	CL799910	chr1	45042646	+	.	.	.	.	.	.	.	.	.	.	.	.	.	.	.	.
294	4	PTCH2	NM_003738	chr1	45060524	45081203	-	.	.	.	.	.	.	.	.	.	.	.	.	.	.	.	.	.	.	.	.
5651	1	UROD	NM_000374	chr1	45250391	45253928	+	.	.	.	.	.	.	.	.	.	.	.	.	.	.	.	.	.	.	.	.
219	4,25	MUTYH	NM_001048174	chr1	45567500	45578216	-	.	.	.	.	.	.	.	.	.	.	.	.	.	.	.	.	.	.	.	.
5459	1	TESK2	NM_007170	chr1	45582141	45729427	-	CL528790	chr1	45702907	-	.	.	.	.	.	.	.	.	.	.	.	.	.	.	.	.
6766	0,5	PRDX1	NM_002574	chr1	45749293	45760197	-	BH609410	chr1	45757454	-	.	.	.	.	.	.	.	.	.	.	.	.	.	.	.	.
2943	1	AKR1A1	NM_001202413	chr1	45789041	45808310	+	.	.	.	.	.	.	.	.	.	.	.	.	.	.	.	.	.	.	.	.
4498	1	NASP	NM_152298	chr1	45822246	45857165	+	.	.	.	.	.	.	.	.	.	.	.	.	.	.	.	.	.	.	.	.
4360	1	MAST2	NM_015112	chr1	46041871	46274383	+	CL799809	chr1	46262703	-	.	.	.	.	.	.	.	.	.	.	.	.	.	.	.	.
1951	1,75	PIK3R3	NM_003629	chr1	46278398	46370967	-	BH609411	chr1	46333315	+	.	.	.	.	.	.	.	.	.	.	.	.	.	.	.	.
4874	1	POMGNT1	NM_001243766	chr1	46426939	46458564	-	.	.	.	.	.	.	.	.	.	.	.	.	.	.	.	.	.	.	.	.
663	3	RAD54L	NM_003579	chr1	46485953	46516732	+	.	.	.	.	.	.	.	.	.	.	.	.	.	.	.	.	.	.	.	.
4307	1	LRRC41	NM_006369	chr1	46516658	46541625	-	.	.	.	.	.	.	.	.	.	.	.	.	.	.	.	.	.	.	.	.
1846	2	UQCRH	NM_006004	chr1	46541966	46555034	+	.	.	.	.	.	.	.	.	.	.	.	.	.	.	.	.	.	.	.	.
4406	1	MKNK1	NM_003684	chr1	46795665	46842553	-	CL528791	chr1	46815406	-	.	.	.	.	.	.	.	.	.	.	.	.	.	.	.	.
3515	1	CYP4A11	NM_000778	chr1	47167432	47179743	-	.	.	.	.	.	.	.	.	.	.	.	.	.	.	.	.	.	.	.	.
3517	1	CYP4X1	NM_178033	chr1	47261826	47289010	+	.	.	.	.	.	.	.	.	.	.	.	.	.	.	.	.	.	.	.	.
3518	1	CYP4Z1	NM_178134	chr1	47305746	47356579	+	.	.	.	.	.	.	.	.	.	.	.	.	.	.	.	.	.	.	.	.
3516	1	CYP4A22	NM_001010969	chr1	47375693	47387113	+	.	.	.	.	.	.	.	.	.	.	.	.	.	.	.	.	.	.	.	.
347	3,75	TAL1	NM_003189	chr1	47454549	47468030	-	.	.	.	.	.	.	.	.	.	.	.	.	.	.	.	.	.	.	.	.
507	3,25	STIL	NM_001048166	chr1	47488397	47552406	-	.	.	.	.	.	.	.	.	.	.	.	.	.	.	.	.	.	.	.	.
3400	1	CMPK1	NM_016308	chr1	47572055	47617098	+	.	.	.	.	.	.	.	.	.	.	.	.	.	.	.	.	.	.	.	.
2123	1,5	ELAVL4	NM_001144774	chr1	50347180	50440127	+	.	.	.	.	.	.	.	.	.	.	.	.	.	.	.	.	.	.	.	.
3593	1	DMRTA2	NM_032110	chr1	50655809	50661707	-	.	.	.	.	.	.	.	.	.	.	.	.	.	.	.	.	.	.	.	.
2582	1,25	FAF1	NM_007051	chr1	50679522	51198524	-	.	.	.	.	.	.	.	.	.	.	.	.	.	.	.	.	.	.	.	.
74	6,25	CDKN2C	NM_001262	chr1	51206954	51212894	+	.	.	.	.	.	.	.	.	.	.	.	.	.	.	.	.	.	.	.	.
725	2,75	EPS15	NM_001159969	chr1	51592522	51660381	-	CL529726	chr1	51699835	+	.	.	.	.	.	.	.	.	.	.	.	.	.	.	.	.
725	2,75	EPS15	NM_001159969	chr1	51592522	51660381	-	AY517024.1	chr1	51751188	+	.	.	.	.	.	.	.	.	.	.	.	.	.	.	.	.
725	2,75	EPS15	NM_001159969	chr1	51592522	51660381	-	AY517026.1	chr1	51754559	-	.	.	.	.	.	.	.	.	.	.	.	.	.	.	.	.
4619	1	NRD1	NM_001101662	chr1	52027453	52117197	-	.	.	.	.	.	.	.	.	.	.	.	.	.	.	.	.	.	.	.	.
5033	1	RAB3B	NM_002867	chr1	52146215	52229024	-	.	.	.	.	.	.	.	.	.	.	.	.	.	.	.	.	.	.	.	.
1869	2	ZFYVE9	NM_004799	chr1	52380633	52584946	+	.	.	.	.	.	.	.	.	.	.	.	.	.	.	.	.	.	.	.	.
6407	0,5	GPX7	NM_015696	chr1	52840630	52847311	+	.	.	.	.	.	.	.	.	.	.	.	.	.	.	.	.	.	.	.	.
3643	1	ECHDC2	NM_001198962	chr1	53134169	53160034	-	.	.	.	.	.	.	.	.	.	.	.	.	.	.	.	.	.	.	.	.
3454	1	CPT2	NM_000098	chr1	53434688	53452457	+	.	.	.	.	.	.	.	.	.	.	.	.	.	.	.	.	.	.	.	.
2882	1	ACOT11	NM_015547	chr1	54786394	54873005	+	.	.	.	.	.	.	.	.	.	.	.	.	.	.	.	.	.	.	.	.
4701	1	PARS2	NM_152268	chr1	54995158	55002814	-	.	.	.	.	.	.	.	.	.	.	.	.	.	.	.	.	.	.	.	.
1353	2	DHCR24	NM_014762	chr1	55087887	55125509	-	.	.	.	.	.	.	.	.	.	.	.	.	.	.	.	.	.	.	.	.
1664	2	PPAP2B	NM_003713	chr1	56733006	56817845	-	.	.	.	.	.	.	.	.	.	.	.	.	.	.	.	.	.	.	.	.

Table S2

tumor associated genes								HIV				MLV				MMTV				MMTV(SIN)				MMTV(SIN)arrest			
rank	score	gene symbol	RefSEq	chrom	txStart	txEnd	strand	integrant	chr	position	strand	integrant	chr	position	strand	integrant	chr	position	strand	integrant	chr	position	strand	integrant	chr	position	strand
1682	2	PRKAA2	NM_006252	chr1	56883577	56953596	+	.	.	.	.	.	.	.	.	.	.	.	.	.	.	.	.	.	.	.	.
3522	1	DAB1	NM_021080	chr1	57236166	58488799	-	.	.	.	.	.	.	.	.	_282_1_185	chr1	57527824	-	78_1_2320_9	chr1	57560570	+	.	.	.	.
3522	1	DAB1	NM_021080	chr1	57236166	58488799	-	.	.	.	.	.	.	.	.	_282_1_185	chr1	57527824	-	1568_169	chr1	57333918	-	.	.	.	.
390	3,5	JUN	NM_002228	chr1	59019050	59022373	-	.	.	.	.	.	.	.	.	.	.	.	.	.	.	.	.	.	.	.	.
3777	1	FGGY	NM_001244714	chr1	59548337	60000990	+	BH609407	chr1	59822576	-	.	.	.	.	.	.	.	.	.	.	.	.	2349_202_1	chr1	59602238	-
3777	1	FGGY	NM_001244714	chr1	59548337	60000990	+	AY517446.1	chr1	59537477	+	.	.	.	.	.	.	.	.	.	.	.	.	2349_202_1	chr1	59602238	-
6444	0,5	HOOK1	NM_015888	chr1	60053120	60114638	+	.	.	.	.	.	.	.	.	.	.	.	.	.	.	.	.	.	.	.	.
1340	2	CYP2J2	NM_000775	chr1	60131567	60165011	-	.	.	.	.	.	.	.	.	.	.	.	.	.	.	.	.	.	.	.	.
5652	1	USP1	NM_003368	chr1	62674970	62690063	+	.	.	.	.	.	.	.	.	.	.	.	.	.	.	.	.	.	.	.	.
6280	0,5	DOCK7	NM_033407	chr1	62692984	62926557	-	CL800808	chr1	62853259	+	.	.	.	.	.	.	.	.	.	.	.	.	.	.	.	.
2962	1	ALG6	NM_013339	chr1	63605848	63676821	+	.	.	.	.	.	.	.	.	.	.	.	.	.	.	.	.	.	.	.	.
1479	2	ITGB3BP	NM_001206739	chr1	63679028	63761532	-	.	.	.	.	.	.	.	.	.	.	.	.	.	.	.	.	.	.	.	.
1611	2	PGM1	NM_002633	chr1	63831534	63898505	+	.	.	.	.	.	.	.	.	.	.	.	.	.	.	.	.	.	.	.	.
502	3,25	ROR1	NM_005012	chr1	64012277	64419767	+	.	.	.	.	.	.	.	.	.	.	.	.	.	.	.	.	.	.	.	.
1486	2	JAK1	NM_002227	chr1	65071493	65204775	-	.	.	.	.	.	.	.	.	.	.	.	.	.	.	.	.	.	.	.	.
6276	0,5	DNAJC6	NM_014787	chr1	65502964	65654140	+	.	.	.	.	.	.	.	.	_420_1_262	chr1	65618277	+	.	.	.	.	.	.	.	.
4277	1	LEPR	NM_001198689	chr1	65763959	65873699	+	.	.	.	.	AY516411	chr1	65765939	+	.	.	.	.	.	.	.	.	.	.	.	.
4733	1	PDE4B	NM_001037339	chr1	66570380	66612850	+	CL528793	chr1	66580193	-	.	.	.	.	.	.	.	.	.	.	.	.	.	.	.	.
5222	1	SGIP1	NM_032291	chr1	66772412	66983356	+	CL528794	chr1	66794186	+	.	.	.	.	.	.	.	.	.	.	.	.	.	.	.	.
6472	0,5	IL23R	NM_144701	chr1	67404756	67498238	+	.	.	.	.	.	.	.	.	.	.	.	.	.	.	.	.	.	.	.	.
2200	1,5	IL12RB2	NM_001258214	chr1	67545634	67635171	+	CL529715	chr1	67564689	-	.	.	.	.	.	.	.	.	.	.	.	.	.	.	.	.
1049	2,25	GADD45A	NM_001199741	chr1	67923447	67926609	+	.	.	.	.	.	.	.	.	.	.	.	.	.	.	.	.	.	.	.	.
102	5,5	DIRAS3	NM_004675	chr1	68284232	68289048	-	.	.	.	.	.	.	.	.	.	.	.	.	.	.	.	.	.	.	.	.
5952	0,75	RPE65	NM_000329	chr1	68667094	68688230	-	.	.	.	.	.	.	.	.	.	.	.	.	.	.	.	.	.	.	.	.
3558	1	DEPDC1	NM_017779	chr1	68712422	68735387	-	CL528795	chr1	68718498	-	.	.	.	.	.	.	.	.	.	.	.	.	.	.	.	.
3558	1	DEPDC1	NM_017779	chr1	68712422	68735387	-	CL529442	chr1	68727925	-	.	.	.	.	.	.	.	.	.	.	.	.	.	.	.	.
886	2,5	LRRC7	NM_020794	chr1	69998445	70361759	+	.	.	.	.	.	.	.	.	.	.	.	.	.	.	.	.	.	.	.	.
3482	1	CTH	NM_001902	chr1	70649488	70678122	+	.	.	.	.	.	.	.	.	.	.	.	.	.	.	.	.	.	.	.	.
2728	1,25	PTGER3	NM_198716	chr1	71090623	71286079	-	.	.	.	.	.	.	.	.	_302_1_193	chr1	71188628	-	.	.	.	.	.	.	.	.
6661	0,5	NEGR1	NM_173808	chr1	71641212	72520865	-	CL800425	chr1	72408172	+	.	.	.	.	.	.	.	.	.	.	.	.	.	.	.	.
6661	0,5	NEGR1	NM_173808	chr1	71641212	72520865	-	CL800620	chr1	72077225	+	.	.	.	.	.	.	.	.	.	.	.	.	.	.	.	.
6661	0,5	NEGR1	NM_173808	chr1	71641212	72520865	-	CL800752	chr1	72018817	+	.	.	.	.	.	.	.	.	.	.	.	.	.	.	.	.
3799	1	FPGT	NM_003838	chr1	74436483	74446736	+	.	.	.	.	.	.	.	.	.	.	.	.	.	.	.	.	.	.	.	.
1817	2	TNNI3K	NM_015978	chr1	74473658	74782704	+	.	.	.	.	.	.	.	.	.	.	.	.	.	.	.	.	.	.	.	.
7256	0,25	CRYZ	NM_001889	chr1	74943759	74971680	-	.	.	.	.	.	.	.	.	.	.	.	.	.	.	.	.	.	.	.	.
1200	2	ACADM	NM_001127328	chr1	75962630	76001943	+	.	.	.	.	.	.	.	.	.	.	.	.	.	.	.	.	.	.	.	.
4443	1	MSH4	NM_002440	chr1	76035143	76151511	+	.	.	.	.	.	.	.	.	.	.	.	.	.	.	.	.	.	.	.	.
5363	1	ST6GALNAC3	NM_152996	chr1	76312976	76869257	+	CL528799	chr1	76338641	-	.	.	.	.	.	.	.	.	100_1_522_2	chr1	76664143	+	.	.	.	.
5365	1	ST6GALNAC5	NM_030965	chr1	77105773	77302325	+	.	.	.	.	.	.	.	.	.	.	.	.	.	.	.	.	.	.	.	.
4799	1	PIGK	NM_005482	chr1	77327254	77457720	-	.	.	.	.	.	.	.	.	.	.	.	.	.	.	.	.	.	.	.	.
355	3,5	AK5	NM_174858	chr1	77520249	77798242	+	.	.	.	.	.	.	.	.	.	.	.	.	.	.	.	.	.	.	.	.
2455	1,5	USP33	NM_015017	chr1	77934261	77998152	-	.	.	.	.	.	.	.	.	.	.	.	.	.	.	.	.	.	.	.	.
7286	0,25	DNAJB4	NM_007034	chr1	78243223	78255583	+	.	.	.	.	.	.	.	.	.	.	.	.	.	.	.	.	.	.	.	.
6463	0,5	IFI44L	NM_006820	chr1	78858675	78884418	+	.	.	.	.	.	.	.	.	.	.	.	.	.	.	.	.	.	.	.	.
3675	1	ELTD1	NM_022159	chr1	79128036	79245083	-	.	.	.	.	.	.	.	.	.	.	.	.	.	.	.	.	.	.	.	.
2232	1,5	LPHN2	NM_012302	chr1	82038669	82230695	+	.	.	.	.	.	.	.	.	_395_1_239	chr1	82209844	+	.	.	.	.	.	.	.	.
2343	1,5	PRKACB	NM_001242858	chr1	84402652	84476769	+	.	.	.	.	.	.	.	.	.	.	.	.	.	.	.	.	.	.	.	.
5357	1	SSX2IP	NM_001166295	chr1	84881977	84928739	-	.	.	.	.	.	.	.	.	.	.	.	.	.	.	.	.	.	.	.	.
1002	2,25	BCL10	NM_003921	chr1	85504047	85515175	-	.	.	.	.	.	.	.	.	.	.	.	.	.	.	.	.	.	.	.	.
6242	0,5	CYR61	NM_001554	chr1	85819031	85822236	+	.	.	.	.	.	.	.	.	.	.	.	.	.	.	.	.	.	.	.	.
7246	0,25	CLCA2	NM_006536	chr1	86662356	86694828	+	.	.	.	.	.	.	.	.	.	.	.	.	.	.	.	.	.	.	.	.

Table S2

tumor associated genes								HIV				MLV				MMTV				MMTV(SIN)				MMTV(SIN)arrest			
rank	score	gene symbol	RefSEq	chrom	txStart	txEnd	strand	integrant	chr	position	strand	integrant	chr	position	strand	integrant	chr	position	strand	integrant	chr	position	strand	integrant	chr	position	strand
6186	0,5	CLCA4	NM_012128	chr1	86785346	86819020	+	.	.	.	.	.	.	.	.	.	.	.	.	.	.	.	.	.	.	.	.
2768	1,25	SH3GLB1	NM_001206652	chr1	86942840	86986455	+	.	.	.	.	.	.	.	.	.	.	.	.	.	.	.	.	.	.	.	.
4081	1	HS2ST1	NM_001134492	chr1	87152922	87336713	+	.	.	.	.	.	.	.	.	.	.	.	.	.	.	.	.	.	.	.	.
6565	0,5	LOC339524	NR_026989	chr1	87368035	87407474	+	.	.	.	.	.	.	.	.	.	.	.	.	.	.	.	.	.	.	.	.
4294	1	LMO4	NM_006769	chr1	87566738	87587194	+	.	.	.	.	.	.	.	.	.	.	.	.	.	.	.	.	.	.	.	.
4821	1	PKN2	NM_006256	chr1	88922509	89074526	+	CL528804	chr1	89070040	-	.	.	.	.	.	.	.	.	.	.	.	.	.	.	.	.
4821	1	PKN2	NM_006256	chr1	88922509	89074526	+	BH609404	chr1	89019150	-	.	.	.	.	.	.	.	.	.	.	.	.	.	.	.	.
2154	1,5	GBP1	NM_002053	chr1	89290574	89303631	-	.	.	.	.	.	.	.	.	.	.	.	.	.	.	.	.	.	.	.	.
2155	1,5	GBP2	NM_004120	chr1	89345897	89364387	-	.	.	.	.	.	.	.	.	.	.	.	.	.	.	.	.	.	.	.	.
1303	2	CDC7	NM_003503	chr1	91738991	91763909	+	.	.	.	.	.	.	.	.	.	.	.	.	.	.	.	.	.	.	.	.
2798	1,25	TGFBR3	NM_003243	chr1	91918487	92124424	-	.	.	.	.	AY516332	chr1	92070642	+	.	.	.	.	.	.	.	.	.	.	.	.
7194	0,25	BRDT	NM_001242810	chr1	92187515	92252573	+	.	.	.	.	.	.	.	.	.	.	.	.	.	.	.	.	.	.	.	.
2125	1,5	EPHX4	NM_173567	chr1	92268120	92301681	+	.	.	.	.	.	.	.	.	.	.	.	.	.	.	.	.	.	.	.	.
6521	0,5	KIAA1107	NM_015237	chr1	92405196	92422868	+	.	.	.	.	.	.	.	.	.	.	.	.	.	.	.	.	.	.	.	.
6382	0,5	GFI1	NM_001127215	chr1	92712905	92721944	-	.	.	.	.	.	.	.	.	.	.	.	.	.	.	.	.	.	.	.	.
6828	0,5	RPL5	NM_000969	chr1	93070181	93080069	+	.	.	.	.	.	.	.	.	.	.	.	.	.	.	.	.	.	.	.	.
1053	2,25	GCLM	NM_002061	chr1	94125177	94147600	-	.	.	.	.	.	.	.	.	.	.	.	.	.	.	.	.	.	.	.	.
1191	2	ABCA4	NM_000350	chr1	94230981	94359293	-	.	.	.	.	.	.	.	.	.	.	.	.	.	.	.	.	.	.	.	.
785	2,5	ARHGAP29	NM_004815	chr1	94407050	94475895	-	CL528805	chr1	94472667	+	.	.	.	.	.	.	.	.	.	.	.	.	.	.	.	.
2857	1	ABCD3	NM_002858	chr1	94656520	94756807	+	.	.	.	.	.	.	.	.	.	.	.	.	.	.	.	.	.	.	.	.
3709	1	F3	NM_001178096	chr1	94767319	94780001	-	.	.	.	.	.	.	.	.	.	.	.	.	.	.	.	.	.	.	.	.
2958	1	ALG14	NM_144988	chr1	95220866	95311095	-	.	.	.	.	.	.	.	.	.	.	.	.	.	.	.	.	.	.	.	.
154	4,75	DPYD	NM_000110	chr1	97315887	98159203	-	CL529472	chr1	98154086	-	.	.	.	.	.	.	.	.	.	.	.	.	.	.	.	.
154	4,75	DPYD	NM_000110	chr1	97315887	98159203	-	CL528806	chr1	97374865	-	.	.	.	.	.	.	.	.	.	.	.	.	.	.	.	.
154	4,75	DPYD	NM_000110	chr1	97315887	98159203	-	CL799700	chr1	97652868	+	.	.	.	.	.	.	.	.	.	.	.	.	.	.	.	.
5933	0,75	LPPR4	NM_014839	chr1	99502435	99547726	+	.	.	.	.	.	.	.	.	.	.	.	.	.	.	.	.	.	.	.	.
2923	1	AGL	NM_000645	chr1	100099353	100162167	+	CL529759	chr1	100121150	+	.	.	.	.	.	.	.	.	.	.	.	.	.	.	.	.
2923	1	AGL	NM_000645	chr1	100099353	100162167	+	CL800188	chr1	100112481	+	.	.	.	.	.	.	.	.	.	.	.	.	.	.	.	.
3531	1	DBT	NM_001918	chr1	100425065	100487997	-	.	.	.	.	.	.	.	.	.	.	.	.	.	.	.	.	.	.	.	.
541	3	CDC14A	NM_033312	chr1	100590610	100737609	+	.	.	.	.	.	.	.	.	.	.	.	.	s1424_218	chr1	100625355	+	.	.	.	.
767	2,75	VCAM1	NM_001078	chr1	100957783	100977189	+	.	.	.	.	.	.	.	.	.	.	.	.	.	.	.	.	.	.	.	.
3706	1	EXTL2	NM_001261440	chr1	101110515	101133323	-	.	.	.	.	.	.	.	.	.	.	.	.	.	.	.	.	.	.	.	.
1737	2	S1PR1	NM_001400	chr1	101474892	101479664	+	.	.	.	.	.	.	.	.	.	.	.	.	.	.	.	.	.	.	.	.
4662	1	OLFM3	NM_058170	chr1	102040714	102235378	-	.	.	.	.	.	.	.	.	.	.	.	.	.	.	.	.	.	.	.	.
1319	2	COL11A1	NM_080630	chr1	103114610	103346640	-	CL528807	chr1	103221621	-	.	.	.	.	.	.	.	.	.	.	.	.	.	.	.	.
2499	1,25	AMY2B	NM_020978	chr1	103898844	103923672	+	.	.	.	.	.	.	.	.	.	.	.	.	.	.	.	.	.	.	.	.
2498	1,25	AMY2A	NM_000699	chr1	103961521	103969923	+	.	.	.	.	.	.	.	.	.	.	.	.	.	.	.	.	.	.	.	.
7161	0,25	AMY1B	NM_001008218	chr1	104031563	104040412	-	.	.	.	.	.	.	.	.	.	.	.	.	.	.	.	.	.	.	.	.
7162	0,25	AMY1C	NM_001008219	chr1	104093963	104102833	+	.	.	.	.	.	.	.	.	.	.	.	.	.	.	.	.	.	.	.	.
1691	2	PRMT6	NM_018137	chr1	107400789	107403439	+	.	.	.	.	.	.	.	.	.	.	.	.	.	.	.	.	.	.	.	.
4639	1	NTNG1	NM_014917	chr1	107484267	107825998	+	.	.	.	.	.	.	.	.	.	.	.	.	s1373_208	chr1	107741672	-	387_100_1	chr1	107584478	+
1978	1,75	VAV3	NM_006113	chr1	107915304	108309068	-	CL799652	chr1	108234172	-	.	.	.	.	.	.	.	.	.	.	.	.	.	.	.	.
7095	0,5	WDR47	NM_001142551	chr1	109314359	109386373	-	BH609401	chr1	109328103	-	.	.	.	.	.	.	.	.	.	.	.	.	.	.	.	.
7386	0,25	KIAA1324	NM_001267048	chr1	109458107	109550926	+	.	.	.	.	.	.	.	.	.	.	.	.	.	.	.	.	.	.	.	.
5182	1	SARS	NM_006513	chr1	109558037	109582327	+	.	.	.	.	AY516340	chr1	109562064	+	.	.	.	.	.	.	.	.	.	.	.	.
7495	0,25	PSRC1	NM_001032291	chr1	109623698	109627313	-	.	.	.	.	.	.	.	.	.	.	.	.	.	.	.	.	.	.	.	.
6400	0,5	GPR61	NM_031936	chr1	109884016	109889978	+	.	.	.	.	.	.	.	.	.	.	.	.	.	.	.	.	.	.	.	.
2163	1,5	GNAI3	NM_006496	chr1	109892708	109939977	+	.	.	.	.	.	.	.	.	.	.	.	.	.	.	.	.	.	.	.	.
2977	1	AMPD2	NM_139156	chr1	109963957	109976200	+	.	.	.	.	.	.	.	.	.	.	.	.	.	.	.	.	.	.	.	.
2609	1,25	GSTM4	NM_000850	chr1	110000220	110005854	+	.	.	.	.	.	.	.	.	.	.	.	.	.	.	.	.	.	.	.	.
852	2,5	GSTM2	NM_001142368	chr1	110012166	110028142	+	.	.	.	.	.	.	.	.	.	.	.	.	.	.	.	.	.	.	.	.

Table S2

tumor associated genes								HIV				MLV				MMTV				MMTV(SIN)				MMTV(SIN)arrest			
rank	score	gene symbol	RefSEq	chrom	txStart	txEnd	strand	integrant	chr	position	strand	integrant	chr	position	strand	integrant	chr	position	strand	integrant	chr	position	strand	integrant	chr	position	strand
1064	2,25	GSTM1	NM_000561	chr1	110031940	110037890	+	.	.	.	.	.	.	.	.	.	.	.	.	.	.	.	.	.	.	.	.
1434	2	GSTM5	NM_000851	chr1	110056386	110062414	+	.	.	.	.	.	.	.	.	.	.	.	.	.	.	.	.	.	.	.	.
1065	2,25	GSTM3	NR_024537	chr1	110078076	110085183	-	.	.	.	.	.	.	.	.	.	.	.	.	.	.	.	.	.	.	.	.
2556	1,25	CSF1	NM_172212	chr1	110254755	110270888	+	.	.	.	.	.	.	.	.	.	.	.	.	.	.	.	.	.	.	.	.
6923	0,5	SLC6A17	NM_001010898	chr1	110494654	110546346	+	.	.	.	.	.	.	.	.	.	.	.	.	.	.	.	.	2694_237_1	chr1	110522282	+
1720	2	RBM15	NM_022768	chr1	110683467	110690826	+	.	.	.	.	.	.	.	.	.	.	.	.	.	.	.	.	.	.	.	.
6490	0,5	KCNA2	NM_004974	chr1	110947298	110950498	-	.	.	.	.	.	.	.	.	.	.	.	.	.	.	.	.	.	.	.	.
6152	0,5	CD53	NM_001040033	chr1	111215343	111244081	+	.	.	.	.	.	.	.	.	.	.	.	.	.	.	.	.	.	.	.	.
5891	0,75	CHI3L2	NM_001025199	chr1	111573855	111587585	+	.	.	.	.	.	.	.	.	.	.	.	.	.	.	.	.	.	.	.	.
3353	1	CHIA	NM_001258004	chr1	111634996	111664711	+	.	.	.	.	.	.	.	.	.	.	.	.	.	.	.	.	.	.	.	.
4675	1	OVGP1	NM_002557	chr1	111758459	111771922	-	.	.	.	.	.	.	.	.	.	.	.	.	.	.	.	.	.	.	.	.
2010	1,5	ATP5F1	NM_001688	chr1	111793265	111806048	+	.	.	.	.	.	.	.	.	.	.	.	.	.	.	.	.	.	.	.	.
2744	1,25	RAP1A	NM_001010935	chr1	111963927	112057624	+	.	.	.	.	.	.	.	.	.	.	.	.	.	.	.	.	.	.	.	.
1859	2	WNT2B	NM_004185	chr1	112811562	112865433	+	.	.	.	.	.	.	.	.	.	.	.	.	.	.	.	.	.	.	.	.
7574	0,25	ST7L	NM_138727	chr1	112867663	112963563	-	.	.	.	.	.	.	.	.	.	.	.	.	.	.	.	.	.	.	.	.
7223	0,25	CAPZA1	NM_006135	chr1	112963597	113015764	+	.	.	.	.	.	.	.	.	.	.	.	.	.	.	.	.	.	.	.	.
751	2,75	RHOC	NM_001042679	chr1	113045271	113051201	-	.	.	.	.	.	.	.	.	.	.	.	.	.	.	.	.	.	.	.	.
4900	1	PPM1J	NM_005167	chr1	113054138	113059473	-	.	.	.	.	.	.	.	.	.	.	.	.	.	.	.	.	.	.	.	.
6901	0,5	SLC16A1	NM_003051	chr1	113255992	113300498	-	.	.	.	.	.	.	.	.	.	.	.	.	.	.	.	.	.	.	.	.
4332	1	MAGI3	NM_152900	chr1	113734997	114030068	+	CL528808	chr1	113775804	-	.	.	.	.	.	.	.	.	.	.	.	.	.	.	.	.
2350	1,5	PTPN22	NM_001193431	chr1	114157955	114215898	-	.	.	.	.	.	.	.	.	.	.	.	.	.	.	.	.	.	.	.	.
3538	1	DCLRE1B	NM_022836	chr1	114249437	114258231	+	.	.	.	.	.	.	.	.	.	.	.	.	.	.	.	.	.	.	.	.
4045	1	HIPK1	NM_181358	chr1	114298021	114322014	+	.	.	.	.	.	.	.	.	.	.	.	.	.	.	.	.	.	.	.	.
977	2,5	TRIM33	NM_033020	chr1	114736921	114855304	-	CL528810	chr1	114758420	-	.	.	.	.	.	.	.	.	.	.	.	.	.	.	.	.
2976	1	AMPD1	NM_000036	chr1	115017242	115039762	-	.	.	.	.	.	.	.	.	.	.	.	.	.	.	.	.	.	.	.	.
46	7,5	NRAS	NM_002524	chr1	115048607	115061038	-	.	.	.	.	.	.	.	.	.	.	.	.	.	.	.	.	.	.	.	.
5417	1	SYCP1	NM_003176	chr1	115198977	115339513	+	.	.	.	.	.	.	.	.	.	.	.	.	.	.	.	.	.	.	.	.
2811	1,25	TSHB	NM_000549	chr1	115373937	115378464	+	.	.	.	.	.	.	.	.	.	.	.	.	.	.	.	.	.	.	.	.
1944	1,75	NGF	NM_002506	chr1	115630059	115682380	-	.	.	.	.	.	.	.	.	.	.	.	.	.	.	.	.	.	.	.	.
5667	1	VANGL1	NM_001172412	chr1	115986772	116042368	+	.	.	.	.	.	.	.	.	.	.	.	.	.	.	.	.	.	.	.	.
5258	1	SLC22A15	NM_018420	chr1	116320641	116414198	+	.	.	.	.	.	.	.	.	.	.	.	.	.	.	.	.	.	.	.	.
1293	2	CD2	NM_001767	chr1	117098608	117113374	+	.	.	.	.	.	.	.	.	.	.	.	.	.	.	.	.	.	.	.	.
2825	1,25	VTCN1	NM_001253850	chr1	117487731	117555105	-	.	.	.	.	.	.	.	.	.	.	.	.	.	.	.	.	.	.	.	.
4337	1	MAN1A2	NM_006699	chr1	117711607	117869843	+	.	.	.	.	.	.	.	.	.	.	.	.	.	.	.	.	.	.	.	.
7010	0,5	TBX15	NM_152380	chr1	119227188	119333702	-	.	.	.	.	.	.	.	.	.	.	.	.	.	.	.	.	.	.	.	.
5685	1	WARS2	NM_201263	chr1	119375361	119484818	-	.	.	.	.	.	.	.	.	.	.	.	.	.	.	.	.	.	.	.	.
4012	1	HAO2	NM_016527	chr1	119712924	119738274	+	.	.	.	.	.	.	.	.	.	.	.	.	.	.	.	.	.	.	.	.
592	3	HSD3B2	NM_001166120	chr1	119759076	119767185	+	.	.	.	.	.	.	.	.	.	.	.	.	.	.	.	.	.	.	.	.
1456	2	HSD3B1	NM_000862	chr1	119851348	119859204	+	.	.	.	.	.	.	.	.	.	.	.	.	.	.	.	.	.	.	.	.
4783	1	PHGDH	NM_006623	chr1	120055941	120088372	+	.	.	.	.	.	.	.	.	.	.	.	.	.	.	.	.	.	.	.	.
4060	1	HMGCS2	NM_001166107	chr1	120092141	120113078	-	.	.	.	.	.	.	.	.	.	.	.	.	.	.	.	.	.	.	.	.
2752	1,25	REG4	NM_001159352	chr1	120138163	120155726	-	.	.	.	.	.	.	.	.	.	.	.	.	.	.	.	.	.	.	.	.
284	4	NOTCH2	NM_024408	chr1	120255698	120413840	-	.	.	.	.	.	.	.	.	.	.	.	.	.	.	.	.	.	.	.	.
3764	1	FCGR1B	NM_001004340	chr1	120727650	120737467	-	.	.	.	.	.	.	.	.	.	.	.	.	.	.	.	.	.	.	.	.
914	2,5	PDE4DIP	NM_022359	chr1	143663117	143787543	-	.	.	.	.	.	.	.	.	.	.	.	.	.	.	.	.	.	.	.	.
4596	1	NOTCH2NL	NM_203458	chr1	143920467	143997269	+	CL528814	chr1	143928973	-	.	.	.	.	.	.	.	.	.	.	.	.	.	.	.	.
4872	1	POLR3GL	NM_032305	chr1	144167592	144181744	-	.	.	.	.	.	.	.	.	.	.	.	.	.	.	.	.	.	.	.	.
6015	0,5	ANKRD34A	NM_001039888	chr1	144181864	144187004	+	.	.	.	.	.	.	.	.	.	.	.	.	.	.	.	.	.	.	.	.
6731	0,5	PEX11B	NM_001184795	chr1	144227916	144235089	+	.	.	.	.	.	.	.	.	.	.	.	.	.	.	.	.	.	.	.	.
4173	1	ITGA10	NM_003637	chr1	144236346	144255225	+	.	.	.	.	.	.	.	.	.	.	.	.	.	.	.	.	.	.	.	.
6743	0,5	PIAS3	NM_006099	chr1	144287344	144297903	+	.	.	.	.	.	.	.	.	.	.	.	.	.	.	.	.	.	.	.	.

Table S2

tumor associated genes								HIV				MLV				MMTV				MMTV(SIN)				MMTV(SIN)arrest			
rank	score	gene symbol	RefSEq	chrom	txStart	txEnd	strand	integrant	chr	position	strand	integrant	chr	position	strand	integrant	chr	position	strand	integrant	chr	position	strand	integrant	chr	position	strand
4869	1	POLR3C	NM_006468	chr1	144303961	144322241	-	.	.	.	.	.	.	.	.	.	.	.	.	.	.	.	.	.	.	.	.
3296	1	CD160	NM_007053	chr1	144407154	144426922	-	.	.	.	.	.	.	.	.	.	.	.	.	.	.	.	.	.	.	.	.
1684	2	PRKAB2	NM_005399	chr1	145093308	145110753	-	.	.	.	.	.	.	.	.	.	.	.	.	.	.	.	.	.	.	.	.
534	3	BCL9	NM_004326	chr1	145479805	145564639	+	CL800205	chr1	145541703	-	.	.	.	.	.	.	.	.	.	.	.	.	.	.	.	.
2889	1	ACP6	NM_016361	chr1	145585791	145609258	-	.	.	.	.	.	.	.	.	.	.	.	.	.	.	.	.	.	.	.	.
7346	0,25	HIST2H3C	NM_021059	chr1	148078882	148079389	-	.	.	.	.	.	.	.	.	.	.	.	.	.	.	.	.	.	.	.	.
2041	1,5	CA14	NM_012113	chr1	148496841	148504102	+	.	.	.	.	.	.	.	.	.	.	.	.	.	.	.	.	.	.	.	.
6092	0,5	C1orf54	NM_024579	chr1	148511806	148519959	+	.	.	.	.	.	.	.	.	.	.	.	.	.	.	.	.	.	.	.	.
5432	1	TARS2	NM_025150	chr1	148726543	148746373	+	.	.	.	.	.	.	.	.	.	.	.	.	.	.	.	.	.	.	.	.
1105	2,25	MCL1	NM_001197320	chr1	148813650	148818838	-	.	.	.	.	.	.	.	.	.	.	.	.	.	.	.	.	.	.	.	.
7261	0,25	CTSS	NM_001199739	chr1	148969295	149005057	-	.	.	.	.	.	.	.	.	.	.	.	.	.	.	.	.	.	.	.	.
5896	0,75	CTSK	NM_000396	chr1	149035307	149047541	-	.	.	.	.	.	.	.	.	.	.	.	.	.	.	.	.	.	.	.	.
357	3,5	ARNT	NM_001197325	chr1	149048804	149115868	-	.	.	.	.	.	.	.	.	.	.	.	.	.	.	.	.	.	.	.	.
677	3	SETDB1	NM_001145415	chr1	149165438	149203844	+	CL528815	chr1	149201875	-	.	.	.	.	.	.	.	.	.	.	.	.	.	.	.	.
4964	1	PRUNE	NM_021222	chr1	149247596	149274813	+	.	.	.	.	.	.	.	.	.	.	.	.	.	.	.	.	.	.	.	.
896	2,5	MLLT11	NM_006818	chr1	149298774	149307597	+	.	.	.	.	.	.	.	.	.	.	.	.	.	.	.	.	.	.	.	.
7089	0,5	VPS72	NM_005997	chr1	149415557	149429264	-	.	.	.	.	.	.	.	.	.	.	.	.	.	.	.	.	.	.	.	.
1627	2	PIP5K1A	NM_001135636	chr1	149437644	149488631	+	.	.	.	.	.	.	.	.	.	.	.	.	.	.	.	.	.	.	.	.
5846	1	ZNF687	NM_020832	chr1	149521414	149531005	+	.	.	.	.	.	.	.	.	.	.	.	.	.	.	.	.	.	.	.	.
639	3	PI4KB	NM_002651	chr1	149530896	149566757	-	.	.	.	.	.	.	.	.	.	.	.	.	.	.	.	.	.	.	.	.
6865	0,5	SELENBP1	NM_003944	chr1	149603401	149611834	-	.	.	.	.	.	.	.	.	.	.	.	.	.	.	.	.	.	.	.	.
5150	1	RORC	NM_005060	chr1	150045170	150070972	-	.	.	.	.	.	.	.	.	.	.	.	.	.	.	.	.	.	.	.	.
2760	1,25	S100A11	NM_005620	chr1	150271605	150276135	-	.	.	.	.	.	.	.	.	.	.	.	.	.	.	.	.	.	.	.	.
6551	0,5	LCE1D	NM_178352	chr1	151035850	151037281	+	.	.	.	.	.	.	.	.	.	.	.	.	.	.	.	.	.	.	.	.
2631	1,25	IVL	NM_005547	chr1	151147662	151150986	+	.	.	.	.	.	.	.	.	.	.	.	.	.	.	.	.	.	.	.	.
7571	0,25	SPRR1B	NM_003125	chr1	151270302	151272000	+	.	.	.	.	.	.	.	.	.	.	.	.	.	.	.	.	.	.	.	.
6966	0,5	SPRR2G	NM_001014291	chr1	151388681	151390051	-	.	.	.	.	.	.	.	.	.	.	.	.	.	.	.	.	.	.	.	.
7401	0,25	LOR	NM_000427	chr1	151498802	151501224	+	.	.	.	.	.	.	.	.	.	.	.	.	.	.	.	.	.	.	.	.
5957	0,75	S100A9	NM_002965	chr1	151596953	151600127	+	.	.	.	.	.	.	.	.	.	.	.	.	.	.	.	.	.	.	.	.
1961	1,75	S100A8	NM_002964	chr1	151629131	151630288	-	.	.	.	.	.	.	.	.	.	.	.	.	.	.	.	.	.	.	.	.
7525	0,25	S100A6	NM_014624	chr1	151773699	151775341	-	.	.	.	.	.	.	.	.	.	.	.	.	.	.	.	.	.	.	.	.
5956	0,75	S100A4	NM_002961	chr1	151782721	151784906	-	.	.	.	.	.	.	.	.	.	.	.	.	.	.	.	.	.	.	.	.
6843	0,5	S100A2	NM_005978	chr1	151800208	151804930	-	.	.	.	.	.	.	.	.	.	.	.	.	.	.	.	.	.	.	.	.
7524	0,25	S100A14	NM_020672	chr1	151853355	151855432	-	.	.	.	.	.	.	.	.	.	.	.	.	.	.	.	.	.	.	.	.
5175	1	S100A13	NM_001024213	chr1	151857899	151866148	-	.	.	.	.	.	.	.	.	.	.	.	.	.	.	.	.	.	.	.	.
6842	0,5	S100A1	NM_006271	chr1	151867496	151871137	+	.	.	.	.	.	.	.	.	.	.	.	.	.	.	.	.	.	.	.	.
1581	2	NPR1	NM_000906	chr1	151917787	151933092	+	.	.	.	.	.	.	.	.	.	.	.	.	.	.	.	.	.	.	.	.
2357	1,5	RAB13	NM_002870	chr1	152220751	152225430	-	.	.	.	.	.	.	.	.	.	.	.	.	.	.	.	.	.	.	.	.
198	4,5	TPM3	NM_001043353	chr1	152394403	152422349	-	.	.	.	.	.	.	.	.	.	.	.	.	.	.	.	.	.	.	.	.
1212	2	ADAR	NM_015841	chr1	152821157	152847348	-	CL799830	chr1	152858723	+	.	.	.	.	.	.	.	.	.	.	.	.	.	.	.	.
4855	1	PMVK	NM_006556	chr1	153163831	153176108	-	.	.	.	.	.	.	.	.	.	.	.	.	.	.	.	.	.	.	.	.
123	5,25	SHC1	NM_003029	chr1	153201397	153213583	-	.	.	.	.	.	.	.	.	.	.	.	.	.	.	.	.	.	.	.	.
3376	1	CKS1B	NM_001826	chr1	153213741	153218349	+	.	.	.	.	.	.	.	.	.	.	.	.	.	.	.	.	.	.	.	.
3781	1	FLAD1	NM_025207	chr1	153222393	153232211	+	.	.	.	.	.	.	.	.	.	.	.	.	.	.	.	.	.	.	.	.
707	2,75	ADAM15	NM_207197	chr1	153290371	153301876	+	.	.	.	.	.	.	.	.	.	.	.	.	.	.	.	.	.	.	.	.
3654	1	EFNA4	NM_182689	chr1	153302836	153308653	+	.	.	.	.	.	.	.	.	.	.	.	.	.	.	.	.	.	.	.	.
2576	1,25	EFNA3	NM_004952	chr1	153317971	153326638	+	.	.	.	.	.	.	.	.	.	.	.	.	.	.	.	.	.	.	.	.
281	4	MUC1	NM_001204291	chr1	153424923	153429330	-	.	.	.	.	.	.	.	.	.	.	.	.	.	.	.	.	.	.	.	.
1807	2	THBS3	NM_007112	chr1	153432002	153444396	-	.	.	.	.	.	.	.	.	.	.	.	.	.	.	.	.	.	.	.	.
3860	1	GBA	NM_001171811	chr1	153470862	153481277	-	.	.	.	.	.	.	.	.	.	.	.	.	.	.	.	.	.	.	.	.
3390	1	CLK2	NM_003993	chr1	153499282	153509905	-	.	.	.	.	.	.	.	.	.	.	.	.	.	.	.	.	.	.	.	.

Table S2

tumor associated genes								HIV				MLV				MMTV				MMTV(SIN)				MMTV(SIN)arrest			
rank	score	gene symbol	RefSEq	chrom	txStart	txEnd	strand	integrant	chr	position	strand	integrant	chr	position	strand	integrant	chr	position	strand	integrant	chr	position	strand	integrant	chr	position	strand
1630	2	PKLR	NM_181871	chr1	153525707	153537416	-	.	.	.	.	.	.	.	.	.	.	.	.	.	.	.	.	.	.	.	.
1402	2	FDPS	NM_001242825	chr1	153545162	153557081	+	.	.	.	.	.	.	.	.	.	.	.	.	.	.	.	.	.	.	.	.
3055	1	ASH1L	NM_018489	chr1	153571675	153798948	-	CL799896	chr1	153683517	-	.	.	.	.	.	.	.	.	.	.	.	.	.	.	.	.
3055	1	ASH1L	NM_018489	chr1	153571675	153798948	-	CL799773	chr1	153604173	-	.	.	.	.	.	.	.	.	.	.	.	.	.	.	.	.
3055	1	ASH1L	NM_018489	chr1	153571675	153798948	-	CL799934	chr1	153672247	-	.	.	.	.	.	.	.	.	.	.	.	.	.	.	.	.
3055	1	ASH1L	NM_018489	chr1	153571675	153798948	-	CL800524	chr1	153612925	+	.	.	.	.	.	.	.	.	.	.	.	.	.	.	.	.
3055	1	ASH1L	NM_018489	chr1	153571675	153798948	-	AY517389.1	chr1	153611094	-	.	.	.	.	.	.	.	.	.	.	.	.	.	.	.	.
3055	1	ASH1L	NM_018489	chr1	153571675	153798948	-	CL799732	chr1	153718925	-	.	.	.	.	.	.	.	.	.	.	.	.	.	.	.	.
6822	0,5	RIT1	NM_001256821	chr1	154134222	154147330	-	.	.	.	.	.	.	.	.	.	.	.	.	.	.	.	.	.	.	.	.
7076	0,5	UBQLN4	NM_020131	chr1	154271715	154290140	-	.	.	.	.	.	.	.	.	.	.	.	.	.	.	.	.	.	.	.	.
4291	1	LMNA	NM_001257374	chr1	154362574	154376504	+	.	.	.	.	.	.	.	.	.	.	.	.	.	.	.	.	.	.	.	.
2512	1,25	BGLAP	NM_199173	chr1	154478574	154479747	+	.	.	.	.	.	.	.	.	.	.	.	.	.	.	.	.	.	.	.	.
6715	0,5	PAQR6	NM_024897	chr1	154479830	154484467	-	.	.	.	.	.	.	.	.	.	.	.	.	.	.	.	.	.	.	.	.
2056	1,5	CCT3	NM_005998	chr1	154545375	154574830	-	.	.	.	.	.	.	.	.	.	.	.	.	.	.	.	.	.	.	.	.
6093	0,5	C1orf61	NM_006365	chr1	154640678	154665808	-	.	.	.	.	.	.	.	.	.	.	.	.	.	.	.	.	.	.	.	.
2510	1,25	BCAN	NM_198427	chr1	154878363	154889888	+	.	.	.	.	.	.	.	.	.	.	.	.	.	.	.	.	.	.	.	.
2693	1,25	NES	NM_006617	chr1	154905179	154913813	-	.	.	.	.	.	.	.	.	.	.	.	.	.	.	.	.	.	.	.	.
7341	0,25	HDGF	NM_001126050	chr1	154978522	154988864	-	.	.	.	.	.	.	.	.	.	.	.	.	.	.	.	.	.	.	.	.
1679	2	PRCC	NM_005973	chr1	155003897	155037233	+	.	.	.	.	.	.	.	.	.	.	.	.	.	.	.	.	.	.	.	.
50	7,25	NTRK1	NM_001007792	chr1	155052165	155118266	+	.	.	.	.	.	.	.	.	.	.	.	.	.	.	.	.	.	.	.	.
1082	2,25	INSRR	NM_014215	chr1	155077288	155095336	-	.	.	.	.	.	.	.	.	.	.	.	.	.	.	.	.	.	.	.	.
3766	1	FCRL5	NM_031281	chr1	155749790	155788934	-	.	.	.	.	.	.	.	.	.	.	.	.	.	.	.	.	.	.	.	.
1401	2	FCRL4	NM_031282	chr1	155810162	155834494	-	.	.	.	.	.	.	.	.	.	.	.	.	.	.	.	.	.	.	.	.
6535	0,5	KIRREL	NM_018240	chr1	156229686	156332468	+	.	.	.	.	.	.	.	.	.	.	.	.	.	.	.	.	.	.	.	.
7233	0,25	CD1A	NM_001763	chr1	156490550	156494682	+	.	.	.	.	.	.	.	.	.	.	.	.	.	.	.	.	.	.	.	.
7235	0,25	CD1C	NM_001765	chr1	156526186	156531188	+	.	.	.	.	.	.	.	.	.	.	.	.	.	.	.	.	.	.	.	.
7234	0,25	CD1B	NM_001764	chr1	156564363	156567945	-	.	.	.	.	.	.	.	.	.	.	.	.	.	.	.	.	.	.	.	.
4419	1	MNDA	NM_002432	chr1	157067791	157085894	+	.	.	.	.	.	.	.	.	.	.	.	.	.	.	.	.	.	.	.	.
2936	1	AIM2	NM_004833	chr1	157298898	157313271	-	.	.	.	.	.	.	.	.	.	.	.	.	.	.	.	.	.	.	.	.
3626	1	DUSP23	NM_017823	chr1	158017382	158018957	+	.	.	.	.	.	.	.	.	.	.	.	.	.	.	.	.	.	.	.	.
6895	0,5	SLAMF8	NM_020125	chr1	158063102	158073906	+	.	.	.	.	.	.	.	.	.	.	.	.	.	.	.	.	.	.	.	.
1620	2	PIGM	NM_145167	chr1	158264085	158268407	-	.	.	.	.	.	.	.	.	.	.	.	.	.	.	.	.	.	.	.	.
6500	0,5	KCNJ10	NM_002241	chr1	158273880	158306675	-	.	.	.	.	.	.	.	.	.	.	.	.	.	.	.	.	.	.	.	.
2009	1,5	ATP1A2	NM_000702	chr1	158352143	158379998	+	.	.	.	.	.	.	.	.	.	.	.	.	.	.	.	.	.	.	.	.
6133	0,5	CASQ1	NM_001231	chr1	158426908	158438300	+	.	.	.	.	.	.	.	.	.	.	.	.	.	.	.	.	.	.	.	.
6732	0,5	PEX19	NM_001193644	chr1	158513222	158521565	-	.	.	.	.	AY516184	chr1	158521088	-	.	.	.	.	.	.	.	.	.	.	.	.
6151	0,5	CD48	NM_001256030	chr1	158916835	158948265	-	.	.	.	.	.	.	.	.	.	.	.	.	.	.	.	.	.	.	.	.
4180	1	ITLN1	NM_017625	chr1	159112953	159121584	-	.	.	.	.	.	.	.	.	.	.	.	.	.	.	.	.	.	.	.	.
6325	0,5	F11R	NM_016946	chr1	159231624	159257757	-	.	.	.	.	.	.	.	.	.	.	.	.	.	.	.	.	.	.	.	.
7080	0,5	USF1	NM_207005	chr1	159275664	159282381	-	.	.	.	.	.	.	.	.	.	.	.	.	.	.	.	.	.	.	.	.
5619	1	UFC1	NM_016406	chr1	159390157	159395270	+	.	.	.	.	.	.	.	.	.	.	.	.	.	.	.	.	.	.	.	.
4904	1	PPOX	NM_000309	chr1	159402804	159407634	+	.	.	.	.	.	.	.	.	.	.	.	.	.	.	.	.	.	.	.	.
3119	1	B4GALT3	NM_003779	chr1	159407723	159414382	-	.	.	.	.	.	.	.	.	.	.	.	.	.	.	.	.	.	.	.	.
4547	1	NDUFS2	NM_004550	chr1	159435728	159450808	+	.	.	.	.	.	.	.	.	.	.	.	.	.	.	.	.	.	.	.	.
6352	0,5	FCER1G	NM_004106	chr1	159451710	159455662	+	.	.	.	.	.	.	.	.	.	.	.	.	.	.	.	.	.	.	.	.
3003	1	APOA2	NM_001643	chr1	159458706	159460042	-	.	.	.	.	.	.	.	.	.	.	.	.	.	.	.	.	.	.	.	.
4609	1	NR1I3	NM_001077482	chr1	159466079	159474624	-	.	.	.	.	.	.	.	.	.	.	.	.	.	.	.	.	.	.	.	.
4436	1	MPZ	NM_000530	chr1	159541148	159546386	-	.	.	.	.	.	.	.	.	.	.	.	.	.	.	.	.	.	.	.	.
303	4	SDHC	NM_001035512	chr1	159550789	159601159	+	CL799887	chr1	159582743	+	.	.	.	.	.	.	.	.	.	.	.	.	.	.	.	.
6353	0,5	FCGR2A	NM_001136219	chr1	159741828	159755984	+	.	.	.	.	.	.	.	.	.	.	.	.	.	.	.	.	.	.	.	.
3765	1	FCGR3A	NM_000569	chr1	159778174	159786442	-	.	.	.	.	.	.	.	.	.	.	.	.	.	.	.	.	.	.	.	.

Table S2

tumor associated genes								HIV				MLV				MMTV				MMTV(SIN)				MMTV(SIN)arrest			
rank	score	gene symbol	RefSEq	chrom	txStart	txEnd	strand	integrant	chr	position	strand	integrant	chr	position	strand	integrant	chr	position	strand	integrant	chr	position	strand	integrant	chr	position	strand
573	3	FCGR2B	NM_001002273	chr1	159899528	159915068	+	.	.	.	.	.	.	.	.	.	.	.	.	.	.	.	.	.	.	.	.
1369	2	DUSP12	NM_007240	chr1	159986204	159993576	+	.	.	.	.	.	.	.	.	.	.	.	.	.	.	.	.	.	.	.	.
5230	1	SH2D1B	NM_053282	chr1	160631679	160648552	-	.	.	.	.	.	.	.	.	.	.	.	.	.	.	.	.	.	.	.	.
5637	1	UHMK1	NM_144624	chr1	160734218	160766043	+	.	.	.	.	.	.	.	.	.	.	.	.	.	.	.	.	.	.	.	.
7073	0,5	UAP1	NM_003115	chr1	160797919	160836257	+	.	.	.	.	.	.	.	.	.	.	.	.	.	.	.	.	.	.	.	.
559	3	DDR2	NM_006182	chr1	160868851	161016871	+	CL528818	chr1	160916391	+	.	.	.	.	.	.	.	.	1250_176	chr1	161011594	-	.	.	.	.
4091	1	HSD17B7	NM_016371	chr1	161027119	161049232	+	.	.	.	.	.	.	.	.	.	.	.	.	.	.	.	.	.	.	.	.
2375	1,5	RGS4	NM_005613	chr1	161305387	161313216	+	.	.	.	.	.	.	.	.	.	.	.	.	.	.	.	.	.	.	.	.
6815	0,5	RGS5	NM_001254748	chr1	161378712	161558205	-	.	.	.	.	.	.	.	.	.	.	.	.	.	.	.	.	.	.	.	.
1948	1,75	NUF2	NM_031423	chr1	161558346	161592177	+	.	.	.	.	.	.	.	.	.	.	.	.	.	.	.	.	.	.	.	.
1598	2	PBX1	NM_001204961	chr1	162795220	163087684	+	.	.	.	.	AY516655	chr1	162983636	+	.	.	.	.	.	.	.	.	.	.	.	.
1505	2	LMX1A	NM_001174069	chr1	163437727	163592102	-	.	.	.	.	.	.	.	.	_208_1_165	chr1	163464748	-	.	.	.	.	.	.	.	.
5173	1	RXRG	NM_006917	chr1	163636782	163681216	-	.	.	.	.	.	.	.	.	.	.	.	.	.	.	.	.	.	.	.	.
2256	1,5	MGST3	NM_004528	chr1	163866733	163891996	+	.	.	.	.	.	.	.	.	.	.	.	.	.	.	.	.	.	.	.	.
2953	1	ALDH9A1	NM_000696	chr1	163898072	163934524	-	.	.	.	.	.	.	.	.	.	.	.	.	.	.	.	.	.	.	.	.
5617	1	UCK2	NM_012474	chr1	164063355	164147479	+	.	.	.	.	.	.	.	.	.	.	.	.	.	.	.	.	.	.	.	.
4326	1	MAEL	NM_032858	chr1	165225142	165258071	+	.	.	.	.	.	.	.	.	.	.	.	.	.	.	.	.	.	.	.	.
3627	1	DUSP27	NM_001080426	chr1	165330710	165365026	+	.	.	.	.	.	.	.	.	.	.	.	.	.	.	.	.	.	.	.	.
6763	0,5	POU2F1	NM_001198786	chr1	165456689	165663206	+	CL528819	chr1	165548234	-	.	.	.	.	.	.	.	.	.	.	.	.	.	.	.	.
3298	1	CD247	NM_198053	chr1	165666500	165754471	-	.	.	.	.	.	.	.	.	.	.	.	.	.	.	.	.	.	.	.	.
5083	1	RCSD1	NM_052862	chr1	165866097	165942110	+	.	.	.	.	.	.	.	.	.	.	.	.	.	.	.	.	.	.	.	.
7435	0,25	MPZL1	NM_003953	chr1	165957810	166027780	+	.	.	.	.	.	.	.	.	.	.	.	.	.	.	.	.	.	.	.	.
1215	2	ADCY10	NM_018417	chr1	166045248	166150077	-	.	.	.	.	.	.	.	.	.	.	.	.	.	.	.	.	.	.	.	.
6393	0,5	GPR161	NM_001267609	chr1	166315403	166373529	-	.	.	.	.	.	.	.	.	.	.	.	.	.	.	.	.	.	.	.	.
6881	0,5	SFT2D2	NM_199344	chr1	166461878	166478712	+	.	.	.	.	.	.	.	.	.	.	.	.	.	.	.	.	.	.	.	.
5880	0,75	ATP1B1	NM_001677	chr1	167342570	167368584	+	.	.	.	.	.	.	.	.	.	.	.	.	.	.	.	.	.	.	.	.
2283	1,5	NME7	NM_013330	chr1	167368392	167603810	-	.	.	.	.	AY516014	chr1	167455227	+	_345_1_213	chr1	167584509	-	.	.	.	.	.	.	.	.
1146	2,25	PRRX1	NM_006902	chr1	168899936	168975165	+	.	.	.	.	.	.	.	.	.	.	.	.	.	.	.	.	.	.	.	.
7314	0,25	FMO1	NM_002021	chr1	169484286	169521737	+	.	.	.	.	.	.	.	.	.	.	.	.	.	.	.	.	.	.	.	.
3789	1	FMO4	NM_002022	chr1	169550109	169577847	+	.	.	.	.	.	.	.	.	.	.	.	.	.	.	.	.	.	.	.	.
6642	0,5	MYOC	NM_000261	chr1	169871179	169888396	-	.	.	.	.	.	.	.	.	.	.	.	.	.	.	.	.	.	.	.	.
721	2,75	DNM3	NM_001136127	chr1	170077243	170648480	+	.	.	.	.	AY516578	chr1	170585058	-	.	.	.	.	.	.	.	.	2856_256_1	chr1	170376556	+
1618	2	PIGC	NM_153747	chr1	170677219	170679853	-	.	.	.	.	.	.	.	.	.	.	.	.	.	.	.	.	.	.	.	.
726	2,75	FASLG	NM_000639	chr1	170894807	170902635	+	.	.	.	.	.	.	.	.	.	.	.	.	.	.	.	.	.	.	.	.
5512	1	TNFSF18	NM_005092	chr1	171276982	171286726	-	.	.	.	.	.	.	.	.	.	.	.	.	.	.	.	.	.	.	.	.
2803	1,25	TNFSF4	NM_003326	chr1	171419492	171443094	-	.	.	.	.	.	.	.	.	.	.	.	.	.	.	.	.	.	.	.	.
4935	1	PRDX6	NM_004905	chr1	171713108	171724569	+	.	.	.	.	.	.	.	.	.	.	.	.	.	.	.	.	.	.	.	.
3333	1	CENPL	NM_001171182	chr1	172035310	172059893	-	.	.	.	.	AY516703	chr1	172059452	-	.	.	.	.	.	.	.	.	.	.	.	.
3530	1	DARS2	NM_018122	chr1	172060419	172094305	+	.	.	.	.	.	.	.	.	.	.	.	.	.	.	.	.	.	.	.	.
6790	0,5	RABGAP1L	NM_014857	chr1	172395174	173193950	+	.	.	.	.	AY516295	chr1	173200063	+	.	.	.	.	.	.	.	.	.	.	.	.
5516	1	TNN	NM_022093	chr1	173303616	173383825	+	.	.	.	.	.	.	.	.	.	.	.	.	.	.	.	.	.	.	.	.
5519	1	TNR	NM_003285	chr1	173558557	173979375	-	.	.	.	.	.	.	.	.	.	.	.	.	.	.	.	.	.	.	.	.
5108	1	RFWD2	NM_022457	chr1	174180589	174442993	-	CL800521	chr1	174264982	+	.	.	.	.	.	.	.	.	.	.	.	.	.	.	.	.
4697	1	PAPPA2	NM_021936	chr1	174698929	174926964	+	.	.	.	.	.	.	.	.	.	.	.	.	.	.	.	.	.	.	.	.
1261	2	ASTN1	NM_207108	chr1	175104576	175400647	-	.	.	.	.	.	.	.	.	.	.	.	.	.	.	.	.	.	.	.	.
6340	0,5	FAM5B	NM_021165	chr1	175407255	175518181	+	.	.	.	.	.	.	.	.	.	.	.	.	.	.	.	.	.	.	.	.
5066	1	RASAL2	NM_170692	chr1	176329486	176715271	+	.	.	.	.	.	.	.	.	.	.	.	.	.	.	.	.	.	.	.	.
6793	0,5	RALGPS2	NM_152663	chr1	176960922	177155860	+	CL800432	chr1	177122241	-	.	.	.	.	.	.	.	.	.	.	.	.	.	.	.	.
2982	1	ANGPTL1	NM_004673	chr1	177085292	177106838	-	CL800658	chr1	177103776	-	.	.	.	.	.	.	.	.	.	.	.	.	.	.	.	.
518	3	ABL2	NM_001136000	chr1	177335084	177378847	-	.	.	.	.	.	.	.	.	.	.	.	.	.	.	.	.	.	.	.	.
2781	1,25	SOAT1	NR_045530	chr1	177529471	177594437	+	.	.	.	.	.	.	.	.	.	.	.	.	.	.	.	.	.	.	.	.

Table S2

tumor associated genes								HIV				MLV				MMTV				MMTV(SIN)				MMTV(SIN)arrest			
rank	score	gene symbol	RefSEq	chrom	txStart	txEnd	strand	integrant	chr	position	strand	integrant	chr	position	strand	integrant	chr	position	strand	integrant	chr	position	strand	integrant	chr	position	strand
3261	1	CACNA1E	NM_001205293	chr1	179719308	180042543	+	.	.	.	.	.	.	.	.	.	.	.	.	.	.	.	.	.	.	.	.
2159	1,5	GLUL	NM_002065	chr1	180617461	180627964	-	.	.	.	.	.	.	.	.	.	.	.	.	.	.	.	.	.	.	.	.
5898	0,75	DHX9	NR_033302	chr1	181075061	181123740	+	.	.	.	.	.	.	.	.	.	.	.	.	.	.	.	.	48_69_2	chr1	181104524	+
2229	1,5	LAMC1	NM_002293	chr1	181259217	181381350	+	.	.	.	.	.	.	.	.	.	.	.	.	.	.	.	.	.	.	.	.
7392	0,25	LAMC2	NM_005562	chr1	181421796	181480885	+	.	.	.	.	.	.	.	.	.	.	.	.	.	.	.	.	.	.	.	.
1579	2	NMNAT2	NM_015039	chr1	181483994	181654257	-	.	.	.	.	.	.	.	.	.	.	.	.	.	.	.	.	.	.	.	.
4508	1	NCF2	NM_001190789	chr1	181791319	181826362	-	.	.	.	.	.	.	.	.	.	.	.	.	.	.	.	.	.	.	.	.
5111	1	RGL1	NM_015149	chr1	181871830	182164289	+	.	.	.	.	.	.	.	.	.	.	.	.	.	.	.	.	.	.	.	.
3010	1	APOBEC4	NM_203454	chr1	181882033	181889071	-	CL528823	chr1	181884517	-	.	.	.	.	.	.	.	.	.	.	.	.	.	.	.	.
6332	0,5	FAM129A	NM_052966	chr1	183026788	183210305	-	.	.	.	.	.	.	.	.	.	.	.	.	.	.	.	.	.	.	.	.
5139	1	RNF2	NM_007212	chr1	183281173	183338363	+	.	.	.	.	.	.	.	.	.	.	.	.	.	.	.	.	.	.	.	.
148	5	TPR	NM_003292	chr1	184547408	184611080	-	AY516976.1	chr1	184564527	+	.	.	.	.	.	.	.	.	.	.	.	.	.	.	.	.
148	5	TPR	NM_003292	chr1	184547408	184611080	-	CL800422	chr1	184575970	-	.	.	.	.	.	.	.	.	.	.	.	.	.	.	.	.
122	5,25	PTGS2	NM_000963	chr1	184907566	184916182	-	.	.	.	.	.	.	.	.	.	.	.	.	.	.	.	.	.	.	.	.
1631	2	PLA2G4A	NM_024420	chr1	185064654	185224736	+	CL528825	chr1	185109217	+	.	.	.	.	.	.	.	.	.	.	.	.	.	.	.	.
1631	2	PLA2G4A	NM_024420	chr1	185064654	185224736	+	CL528826	chr1	185199525	-	.	.	.	.	.	.	.	.	.	.	.	.	.	.	.	.
1631	2	PLA2G4A	NM_024420	chr1	185064654	185224736	+	AY516927.1	chr1	185114218	-	.	.	.	.	.	.	.	.	.	.	.	.	.	.	.	.
2132	1,5	FAM5C	NM_199051	chr1	188333419	188713382	-	.	.	.	.	AY515864	chr1	188357150	+	.	.	.	.	1731_104	chr1	188596768	+	2967_270_5	chr1	188362110	+
6812	0,5	RGS1	NM_002922	chr1	190811479	190815782	+	CL529671	chr1	190813403	+	.	.	.	.	.	.	.	.	.	.	.	.	.	.	.	.
5615	1	UCHL5	NR_037607	chr1	191255639	191295860	-	.	.	.	.	.	.	.	.	.	.	.	.	.	.	.	.	.	.	.	.
241	4	CDC73	NM_024529	chr1	191357710	191490565	+	.	.	.	.	.	.	.	.	.	.	.	.	.	.	.	.	.	.	.	.
3106	1	B3GALT2	NM_003783	chr1	191414482	191422366	-	.	.	.	.	.	.	.	.	.	.	.	.	.	.	.	.	.	.	.	.
1894	1,75	CFH	NM_001014975	chr1	194887630	194937318	+	CL528828	chr1	194979852	+	.	.	.	.	.	.	.	.	.	.	.	.	.	.	.	.
2008	1,5	ASPM	NM_001206846	chr1	195319879	195382447	-	.	.	.	.	.	.	.	.	.	.	.	.	.	.	.	.	.	.	.	.
6214	0,5	CRB1	NM_001257965	chr1	195437214	195714208	+	.	.	.	.	.	.	.	.	.	.	.	.	.	.	.	.	.	.	.	.
4563	1	NEK7	NM_133494	chr1	196392730	196558171	+	AY516977.1	chr1	196514203	+	.	.	.	.	.	.	.	.	.	.	.	.	.	.	.	.
3094	1	ATP6V1G3	NM_133262	chr1	196758974	196776698	-	.	.	.	.	.	.	.	.	.	.	.	.	.	.	.	.	.	.	.	.
342	3,75	PTPRC	NR_052021	chr1	196874720	196928957	+	.	.	.	.	.	.	.	.	.	.	.	.	.	.	.	.	.	.	.	.
4616	1	NR5A2	NM_205860	chr1	198263392	198413173	+	.	.	.	.	.	.	.	.	.	.	.	.	.	.	.	.	.	.	.	.
2632	1,25	KIF14	NM_014875	chr1	198787247	198856485	-	AY517078.1	chr1	198838883	-	.	.	.	.	.	.	.	.	.	.	.	.	.	.	.	.
6089	0,5	C1orf106	NM_001142569	chr1	199130571	199151487	+	.	.	.	.	.	.	.	.	.	.	.	.	.	.	.	.	.	.	.	.
6531	0,5	KIF21B	NM_001252103	chr1	199205136	199259451	-	.	.	.	.	.	.	.	.	.	.	.	.	.	.	.	.	.	.	.	.
3262	1	CACNA1S	NM_000069	chr1	199275262	199348317	-	.	.	.	.	.	.	.	.	.	.	.	.	.	.	.	.	.	.	.	.
7040	0,5	TNNT2	NM_000364	chr1	199594764	199613428	-	.	.	.	.	.	.	.	.	.	.	.	.	.	.	.	.	.	.	.	.
6225	0,5	CSRP1	NM_001193570	chr1	199719280	199743010	-	.	.	.	.	.	.	.	.	.	.	.	.	.	.	.	.	.	.	.	.
2206	1,5	IPO9	NM_018085	chr1	200064910	200120045	+	.	.	.	.	.	.	.	.	.	.	.	.	.	.	.	.	.	.	.	.
7300	0,25	ELF3	NM_001114309	chr1	200246312	200252938	+	.	.	.	.	.	.	.	.	.	.	.	.	.	.	.	.	.	.	.	.
3038	1	ARL8A	NM_001256129	chr1	200369154	200380494	-	.	.	.	.	.	.	.	.	.	.	.	.	.	.	.	.	.	.	.	.
5002	1	PTPN7	NR_037664	chr1	200382763	200397339	-	.	.	.	.	.	.	.	.	.	.	.	.	.	.	.	.	.	.	.	.
4278	1	LGR6	NM_021636	chr1	200439527	200555512	+	.	.	.	.	.	.	.	.	.	.	.	.	.	.	.	.	.	.	.	.
4909	1	PPP1R12B	NM_032104	chr1	200698493	200824320	+	CL528829	chr1	200732742	-	.	.	.	.	.	.	.	.	.	.	.	.	.	.	.	.
1489	2	KDM5B	NM_006618	chr1	200963154	201044172	-	.	.	.	.	.	.	.	.	.	.	.	.	.	.	.	.	.	.	.	.
6537	0,5	KLHL12	NM_021633	chr1	201126852	201162994	-	.	.	.	.	.	.	.	.	.	.	.	.	.	.	.	.	.	.	.	.
3499	1	CYB5R1	NM_016243	chr1	201197624	201203027	-	.	.	.	.	.	.	.	.	.	.	.	.	.	.	.	.	.	.	.	.
2331	1,5	PPFIA4	NM_015053	chr1	201286933	201314487	+	.	.	.	.	.	.	.	.	.	.	.	.	.	.	.	.	.	.	.	.
1895	1,75	CHI3L1	NM_001276	chr1	201414681	201422545	-	.	.	.	.	.	.	.	.	.	.	.	.	.	.	.	.	.	.	.	.
3354	1	CHIT1	NR_045785	chr1	201451829	201465483	-	.	.	.	.	.	.	.	.	.	.	.	.	.	.	.	.	.	.	.	.
6071	0,5	BTG2	NM_006763	chr1	201541286	201545352	+	.	.	.	.	.	.	.	.	.	.	.	.	.	.	.	.	.	.	.	.
2143	1,5	FMOD	NM_002023	chr1	201576374	201586912	-	.	.	.	.	.	.	.	.	.	.	.	.	.	.	.	.	.	.	.	.
2723	1,25	PRELP	NM_201348	chr1	201711505	201727102	+	.	.	.	.	.	.	.	.	.	.	.	.	.	.	.	.	.	.	.	.
7457	0,25	OPTC	NM_014359	chr1	201729893	201744700	+	.	.	.	.	.	.	.	.	.	.	.	.	.	.	.	.	.	.	.	.

Table S2

tumor associated genes								HIV				MLV				MMTV				MMTV(SIN)				MMTV(SIN)arrest			
rank	score	gene symbol	RefSEq	chrom	txStart	txEnd	strand	integrant	chr	position	strand	integrant	chr	position	strand	integrant	chr	position	strand	integrant	chr	position	strand	integrant	chr	position	strand
6044	0,5	ATP2B4	NM_001684	chr1	201862537	201979832	+	CL800431	chr1	201868929	+	.	.	.	.	.	.	.	.	.	.	.	.	.	.	.	.
7562	0,25	SNORA77	NR_003019	chr1	201965331	201965456	+	.	.	.	.	.	.	.	.	.	.	.	.	.	.	.	.	.	.	.	.
6550	0,5	LAX1	NM_017773	chr1	202000906	202012103	+	.	.	.	.	.	.	.	.	.	.	.	.	.	.	.	.	.	.	.	.
2467	1,5	ZC3H11A	NM_014827	chr1	202031373	202089879	+	.	.	.	.	.	.	.	.	.	.	.	.	.	.	.	.	1746_163_1	chr1	202086549	-
2779	1,25	SNRPE	NM_003094	chr1	202097362	202106903	+	.	.	.	.	.	.	.	.	.	.	.	.	.	.	.	.	.	.	.	.
7564	0,25	SOX13	NM_005686	chr1	202308868	202363494	+	.	.	.	.	.	.	.	.	.	.	.	.	.	.	.	.	.	.	.	.
828	2,5	ETNK2	NM_018208	chr1	202366812	202387930	-	.	.	.	.	.	.	.	.	.	.	.	.	.	.	.	.	.	.	.	.
7511	0,25	REN	NM_000537	chr1	202390566	202402088	-	.	.	.	.	.	.	.	.	.	.	.	.	.	.	.	.	.	.	.	.
7388	0,25	KISS1	NM_002256	chr1	202426091	202432242	-	.	.	.	.	.	.	.	.	.	.	.	.	.	.	.	.	.	.	.	.
7332	0,25	GOLT1A	NM_198447	chr1	202433910	202449843	-	.	.	.	.	.	.	.	.	.	.	.	.	.	.	.	.	.	.	.	.
5945	0,75	PLEKHA6	NM_014935	chr1	202454601	202595680	-	.	.	.	.	.	.	.	.	.	.	.	.	.	.	.	.	.	.	.	.
1139	2,25	PPP1R15B	NM_032833	chr1	202639114	202647567	-	.	.	.	.	.	.	.	.	.	.	.	.	.	.	.	.	.	.	.	.
412	3,5	PIK3C2B	NM_002646	chr1	202658380	202726097	-	.	.	.	.	.	.	.	.	.	.	.	.	.	.	.	.	.	.	.	.
66	6,5	MDM4	NR_024171	chr1	202752129	202793871	+	.	.	.	.	.	.	.	.	.	.	.	.	.	.	.	.	.	.	.	.
6579	0,5	LRRN2	NM_006338	chr1	202852925	202921104	-	.	.	.	.	.	.	.	.	.	.	.	.	.	.	.	.	.	.	.	.
4572	1	NFASC	NM_001005388	chr1	203064404	203258573	+	.	.	.	.	.	.	.	.	.	.	.	.	.	.	.	.	.	.	.	.
6197	0,5	CNTN2	NM_005076	chr1	203278962	203313794	+	.	.	.	.	.	.	.	.	.	.	.	.	.	.	.	.	.	.	.	.
5069	1	RBBP5	NM_001193272	chr1	203321892	203357773	-	.	.	.	.	.	.	.	.	.	.	.	.	.	.	.	.	.	.	.	.
3612	1	DSTYK	NM_199462	chr1	203378253	203447350	-	.	.	.	.	.	.	.	.	.	.	.	.	.	.	.	.	.	.	.	.
4640	1	NUAK2	NM_030952	chr1	203537813	203557506	-	.	.	.	.	.	.	.	.	.	.	.	.	.	.	.	.	.	.	.	.
2223	1,5	KLHDC8A	NM_018203	chr1	203572270	203592662	-	.	.	.	.	.	.	.	.	.	.	.	.	.	.	.	.	.	.	.	.
4389	1	MFSD4	NM_181644	chr1	203804734	203838669	+	.	.	.	.	.	.	.	.	.	.	.	.	.	.	.	.	.	.	.	.
958	2,5	SLC45A3	NM_033102	chr1	203893603	203916253	-	.	.	.	.	.	.	.	.	.	.	.	.	.	.	.	.	.	.	.	.
4641	1	NUCKS1	NM_022731	chr1	203948569	203985995	-	.	.	.	.	.	.	.	.	.	.	.	.	.	.	.	.	.	.	.	.
5044	1	RAB7L1	NM_001135664	chr1	204003736	204011233	-	.	.	.	.	.	.	.	.	.	.	.	.	.	.	.	.	.	.	.	.
4126	1	IKBKE	NM_001193321	chr1	204710208	204736846	+	.	.	.	.	.	.	.	.	.	.	.	.	.	.	.	.	.	.	.	.
7504	0,25	RASSF5	NM_182663	chr1	204747501	204829239	+	.	.	.	.	.	.	.	.	.	.	.	.	.	.	.	.	2504_217_3	chr1	204795985	+
3635	1	DYRK3	NM_003582	chr1	204875503	204889165	+	.	.	.	.	.	.	.	.	.	.	.	.	.	.	.	.	.	.	.	.
2664	1,25	MAPKAPK2	NM_032960	chr1	204924987	204974253	+	.	.	.	.	.	.	.	.	.	.	.	.	.	.	.	.	.	.	.	.
2624	1,25	IL10	NM_000572	chr1	205007570	205012462	-	.	.	.	.	.	.	.	.	.	.	.	.	.	.	.	.	.	.	.	.
4138	1	IL24	NM_001185157	chr1	205137410	205144107	+	.	.	.	.	.	.	.	.	.	.	.	.	.	.	.	.	.	.	.	.
5725	1	YOD1	NM_018566	chr1	205283816	205291045	-	.	.	.	.	.	.	.	.	.	.	.	.	.	.	.	.	.	.	.	.
4758	1	PFKFB2	NM_006212	chr1	205293242	205317785	+	.	.	.	.	.	.	.	.	.	.	.	.	.	.	.	.	.	.	.	.
3232	1	C4BPA	NM_000715	chr1	205344229	205384940	+	.	.	.	.	.	.	.	.	.	.	.	.	.	.	.	.	.	.	.	.
1887	1,75	CD46	NM_172352	chr1	205992005	206035484	+	.	.	.	.	.	.	.	.	.	.	.	.	.	.	.	.	.	.	.	.
2538	1,25	CD34	NM_001773	chr1	206126505	206151306	-	.	.	.	.	.	.	.	.	.	.	.	.	.	.	.	.	.	.	.	.
1285	2	CAMK1G	NM_020439	chr1	207823667	207853907	+	.	.	.	.	.	.	.	.	.	.	.	.	.	.	.	.	.	.	.	.
2645	1,25	LAMB3	NM_001127641	chr1	207854840	207892297	-	.	.	.	.	.	.	.	.	.	.	.	.	.	.	.	.	.	.	.	.
2145	1,5	G0S2	NM_015714	chr1	207915292	207916358	+	.	.	.	.	.	.	.	.	.	.	.	.	.	.	.	.	.	.	.	.
381	3,5	HSD11B1	NM_181755	chr1	207926147	207974918	+	.	.	.	.	.	.	.	.	.	.	.	.	.	.	.	.	.	.	.	.
1573	2	NEK2	NM_002497	chr1	209902736	209915595	-	.	.	.	.	.	.	.	.	.	.	.	.	.	.	.	.	.	.	.	.
3614	1	DTL	NM_016448	chr1	210275541	210344810	+	.	.	.	.	.	.	.	.	.	.	.	.	.	.	.	.	.	.	.	.
2340	1,5	PPP2R5A	NM_006243	chr1	210525501	210601828	+	.	.	.	.	.	.	.	.	.	.	.	.	.	.	.	.	.	.	.	.
789	2,5	ATF3	NM_001206486	chr1	210854982	210860742	+	.	.	.	.	.	.	.	.	.	.	.	.	.	.	.	.	.	.	.	.
2289	1,5	NSL1	NM_001042549	chr1	210966117	211031762	-	.	.	.	.	.	.	.	.	_144_1_139	chr1	210968126	+	.	.	.	.	.	.	.	.
7208	0,25	C1orf227	NM_001024601	chr1	211070107	211087614	-	.	.	.	.	.	.	.	.	.	.	.	.	.	.	.	.	.	.	.	.
1733	2	RPS6KC1	NM_012424	chr1	211291210	211513431	+	CL800018	chr1	211419066	+	.	.	.	.	.	.	.	.	.	.	.	.	.	.	.	.
2346	1,5	PROX1	NM_002763	chr1	212228466	212281470	+	.	.	.	.	.	.	.	.	.	.	.	.	.	.	.	.	.	.	.	.
1768	2	SMYD2	NM_020197	chr1	212521187	212577100	+	.	.	.	.	.	.	.	.	.	.	.	.	.	.	.	.	.	.	.	.
2349	1,5	PTPN14	NM_005401	chr1	212588661	212791647	-	.	.	.	.	AY516620	chr1	212767972	-	.	.	.	.	.	.	.	.	.	.	.	.
808	2,5	CENPF	NM_016343	chr1	212843154	212904537	+	.	.	.	.	.	.	.	.	.	.	.	.	.	.	.	.	.	.	.	.

Table S2

tumor associated genes								HIV				MLV				MMTV				MMTV(SIN)				MMTV(SIN)arrest			
rank	score	gene symbol	RefSEq	chrom	txStart	txEnd	strand	integrant	chr	position	strand	integrant	chr	position	strand	integrant	chr	position	strand	integrant	chr	position	strand	integrant	chr	position	strand
3700	1	ESRRG	NM_001243505	chr1	214743210	215329819	-	.	.	.	.	.	.	.	.	.	.	.	.	.	.	.	.	.	.	.	.
1973	1,75	TGFB2	NM_001135599	chr1	216585298	216684584	+	.	.	.	.	AY516388	chr1	216621665	-	.	.	.	.	.	.	.	.	.	.	.	.
1973	1,75	TGFB2	NM_001135599	chr1	216585298	216684584	+	.	.	.	.	AY516051	chr1	216623164	+	.	.	.	.	.	.	.	.	.	.	.	.
6916	0,5	SLC30A10	NM_018713	chr1	218154228	218168616	-	.	.	.	.	.	.	.	.	.	.	.	.	.	.	.	.	.	.	.	.
3693	1	EPRS	NM_004446	chr1	218208564	218286623	-	CL800374	chr1	218226996	+	.	.	.	.	.	.	.	.	.	.	.	.	.	.	.	.
3157	1	BPNT1	NM_006085	chr1	218297446	218329814	-	.	.	.	.	.	.	.	.	.	.	.	.	.	.	.	.	.	.	.	.
4105	1	IARS2	NM_018060	chr1	218334077	218388006	+	CL529562	chr1	218362903	+	.	.	.	.	.	.	.	.	.	.	.	.	.	.	.	.
1523	2	MARK1	NM_018650	chr1	218768190	218904422	+	.	.	.	.	.	.	.	.	.	.	.	.	.	.	.	.	.	.	.	.
6090	0,5	C1orf115	NM_024709	chr1	218930250	218939122	+	.	.	.	.	.	.	.	.	.	.	.	.	.	.	.	.	.	.	.	.
1368	2	DUSP10	NM_007207	chr1	219941386	219982139	-	.	.	.	.	.	.	.	.	.	.	.	.	.	.	.	.	.	.	.	.
6990	0,5	SUSD4	NM_001037175	chr1	221474874	221604167	-	.	.	.	.	.	.	.	.	.	.	.	.	.	.	.	.	.	.	.	.
1976	1,75	TP53BP2	NM_005426	chr1	222034217	222100297	-	.	.	.	.	.	.	.	.	.	.	.	.	.	.	.	.	.	.	.	.
7301	0,25	ENAH	NM_018212	chr1	223741156	223907468	-	CL528834	chr1	223788450	-	.	.	.	.	.	.	.	.	.	.	.	.	.	.	.	.
3692	1	EPHX1	NM_000120	chr1	224079624	224099885	+	.	.	.	.	.	.	.	.	.	.	.	.	.	.	.	.	.	.	.	.
5013	1	PYCR2	NM_013328	chr1	224174202	224178588	-	.	.	.	.	.	.	.	.	.	.	.	.	.	.	.	.	.	.	.	.
2231	1,5	LEFTY2	NM_001172425	chr1	224190920	224195706	-	.	.	.	.	.	.	.	.	.	.	.	.	.	.	.	.	.	.	.	.
4000	1	H3F3A	NM_002107	chr1	224317030	224326326	+	.	.	.	.	.	.	.	.	.	.	.	.	.	.	.	.	.	.	.	.
161	4,75	PARP1	NM_001618	chr1	224615014	224662424	-	.	.	.	.	.	.	.	.	.	.	.	.	.	.	.	.	.	.	.	.
1482	2	ITPKB	NM_002221	chr1	224886013	224993499	-	.	.	.	.	.	.	.	.	.	.	.	.	.	.	.	.	.	.	.	.
4967	1	PSEN2	NM_000447	chr1	225124895	225150427	+	.	.	.	.	.	.	.	.	.	.	.	.	.	.	.	.	.	.	.	.
2062	1,5	CDC42BPA	NM_014826	chr1	225244188	225572449	-	AY517023.1	chr1	225453885	-	.	.	.	.	.	.	.	.	.	.	.	.	.	.	.	.
4189	1	JMJD4	NM_001161465	chr1	225985512	225989735	-	.	.	.	.	.	.	.	.	.	.	.	.	.	.	.	.	.	.	.	.
1862	2	WNT9A	NM_003395	chr1	226175787	226202299	-	.	.	.	.	.	.	.	.	.	.	.	.	.	.	.	.	.	.	.	.
5705	1	WNT3A	NM_033131	chr1	226261345	226315595	+	.	.	.	.	.	.	.	.	.	.	.	.	.	.	.	.	.	.	.	.
3015	1	ARF1	NM_001658	chr1	226336983	226353536	+	.	.	.	.	.	.	.	.	.	.	.	.	.	.	.	.	.	.	.	.
853	2,5	GUK1	NM_000858	chr1	226394551	226403278	+	.	.	.	.	.	.	.	.	.	.	.	.	.	.	.	.	.	.	.	.
4654	1	OBSCN	NM_001098623	chr1	226462483	226633198	+	BH609442	chr1	226582094	+	.	.	.	.	.	.	.	.	.	.	.	.	.	.	.	.
7047	0,5	TRIM17	NM_016102	chr1	226662258	226671206	-	.	.	.	.	.	.	.	.	.	.	.	.	.	.	.	.	.	.	.	.
5036	1	RAB4A	NM_004578	chr1	227473501	227507141	+	.	.	.	.	.	.	.	.	.	.	.	.	.	.	.	.	.	.	.	.
5984	0,5	ACTA1	NM_001100	chr1	227633615	227636466	-	.	.	.	.	.	.	.	.	.	.	.	.	.	.	.	.	.	.	.	.
407	3,5	NUP133	NM_018230	chr1	227643666	227710711	-	.	.	.	.	.	.	.	.	.	.	.	.	.	.	.	.	113_79_1	chr1	227689085	+
1195	2	ABCB10	NM_012089	chr1	227718951	227761065	-	.	.	.	.	.	.	.	.	.	.	.	.	.	.	.	.	.	.	.	.
5649	1	URB2	NM_014777	chr1	227828603	227862569	+	.	.	.	.	.	.	.	.	.	.	.	.	.	.	.	.	.	.	.	.
3847	1	GALNT2	NM_004481	chr1	228269578	228484498	+	CL528835	chr1	228295841	+	.	.	.	.	.	.	.	.	.	.	.	.	.	.	.	.
3847	1	GALNT2	NM_004481	chr1	228269578	228484498	+	BH609443	chr1	228336056	-	.	.	.	.	.	.	.	.	.	.	.	.	.	.	.	.
4768	1	PGBD5	NM_001258311	chr1	228524014	228628297	-	.	.	.	.	.	.	.	.	.	.	.	.	.	.	.	.	.	.	.	.
5998	0,5	AGT	NM_000029	chr1	228904891	228916959	-	.	.	.	.	.	.	.	.	.	.	.	.	.	.	.	.	.	.	.	.
3929	1	GNPAT	NM_014236	chr1	229443541	229480342	+	.	.	.	.	.	.	.	.	.	.	.	.	.	.	.	.	.	.	.	.
3658	1	EGLN1	NM_022051	chr1	229566119	229627413	-	CL800754	chr1	229610064	+	.	.	.	.	.	.	.	.	.	.	.	.	.	.	.	.
7053	0,5	TSNAX	NM_005999	chr1	229731021	229768892	+	CL800847	chr1	229746944	-	.	.	.	.	.	.	.	.	.	.	.	.	.	.	.	.
1492	2	KIAA1804	NM_032435	chr1	231530136	231587517	+	.	.	.	.	.	.	.	.	.	.	.	.	.	.	.	.	.	.	.	.
2214	1,5	KCNK1	NM_002245	chr1	231816372	231874881	+	.	.	.	.	.	.	.	.	.	.	.	.	.	.	.	.	.	.	.	.
5273	1	SLC35F3	NM_173508	chr1	232107301	232526885	+	.	.	.	.	.	.	.	.	.	.	.	.	.	.	.	.	.	.	.	.
3031	1	ARID4B	NM_016374	chr1	233396832	233558155	-	.	.	.	.	.	.	.	.	.	.	.	.	.	.	.	.	424_103_1	chr1	233537636	+
3883	1	GGPS1	NR_036605	chr1	233558491	233574467	+	.	.	.	.	.	.	.	.	.	.	.	.	.	.	.	.	.	.	.	.
3925	1	GNG4	NM_001098722	chr1	233777607	233879916	-	.	.	.	.	.	.	.	.	.	.	.	.	.	.	.	.	.	.	.	.
6670	0,5	NID1	NM_002508	chr1	234205754	234295104	-	.	.	.	.	.	.	.	.	.	.	.	.	.	.	.	.	.	.	.	.
7396	0,25	LGALS8	NM_201543	chr1	234753361	234782902	+	.	.	.	.	.	.	.	.	.	.	.	.	.	.	.	.	.	.	.	.
2684	1,25	MTR	NM_000254	chr1	235025203	235133904	+	.	.	.	.	.	.	.	.	.	.	.	.	.	.	.	.	.	.	.	.
5174	1	RYR2	NM_001035	chr1	235272324	236063911	+	.	.	.	.	AY516447	chr1	235615786	+	_384_1_232	chr1	235928621	+	.	.	.	.	.	.	.	.
6360	0,5	FMN2	NM_020066	chr1	238321807	238705112	+	.	.	.	.	.	.	.	.	.	.	.	.	.	.	.	.	.	.	.	.

Table S2

tumor associated genes								HIV				MLV				MMTV				MMTV(SIN)				MMTV(SIN)arrest			
rank	score	gene symbol	RefSEq	chrom	txStart	txEnd	strand	integrant	chr	position	strand	integrant	chr	position	strand	integrant	chr	position	strand	integrant	chr	position	strand	integrant	chr	position	strand
3966	1	GREM2	NM_022469	chr1	238719495	238842085	-	.	.	.	.	.	.	.	.	.	.	.	.	.	.	.	.	2133_185_1	chr1	238834332	+
6816	0,5	RGS7	NM_002924	chr1	239005439	239587101	-	.	.	.	.	.	.	.	.	.	.	.	.	.	.	.	.	.	.	.	.
139	5	FH	NM_000143	chr1	239727479	239749708	-	.	.	.	.	.	.	.	.	.	.	.	.	.	.	.	.	.	.	.	.
4242	1	KMO	NM_003679	chr1	239762056	239825572	+	.	.	.	.	.	.	.	.	.	.	.	.	.	.	.	.	.	.	.	.
3704	1	EXO1	NM_130398	chr1	240078115	240119864	+	.	.	.	.	.	.	.	.	.	.	.	.	.	.	.	.	.	.	.	.
442	3,25	AKT3	NM_181690	chr1	241718157	242073207	-	CL799602	chr1	242054975	-	AY516765	chr1	241753682	+	.	.	.	.	.	.	.	.	.	.	.	.
1226	2	ADSS	NM_001126	chr1	242638416	242682059	-	.	.	.	.	.	.	.	.	.	.	.	.	.	.	.	.	.	.	.	.
6442	0,5	HNRNPU	NM_004501	chr1	243080224	243094450	-	.	.	.	.	.	.	.	.	.	.	.	.	.	.	.	.	.	.	.	.
3650	1	EFCAB2	NR_026586	chr1	243199906	243355153	+	.	.	.	.	.	.	.	.	.	.	.	.	.	.	.	.	.	.	.	.
1578	2	NLRP3	NM_004895	chr1	245647973	245679029	+	.	.	.	.	.	.	.	.	.	.	.	.	.	.	.	.	.	.	.	.
5557	1	TRIM58	NM_015431	chr1	246087123	246110061	+	.	.	.	.	.	.	.	.	.	.	.	.	.	.	.	.	.	.	.	.
4666	1	OR2L13	NM_175911	chr1	246167115	246330847	+	.	.	.	.	.	.	.	.	.	.	.	.	.	.	.	.	.	.	.	.
2887	1	ACP1	NM_001040649	chr2	254868	262481	+	.	.	.	.	.	.	.	.	.	.	.	.	.	.	.	.	.	.	.	.
124	5,25	TPO	NM_175719	chr2	1396239	1525506	+	.	.	.	.	.	.	.	.	.	.	.	.	.	.	.	.	.	.	.	.
1704	2	PXDN	NM_012293	chr2	1614665	1727298	-	CL528837	chr2	1628246	+	.	.	.	.	.	.	.	.	.	.	.	.	.	.	.	.
4482	1	MYT1L	NM_015025	chr2	1771891	2314052	-	.	.	.	.	.	.	.	.	.	.	.	.	26_1_1468_4	chr2	1965814	-	.	.	.	.
5130	1	RNASEH1	NM_002936	chr2	3570565	3583815	-	.	.	.	.	.	.	.	.	.	.	.	.	.	.	.	.	.	.	.	.
2968	1	ALLC	NM_018436	chr2	3683660	3728135	+	.	.	.	.	.	.	.	.	.	.	.	.	.	.	.	.	.	.	.	.
959	2,5	SOX11	NM_003108	chr2	5750249	5758968	+	.	.	.	.	.	.	.	.	.	.	.	.	.	.	.	.	.	.	.	.
2620	1,25	ID2	NM_002166	chr2	8739563	8742034	+	.	.	.	.	.	.	.	.	.	.	.	.	.	.	.	.	.	.	.	.
1208	2	ADAM17	NM_003183	chr2	9546861	9613368	-	CL529657	chr2	9567260	-	.	.	.	.	.	.	.	.	.	.	.	.	.	.	.	.
1865	2	YWHAQ	NM_006826	chr2	9641556	9688557	-	.	.	.	.	.	.	.	.	.	.	.	.	.	.	.	.	.	.	.	.
5169	1	RRM2	NM_001034	chr2	10180313	10188997	+	.	.	.	.	.	.	.	.	.	.	.	.	.	.	.	.	.	.	.	.
4657	1	ODC1	NM_002539	chr2	10497958	10505904	-	.	.	.	.	.	.	.	.	.	.	.	.	.	.	.	.	.	.	.	.
3091	1	ATP6V1C2	NM_001039362	chr2	10779225	10842687	+	.	.	.	.	.	.	.	.	.	.	.	.	.	.	.	.	.	.	.	.
2213	1,5	KCNF1	NM_002236	chr2	10969513	10971802	+	.	.	.	.	.	.	.	.	.	.	.	.	.	.	.	.	.	.	.	.
6357	0,5	FLJ33534	NR_040080	chr2	11157427	11189753	-	.	.	.	.	.	.	.	.	.	.	.	.	.	.	.	.	.	.	.	.
5146	1	ROCK2	NM_004850	chr2	11239228	11402162	-	.	.	.	.	.	.	.	.	.	.	.	.	.	.	.	.	.	.	.	.
7295	0,25	E2F6	NR_003094	chr2	11501951	11523748	-	.	.	.	.	.	.	.	.	.	.	.	.	.	.	.	.	.	.	.	.
1947	1,75	NTSR2	NM_012344	chr2	11715754	11727780	-	.	.	.	.	.	.	.	.	.	.	.	.	.	.	.	.	.	.	.	.
5549	1	TRIB2	NR_027303	chr2	12774448	12800309	+	CL529468	chr2	12780224	-	.	.	.	.	.	.	.	.	.	.	.	.	.	.	.	.
337	3,75	MYCN	NM_005378	chr2	15998133	16004580	+	.	.	.	.	.	.	.	.	.	.	.	.	.	.	.	.	.	.	.	.
2824	1,25	VSNL1	NM_003385	chr2	17585287	17701187	+	.	.	.	.	.	.	.	.	.	.	.	.	.	.	.	.	.	.	.	.
3875	1	GEN1	NM_001130009	chr2	17798894	17830113	+	.	.	.	.	.	.	.	.	.	.	.	.	.	.	.	.	.	.	.	.
5086	1	RDH14	NM_020905	chr2	18599469	18605440	-	.	.	.	.	.	.	.	.	.	.	.	.	.	.	.	.	.	.	.	.
4633	1	NT5C1B	NM_001199086	chr2	18607617	18634327	-	.	.	.	.	.	.	.	.	.	.	.	.	.	.	.	.	.	.	.	.
753	2,75	SDC1	NM_002997	chr2	20264038	20288408	-	.	.	.	.	.	.	.	.	.	.	.	.	.	.	.	.	.	.	.	.
1158	2,25	RHOB	NM_004040	chr2	20510315	20512682	+	.	.	.	.	.	.	.	.	.	.	.	.	.	.	.	.	.	.	.	.
1561	2	NCOA1	NM_147233	chr2	24660849	24847074	+	.	.	.	.	.	.	.	.	_459_1_335	chr2	24799798	+	.	.	.	.	.	.	.	.
3336	1	CENPO	NM_001199803	chr2	24869678	24898749	+	.	.	.	.	.	.	.	.	.	.	.	.	.	.	.	.	.	.	.	.
1217	2	ADCY3	NM_004036	chr2	24895541	24995559	-	.	.	.	.	.	.	.	.	.	.	.	.	s1840_314	chr2	24911604	+	.	.	.	.
6275	0,5	DNAJC27	NM_001198559	chr2	25020008	25048467	-	.	.	.	.	.	.	.	.	.	.	.	.	.	.	.	.	.	.	.	.
2718	1,25	POMC	NM_000939	chr2	25237225	25245063	-	.	.	.	.	.	.	.	.	.	.	.	.	.	.	.	.	.	.	.	.
1364	2	DNMT3A	NM_022552	chr2	25309333	25418288	-	.	.	.	.	.	.	.	.	.	.	.	.	.	.	.	.	.	.	.	.
2112	1,5	DTNB	NM_033148	chr2	25453615	25750020	-	.	.	.	.	.	.	.	.	.	.	.	.	.	.	.	.	.	.	.	.
4005	1	HADHA	NM_000182	chr2	26267007	26321098	-	.	.	.	.	.	.	.	.	.	.	.	.	.	.	.	.	.	.	.	.
4006	1	HADHB	NM_000183	chr2	26321119	26366837	+	.	.	.	.	.	.	.	.	.	.	.	.	.	.	.	.	.	.	.	.
4674	1	OTOF	NM_194248	chr2	26533574	26635070	-	.	.	.	.	.	.	.	.	.	.	.	.	.	.	.	.	.	.	.	.
4202	1	KCNK3	NM_002246	chr2	26769084	26807570	+	.	.	.	.	.	.	.	.	.	.	.	.	.	.	.	.	.	.	.	.
3327	1	CENPA	NM_001042426	chr2	26862386	26870959	+	.	.	.	.	.	.	.	.	.	.	.	.	.	.	.	.	.	.	.	.
7291	0,25	DPYSL5	NM_001253724	chr2	26924795	27026723	+	.	.	.	.	.	.	.	.	.	.	.	.	.	.	.	.	.	.	.	.

Table S2

tumor associated genes								HIV				MLV				MMTV				MMTV(SIN)				MMTV(SIN)arrest			
rank	score	gene symbol	RefSEq	chrom	txStart	txEnd	strand	integrant	chr	position	strand	integrant	chr	position	strand	integrant	chr	position	strand	integrant	chr	position	strand	integrant	chr	position	strand
6594	0,5	MAPRE3	NM_012326	chr2	27047028	27103591	+	.	.	.	.	.	.	.	.	.	.	.	.	.	.	.	.	.	.	.	.
4213	1	KHK	NM_006488	chr2	27163114	27177123	+	.	.	.	.	.	.	.	.	.	.	.	.	.	.	.	.	.	.	.	.
1284	2	CAD	NM_004341	chr2	27293761	27320158	+	.	.	.	.	.	.	.	.	.	.	.	.	.	.	.	.	.	.	.	.
5270	1	SLC30A3	NM_003459	chr2	27330943	27339464	-	.	.	.	.	.	.	.	.	.	.	.	.	.	.	.	.	.	.	.	.
7563	0,25	SNX17	NR_049784	chr2	27446866	27453904	+	.	.	.	.	.	.	.	.	.	.	.	.	.	.	.	.	.	.	.	.
2332	1,5	PPM1G	NM_177983	chr2	27457569	27486054	-	CL528842	chr2	27474457	-	.	.	.	.	.	.	.	.	.	.	.	.	.	.	.	.
6139	0,5	CCDC121	NM_024584	chr2	27702009	27705402	-	.	.	.	.	.	.	.	.	.	.	.	.	.	.	.	.	.	.	.	.
2366	1,5	RBKS	NM_022128	chr2	27857769	27966727	-	.	.	.	.	.	.	.	.	.	.	.	.	.	.	.	.	.	.	.	.
4835	1	PLB1	NM_153021	chr2	28572441	28720157	+	.	.	.	.	.	.	.	.	.	.	.	.	.	.	.	.	.	.	.	.
4905	1	PPP1CB	NM_002709	chr2	28828129	28879310	+	.	.	.	.	.	.	.	.	.	.	.	.	.	.	.	.	.	.	.	.
72	6,25	ALK	NM_004304	chr2	29269143	29997981	-	.	.	.	.	.	.	.	.	.	.	.	.	.	.	.	.	.	.	.	.
7111	0,5	YPEL5	NM_001127401	chr2	30223253	30236903	+	.	.	.	.	.	.	.	.	.	.	.	.	.	.	.	.	.	.	.	.
4264	1	LBH	NM_030915	chr2	30307900	30336403	+	.	.	.	.	.	.	.	.	.	.	.	.	.	.	.	.	.	.	.	.
4266	1	LCLAT1	NM_182551	chr2	30523626	30720595	+	CL799916	chr2	30615850	-	.	.	.	.	.	.	.	.	.	.	.	.	.	.	.	.
1863	2	XDH	NM_000379	chr2	31410691	31491115	-	.	.	.	.	.	.	.	.	.	.	.	.	.	.	.	.	.	.	.	.
1774	2	SRD5A2	NM_000348	chr2	31603159	31659544	-	.	.	.	.	.	.	.	.	.	.	.	.	.	.	.	.	.	.	.	.
6961	0,5	SPAST	NM_014946	chr2	32142183	32236210	+	.	.	.	.	.	.	.	.	.	.	.	.	.	.	.	.	.	.	.	.
238	4	BIRC6	NM_016252	chr2	32435599	32697469	+	.	.	.	.	.	.	.	.	.	.	.	.	.	.	.	.	.	.	.	.
274	4	LTBP1	NM_001166264	chr2	33213167	33478079	+	.	.	.	.	.	.	.	.	.	.	.	.	.	.	.	.	.	.	.	.
5068	1	RASGRP3	NM_001139488	chr2	33554824	33643302	+	.	.	.	.	.	.	.	.	.	.	.	.	.	.	.	.	.	.	.	.
3458	1	CRIM1	NM_016441	chr2	36436873	36631782	+	.	.	.	.	.	.	.	.	.	.	.	.	.	.	.	.	.	.	.	.
1379	2	EIF2AK2	NM_001135652	chr2	37185786	37228469	-	.	.	.	.	.	.	.	.	.	.	.	.	.	.	.	.	.	.	.	.
4943	1	PRKD3	NM_005813	chr2	37331149	37397726	-	.	.	.	.	.	.	.	.	.	.	.	.	.	.	.	.	.	.	.	.
7309	0,25	FAM82A1	NM_001170792	chr2	38009405	38098388	+	.	.	.	.	.	.	.	.	_167_1_152	chr2	38020504	+	.	.	.	.	.	.	.	.
247	4	CYP1B1	NM_000104	chr2	38148249	38156827	-	.	.	.	.	.	.	.	.	.	.	.	.	.	.	.	.	.	.	.	.
3842	1	GALM	NM_138801	chr2	38746555	38815413	+	.	.	.	.	.	.	.	.	.	.	.	.	.	.	.	.	.	.	.	.
194	4,5	SOS1	NM_005633	chr2	39062193	39201108	-	.	.	.	.	.	.	.	.	.	.	.	.	.	.	.	.	3157_305_1	chr2	39154806	-
3319	1	CDKL4	NM_001009565	chr2	39259191	39310177	-	.	.	.	.	.	.	.	.	.	.	.	.	.	.	.	.	.	.	.	.
1520	2	MAP4K3	NM_001270425	chr2	39329910	39517957	-	.	.	.	.	.	.	.	.	.	.	.	.	.	.	.	.	.	.	.	.
6929	0,5	SLC8A1	NM_001112801	chr2	40192789	40510948	-	.	.	.	.	.	.	.	.	.	.	.	.	.	.	.	.	.	.	.	.
1382	2	EML4	NM_019063	chr2	42249993	42413192	+	.	.	.	.	.	.	.	.	.	.	.	.	.	.	.	.	2307_200_2	chr2	42381095	-
4003	1	HAAO	NM_012205	chr2	42847732	42873255	-	.	.	.	.	.	.	.	.	.	.	.	.	.	.	.	.	.	.	.	.
4303	1	LRPPRC	NM_133259	chr2	43966866	44076648	-	.	.	.	.	.	.	.	.	.	.	.	.	.	.	.	.	.	.	.	.
4898	1	PPM1B	NM_002706	chr2	44249503	44312115	+	.	.	.	.	.	.	.	.	.	.	.	.	.	.	.	.	.	.	.	.
4936	1	PREPL	NM_001171603	chr2	44398251	44442505	-	.	.	.	.	.	.	.	.	.	.	.	.	.	.	.	.	.	.	.	.
2344	1,5	PRKCE	NM_005400	chr2	45732546	46268633	+	CL528844	chr2	46201424	-	.	.	.	.	.	.	.	.	.	.	.	.	.	.	.	.
1903	1,75	EPAS1	NM_001430	chr2	46378044	46467346	+	.	.	.	.	AY516173	chr2	46396653	+	.	.	.	.	.	.	.	.	.	.	.	.
3092	1	ATP6V1E2	NM_080653	chr2	46592489	46600600	-	.	.	.	.	.	.	.	.	.	.	.	.	.	.	.	.	.	.	.	.
5119	1	RHOQ	NM_012249	chr2	46623370	46665331	+	.	.	.	.	.	.	.	.	.	.	.	.	.	.	.	.	.	.	.	.
4797	1	PIGF	NM_002643	chr2	46661916	46697755	-	.	.	.	.	.	.	.	.	.	.	.	.	.	.	.	.	.	.	.	.
5314	1	SOCS5	NM_014011	chr2	46779602	46843431	+	.	.	.	.	.	.	.	.	.	.	.	.	.	.	.	.	.	.	.	.
2046	1,5	CALM2	NM_001743	chr2	47240724	47257244	-	.	.	.	.	.	.	.	.	.	.	.	.	.	.	.	.	.	.	.	.
25	8,75	MSH2	NM_001258281	chr2	47483709	47563871	+	.	.	.	.	.	.	.	.	.	.	.	.	.	.	.	.	.	.	.	.
29	8,5	MSH6	NM_000179	chr2	47863724	47887596	+	.	.	.	.	.	.	.	.	.	.	.	.	.	.	.	.	.	.	.	.
3754	1	FBXO11	NM_001190274	chr2	47887562	47986436	-	.	.	.	.	.	.	.	.	.	.	.	.	.	.	.	.	.	.	.	.
6983	0,5	STON1	NM_001198595	chr2	48610811	48679158	+	.	.	.	.	.	.	.	.	.	.	.	.	.	.	.	.	.	.	.	.
4279	1	LHCGR	NM_000233	chr2	48767416	48836384	-	.	.	.	.	.	.	.	.	.	.	.	.	.	.	.	.	.	.	.	.
4625	1	NRXN1	NM_004801	chr2	49999146	51113178	-	CL800703	chr2	50918212	+	AY516561	chr2	50028279	+	.	.	.	.	.	.	.	.	620_113_1	chr2	50553187	-
1987	1,5	ACYP2	NM_138448	chr2	54195913	54385939	+	.	.	.	.	.	.	.	.	.	.	.	.	.	.	.	.	.	.	.	.
6836	0,5	RTN4	NM_207521	chr2	55052830	55129830	-	AY517013.1	chr2	55108821	-	.	.	.	.	.	.	.	.	.	.	.	.	.	.	.	.
6831	0,5	RPS27A	NM_001135592	chr2	55312542	55316493	+	.	.	.	.	.	.	.	.	.	.	.	.	.	.	.	.	.	.	.	.

Table S2

tumor associated genes								HIV				MLV				MMTV				MMTV(SIN)				MMTV(SIN)arrest			
rank	score	gene symbol	RefSEq	chrom	txStart	txEnd	strand	integrant	chr	position	strand	integrant	chr	position	strand	integrant	chr	position	strand	integrant	chr	position	strand	integrant	chr	position	strand
4861	1	PNPT1	NM_033109	chr2	55714701	55774515	-	.	.	.	.	.	.	.	.	.	.	.	.	.	.	.	.	.	.	.	.
5903	0,75	EFEMP1	NM_001039349	chr2	55946600	56004802	-	.	.	.	.	.	.	.	.	.	.	.	.	.	.	.	.	.	.	.	.
6142	0,5	CCDC85A	NM_001080433	chr2	56264761	56466813	+	.	.	.	.	.	.	.	.	.	.	.	.	s993_162	chr2	56300634	+	.	.	.	.
1851	2	VRK2	NR_049781	chr2	58127280	58240559	+	CL528846	chr2	58213465	+	.	.	.	.	.	.	.	.	.	.	.	.	.	.	.	.
3734	1	FANCL	NM_018062	chr2	58239881	58322019	-	CL528847	chr2	58294130	+	.	.	.	.	.	.	.	.	.	.	.	.	.	.	.	.
3734	1	FANCL	NM_018062	chr2	58239881	58322019	-	AY516943.1	chr2	58272686	+	.	.	.	.	.	.	.	.	.	.	.	.	.	.	.	.
3734	1	FANCL	NM_018062	chr2	58239881	58322019	-	CL799616	chr2	58304713	-	.	.	.	.	.	.	.	.	.	.	.	.	.	.	.	.
169	4,5	BCL11A	NM_138559	chr2	60531805	60634137	-	.	.	.	.	.	.	.	.	.	.	.	.	.	.	.	.	.	.	.	.
1156	2,25	REL	NM_002908	chr2	60962255	61003682	+	.	.	.	.	.	.	.	.	.	.	.	.	.	.	.	.	.	.	.	.
5657	1	USP34	NM_014709	chr2	61268093	61551353	-	CL800030	chr2	61331354	+	.	.	.	.	.	.	.	.	.	.	.	.	.	.	.	.
5657	1	USP34	NM_014709	chr2	61268093	61551353	-	CL800454	chr2	61307860	-	.	.	.	.	.	.	.	.	.	.	.	.	.	.	.	.
2461	1,5	XPO1	NM_003400	chr2	61558572	61618922	-	.	.	.	.	.	.	.	.	.	.	.	.	.	.	.	.	.	.	.	.
3722	1	FAM161A	NM_001201543	chr2	61905486	61934782	-	.	.	.	.	.	.	.	.	.	.	.	.	.	.	.	.	.	.	.	.
6144	0,5	CCT4	NM_001256721	chr2	61948765	61969310	-	.	.	.	.	.	.	.	.	.	.	.	.	.	.	.	.	.	.	.	.
3421	1	COMMD1	NM_152516	chr2	61986306	62216709	+	.	.	.	.	.	.	.	.	.	.	.	.	.	.	.	.	.	.	.	.
2018	1,5	B3GNT2	NM_006577	chr2	62276765	62305370	+	.	.	.	.	.	.	.	.	.	.	.	.	.	.	.	.	.	.	.	.
620	3	MDH1	NM_005917	chr2	63669246	63687834	+	.	.	.	.	.	.	.	.	.	.	.	.	.	.	.	.	.	.	.	.
5623	1	UGP2	NM_006759	chr2	63922517	63972200	+	.	.	.	.	.	.	.	.	.	.	.	.	.	.	.	.	.	.	.	.
6730	0,5	PELI1	NM_020651	chr2	64173289	64225109	-	CL528848	chr2	64187053	+	.	.	.	.	.	.	.	.	.	.	.	.	.	.	.	.
5023	1	RAB1A	NM_015543	chr2	65167491	65210939	-	CL528849	chr2	65193733	+	.	.	.	.	.	.	.	.	.	.	.	.	.	.	.	.
2489	1,25	ACTR2	NM_005722	chr2	65308332	65351891	+	.	.	.	.	.	.	.	.	.	.	.	.	.	.	.	.	.	.	.	.
5343	1	SPRED2	NM_001128210	chr2	65391488	65447435	-	.	.	.	.	.	.	.	.	.	.	.	.	.	.	.	.	.	.	.	.
6606	0,5	MEIS1	NM_002398	chr2	66516035	66653395	+	.	.	.	.	.	.	.	.	.	.	.	.	.	.	.	.	.	.	.	.
934	2,5	PPP3R1	NM_000945	chr2	68259492	68333155	-	.	.	.	.	.	.	.	.	.	.	.	.	.	.	.	.	.	.	.	.
3025	1	ARHGAP25	NM_001166276	chr2	68855436	68907461	+	CL800474	chr2	68838709	-	.	.	.	.	_338_1_208	chr2	68897991	-	.	.	.	.	.	.	.	.
3897	1	GKN1	NM_019617	chr2	69055208	69061616	+	.	.	.	.	.	.	.	.	.	.	.	.	.	.	.	.	.	.	.	.
3878	1	GFPT1	NM_002056	chr2	69400404	69467890	-	.	.	.	.	.	.	.	.	.	.	.	.	.	.	.	.	.	.	.	.
2843	1	AAK1	NM_014911	chr2	69538630	69724481	-	.	.	.	.	.	.	.	.	.	.	.	.	.	.	.	.	.	.	.	.
6019	0,5	ANXA4	NM_001153	chr2	69822630	69907100	+	CL800507	chr2	69842439	+	.	.	.	.	.	.	.	.	.	.	.	.	.	.	.	.
4466	1	MXD1	NM_001202513	chr2	69995676	70023580	+	.	.	.	.	.	.	.	.	.	.	.	.	.	.	.	.	.	.	.	.
7016	0,5	TGFA	NM_003236	chr2	70527919	70634655	-	.	.	.	.	.	.	.	.	.	.	.	.	.	.	.	.	.	.	.	.
7086	0,5	VAX2	NM_012476	chr2	70981227	71014083	+	.	.	.	.	.	.	.	.	.	.	.	.	.	.	.	.	.	.	.	.
3089	1	ATP6V1B1	NM_001692	chr2	71016505	71046069	+	.	.	.	.	.	.	.	.	.	.	.	.	.	.	.	.	.	.	.	.
1555	2	NAGK	NM_017567	chr2	71148915	71159506	+	.	.	.	.	.	.	.	.	.	.	.	.	.	.	.	.	.	.	.	.
4370	1	MCEE	NM_032601	chr2	71190313	71210902	-	.	.	.	.	.	.	.	.	.	.	.	.	.	.	.	.	.	.	.	.
6240	0,5	CYP26B1	NM_019885	chr2	72209874	72228471	-	.	.	.	.	.	.	.	.	.	.	.	.	.	.	.	.	.	.	.	.
5342	1	SPR	NM_003124	chr2	72968019	72972797	+	.	.	.	.	.	.	.	.	.	.	.	.	.	.	.	.	.	.	.	.
6307	0,5	EMX1	NM_004097	chr2	72998111	73015528	+	.	.	.	.	.	.	.	.	.	.	.	.	.	.	.	.	.	.	.	.
6145	0,5	CCT7	NM_001166284	chr2	73314871	73333658	+	.	.	.	.	.	.	.	.	.	.	.	.	.	.	.	.	.	.	.	.
6350	0,5	FBXO41	NM_001080410	chr2	73335317	73350266	-	.	.	.	.	.	.	.	.	.	.	.	.	.	.	.	.	.	.	.	.
6302	0,5	EGR4	NM_001965	chr2	73371564	73374337	-	.	.	.	.	.	.	.	.	.	.	.	.	.	.	.	.	.	.	.	.
3617	1	DUSP11	NM_003584	chr2	73842832	73860792	-	CL800067	chr2	73845151	+	.	.	.	.	.	.	.	.	.	.	.	.	.	.	.	.
3571	1	DGUOK	NM_080918	chr2	74007460	74039596	+	.	.	.	.	.	.	.	.	.	.	.	.	.	.	.	.	.	.	.	.
4454	1	MTHFD2	NM_006636	chr2	74279197	74295932	+	.	.	.	.	.	.	.	.	.	.	.	.	.	.	.	.	.	.	.	.
6257	0,5	DCTN1	NM_001190836	chr2	74441788	74472722	-	.	.	.	.	.	.	.	.	.	.	.	.	.	.	.	.	.	.	.	.
4423	1	MOGS	NM_001146158	chr2	74541691	74546045	-	.	.	.	.	.	.	.	.	.	.	.	.	.	.	.	.	.	.	.	.
2109	1,5	DOK1	NM_001197260	chr2	74629654	74638186	+	.	.	.	.	.	.	.	.	.	.	.	.	.	.	.	.	.	.	.	.
6868	0,5	SEMA4F	NM_004263	chr2	74734900	74762693	+	CL800417	chr2	74743999	-	.	.	.	.	.	.	.	.	.	.	.	.	.	.	.	.
1451	2	HK2	NM_000189	chr2	74913289	74973989	+	.	.	.	.	.	.	.	.	.	.	.	.	.	.	.	.	.	.	.	.
1645	2	POLE4	NM_019896	chr2	75039282	75050367	+	.	.	.	.	.	.	.	.	.	.	.	.	.	.	.	.	.	.	.	.
4311	1	LRRTM4	NM_024993	chr2	77597294	77603010	-	.	.	.	.	.	.	.	.	.	.	.	.	.	.	.	.	.	.	.	.

Table S2

tumor associated genes								HIV				MLV				MMTV				MMTV(SIN)				MMTV(SIN)arrest			
rank	score	gene symbol	RefSEq	chrom	txStart	txEnd	strand	integrant	chr	position	strand	integrant	chr	position	strand	integrant	chr	position	strand	integrant	chr	position	strand	integrant	chr	position	strand
364	3,5	CTNNA2	NM_001164883	chr2	79593567	80729499	+	.	.	.	.	.	.	.	.	_156_1_144	chr2	80594202	+	101_1_553_21	chr2	79710584	+	.	.	.	.
5399	1	SUCLG1	NM_003849	chr2	84504157	84540097	-	.	.	.	.	.	.	.	.	.	.	.	.	.	.	.	.	.	.	.	.
2801	1,25	TMSB10	NM_021103	chr2	84986273	84987310	+	.	.	.	.	.	.	.	.	.	.	.	.	.	.	.	.	.	.	.	.
5447	1	TCF7L1	NM_031283	chr2	85214093	85391022	+	.	.	.	.	.	.	.	.	.	.	.	.	.	.	.	.	.	.	.	.
7017	0,5	TGOLN2	NM_001206841	chr2	85398651	85408930	-	.	.	.	.	.	.	.	.	.	.	.	.	.	.	.	.	.	.	.	.
3881	1	GGCX	NM_000821	chr2	85625488	85642168	-	.	.	.	.	.	.	.	.	.	.	.	.	.	.	.	.	.	.	.	.
7615	0,25	VAMP8	NM_003761	chr2	85658124	85662667	+	.	.	.	.	.	.	.	.	.	.	.	.	.	.	.	.	.	.	.	.
7085	0,5	VAMP5	NM_006634	chr2	85665041	85674022	+	.	.	.	.	.	.	.	.	.	.	.	.	.	.	.	.	.	.	.	.
7543	0,25	SFTPB	NM_000542	chr2	85737950	85749375	-	.	.	.	.	.	.	.	.	.	.	.	.	.	.	.	.	.	.	.	.
7331	0,25	GNLY	NM_012483	chr2	85774924	85779386	+	.	.	.	.	.	.	.	.	.	.	.	.	.	.	.	.	.	.	.	.
5360	1	ST3GAL5	NM_003896	chr2	85919781	85969668	-	.	.	.	.	.	.	.	.	.	.	.	.	.	.	.	.	.	.	.	.
1648	2	POLR1A	NM_015425	chr2	86106961	86186789	-	.	.	.	.	.	.	.	.	.	.	.	.	.	.	.	.	.	.	.	.
5092	1	REEP1	NM_001164730	chr2	86294630	86418717	-	.	.	.	.	.	.	.	.	.	.	.	.	.	.	.	.	.	.	.	.
4206	1	KDM3A	NM_001146688	chr2	86522094	86573350	+	.	.	.	.	.	.	.	.	.	.	.	.	.	.	.	.	.	.	.	.
3664	1	EIF2AK3	NM_004836	chr2	88637373	88708209	-	CL529402	chr2	88649904	+	.	.	.	.	.	.	.	.	.	.	.	.	.	.	.	.
5160	1	RPIA	NM_144563	chr2	88772290	88831567	+	.	.	.	.	.	.	.	.	.	.	.	.	.	.	.	.	.	.	.	.
2654	1,25	MAL	NM_022440	chr2	95055126	95083464	+	.	.	.	.	.	.	.	.	.	.	.	.	.	.	.	.	.	.	.	.
6498	0,5	KCNIP3	NM_013434	chr2	95326798	95415552	+	.	.	.	.	.	.	.	.	.	.	.	.	.	.	.	.	.	.	.	.
564	3	DUSP2	NM_004418	chr2	96172634	96174906	-	.	.	.	.	.	.	.	.	.	.	.	.	.	.	.	.	.	.	.	.
5307	1	SNRNP200	NM_014014	chr2	96303800	96335034	-	.	.	.	.	.	.	.	.	.	.	.	.	.	.	.	.	.	.	.	.
4503	1	NCAPH	NM_015341	chr2	96365210	96405001	+	.	.	.	.	.	.	.	.	.	.	.	.	.	.	.	.	.	.	.	.
3406	1	CNNM4	NM_020184	chr2	96790365	96841355	+	.	.	.	.	.	.	.	.	.	.	.	.	.	.	.	.	.	.	.	.
2088	1,5	COX5B	NM_001862	chr2	97628952	97631089	+	.	.	.	.	AY516759	chr2	97629488	+	.	.	.	.	.	.	.	.	.	.	.	.
2466	1,5	ZAP70	NM_001079	chr2	97696462	97722755	+	.	.	.	.	.	.	.	.	.	.	.	.	.	.	.	.	.	.	.	.
6193	0,5	CNGA3	NM_001298	chr2	98329049	98381496	+	.	.	.	.	.	.	.	.	.	.	.	.	.	.	.	.	.	.	.	.
385	3,5	INPP4A	NM_004027	chr2	98427752	98573928	+	.	.	.	.	.	.	.	.	.	.	.	.	.	.	.	.	.	.	.	.
4393	1	MGAT4A	NM_012214	chr2	98602000	98714021	-	BH609455	chr2	98674903	-	.	.	.	.	.	.	.	.	.	.	.	.	.	.	.	.
5569	1	TSGA10	NM_182911	chr2	98980155	99124469	-	.	.	.	.	.	.	.	.	.	.	.	.	.	.	.	.	.	.	.	.
4288	1	LIPT1	NM_145199	chr2	99137849	99146045	+	.	.	.	.	.	.	.	.	.	.	.	.	.	.	.	.	.	.	.	.
5096	1	REV1	NM_001037872	chr2	99383369	99472912	-	CL799924	chr2	99418665	-	.	.	.	.	.	.	.	.	.	.	.	.	.	.	.	.
5096	1	REV1	NM_001037872	chr2	99383369	99472912	-	BH609456	chr2	99426009	-	.	.	.	.	.	.	.	.	.	.	.	.	.	.	.	.
1228	2	AFF3	NM_001025108	chr2	99530147	100088477	-	.	.	.	.	AY516790	chr2	99680116	-	.	.	.	.	2246_111	chr2	99872049	-	.	.	.	.
6678	0,5	NPAS2	NM_002518	chr2	100803044	100979719	+	.	.	.	.	AY515902	chr2	100875154	+	.	.	.	.	.	.	.	.	.	.	.	.
6217	0,5	CREG2	NM_153836	chr2	101331247	101370397	-	.	.	.	.	.	.	.	.	.	.	.	.	.	.	.	.	.	.	.	.
892	2,5	MAP4K4	NM_145686	chr2	101680596	101877584	+	CL800313	chr2	101847709	+	.	.	.	.	.	.	.	.	.	.	.	.	.	.	.	.
4135	1	IL1R2	NM_001261419	chr2	101974737	102007650	+	.	.	.	.	.	.	.	.	.	.	.	.	.	.	.	.	.	.	.	.
731	2,75	IL1R1	NM_000877	chr2	102136833	102162766	+	.	.	.	.	.	.	.	.	.	.	.	.	.	.	.	.	.	.	.	.
6471	0,5	IL1RL1	NM_003856	chr2	102320148	102327614	+	.	.	.	.	.	.	.	.	.	.	.	.	.	.	.	.	.	.	.	.
4134	1	IL18R1	NM_003855	chr2	102345528	102381649	+	.	.	.	.	.	.	.	.	.	.	.	.	.	.	.	.	.	.	.	.
833	2,5	FHL2	NM_001039492	chr2	105343714	105382007	-	.	.	.	.	AY516497	chr2	105359151	+	.	.	.	.	.	.	.	.	.	.	.	.
6652	0,5	NCK2	NM_003581	chr2	105727951	105877162	+	CL528854	chr2	105774823	+	.	.	.	.	.	.	.	.	.	.	.	.	.	.	.	.
6652	0,5	NCK2	NM_003581	chr2	105727951	105877162	+	AY517158.1	chr2	105850887	-	.	.	.	.	.	.	.	.	.	.	.	.	.	.	.	.
5665	1	UXS1	NM_025076	chr2	106076190	106177227	-	.	.	.	.	.	.	.	.	.	.	.	.	.	.	.	.	.	.	.	.
5362	1	ST6GAL2	NM_001142352	chr2	106795963	106869995	-	.	.	.	.	.	.	.	.	.	.	.	.	.	.	.	.	.	.	.	.
5407	1	SULT1C2	NM_176825	chr2	108271526	108292803	+	.	.	.	.	.	.	.	.	.	.	.	.	.	.	.	.	.	.	.	.
5408	1	SULT1C4	NM_006588	chr2	108360852	108370702	+	.	.	.	.	.	.	.	.	.	.	.	.	.	.	.	.	.	.	.	.
7397	0,25	LIMS1	NM_004987	chr2	108571188	108670134	+	CL529411	chr2	108610573	-	.	.	.	.	.	.	.	.	.	.	.	.	.	.	.	.
7397	0,25	LIMS1	NM_004987	chr2	108571188	108670134	+	CL800063	chr2	108538065	+	.	.	.	.	.	.	.	.	.	.	.	.	.	.	.	.
2362	1,5	RANBP2	NM_006267	chr2	108702368	108768699	+	.	.	.	.	.	.	.	.	.	.	.	.	.	.	.	.	.	.	.	.
1006	2,25	BUB1	NM_004336	chr2	111111880	111152155	-	CL529765	chr2	111131168	-	.	.	.	.	.	.	.	.	.	.	.	.	.	.	.	.
7183	0,25	BCL2L11	NM_006538	chr2	111594961	111642493	+	.	.	.	.	.	.	.	.	.	.	.	.	.	.	.	.	1971_175_1	chr2	111622184	-

Table S2

tumor associated genes								HIV				MLV				MMTV				MMTV(SIN)				MMTV(SIN)arrest			
rank	score	gene symbol	RefSEq	chrom	txStart	txEnd	strand	integrant	chr	position	strand	integrant	chr	position	strand	integrant	chr	position	strand	integrant	chr	position	strand	integrant	chr	position	strand
2979	1	ANAPC1	NM_022662	chr2	112241684	112358212	-	.	.	.	.	.	.	.	.	.	.	.	.	.	.	.	.	.	.	.	.
1109	2,25	MERTK	NM_006343	chr2	112372661	112503416	+	.	.	.	.	.	.	.	.	.	.	.	.	.	.	.	.	.	.	.	.
1827	2	TTL	NM_153712	chr2	112956213	113006693	+	.	.	.	.	.	.	.	.	.	.	.	.	.	.	.	.	.	.	.	.
1649	2	POLR1B	NM_019014	chr2	113015962	113051198	+	.	.	.	.	.	.	.	.	.	.	.	.	.	.	.	.	.	.	.	.
1078	2,25	IL1A	NM_000575	chr2	113247962	113259442	-	.	.	.	.	.	.	.	.	.	.	.	.	.	.	.	.	.	.	.	.
730	2,75	IL1B	NM_000576	chr2	113303807	113310827	-	.	.	.	.	.	.	.	.	.	.	.	.	.	.	.	.	.	.	.	.
1079	2,25	IL1RN	NM_173842	chr2	113601608	113608064	+	.	.	.	.	.	.	.	.	.	.	.	.	.	.	.	.	.	.	.	.
636	3	PAX8	NM_013953	chr2	113690044	113752968	-	.	.	.	.	.	.	.	.	.	.	.	.	.	.	.	.	.	.	.	.
5048	1	RABL2A	NM_007082	chr2	114101286	114117445	+	.	.	.	.	.	.	.	.	.	.	.	.	.	.	.	.	.	.	.	.
1365	2	DPP10	NM_001178034	chr2	115635982	116318796	+	.	.	.	.	.	.	.	.	.	.	.	.	.	.	.	.	.	.	.	.
1348	2	DDX18	NM_006773	chr2	118288724	118306423	+	AY517453.1	chr2	118293024	+	.	.	.	.	.	.	.	.	.	.	.	.	.	.	.	.
6308	0,5	EN1	NM_001426	chr2	119316216	119322229	-	.	.	.	.	.	.	.	.	.	.	.	.	.	.	.	.	.	.	.	.
5000	1	PTPN4	NM_002830	chr2	120233676	120458944	+	BH609457	chr2	120428074	-	.	.	.	.	.	.	.	.	.	.	.	.	.	.	.	.
1473	2	INHBB	NM_002193	chr2	120820188	120825853	+	.	.	.	.	.	.	.	.	.	.	.	.	.	.	.	.	.	.	.	.
1913	1,75	GLI2	NM_005270	chr2	121271336	121466699	+	.	.	.	.	.	.	.	.	.	.	.	.	.	.	.	.	.	.	.	.
3378	1	CLASP1	NM_001142274	chr2	121811821	122123522	-	CL529412	chr2	121902262	-	.	.	.	.	.	.	.	.	.	.	.	.	.	.	.	.
7252	0,25	CNTNAP5	NM_130773	chr2	124499333	125389333	+	.	.	.	.	.	.	.	.	.	.	.	.	.	.	.	.	.	.	.	.
6419	0,5	GYPC	NM_016815	chr2	127129980	127170721	+	.	.	.	.	.	.	.	.	.	.	.	.	.	.	.	.	.	.	.	.
2513	1,25	BIN1	NM_139350	chr2	127522068	127581373	-	.	.	.	.	.	.	.	.	.	.	.	.	.	.	.	.	.	.	.	.
32	8,25	ERCC3	NM_000122	chr2	127731335	127768222	-	.	.	.	.	.	.	.	.	.	.	.	.	.	.	.	.	.	.	.	.
2657	1,25	MAP3K2	NM_006609	chr2	127772714	127817275	-	.	.	.	.	.	.	.	.	.	.	.	.	.	.	.	.	.	.	.	.
1915	1,75	GPR17	NM_001161416	chr2	128119908	128126683	+	.	.	.	.	.	.	.	.	.	.	.	.	.	.	.	.	.	.	.	.
1652	2	POLR2D	NM_004805	chr2	128320309	128332199	-	.	.	.	.	.	.	.	.	.	.	.	.	.	.	.	.	.	.	.	.
4085	1	HS6ST1	NM_004807	chr2	128739523	128792641	-	.	.	.	.	.	.	.	.	.	.	.	.	.	.	.	.	.	.	.	.
5042	1	RAB6C	NM_032144	chr2	130453704	130456781	+	.	.	.	.	.	.	.	.	.	.	.	.	.	.	.	.	.	.	.	.
4144	1	IMP4	NM_033416	chr2	130816958	130820667	+	.	.	.	.	.	.	.	.	.	.	.	.	.	.	.	.	.	.	.	.
4994	1	PTPN18	NM_001142370	chr2	130830049	130849452	+	.	.	.	.	.	.	.	.	.	.	.	.	.	.	.	.	.	.	.	.
6330	0,5	FAM123C	NM_001105194	chr2	131230333	131242177	+	.	.	.	.	.	.	.	.	.	.	.	.	.	.	.	.	.	.	.	.
2674	1,25	MGAT5	NM_002410	chr2	134728299	134928662	+	.	.	.	.	.	.	.	.	.	.	.	.	.	.	.	.	.	.	.	.
2881	1	ACMSD	NM_138326	chr2	135312655	135376072	+	.	.	.	.	.	.	.	.	.	.	.	.	.	.	.	.	.	.	.	.
5726	1	YSK4	NM_025052	chr2	135438742	135498718	-	.	.	.	.	.	.	.	.	.	.	.	.	.	.	.	.	.	.	.	.
6785	0,5	R3HDM1	NM_015361	chr2	136005552	136199309	+	AY517160.1	chr2	136142571	+	.	.	.	.	.	.	.	.	.	.	.	.	.	.	.	.
4270	1	LCT	NM_002299	chr2	136261884	136311220	-	.	.	.	.	.	.	.	.	.	.	.	.	.	.	.	.	.	.	.	.
3529	1	DARS	NM_001349	chr2	136380723	136459692	-	.	.	.	.	.	.	.	.	.	.	.	.	.	.	.	.	.	.	.	.
365	3,5	CXCR4	NM_001008540	chr2	136588388	136590283	-	.	.	.	.	.	.	.	.	.	.	.	.	.	.	.	.	.	.	.	.
4065	1	HNMT	NM_001024074	chr2	138438277	138441484	+	.	.	.	.	.	.	.	.	.	.	.	.	.	.	.	.	.	.	.	.
6697	0,5	NXPH2	NM_007226	chr2	139143196	139254281	-	.	.	.	.	.	.	.	.	.	.	.	.	.	.	.	.	.	.	.	.
1096	2,25	LRP1B	NM_018557	chr2	140705465	142605740	-	.	.	.	.	.	.	.	.	.	.	.	.	38_1_1818_5	chr2	141578213	-	.	.	.	.
2227	1,5	KYNU	NM_001199241	chr2	143351664	143516355	+	.	.	.	.	.	.	.	.	.	.	.	.	.	.	.	.	.	.	.	.
7168	0,25	ARHGAP15	NM_018460	chr2	143603368	144242391	+	CL529501	chr2	143716956	+	AY515919	chr2	143925661	-	.	.	.	.	.	.	.	.	.	.	.	.
2469	1,5	ZEB2	NR_033258	chr2	144985421	144994428	-	.	.	.	.	.	.	.	.	.	.	.	.	.	.	.	.	.	.	.	.
774	2,5	ACVR2A	NM_001616	chr2	148319039	148404863	+	CL528857	chr2	148350477	-	.	.	.	.	.	.	.	.	.	.	.	.	.	.	.	.
6315	0,5	EPC2	NM_015630	chr2	149119029	149261606	+	.	.	.	.	.	.	.	.	.	.	.	.	.	.	.	.	.	.	.	.
2381	1,5	RND3	NM_001254738	chr2	151032952	151052455	-	.	.	.	.	.	.	.	.	.	.	.	.	.	.	.	.	.	.	.	.
2444	1,5	TNFAIP6	NM_007115	chr2	151922351	151944808	+	.	.	.	.	.	.	.	.	.	.	.	.	.	.	.	.	.	.	.	.
1726	2	RIF1	NM_001177663	chr2	151974642	152042106	+	CL528858	chr2	152039872	+	.	.	.	.	.	.	.	.	.	.	.	.	.	.	.	.
1726	2	RIF1	NM_001177663	chr2	151974642	152042106	+	AY516922.1	chr2	152035640	+	.	.	.	.	.	.	.	.	.	.	.	.	.	.	.	.
6979	0,5	STAM2	NM_005843	chr2	152681560	152740752	-	.	.	.	.	.	.	.	.	.	.	.	.	.	.	.	.	.	.	.	.
7313	0,25	FMNL2	NM_052905	chr2	152899996	153214594	+	.	.	.	.	.	.	.	.	.	.	.	.	.	.	.	.	.	.	.	.
5953	0,75	RPRM	NM_019845	chr2	154042097	154043568	-	.	.	.	.	.	.	.	.	.	.	.	.	.	.	.	.	.	.	.	.
3846	1	GALNT13	NM_052917	chr2	154436671	155018735	+	.	.	.	.	.	.	.	.	.	.	.	.	.	.	.	.	.	.	.	.

Table S2

tumor associated genes								HIV				MLV				MMTV				MMTV(SIN)				MMTV(SIN)arrest			
rank	score	gene symbol	RefSEq	chrom	txStart	txEnd	strand	integrant	chr	position	strand	integrant	chr	position	strand	integrant	chr	position	strand	integrant	chr	position	strand	integrant	chr	position	strand
5931	0,75	KCNJ3	NM_001260509	chr2	155263338	155274608	+	.	.	.	.	.	.	.	.	.	.	.	.	.	.	.	.	.	.	.	.
1583	2	NR4A2	NM_006186	chr2	156889189	156897533	-	.	.	.	.	.	.	.	.	.	.	.	.	.	.	.	.	.	.	.	.
3943	1	GPD2	NM_001083112	chr2	157000210	157151161	+	CL529456	chr2	157091916	+	.	.	.	.	.	.	.	.	.	.	.	.	.	.	.	.
579	3	GALNT5	NM_014568	chr2	157822585	157876159	+	.	.	.	.	.	.	.	.	.	.	.	.	.	.	.	.	.	.	.	.
6321	0,5	ERMN	NM_020711	chr2	157883370	157890662	-	.	.	.	.	.	.	.	.	.	.	.	.	.	.	.	.	.	.	.	.
6245	0,5	CYTIP	NM_004288	chr2	157979376	158008850	-	.	.	.	.	.	.	.	.	.	.	.	.	.	.	.	.	.	.	.	.
2897	1	ACVR1C	NM_001111033	chr2	158091524	158193645	-	.	.	.	.	.	.	.	.	.	.	.	.	.	.	.	.	.	.	.	.
5987	0,5	ACVR1	NM_001105	chr2	158301204	158439869	-	.	.	.	.	.	.	.	.	.	.	.	.	.	.	.	.	.	.	.	.
5644	1	UPP2	NM_001135098	chr2	158559936	158700912	+	.	.	.	.	.	.	.	.	.	.	.	.	.	.	.	.	.	.	.	.
4319	1	LY75	NM_002349	chr2	160368113	160469513	-	.	.	.	.	.	.	.	.	.	.	.	.	.	.	.	.	.	.	.	.
6487	0,5	ITGB6	NM_000888	chr2	160666478	160764836	-	.	.	.	.	.	.	.	.	.	.	.	.	.	.	.	.	.	.	.	.
6803	0,5	RBMS1	NM_002897	chr2	160836907	161058564	-	CL799860	chr2	160858419	-	.	.	.	.	.	.	.	.	.	.	.	.	.	.	.	.
7009	0,5	TBR1	NM_006593	chr2	161980865	161989819	+	.	.	.	.	.	.	.	.	.	.	.	.	.	.	.	.	.	.	.	.
5280	1	SLC4A10	NM_001178016	chr2	162189090	162550032	+	CL800219	chr2	162516552	+	.	.	.	.	.	.	.	.	.	.	.	.	.	.	.	.
6286	0,5	DPP4	NM_001935	chr2	162557000	162639298	-	.	.	.	.	.	.	.	.	.	.	.	.	.	.	.	.	.	.	.	.
2600	1,25	GCG	NM_002054	chr2	162707624	162717160	-	.	.	.	.	.	.	.	.	.	.	.	.	.	.	.	.	.	.	.	.
1908	1,75	FAP	NM_004460	chr2	162735445	162808291	-	.	.	.	.	.	.	.	.	.	.	.	.	.	.	.	.	.	.	.	.
4117	1	IFIH1	NM_022168	chr2	162831834	162883464	-	CL529598	chr2	162853913	-	.	.	.	.	.	.	.	.	.	.	.	.	.	.	.	.
3964	1	GRB14	NM_004490	chr2	165057568	165186606	-	.	.	.	.	.	.	.	.	.	.	.	.	.	.	.	.	.	.	.	.
5198	1	SCN3A	NM_006922	chr2	165652275	165768823	-	.	.	.	.	.	.	.	.	.	.	.	.	.	.	.	.	.	.	.	.
6856	0,5	SCN2A	NM_001040142	chr2	165804157	165957066	+	.	.	.	.	.	.	.	.	.	.	.	.	.	.	.	.	.	.	.	.
2596	1,25	GALNT3	NM_004482	chr2	166312558	166359049	-	.	.	.	.	.	.	.	.	.	.	.	.	.	.	.	.	.	.	.	.
6854	0,5	SCN1A	NM_001165964	chr2	166553915	166638395	-	.	.	.	.	.	.	.	.	.	.	.	.	.	.	.	.	.	.	.	.
5199	1	SCN9A	NM_002977	chr2	166759942	166940743	-	CL800379	chr2	166902465	-	.	.	.	.	.	.	.	.	.	.	.	.	.	.	.	.
2016	1,5	B3GALT1	NM_020981	chr2	168383427	168435612	+	.	.	.	.	.	.	.	.	.	.	.	.	.	.	.	.	.	.	.	.
5388	1	STK39	NM_013233	chr2	168518775	168812351	-	CL529527	chr2	168705810	-	.	.	.	.	.	.	.	.	.	.	.	.	.	.	.	.
6677	0,5	NOSTRIN	NM_052946	chr2	169367352	169430095	+	.	.	.	.	.	.	.	.	.	.	.	.	.	.	.	.	.	.	.	.
5326	1	SPC25	NM_020675	chr2	169435646	169455190	-	.	.	.	.	.	.	.	.	.	.	.	.	.	.	.	.	.	.	.	.
3827	1	G6PC2	NM_021176	chr2	169465995	169474756	+	.	.	.	.	.	.	.	.	.	.	.	.	.	.	.	.	.	.	.	.
1196	2	ABCB11	NM_003742	chr2	169487694	169596079	-	.	.	.	.	.	.	.	.	.	.	.	.	.	.	.	.	.	.	.	.
3577	1	DHRS9	NM_005771	chr2	169629544	169660923	+	.	.	.	.	.	.	.	.	.	.	.	.	.	.	.	.	.	.	.	.
1510	2	LRP2	NM_004525	chr2	169691864	169927368	-	.	.	.	.	.	.	.	.	.	.	.	.	.	.	.	.	.	.	.	.
3739	1	FASTKD1	NM_024622	chr2	170094508	170138670	-	.	.	.	.	.	.	.	.	.	.	.	.	.	.	.	.	.	.	.	.
4790	1	PHOSPHO2	NM_001199288	chr2	170259209	170266465	+	.	.	.	.	.	.	.	.	.	.	.	.	.	.	.	.	.	.	.	.
4479	1	MYO3B	NR_045682	chr2	170742900	171219920	+	BH609462	chr2	171084297	+	.	.	.	.	.	.	.	.	.	.	.	.	.	.	.	.
5321	1	SP5	NM_001003845	chr2	171280102	171282744	+	.	.	.	.	.	.	.	.	.	.	.	.	.	.	.	.	.	.	.	.
2148	1,5	GAD1	NM_000817	chr2	171381445	171425905	+	.	.	.	.	.	.	.	.	.	.	.	.	.	.	.	.	.	.	.	.
5476	1	TLK1	NM_001136554	chr2	171555578	171796070	-	.	.	.	.	.	.	.	.	.	.	.	.	.	.	.	.	.	.	.	.
6238	0,5	CYBRD1	NM_001127383	chr2	172087111	172122889	+	.	.	.	.	.	.	.	.	.	.	.	.	.	.	.	.	.	.	.	.
2179	1,5	HAT1	NM_003642	chr2	172487180	172556846	+	.	.	.	.	.	.	.	.	.	.	.	.	.	.	.	.	.	.	.	.
6268	0,5	DLX1	NM_001038493	chr2	172658453	172662647	+	.	.	.	.	.	.	.	.	.	.	.	.	.	.	.	.	.	.	.	.
7278	0,25	DLX2	NM_004405	chr2	172672411	172675724	-	.	.	.	.	.	.	.	.	.	.	.	.	.	.	.	.	.	.	.	.
1925	1,75	ITGA6	NM_000210	chr2	173000559	173079427	+	.	.	.	.	.	.	.	.	.	.	.	.	.	.	.	.	.	.	.	.
1607	2	PDK1	NM_002610	chr2	173129024	173172108	+	.	.	.	.	.	.	.	.	.	.	.	.	.	.	.	.	.	.	.	.
5062	1	RAPGEF4	NM_007023	chr2	173308770	173625866	+	.	.	.	.	.	.	.	.	.	.	.	.	.	.	.	.	.	.	.	.
2465	1,5	ZAK	NM_133646	chr2	173648810	173800119	+	CL528863	chr2	173742209	+	.	.	.	.	.	.	.	.	.	.	.	.	.	.	.	.
6160	0,5	CDCA7	NM_031942	chr2	173927806	173941964	+	.	.	.	.	.	.	.	.	.	.	.	.	.	.	.	.	.	.	.	.
6959	0,5	SP3	NM_001017371	chr2	174479432	174537193	-	AY517092.1	chr2	174490481	-	.	.	.	.	.	.	.	.	.	.	.	.	.	.	.	.
7098	0,5	WIPF1	NM_001077269	chr2	175132547	175255873	-	.	.	.	.	.	.	.	.	.	.	.	.	.	.	.	.	.	.	.	.
6178	0,5	CHRNA1	NM_000079	chr2	175320568	175337446	-	.	.	.	.	.	.	.	.	.	.	.	.	.	.	.	.	.	.	.	.
320	3,75	CHN1	NM_001822	chr2	175372287	175578227	-	.	.	.	.	.	.	.	.	.	.	.	.	.	.	.	.	.	.	.	.

Table S2

tumor associated genes								HIV				MLV				MMTV				MMTV(SIN)				MMTV(SIN)arrest			
rank	score	gene symbol	RefSEq	chrom	txStart	txEnd	strand	integrant	chr	position	strand	integrant	chr	position	strand	integrant	chr	position	strand	integrant	chr	position	strand	integrant	chr	position	strand
788	2,5	ATF2	NM_001256092	chr2	175645223	175741180	-	.	.	.	.	.	.	.	.	.	.	.	.	.	.	.	.	.	.	.	.
3077	1	ATP5G3	NM_001002258	chr2	175749231	175754637	-	.	.	.	.	.	.	.	.	.	.	.	.	.	.	.	.	.	.	.	.
589	3	HOXD13	NM_000523	chr2	176665777	176668912	+	.	.	.	.	.	.	.	.	.	.	.	.	.	.	.	.	.	.	.	.
265	4	HOXD11	NM_021192	chr2	176680329	176682562	+	.	.	.	.	.	.	.	.	.	.	.	.	.	.	.	.	.	.	.	.
6449	0,5	HOXD10	NM_002148	chr2	176689737	176692916	+	.	.	.	.	.	.	.	.	.	.	.	.	.	.	.	.	.	.	.	.
4074	1	HOXD9	NM_014213	chr2	176695658	176697891	+	.	.	.	.	.	.	.	.	.	.	.	.	.	.	.	.	.	.	.	.
5918	0,75	HOXD3	NM_006898	chr2	176737050	176746072	+	.	.	.	.	.	.	.	.	.	.	.	.	.	.	.	.	.	.	.	.
6438	0,5	HNRNPA3	NM_194247	chr2	177785667	177796931	+	.	.	.	.	.	.	.	.	.	.	.	.	.	.	.	.	.	.	.	.
2929	1	AGPS	NM_003659	chr2	177965716	178116810	+	.	.	.	.	.	.	.	.	.	.	.	.	.	.	.	.	.	.	.	.
4730	1	PDE11A	NM_001077197	chr2	178196222	178681312	-	.	.	.	.	.	.	.	.	.	.	.	.	.	.	.	.	.	.	.	.
2345	1,5	PRKRA	NM_001139518	chr2	179004386	179023601	-	.	.	.	.	.	.	.	.	.	.	.	.	.	.	.	.	.	.	.	.
1828	2	TTN	NM_133379	chr2	179318291	179380395	-	.	.	.	.	.	.	.	.	.	.	.	.	59_1_2118_7	chr2	179110944	+	.	.	.	.
7134	0,5	ZNF385B	NM_001113398	chr2	180014955	180135560	-	CL528864	chr2	180095557	-	.	.	.	.	.	.	.	.	.	.	.	.	.	.	.	.
5601	1	UBE2E3	NM_006357	chr2	181553586	181636395	+	.	.	.	.	.	.	.	.	.	.	.	.	.	.	.	.	.	.	.	.
2208	1,5	ITGA4	NM_000885	chr2	182029863	182110713	+	.	.	.	.	.	.	.	.	.	.	.	.	.	.	.	.	.	.	.	.
3342	1	CERKL	NM_001160277	chr2	182109645	182230079	-	.	.	.	.	.	.	.	.	.	.	.	.	.	.	.	.	.	.	.	.
4571	1	NEUROD1	NM_002500	chr2	182249077	182253637	-	.	.	.	.	.	.	.	.	.	.	.	.	.	.	.	.	.	.	.	.
4914	1	PPP1R1C	NM_001080545	chr2	182558795	182690728	+	AY517330.1	chr2	182567009	+	.	.	.	.	.	.	.	.	.	.	.	.	.	.	.	.
1602	2	PDE1A	NM_001003683	chr2	182740858	183095817	-	.	.	.	.	AY516448	chr2	182863557	-	.	.	.	.	.	.	.	.	.	.	.	.
6363	0,5	FRZB	NM_001463	chr2	183406249	183439743	-	.	.	.	.	.	.	.	.	.	.	.	.	.	.	.	.	.	.	.	.
3623	1	DUSP19	NM_080876	chr2	183651531	183672967	+	.	.	.	.	.	.	.	.	.	.	.	.	.	.	.	.	.	.	.	.
6691	0,5	NUP35	NM_138285	chr2	183697327	183734653	+	.	.	.	.	.	.	.	.	.	.	.	.	.	.	.	.	.	.	.	.
7141	0,5	ZNF804A	NM_194250	chr2	185171337	185512459	+	CL800467	chr2	185190677	+	.	.	.	.	.	.	.	.	.	.	.	.	.	.	.	.
388	3,5	ITGAV	NM_001145000	chr2	187163034	187253873	+	.	.	.	.	.	.	.	.	.	.	.	.	.	.	.	.	.	.	.	.
829	2,5	FAM171B	NM_177454	chr2	187267033	187336757	+	.	.	.	.	.	.	.	.	.	.	.	.	.	.	.	.	.	.	.	.
6122	0,5	CALCRL	NM_005795	chr2	187916093	188021266	-	.	.	.	.	.	.	.	.	.	.	.	.	.	.	.	.	.	.	.	.
7584	0,25	TFPI	NM_006287	chr2	188037202	188127464	-	.	.	.	.	.	.	.	.	.	.	.	.	.	.	.	.	.	.	.	.
6418	0,5	GULP1	NM_016315	chr2	188864640	189168897	+	CL799853	chr2	189100224	-	.	.	.	.	.	.	.	.	.	.	.	.	.	.	.	.
548	3	COL3A1	NM_000090	chr2	189547343	189585717	+	.	.	.	.	.	.	.	.	.	.	.	.	.	.	.	.	.	.	.	.
416	3,5	PMS1	NM_001128144	chr2	190357055	190450600	+	CL800743	chr2	190374674	-	.	.	.	.	.	.	.	.	.	.	.	.	.	.	.	.
416	3,5	PMS1	NM_001128144	chr2	190357055	190450600	+	BH609465	chr2	190409036	+	.	.	.	.	.	.	.	.	.	.	.	.	.	.	.	.
7438	0,25	MSTN	NM_005259	chr2	190628670	190635700	-	.	.	.	.	.	.	.	.	.	.	.	.	.	.	.	.	.	.	.	.
4042	1	HIBCH	NM_014362	chr2	190777604	190893016	-	.	.	.	.	.	.	.	.	.	.	.	.	.	.	.	.	.	.	.	.
600	3	INPP1	NM_001128928	chr2	190916440	190944636	+	.	.	.	.	.	.	.	.	.	.	.	.	.	.	.	.	.	.	.	.
2602	1,25	GLS	NM_001256310	chr2	191453791	191508260	+	CL800049	chr2	191521211	-	.	.	.	.	.	.	.	.	.	.	.	.	.	.	.	.
2602	1,25	GLS	NM_001256310	chr2	191453791	191508260	+	CL528866	chr2	191474768	+	.	.	.	.	.	.	.	.	.	.	.	.	.	.	.	.
756	2,75	STAT1	NM_139266	chr2	191548507	191587221	-	.	.	.	.	.	.	.	.	.	.	.	.	.	.	.	.	.	.	.	.
1780	2	STAT4	NM_003151	chr2	191602546	191724231	-	.	.	.	.	.	.	.	.	.	.	.	.	.	.	.	.	.	.	.	.
4477	1	MYO1B	NM_001130158	chr2	191818351	191998360	+	.	.	.	.	.	.	.	.	.	.	.	.	.	.	.	.	.	.	.	.
969	2,5	TMEFF2	NM_016192	chr2	192522991	192767889	-	CL528867	chr2	192570584	+	.	.	.	.	.	.	.	.	22_1_1283_4	chr2	192729008	-	.	.	.	.
5378	1	STK17B	NM_004226	chr2	196706551	196744581	-	.	.	.	.	.	.	.	.	.	.	.	.	.	.	.	.	.	.	.	.
4027	1	HECW2	NM_020760	chr2	196772221	197165580	-	CL529750	chr2	196961655	-	.	.	.	.	.	.	.	.	.	.	.	.	.	.	.	.
4766	1	PGAP1	NM_024989	chr2	197405972	197499699	-	CL800546	chr2	197417136	-	.	.	.	.	.	.	.	.	.	.	.	.	.	.	.	.
1524	2	MARS2	NM_138395	chr2	198278272	198281359	+	.	.	.	.	.	.	.	.	.	.	.	.	.	.	.	.	.	.	.	.
2714	1,25	PLCL1	NM_006226	chr2	198377670	198722853	+	.	.	.	.	.	.	.	.	.	.	.	.	.	.	.	.	.	.	.	.
5227	1	SGOL2	NM_152524	chr2	201099109	201157063	+	.	.	.	.	.	.	.	.	.	.	.	.	.	.	.	.	.	.	.	.
2996	1	AOX1	NM_001159	chr2	201158975	201244462	+	.	.	.	.	.	.	.	.	_366_1_224	chr2	201182639	-	.	.	.	.	.	.	.	.
2552	1,25	CLK1	NR_027855	chr2	201425976	201437712	-	.	.	.	.	.	.	.	.	.	.	.	.	.	.	.	.	.	.	.	.
4538	1	NDUFB3	NM_002491	chr2	201644706	201658718	+	.	.	.	.	.	.	.	.	.	.	.	.	.	.	.	.	.	.	.	.
3345	1	CFLAR	NM_001202516	chr2	201689121	201745656	+	.	.	.	.	.	.	.	.	.	.	.	.	.	.	.	.	.	.	.	.
2531	1,25	CASP10	NM_032974	chr2	201755865	201802374	+	.	.	.	.	.	.	.	.	.	.	.	.	.	.	.	.	.	.	.	.

Table S2

tumor associated genes								HIV				MLV				MMTV				MMTV(SIN)				MMTV(SIN)arrest			
rank	score	gene symbol	RefSEq	chrom	txStart	txEnd	strand	integrant	chr	position	strand	integrant	chr	position	strand	integrant	chr	position	strand	integrant	chr	position	strand	integrant	chr	position	strand
711	2,75	CASP8	NM_001080125	chr2	201830998	201860679	+	.	.	.	.	.	.	.	.	.	.	.	.	.	.	.	.	.	.	.	.
1243	2	ALS2	NM_001135745	chr2	202332425	202354140	-	.	.	.	.	.	.	.	.	.	.	.	.	.	.	.	.	.	.	.	.
578	3	FZD7	NM_003507	chr2	202607554	202611405	+	.	.	.	.	.	.	.	.	.	.	.	.	.	.	.	.	.	.	.	.
795	2,5	BMPR2	NM_001204	chr2	202949294	203140719	+	.	.	.	.	.	.	.	.	.	.	.	.	.	.	.	.	.	.	.	.
2746	1,25	RAPH1	NM_203365	chr2	204014436	204108303	-	.	.	.	.	.	.	.	.	.	.	.	.	.	.	.	.	.	.	.	.
2099	1,5	CTLA4	NM_005214	chr2	204440755	204446928	+	.	.	.	.	.	.	.	.	.	.	.	.	.	.	.	.	.	.	.	.
1122	2,25	NRP2	NM_201267	chr2	206255468	206350125	+	.	.	.	.	.	.	.	.	.	.	.	.	.	.	.	.	.	.	.	.
4546	1	NDUFS1	NM_005006	chr2	206696047	206732488	-	.	.	.	.	.	.	.	.	.	.	.	.	.	.	.	.	.	.	.	.
3740	1	FASTKD2	NM_014929	chr2	207338356	207369156	+	.	.	.	.	.	.	.	.	.	.	.	.	.	.	.	.	.	.	.	.
1017	2,25	CREB1	NM_134442	chr2	208102860	208178529	+	.	.	.	.	.	.	.	.	.	.	.	.	.	.	.	.	.	.	.	.
3825	1	FZD5	NM_003468	chr2	208335554	208342388	-	.	.	.	.	.	.	.	.	.	.	.	.	.	.	.	.	.	.	.	.
266	4	IDH1	NM_005896	chr2	208809197	208828051	-	.	.	.	.	.	.	.	.	.	.	.	.	.	.	.	.	.	.	.	.
640	3	PIKFYVE	NM_001178000	chr2	208839235	208878516	+	.	.	.	.	.	.	.	.	.	.	.	.	.	.	.	.	.	.	.	.
483	3,25	MAP2	NM_031845	chr2	210152647	210307079	+	.	.	.	.	.	.	.	.	.	.	.	.	.	.	.	.	.	.	.	.
5157	1	RPE	NM_006916	chr2	210575596	210594195	+	.	.	.	.	.	.	.	.	.	.	.	.	.	.	.	.	.	.	.	.
2870	1	ACADL	NM_001608	chr2	210760960	210798460	-	.	.	.	.	.	.	.	.	.	.	.	.	.	.	.	.	.	.	.	.
3451	1	CPS1	NM_001122634	chr2	211166323	211252076	+	.	.	.	.	.	.	.	.	.	.	.	.	.	.	.	.	.	.	.	.
137	5	ERBB4	NM_001042599	chr2	211948686	213111597	-	.	.	.	.	.	.	.	.	.	.	.	.	.	.	.	.	.	.	.	.
128	5	BARD1	NM_000465	chr2	215301519	215382673	-	CL528871	chr2	215317108	-	.	.	.	.	.	.	.	.	.	.	.	.	.	.	.	.
128	5	BARD1	NM_000465	chr2	215301519	215382673	-	CL529698	chr2	215325389	+	.	.	.	.	.	.	.	.	.	.	.	.	.	.	.	.
2850	1	ABCA12	NM_015657	chr2	215504510	215605055	-	.	.	.	.	.	.	.	.	.	.	.	.	282_80	chr2	215682877	-	.	.	.	.
531	3	ATIC	NM_004044	chr2	215884923	215922741	+	.	.	.	.	.	.	.	.	.	.	.	.	.	.	.	.	.	.	.	.
180	4,5	FN1	NM_212482	chr2	215933423	216009036	-	.	.	.	.	.	.	.	.	.	.	.	.	.	.	.	.	.	.	.	.
4751	1	PECR	NM_018441	chr2	216611355	216654784	-	.	.	.	.	.	.	.	.	.	.	.	.	.	.	.	.	.	.	.	.
1980	1,75	XRCC5	NM_021141	chr2	216682264	216779261	+	.	.	.	.	.	.	.	.	.	.	.	.	.	.	.	.	.	.	.	.
5293	1	SMARCAL1	NM_001127207	chr2	216985717	217056019	+	.	.	.	.	.	.	.	.	.	.	.	.	.	.	.	.	.	.	.	.
861	2,5	IGFBP2	NM_000597	chr2	217206371	217237403	+	.	.	.	.	.	.	.	.	.	.	.	.	.	.	.	.	.	.	.	.
1076	2,25	IGFBP5	NM_000599	chr2	217245072	217268517	-	.	.	.	.	.	.	.	.	.	.	.	.	.	.	.	.	.	.	.	.
7025	0,5	TMBIM1	NM_022152	chr2	218847160	218865524	-	.	.	.	.	.	.	.	.	.	.	.	.	.	.	.	.	.	.	.	.
6897	0,5	SLC11A1	NM_000578	chr2	218954995	218969861	+	.	.	.	.	.	.	.	.	.	.	.	.	.	.	.	.	.	.	.	.
4837	1	PLCD4	NM_032726	chr2	219180731	219210153	+	.	.	.	.	.	.	.	.	.	.	.	.	.	.	.	.	.	.	.	.
2423	1,5	STK36	NM_015690	chr2	219244992	219275684	+	.	.	.	.	.	.	.	.	.	.	.	.	.	.	.	.	.	.	.	.
3505	1	CYP27A1	NM_000784	chr2	219354715	219388260	+	.	.	.	.	.	.	.	.	.	.	.	.	.	.	.	.	.	.	.	.
4942	1	PRKAG3	NM_017431	chr2	219395349	219404756	-	.	.	.	.	.	.	.	.	.	.	.	.	.	.	.	.	.	.	.	.
1861	2	WNT6	NM_006522	chr2	219432789	219447198	+	.	.	.	.	.	.	.	.	.	.	.	.	.	.	.	.	.	.	.	.
5703	1	WNT10A	NM_025216	chr2	219453498	219466895	+	.	.	.	.	.	.	.	.	.	.	.	.	.	.	.	.	.	.	.	.
3316	1	CDK5R2	NM_003936	chr2	219532593	219535121	+	.	.	.	.	.	.	.	.	.	.	.	.	.	.	.	.	.	.	.	.
1403	2	FEV	NM_017521	chr2	219554052	219558623	-	.	.	.	.	.	.	.	.	.	.	.	.	.	.	.	.	.	.	.	.
4578	1	NHEJ1	NM_024782	chr2	219648289	219733831	-	CL800250	chr2	219664379	-	.	.	.	.	.	.	.	.	.	.	.	.	.	.	.	.
2853	1	ABCB6	NM_005689	chr2	219782731	219791956	-	.	.	.	.	.	.	.	.	.	.	.	.	.	.	.	.	.	.	.	.
5376	1	STK16	NR_026909	chr2	219818444	219823303	+	.	.	.	.	.	.	.	.	.	.	.	.	.	.	.	.	.	.	.	.
7067	0,5	TUBA4A	NM_006000	chr2	219823244	219826882	-	.	.	.	.	.	.	.	.	.	.	.	.	.	.	.	.	.	.	.	.
2734	1,25	PTPRN	NM_002846	chr2	219862588	219882539	-	.	.	.	.	.	.	.	.	.	.	.	.	.	.	.	.	.	.	.	.
6808	0,5	RESP18	NM_001007089	chr2	219900374	219906143	-	.	.	.	.	.	.	.	.	.	.	.	.	.	.	.	.	.	.	.	.
1033	2,25	DES	NM_001927	chr2	219991342	219999705	+	.	.	.	.	.	.	.	.	.	.	.	.	.	.	.	.	.	.	.	.
5330	1	SPEG	NM_005876	chr2	220007943	220066598	+	.	.	.	.	.	.	.	.	.	.	.	.	.	.	.	.	.	.	.	.
3910	1	GMPPA	NM_013335	chr2	220071830	220079962	+	.	.	.	.	.	.	.	.	.	.	.	.	.	.	.	.	.	.	.	.
3357	1	CHPF	NM_001195731	chr2	220111912	220116063	-	.	.	.	.	.	.	.	.	.	.	.	.	.	.	.	.	.	.	.	.
4148	1	INHA	NM_002191	chr2	220145197	220148679	+	.	.	.	.	.	.	.	.	.	.	.	.	.	.	.	.	.	.	.	.
5375	1	STK11IP	NM_052902	chr2	220170839	220189417	+	.	.	.	.	.	.	.	.	.	.	.	.	.	.	.	.	.	.	.	.
826	2,5	EPHA4	NM_004438	chr2	221990990	222145254	-	CL528874	chr2	222069056	-	.	.	.	.	.	.	.	.	.	.	.	.	.	.	.	.

Table S2

tumor associated genes								HIV				MLV				MMTV				MMTV(SIN)				MMTV(SIN)arrest			
rank	score	gene symbol	RefSEq	chrom	txStart	txEnd	strand	integrant	chr	position	strand	integrant	chr	position	strand	integrant	chr	position	strand	integrant	chr	position	strand	integrant	chr	position	strand
1126	2,25	PAX3	NM_013942	chr2	222866592	222871959	-	.	.	.	.	.	.	.	.	.	.	.	.	.	.	.	.	.	.	.	.
954	2,5	SGPP2	NM_152386	chr2	222997565	223131861	+	.	.	.	.	.	.	.	.	.	.	.	.	81_1_2775_20	chr2	223029475	+	.	.	.	.
3738	1	FARSB	NM_005687	chr2	223144405	223229071	-	CL800097	chr2	223176078	-	.	.	.	.	.	.	.	.	.	.	.	.	.	.	.	.
1985	1,5	ACSL3	NM_203372	chr2	223433975	223516363	+	BH609468	chr2	223473930	-	.	.	.	.	.	.	.	.	.	.	.	.	.	.	.	.
6850	0,5	SCG2	NM_003469	chr2	224169901	224175461	-	.	.	.	.	.	.	.	.	.	.	.	.	.	.	.	.	3277_340_1	chr2	224171440	+
3487	1	CUL3	NM_003590	chr2	225043110	225158358	-	.	.	.	.	.	.	.	.	.	.	.	.	.	.	.	.	.	.	.	.
141	5	IRS1	NM_005544	chr2	227304276	227371750	-	.	.	.	.	.	.	.	.	.	.	.	.	.	.	.	.	.	.	.	.
5116	1	RHBDD1	NM_001167608	chr2	227408914	227572167	+	BH609469	chr2	227419256	+	.	.	.	.	.	.	.	.	.	.	.	.	.	.	.	.
5116	1	RHBDD1	NM_001167608	chr2	227408914	227572167	+	CL529691	chr2	227499782	-	.	.	.	.	.	.	.	.	.	.	.	.	.	.	.	.
3416	1	COL4A4	NM_000092	chr2	227575670	227737519	-	.	.	.	.	.	.	.	.	.	.	.	.	.	.	.	.	.	.	.	.
2086	1,5	COL4A3	NM_000091	chr2	227737524	227887752	+	.	.	.	.	.	.	.	.	.	.	.	.	.	.	.	.	.	.	.	.
7587	0,25	TM4SF20	NM_024795	chr2	227935117	227952266	-	.	.	.	.	.	.	.	.	.	.	.	.	.	.	.	.	.	.	.	.
683	3	SPHKAP	NM_001142644	chr2	228552913	228754605	-	.	.	.	.	.	.	.	.	.	.	.	.	.	.	.	.	.	.	.	.
6277	0,5	DNER	NM_139072	chr2	229930588	230287530	-	.	.	.	.	.	.	.	.	.	.	.	.	.	.	.	.	.	.	.	.
1822	2	TRIP12	NM_004238	chr2	230340173	230494899	-	.	.	.	.	.	.	.	.	.	.	.	.	.	.	.	.	.	.	.	.
5320	1	SP110	NM_004509	chr2	230741889	230793071	-	.	.	.	.	.	.	.	.	.	.	.	.	.	.	.	.	.	.	.	.
6958	0,5	SP100	NM_001080391	chr2	230989114	231118561	+	.	.	.	.	.	.	.	.	.	.	.	.	.	.	.	.	.	.	.	.
6488	0,5	ITM2C	NM_001012514	chr2	231437864	231452207	+	.	.	.	.	.	.	.	.	.	.	.	.	.	.	.	.	.	.	.	.
3116	1	B3GNT7	NM_145236	chr2	231968578	231974119	+	.	.	.	.	.	.	.	.	.	.	.	.	.	.	.	.	.	.	.	.
1560	2	NCL	NM_005381	chr2	232027702	232037449	-	.	.	.	.	.	.	.	.	.	.	.	.	.	.	.	.	.	.	.	.
2348	1,5	PTMA	NM_002823	chr2	232281478	232286494	+	.	.	.	.	.	.	.	.	.	.	.	.	.	.	.	.	.	.	.	.
2311	1,5	PDE6D	NM_002601	chr2	232305390	232354218	-	CL800429	chr2	232313248	-	.	.	.	.	.	.	.	.	.	.	.	.	.	.	.	.
7449	0,25	NPPC	NM_024409	chr2	232495048	232499282	-	.	.	.	.	.	.	.	.	.	.	.	.	.	.	.	.	.	.	.	.
991	2,25	ALPP	NM_001632	chr2	232951591	232955843	+	.	.	.	.	.	.	.	.	.	.	.	.	.	.	.	.	.	.	.	.
2972	1	ALPPL2	NM_031313	chr2	232979795	232983669	+	.	.	.	.	.	.	.	.	.	.	.	.	.	.	.	.	.	.	.	.
1242	2	ALPI	NM_001631	chr2	233029076	233032986	+	.	.	.	.	.	.	.	.	.	.	.	.	.	.	.	.	.	.	.	.
6305	0,5	EIF4E2	NM_004846	chr2	233123600	233142164	+	.	.	.	.	.	.	.	.	.	.	.	.	.	.	.	.	.	.	.	.
6298	0,5	EFHD1	NM_001243252	chr2	233179010	233255735	+	.	.	.	.	.	.	.	.	.	.	.	.	.	.	.	.	.	.	.	.
2280	1,5	NGEF	NM_001114090	chr2	233451639	233501105	-	.	.	.	.	AY516661	chr2	233476504	-	.	.	.	.	.	.	.	.	.	.	.	.
4568	1	NEU2	NM_005383	chr2	233605625	233608011	+	.	.	.	.	.	.	.	.	.	.	.	.	.	.	.	.	.	.	.	.
2627	1,25	INPP5D	NM_005541	chr2	233633279	233781288	+	.	.	.	.	.	.	.	.	.	.	.	.	.	.	.	.	.	.	.	.
368	3,5	DGKD	NM_003648	chr2	233961538	234045482	+	.	.	.	.	.	.	.	.	.	.	.	.	.	.	.	.	.	.	.	.
5628	1	UGT1A8	NM_019076	chr2	234191029	234346684	+	.	.	.	.	.	.	.	.	.	.	.	.	.	.	.	.	.	.	.	.
2819	1,25	UGT1A10	NM_019075	chr2	234209861	234346690	+	.	.	.	.	.	.	.	.	.	.	.	.	.	.	.	.	.	.	.	.
5629	1	UGT1A9	NM_021027	chr2	234245282	234346690	+	.	.	.	.	.	.	.	.	.	.	.	.	.	.	.	.	.	.	.	.
5627	1	UGT1A7	NM_019077	chr2	234255322	234346684	+	.	.	.	.	.	.	.	.	.	.	.	.	.	.	.	.	.	.	.	.
2820	1,25	UGT1A6	NM_001072	chr2	234266250	234346690	+	.	.	.	.	.	.	.	.	.	.	.	.	.	.	.	.	.	.	.	.
5626	1	UGT1A5	NM_019078	chr2	234286376	234346684	+	.	.	.	.	.	.	.	.	.	.	.	.	.	.	.	.	.	.	.	.
5625	1	UGT1A4	NM_007120	chr2	234292176	234346684	+	.	.	.	.	.	.	.	.	.	.	.	.	.	.	.	.	.	.	.	.
5624	1	UGT1A3	NM_019093	chr2	234302511	234346684	+	.	.	.	.	.	.	.	.	.	.	.	.	.	.	.	.	.	.	.	.
1179	2,25	UGT1A1	NM_000463	chr2	234333657	234346684	+	.	.	.	.	.	.	.	.	.	.	.	.	.	.	.	.	.	.	.	.
3035	1	ARL4C	NM_005737	chr2	235066424	235070432	-	.	.	.	.	.	.	.	.	.	.	.	.	.	.	.	.	.	.	.	.
6481	0,5	IQCA1	NR_073043	chr2	236897528	237080917	-	.	.	.	.	.	.	.	.	.	.	.	.	.	.	.	.	.	.	.	.
1022	2,25	CXCR7	NM_020311	chr2	237143118	237155733	+	.	.	.	.	.	.	.	.	.	.	.	.	.	.	.	.	.	.	.	.
6203	0,5	COL6A3	NM_004369	chr2	237897393	237987589	-	.	.	.	.	.	.	.	.	.	.	.	.	.	.	.	.	.	.	.	.
2233	1,5	LRRFIP1	NM_001137551	chr2	238265545	238355029	+	CL528879	chr2	238210241	+	.	.	.	.	_225_1_169	chr2	238349729	+	.	.	.	.	2128_185_1	chr2	238301072	-
5197	1	SCLY	NM_016510	chr2	238634303	238672793	+	.	.	.	.	.	.	.	.	.	.	.	.	.	.	.	.	.	.	.	.
4142	1	ILKAP	NM_030768	chr2	238743781	238777063	-	AY517097.1	chr2	238750950	-	.	.	.	.	.	.	.	.	.	.	.	.	.	.	.	.
4033	1	HES6	NM_018645	chr2	238811646	238813420	-	.	.	.	.	.	.	.	.	.	.	.	.	.	.	.	.	.	.	.	.
262	4	HDAC4	NM_006037	chr2	239634800	239987580	-	.	.	.	.	AY516678	chr2	239862429	+	.	.	.	.	1608_328	chr2	239784146	+	.	.	.	.
2273	1,5	NDUFA10	NM_004544	chr2	240545461	240613492	-	.	.	.	.	.	.	.	.	.	.	.	.	.	.	.	.	.	.	.	.

Table S2

tumor associated genes								HIV				MLV				MMTV				MMTV(SIN)				MMTV(SIN)arrest			
rank	score	gene symbol	RefSEq	chrom	txStart	txEnd	strand	integrant	chr	position	strand	integrant	chr	position	strand	integrant	chr	position	strand	integrant	chr	position	strand	integrant	chr	position	strand
3940	1	GPC1	NM_002081	chr2	241023787	241056168	+	.	.	.	.	.	.	.	.	.	.	.	.	.	.	.	.	.	.	.	.
3628	1	DUSP28	NM_001033575	chr2	241148143	241152104	+	.	.	.	.	.	.	.	.	.	.	.	.	.	.	.	.	.	.	.	.
2930	1	AGXT	NM_000030	chr2	241456834	241467209	+	.	.	.	.	.	.	.	.	.	.	.	.	.	.	.	.	.	.	.	.
4703	1	PASK	NM_001252124	chr2	241701722	241737592	-	.	.	.	.	.	.	.	.	.	.	.	.	.	.	.	.	.	.	.	.
4919	1	PPP1R7	NM_002712	chr2	241738574	241771112	+	CL528881	chr2	241751961	-	.	.	.	.	.	.	.	.	.	.	.	.	.	.	.	.
4025	1	HDLBP	NM_005336	chr2	241815354	241903788	-	.	.	.	.	.	.	.	.	.	.	.	.	.	.	.	.	.	.	.	.
5380	1	STK25	NM_006374	chr2	242083104	242096707	-	.	.	.	.	.	.	.	.	.	.	.	.	.	.	.	.	.	.	.	.
2113	1,5	DTYMK	NM_012145	chr2	242263829	242275056	-	.	.	.	.	.	.	.	.	.	.	.	.	.	.	.	.	.	.	.	.
2204	1,5	ING5	NM_032329	chr2	242290128	242317569	+	.	.	.	.	.	.	.	.	.	.	.	.	.	.	.	.	.	.	.	.
4570	1	NEU4	NM_001167599	chr2	242400702	242407412	+	.	.	.	.	.	.	.	.	.	.	.	.	.	.	.	.	.	.	.	.
4728	1	PDCD1	NM_005018	chr2	242440705	242449731	-	.	.	.	.	.	.	.	.	.	.	.	.	.	.	.	.	.	.	.	.
362	3,5	CHL1	NM_006614	chr3	213278	426097	+	CL528882	chr3	304882	-	.	.	.	.	.	.	.	.	.	.	.	.	.	.	.	.
2083	1,5	CNTN6	NM_014461	chr3	1109628	1420278	+	.	.	.	.	.	.	.	.	.	.	.	.	.	.	.	.	.	.	.	.
2082	1,5	CNTN4	NM_001206956	chr3	2908898	3074645	+	.	.	.	.	.	.	.	.	.	.	.	.	.	.	.	.	.	.	.	.
6215	0,5	CRBN	NM_001173482	chr3	3166316	3196401	-	.	.	.	.	.	.	.	.	.	.	.	.	.	.	.	.	.	.	.	.
1483	2	ITPR1	NM_001168272	chr3	4510031	4864524	+	CL529525	chr3	4826377	+	.	.	.	.	.	.	.	.	.	.	.	.	.	.	.	.
7188	0,25	BHLHE40	NM_003670	chr3	4996096	5001865	+	.	.	.	.	.	.	.	.	.	.	.	.	.	.	.	.	.	.	.	.
3039	1	ARL8B	NM_018184	chr3	5138929	5197601	+	.	.	.	.	AY516866	chr3	5178398	-	.	.	.	.	.	.	.	.	.	.	.	.
6415	0,5	GRM7	NM_181874	chr3	6877801	7758218	+	.	.	.	.	AY516227	chr3	7356962	+	.	.	.	.	.	.	.	.	.	.	.	.
6134	0,5	CAV3	NM_033337	chr3	8750485	8763451	+	.	.	.	.	.	.	.	.	.	.	.	.	.	.	.	.	.	.	.	.
5049	1	RAD18	NM_020165	chr3	8893879	8980159	-	.	.	.	.	.	.	.	.	.	.	.	.	.	.	.	.	.	.	.	.
6971	0,5	SRGAP3	NM_014850	chr3	8997275	9266369	-	.	.	.	.	.	.	.	.	.	.	.	.	s1865_328	chr3	9206606	+	.	.	.	.
6213	0,5	CPNE9	NM_153635	chr3	9720509	9746592	+	.	.	.	.	.	.	.	.	.	.	.	.	.	.	.	.	.	.	.	.
6066	0,5	BRPF1	NM_004634	chr3	9748433	9764699	+	.	.	.	.	.	.	.	.	.	.	.	.	.	.	.	.	.	.	.	.
4659	1	OGG1	NM_002542	chr3	9766627	9774089	+	.	.	.	.	.	.	.	.	.	.	.	.	.	.	.	.	.	.	.	.
3270	1	CAMK1	NM_003656	chr3	9774028	9786668	-	.	.	.	.	.	.	.	.	.	.	.	.	.	.	.	.	.	.	.	.
6774	0,5	PRRT3	NM_207351	chr3	9962225	9969078	-	.	.	.	.	.	.	.	.	.	.	.	.	.	.	.	.	.	.	.	.
157	4,75	FANCD2	NM_033084	chr3	10043112	10116344	+	.	.	.	.	.	.	.	.	.	.	.	.	.	.	.	.	.	.	.	.
70	6,5	VHL	NM_000551	chr3	10158318	10170354	+	.	.	.	.	.	.	.	.	.	.	.	.	.	.	.	.	.	.	.	.
4164	1	IRAK2	NM_001570	chr3	10181562	10260427	+	.	.	.	.	.	.	.	.	.	.	.	.	.	.	.	.	.	.	.	.
5204	1	SEC13	NM_001136232	chr3	10317614	10337725	-	.	.	.	.	.	.	.	.	.	.	.	.	.	.	.	.	.	.	.	.
6043	0,5	ATP2B2	NM_001683	chr3	10340706	10522268	-	.	.	.	.	.	.	.	.	.	.	.	.	.	.	.	.	.	.	.	.
6921	0,5	SLC6A11	NM_014229	chr3	10832916	10955146	+	.	.	.	.	.	.	.	.	.	.	.	.	.	.	.	.	.	.	.	.
6920	0,5	SLC6A1	NM_003042	chr3	11009419	11055935	+	.	.	.	.	.	.	.	.	.	.	.	.	.	.	.	.	.	.	.	.
4080	1	HRH1	NM_001098211	chr3	11242668	11279939	+	.	.	.	.	.	.	.	.	.	.	.	.	.	.	.	.	.	.	.	.
5674	1	VGLL4	NM_014667	chr3	11572543	11737220	-	.	.	.	.	.	.	.	.	.	.	.	.	.	.	.	.	.	.	.	.
5418	1	SYN2	NM_003178	chr3	12020861	12202226	+	.	.	.	.	.	.	.	.	.	.	.	.	2159_73	chr3	12112559	+	.	.	.	.
7022	0,5	TIMP4	NM_003256	chr3	12169567	12175851	-	.	.	.	.	.	.	.	.	.	.	.	.	.	.	.	.	.	.	.	.
189	4,5	PPARG	NM_138712	chr3	12304348	12450855	+	BH609484	chr3	12364515	-	.	.	.	.	.	.	.	.	.	.	.	.	.	.	.	.
344	3,75	RAF1	NM_002880	chr3	12600099	12680700	-	.	.	.	.	.	.	.	.	.	.	.	.	.	.	.	.	.	.	.	.
2293	1,5	NUP210	NM_024923	chr3	13332736	13436809	-	.	.	.	.	.	.	.	.	.	.	.	.	.	.	.	.	.	.	.	.
4024	1	HDAC11	NM_024827	chr3	13496714	13522924	+	.	.	.	.	.	.	.	.	.	.	.	.	.	.	.	.	.	.	.	.
6346	0,5	FBLN2	NM_001998	chr3	13565624	13654923	+	.	.	.	.	.	.	.	.	.	.	.	.	.	.	.	.	.	.	.	.
7101	0,5	WNT7A	NM_004625	chr3	13835082	13896619	-	.	.	.	.	.	.	.	.	.	.	.	.	.	.	.	.	.	.	.	.
7034	0,5	TMEM43	NM_024334	chr3	14141440	14160181	+	.	.	.	.	.	.	.	.	.	.	.	.	.	.	.	.	.	.	.	.
126	5,25	XPC	NM_001145769	chr3	14161648	14195176	-	.	.	.	.	.	.	.	.	.	.	.	.	.	.	.	.	.	.	.	.
4611	1	NR2C2	NM_003298	chr3	14964239	15065784	+	.	.	.	.	.	.	.	.	.	.	.	.	.	.	.	.	.	.	.	.
4385	1	METTL6	NM_152396	chr3	15426380	15444046	-	.	.	.	.	.	.	.	.	.	.	.	.	.	.	.	.	.	.	.	.
3164	1	BTD	NM_000060	chr3	15618258	15662329	+	.	.	.	.	.	.	.	.	.	.	.	.	.	.	.	.	.	.	.	.
3851	1	GALNTL2	NM_054110	chr3	16191187	16246257	+	.	.	.	.	.	.	.	.	.	.	.	.	.	.	.	.	.	.	.	.
5183	1	SATB1	NM_001195470	chr3	18364136	18441833	-	.	.	.	.	.	.	.	.	.	.	.	.	.	.	.	.	.	.	.	.

Table S2

tumor associated genes								HIV				MLV				MMTV				MMTV(SIN)				MMTV(SIN)arrest			
rank	score	gene symbol	RefSEq	chrom	txStart	txEnd	strand	integrant	chr	position	strand	integrant	chr	position	strand	integrant	chr	position	strand	integrant	chr	position	strand	integrant	chr	position	strand
4200	1	KCNH8	NM_144633	chr3	19165020	19552139	+	.	.	.	.	.	.	.	.	.	.	.	.	.	.	.	.	.	.	.	.
2739	1,25	RAB5A	NM_004162	chr3	19963575	20001671	+	.	.	.	.	.	.	.	.	.	.	.	.	.	.	.	.	.	.	.	.
270	4	KAT2B	NM_003884	chr3	20056527	20170900	+	.	.	.	.	.	.	.	.	.	.	.	.	.	.	.	.	.	.	.	.
5226	1	SGOL1	NM_001012411	chr3	20184939	20202702	-	.	.	.	.	.	.	.	.	.	.	.	.	.	.	.	.	.	.	.	.
5600	1	UBE2E2	NM_152653	chr3	23219787	23607300	+	.	.	.	.	.	.	.	.	.	.	.	.	.	.	.	.	.	.	.	.
1832	2	UBE2E1	NM_001202476	chr3	23826937	23908135	+	.	.	.	.	.	.	.	.	.	.	.	.	.	.	.	.	.	.	.	.
4579	1	NKIRAS1	NM_020345	chr3	23908575	23933541	-	.	.	.	.	.	.	.	.	.	.	.	.	.	.	.	.	.	.	.	.
4605	1	NR1D2	NM_005126	chr3	23961754	23997113	+	.	.	.	.	.	.	.	.	.	.	.	.	.	.	.	.	.	.	.	.
1174	2,25	THRB	NM_001252634	chr3	24133648	24511317	-	.	.	.	.	.	.	.	.	.	.	.	.	.	.	.	.	.	.	.	.
666	3	RARB	NM_016152	chr3	25444757	25614426	+	.	.	.	.	.	.	.	.	.	.	.	.	.	.	.	.	.	.	.	.
695	3	TOP2B	NM_001068	chr3	25614478	25680792	-	CL529717	chr3	25654896	+	.	.	.	.	.	.	.	.	.	.	.	.	.	.	.	.
4678	1	OXSM	NR_026937	chr3	25806566	25811029	+	.	.	.	.	.	.	.	.	.	.	.	.	.	.	.	.	.	.	.	.
6576	0,5	LRRC3B	NM_052953	chr3	26639303	26727269	+	.	.	.	.	.	.	.	.	.	.	.	.	.	.	.	.	.	.	.	.
904	2,5	NEK10	NM_199347	chr3	27232100	27385916	-	.	.	.	.	.	.	.	.	.	.	.	.	s1485_227	chr3	27294505	-	.	.	.	.
93	6	TGFBR2	NM_001024847	chr3	30622997	30710637	+	CL529738	chr3	30636338	+	.	.	.	.	.	.	.	.	.	.	.	.	.	.	.	.
5393	1	STT3B	NM_178862	chr3	31549494	31652560	+	CL800722	chr3	31594092	-	.	.	.	.	.	.	.	.	.	.	.	.	.	.	.	.
3942	1	GPD1L	NM_015141	chr3	32123006	32185211	+	.	.	.	.	.	.	.	.	.	.	.	.	.	.	.	.	.	.	.	.
5558	1	TRIM71	NM_001039111	chr3	32834513	32908775	+	.	.	.	.	.	.	.	.	.	.	.	.	.	.	.	.	.	.	.	.
2537	1,25	CCR4	NM_005508	chr3	32968069	32971407	+	.	.	.	.	.	.	.	.	.	.	.	.	.	.	.	.	.	.	.	.
3899	1	GLB1	NM_000404	chr3	33013103	33113698	-	.	.	.	.	.	.	.	.	.	.	.	.	.	.	.	.	.	.	.	.
2793	1,25	SUSD5	NM_015551	chr3	33166540	33235711	-	.	.	.	.	.	.	.	.	.	.	.	.	.	.	.	.	.	.	.	.
3747	1	FBXL2	NM_012157	chr3	33293937	33403761	+	.	.	.	.	.	.	.	.	.	.	.	.	.	.	.	.	.	.	.	.
2077	1,5	CLASP2	NM_001207044	chr3	33512741	33675937	-	.	.	.	.	.	.	.	.	.	.	.	.	.	.	.	.	.	.	.	.
6978	0,5	STAC	NM_003149	chr3	36397100	36564500	+	.	.	.	.	.	.	.	.	.	.	.	.	.	.	.	.	.	.	.	.
3536	1	DCLK3	NM_033403	chr3	36728916	36756356	-	.	.	.	.	.	.	.	.	.	.	.	.	.	.	.	.	.	.	.	.
24	8,75	MLH1	NM_001258271	chr3	37009844	37067341	+	.	.	.	.	.	.	.	.	.	.	.	.	.	.	.	.	.	.	.	.
7213	0,25	C3orf35	NM_178339	chr3	37415971	37451992	+	.	.	.	.	.	.	.	.	.	.	.	.	.	.	.	.	.	.	.	.
4175	1	ITGA9	NM_002207	chr3	37468816	37836285	+	.	.	.	.	.	.	.	.	.	.	.	.	.	.	.	.	1571_154_1	chr3	37667404	+
1633	2	PLCD1	NM_001130964	chr3	38023990	38041282	-	.	.	.	.	.	.	.	.	.	.	.	.	.	.	.	.	.	.	.	.
459	3,25	DLEC1	NM_007337	chr3	38055699	38139232	+	.	.	.	.	.	.	.	.	.	.	.	.	.	.	.	.	.	.	.	.
2864	1	ACAA1	NM_001607	chr3	38139204	38153737	-	.	.	.	.	.	.	.	.	.	.	.	.	.	.	.	.	.	.	.	.
4470	1	MYD88	NM_002468	chr3	38154972	38159516	+	.	.	.	.	.	.	.	.	.	.	.	.	.	.	.	.	.	.	.	.
5717	1	XYLB	NM_005108	chr3	38363254	38431471	+	.	.	.	.	.	.	.	.	.	.	.	.	.	.	.	.	.	.	.	.
5988	0,5	ACVR2B	NM_001106	chr3	38470793	38509637	+	.	.	.	.	.	.	.	.	.	.	.	.	.	.	.	.	.	.	.	.
7257	0,25	CSRNP1	NM_033027	chr3	39158345	39170106	-	.	.	.	.	.	.	.	.	.	.	.	.	.	.	.	.	.	.	.	.
5712	1	XIRP1	NM_194293	chr3	39199710	39209081	-	.	.	.	.	.	.	.	.	.	.	.	.	.	.	.	.	.	.	.	.
3490	1	CX3CR1	NM_001171171	chr3	39279988	39297768	-	.	.	.	.	.	.	.	.	.	.	.	.	.	.	.	.	.	.	.	.
2757	1,25	RPSA	NM_001012321	chr3	39424114	39429036	+	.	.	.	.	.	.	.	.	.	.	.	.	.	.	.	.	.	.	.	.
2261	1,5	MOBP	NM_182935	chr3	39484073	39532498	+	.	.	.	.	.	.	.	.	.	.	.	.	.	.	.	.	.	.	.	.
6645	0,5	MYRIP	NM_015460	chr3	39826306	40276815	+	.	.	.	.	.	.	.	.	.	.	.	.	.	.	.	.	.	.	.	.
2124	1,5	ENTPD3	NM_001248	chr3	40403676	40445114	+	.	.	.	.	.	.	.	.	.	.	.	.	.	.	.	.	.	.	.	.
7519	0,25	RPL14	NM_003973	chr3	40473786	40478867	+	.	.	.	.	.	.	.	.	.	.	.	.	.	.	.	.	.	.	.	.
57	6,75	CTNNB1	NM_001098209	chr3	41215945	41256943	+	.	.	.	.	.	.	.	.	.	.	.	.	.	.	.	.	.	.	.	.
5641	1	ULK4	NM_017886	chr3	41263093	41978664	-	CL800529	chr3	41953421	+	.	.	.	.	.	.	.	.	.	.	.	.	.	.	.	.
447	3,25	CCK	NM_000729	chr3	42274321	42282666	-	.	.	.	.	.	.	.	.	.	.	.	.	.	.	.	.	.	.	.	.
6587	0,5	LYZL4	NM_144634	chr3	42413578	42427069	-	.	.	.	.	.	.	.	.	.	.	.	.	.	.	.	.	.	.	.	.
5974	0,75	VIPR1	NM_001251884	chr3	42519107	42554069	+	.	.	.	.	.	.	.	.	.	.	.	.	.	.	.	.	.	.	.	.
6428	0,5	HHATL	NR_027753	chr3	42709158	42718017	-	.	.	.	.	.	.	.	.	.	.	.	.	.	.	.	.	.	.	.	.
2778	1,25	SNRK	NM_001100594	chr3	43303007	43367638	+	CL529725	chr3	43317609	+	.	.	.	.	.	.	.	.	.	.	.	.	.	.	.	.
2778	1,25	SNRK	NM_001100594	chr3	43303007	43367638	+	CL529241	chr3	43306687	+	.	.	.	.	.	.	.	.	.	.	.	.	.	.	.	.
7627	0,25	ZNF35	NM_003420	chr3	44665236	44677287	+	.	.	.	.	.	.	.	.	.	.	.	.	.	.	.	.	.	.	.	.

Table S2

tumor associated genes								HIV				MLV				MMTV				MMTV(SIN)				MMTV(SIN)arrest			
rank	score	gene symbol	RefSEq	chrom	txStart	txEnd	strand	integrant	chr	position	strand	integrant	chr	position	strand	integrant	chr	position	strand	integrant	chr	position	strand	integrant	chr	position	strand
6528	0,5	KIF15	NM_020242	chr3	44778212	44869752	+	.	.	.	.	.	.	.	.	.	.	.	.	.	.	.	.	.	.	.	.
3385	1	CLEC3B	NM_003278	chr3	45042762	45052567	+	.	.	.	.	.	.	.	.	.	.	.	.	.	.	.	.	.	.	.	.
5888	0,75	CDCP1	NM_022842	chr3	45098772	45162918	-	.	.	.	.	.	.	.	.	.	.	.	.	.	.	.	.	.	.	.	.
7592	0,25	TMEM158	NM_015444	chr3	45240959	45242818	-	.	.	.	.	.	.	.	.	.	.	.	.	.	.	.	.	.	.	.	.
4261	1	LARS2	NM_015340	chr3	45405078	45565332	+	.	.	.	.	.	.	.	.	.	.	.	.	.	.	.	.	.	.	.	.
7231	0,25	CCR9	NM_031200	chr3	45902999	45919671	+	.	.	.	.	.	.	.	.	.	.	.	.	.	.	.	.	.	.	.	.
448	3,25	CCR1	NM_001295	chr3	46218203	46224836	-	.	.	.	.	.	.	.	.	.	.	.	.	.	.	.	.	.	.	.	.
3292	1	CCR2	NM_001123041	chr3	46370238	46377417	+	.	.	.	.	.	.	.	.	.	.	.	.	.	.	.	.	.	.	.	.
1886	1,75	CCR5	NM_001100168	chr3	46386636	46392701	+	.	.	.	.	.	.	.	.	.	.	.	.	.	.	.	.	.	.	.	.
4316	1	LTF	NM_001199149	chr3	46452499	46480165	-	.	.	.	.	.	.	.	.	.	.	.	.	.	.	.	.	.	.	.	.
4305	1	LRRC2	NM_024512	chr3	46531881	46583044	-	.	.	.	.	.	.	.	.	.	.	.	.	.	.	.	.	.	.	.	.
7496	0,25	PTH1R	NM_001184744	chr3	46898742	46920293	+	.	.	.	.	.	.	.	.	.	.	.	.	.	.	.	.	.	.	.	.
5213	1	SETD2	NM_014159	chr3	47032901	47180471	-	CL799694	chr3	47067123	-	.	.	.	.	.	.	.	.	.	.	.	.	.	.	.	.
5213	1	SETD2	NM_014159	chr3	47032901	47180471	-	CL800569	chr3	47109222	-	.	.	.	.	.	.	.	.	.	.	.	.	.	.	.	.
4998	1	PTPN23	NM_015466	chr3	47397494	47429935	+	CL799796	chr3	47404054	+	.	.	.	.	.	.	.	.	.	.	.	.	.	.	.	.
6224	0,5	CSPG5	NM_006574	chr3	47578731	47595363	-	.	.	.	.	.	.	.	.	.	.	.	.	.	.	.	.	.	.	.	.
319	3,75	CDC25A	NM_201567	chr3	48173671	48204805	-	.	.	.	.	.	.	.	.	.	.	.	.	.	.	.	.	.	.	.	.
4591	1	NME6	NM_005793	chr3	48310592	48317852	-	.	.	.	.	.	.	.	.	.	.	.	.	.	.	.	.	.	.	.	.
5337	1	SPINK8	NM_001080525	chr3	48323339	48344835	-	.	.	.	.	.	.	.	.	.	.	.	.	.	.	.	.	.	.	.	.
5547	1	TREX1	NM_016381	chr3	48482232	48484048	+	.	.	.	.	.	.	.	.	.	.	.	.	.	.	.	.	.	.	.	.
4760	1	PFKFB4	NM_004567	chr3	48530120	48569231	-	.	.	.	.	.	.	.	.	.	.	.	.	.	.	.	.	.	.	.	.
3420	1	COL7A1	NM_000094	chr3	48576509	48607597	-	.	.	.	.	.	.	.	.	.	.	.	.	.	.	.	.	.	.	.	.
5647	1	UQCRC1	NM_003365	chr3	48611435	48622102	-	.	.	.	.	.	.	.	.	.	.	.	.	.	.	.	.	.	.	.	.
7554	0,25	SLC26A6	NM_134263	chr3	48638159	48647930	-	.	.	.	.	.	.	.	.	.	.	.	.	.	.	.	.	.	.	.	.
7241	0,25	CELSR3	NM_001407	chr3	48648899	48675352	-	.	.	.	.	.	.	.	.	.	.	.	.	.	.	.	.	.	.	.	.
1117	2,25	NCKIPSD	NM_184231	chr3	48686281	48698338	-	.	.	.	.	.	.	.	.	.	.	.	.	.	.	.	.	.	.	.	.
4155	1	IP6K2	NM_001190316	chr3	48705886	48729715	-	.	.	.	.	.	.	.	.	.	.	.	.	.	.	.	.	.	.	.	.
2724	1,25	PRKAR2A	NM_004157	chr3	48763096	48860274	-	.	.	.	.	.	.	.	.	.	.	.	.	.	.	.	.	.	.	.	.
1472	2	IMPDH2	NM_000884	chr3	49036765	49041879	-	.	.	.	.	.	.	.	.	.	.	.	.	.	.	.	.	.	.	.	.
5016	1	QARS	NM_005051	chr3	49108368	49117175	-	.	.	.	.	.	.	.	.	.	.	.	.	.	.	.	.	.	.	.	.
6546	0,5	LAMB2	NM_002292	chr3	49133550	49145603	-	.	.	.	.	.	.	.	.	.	.	.	.	.	.	.	.	.	.	.	.
2823	1,25	USP4	NM_003363	chr3	49289580	49352540	-	CL799893	chr3	49337719	+	.	.	.	.	.	.	.	.	.	.	.	.	.	.	.	.
3958	1	GPX1	NM_201397	chr3	49369612	49370795	-	.	.	.	.	.	.	.	.	.	.	.	.	.	.	.	.	.	.	.	.
299	4	RHOA	NM_001664	chr3	49371582	49424530	-	CL799772	chr3	49402003	-	.	.	.	.	.	.	.	.	.	.	.	.	.	.	.	.
7582	0,25	TCTA	NM_022171	chr3	49424642	49428913	+	.	.	.	.	.	.	.	.	.	.	.	.	.	.	.	.	.	.	.	.
2978	1	AMT	NM_001164711	chr3	49429214	49435115	-	.	.	.	.	.	.	.	.	.	.	.	.	.	.	.	.	.	.	.	.
6252	0,5	DAG1	NM_001177638	chr3	49482568	49548055	+	.	.	.	.	.	.	.	.	.	.	.	.	.	.	.	.	.	.	.	.
6068	0,5	BSN	NM_003458	chr3	49566925	49683986	+	.	.	.	.	.	.	.	.	.	.	.	.	.	.	.	.	.	.	.	.
1114	2,25	MST1	NM_020998	chr3	49696383	49701200	-	.	.	.	.	.	.	.	.	.	.	.	.	.	.	.	.	.	.	.	.
3911	1	GMPPB	NM_013334	chr3	49733935	49736388	-	.	.	.	.	.	.	.	.	.	.	.	.	.	.	.	.	.	.	.	.
7611	0,25	UBA7	NM_003335	chr3	49817641	49826395	-	.	.	.	.	.	.	.	.	.	.	.	.	.	.	.	.	.	.	.	.
5541	1	TRAIP	NM_005879	chr3	49841031	49868996	-	.	.	.	.	.	.	.	.	.	.	.	.	.	.	.	.	.	.	.	.
2050	1,5	CAMKV	NM_024046	chr3	49870425	49882373	-	.	.	.	.	.	.	.	.	.	.	.	.	.	.	.	.	.	.	.	.
280	4	MST1R	NM_001244937	chr3	49899439	49916310	-	.	.	.	.	.	.	.	.	.	.	.	.	.	.	.	.	.	.	.	.
7507	0,25	RBM6	NM_001167582	chr3	49952480	50089689	+	CL528884	chr3	50072626	+	.	.	.	.	.	.	.	.	.	.	.	.	.	.	.	.
7507	0,25	RBM6	NM_001167582	chr3	49952480	50089689	+	CL800375	chr3	50056718	+	.	.	.	.	.	.	.	.	.	.	.	.	.	.	.	.
7507	0,25	RBM6	NM_001167582	chr3	49952480	50089689	+	CL800557	chr3	49963466	+	.	.	.	.	.	.	.	.	.	.	.	.	.	.	.	.
7506	0,25	RBM5	NM_005778	chr3	50101344	50131401	+	CL800686	chr3	50121836	-	.	.	.	.	.	.	.	.	.	.	.	.	.	.	.	.
7506	0,25	RBM5	NM_005778	chr3	50101344	50131401	+	CL800600	chr3	50113942	+	.	.	.	.	.	.	.	.	.	.	.	.	.	.	.	.
2763	1,25	SEMA3F	NM_004186	chr3	50167851	50201512	+	.	.	.	.	.	.	.	.	.	.	.	.	.	.	.	.	.	.	.	.
3918	1	GNAT1	NM_000172	chr3	50204046	50210133	+	.	.	.	.	.	.	.	.	.	.	.	.	.	.	.	.	.	.	.	.

Table S2

tumor associated genes								HIV				MLV				MMTV				MMTV(SIN)				MMTV(SIN)arrest			
rank	score	gene symbol	RefSEq	chrom	txStart	txEnd	strand	integrant	chr	position	strand	integrant	chr	position	strand	integrant	chr	position	strand	integrant	chr	position	strand	integrant	chr	position	strand
2775	1,25	SLC38A3	NM_006841	chr3	50217695	50233410	+	.	.	.	.	.	.	.	.	.	.	.	.	.	.	.	.	.	.	.	.
3916	1	GNAI2	NM_001166425	chr3	50259329	50271790	+	CL800644	chr3	50269769	-	.	.	.	.	.	.	.	.	.	.	.	.	.	.	.	.
1962	1,75	SEMA3B	NM_004636	chr3	50280043	50289576	+	.	.	.	.	.	.	.	.	.	.	.	.	.	.	.	.	.	.	.	.
4499	1	NAT6	NM_001200018	chr3	50308836	50311324	-	.	.	.	.	.	.	.	.	.	.	.	.	.	.	.	.	.	.	.	.
2617	1,25	HYAL1	NM_153281	chr3	50312323	50324816	-	.	.	.	.	.	.	.	.	.	.	.	.	.	.	.	.	.	.	.	.
2618	1,25	HYAL2	NM_003773	chr3	50330224	50335285	-	.	.	.	.	.	.	.	.	.	.	.	.	.	.	.	.	.	.	.	.
2814	1,25	TUSC2	NM_007275	chr3	50337344	50340672	-	.	.	.	.	.	.	.	.	.	.	.	.	.	.	.	.	.	.	.	.
1155	2,25	RASSF1	NM_170712	chr3	50342220	50350668	-	.	.	.	.	.	.	.	.	.	.	.	.	.	.	.	.	.	.	.	.
1188	2,25	ZMYND10	NM_015896	chr3	50353540	50358160	-	.	.	.	.	.	.	.	.	.	.	.	.	.	.	.	.	.	.	.	.
7221	0,25	CACNA2D2	NM_001005505	chr3	50375234	50515896	-	.	.	.	.	.	.	.	.	.	.	.	.	.	.	.	.	.	.	.	.
4030	1	HEMK1	NM_016173	chr3	50581912	50597425	+	.	.	.	.	.	.	.	.	.	.	.	.	.	.	.	.	.	.	.	.
2242	1,5	MAPKAPK3	NM_001243926	chr3	50624296	50661732	+	.	.	.	.	.	.	.	.	.	.	.	.	.	.	.	.	.	.	.	.
3600	1	DOCK3	NM_004947	chr3	50687675	51396669	+	.	.	.	.	.	.	.	.	.	.	.	.	.	.	.	.	1085_129_1	chr3	50852065	-
6800	0,5	RBM15B	NM_013286	chr3	51403738	51410376	+	.	.	.	.	.	.	.	.	.	.	.	.	.	.	.	.	.	.	.	.
6791	0,5	RAD54L2	NM_015106	chr3	51550635	51672652	+	.	.	.	.	.	.	.	.	.	.	.	.	.	.	.	.	.	.	.	.
6401	0,5	GPR62	NM_080865	chr3	51964369	51966560	+	.	.	.	.	.	.	.	.	.	.	.	.	.	.	.	.	.	.	.	.
7465	0,25	PCBP4	NM_001174100	chr3	51966509	51976522	-	.	.	.	.	.	.	.	.	.	.	.	.	.	.	.	.	.	.	.	.
2490	1,25	ACY1	NM_001198895	chr3	51992339	51998258	+	.	.	.	.	.	.	.	.	.	.	.	.	.	.	.	.	.	.	.	.
3631	1	DUSP7	NM_001947	chr3	52057976	52065501	-	.	.	.	.	.	.	.	.	.	.	.	.	.	.	.	.	.	.	.	.
2944	1	ALAS1	NM_199166	chr3	52207138	52223383	+	.	.	.	.	.	.	.	.	.	.	.	.	.	.	.	.	.	.	.	.
1814	2	TLR9	NM_017442	chr3	52230135	52235219	-	.	.	.	.	.	.	.	.	.	.	.	.	.	.	.	.	.	.	.	.
7610	0,25	TWF2	NM_007284	chr3	52237665	52248223	-	.	.	.	.	.	.	.	.	.	.	.	.	.	.	.	.	.	.	.	.
4902	1	PPM1M	NM_001122870	chr3	52255225	52259655	+	.	.	.	.	.	.	.	.	.	.	.	.	.	.	.	.	.	.	.	.
3908	1	GLYCTK	NR_026700	chr3	52296875	52304312	+	.	.	.	.	.	.	.	.	.	.	.	.	.	.	.	.	.	.	.	.
7181	0,25	BAP1	NM_004656	chr3	52410064	52419049	-	.	.	.	.	.	.	.	.	.	.	.	.	.	.	.	.	.	.	.	.
2420	1,5	STAB1	NM_015136	chr3	52504395	52533551	+	.	.	.	.	.	.	.	.	.	.	.	.	.	.	.	.	.	.	.	.
7450	0,25	NT5DC2	NM_001134231	chr3	52533424	52542833	-	.	.	.	.	.	.	.	.	.	.	.	.	.	.	.	.	.	.	.	.
7464	0,25	PBRM1	NM_018313	chr3	52554407	52694906	-	BH609476	chr3	52616640	+	.	.	.	.	.	.	.	.	.	.	.	.	.	.	.	.
7464	0,25	PBRM1	NM_018313	chr3	52554407	52694906	-	CL800516	chr3	52676146	-	.	.	.	.	.	.	.	.	.	.	.	.	.	.	.	.
7464	0,25	PBRM1	NM_018313	chr3	52554407	52694906	-	CL799661	chr3	52651540	+	.	.	.	.	.	.	.	.	.	.	.	.	.	.	.	.
7464	0,25	PBRM1	NM_018313	chr3	52554407	52694906	-	CL800523	chr3	52627100	-	.	.	.	.	.	.	.	.	.	.	.	.	.	.	.	.
7464	0,25	PBRM1	NM_018313	chr3	52554407	52694906	-	CL799802	chr3	52657700	-	.	.	.	.	.	.	.	.	.	.	.	.	.	.	.	.
7464	0,25	PBRM1	NM_018313	chr3	52554407	52694906	-	AY516938.1	chr3	52641517	-	.	.	.	.	.	.	.	.	.	.	.	.	.	.	.	.
2603	1,25	GNL3	NM_206825	chr3	52694975	52703550	+	.	.	.	.	.	.	.	.	.	.	.	.	.	.	.	.	.	.	.	.
5327	1	SPCS1	NM_014041	chr3	52714896	52717237	+	.	.	.	.	.	.	.	.	.	.	.	.	.	.	.	.	.	.	.	.
4560	1	NEK4	NM_003157	chr3	52719835	52780005	-	.	.	.	.	.	.	.	.	.	.	.	.	.	.	.	.	.	.	.	.
5107	1	RFT1	NM_052859	chr3	53097540	53139510	-	.	.	.	.	.	.	.	.	.	.	.	.	.	.	.	.	.	.	.	.
420	3,5	PRKCD	NM_006254	chr3	53170262	53201773	+	.	.	.	.	.	.	.	.	.	.	.	.	.	.	.	.	.	.	.	.
5471	1	TKT	NM_001064	chr3	53234692	53265170	-	.	.	.	.	.	.	.	.	.	.	.	.	.	.	.	.	.	.	.	.
6256	0,5	DCP1A	NM_018403	chr3	53292484	53356694	-	.	.	.	.	.	.	.	.	.	.	.	.	.	.	.	.	.	.	.	.
3350	1	CHDH	NM_018397	chr3	53825363	53855460	-	.	.	.	.	.	.	.	.	.	.	.	.	.	.	.	.	.	.	.	.
4132	1	IL17RB	NM_018725	chr3	53855616	53874867	+	.	.	.	.	.	.	.	.	.	.	.	.	.	.	.	.	.	.	.	.
361	3,5	CACNA2D3	NM_018398	chr3	54131732	55083624	+	.	.	.	.	.	.	.	.	.	.	.	.	.	.	.	.	.	.	.	.
7100	0,5	WNT5A	NM_001256105	chr3	55474782	55490466	-	.	.	.	.	.	.	.	.	.	.	.	.	.	.	.	.	.	.	.	.
3695	1	ERC2	NM_015576	chr3	55517375	56477431	-	.	.	.	.	AY516096	chr3	55760762	-	.	.	.	.	.	.	.	.	2505_217_10	chr3	55700034	+
4133	1	IL17RD	NM_017563	chr3	57099049	57174443	-	.	.	.	.	.	.	.	.	.	.	.	.	.	.	.	.	.	.	.	.
3017	1	ARF4	NM_001660	chr3	57532129	57558255	-	.	.	.	.	.	.	.	.	.	.	.	.	.	.	.	.	.	.	.	.
1408	2	FLNB	NM_001457	chr3	57969166	58133022	+	BH609475	chr3	57978712	+	.	.	.	.	.	.	.	.	.	.	.	.	.	.	.	.
5011	1	PXK	NM_017771	chr3	58293656	58385918	+	.	.	.	.	AY515915	chr3	58345764	+	.	.	.	.	.	.	.	.	.	.	.	.
5011	1	PXK	NM_017771	chr3	58293656	58385918	+	.	.	.	.	AY516520	chr3	58296963	-	.	.	.	.	.	.	.	.	.	.	.	.
4743	1	PDHB	NM_001173468	chr3	58388396	58394619	-	.	.	.	.	.	.	.	.	.	.	.	.	.	.	.	.	.	.	.	.

Table S2

tumor associated genes								HIV				MLV				MMTV				MMTV(SIN)				MMTV(SIN)arrest			
rank	score	gene symbol	RefSEq	chrom	txStart	txEnd	strand	integrant	chr	position	strand	integrant	chr	position	strand	integrant	chr	position	strand	integrant	chr	position	strand	integrant	chr	position	strand
5983	0,5	ACOX2	NM_003500	chr3	58465902	58497969	-	.	.	.	.	.	.	.	.	.	.	.	.	.	.	.	.	.	.	.	.
6326	0,5	FAM107A	NM_007177	chr3	58524884	58538531	-	.	.	.	.	.	.	.	.	.	.	.	.	.	.	.	.	.	.	.	.
326	3,75	FHIT	NM_001166243	chr3	59710075	61212173	-	CL800041	chr3	60729653	+	.	.	.	.	.	.	.	.	2382_297	chr3	60703533	+	.	.	.	.
326	3,75	FHIT	NM_001166243	chr3	59710075	61212173	-	BH609473	chr3	60272159	-	.	.	.	.	.	.	.	.	2382_297	chr3	60703533	+	.	.	.	.
1151	2,25	PTPRG	NM_002841	chr3	61522282	62255613	+	.	.	.	.	.	.	.	.	.	.	.	.	.	.	.	.	.	.	.	.
3231	1	C3orf14	NM_020685	chr3	62280435	62294360	+	.	.	.	.	.	.	.	.	.	.	.	.	.	.	.	.	.	.	.	.
3770	1	FEZF2	NM_018008	chr3	62330386	62334230	-	.	.	.	.	.	.	.	.	.	.	.	.	.	.	.	.	.	.	.	.
6998	0,5	SYNPR	NM_001130003	chr3	63238953	63577637	+	.	.	.	.	.	.	.	.	.	.	.	.	.	.	.	.	.	.	.	.
2904	1	ADAMTS9	NM_182920	chr3	64476370	64648405	-	.	.	.	.	.	.	.	.	.	.	.	.	.	.	.	.	.	.	.	.
1514	2	MAGI1	NM_004742	chr3	65319983	65999549	-	.	.	.	.	.	.	.	.	.	.	.	.	.	.	.	.	.	.	.	.
7402	0,25	LRIG1	NM_015541	chr3	66511910	66633535	-	.	.	.	.	.	.	.	.	.	.	.	.	.	.	.	.	.	.	.	.
5400	1	SUCLG2	NM_001177599	chr3	67493573	67787728	-	CL529275	chr3	67708954	-	AY516326	chr3	67784812	-	.	.	.	.	.	.	.	.	.	.	.	.
3727	1	FAM19A1	NM_213609	chr3	68136048	68677461	+	.	.	.	.	.	.	.	.	.	.	.	.	.	.	.	.	.	.	.	.
6336	0,5	FAM19A4	NM_001005527	chr3	68863604	69064451	-	.	.	.	.	.	.	.	.	.	.	.	.	.	.	.	.	.	.	.	.
5501	1	TMF1	NM_007114	chr3	69151667	69184174	-	CL528886	chr3	69152936	+	.	.	.	.	.	.	.	.	.	.	.	.	.	.	.	.
1532	2	MITF	NM_198178	chr3	70068440	70100178	+	CL799640	chr3	69942751	-	AY516863	chr3	69901940	-	.	.	.	.	.	.	.	.	.	.	.	.
3797	1	FOXP1	NM_001012505	chr3	71329723	71715830	-	CL800145	chr3	71277530	+	.	.	.	.	.	.	.	.	.	.	.	.	.	.	.	.
3797	1	FOXP1	NM_001012505	chr3	71329723	71715830	-	BH609472	chr3	71491114	-	.	.	.	.	.	.	.	.	.	.	.	.	.	.	.	.
3797	1	FOXP1	NM_001012505	chr3	71329723	71715830	-	CL799806	chr3	71337713	+	.	.	.	.	.	.	.	.	.	.	.	.	.	.	.	.
4926	1	PPP4R2	NM_174907	chr3	73128808	73197701	+	CL529276	chr3	73159692	+	.	.	.	.	.	.	.	.	.	.	.	.	.	.	.	.
1317	2	CNTN3	NM_020872	chr3	74394411	74653033	-	.	.	.	.	.	.	.	.	.	.	.	.	.	.	.	.	.	.	.	.
669	3	ROBO2	NM_001128929	chr3	77229852	77781804	+	.	.	.	.	.	.	.	.	.	.	.	.	.	.	.	.	.	.	.	.
501	3,25	ROBO1	NM_001145845	chr3	78729077	79151299	-	CL528887	chr3	78871386	+	.	.	.	.	_335_1_206	chr3	79512511	+	.	.	.	.	.	.	.	.
3862	1	GBE1	NM_000158	chr3	81621539	81893640	-	CL799594	chr3	81877772	-	.	.	.	.	.	.	.	.	.	.	.	.	.	.	.	.
135	5	EPHA3	NM_182644	chr3	89239363	89532185	+	.	.	.	.	.	.	.	.	.	.	.	.	.	.	.	.	.	.	.	.
2725	1,25	PROS1	NM_000313	chr3	95074570	95175624	-	.	.	.	.	.	.	.	.	.	.	.	.	.	.	.	.	.	.	.	.
827	2,5	EPHA6	NM_173655	chr3	98641179	98850111	+	.	.	.	.	.	.	.	.	.	.	.	.	.	.	.	.	.	.	.	.
7422	0,25	MINA	NM_001261829	chr3	99143350	99173985	-	.	.	.	.	.	.	.	.	.	.	.	.	.	.	.	.	.	.	.	.
3450	1	CPOX	NM_000097	chr3	99780979	99795145	-	AY517177.1	chr3	99793017	-	.	.	.	.	.	.	.	.	.	.	.	.	.	.	.	.
5361	1	ST3GAL6	NM_006100	chr3	99934261	99995926	+	.	.	.	.	.	.	.	.	.	.	.	.	.	.	.	.	.	.	.	.
3533	1	DCBLD2	NM_080927	chr3	99997503	100103223	-	.	.	.	.	.	.	.	.	.	.	.	.	.	.	.	.	.	.	.	.
7035	0,5	TMEM45A	NM_018004	chr3	101694152	101778975	+	.	.	.	.	.	.	.	.	_262_1_178	chr3	101730178	+	.	.	.	.	.	.	.	.
690	3	TFG	NM_001007565	chr3	101910823	101950501	+	.	.	.	.	.	.	.	.	.	.	.	.	.	.	.	.	.	.	.	.
7148	0,25	ABI3BP	NM_015429	chr3	101950868	102195024	-	.	.	.	.	.	.	.	.	.	.	.	.	.	.	.	.	.	.	.	.
709	2,75	ALCAM	NM_001243283	chr3	106568246	106726920	+	.	.	.	.	.	.	.	.	.	.	.	.	.	.	.	.	.	.	.	.
2054	1,5	CBLB	NM_170662	chr3	106859798	107070577	-	AY517375.1	chr3	106896468	-	.	.	.	.	.	.	.	.	.	.	.	.	.	.	.	.
2054	1,5	CBLB	NM_170662	chr3	106859798	107070577	-	CL529618	chr3	107048745	+	.	.	.	.	.	.	.	.	.	.	.	.	.	.	.	.
2059	1,5	CD47	NM_198793	chr3	109244630	109292625	-	.	.	.	.	.	.	.	.	.	.	.	.	.	.	.	.	.	.	.	.
3638	1	DZIP3	NM_014648	chr3	109791026	109896383	+	.	.	.	.	.	.	.	.	.	.	.	.	.	.	.	.	.	.	.	.
5543	1	TRAT1	NM_016388	chr3	110024320	110056404	+	.	.	.	.	.	.	.	.	.	.	.	.	.	.	.	.	.	.	.	.
7007	0,5	TAGLN3	NM_001008273	chr3	113200696	113215425	+	.	.	.	.	.	.	.	.	.	.	.	.	.	.	.	.	.	.	.	.
3287	1	CCDC80	NM_199512	chr3	113806098	113842667	-	.	.	.	.	.	.	.	.	.	.	.	.	.	.	.	.	.	.	.	.
2012	1,5	ATP6V1A	NM_001690	chr3	114948555	115013595	+	.	.	.	.	.	.	.	.	.	.	.	.	.	.	.	.	.	.	.	.
5739	1	ZDHHC23	NM_173570	chr3	115149437	115164517	+	.	.	.	.	.	.	.	.	.	.	.	.	.	.	.	.	.	.	.	.
6784	0,5	QTRTD1	NM_001256837	chr3	115258271	115289958	+	.	.	.	.	.	.	.	.	.	.	.	.	.	.	.	.	.	.	.	.
3120	1	B4GALT4	NM_212543	chr3	120413278	120442442	-	CL529440	chr3	120433930	+	.	.	.	.	.	.	.	.	.	.	.	.	.	.	.	.
3120	1	B4GALT4	NM_212543	chr3	120413278	120442442	-	CL799759	chr3	120439945	+	.	.	.	.	.	.	.	.	.	.	.	.	.	.	.	.
2540	1,25	CD80	NM_005191	chr3	120725829	120761171	-	.	.	.	.	.	.	.	.	.	.	.	.	.	.	.	.	.	.	.	.
4824	1	PLA1A	NM_001206961	chr3	120799384	120831348	+	.	.	.	.	.	.	.	.	.	.	.	.	.	.	.	.	.	.	.	.
2553	1,25	COX17	NM_005694	chr3	120871061	120878933	-	.	.	.	.	.	.	.	.	.	.	.	.	.	.	.	.	.	.	.	.
4608	1	NR1I2	NM_033013	chr3	120982020	121020022	+	.	.	.	.	.	.	.	.	.	.	.	.	.	.	.	.	.	.	.	.

Table S2

tumor associated genes								HIV				MLV				MMTV				MMTV(SIN)				MMTV(SIN)arrest			
rank	score	gene symbol	RefSEq	chrom	txStart	txEnd	strand	integrant	chr	position	strand	integrant	chr	position	strand	integrant	chr	position	strand	integrant	chr	position	strand	integrant	chr	position	strand
181	4,5	GSK3B	NM_002093	chr3	121023491	121295954	-	AY516910.1	chr3	121213110	-	.	.	.	.	.	.	.	.	.	.	.	.	.	.	.	.
181	4,5	GSK3B	NM_002093	chr3	121023491	121295954	-	BH609486	chr3	121106316	-	.	.	.	.	.	.	.	.	.	.	.	.	.	.	.	.
4539	1	NDUFB4	NM_001168331	chr3	121797817	121803948	+	.	.	.	.	.	.	.	.	.	.	.	.	.	.	.	.	.	.	.	.
4037	1	HGD	NM_000187	chr3	121829704	121884108	-	.	.	.	.	.	.	.	.	.	.	.	.	.	.	.	.	.	.	.	.
4867	1	POLQ	NM_199420	chr3	122632962	122747543	-	.	.	.	.	.	.	.	.	.	.	.	.	.	.	.	.	.	.	.	.
6349	0,5	FBXO40	NM_016298	chr3	122794859	122831829	+	.	.	.	.	.	.	.	.	.	.	.	.	.	.	.	.	.	.	.	.
2181	1,5	HCLS1	NM_005335	chr3	122832935	122862481	-	.	.	.	.	.	.	.	.	.	.	.	.	.	.	.	.	.	.	.	.
2061	1,5	CD86	NM_001206924	chr3	123256898	123322678	+	.	.	.	.	.	.	.	.	.	.	.	.	.	.	.	.	.	.	.	.
1019	2,25	CSTA	NM_005213	chr3	123526700	123543505	+	.	.	.	.	.	.	.	.	.	.	.	.	.	.	.	.	.	.	.	.
1366	2	DTX3L	NM_138287	chr3	123765874	123776739	+	.	.	.	.	.	.	.	.	.	.	.	.	.	.	.	.	.	.	.	.
5208	1	SEMA5B	NM_001256347	chr3	124110729	124177671	-	.	.	.	.	.	.	.	.	.	.	.	.	.	.	.	.	.	.	.	.
1219	2	ADCY5	NM_183357	chr3	124483832	124650082	-	.	.	.	.	.	.	.	.	.	.	.	.	.	.	.	.	.	.	.	.
4992	1	PTPLB	NM_198402	chr3	124696052	124786614	-	.	.	.	.	.	.	.	.	.	.	.	.	.	.	.	.	.	.	.	.
4474	1	MYLK	NM_053028	chr3	124813832	125085839	-	.	.	.	.	.	.	.	.	.	.	.	.	.	.	.	.	.	.	.	.
269	4	KALRN	NR_028136	chr3	125296247	125366223	+	.	.	.	.	.	.	.	.	.	.	.	.	107_1_829_28	chr3	125681189	+	.	.	.	.
1841	2	UMPS	NM_000373	chr3	125931902	125950809	+	.	.	.	.	.	.	.	.	.	.	.	.	.	.	.	.	.	.	.	.
7126	0,5	ZNF148	NM_021964	chr3	126427202	126576888	-	AY516918.1	chr3	126519074	-	.	.	.	.	.	.	.	.	.	.	.	.	.	.	.	.
7126	0,5	ZNF148	NM_021964	chr3	126427202	126576888	-	CL800113	chr3	126478953	-	.	.	.	.	.	.	.	.	.	.	.	.	.	.	.	.
6950	0,5	SNX4	NM_003794	chr3	126648183	126721748	-	BH609487	chr3	126698643	-	.	.	.	.	.	.	.	.	.	.	.	.	.	.	.	.
6950	0,5	SNX4	NM_003794	chr3	126648183	126721748	-	BH609489	chr3	126649167	-	.	.	.	.	.	.	.	.	.	.	.	.	.	.	.	.
6950	0,5	SNX4	NM_003794	chr3	126648183	126721748	-	BH609488	chr3	126692280	-	.	.	.	.	.	.	.	.	.	.	.	.	.	.	.	.
6950	0,5	SNX4	NM_003794	chr3	126648183	126721748	-	CL529564	chr3	126689285	-	.	.	.	.	.	.	.	.	.	.	.	.	.	.	.	.
1993	1,5	ALDH1L1	NR_072979	chr3	127305093	127382335	-	.	.	.	.	.	.	.	.	.	.	.	.	.	.	.	.	.	.	.	.
5650	1	UROC1	NM_001165974	chr3	127682697	127719306	-	.	.	.	.	.	.	.	.	.	.	.	.	.	.	.	.	.	.	.	.
3362	1	CHST13	NM_152889	chr3	127725820	127744824	+	.	.	.	.	.	.	.	.	.	.	.	.	.	.	.	.	.	.	.	.
6756	0,5	PLXNA1	NM_032242	chr3	128190126	128238925	+	.	.	.	.	.	.	.	.	.	.	.	.	.	.	.	.	.	.	.	.
1937	1,75	MCM2	NM_004526	chr3	128799942	128823968	+	.	.	.	.	.	.	.	.	.	.	.	.	.	.	.	.	.	.	.	.
6761	0,5	PODXL2	NM_015720	chr3	128830728	128874342	+	.	.	.	.	.	.	.	.	.	.	.	.	.	.	.	.	.	.	.	.
2863	1	ABTB1	NM_172027	chr3	128874470	128882459	+	.	.	.	.	.	.	.	.	.	.	.	.	.	.	.	.	.	.	.	.
2254	1,5	MGLL	NM_001256585	chr3	128890594	129024415	-	.	.	.	.	.	.	.	.	.	.	.	.	.	.	.	.	.	.	.	.
5206	1	SEC61A1	NM_013336	chr3	129253901	129273216	+	.	.	.	.	.	.	.	.	.	.	.	.	.	.	.	.	.	.	.	.
6840	0,5	RUVBL1	NM_003707	chr3	129282489	129325361	-	CL800362	chr3	129305243	+	.	.	.	.	.	.	.	.	.	.	.	.	.	.	.	.
3649	1	EEFSEC	NM_021937	chr3	129355002	129610179	+	CL528891	chr3	129436545	-	.	.	.	.	.	.	.	.	.	.	.	.	.	.	.	.
2598	1,25	GATA2	NM_032638	chr3	129680954	129694720	-	.	.	.	.	.	.	.	.	.	.	.	.	.	.	.	.	.	.	.	.
424	3,5	RPN1	NM_002950	chr3	129821502	129852409	-	.	.	.	.	.	.	.	.	.	.	.	.	.	.	.	.	.	.	.	.
5043	1	RAB7A	NM_004637	chr3	129927668	130016331	+	.	.	.	.	.	.	.	.	.	.	.	.	.	.	.	.	.	.	.	.
2869	1	ACAD9	NR_033426	chr3	130081022	130114647	+	AY517139.1	chr3	130088851	+	.	.	.	.	.	.	.	.	.	.	.	.	.	.	.	.
1316	2	CNBP	NM_001127192	chr3	130369347	130385500	-	BH609491	chr3	130376262	-	.	.	.	.	.	.	.	.	.	.	.	.	.	.	.	.
4364	1	MBD4	NM_003925	chr3	130632482	130641542	-	CL799912	chr3	130636773	+	.	.	.	.	.	.	.	.	.	.	.	.	.	.	.	.
7027	0,5	TMCC1	NM_001017395	chr3	130849324	131082092	-	CL799836	chr3	131051404	-	AY516861	chr3	130858204	+	.	.	.	.	.	.	.	.	.	.	.	.
7601	0,25	TRH	NM_007117	chr3	131175803	131179469	+	.	.	.	.	.	.	.	.	.	.	.	.	.	.	.	.	.	.	.	.
1131	2,25	PIK3R4	NM_014602	chr3	131880467	131948386	-	.	.	.	.	.	.	.	.	.	.	.	.	.	.	.	.	.	.	.	.
2692	1,25	NEK11	NM_001146003	chr3	132228383	132551999	+	CL528892	chr3	132289555	-	.	.	.	.	.	.	.	.	.	.	.	.	.	.	.	.
3448	1	CPNE4	NM_130808	chr3	132736266	133236534	-	AY517010.1	chr3	133019926	+	.	.	.	.	.	.	.	.	.	.	.	.	.	.	.	.
771	2,5	ACPP	NM_001099	chr3	133518900	133560380	+	.	.	.	.	.	.	.	.	.	.	.	.	.	.	.	.	.	.	.	.
6274	0,5	DNAJC13	NM_015268	chr3	133619242	133740566	+	CL799967	chr3	133662346	+	AY516679	chr3	133688450	-	.	.	.	.	.	.	.	.	.	.	.	.
6165	0,5	CDV3	NM_017548	chr3	134775123	134791808	+	.	.	.	.	.	.	.	.	.	.	.	.	.	.	.	.	.	.	.	.
436	3,5	TOPBP1	NM_007027	chr3	134802138	134863427	-	.	.	.	.	.	.	.	.	.	.	.	.	.	.	.	.	.	.	.	.
5461	1	TF	NM_001063	chr3	134947666	134980540	+	.	.	.	.	.	.	.	.	.	.	.	.	.	.	.	.	.	.	.	.
5041	1	RAB6B	NM_016577	chr3	135025769	135097381	-	.	.	.	.	.	.	.	.	.	.	.	.	.	.	.	.	.	.	.	.
1160	2,25	RYK	NM_001005861	chr3	135358667	135452276	-	AY517007.1	chr3	135390062	+	.	.	.	.	.	.	.	.	.	.	.	.	.	.	.	.

Table S2

tumor associated genes								HIV				MLV				MMTV				MMTV(SIN)				MMTV(SIN)arrest			
rank	score	gene symbol	RefSEq	chrom	txStart	txEnd	strand	integrant	chr	position	strand	integrant	chr	position	strand	integrant	chr	position	strand	integrant	chr	position	strand	integrant	chr	position	strand
2975	1	AMOTL2	NM_016201	chr3	135556879	135576096	-	.	.	.	.	.	.	.	.	.	.	.	.	.	.	.	.	.	.	.	.
2500	1,25	ANAPC13	NM_001242374	chr3	135679235	135687555	-	.	.	.	.	.	.	.	.	.	.	.	.	.	.	.	.	.	.	.	.
7242	0,25	CEP63	NM_001042384	chr3	135687264	135776545	+	CL528894	chr3	135691466	+	.	.	.	.	.	.	.	.	.	.	.	.	.	.	.	.
104	5,5	EPHB1	NM_004441	chr3	135996788	136461997	+	.	.	.	.	.	.	.	.	.	.	.	.	23_3_2594_14	chr3	136141247	-	.	.	.	.
1672	2	PPP2R3A	NM_181897	chr3	137224266	137349442	+	.	.	.	.	.	.	.	.	.	.	.	.	.	.	.	.	.	.	.	.
6631	0,5	MSL2	NM_018133	chr3	137350449	137397378	-	.	.	.	.	.	.	.	.	.	.	.	.	.	.	.	.	.	.	.	.
4704	1	PCCB	NM_000532	chr3	137451856	137531703	+	AY517239.1	chr3	137510662	+	.	.	.	.	.	.	.	.	.	.	.	.	.	.	.	.
5369	1	STAG1	NM_005862	chr3	137538688	137953935	-	CL800286	chr3	137849054	+	.	.	.	.	.	.	.	.	.	.	.	.	.	.	.	.
5369	1	STAG1	NM_005862	chr3	137538688	137953935	-	CL799990	chr3	137783202	-	.	.	.	.	.	.	.	.	.	.	.	.	.	.	.	.
1116	2,25	NCK1	NM_001190796	chr3	138132006	138150658	+	.	.	.	.	.	.	.	.	.	.	.	.	.	.	.	.	.	.	.	.
2263	1,5	MRAS	NM_012219	chr3	139549179	139607067	+	.	.	.	.	.	.	.	.	.	.	.	.	.	.	.	.	.	.	.	.
30	8,5	PIK3CB	NM_006219	chr3	139854229	139960891	-	.	.	.	.	.	.	.	.	.	.	.	.	.	.	.	.	.	.	.	.
3796	1	FOXL2	NM_023067	chr3	140145755	140148672	-	.	.	.	.	.	.	.	.	.	.	.	.	.	.	.	.	.	.	.	.
1959	1,75	RBP1	NM_001130993	chr3	140727316	140741361	-	.	.	.	.	.	.	.	.	.	.	.	.	.	.	.	.	.	.	.	.
4593	1	NMNAT3	NM_178177	chr3	140761712	140879575	-	CL800171	chr3	140875982	-	.	.	.	.	.	.	.	.	.	.	.	.	.	.	.	.
3397	1	CLSTN2	NM_022131	chr3	141136716	141769609	+	.	.	.	.	.	.	.	.	.	.	.	.	.	.	.	.	.	.	.	.
1820	2	TRIM42	NM_152616	chr3	141879555	141902682	+	.	.	.	.	.	.	.	.	.	.	.	.	.	.	.	.	.	.	.	.
7517	0,25	RNF7	NR_037702	chr3	142939740	142948335	+	.	.	.	.	.	.	.	.	.	.	.	.	.	.	.	.	.	.	.	.
2175	1,5	GRK7	NM_139209	chr3	142979732	143018582	+	.	.	.	.	.	.	.	.	.	.	.	.	.	.	.	.	.	.	.	.
5462	1	TFDP2	NM_006286	chr3	143145959	143230197	-	.	.	.	.	.	.	.	.	.	.	.	.	.	.	.	.	.	.	.	.
3895	1	GK5	NM_001039547	chr3	143359058	143427139	-	CL529457	chr3	143420501	+	.	.	.	.	.	.	.	.	.	.	.	.	.	.	.	.
236	4	ATR	NM_001184	chr3	143650766	143780358	-	CL799782	chr3	143683883	-	.	.	.	.	.	.	.	.	.	.	.	.	.	.	.	.
4719	1	PCOLCE2	NM_013363	chr3	144019391	144090735	-	.	.	.	.	.	.	.	.	.	.	.	.	.	.	.	.	.	.	.	.
3363	1	CHST2	NM_004267	chr3	144321307	144325546	+	.	.	.	.	.	.	.	.	.	.	.	.	.	.	.	.	.	.	.	.
4846	1	PLOD2	NM_182943	chr3	147269917	147361972	-	.	.	.	.	.	.	.	.	.	.	.	.	.	.	.	.	.	.	.	.
920	2,5	PLSCR1	NM_021105	chr3	147715656	147745318	-	.	.	.	.	.	.	.	.	.	.	.	.	.	.	.	.	.	.	.	.
7622	0,25	ZIC4	NR_033118	chr3	148586524	148592907	-	.	.	.	.	.	.	.	.	.	.	.	.	.	.	.	.	.	.	.	.
5748	1	ZIC1	NM_003412	chr3	148609870	148617196	+	.	.	.	.	.	.	.	.	.	.	.	.	.	.	.	.	.	.	.	.
6000	0,5	AGTR1	NM_031850	chr3	149898347	149943480	+	.	.	.	.	.	.	.	.	.	.	.	.	.	.	.	.	.	.	.	.
7350	0,25	HLTF	NM_003071	chr3	150230593	150287031	-	CL528897	chr3	150250051	-	.	.	.	.	.	.	.	.	.	.	.	.	.	.	.	.
1897	1,75	CP	NM_000096	chr3	150372979	150422522	-	.	.	.	.	.	.	.	.	.	.	.	.	.	.	.	.	.	.	.	.
7586	0,25	TM4SF1	NM_014220	chr3	150569494	150578258	-	.	.	.	.	.	.	.	.	.	.	.	.	.	.	.	.	.	.	.	.
7103	0,5	WWTR1	NM_001168278	chr3	150717711	150903750	-	.	.	.	.	.	.	.	.	.	.	.	.	.	.	.	.	.	.	.	.
6734	0,5	PFN2	NM_002628	chr3	151165380	151171431	-	.	.	.	.	.	.	.	.	.	.	.	.	.	.	.	.	.	.	.	.
5240	1	SIAH2	NM_005067	chr3	151941599	151963953	-	.	.	.	.	.	.	.	.	.	.	.	.	.	.	.	.	.	.	.	.
2841	1	AADAC	NM_001086	chr3	153014550	153028966	+	CL799709	chr3	153025148	+	.	.	.	.	.	.	.	.	.	.	.	.	.	.	.	.
1938	1,75	MME	NM_000902	chr3	156280129	156384212	+	.	.	.	.	.	.	.	.	.	.	.	.	.	.	.	.	.	.	.	.
5271	1	SLC33A1	NM_004733	chr3	157026994	157054942	-	.	.	.	.	.	.	.	.	.	.	.	.	.	.	.	.	.	.	.	.
260	4	GMPS	NM_003875	chr3	157071018	157138214	+	.	.	.	.	.	.	.	.	.	.	.	.	.	.	.	.	.	.	.	.
6491	0,5	KCNAB1	NM_172159	chr3	157491469	157739621	+	.	.	.	.	.	.	.	.	.	.	.	.	.	.	.	.	.	.	.	.
1957	1,75	PTX3	NM_002852	chr3	158637273	158644111	+	.	.	.	.	.	.	.	.	.	.	.	.	.	.	.	.	.	.	.	.
5673	1	VEPH1	NM_001167916	chr3	158660103	158704109	-	.	.	.	.	.	.	.	.	.	.	.	.	.	.	.	.	.	.	.	.
956	2,5	SHOX2	NM_003030	chr3	159296493	159306646	-	.	.	.	.	.	.	.	.	.	.	.	.	.	.	.	.	.	.	.	.
1534	2	MLF1	NM_001130156	chr3	159771646	159806943	+	.	.	.	.	.	.	.	.	.	.	.	.	.	.	.	.	.	.	.	.
7499	0,25	RARRES1	NM_206963	chr3	159897590	159932969	-	.	.	.	.	.	.	.	.	.	.	.	.	.	.	.	.	.	.	.	.
6609	0,5	MFSD1	NM_022736	chr3	160002408	160030202	+	.	.	.	.	.	.	.	.	.	.	.	.	.	.	.	.	.	.	.	.
6852	0,5	SCHIP1	NM_001197107	chr3	160473729	161097849	+	.	.	.	.	.	.	.	.	.	.	.	.	.	.	.	.	.	.	.	.
4128	1	IL12A	NM_000882	chr3	161189316	161196500	+	.	.	.	.	.	.	.	.	.	.	.	.	.	.	.	.	.	.	.	.
6937	0,5	SMC4	NM_005496	chr3	161601040	161635435	+	AY516996.1	chr3	161605563	+	.	.	.	.	.	.	.	.	.	.	.	.	.	.	.	.
4901	1	PPM1L	NM_139245	chr3	161956689	162271511	+	CL529283	chr3	161992175	+	.	.	.	.	.	.	.	.	.	.	.	.	2581_224_1	chr3	162115520	+
3105	1	B3GALNT1	NM_003781	chr3	162284364	162305854	-	.	.	.	.	.	.	.	.	.	.	.	.	.	.	.	.	.	.	.	.

Table S2

tumor associated genes								HIV				MLV				MMTV				MMTV(SIN)				MMTV(SIN)arrest			
rank	score	gene symbol	RefSEq	chrom	txStart	txEnd	strand	integrant	chr	position	strand	integrant	chr	position	strand	integrant	chr	position	strand	integrant	chr	position	strand	integrant	chr	position	strand
5238	1	SI	NM_001041	chr3	166179379	166278977	-	.	.	.	.	.	.	.	.	.	.	.	.	.	.	.	.	.	.	.	.
5290	1	SLITRK3	NM_014926	chr3	166387201	166397163	-	.	.	.	.	.	.	.	.	.	.	.	.	.	.	.	.	.	.	.	.
1877	1,75	BCHE	NM_000055	chr3	166973385	167037947	-	.	.	.	.	.	.	.	.	.	.	.	.	.	.	.	.	.	.	.	.
2393	1,5	SERPINI1	NM_005025	chr3	168936204	169026051	+	.	.	.	.	.	.	.	.	.	.	.	.	.	.	.	.	.	.	.	.
2795	1,25	TERC	NR_001566	chr3	170965091	170965542	-	.	.	.	.	.	.	.	.	.	.	.	.	.	.	.	.	.	.	.	.
3951	1	GPR160	NM_014373	chr3	171238428	171285877	+	.	.	.	.	.	.	.	.	.	.	.	.	.	.	.	.	.	.	.	.
496	3,25	PRKCI	NM_002740	chr3	171422913	171506464	+	.	.	.	.	.	.	.	.	.	.	.	.	.	.	.	.	.	.	.	.
2402	1,5	SKIL	NM_001248008	chr3	171560104	171597331	+	.	.	.	.	.	.	.	.	.	.	.	.	.	.	.	.	435_104_1	chr3	171594141	+
3383	1	CLDN11	NM_005602	chr3	171619346	171635173	+	.	.	.	.	.	.	.	.	.	.	.	.	.	.	.	.	.	.	.	.
6927	0,5	SLC7A14	NM_020949	chr3	171660035	171786557	-	.	.	.	.	.	.	.	.	.	.	.	.	.	.	.	.	.	.	.	.
3672	1	EIF5A2	NM_020390	chr3	172088897	172109120	-	.	.	.	.	.	.	.	.	.	.	.	.	.	.	.	.	.	.	.	.
5963	0,75	SLC2A2	NM_000340	chr3	172196830	172227462	-	.	.	.	.	.	.	.	.	.	.	.	.	.	.	.	.	.	.	.	.
5514	1	TNIK	NM_001161560	chr3	172262985	172660891	-	AY517191.1	chr3	172309420	-	.	.	.	.	.	.	.	.	.	.	.	.	.	.	.	.
1636	2	PLD1	NM_002662	chr3	172800888	173010978	-	.	.	.	.	.	.	.	.	.	.	.	.	.	.	.	.	.	.	.	.
3791	1	FNDC3B	NM_001135095	chr3	173241037	173601186	+	.	.	.	.	.	.	.	.	.	.	.	.	.	.	.	.	.	.	.	.
3888	1	GHSR	NM_004122	chr3	173648026	173648940	-	.	.	.	.	.	.	.	.	.	.	.	.	.	.	.	.	.	.	.	.
1177	2,25	TNFSF10	NR_033994	chr3	173705991	173723991	-	.	.	.	.	.	.	.	.	.	.	.	.	.	.	.	.	.	.	.	.
6296	0,5	ECT2	NM_001258315	chr3	173951168	174021958	+	CL529431	chr3	173974026	+	.	.	.	.	.	.	.	.	.	.	.	.	.	.	.	.
4583	1	NLGN1	NM_014932	chr3	174598937	175483810	+	.	.	.	.	.	.	.	.	.	.	.	.	.	.	.	.	.	.	.	.
7623	0,25	ZMAT3	NM_022470	chr3	180217706	180272350	-	.	.	.	.	.	.	.	.	.	.	.	.	.	.	.	.	.	.	.	.
5	13,75	PIK3CA	NM_006218	chr3	180349004	180435191	+	CL529749	chr3	180410411	+	.	.	.	.	.	.	.	.	.	.	.	.	.	.	.	.
4387	1	MFN1	NM_033540	chr3	180548173	180593702	+	.	.	.	.	.	.	.	.	.	.	.	.	.	.	.	.	.	.	.	.
5985	0,5	ACTL6A	NM_178042	chr3	180763401	180788887	+	.	.	.	.	.	.	.	.	.	.	.	.	.	.	.	.	.	.	.	.
4540	1	NDUFB5	NM_002492	chr3	180805268	180824982	+	.	.	.	.	.	.	.	.	.	.	.	.	.	.	.	.	.	.	.	.
6733	0,5	PEX5L	NM_001256751	chr3	180995440	181237535	-	.	.	.	.	.	.	.	.	.	.	.	.	91_1_2391_11	chr3	181228170	+	.	.	.	.
5318	1	SOX2	NM_003106	chr3	182912405	182914917	+	.	.	.	.	.	.	.	.	.	.	.	.	.	.	.	.	.	.	.	.
7270	0,25	DCUN1D1	NM_020640	chr3	184143252	184181020	-	.	.	.	.	.	.	.	.	.	.	.	.	.	.	.	.	.	.	.	.
4368	1	MCCC1	NM_020166	chr3	184215699	184300059	-	CL528899	chr3	184269870	+	.	.	.	.	.	.	.	.	.	.	.	.	.	.	.	.
7393	0,25	LAMP3	NM_014398	chr3	184322696	184363361	-	.	.	.	.	.	.	.	.	.	.	.	.	.	.	.	.	.	.	.	.
3114	1	B3GNT5	NM_032047	chr3	184453725	184473873	+	.	.	.	.	.	.	.	.	.	.	.	.	.	.	.	.	.	.	.	.
6593	0,5	MAP6D1	NM_024871	chr3	185016357	185026087	-	.	.	.	.	.	.	.	.	.	.	.	.	.	.	.	.	.	.	.	.
2485	1,25	ABCC5	NM_001023587	chr3	185184234	185218421	-	.	.	.	.	.	.	.	.	.	.	.	.	.	.	.	.	.	.	.	.
3666	1	EIF2B5	NM_003907	chr3	185335503	185345793	+	.	.	.	.	.	.	.	.	.	.	.	.	.	.	.	.	.	.	.	.
2574	1,25	DVL3	NM_004423	chr3	185355977	185374008	+	CL528900	chr3	185366435	-	.	.	.	.	.	.	.	.	.	.	.	.	.	.	.	.
2960	1	ALG3	NR_024533	chr3	185442810	185449453	-	.	.	.	.	.	.	.	.	.	.	.	.	.	.	.	.	.	.	.	.
3272	1	CAMK2N2	NM_033259	chr3	185459696	185461945	-	.	.	.	.	.	.	.	.	.	.	.	.	.	.	.	.	.	.	.	.
6294	0,5	ECE2	NM_001100120	chr3	185476492	185493513	+	.	.	.	.	.	.	.	.	.	.	.	.	.	.	.	.	.	.	.	.
3670	1	EIF4G1	NM_198244	chr3	185514976	185535840	+	.	.	.	.	.	.	.	.	.	.	.	.	.	.	.	.	.	.	.	.
6333	0,5	FAM131A	NM_001171093	chr3	185536410	185546757	+	.	.	.	.	.	.	.	.	.	.	.	.	.	.	.	.	.	.	.	.
7247	0,25	CLCN2	NM_001171087	chr3	185546666	185562133	-	.	.	.	.	.	.	.	.	.	.	.	.	.	.	.	.	.	.	.	.
925	2,5	POLR2H	NM_006232	chr3	185563887	185569057	+	.	.	.	.	.	.	.	.	.	.	.	.	.	.	.	.	.	.	.	.
6176	0,5	CHRD	NM_003741	chr3	185580554	185590311	+	.	.	.	.	.	.	.	.	.	.	.	.	.	.	.	.	.	.	.	.
724	2,75	EPHB3	NM_004443	chr3	185762280	185782890	+	.	.	.	.	.	.	.	.	.	.	.	.	.	.	.	.	.	.	.	.
3662	1	EHHADH	NM_001166415	chr3	186391105	186454580	-	.	.	.	.	.	.	.	.	.	.	.	.	.	.	.	.	.	.	.	.
4346	1	MAP3K13	NM_001242317	chr3	186483422	186689576	+	.	.	.	.	.	.	.	.	.	.	.	.	.	.	.	.	.	.	.	.
6468	0,5	IGF2BP2	NM_001007225	chr3	186844220	187025521	-	.	.	.	.	.	.	.	.	.	.	.	.	.	.	.	.	.	.	.	.
1392	2	ETV5	NM_004454	chr3	187246799	187309595	-	.	.	.	.	.	.	.	.	.	.	.	.	.	.	.	.	.	.	.	.
322	3,75	DGKG	NM_001346	chr3	187347683	187562717	-	.	.	.	.	.	.	.	.	.	.	.	.	.	.	.	.	.	.	.	.
6450	0,5	HRG	NM_000412	chr3	187866491	187878717	+	.	.	.	.	.	.	.	.	.	.	.	.	.	.	.	.	.	.	.	.
2638	1,25	KNG1	NM_001102416	chr3	187917791	187943372	+	.	.	.	.	.	.	.	.	.	.	.	.	.	.	.	.	.	.	.	.
821	2,5	EIF4A2	NM_001967	chr3	187984054	187990379	+	.	.	.	.	.	.	.	.	.	.	.	.	.	.	.	.	.	.	.	.

Table S2

tumor associated genes								HIV				MLV				MMTV				MMTV(SIN)				MMTV(SIN)arrest			
rank	score	gene symbol	RefSEq	chrom	txStart	txEnd	strand	integrant	chr	position	strand	integrant	chr	position	strand	integrant	chr	position	strand	integrant	chr	position	strand	integrant	chr	position	strand
5100	1	RFC4	NM_002916	chr3	187990375	188006984	-	.	.	.	.	.	.	.	.	.	.	.	.	.	.	.	.	.	.	.	.
2419	1,5	ST6GAL1	NM_173217	chr3	188131008	188279035	+	.	.	.	.	.	.	.	.	.	.	.	.	.	.	.	.	.	.	.	.
6827	0,5	RPL39L	NM_052969	chr3	188321434	188339957	-	.	.	.	.	.	.	.	.	.	.	.	.	.	.	.	.	.	.	.	.
1968	1,75	SST	NM_001048	chr3	188869387	188870895	-	.	.	.	.	.	.	.	.	.	.	.	.	.	.	.	.	.	.	.	.
533	3	BCL6	NM_001134738	chr3	188921858	188935389	-	.	.	.	.	.	.	.	.	.	.	.	.	.	.	.	.	.	.	.	.
1508	2	LPP	NM_001167672	chr3	189354356	190091154	+	CL528901	chr3	189437448	-	.	.	.	.	_280_1_183	chr3	189423845	+	.	.	.	.	.	.	.	.
1508	2	LPP	NM_001167672	chr3	189354356	190091154	+	CL528902	chr3	189941844	-	.	.	.	.	_280_1_183	chr3	189423845	+	.	.	.	.	.	.	.	.
763	2,75	TP63	NM_001114981	chr3	190990142	191097762	+	.	.	.	.	.	.	.	.	.	.	.	.	.	.	.	.	.	.	.	.
4136	1	IL1RAP	NM_001167931	chr3	191714533	191857680	+	.	.	.	.	.	.	.	.	.	.	.	.	.	.	.	.	.	.	.	.
5908	0,75	FGF12	NM_021032	chr3	193339875	193609532	-	.	.	.	.	.	.	.	.	.	.	.	.	.	.	.	.	.	.	.	.
1072	2,25	HRASLS	NM_020386	chr3	194441610	194471338	+	.	.	.	.	.	.	.	.	.	.	.	.	.	.	.	.	.	.	.	.
3065	1	ATP13A5	NM_198505	chr3	194475524	194579208	-	.	.	.	.	.	.	.	.	.	.	.	.	.	.	.	.	.	.	.	.
4663	1	OPA1	NM_130834	chr3	194793626	194898294	+	.	.	.	.	.	.	.	.	.	.	.	.	.	.	.	.	.	.	.	.
2614	1,25	HES1	NM_005524	chr3	195336624	195339095	+	.	.	.	.	.	.	.	.	.	.	.	.	.	.	.	.	.	.	.	.
4915	1	PPP1R2	NM_006241	chr3	196722509	196751513	-	.	.	.	.	AY516583	chr3	196749033	-	.	.	.	.	.	.	.	.	.	.	.	.
996	2,25	APOD	NM_001647	chr3	196776861	196792365	-	.	.	.	.	.	.	.	.	.	.	.	.	.	.	.	.	.	.	.	.
311	4	TNK2	NM_001010938	chr3	197074632	197106829	-	.	.	.	.	.	.	.	.	.	.	.	.	.	.	.	.	.	.	.	.
433	3,5	TFRC	NM_001128148	chr3	197260551	197293429	-	.	.	.	.	.	.	.	.	.	.	.	.	.	.	.	.	.	.	.	.
4725	1	PCYT1A	NM_005017	chr3	197449649	197498981	-	.	.	.	.	.	.	.	.	.	.	.	.	.	.	.	.	.	.	.	.
4806	1	PIGX	NM_017861	chr3	197923641	197947273	+	.	.	.	.	.	.	.	.	.	.	.	.	.	.	.	.	.	.	.	.
4689	1	PAK2	NM_002577	chr3	197951124	198043915	+	.	.	.	.	.	.	.	.	.	.	.	.	.	.	.	.	.	.	.	.
4807	1	PIGZ	NM_025163	chr3	198157610	198180101	-	.	.	.	.	.	.	.	.	.	.	.	.	.	.	.	.	.	.	.	.
2107	1,5	DLG1	NM_001204388	chr3	198253827	198395399	-	.	.	.	.	.	.	.	.	.	.	.	.	.	.	.	.	.	.	.	.
3138	1	BDH1	NM_203315	chr3	198721050	198784591	-	.	.	.	.	.	.	.	.	.	.	.	.	.	.	.	.	.	.	.	.
6570	0,5	LRCH3	NM_032773	chr3	199002541	199082853	+	CL529286	chr3	199026514	+	.	.	.	.	.	.	.	.	.	.	.	.	.	.	.	.
4798	1	PIGG	NM_017733	chr4	482988	523320	+	.	.	.	.	.	.	.	.	.	.	.	.	.	.	.	.	.	.	.	.
3079	1	ATP5I	NR_033743	chr4	656224	658127	-	.	.	.	.	.	.	.	.	.	.	.	.	.	.	.	.	.	.	.	.
6211	0,5	CPLX1	NM_006651	chr4	768744	809945	-	.	.	.	.	.	.	.	.	.	.	.	.	.	.	.	.	.	.	.	.
3835	1	GAK	NM_005255	chr4	833064	916174	-	.	.	.	.	.	.	.	.	.	.	.	.	.	.	.	.	.	.	.	.
1352	2	DGKQ	NM_001347	chr4	942671	957348	-	.	.	.	.	.	.	.	.	.	.	.	.	.	.	.	.	.	.	.	.
1463	2	IDUA	NM_000203	chr4	970784	988317	+	.	.	.	.	.	.	.	.	.	.	.	.	.	.	.	.	.	.	.	.
3776	1	FGFRL1	NM_001004356	chr4	995609	1010686	+	.	.	.	.	.	.	.	.	.	.	.	.	.	.	.	.	.	.	.	.
7570	0,25	SPON2	NM_001199021	chr4	1150720	1192750	-	.	.	.	.	.	.	.	.	.	.	.	.	.	.	.	.	.	.	.	.
811	2,5	CTBP1	NM_001012614	chr4	1195227	1232908	-	.	.	.	.	.	.	.	.	.	.	.	.	.	.	.	.	.	.	.	.
5424	1	TACC3	NM_006342	chr4	1693014	1716703	+	.	.	.	.	.	.	.	.	.	.	.	.	.	.	.	.	.	.	.	.
28	8,5	FGFR3	NM_000142	chr4	1764836	1780397	+	.	.	.	.	.	.	.	.	.	.	.	.	.	.	.	.	.	.	.	.
1854	2	WHSC1	NM_001042424	chr4	1842920	1953732	+	.	.	.	.	.	.	.	.	.	.	.	.	.	.	.	.	.	.	.	.
6648	0,5	NAT8L	NM_178557	chr4	2031036	2040614	+	.	.	.	.	.	.	.	.	.	.	.	.	.	.	.	.	.	.	.	.
1647	2	POLN	NM_181808	chr4	2043442	2200756	-	CL799646	chr4	2194776	-	.	.	.	.	.	.	.	.	.	.	.	.	.	.	.	.
1647	2	POLN	NM_181808	chr4	2043442	2200756	-	CL529597	chr4	2199710	-	.	.	.	.	.	.	.	.	.	.	.	.	.	.	.	.
1647	2	POLN	NM_181808	chr4	2043442	2200756	-	BH609500	chr4	2186001	+	.	.	.	.	.	.	.	.	.	.	.	.	.	.	.	.
7123	0,5	ZFYVE28	NM_001172659	chr4	2241121	2336402	-	.	.	.	.	.	.	.	.	.	.	.	.	.	.	.	.	.	.	.	.
7545	0,25	SH3BP2	NM_001122681	chr4	2764547	2812621	+	.	.	.	.	.	.	.	.	.	.	.	.	.	.	.	.	.	.	.	.
3972	1	GRK4	NM_001004057	chr4	2935140	3012272	+	.	.	.	.	.	.	.	.	.	.	.	.	.	.	.	.	.	.	.	.
2196	1,5	HTT	NM_002111	chr4	3046205	3215485	+	.	.	.	.	.	.	.	.	.	.	.	.	.	.	.	.	.	.	.	.
6814	0,5	RGS12	NM_198227	chr4	3341521	3411438	+	.	.	.	.	.	.	.	.	.	.	.	.	.	.	.	.	.	.	.	.
7344	0,25	HGFAC	NM_001528	chr4	3413523	3421011	+	.	.	.	.	.	.	.	.	.	.	.	.	.	.	.	.	.	.	.	.
5996	0,5	ADRA2C	NM_000683	chr4	3738093	3740051	+	.	.	.	.	.	.	.	.	.	.	.	.	.	.	.	.	.	.	.	.
6248	0,5	D4S234E	NM_001040101	chr4	4438883	4471686	+	.	.	.	.	.	.	.	.	.	.	.	.	.	.	.	.	.	.	.	.
4446	1	MSX1	NM_002448	chr4	4912292	4916561	+	.	.	.	.	.	.	.	.	.	.	.	.	.	.	.	.	.	.	.	.
6246	0,5	CYTL1	NM_018659	chr4	5067214	5072098	-	.	.	.	.	.	.	.	.	.	.	.	.	.	.	.	.	.	.	.	.

Table S2

tumor associated genes								HIV				MLV				MMTV				MMTV(SIN)				MMTV(SIN)arrest			
rank	score	gene symbol	RefSEq	chrom	txStart	txEnd	strand	integrant	chr	position	strand	integrant	chr	position	strand	integrant	chr	position	strand	integrant	chr	position	strand	integrant	chr	position	strand
2790	1,25	STK32B	NM_018401	chr4	5104427	5553626	+	CL528905	chr4	5199607	+	.	.	.	.	.	.	.	.	.	.	.	.	.	.	.	.
5894	0,75	CRMP1	NM_001313	chr4	5873391	5941216	-	.	.	.	.	.	.	.	.	.	.	.	.	.	.	.	.	.	.	.	.
2210	1,5	JAKMIP1	NM_001099433	chr4	6078826	6253219	-	.	.	.	.	.	.	.	.	.	.	.	.	.	.	.	.	.	.	.	.
1955	1,75	PPP2R2C	NM_001206995	chr4	6373205	6608306	-	.	.	.	.	.	.	.	.	.	.	.	.	.	.	.	.	.	.	.	.
4339	1	MAN2B2	NM_015274	chr4	6627802	6675089	+	.	.	.	.	.	.	.	.	.	.	.	.	.	.	.	.	.	.	.	.
7526	0,25	S100P	NM_005980	chr4	6746466	6749798	+	.	.	.	.	.	.	.	.	.	.	.	.	.	.	.	.	.	.	.	.
6955	0,5	SORCS2	NM_020777	chr4	7245274	7795464	+	.	.	.	.	.	.	.	.	.	.	.	.	.	.	.	.	.	.	.	.
5997	0,5	AFAP1	NM_198595	chr4	7811339	7992553	-	.	.	.	.	.	.	.	.	.	.	.	.	.	.	.	.	.	.	.	.
1198	2	ABLIM2	NM_001130086	chr4	8017936	8211459	-	.	.	.	.	.	.	.	.	.	.	.	.	.	.	.	.	.	.	.	.
5232	1	SH3TC1	NM_018986	chr4	8251959	8293730	+	.	.	.	.	.	.	.	.	.	.	.	.	.	.	.	.	.	.	.	.
2886	1	ACOX3	NM_003501	chr4	8418908	8493352	-	.	.	.	.	.	.	.	.	.	.	.	.	.	.	.	.	.	.	.	.
6402	0,5	GPR78	NR_045511	chr4	8632863	8642650	+	.	.	.	.	.	.	.	.	.	.	.	.	.	.	.	.	.	.	.	.
6289	0,5	DRD5	NM_000798	chr4	9392355	9394731	+	.	.	.	.	.	.	.	.	.	.	.	.	.	.	.	.	.	.	.	.
5689	1	WDR1	NM_017491	chr4	9685060	9727671	-	.	.	.	.	.	.	.	.	.	.	.	.	.	.	.	.	.	.	.	.
4082	1	HS3ST1	NM_005114	chr4	11009085	11039635	-	.	.	.	.	.	.	.	.	.	.	.	.	.	.	.	.	.	.	.	.
5026	1	RAB28	NM_001017979	chr4	12978444	13095087	-	AY516975.1	chr4	13085431	+	.	.	.	.	.	.	.	.	.	.	.	.	.	.	.	.
3750	1	FBXL5	NR_036464	chr4	15215104	15266133	-	.	.	.	.	.	.	.	.	.	.	.	.	.	.	.	.	.	.	.	.
3163	1	BST1	NM_004334	chr4	15313670	15342894	+	.	.	.	.	.	.	.	.	.	.	.	.	.	.	.	.	.	.	.	.
3299	1	CD38	NM_001775	chr4	15389028	15459804	+	.	.	.	.	.	.	.	.	.	.	.	.	.	.	.	.	.	.	.	.
6769	0,5	PROM1	NM_001145852	chr4	15578946	15686839	-	.	.	.	.	.	.	.	.	.	.	.	.	.	.	.	.	.	.	.	.
2355	1,5	QDPR	NM_000320	chr4	17097113	17122955	-	.	.	.	.	.	.	.	.	.	.	.	.	.	.	.	.	.	.	.	.
4258	1	LAP3	NM_015907	chr4	17188024	17218688	+	BH609497	chr4	17212882	+	.	.	.	.	.	.	.	.	.	.	.	.	.	.	.	.
2270	1,5	NCAPG	NM_022346	chr4	17421622	17455585	+	AY517116.1	chr4	17444556	+	.	.	.	.	.	.	.	.	.	.	.	.	.	.	.	.
2776	1,25	SLIT2	NM_004787	chr4	19864332	20229886	+	.	.	.	.	.	.	.	.	.	.	.	.	.	.	.	.	2511_217_1	chr4	20209426	+
3861	1	GBA3	NM_020973	chr4	22303645	22430290	+	.	.	.	.	.	.	.	.	.	.	.	.	.	.	.	.	.	.	.	.
190	4,5	PPARGC1A	NM_013261	chr4	23402741	23500798	-	.	.	.	.	.	.	.	.	.	.	.	.	.	.	.	.	.	.	.	.
4793	1	PI4K2B	NM_018323	chr4	24844750	24889929	+	CL529547	chr4	24879030	-	.	.	.	.	.	.	.	.	.	.	.	.	.	.	.	.
529	3	ANAPC4	NM_013367	chr4	24987945	25029218	+	.	.	.	.	.	.	.	.	.	.	.	.	.	.	.	.	.	.	.	.
5272	1	SLC34A2	NM_001177998	chr4	25266532	25289466	+	.	.	.	.	.	.	.	.	.	.	.	.	.	.	.	.	.	.	.	.
947	2,5	RBPJ	NM_005349	chr4	25930429	26045850	+	CL529287	chr4	26043618	+	.	.	.	.	.	.	.	.	.	.	.	.	.	.	.	.
947	2,5	RBPJ	NM_005349	chr4	25930429	26045850	+	CL529672	chr4	25982020	+	.	.	.	.	.	.	.	.	.	.	.	.	.	.	.	.
2308	1,5	PCDH7	NM_032457	chr4	30331134	30757521	+	.	.	.	.	.	.	.	.	.	.	.	.	.	.	.	.	.	.	.	.
6028	0,5	ARAP2	NM_015230	chr4	35744014	35922374	-	CL529539	chr4	35900289	-	.	.	.	.	.	.	.	.	.	.	.	.	.	.	.	.
6028	0,5	ARAP2	NM_015230	chr4	35744014	35922374	-	CL799645	chr4	35763221	-	.	.	.	.	.	.	.	.	.	.	.	.	.	.	.	.
6524	0,5	KIAA1239	NM_001144990	chr4	36923084	37127482	+	.	.	.	.	.	.	.	.	.	.	.	.	.	.	.	.	.	.	.	.
4774	1	PGM2	NM_018290	chr4	37504676	37540954	+	.	.	.	.	.	.	.	.	.	.	.	.	.	.	.	.	.	.	.	.
6809	0,5	RFC1	NM_002913	chr4	38965463	39044396	-	.	.	.	.	.	.	.	.	.	.	.	.	.	.	.	.	.	.	.	.
4282	1	LIAS	NM_006859	chr4	39137059	39155666	+	.	.	.	.	.	.	.	.	.	.	.	.	.	.	.	.	.	.	.	.
5622	1	UGDH	NM_003359	chr4	39176770	39205613	-	.	.	.	.	.	.	.	.	.	.	.	.	.	.	.	.	.	.	.	.
5604	1	UBE2K	NM_005339	chr4	39376058	39460805	+	.	.	.	.	.	.	.	.	.	.	.	.	.	.	.	.	.	.	.	.
1553	2	N4BP2	NM_018177	chr4	39734918	39836267	+	.	.	.	.	.	.	.	.	.	.	.	.	.	.	.	.	.	.	.	.
1724	2	RHOH	NM_004310	chr4	39874921	39922676	+	.	.	.	.	.	.	.	.	.	.	.	.	.	.	.	.	.	.	.	.
6179	0,5	CHRNA9	NM_017581	chr4	40032102	40051991	+	.	.	.	.	.	.	.	.	.	.	.	.	.	.	.	.	.	.	.	.
6802	0,5	RBM47	NM_019027	chr4	40120028	40212747	-	.	.	.	.	AY516269	chr4	40205527	+	.	.	.	.	.	.	.	.	.	.	.	.
1834	2	UCHL1	NM_004181	chr4	40953654	40965203	+	.	.	.	.	.	.	.	.	.	.	.	.	.	.	.	.	.	.	.	.
1615	2	PHOX2B	NM_003924	chr4	41440855	41445744	-	.	.	.	.	.	.	.	.	.	.	.	.	.	.	.	.	.	.	.	.
6512	0,5	KCTD8	NM_198353	chr4	43870676	44145581	-	.	.	.	.	.	.	.	.	.	.	.	.	.	.	.	.	.	.	.	.
3931	1	GNPDA2	NM_001270880	chr4	44398568	44423408	-	.	.	.	.	.	.	.	.	.	.	.	.	.	.	.	.	.	.	.	.
6375	0,5	GABRG1	NM_173536	chr4	45732543	45820839	-	.	.	.	.	.	.	.	.	.	.	.	.	.	.	.	.	.	.	.	.
3830	1	GABRA2	NM_001114175	chr4	45946338	46086153	-	.	.	.	.	.	.	.	.	.	.	.	.	.	.	.	.	.	.	.	.
3441	1	COX7B2	NM_130902	chr4	46431603	46606009	-	.	.	.	.	.	.	.	.	.	.	.	.	.	.	.	.	.	.	.	.

Table S2

tumor associated genes								HIV				MLV				MMTV				MMTV(SIN)				MMTV(SIN)arrest			
rank	score	gene symbol	RefSEq	chrom	txStart	txEnd	strand	integrant	chr	position	strand	integrant	chr	position	strand	integrant	chr	position	strand	integrant	chr	position	strand	integrant	chr	position	strand
3831	1	GABRA4	NM_001204266	chr4	46615673	46690279	-	.	.	.	.	.	.	.	.	.	.	.	.	.	.	.	.	.	.	.	.
3832	1	GABRB1	NM_000812	chr4	46728051	47123204	+	.	.	.	.	AY515869	chr4	46892391	-	.	.	.	.	.	.	.	.	.	.	.	.
5592	1	TXK	NM_003328	chr4	47763166	47831030	-	.	.	.	.	.	.	.	.	.	.	.	.	.	.	.	.	.	.	.	.
1800	2	TEC	NM_003215	chr4	47832556	47966571	-	.	.	.	.	.	.	.	.	.	.	.	.	.	.	.	.	.	.	.	.
7501	0,25	RASL11B	NM_023940	chr4	53423251	53427759	+	.	.	.	.	.	.	.	.	.	.	.	.	.	.	.	.	.	.	.	.
1045	2,25	FIP1L1	NM_030917	chr4	53938576	54020860	+	.	.	.	.	.	.	.	.	.	.	.	.	.	.	.	.	.	.	.	.
4295	1	LNX1	NM_032622	chr4	54021193	54119193	-	.	.	.	.	.	.	.	.	.	.	.	.	.	.	.	.	.	.	.	.
2756	1,25	RPL21P44	NR_027153	chr4	54546422	54548206	-	.	.	.	.	.	.	.	.	.	.	.	.	.	.	.	.	.	.	.	.
452	3,25	CHIC2	NM_012110	chr4	54570714	54625572	-	.	.	.	.	.	.	.	.	.	.	.	.	.	.	.	.	.	.	.	.
7337	0,25	GSX2	NM_133267	chr4	54661004	54662879	+	.	.	.	.	.	.	.	.	.	.	.	.	.	.	.	.	.	.	.	.
11	11	PDGFRA	NM_006206	chr4	54790020	54859169	+	.	.	.	.	.	.	.	.	.	.	.	.	.	.	.	.	.	.	.	.
34	8,25	KIT	NM_000222	chr4	55218851	55301638	+	.	.	.	.	.	.	.	.	.	.	.	.	.	.	.	.	.	.	.	.
81	6,25	KDR	NM_002253	chr4	55639182	55686519	-	.	.	.	.	.	.	.	.	.	.	.	.	.	.	.	.	.	.	.	.
7572	0,25	SRD5A3	NM_024592	chr4	55907144	55934023	+	.	.	.	.	.	.	.	.	.	.	.	.	.	.	.	.	.	.	.	.
7593	0,25	TMEM165	NM_018475	chr4	55956836	55987099	+	.	.	.	.	.	.	.	.	.	.	.	.	.	.	.	.	.	.	.	.
3393	1	CLOCK	NM_001267843	chr4	55988824	56106856	-	AY517041.1	chr4	55997617	+	.	.	.	.	.	.	.	.	.	.	.	.	.	.	.	.
7469	0,25	PDCL2	NM_152401	chr4	56117448	56153136	-	.	.	.	.	.	.	.	.	.	.	.	.	.	.	.	.	.	.	.	.
5939	0,75	NMU	NM_006681	chr4	56156154	56197222	-	.	.	.	.	.	.	.	.	.	.	.	.	.	.	.	.	.	.	.	.
6324	0,5	EXOC1	NM_018261	chr4	56414572	56466001	+	CL529538	chr4	56446780	-	.	.	.	.	.	.	.	.	.	.	.	.	.	.	.	.
4891	1	PPAT	NM_002703	chr4	56954285	56996559	-	.	.	.	.	.	.	.	.	.	.	.	.	.	.	.	.	.	.	.	.
4688	1	PAICS	NM_006452	chr4	56996671	57022291	+	CL528906	chr4	57002038	+	.	.	.	.	.	.	.	.	.	.	.	.	.	.	.	.
3040	1	ARL9	NM_206919	chr4	57066131	57084815	+	.	.	.	.	.	.	.	.	.	.	.	.	.	.	.	.	.	.	.	.
7355	0,25	HOPX	NM_032495	chr4	57208910	57242629	-	.	.	.	.	.	.	.	.	.	.	.	.	.	.	.	.	.	.	.	.
5332	1	SPINK2	NM_021114	chr4	57370790	57382650	-	.	.	.	.	.	.	.	.	.	.	.	.	.	.	.	.	.	.	.	.
7512	0,25	REST	NM_001193508	chr4	57469835	57496767	+	.	.	.	.	.	.	.	.	.	.	.	.	.	.	.	.	.	.	.	.
647	3	POLR2B	NM_000938	chr4	57539865	57592091	+	.	.	.	.	.	.	.	.	.	.	.	.	.	.	.	.	.	.	.	.
2622	1,25	IGFBP7	NM_001553	chr4	57591993	57671308	-	.	.	.	.	.	.	.	.	.	.	.	.	.	.	.	.	.	.	.	.
1507	2	LPHN3	NM_015236	chr4	62045433	62620763	+	CL799792	chr4	62438057	-	.	.	.	.	.	.	.	.	.	.	.	.	748_118_1	chr4	62550576	-
463	3,25	EPHA5	NM_182472	chr4	65867875	66218248	-	.	.	.	.	.	.	.	.	.	.	.	.	.	.	.	.	.	.	.	.
3329	1	CENPC1	NM_001812	chr4	68020583	68093851	-	.	.	.	.	.	.	.	.	.	.	.	.	.	.	.	.	.	.	.	.
5422	1	SYT14L	NR_027094	chr4	68608922	68611610	-	.	.	.	.	.	.	.	.	.	.	.	.	.	.	.	.	.	.	.	.
5634	1	UGT2B17	NM_001077	chr4	69085497	69116840	-	.	.	.	.	.	.	.	.	.	.	.	.	.	.	.	.	.	.	.	.
5633	1	UGT2B15	NM_001076	chr4	69194909	69219089	-	.	.	.	.	.	.	.	.	.	.	.	.	.	.	.	.	.	.	.	.
5632	1	UGT2B10	NM_001075	chr4	69716301	69732330	+	.	.	.	.	.	.	.	.	.	.	.	.	.	.	.	.	.	.	.	.
5631	1	UGT2A3	NM_024743	chr4	69828765	69852098	-	.	.	.	.	.	.	.	.	.	.	.	.	.	.	.	.	.	.	.	.
5636	1	UGT2B7	NM_001074	chr4	69996781	70013294	+	.	.	.	.	.	.	.	.	.	.	.	.	.	.	.	.	.	.	.	.
1837	2	UGT2B11	NM_001073	chr4	70100635	70115038	-	.	.	.	.	.	.	.	.	.	.	.	.	.	.	.	.	.	.	.	.
5635	1	UGT2B28	NM_053039	chr4	70180805	70195357	+	.	.	.	.	.	.	.	.	.	.	.	.	.	.	.	.	.	.	.	.
1838	2	UGT2B4	NM_021139	chr4	70380471	70396215	-	.	.	.	.	.	.	.	.	.	.	.	.	.	.	.	.	.	.	.	.
5630	1	UGT2A1	NM_006798	chr4	70488723	70553556	-	.	.	.	.	.	.	.	.	.	.	.	.	.	.	.	.	.	.	.	.
5406	1	SULT1B1	NM_014465	chr4	70627274	70661019	-	CL799883	chr4	70649325	+	.	.	.	.	.	.	.	.	.	.	.	.	.	.	.	.
1786	2	SULT1E1	NM_005420	chr4	70741518	70760459	-	.	.	.	.	.	.	.	.	.	.	.	.	.	.	.	.	.	.	.	.
6839	0,5	RUFY3	NM_001037442	chr4	71806559	71893200	+	CL529731	chr4	71847911	+	.	.	.	.	.	.	.	.	.	.	.	.	.	.	.	.
3535	1	DCK	NM_000788	chr4	72078128	72115493	+	.	.	.	.	.	.	.	.	.	.	.	.	.	.	.	.	.	.	.	.
5281	1	SLC4A4	NM_003759	chr4	72423633	72656668	+	.	.	.	.	.	.	.	.	.	.	.	.	.	.	.	.	.	.	.	.
3865	1	GC	NM_001204307	chr4	72826274	72888622	-	.	.	.	.	.	.	.	.	.	.	.	.	.	.	.	.	.	.	.	.
5875	0,75	ADAMTS3	NM_014243	chr4	73365549	73653380	-	.	.	.	.	AY516344	chr4	73530678	-	.	.	.	.	.	.	.	.	.	.	.	.
2989	1	ANKRD17	NM_198889	chr4	74159365	74343366	-	AY517113.1	chr4	74294562	-	.	.	.	.	.	.	.	.	.	.	.	.	.	.	.	.
2493	1,25	AFP	NM_001134	chr4	74520796	74540356	+	.	.	.	.	.	.	.	.	.	.	.	.	.	.	.	.	.	.	.	.
597	3	IL8	NM_000584	chr4	74825086	74828297	+	.	.	.	.	.	.	.	.	.	.	.	.	.	.	.	.	.	.	.	.
4892	1	PPBP	NM_002704	chr4	75071019	75072771	-	.	.	.	.	.	.	.	.	.	.	.	.	.	.	.	.	.	.	.	.

Table S2

tumor associated genes								HIV				MLV				MMTV				MMTV(SIN)				MMTV(SIN)arrest			
rank	score	gene symbol	RefSEq	chrom	txStart	txEnd	strand	integrant	chr	position	strand	integrant	chr	position	strand	integrant	chr	position	strand	integrant	chr	position	strand	integrant	chr	position	strand
6236	0,5	CXCL5	NM_002994	chr4	75080222	75083280	-	.	.	.	.	.	.	.	.	.	.	.	.	.	.	.	.	.	.	.	.
6235	0,5	CXCL3	NM_002090	chr4	75121175	75123354	-	.	.	.	.	.	.	.	.	.	.	.	.	.	.	.	.	.	.	.	.
6234	0,5	CXCL2	NM_002089	chr4	75181617	75183861	-	.	.	.	.	.	.	.	.	.	.	.	.	.	.	.	.	.	.	.	.
1389	2	EREG	NM_001432	chr4	75449723	75473341	+	.	.	.	.	AY516540	chr4	75461235	+	.	.	.	.	.	.	.	.	.	.	.	.
2003	1,5	AREG	NM_001657	chr4	75529716	75539590	+	.	.	.	.	.	.	.	.	.	.	.	.	.	.	.	.	.	.	.	.
2030	1,5	BTC	NM_001729	chr4	75890471	75938906	-	.	.	.	.	.	.	.	.	.	.	.	.	.	.	.	.	.	.	.	.
500	3,25	RCHY1	NM_015436	chr4	76623270	76658664	-	CL528774	chr4	76651763	+	.	.	.	.	.	.	.	.	.	.	.	.	.	.	.	.
2066	1,5	CDKL2	NM_003948	chr4	76720727	76774745	-	.	.	.	.	.	.	.	.	.	.	.	.	.	.	.	.	.	.	.	.
4895	1	PPEF2	NM_006239	chr4	77000049	77042705	-	.	.	.	.	.	.	.	.	.	.	.	.	.	.	.	.	.	.	.	.
4487	1	NAAA	NM_014435	chr4	77053832	77081190	-	.	.	.	.	AY516803	chr4	77080152	+	.	.	.	.	.	.	.	.	.	.	.	.
3492	1	CXCL9	NM_002416	chr4	77141646	77147665	-	.	.	.	.	.	.	.	.	.	.	.	.	.	.	.	.	.	.	.	.
6232	0,5	CXCL10	NM_001565	chr4	77161292	77163713	-	.	.	.	.	.	.	.	.	.	.	.	.	.	.	.	.	.	.	.	.
6233	0,5	CXCL11	NM_005409	chr4	77173863	77176374	-	.	.	.	.	.	.	.	.	.	.	.	.	.	.	.	.	.	.	.	.
6692	0,5	NUP54	NM_017426	chr4	77254840	77288679	-	BH609504	chr4	77274308	+	.	.	.	.	.	.	.	.	.	.	.	.	.	.	.	.
5237	1	SHROOM3	NM_020859	chr4	77575276	77923429	+	.	.	.	.	.	.	.	.	.	.	.	.	.	.	.	.	1863_169_7	chr4	77910929	-
5237	1	SHROOM3	NM_020859	chr4	77575276	77923429	+	.	.	.	.	.	.	.	.	.	.	.	.	.	.	.	.	1529_152_1	chr4	77599524	-
2992	1	ANXA3	NM_005139	chr4	79691765	79750629	+	.	.	.	.	.	.	.	.	.	.	.	.	.	.	.	.	.	.	.	.
3153	1	BMP2K	NM_198892	chr4	79916555	80052365	+	.	.	.	.	.	.	.	.	.	.	.	.	.	.	.	.	.	.	.	.
7312	0,25	FGF5	NM_033143	chr4	81406765	81431195	+	CL528908	chr4	81414824	-	.	.	.	.	.	.	.	.	.	.	.	.	.	.	.	.
7191	0,25	BMP3	NM_001201	chr4	82171142	82197709	+	.	.	.	.	.	.	.	.	.	.	.	.	.	.	.	.	.	.	.	.
4945	1	PRKG2	NM_006259	chr4	82228860	82345239	-	.	.	.	.	.	.	.	.	.	.	.	.	.	.	.	.	.	.	.	.
6748	0,5	PLAC8	NM_016619	chr4	84230234	84254935	-	.	.	.	.	.	.	.	.	.	.	.	.	.	.	.	.	.	.	.	.
3423	1	COQ2	NM_015697	chr4	84404000	84425091	-	.	.	.	.	.	.	.	.	.	.	.	.	.	.	.	.	.	.	.	.
1071	2,25	HPSE	NM_006665	chr4	84432637	84475330	-	CL529688	chr4	84464730	-	.	.	.	.	.	.	.	.	.	.	.	.	.	.	.	.
4029	1	HELQ	NM_133636	chr4	84547522	84596049	-	.	.	.	.	.	.	.	.	.	.	.	.	.	.	.	.	.	.	.	.
4582	1	NKX6-1	NM_006168	chr4	85633459	85638411	-	.	.	.	.	.	.	.	.	.	.	.	.	.	.	.	.	.	.	.	.
3322	1	CDS1	NM_001263	chr4	85723080	85791517	+	.	.	.	.	.	.	.	.	.	.	.	.	.	.	.	.	.	.	.	.
5688	1	WDFY3	NM_014991	chr4	85809716	86106568	-	CL799532	chr4	85961713	+	.	.	.	.	.	.	.	.	.	.	.	.	.	.	.	.
2659	1,25	MAPK10	NM_138980	chr4	87155299	87500399	-	.	.	.	.	AY516294	chr4	87358914	+	.	.	.	.	.	.	.	.	.	.	.	.
1150	2,25	PTPN13	NM_080684	chr4	87734491	87955352	+	.	.	.	.	.	.	.	.	_174_1_155	chr4	87909221	+	.	.	.	.	.	.	.	.
1227	2	AFF1	NM_001166693	chr4	88075177	88281230	+	.	.	.	.	.	.	.	.	_281_1_184	chr4	88172116	-	.	.	.	.	.	.	.	.
4648	1	NUDT9	NM_024047	chr4	88562751	88599630	+	.	.	.	.	.	.	.	.	.	.	.	.	.	.	.	.	.	.	.	.
6960	0,5	SPARCL1	NM_004684	chr4	88613511	88669679	-	.	.	.	.	.	.	.	.	.	.	.	.	.	.	.	.	.	.	.	.
7292	0,25	DSPP	NM_014208	chr4	88748704	88757049	+	.	.	.	.	.	.	.	.	.	.	.	.	.	.	.	.	.	.	.	.
7281	0,25	DMP1	NM_004407	chr4	88790477	88804536	+	.	.	.	.	.	.	.	.	.	.	.	.	.	.	.	.	.	.	.	.
6460	0,5	IBSP	NM_004967	chr4	88939725	88952625	+	.	.	.	.	.	.	.	.	.	.	.	.	.	.	.	.	.	.	.	.
684	3	SPP1	NM_001251830	chr4	89115825	89123587	+	.	.	.	.	.	.	.	.	.	.	.	.	.	.	.	.	.	.	.	.
351	3,5	ABCG2	NM_001257386	chr4	89230439	89371498	-	.	.	.	.	.	.	.	.	.	.	.	.	.	.	.	.	.	.	.	.
2334	1,5	PPM1K	NM_152542	chr4	89400555	89424912	-	.	.	.	.	.	.	.	.	.	.	.	.	.	.	.	.	.	.	.	.
4495	1	NAP1L5	NM_153757	chr4	89836088	89838046	-	.	.	.	.	.	.	.	.	.	.	.	.	.	.	.	.	.	.	.	.
5305	1	SNCA	NM_007308	chr4	90864272	90977150	-	.	.	.	.	.	.	.	.	.	.	.	.	.	.	.	.	.	.	.	.
6409	0,5	GRID2	NM_001510	chr4	93444572	94912672	+	.	.	.	.	.	.	.	.	.	.	.	.	.	.	.	.	.	.	.	.
1005	2,25	BMPR1B	NM_001256792	chr4	96136405	96298624	+	.	.	.	.	.	.	.	.	.	.	.	.	.	.	.	.	38_67_1	chr4	96056529	-
4742	1	PDHA2	NM_005390	chr4	96980261	96981648	+	.	.	.	.	.	.	.	.	.	.	.	.	.	.	.	.	.	.	.	.
944	2,5	RAP1GDS1	NM_001100430	chr4	99401549	99584035	+	.	.	.	.	.	.	.	.	.	.	.	.	.	.	.	.	.	.	.	.
371	3,5	EIF4E	NM_001130678	chr4	100018629	100069266	-	.	.	.	.	.	.	.	.	.	.	.	.	.	.	.	.	.	.	.	.
7416	0,25	METAP1	NM_015143	chr4	100135810	100202983	+	CL529737	chr4	100182669	+	.	.	.	.	.	.	.	.	.	.	.	.	653_115_1	chr4	100179454	-
7416	0,25	METAP1	NM_015143	chr4	100135810	100202983	+	CL799935	chr4	100177630	-	.	.	.	.	.	.	.	.	.	.	.	.	653_115_1	chr4	100179454	-
2911	1	ADH5	NM_000671	chr4	100211152	100228954	-	.	.	.	.	.	.	.	.	.	.	.	.	.	.	.	.	.	.	.	.
2910	1	ADH4	NM_000670	chr4	100263855	100284472	-	.	.	.	.	.	.	.	.	.	.	.	.	.	.	.	.	.	.	.	.
2912	1	ADH6	NM_001102470	chr4	100342817	100359426	-	.	.	.	.	.	.	.	.	.	.	.	.	.	.	.	.	.	.	.	.

Table S2

tumor associated genes								HIV				MLV				MMTV				MMTV(SIN)				MMTV(SIN)arrest			
rank	score	gene symbol	RefSEq	chrom	txStart	txEnd	strand	integrant	chr	position	strand	integrant	chr	position	strand	integrant	chr	position	strand	integrant	chr	position	strand	integrant	chr	position	strand
1223	2	ADH1A	NM_000667	chr4	100416545	100431208	-	.	.	.	.	.	.	.	.	.	.	.	.	.	.	.	.	.	.	.	.
2909	1	ADH1B	NM_000668	chr4	100446549	100461595	-	.	.	.	.	.	.	.	.	.	.	.	.	.	.	.	.	.	.	.	.
2491	1,25	ADH1C	NM_000669	chr4	100476671	100492940	-	.	.	.	.	.	.	.	.	.	.	.	.	.	.	.	.	.	.	.	.
1224	2	ADH7	NM_001166504	chr4	100552440	100575314	-	.	.	.	.	.	.	.	.	.	.	.	.	.	.	.	.	.	.	.	.
6258	0,5	DDIT4L	NM_145244	chr4	101326049	101330678	-	.	.	.	.	.	.	.	.	.	.	.	.	.	.	.	.	.	.	.	.
653	3	PPP3CA	NM_000944	chr4	102163609	102487651	-	BH609509	chr4	102317217	+	.	.	.	.	.	.	.	.	.	.	.	.	.	.	.	.
653	3	PPP3CA	NM_000944	chr4	102163609	102487651	-	CL529569	chr4	102163652	-	.	.	.	.	.	.	.	.	.	.	.	.	.	.	.	.
627	3	NFKB1	NM_001165412	chr4	103641517	103757507	+	CL529744	chr4	103686732	+	.	.	.	.	.	.	.	.	.	.	.	.	.	.	.	.
4341	1	MANBA	NM_005908	chr4	103771690	103901196	-	.	.	.	.	.	.	.	.	.	.	.	.	.	.	.	.	.	.	.	.
5599	1	UBE2D3	NM_181890	chr4	103936216	104009473	-	CL799632	chr4	103938782	-	.	.	.	.	.	.	.	.	.	.	.	.	.	.	.	.
3139	1	BDH2	NM_020139	chr4	104218230	104240473	-	.	.	.	.	.	.	.	.	.	.	.	.	.	.	.	.	.	.	.	.
3330	1	CENPE	NM_001813	chr4	104246411	104339015	-	BH609510	chr4	104304902	+	.	.	.	.	.	.	.	.	.	.	.	.	.	.	.	.
3330	1	CENPE	NM_001813	chr4	104246411	104339015	-	AY516961.1	chr4	104336294	+	.	.	.	.	.	.	.	.	.	.	.	.	.	.	.	.
3494	1	CXXC4	NM_025212	chr4	105608911	105635507	-	.	.	.	.	.	.	.	.	.	.	.	.	.	.	.	.	.	.	.	.
4885	1	PPA2	NM_176866	chr4	106509682	106614676	-	.	.	.	.	.	.	.	.	.	.	.	.	.	.	.	.	.	.	.	.
4600	1	NPNT	NM_001184691	chr4	107036045	107112277	+	.	.	.	.	.	.	.	.	.	.	.	.	.	.	.	.	.	.	.	.
3584	1	DKK2	NM_014421	chr4	108062407	108176902	-	.	.	.	.	.	.	.	.	.	.	.	.	.	.	.	.	.	.	.	.
4698	1	PAPSS1	NM_005443	chr4	108754270	108860868	-	CL800114	chr4	108852522	+	.	.	.	.	.	.	.	.	.	.	.	.	.	.	.	.
1752	2	SGMS2	NM_001136258	chr4	108965169	109055652	+	.	.	.	.	.	.	.	.	.	.	.	.	.	.	.	.	.	.	.	.
4004	1	HADH	NM_005327	chr4	109130318	109175780	+	.	.	.	.	.	.	.	.	.	.	.	.	.	.	.	.	.	.	.	.
1500	2	LEF1	NM_016269	chr4	109188149	109309561	-	.	.	.	.	.	.	.	.	.	.	.	.	.	.	.	.	.	.	.	.
1992	1,5	AGXT2L1	NM_001146627	chr4	109882650	109903684	-	.	.	.	.	.	.	.	.	.	.	.	.	.	.	.	.	.	.	.	.
6138	0,5	CCDC109B	NM_017918	chr4	110700803	110828321	+	.	.	.	.	.	.	.	.	.	.	.	.	.	.	.	.	.	.	.	.
3281	1	CASP6	NM_001226	chr4	110829233	110844078	-	.	.	.	.	.	.	.	.	.	.	.	.	.	.	.	.	.	.	.	.
4826	1	PLA2G12A	NM_030821	chr4	110850593	110870691	-	.	.	.	.	.	.	.	.	.	.	.	.	.	.	.	.	.	.	.	.
6170	0,5	CFI	NM_000204	chr4	110881296	110942784	-	.	.	.	.	.	.	.	.	.	.	.	.	.	.	.	.	.	.	.	.
95	5,75	EGF	NM_001963	chr4	111053488	111153567	+	.	.	.	.	.	.	.	.	.	.	.	.	.	.	.	.	.	.	.	.
3674	1	ELOVL6	NM_001130721	chr4	111189677	111339220	-	.	.	.	.	.	.	.	.	.	.	.	.	.	.	.	.	.	.	.	.
3680	1	ENPEP	NM_001977	chr4	111616677	111703942	+	.	.	.	.	.	.	.	.	.	.	.	.	.	.	.	.	.	.	.	.
7479	0,25	PITX2	NM_153426	chr4	111758028	111782728	-	.	.	.	.	.	.	.	.	.	.	.	.	.	.	.	.	.	.	.	.
2969	1	ALPK1	NM_001253884	chr4	113437947	113583213	+	.	.	.	.	.	.	.	.	.	.	.	.	.	.	.	.	.	.	.	.
2984	1	ANK2	NM_001127493	chr4	113958687	114524345	+	.	.	.	.	AY516702	chr4	114072104	-	.	.	.	.	105_1_747_24	chr4	113992211	+	1851_168_1	chr4	114418526	-
3271	1	CAMK2D	NM_172114	chr4	114597792	114902532	-	CL529409	chr4	114864793	+	.	.	.	.	.	.	.	.	.	.	.	.	.	.	.	.
3271	1	CAMK2D	NM_172114	chr4	114597792	114902532	-	CL529408	chr4	114815732	+	.	.	.	.	.	.	.	.	.	.	.	.	.	.	.	.
3049	1	ARSJ	NM_024590	chr4	115040888	115120327	-	.	.	.	.	.	.	.	.	.	.	.	.	.	.	.	.	.	.	.	.
1839	2	UGT8	NM_003360	chr4	115762971	115817651	+	.	.	.	.	.	.	.	.	.	.	.	.	.	.	.	.	.	.	.	.
4522	1	NDST4	NM_022569	chr4	115968375	116254481	-	.	.	.	.	.	.	.	.	.	.	.	.	.	.	.	.	.	.	.	.
4521	1	NDST3	NM_004784	chr4	119174947	119399237	+	.	.	.	.	.	.	.	.	.	.	.	.	.	.	.	.	.	.	.	.
4960	1	PRSS12	NM_003619	chr4	119420640	119493370	-	.	.	.	.	.	.	.	.	.	.	.	.	.	.	.	.	.	.	.	.
6864	0,5	SEC24D	NM_014822	chr4	119863425	119976774	-	.	.	.	.	.	.	.	.	.	.	.	.	.	.	.	.	.	.	.	.
6997	0,5	SYNPO2	NM_001128933	chr4	120029443	120179117	+	.	.	.	.	.	.	.	.	.	.	.	.	.	.	.	.	.	.	.	.
1606	2	PDE5A	NM_033430	chr4	120634997	120767890	-	.	.	.	.	.	.	.	.	.	.	.	.	.	.	.	.	.	.	.	.
482	3,25	MAD2L1	NM_002358	chr4	121200024	121207461	-	.	.	.	.	.	.	.	.	.	.	.	.	.	.	.	.	.	.	.	.
6020	0,5	ANXA5	NM_001154	chr4	122808601	122837597	-	CL800810	chr4	122821513	-	.	.	.	.	.	.	.	.	.	.	.	.	.	.	.	.
5490	1	TMEM155	NM_152399	chr4	122899534	122905790	-	.	.	.	.	.	.	.	.	.	.	.	.	.	.	.	.	.	.	.	.
712	2,75	CCNA2	NM_001237	chr4	122957048	122964538	-	.	.	.	.	.	.	.	.	.	.	.	.	.	.	.	.	.	.	.	.
331	3,75	IL2	NM_000586	chr4	123592075	123597100	-	.	.	.	.	.	.	.	.	.	.	.	.	.	.	.	.	.	.	.	.
7372	0,25	IL21	NM_001207006	chr4	123753232	123761662	-	.	.	.	.	.	.	.	.	.	.	.	.	.	.	.	.	.	.	.	.
467	3,25	FGF2	NM_002006	chr4	123967312	124038840	+	.	.	.	.	.	.	.	.	.	.	.	.	.	.	.	.	.	.	.	.
3743	1	FAT4	NM_024582	chr4	126457016	126633537	+	.	.	.	.	.	.	.	.	.	.	.	.	.	.	.	.	.	.	.	.
4844	1	PLK4	NM_001190799	chr4	129021465	129039827	+	.	.	.	.	.	.	.	.	.	.	.	.	.	.	.	.	.	.	.	.

Table S2

tumor associated genes								HIV				MLV				MMTV				MMTV(SIN)				MMTV(SIN)arrest			
rank	score	gene symbol	RefSEq	chrom	txStart	txEnd	strand	integrant	chr	position	strand	integrant	chr	position	strand	integrant	chr	position	strand	integrant	chr	position	strand	integrant	chr	position	strand
1613	2	PGRMC2	NM_006320	chr4	129409841	129429434	-	CL799699	chr4	129414364	-	.	.	.	.	.	.	.	.	.	.	.	.	.	.	.	.
4707	1	PCDH18	NM_019035	chr4	138659521	138673079	-	.	.	.	.	.	.	.	.	.	.	.	.	.	.	.	.	.	.	.	.
6926	0,5	SLC7A11	NM_014331	chr4	139304697	139382953	-	CL799685	chr4	139351456	-	.	.	.	.	.	.	.	.	.	.	.	.	.	.	.	.
4544	1	NDUFC1	NM_001184989	chr4	140430520	140443155	-	.	.	.	.	.	.	.	.	.	.	.	.	.	.	.	.	.	.	.	.
676	3	SETD7	NM_030648	chr4	140646641	140697027	-	.	.	.	.	.	.	.	.	.	.	.	.	.	.	.	.	.	.	.	.
2255	1,5	MGST2	NM_001204366	chr4	140806371	140881349	+	.	.	.	.	.	.	.	.	.	.	.	.	.	.	.	.	.	.	.	.
4335	1	MAML3	NM_018717	chr4	140856995	141294683	-	CL528921	chr4	141138375	+	AY516468	chr4	141237075	-	.	.	.	.	.	.	.	.	.	.	.	.
4335	1	MAML3	NM_018717	chr4	140856995	141294683	-	CL800174	chr4	140938059	+	AY516468	chr4	141237075	-	.	.	.	.	.	.	.	.	.	.	.	.
1313	2	CLGN	NM_001130675	chr4	141529056	141568265	-	.	.	.	.	.	.	.	.	.	.	.	.	.	.	.	.	.	.	.	.
865	2,5	IL15	NR_037840	chr4	142777198	142874590	+	.	.	.	.	.	.	.	.	.	.	.	.	.	.	.	.	.	.	.	.
601	3	INPP4B	NM_001101669	chr4	143168631	143987054	-	CL529463	chr4	143215005	-	.	.	.	.	.	.	.	.	1753_151	chr4	143615709	+	.	.	.	.
601	3	INPP4B	NM_001101669	chr4	143168631	143987054	-	CL529295	chr4	143842721	-	.	.	.	.	.	.	.	.	1753_151	chr4	143615709	+	.	.	.	.
212	4,25	GAB1	NM_002039	chr4	144477432	144615168	+	.	.	.	.	.	.	.	.	.	.	.	.	.	.	.	.	.	.	.	.
1763	2	SMARCA5	NM_003601	chr4	144654065	144698092	+	AY516923.1	chr4	144665045	-	.	.	.	.	.	.	.	.	.	.	.	.	.	.	.	.
1763	2	SMARCA5	NM_003601	chr4	144654065	144698092	+	CL529761	chr4	144671007	+	.	.	.	.	.	.	.	.	.	.	.	.	.	.	.	.
7338	0,25	GYPB	NM_002100	chr4	145136706	145159946	-	.	.	.	.	.	.	.	.	.	.	.	.	.	.	.	.	.	.	.	.
3997	1	GYPA	NM_002099	chr4	145249905	145281354	-	.	.	.	.	.	.	.	.	.	.	.	.	.	.	.	.	.	.	.	.
4040	1	HHIP	NM_022475	chr4	145786597	145879331	+	.	.	.	.	.	.	.	.	.	.	.	.	.	.	.	.	.	.	.	.
992	2,25	ANAPC10	NM_001256706	chr4	146135176	146238821	-	AY517173.1	chr4	146222409	-	.	.	.	.	.	.	.	.	.	.	.	.	.	.	.	.
992	2,25	ANAPC10	NM_001256706	chr4	146135176	146238821	-	CL529752	chr4	146206074	-	.	.	.	.	.	.	.	.	.	.	.	.	.	.	.	.
1966	1,75	SMAD1	NM_005900	chr4	146622400	146699775	+	CL799911	chr4	146670020	+	.	.	.	.	.	.	.	.	.	.	.	.	.	.	.	.
1374	2	EDNRA	NM_001957	chr4	148621518	148685556	+	.	.	.	.	.	.	.	.	.	.	.	.	.	.	.	.	.	.	.	.
3024	1	ARHGAP10	NM_024605	chr4	148872902	149213377	+	CL529296	chr4	148945092	+	.	.	.	.	.	.	.	.	.	.	.	.	597_111_2	chr4	149022888	+
4615	1	NR3C2	NM_000901	chr4	149219364	149583122	-	.	.	.	.	.	.	.	.	.	.	.	.	s375_110	chr4	149287550	+	.	.	.	.
1344	2	DCLK2	NM_001040261	chr4	151218875	151398058	+	.	.	.	.	.	.	.	.	.	.	.	.	.	.	.	.	.	.	.	.
4300	1	LRBA	NM_001199282	chr4	151405260	152155869	-	CL529705	chr4	151473314	+	.	.	.	.	.	.	.	.	.	.	.	.	.	.	.	.
4300	1	LRBA	NM_001199282	chr4	151405260	152155869	-	CL529706	chr4	151549274	-	.	.	.	.	.	.	.	.	.	.	.	.	.	.	.	.
4300	1	LRBA	NM_001199282	chr4	151405260	152155869	-	CL529707	chr4	151984698	-	.	.	.	.	.	.	.	.	.	.	.	.	.	.	.	.
7521	0,25	RPS3A	NM_001006	chr4	152240174	152245254	+	.	.	.	.	.	.	.	.	.	.	.	.	.	.	.	.	.	.	.	.
23	9	FBXW7	NM_033632	chr4	153461859	153675635	-	CL800679	chr4	153506574	-	.	.	.	.	.	.	.	.	.	.	.	.	.	.	.	.
23	9	FBXW7	NM_033632	chr4	153461859	153675635	-	CL529297	chr4	153614373	+	.	.	.	.	.	.	.	.	.	.	.	.	.	.	.	.
7602	0,25	TRIM2	NM_001130067	chr4	154293719	154479924	+	.	.	.	.	.	.	.	.	.	.	.	.	.	.	.	.	.	.	.	.
6620	0,5	MND1	NM_001253861	chr4	154485250	154555697	+	.	.	.	.	.	.	.	.	.	.	.	.	.	.	.	.	.	.	.	.
7024	0,5	TLR2	NM_003264	chr4	154824890	154846692	+	.	.	.	.	.	.	.	.	.	.	.	.	.	.	.	.	.	.	.	.
1164	2,25	SFRP2	NM_003013	chr4	154921191	154929678	-	.	.	.	.	.	.	.	.	.	.	.	.	.	.	.	.	.	.	.	.
3994	1	GUCY1A3	NM_000856	chr4	156807311	156877664	+	CL800146	chr4	156846964	+	.	.	.	.	.	.	.	.	.	.	.	.	.	.	.	.
3995	1	GUCY1B3	NM_000857	chr4	156899575	156948233	+	.	.	.	.	.	.	.	.	.	.	.	.	.	.	.	.	.	.	.	.
1972	1,75	TDO2	NM_005651	chr4	157044294	157061008	+	.	.	.	.	.	.	.	.	_293_1_188	chr4	157056546	+	.	.	.	.	.	.	.	.
7470	0,25	PDGFC	NM_016205	chr4	157902212	158111996	-	CL800534	chr4	157909155	-	.	.	.	.	.	.	.	.	.	.	.	.	.	.	.	.
1916	1,75	GRIA2	NM_001083620	chr4	158361269	158506676	+	.	.	.	.	AY516434	chr4	158406607	+	.	.	.	.	.	.	.	.	.	.	.	.
5488	1	TMEM144	NM_018342	chr4	159350850	159395889	+	.	.	.	.	.	.	.	.	.	.	.	.	.	.	.	.	.	.	.	.
6100	0,5	C4orf45	NM_152543	chr4	160034133	160175783	-	.	.	.	.	.	.	.	.	_224_1_169	chr4	160133424	+	.	.	.	.	.	.	.	.
5061	1	RAPGEF2	NM_014247	chr4	160408447	160500751	+	CL800101	chr4	160441422	+	.	.	.	.	.	.	.	.	.	.	.	.	.	.	.	.
1411	2	FSTL5	NM_020116	chr4	162524493	163304636	-	CL800681	chr4	162786086	-	.	.	.	.	.	.	.	.	.	.	.	.	.	.	.	.
6683	0,5	NPY1R	NM_000909	chr4	164464566	164473397	-	.	.	.	.	.	.	.	.	.	.	.	.	.	.	.	.	.	.	.	.
5473	1	TKTL2	NM_032136	chr4	164611696	164614497	-	.	.	.	.	.	.	.	.	.	.	.	.	.	.	.	.	.	.	.	.
4235	1	KLHL2	NM_001161522	chr4	166350620	166463758	+	.	.	.	.	.	.	.	.	.	.	.	.	.	.	.	.	.	.	.	.
6208	0,5	CPE	NM_001873	chr4	166519546	166638932	+	.	.	.	.	.	.	.	.	.	.	.	.	.	.	.	.	.	.	.	.
2991	1	ANXA10	NM_007193	chr4	169250262	169345468	+	.	.	.	.	.	.	.	.	.	.	.	.	.	.	.	.	.	.	.	.
4558	1	NEK1	NM_012224	chr4	170550995	170770353	-	CL529690	chr4	170756669	-	.	.	.	.	.	.	.	.	.	.	.	.	.	.	.	.
3379	1	CLCN3	NM_001243374	chr4	170817787	170880913	+	.	.	.	.	.	.	.	.	.	.	.	.	.	.	.	.	.	.	.	.

Table S2

tumor associated genes								HIV				MLV				MMTV				MMTV(SIN)				MMTV(SIN)arrest			
rank	score	gene symbol	RefSEq	chrom	txStart	txEnd	strand	integrant	chr	position	strand	integrant	chr	position	strand	integrant	chr	position	strand	integrant	chr	position	strand	integrant	chr	position	strand
2842	1	AADAT	NM_182662	chr4	171217947	171247947	-	.	.	.	.	.	.	.	.	.	.	.	.	42_1_1903_6	chr4	171224313	+	.	.	.	.
1420	2	GALNTL6	NM_001034845	chr4	172971149	174198133	+	CL799922	chr4	173376707	-	.	.	.	.	.	.	.	.	.	.	.	.	.	.	.	.
3849	1	GALNT7	NM_017423	chr4	174326478	174481693	+	.	.	.	.	.	.	.	.	.	.	.	.	.	.	.	.	.	.	.	.
1069	2,25	HMGB2	NM_001130689	chr4	174489101	174491495	-	.	.	.	.	.	.	.	.	.	.	.	.	.	.	.	.	.	.	.	.
7360	0,25	HPGD	NM_001256306	chr4	175647902	175680624	-	.	.	.	.	.	.	.	.	.	.	.	.	.	.	.	.	.	.	.	.
2901	1	ADAM29	NM_001130704	chr4	176076083	176135906	+	.	.	.	.	.	.	.	.	.	.	.	.	.	.	.	.	.	.	.	.
3944	1	GPM6A	NM_001261448	chr4	176791081	176945821	-	.	.	.	.	.	.	.	.	.	.	.	.	.	.	.	.	.	.	.	.
5328	1	SPCS3	NM_021928	chr4	177478083	177490390	+	.	.	.	.	.	.	.	.	.	.	.	.	.	.	.	.	.	.	.	.
514	3,25	VEGFC	NM_005429	chr4	177841684	177950889	-	.	.	.	.	.	.	.	.	.	.	.	.	.	.	.	.	.	.	.	.
1572	2	NEIL3	NM_018248	chr4	178467984	178521086	+	.	.	.	.	.	.	.	.	.	.	.	.	.	.	.	.	.	.	.	.
2920	1	AGA	NR_033655	chr4	178588922	178600651	-	.	.	.	.	.	.	.	.	.	.	.	.	.	.	.	.	.	.	.	.
3540	1	DCTD	NM_001012732	chr4	184048237	184075624	-	.	.	.	.	.	.	.	.	.	.	.	.	.	.	.	.	.	.	.	.
2626	1,25	ING2	NM_001564	chr4	184663213	184669243	+	.	.	.	.	.	.	.	.	.	.	.	.	.	.	.	.	.	.	.	.
6984	0,5	STOX2	NM_020225	chr4	185063502	185175869	+	.	.	.	.	.	.	.	.	.	.	.	.	.	.	.	.	.	.	.	.
3684	1	ENPP6	NM_153343	chr4	185246852	185376108	-	.	.	.	.	.	.	.	.	.	.	.	.	.	.	.	.	.	.	.	.
539	3	CASP3	NM_004346	chr4	185785843	185807623	-	.	.	.	.	.	.	.	.	.	.	.	.	.	.	.	.	.	.	.	.
895	2,5	MLF1IP	NM_024629	chr4	185852212	185892280	-	.	.	.	.	.	.	.	.	.	.	.	.	.	.	.	.	.	.	.	.
1984	1,5	ACSL1	NM_001995	chr4	185913742	185984209	-	.	.	.	.	.	.	.	.	.	.	.	.	.	.	.	.	.	.	.	.
1813	2	TLR3	NM_003265	chr4	187227302	187243246	+	.	.	.	.	.	.	.	.	.	.	.	.	.	.	.	.	.	.	.	.
7311	0,25	FAT1	NM_005245	chr4	187745930	187881981	-	.	.	.	.	.	.	.	.	.	.	.	.	.	.	.	.	.	.	.	.
1372	2	DUX4	NM_033178	chr4	191229360	191230977	+	.	.	.	.	.	.	.	.	.	.	.	.	.	.	.	.	.	.	.	.
5202	1	SDHA	NM_004168	chr5	271355	309814	+	BH609520	chr5	274392	+	.	.	.	.	.	.	.	.	.	.	.	.	.	.	.	.
7468	0,25	PDCD6	NM_001267556	chr5	324735	368089	+	.	.	.	.	.	.	.	.	.	.	.	.	.	.	.	.	.	.	.	.
2495	1,25	AHRR	NM_001242412	chr5	357290	491405	+	.	.	.	.	AY516392	chr5	443557	-	.	.	.	.	.	.	.	.	.	.	.	.
2495	1,25	AHRR	NM_001242412	chr5	357290	491405	+	.	.	.	.	AY516699	chr5	484093	-	.	.	.	.	.	.	.	.	.	.	.	.
7043	0,5	TPPP	NM_007030	chr5	712976	746510	-	.	.	.	.	.	.	.	.	.	.	.	.	.	.	.	.	.	.	.	.
7118	0,5	ZDHHC11	NM_024786	chr5	848719	904101	-	.	.	.	.	.	.	.	.	.	.	.	.	78_1_2315_9	chr5	888723	+	.	.	.	.
84	6,25	TERT	NM_001193376	chr5	1306286	1348162	-	.	.	.	.	.	.	.	.	.	.	.	.	.	.	.	.	.	.	.	.
3395	1	CLPTM1L	NM_030782	chr5	1370999	1398002	-	.	.	.	.	.	.	.	.	.	.	.	.	.	.	.	.	.	.	.	.
5284	1	SLC6A3	NM_001044	chr5	1445904	1498543	-	.	.	.	.	.	.	.	.	.	.	.	.	.	.	.	.	.	.	.	.
4550	1	NDUFS6	NM_004553	chr5	1854495	1869167	+	.	.	.	.	.	.	.	.	.	.	.	.	.	.	.	.	.	.	.	.
4167	1	IRX2	NM_001134222	chr5	2799278	2804769	-	.	.	.	.	.	.	.	.	.	.	.	.	.	.	.	.	.	.	.	.
1773	2	SRD5A1	NM_001047	chr5	6686499	6722675	+	.	.	.	.	.	.	.	.	.	.	.	.	.	.	.	.	.	.	.	.
1216	2	ADCY2	NM_020546	chr5	7449342	7883194	+	.	.	.	.	.	.	.	.	.	.	.	.	49_1_2041_6	chr5	7531177	-	2083_182_1	chr5	7763581	-
1216	2	ADCY2	NM_020546	chr5	7449342	7883194	+	.	.	.	.	.	.	.	.	.	.	.	.	48_1_2023_6	chr5	7531179	-	2083_182_1	chr5	7763581	-
3741	1	FASTKD3	NM_024091	chr5	7912271	7922150	-	.	.	.	.	.	.	.	.	.	.	.	.	.	.	.	.	.	.	.	.
7440	0,25	MTRR	NM_024010	chr5	7922216	7954235	+	.	.	.	.	.	.	.	.	.	.	.	.	.	.	.	.	.	.	.	.
6869	0,5	SEMA5A	NM_003966	chr5	9088137	9599233	-	.	.	.	.	.	.	.	.	.	.	.	.	.	.	.	.	.	.	.	.
2057	1,5	CCT5	NM_012073	chr5	10303281	10319501	+	.	.	.	.	AY516775	chr5	10304097	+	.	.	.	.	.	.	.	.	.	.	.	.
3399	1	CMBL	NM_138809	chr5	10330706	10361168	-	.	.	.	.	.	.	.	.	.	.	.	.	.	.	.	.	.	.	.	.
812	2,5	CTNND2	NM_001332	chr5	11024951	11957110	-	AY517447.1	chr5	11514646	+	.	.	.	.	.	.	.	.	32_7_1611_5	chr5	11788310	-	.	.	.	.
812	2,5	CTNND2	NM_001332	chr5	11024951	11957110	-	AY517447.1	chr5	11514646	+	.	.	.	.	.	.	.	.	55_1_2084_7	chr5	11362606	-	.	.	.	.
1821	2	TRIO	NM_007118	chr5	14196828	14562458	+	.	.	.	.	.	.	.	.	.	.	.	.	.	.	.	.	.	.	.	.
3752	1	FBXL7	NM_012304	chr5	15553304	15992900	+	.	.	.	.	.	.	.	.	.	.	.	.	19_1_2569_13	chr5	15855916	-	.	.	.	.
6595	0,5	MARCH11	NM_001102562	chr5	16120473	16232897	-	.	.	.	.	.	.	.	.	.	.	.	.	.	.	.	.	.	.	.	.
6638	0,5	MYO10	NM_012334	chr5	16715015	16989385	-	.	.	.	.	AY516083	chr5	16970135	+	.	.	.	.	.	.	.	.	.	.	.	.
6638	0,5	MYO10	NM_012334	chr5	16715015	16989385	-	.	.	.	.	AY516409	chr5	16970500	+	.	.	.	.	.	.	.	.	.	.	.	.
6638	0,5	MYO10	NM_012334	chr5	16715015	16989385	-	.	.	.	.	AY516813	chr5	16948862	+	.	.	.	.	.	.	.	.	.	.	.	.
6564	0,5	LOC285696	NR_027253	chr5	17183136	17270531	-	.	.	.	.	.	.	.	.	.	.	.	.	.	.	.	.	.	.	.	.
2023	1,5	BASP1	NM_006317	chr5	17270749	17329943	+	.	.	.	.	AY516218	chr5	17295907	-	.	.	.	.	.	.	.	.	.	.	.	.
6162	0,5	CDH18	NM_001167667	chr5	19508911	20024110	-	.	.	.	.	.	.	.	.	.	.	.	.	.	.	.	.	.	.	.	.

Table S2

tumor associated genes								HIV				MLV				MMTV				MMTV(SIN)				MMTV(SIN)arrest			
rank	score	gene symbol	RefSeq	chrom	txStart	txEnd	strand	integrant	chr	position	strand	integrant	chr	position	strand	integrant	chr	position	strand	integrant	chr	position	strand	integrant	chr	position	strand
6161	0,5	CDH12	NM_004061	chr5	21786729	22889488	-	.	.	.	.	AY516463	chr5	22796568	+	_399_1_240	chr5	21903523	+	.	.	.	.	.	.	.	.
4934	1	PRDM9	NM_020227	chr5	23543480	23564463	+	.	.	.	.	.	.	.	.	.	.	.	.	.	.	.	.	.	.	.	.
2064	1,5	CDH10	NM_006727	chr5	24522965	24680842	-	.	.	.	.	.	.	.	.	.	.	.	.	.	.	.	.	.	.	.	.
3312	1	CDH9	NM_016279	chr5	26916465	27074446	-	CL799744	chr5	26941912	-	.	.	.	.	.	.	.	.	.	.	.	.	.	.	.	.
5890	0,75	CDH6	NM_004932	chr5	31229518	31365010	+	.	.	.	.	.	.	.	.	.	.	.	.	.	.	.	.	.	.	.	.
2317	1,5	PDZD2	NM_178140	chr5	31834787	32146795	+	.	.	.	.	.	.	.	.	_452_1_310	chr5	32087941	-	.	.	.	.	.	.	.	.
3934	1	GOLPH3	NM_022130	chr5	32160580	32210182	-	CL800096	chr5	32195103	+	.	.	.	.	.	.	.	.	.	.	.	.	2514_217_3	chr5	32196399	+
3934	1	GOLPH3	NM_022130	chr5	32160580	32210182	-	CL528931	chr5	32195707	-	.	.	.	.	.	.	.	.	.	.	.	.	2514_217_3	chr5	32196399	+
2431	1,5	TARS	NM_001258437	chr5	33476558	33503953	+	.	.	.	.	.	.	.	.	.	.	.	.	.	.	.	.	.	.	.	.
520	3	ADAMTS12	NM_030955	chr5	33563043	33927881	-	.	.	.	.	.	.	.	.	.	.	.	.	.	.	.	.	770_119_1	chr5	33649746	+
2973	1	AMACR	NM_001167595	chr5	34022847	34043977	-	.	.	.	.	.	.	.	.	.	.	.	.	.	.	.	.	.	.	.	.
2740	1,25	RAD1	NR_026591	chr5	34941122	34951488	-	.	.	.	.	.	.	.	.	.	.	.	.	.	.	.	.	.	.	.	.
2931	1	AGXT2	NM_031900	chr5	35033962	35083997	-	.	.	.	.	.	.	.	.	.	.	.	.	.	.	.	.	.	.	.	.
1080	2,25	IL7R	NM_002185	chr5	35892733	35915462	+	.	.	.	.	AY516240	chr5	35893123	-	.	.	.	.	.	.	.	.	.	.	.	.
192	4,5	SKP2	NM_005983	chr5	36187901	36219899	+	.	.	.	.	.	.	.	.	.	.	.	.	.	.	.	.	.	.	.	.
6906	0,5	SLC1A3	NM_001166696	chr5	36642213	36645156	+	.	.	.	.	.	.	.	.	.	.	.	.	.	.	.	.	.	.	.	.
906	2,5	NIPBL	NM_133433	chr5	36912617	37101678	+	CL799831	chr5	36957041	+	.	.	.	.	.	.	.	.	.	.	.	.	.	.	.	.
906	2,5	NIPBL	NM_133433	chr5	36912617	37101678	+	BH609517	chr5	36970909	+	.	.	.	.	.	.	.	.	.	.	.	.	.	.	.	.
3234	1	C5orf42	NM_023073	chr5	37142086	37285287	-	.	.	.	.	.	.	.	.	.	.	.	.	.	.	.	.	.	.	.	.
6690	0,5	NUP155	NM_004298	chr5	37327697	37406644	-	.	.	.	.	.	.	.	.	.	.	.	.	.	.	.	.	.	.	.	.
3657	1	EGFLAM	NM_152403	chr5	38294267	38501339	+	.	.	.	.	.	.	.	.	.	.	.	.	.	.	.	.	.	.	.	.
480	3,25	LIFR	NM_001127671	chr5	38510821	38592505	-	.	.	.	.	.	.	.	.	.	.	.	.	.	.	.	.	.	.	.	.
1591	2	OSMR	NM_001168355	chr5	38881716	38922516	+	.	.	.	.	.	.	.	.	.	.	.	.	.	.	.	.	.	.	.	.
668	3	RICTOR	NM_152756	chr5	38973779	39110258	-	.	.	.	.	.	.	.	.	.	.	.	.	.	.	.	.	.	.	.	.
7319	0,25	FYB	NM_001465	chr5	39141110	39255437	-	.	.	.	.	AY515913	chr5	39154060	+	.	.	.	.	.	.	.	.	.	.	.	.
367	3,5	DAB2	NM_001343	chr5	39407532	39461092	-	.	.	.	.	.	.	.	.	.	.	.	.	.	.	.	.	.	.	.	.
6782	0,5	PTGER4	NM_000958	chr5	40715788	40729594	+	.	.	.	.	.	.	.	.	.	.	.	.	.	.	.	.	.	.	.	.
1681	2	PRKAA1	NM_006251	chr5	40795237	40834054	-	CL800340	chr5	40809679	-	.	.	.	.	.	.	.	.	.	.	.	.	.	.	.	.
3277	1	CARD6	NM_032587	chr5	40877166	40891213	+	.	.	.	.	.	.	.	.	.	.	.	.	.	.	.	.	.	.	.	.
2325	1,5	PLCXD3	NM_001005473	chr5	41342804	41546487	-	.	.	.	.	.	.	.	.	.	.	.	.	.	.	.	.	.	.	.	.
2301	1,5	OXCT1	NM_000436	chr5	41765923	41906548	-	CL800631	chr5	41792440	-	AY516844	chr5	41906156	+	.	.	.	.	.	.	.	.	.	.	.	.
2301	1,5	OXCT1	NM_000436	chr5	41765923	41906548	-	CL529255	chr5	41895656	-	AY516844	chr5	41906156	+	.	.	.	.	.	.	.	.	.	.	.	.
2301	1,5	OXCT1	NM_000436	chr5	41765923	41906548	-	CL528934	chr5	41806235	+	AY516844	chr5	41906156	+	.	.	.	.	.	.	.	.	.	.	.	.
3758	1	FBXO4	NM_033484	chr5	41961112	41970942	+	.	.	.	.	.	.	.	.	.	.	.	.	.	.	.	.	.	.	.	.
845	2,5	GHR	NM_001242399	chr5	42460310	42757737	+	.	.	.	.	.	.	.	.	_305_1_194	chr5	42521135	+	25_1_1389_4!	chr5	42552002	+	.	.	.	.
5209	1	SEPP1	NM_005410	chr5	42835738	42847781	-	.	.	.	.	.	.	.	.	.	.	.	.	.	.	.	.	.	.	.	.
4059	1	HMGCS1	NM_002130	chr5	43323328	43349371	-	.	.	.	.	.	.	.	.	.	.	.	.	.	.	.	.	.	.	.	.
4594	1	NNT	NM_182977	chr5	43638985	43741425	+	AY516960.1	chr5	43715191	+	.	.	.	.	.	.	.	.	.	.	.	.	.	.	.	.
6425	0,5	HCN1	NM_021072	chr5	45290808	45731977	-	.	.	.	.	.	.	.	.	.	.	.	.	1053_181	chr5	45508598	-	.	.	.	.
6717	0,5	PARP8	NM_001178055	chr5	49997489	50178113	+	CL800186	chr5	50110717	-	AY515925	chr5	50005533	+	.	.	.	.	.	.	.	.	394_101_2	chr5	50116460	+
6485	0,5	ISL1	NM_002202	chr5	50714714	50726320	+	.	.	.	.	.	.	.	.	.	.	.	.	.	.	.	.	.	.	.	.
2629	1,25	ITGA2	NM_002203	chr5	52320912	52426366	+	CL529568	chr5	52398844	+	.	.	.	.	.	.	.	.	.	.	.	.	.	.	.	.
6364	0,5	FST	NM_013409	chr5	52812020	52818061	+	.	.	.	.	.	.	.	.	.	.	.	.	.	.	.	.	.	.	.	.
4548	1	NDUFS4	NM_002495	chr5	52892221	53014928	+	CL800486	chr5	52922491	-	.	.	.	.	.	.	.	.	.	.	.	.	.	.	.	.
6454	0,5	HSPB3	NM_006308	chr5	53787187	53787971	+	.	.	.	.	.	.	.	.	.	.	.	.	.	.	.	.	.	.	.	.
6322	0,5	ESM1	NM_001135604	chr5	54309451	54317171	-	.	.	.	.	.	.	.	.	.	.	.	.	.	.	.	.	.	.	.	.
5250	1	SKIV2L2	NM_015360	chr5	54639332	54757166	+	BH609522	chr5	54711889	-	.	.	.	.	.	.	.	.	.	.	.	.	.	.	.	.
1663	2	PPAP2A	NM_003711	chr5	54756439	54866630	-	.	.	.	.	.	.	.	.	.	.	.	.	.	.	.	.	.	.	.	.
1349	2	DDX4	NM_001166534	chr5	55098469	55148731	+	.	.	.	.	.	.	.	.	.	.	.	.	.	.	.	.	.	.	.	.
866	2,5	IL6ST	NM_001190981	chr5	55266681	55326578	-	.	.	.	.	.	.	.	.	.	.	.	.	.	.	.	.	.	.	.	.
335	3,75	MAP3K1	NM_005921	chr5	56146656	56227735	+	.	.	.	.	.	.	.	.	.	.	.	.	.	.	.	.	.	.	.	.

Table S2

tumor associated genes								HIV				MLV				MMTV				MMTV(SIN)				MMTV(SIN)arrest			
rank	score	gene symbol	RefSEq	chrom	txStart	txEnd	strand	integrant	chr	position	strand	integrant	chr	position	strand	integrant	chr	position	strand	integrant	chr	position	strand	integrant	chr	position	strand
4843	1	PLK2	NM_001252226	chr5	57785566	57791723	-	.	.	.	.	.	.	.	.	.	.	.	.	.	.	.	.	.	.	.	.
5034	1	RAB3C	NM_138453	chr5	57914695	58183163	+	.	.	.	.	.	.	.	.	.	.	.	.	.	.	.	.	.	.	.	.
1605	2	PDE4D	NM_001197218	chr5	58300622	59100195	-	.	.	.	.	AY515917	chr5	58938378	+	.	.	.	.	110_1_953_29	chr5	59353824	+	.	.	.	.
1605	2	PDE4D	NM_001197218	chr5	58300622	59100195	-	.	.	.	.	AY515972	chr5	58687002	-	.	.	.	.	110_1_953_29	chr5	59353824	+	.	.	.	.
6718	0,5	PART1	NR_028509	chr5	59819515	59858002	+	.	.	.	.	.	.	.	.	.	.	.	.	.	.	.	.	2042_179_2	chr5	59832001	+
6263	0,5	DEPDC1B	NM_018369	chr5	59928495	60031750	-	.	.	.	.	.	.	.	.	.	.	.	.	.	.	.	.	.	.	.	.
1388	2	ERCC8	NM_000082	chr5	60205415	60276662	-	.	.	.	.	.	.	.	.	.	.	.	.	.	.	.	.	.	.	.	.
1476	2	IPO11	NM_016338	chr5	61744329	61960172	+	.	.	.	.	.	.	.	.	.	.	.	.	.	.	.	.	.	.	.	.
2071	1,5	CENPK	NM_022145	chr5	64849348	64894751	-	CL529520	chr5	64851382	-	.	.	.	.	.	.	.	.	78_1_2316_9	chr5	64879754	+	.	.	.	.
5550	1	TRIM23	NM_001656	chr5	64921262	64955943	-	.	.	.	.	.	.	.	.	.	.	.	.	.	.	.	.	.	.	.	.
5907	0,75	ERBB2IP	NM_018695	chr5	65258137	65412607	+	.	.	.	.	.	.	.	.	.	.	.	.	.	.	.	.	.	.	.	.
4361	1	MAST4	NM_198828	chr5	65927931	66318107	+	.	.	.	.	.	.	.	.	.	.	.	.	.	.	.	.	.	.	.	.
31	8,5	PIK3R1	NM_181523	chr5	67547339	67633405	+	CL528938	chr5	67632683	-	.	.	.	.	.	.	.	.	.	.	.	.	.	.	.	.
31	8,5	PIK3R1	NM_181523	chr5	67547339	67633405	+	CL529439	chr5	67591759	+	.	.	.	.	.	.	.	.	.	.	.	.	.	.	.	.
2055	1,5	CCNB1	NM_031966	chr5	68498592	68509826	+	.	.	.	.	.	.	.	.	.	.	.	.	.	.	.	.	.	.	.	.
3331	1	CENPH	NM_022909	chr5	68521130	68541940	+	.	.	.	.	.	.	.	.	.	.	.	.	.	.	.	.	.	.	.	.
242	4	CDK7	NM_001799	chr5	68566377	68609013	+	.	.	.	.	.	.	.	.	.	.	.	.	.	.	.	.	.	.	.	.
1971	1,75	TAF9	NM_001015892	chr5	68696325	68701596	-	.	.	.	.	.	.	.	.	.	.	.	.	.	.	.	.	.	.	.	.
2741	1,25	RAD17	NM_133340	chr5	68702617	68746387	+	.	.	.	.	.	.	.	.	.	.	.	.	.	.	.	.	.	.	.	.
4656	1	OCLN	NM_001205255	chr5	68835759	68889687	+	.	.	.	.	AY516786	chr5	68857960	-	.	.	.	.	.	.	.	.	2000_177_1	chr5	68853939	+
3986	1	GTF2H2	NM_001515	chr5	68891809	68924334	+	.	.	.	.	.	.	.	.	.	.	.	.	.	.	.	.	.	.	.	.
6938	0,5	SMN1	NM_000344	chr5	69381105	69409174	+	.	.	.	.	.	.	.	.	.	.	.	.	.	.	.	.	.	.	.	.
4369	1	MCCC2	NM_022132	chr5	70918870	70990286	+	.	.	.	.	.	.	.	.	.	.	.	.	.	.	.	.	.	.	.	.
3279	1	CARTPT	NM_004291	chr5	71050745	71052631	+	.	.	.	.	.	.	.	.	.	.	.	.	.	.	.	.	.	.	.	.
4342	1	MAP1B	NM_005909	chr5	71438873	71541153	+	.	.	.	.	.	.	.	.	.	.	.	.	.	.	.	.	.	.	.	.
5517	1	TNPO1	NM_002270	chr5	72148173	72245971	+	.	.	.	.	.	.	.	.	.	.	.	.	.	.	.	.	.	.	.	.
5492	1	TMEM171	NM_001161342	chr5	72452143	72463400	+	.	.	.	.	.	.	.	.	.	.	.	.	.	.	.	.	.	.	.	.
6309	0,5	ENC1	NM_003633	chr5	73958986	73973005	-	.	.	.	.	.	.	.	.	.	.	.	.	.	.	.	.	.	.	.	.
1918	1,75	HEXB	NM_000521	chr5	74016724	74052869	+	.	.	.	.	.	.	.	.	.	.	.	.	.	.	.	.	.	.	.	.
3871	1	GCNT4	NM_016591	chr5	74359044	74362480	-	.	.	.	.	.	.	.	.	.	.	.	.	.	.	.	.	.	.	.	.
4058	1	HMGCR	NM_000859	chr5	74668748	74693682	+	.	.	.	.	.	.	.	.	.	.	.	.	.	.	.	.	.	.	.	.
4865	1	POLK	NM_016218	chr5	74843412	74931402	+	CL800094	chr5	74872691	-	.	.	.	.	.	.	.	.	47_1_2013_6	chr5	74929003	+	.	.	.	.
3708	1	F2RL2	NM_001256566	chr5	75947062	75954994	-	.	.	.	.	.	.	.	.	.	.	.	.	.	.	.	.	.	.	.	.
1906	1,75	F2R	NM_001992	chr5	76047623	76067351	+	.	.	.	.	.	.	.	.	.	.	.	.	.	.	.	.	.	.	.	.
4739	1	PDE8B	NM_003719	chr5	76542461	76759836	+	.	.	.	.	.	.	.	.	.	.	.	.	459_119	chr5	76712070	+	.	.	.	.
6848	0,5	SCAMP1	NM_004866	chr5	77692094	77812318	+	.	.	.	.	.	.	.	.	.	.	.	.	.	.	.	.	.	.	.	.
6557	0,5	LHFPL2	NM_005779	chr5	77816793	77980404	-	.	.	.	.	.	.	.	.	.	.	.	.	.	.	.	.	.	.	.	.
2504	1,25	ARSB	NM_198709	chr5	78147089	78317522	-	.	.	.	.	.	.	.	.	.	.	.	.	1535_205	chr5	78163370	-	.	.	.	.
3591	1	DMGDH	NM_013391	chr5	78329184	78401205	-	.	.	.	.	.	.	.	.	.	.	.	.	.	.	.	.	.	.	.	.
3147	1	BHMT2	NM_017614	chr5	78401302	78421653	+	.	.	.	.	.	.	.	.	.	.	.	.	.	.	.	.	.	.	.	.
3146	1	BHMT	NM_001713	chr5	78443359	78463869	+	.	.	.	.	.	.	.	.	.	.	.	.	.	.	.	.	.	.	.	.
3402	1	CMYA5	NM_153610	chr5	79021414	79131805	+	.	.	.	.	.	.	.	.	.	.	.	.	.	.	.	.	.	.	.	.
7018	0,5	THBS4	NM_003248	chr5	79366925	79414863	+	.	.	.	.	.	.	.	.	.	.	.	.	.	.	.	.	.	.	.	.
1354	2	DHFR	NM_000791	chr5	79957800	79986556	-	.	.	.	.	.	.	.	.	.	.	.	.	.	.	.	.	.	.	.	.
1544	2	MSH3	NM_002439	chr5	79986222	80208390	+	.	.	.	.	AY516272	chr5	80204900	+	.	.	.	.	.	.	.	.	540_109_1	chr5	80022750	-
946	2,5	RASGRF2	NM_006909	chr5	80292263	80561737	+	.	.	.	.	.	.	.	.	.	.	.	.	.	.	.	.	.	.	.	.
2076	1,5	CKMT2	NM_001099736	chr5	80564894	80597973	+	.	.	.	.	.	.	.	.	.	.	.	.	.	.	.	.	.	.	.	.
2883	1	ACOT12	NM_130767	chr5	80661702	80725744	-	.	.	.	.	.	.	.	.	.	.	.	.	.	.	.	.	.	.	.	.
2462	1,5	XRCC4	NM_022550	chr5	82409072	82685335	+	.	.	.	.	.	.	.	.	.	.	.	.	.	.	.	.	.	.	.	.
5671	1	VCAN	NM_001164097	chr5	82803248	82913878	+	.	.	.	.	.	.	.	.	.	.	.	.	.	.	.	.	.	.	.	.
2612	1,25	HAPLN1	NM_001884	chr5	82969772	83052652	-	.	.	.	.	.	.	.	.	.	.	.	.	.	.	.	.	.	.	.	.

Table S2

tumor associated genes								HIV				MLV				MMTV				MMTV(SIN)				MMTV(SIN)arrest			
rank	score	gene symbol	RefSEq	chrom	txStart	txEnd	strand	integrant	chr	position	strand	integrant	chr	position	strand	integrant	chr	position	strand	integrant	chr	position	strand	integrant	chr	position	strand
818	2,5	EDIL3	NM_005711	chr5	83273881	83716367	-	.	.	.	.	.	.	.	.	.	.	.	.	.	.	.	.	.	.	.	.
3442	1	COX7C	NM_001867	chr5	85949539	85952339	+	.	.	.	.	.	.	.	.	.	.	.	.	.	.	.	.	.	.	.	.
1153	2,25	RASA1	NM_022650	chr5	86600496	86723499	+	CL528940	chr5	86702733	-	.	.	.	.	.	.	.	.	.	.	.	.	.	.	.	.
3290	1	CCNH	NM_001239	chr5	86725834	86744606	-	.	.	.	.	.	.	.	.	.	.	.	.	.	.	.	.	.	.	.	.
1530	2	MEF2C	NM_001193350	chr5	88049813	88215039	-	.	.	.	.	.	.	.	.	.	.	.	.	.	.	.	.	.	.	.	.
1659	2	POLR3G	NM_006467	chr5	89806436	89846125	+	.	.	.	.	.	.	.	.	.	.	.	.	.	.	.	.	.	.	.	.
6404	0,5	GPR98	NR_003149	chr5	89890372	90495789	+	BH609526	chr5	90125810	-	.	.	.	.	.	.	.	.	.	.	.	.	.	.	.	.
4614	1	NR2F1	NM_005654	chr5	92944798	92955542	+	.	.	.	.	.	.	.	.	.	.	.	.	.	.	.	.	.	.	.	.
2249	1,5	MCTP1	NM_001002796	chr5	94068046	94443301	-	.	.	.	.	.	.	.	.	_329_1_204	chr5	94641170	-	.	.	.	.	.	.	.	.
3950	1	GPR150	NM_199243	chr5	94981735	94983040	+	.	.	.	.	.	.	.	.	.	.	.	.	.	.	.	.	.	.	.	.
5117	1	RHOBTB3	NM_014899	chr5	95092605	95157827	+	.	.	.	.	.	.	.	.	.	.	.	.	.	.	.	.	.	.	.	.
5942	0,75	PCSK1	NM_001177875	chr5	95751795	95793619	-	.	.	.	.	.	.	.	.	.	.	.	.	.	.	.	.	.	.	.	.
2052	1,5	CAST	NM_173060	chr5	96064248	96136141	+	.	.	.	.	.	.	.	.	.	.	.	.	.	.	.	.	.	.	.	.
6320	0,5	ERAP2	NM_022350	chr5	96237399	96281162	+	.	.	.	.	.	.	.	.	.	.	.	.	.	.	.	.	.	.	.	.
5123	1	RIOK2	NM_001159749	chr5	96528206	96544761	-	.	.	.	.	.	.	.	.	.	.	.	.	.	.	.	.	.	.	.	.
7560	0,25	SLCO6A1	NM_173488	chr5	101735550	101862619	-	.	.	.	.	.	.	.	.	.	.	.	.	.	.	.	.	.	.	.	.
4644	1	NUDT12	NM_031438	chr5	102912454	102926389	-	.	.	.	.	.	.	.	.	.	.	.	.	.	.	.	.	.	.	.	.
1909	1,75	FER	NM_005246	chr5	108111421	108551272	+	.	.	.	.	.	.	.	.	.	.	.	.	.	.	.	.	.	.	.	.
7427	0,25	MIR548C	NR_030347	chr5	109049179	109225081	+	.	.	.	.	.	.	.	.	.	.	.	.	.	.	.	.	.	.	.	.
6592	0,5	MAN2A1	NM_002372	chr5	109053054	109231328	+	.	.	.	.	.	.	.	.	.	.	.	.	.	.	.	.	.	.	.	.
1007	2,25	CAMK4	NM_001744	chr5	110587845	110848647	+	AY517064.1	chr5	110744338	+	.	.	.	.	.	.	.	.	.	.	.	.	.	.	.	.
6312	0,5	EPB41L4A	NM_022140	chr5	111526213	111782909	-	.	.	.	.	.	.	.	.	.	.	.	.	.	.	.	.	.	.	.	.
6356	0,5	FLJ11235	NR_027706	chr5	111783178	111784574	+	.	.	.	.	.	.	.	.	.	.	.	.	.	.	.	.	.	.	.	.
13	10,25	APC	NM_000038	chr5	112101454	112209835	+	CL799870	chr5	112142346	-	.	.	.	.	.	.	.	.	.	.	.	.	.	.	.	.
2751	1,25	REEP5	NM_005669	chr5	112239979	112285930	-	.	.	.	.	.	.	.	.	.	.	.	.	.	.	.	.	.	.	.	.
6601	0,5	MCC	NM_002387	chr5	112385694	112658511	-	.	.	.	.	.	.	.	.	.	.	.	.	.	.	.	.	.	.	.	.
5574	1	TSSK1B	NM_032028	chr5	112796149	112798627	-	.	.	.	.	.	.	.	.	.	.	.	.	.	.	.	.	.	.	.	.
1819	2	TRIM36	NM_001017398	chr5	114541134	114544142	-	.	.	.	.	.	.	.	.	.	.	.	.	.	.	.	.	.	.	.	.
4771	1	PGGT1B	NM_005023	chr5	114574425	114626468	-	.	.	.	.	.	.	.	.	.	.	.	.	.	.	.	.	.	.	.	.
3320	1	CDO1	NM_001801	chr5	115168328	115180304	-	.	.	.	.	.	.	.	.	.	.	.	.	.	.	.	.	.	.	.	.
6870	0,5	SEMA6A	NM_020796	chr5	115807149	115938450	-	.	.	.	.	.	.	.	.	.	.	.	.	.	.	.	.	.	.	.	.
7037	0,5	TNFAIP8	NM_014350	chr5	118719494	118758193	+	CL529305	chr5	118711335	-	.	.	.	.	.	.	.	.	.	.	.	.	.	.	.	.
1455	2	HSD17B4	NM_001199291	chr5	118816100	118905929	+	.	.	.	.	.	.	.	.	.	.	.	.	.	.	.	.	139_83_1	chr5	118890002	+
4957	1	PRR16	NM_016644	chr5	119827917	120050863	+	CL528947	chr5	119956240	+	.	.	.	.	.	.	.	.	.	.	.	.	.	.	.	.
3811	1	FTMT	NM_177478	chr5	121215548	121216422	+	.	.	.	.	.	.	.	.	.	.	.	.	.	.	.	.	.	.	.	.
395	3,5	LOX	NM_001178102	chr5	121426788	121440817	-	.	.	.	.	.	.	.	.	.	.	.	.	.	.	.	.	.	.	.	.
6948	0,5	SNX2	NM_003100	chr5	122138648	122193701	+	.	.	.	.	.	.	.	.	.	.	.	.	.	.	.	.	.	.	.	.
3471	1	CSNK1G3	NM_001270573	chr5	122875691	122980637	+	AY516909.1	chr5	122919471	+	.	.	.	.	.	.	.	.	.	.	.	.	.	.	.	.
3471	1	CSNK1G3	NM_001270573	chr5	122875691	122980637	+	CL529242	chr5	122906002	+	.	.	.	.	.	.	.	.	.	.	.	.	.	.	.	.
1872	2	ZNF608	NM_020747	chr5	124000508	124108704	-	.	.	.	.	.	.	.	.	.	.	.	.	.	.	.	.	.	.	.	.
528	3	ALDH7A1	NM_001182	chr5	125905431	125958981	-	.	.	.	.	.	.	.	.	.	.	.	.	.	.	.	.	.	.	.	.
4292	1	LMNB1	NM_005573	chr5	126140213	126200611	+	.	.	.	.	.	.	.	.	.	.	.	.	.	.	.	.	.	.	.	.
6230	0,5	CTXN3	NM_001127385	chr5	127016605	127022221	+	.	.	.	.	.	.	.	.	.	.	.	.	.	.	.	.	.	.	.	.
830	2,5	FBN2	NM_001999	chr5	127621499	127901634	-	CL528949	chr5	127795889	+	.	.	.	.	.	.	.	.	.	.	.	.	.	.	.	.
3367	1	CHSY3	NM_175856	chr5	129268421	129550226	+	.	.	.	.	.	.	.	.	.	.	.	.	.	.	.	.	.	.	.	.
352	3,5	ACSL6	NM_001205247	chr5	131313565	131375254	-	.	.	.	.	.	.	.	.	.	.	.	.	.	.	.	.	.	.	.	.
476	3,25	IL3	NM_000588	chr5	131424245	131426795	+	.	.	.	.	.	.	.	.	.	.	.	.	.	.	.	.	.	.	.	.
1018	2,25	CSF2	NM_000758	chr5	131437383	131439762	+	.	.	.	.	.	.	.	.	.	.	.	.	.	.	.	.	.	.	.	.
2302	1,5	P4HA2	NM_001142598	chr5	131556202	131591455	-	.	.	.	.	.	.	.	.	.	.	.	.	.	.	.	.	.	.	.	.
6728	0,5	PDLIM4	NM_003687	chr5	131621249	131637046	+	.	.	.	.	.	.	.	.	.	.	.	.	.	.	.	.	.	.	.	.
6483	0,5	IRF1	NM_002198	chr5	131845199	131854364	-	.	.	.	.	.	.	.	.	.	.	.	.	.	.	.	.	.	.	.	.

Table S2

tumor associated genes								HIV				MLV				MMTV				MMTV(SIN)				MMTV(SIN)arrest			
rank	score	gene symbol	RefSEq	chrom	txStart	txEnd	strand	integrant	chr	position	strand	integrant	chr	position	strand	integrant	chr	position	strand	integrant	chr	position	strand	integrant	chr	position	strand
2625	1,25	IL5	NM_000879	chr5	131905034	131907113	-	.	.	.	.	.	.	.	.	.	.	.	.	.	.	.	.	.	.	.	.
112	5,5	RAD50	NM_005732	chr5	131920514	132008212	+	CL529684	chr5	131945962	-	.	.	.	.	.	.	.	.	.	.	.	.	.	.	.	.
112	5,5	RAD50	NM_005732	chr5	131920514	132008212	+	BH609529	chr5	131959142	-	.	.	.	.	.	.	.	.	.	.	.	.	.	.	.	.
863	2,5	IL13	NM_002188	chr5	132021763	132024700	+	.	.	.	.	.	.	.	.	.	.	.	.	.	.	.	.	.	.	.	.
2202	1,5	IL4	NM_172348	chr5	132037576	132046269	+	.	.	.	.	.	.	.	.	.	.	.	.	.	.	.	.	.	.	.	.
5648	1	UQCRQ	NM_014402	chr5	132230217	132232435	+	.	.	.	.	.	.	.	.	.	.	.	.	.	.	.	.	.	.	.	.
776	2,5	AFF4	NM_014423	chr5	132238969	132327253	-	.	.	.	.	.	.	.	.	.	.	.	.	.	.	.	.	.	.	.	.
7117	0,5	ZCCHC10	NM_017665	chr5	132360575	132390139	-	.	.	.	.	.	.	.	.	.	.	.	.	.	.	.	.	.	.	.	.
474	3,25	HSPA4	NM_002154	chr5	132415560	132468608	+	.	.	.	.	.	.	.	.	.	.	.	.	.	.	.	.	.	.	.	.
6365	0,5	FSTL4	NM_015082	chr5	132560050	132976122	-	.	.	.	.	.	.	.	.	.	.	.	.	.	.	.	.	1063_128_3	chr5	132690984	-
5446	1	TCF7	NR_033449	chr5	133479248	133511819	+	.	.	.	.	.	.	.	.	.	.	.	.	.	.	.	.	.	.	.	.
5251	1	SKP1	NM_170679	chr5	133519980	133540623	-	.	.	.	.	.	.	.	.	.	.	.	.	.	.	.	.	.	.	.	.
1140	2,25	PPP2CA	NM_002715	chr5	133560046	133589849	-	AY517316.1	chr5	133583128	+	.	.	.	.	.	.	.	.	.	.	.	.	.	.	.	.
3318	1	CDKL3	NM_016508	chr5	133671590	133730664	-	.	.	.	.	.	.	.	.	.	.	.	.	.	.	.	.	.	.	.	.
697	3	UBE2B	NM_003337	chr5	133734768	133755698	+	.	.	.	.	.	.	.	.	.	.	.	.	.	.	.	.	.	.	.	.
7478	0,25	PITX1	NM_002653	chr5	134391322	134397863	-	.	.	.	.	.	.	.	.	.	.	.	.	.	.	.	.	.	.	.	.
2561	1,25	CXCL14	NM_004887	chr5	134934267	134942868	-	.	.	.	.	.	.	.	.	.	.	.	.	.	.	.	.	.	.	.	.
3748	1	FBXL21	NM_012159	chr5	135293904	135305266	+	.	.	.	.	.	.	.	.	.	.	.	.	.	.	.	.	.	.	.	.
509	3,25	TGFBI	NM_000358	chr5	135392482	135427406	+	.	.	.	.	.	.	.	.	.	.	.	.	.	.	.	.	.	.	.	.
6935	0,5	SMAD5	NM_001001420	chr5	135496434	135546321	+	.	.	.	.	.	.	.	.	.	.	.	.	.	.	.	.	.	.	.	.
5341	1	SPOCK1	NM_004598	chr5	136338885	136862917	-	.	.	.	.	.	.	.	.	.	.	.	.	.	.	.	.	1799_166_1	chr5	136402782	-
6539	0,5	KLHL3	NM_001257194	chr5	136981087	137084898	-	.	.	.	.	.	.	.	.	.	.	.	.	.	.	.	.	.	.	.	.
5706	1	WNT8A	NM_058244	chr5	137447672	137455098	+	.	.	.	.	.	.	.	.	.	.	.	.	.	.	.	.	.	.	.	.
2028	1,5	BRD8	NM_001164326	chr5	137520471	137542257	-	.	.	.	.	.	.	.	.	.	.	.	.	.	.	.	.	.	.	.	.
4229	1	KIF20A	NM_005733	chr5	137542315	137551303	+	.	.	.	.	.	.	.	.	.	.	.	.	.	.	.	.	.	.	.	.
3305	1	CDC23	NM_004661	chr5	137551235	137576931	-	.	.	.	.	.	.	.	.	.	.	.	.	.	.	.	.	.	.	.	.
450	3,25	CDC25C	NM_001790	chr5	137648857	137695415	-	.	.	.	.	.	.	.	.	.	.	.	.	.	.	.	.	.	.	.	.
4207	1	KDM3B	NM_016604	chr5	137716183	137800615	+	.	.	.	.	.	.	.	.	.	.	.	.	.	.	.	.	.	.	.	.
3661	1	EGR1	NM_001964	chr5	137829079	137832903	+	.	.	.	.	.	.	.	.	.	.	.	.	.	.	.	.	.	.	.	.
4095	1	HSPA9	NM_004134	chr5	137918469	137939217	-	.	.	.	.	.	.	.	.	.	.	.	.	.	.	.	.	.	.	.	.
246	4	CTNNA1	NM_001903	chr5	138117005	138298622	+	CL800497	chr5	138194673	-	.	.	.	.	.	.	.	.	.	.	.	.	.	.	.	.
6597	0,5	MATR3	NM_001194955	chr5	138657337	138695265	+	BH609531	chr5	138676671	+	.	.	.	.	.	.	.	.	.	.	.	.	.	.	.	.
1831	2	UBE2D2	NM_003339	chr5	138920934	138988202	+	.	.	.	.	.	.	.	.	.	.	.	.	.	.	.	.	.	.	.	.
6779	0,5	PSD2	NM_032289	chr5	139155589	139204232	+	.	.	.	.	.	.	.	.	.	.	.	.	.	.	.	.	.	.	.	.
4620	1	NRG2	NM_001184935	chr5	139206547	139403068	-	.	.	.	.	.	.	.	.	.	.	.	.	.	.	.	.	.	.	.	.
4021	1	HBEGF	NM_001945	chr5	139692611	139706372	-	.	.	.	.	.	.	.	.	.	.	.	.	.	.	.	.	.	.	.	.
2985	1	ANKHD1	NM_024668	chr5	139761582	139832246	+	CL800149	chr5	139874726	+	.	.	.	.	.	.	.	.	.	.	.	.	.	.	.	.
5347	1	SRA1	NM_001253764	chr5	139909835	139917225	-	.	.	.	.	.	.	.	.	.	.	.	.	.	.	.	.	.	.	.	.
6147	0,5	CD14	NM_000591	chr5	139991496	139993219	-	.	.	.	.	.	.	.	.	.	.	.	.	.	.	.	.	.	.	.	.
4526	1	NDUFA2	NM_002488	chr5	140005131	140007554	-	.	.	.	.	.	.	.	.	.	.	.	.	.	.	.	.	.	.	.	.
4014	1	HARS	NM_001258040	chr5	140033673	140051496	-	.	.	.	.	.	.	.	.	.	.	.	.	.	.	.	.	.	.	.	.
4015	1	HARS2	NM_012208	chr5	140051201	140059074	+	.	.	.	.	.	.	.	.	.	.	.	.	.	.	.	.	.	.	.	.
4710	1	PCDHA9	NM_014005	chr5	140207540	140213928	+	.	.	.	.	.	.	.	.	.	.	.	.	.	.	.	.	.	.	.	.
6720	0,5	PCDHAC2	NM_031883	chr5	140326535	140329190	+	.	.	.	.	.	.	.	.	.	.	.	.	.	.	.	.	.	.	.	.
4712	1	PCDHB2	NM_018936	chr5	140454420	140457148	+	.	.	.	.	.	.	.	.	.	.	.	.	.	.	.	.	.	.	.	.
4713	1	PCDHB3	NM_018937	chr5	140460417	140463590	+	.	.	.	.	.	.	.	.	.	.	.	.	.	.	.	.	.	.	.	.
4714	1	PCDHB5	NM_015669	chr5	140494983	140497888	+	.	.	.	.	.	.	.	.	.	.	.	.	.	.	.	.	.	.	.	.
6721	0,5	PCDHB17	NR_001280	chr5	140515763	140518174	+	.	.	.	.	.	.	.	.	.	.	.	.	.	.	.	.	.	.	.	.
4711	1	PCDHB15	NM_018935	chr5	140605330	140607985	+	.	.	.	.	.	.	.	.	.	.	.	.	.	.	.	.	.	.	.	.
6722	0,5	PCDHGB7	NM_032101	chr5	140777397	140780180	+	.	.	.	.	.	.	.	.	.	.	.	.	.	.	.	.	.	.	.	.
6264	0,5	DIAPH1	NM_001079812	chr5	140874771	140978806	-	.	.	.	.	.	.	.	.	.	.	.	.	.	.	.	.	.	.	.	.

Table S2

tumor associated genes								HIV				MLV				MMTV				MMTV(SIN)				MMTV(SIN)arrest			
rank	score	gene symbol	RefSEq	chrom	txStart	txEnd	strand	integrant	chr	position	strand	integrant	chr	position	strand	integrant	chr	position	strand	integrant	chr	position	strand	integrant	chr	position	strand
329	3,75	HDAC3	NM_003883	chr5	140980626	140996607	-	.	.	.	.	.	.	.	.	.	.	.	.	.	.	.	.	.	.	.	.
6806	0,5	RELL2	NM_173828	chr5	140996700	141000815	+	.	.	.	.	.	.	.	.	.	.	.	.	.	.	.	.	.	.	.	.
3014	1	ARAP3	NM_022481	chr5	141013151	141041984	-	.	.	.	.	.	.	.	.	.	.	.	.	.	.	.	.	.	.	.	.
5137	1	RNF14	NM_001201365	chr5	141326585	141350040	+	AY517208.1	chr5	141344452	-	.	.	.	.	.	.	.	.	.	.	.	.	.	.	.	.
3930	1	GNPDA1	NM_005471	chr5	141360417	141372804	-	.	.	.	.	.	.	.	.	.	.	.	.	.	.	.	.	.	.	.	.
2416	1,5	SPRY4	NM_001127496	chr5	141670175	141684804	-	.	.	.	.	.	.	.	.	.	.	.	.	.	.	.	.	.	.	.	.
831	2,5	FGF1	NM_001257212	chr5	141951926	141981104	-	.	.	.	.	.	.	.	.	.	.	.	.	.	.	.	.	.	.	.	.
1253	2	ARHGAP26	NM_001135608	chr5	142130475	142588765	+	.	.	.	.	AY516005	chr5	142235529	+	.	.	.	.	1894_215	chr5	142362827	+	.	.	.	.
221	4,25	NR3C1	NM_001204263	chr5	142637688	142763447	-	.	.	.	.	.	.	.	.	.	.	.	.	.	.	.	.	.	.	.	.
4260	1	LARS	NM_020117	chr5	145472781	145542487	-	.	.	.	.	.	.	.	.	.	.	.	.	.	.	.	.	.	.	.	.
5076	1	RBM27	NM_018989	chr5	145563355	145648977	+	.	.	.	.	.	.	.	.	.	.	.	.	.	.	.	.	.	.	.	.
293	4	PPP2R2B	NM_181675	chr5	145949260	146238520	-	.	.	.	.	.	.	.	.	.	.	.	.	.	.	.	.	.	.	.	.
5383	1	STK32A	NM_145001	chr5	146594771	146708585	+	.	.	.	.	.	.	.	.	.	.	.	.	.	.	.	.	.	.	.	.
4185	1	JAKMIP2	NM_014790	chr5	146950898	147142445	-	.	.	.	.	.	.	.	.	.	.	.	.	.	.	.	.	.	.	.	.
1169	2,25	SPINK1	NM_003122	chr5	147184335	147191453	-	.	.	.	.	.	.	.	.	.	.	.	.	.	.	.	.	.	.	.	.
5334	1	SPINK5	NM_001127699	chr5	147423727	147484696	+	.	.	.	.	.	.	.	.	.	.	.	.	.	.	.	.	.	.	.	.
5335	1	SPINK6	NM_205841	chr5	147562549	147574893	+	.	.	.	.	.	.	.	.	.	.	.	.	.	.	.	.	.	.	.	.
5336	1	SPINK7	NM_032566	chr5	147672182	147675674	+	.	.	.	.	.	.	.	.	.	.	.	.	.	.	.	.	.	.	.	.
5338	1	SPINK9	NM_001040433	chr5	147695314	147699605	+	.	.	.	.	.	.	.	.	.	.	.	.	.	.	.	.	.	.	.	.
7155	0,25	ADRB2	NM_000024	chr5	148186348	148188390	+	.	.	.	.	.	.	.	.	.	.	.	.	.	.	.	.	.	.	.	.
1468	2	IL17B	NM_014443	chr5	148734022	148739031	-	.	.	.	.	.	.	.	.	.	.	.	.	.	.	.	.	.	.	.	.
3469	1	CSNK1A1	NM_001025105	chr5	148855037	148911200	-	.	.	.	.	.	.	.	.	.	.	.	.	.	.	.	.	.	.	.	.
650	3	PPARGC1B	NM_133263	chr5	149090007	149214778	+	.	.	.	.	.	.	.	.	.	.	.	.	.	.	.	.	2223_194_1	chr5	149213355	+
153	4,75	CSF1R	NM_005211	chr5	149413046	149473128	-	.	.	.	.	.	.	.	.	.	.	.	.	.	.	.	.	.	.	.	.
51	7,25	PDGFRB	NM_002609	chr5	149473594	149515615	-	.	.	.	.	.	.	.	.	.	.	.	.	.	.	.	.	.	.	.	.
798	2,5	CAMK2A	NM_015981	chr5	149579246	149649596	-	.	.	.	.	.	.	.	.	.	.	.	.	.	.	.	.	.	.	.	.
2060	1,5	CD74	NM_004355	chr5	149761392	149772692	-	.	.	.	.	.	.	.	.	.	.	.	.	.	.	.	.	.	.	.	.
4519	1	NDST1	NM_001543	chr5	149867866	149917966	+	.	.	.	.	.	.	.	.	.	.	.	.	.	.	.	.	.	.	.	.
6801	0,5	RBM22	NM_018047	chr5	150050544	150060862	-	.	.	.	.	.	.	.	.	.	.	.	.	.	.	.	.	.	.	.	.
3960	1	GPX3	NM_002084	chr5	150380191	150388747	+	.	.	.	.	.	.	.	.	.	.	.	.	.	.	.	.	.	.	.	.
1168	2,25	SPARC	NM_003118	chr5	151020851	151046808	-	.	.	.	.	.	.	.	.	.	.	.	.	.	.	.	.	.	.	.	.
3061	1	ATOX1	NM_004045	chr5	151102575	151118403	-	.	.	.	.	.	.	.	.	.	.	.	.	.	.	.	.	.	.	.	.
7321	0,25	G3BP1	NM_005754	chr5	151131668	151165108	+	.	.	.	.	.	.	.	.	.	.	.	.	.	.	.	.	.	.	.	.
7330	0,25	GLRA1	NM_000171	chr5	151182266	151284590	-	.	.	.	.	.	.	.	.	.	.	.	.	.	.	.	.	.	.	.	.
3845	1	GALNT10	NM_198321	chr5	153550487	153780736	+	.	.	.	.	.	.	.	.	.	.	.	.	.	.	.	.	.	.	.	.
4010	1	HAND1	NM_004821	chr5	153834724	153838017	-	.	.	.	.	.	.	.	.	.	.	.	.	.	.	.	.	.	.	.	.
6548	0,5	LARP1	NM_015315	chr5	154072654	154177356	+	.	.	.	.	.	.	.	.	.	.	.	.	.	.	.	.	.	.	.	.
1086	2,25	ITK	NM_005546	chr5	156540484	156614687	+	.	.	.	.	.	.	.	.	.	.	.	.	.	.	.	.	.	.	.	.
6239	0,5	CYFIP2	NM_001037332	chr5	156625764	156755184	+	.	.	.	.	AY516815	chr5	156642301	+	.	.	.	.	.	.	.	.	.	.	.	.
775	2,5	ADAM19	NM_033274	chr5	156836889	156935361	-	.	.	.	.	.	.	.	.	.	.	.	.	.	.	.	.	.	.	.	.
5612	1	UBLCP1	NM_145049	chr5	158622666	158645626	+	.	.	.	.	.	.	.	.	.	.	.	.	.	.	.	.	.	.	.	.
4129	1	IL12B	NM_002187	chr5	158674368	158690059	-	.	.	.	.	.	.	.	.	.	.	.	.	.	.	.	.	.	.	.	.
7058	0,5	TTC1	NM_003314	chr5	159368757	159425128	+	.	.	.	.	.	.	.	.	.	.	.	.	.	.	.	.	.	.	.	.
750	2,75	PTTG1	NM_004219	chr5	159781442	159788324	+	.	.	.	.	.	.	.	.	.	.	.	.	.	.	.	.	.	.	.	.
6041	0,5	ATP10B	NM_025153	chr5	159922704	160211797	-	.	.	.	.	.	.	.	.	.	.	.	.	.	.	.	.	.	.	.	.
6374	0,5	GABRB2	NM_000813	chr5	160648013	160907708	-	.	.	.	.	.	.	.	.	.	.	.	.	.	.	.	.	.	.	.	.
3829	1	GABRA1	NM_001127646	chr5	161208475	161259543	+	.	.	.	.	.	.	.	.	.	.	.	.	.	.	.	.	.	.	.	.
2147	1,5	GABRG2	NM_000816	chr5	161427225	161515123	+	.	.	.	.	.	.	.	.	.	.	.	.	.	.	.	.	.	.	.	.
803	2,5	CCNG1	NM_199246	chr5	162797154	162804600	+	.	.	.	.	.	.	.	.	.	.	.	.	.	.	.	.	.	.	.	.
6435	0,5	HMMR	NM_001142556	chr5	162820094	162851530	+	BH609532	chr5	162834009	-	.	.	.	.	.	.	.	.	.	.	.	.	.	.	.	.
2246	1,5	MAT2B	NM_182796	chr5	162862808	162878906	+	CL529699	chr5	162867480	+	.	.	.	.	.	.	.	.	.	.	.	.	.	.	.	.

Table S2

tumor associated genes								HIV				MLV				MMTV				MMTV(SIN)				MMTV(SIN)arrest			
rank	score	gene symbol	RefSEq	chrom	txStart	txEnd	strand	integrant	chr	position	strand	integrant	chr	position	strand	integrant	chr	position	strand	integrant	chr	position	strand	integrant	chr	position	strand
5064	1	RARS	NM_002887	chr5	167846040	167878887	+	.	.	.	.	.	.	.	.	.	.	.	.	.	.	.	.	.	.	.	.
3744	1	FBLL1	NR_024356	chr5	167889159	167890217	+	.	.	.	.	.	.	.	.	.	.	.	.	.	.	.	.	.	.	.	.
4695	1	PANK3	NM_024594	chr5	167915205	167939192	-	.	.	.	.	.	.	.	.	.	.	.	.	.	.	.	.	.	.	.	.
6553	0,5	LCP2	NM_005565	chr5	169607665	169657400	-	CL528955	chr5	169641946	-	.	.	.	.	.	.	.	.	.	.	.	.	.	.	.	.
6497	0,5	KCNIP1	NM_001034837	chr5	169863625	170096214	+	.	.	.	.	.	.	.	.	.	.	.	.	.	.	.	.	.	.	.	.
664	3	RANBP17	NM_022897	chr5	170221473	170659624	+	.	.	.	.	.	.	.	.	.	.	.	.	.	.	.	.	1874_170_1	chr5	170418054	+
692	3	TLX3	NM_021025	chr5	170668892	170671743	+	.	.	.	.	.	.	.	.	.	.	.	.	.	.	.	.	.	.	.	.
405	3,5	NPM1	NM_002520	chr5	170747312	170770493	+	.	.	.	.	.	.	.	.	.	.	.	.	.	.	.	.	.	.	.	.
1398	2	FBXW11	NM_012300	chr5	171221160	171366482	-	.	.	.	.	.	.	.	.	.	.	.	.	.	.	.	.	.	.	.	.
2422	1,5	STK10	NM_005990	chr5	171401678	171547951	-	.	.	.	.	.	.	.	.	.	.	.	.	.	.	.	.	.	.	.	.
1037	2,25	DUSP1	NM_004417	chr5	172127697	172130809	-	.	.	.	.	.	.	.	.	.	.	.	.	.	.	.	.	.	.	.	.
3088	1	ATP6V0E1	NM_003945	chr5	172343368	172394506	+	.	.	.	.	.	.	.	.	.	.	.	.	.	.	.	.	.	.	.	.
6981	0,5	STC2	NM_003714	chr5	172674331	172689112	-	.	.	.	.	.	.	.	.	.	.	.	.	.	.	.	.	.	.	.	.
3155	1	BOD1	NM_138369	chr5	172966753	172976272	-	.	.	.	.	.	.	.	.	.	.	.	.	.	.	.	.	.	.	.	.
6436	0,5	HMP19	NM_015980	chr5	173405212	173468788	+	.	.	.	.	.	.	.	.	.	.	.	.	.	.	.	.	.	.	.	.
4447	1	MSX2	NM_002449	chr5	174084180	174090508	+	.	.	.	.	.	.	.	.	.	.	.	.	.	.	.	.	.	.	.	.
6288	0,5	DRD1	NM_000794	chr5	174800280	174803769	-	.	.	.	.	.	.	.	.	.	.	.	.	.	.	.	.	.	.	.	.
7361	0,25	HRH2	NM_001131055	chr5	175017645	175045851	+	.	.	.	.	.	.	.	.	.	.	.	.	.	.	.	.	.	.	.	.
3446	1	CPLX2	NM_001008220	chr5	175231106	175243629	+	.	.	.	.	.	.	.	.	.	.	.	.	.	.	.	.	.	.	.	.
3398	1	CLTB	NM_001834	chr5	175752061	175776176	-	.	.	.	.	.	.	.	.	.	.	.	.	.	.	.	.	.	.	.	.
5572	1	TSPAN17	NM_012171	chr5	176006993	176018665	+	.	.	.	.	.	.	.	.	.	.	.	.	.	.	.	.	.	.	.	.
7079	0,5	UNC5A	NM_133369	chr5	176170165	176240505	+	.	.	.	.	.	.	.	.	.	.	.	.	.	.	.	.	.	.	.	.
2186	1,5	HK3	NM_002115	chr5	176240475	176258939	-	.	.	.	.	.	.	.	.	.	.	.	.	.	.	.	.	.	.	.	.
117	5,25	FGFR4	NM_213647	chr5	176446526	176457732	+	.	.	.	.	.	.	.	.	.	.	.	.	.	.	.	.	.	.	.	.
285	4	NSD1	NM_172349	chr5	176492685	176659820	+	CL529766	chr5	176588472	-	.	.	.	.	.	.	.	.	.	.	.	.	.	.	.	.
285	4	NSD1	NM_172349	chr5	176492685	176659820	+	CL799610	chr5	176600711	+	.	.	.	.	.	.	.	.	.	.	.	.	.	.	.	.
285	4	NSD1	NM_172349	chr5	176492685	176659820	+	CL800777	chr5	176569940	+	.	.	.	.	.	.	.	.	.	.	.	.	.	.	.	.
3974	1	GRK6	NM_001004105	chr5	176786292	176800960	+	CL799812	chr5	176798856	+	.	.	.	.	.	.	.	.	.	.	.	.	.	.	.	.
2102	1,5	DBN1	NM_004395	chr5	176816219	176833300	-	CL800659	chr5	176824804	+	.	.	.	.	.	.	.	.	.	.	.	.	.	.	.	.
6282	0,5	DOK3	NM_024872	chr5	176863356	176869464	-	.	.	.	.	.	.	.	.	.	.	.	.	.	.	.	.	.	.	.	.
3548	1	DDX41	NM_016222	chr5	176871183	176876573	-	.	.	.	.	.	.	.	.	.	.	.	.	.	.	.	.	.	.	.	.
3122	1	B4GALT7	NM_007255	chr5	176959724	176969952	+	.	.	.	.	.	.	.	.	.	.	.	.	.	.	.	.	.	.	.	.
6334	0,5	FAM153A	NM_173663	chr5	177082970	177140111	-	.	.	.	.	.	.	.	.	.	.	.	.	.	.	.	.	.	.	.	.
7445	0,25	NHP2	NM_017838	chr5	177509071	177513567	-	.	.	.	.	.	.	.	.	.	.	.	.	.	.	.	.	.	.	.	.
6439	0,5	HNRNPAB	NM_004499	chr5	177564113	177570790	+	.	.	.	.	.	.	.	.	.	.	.	.	.	.	.	.	.	.	.	.
3392	1	CLK4	NM_020666	chr5	177962270	177986660	-	.	.	.	.	.	.	.	.	.	.	.	.	.	.	.	.	.	.	.	.
2903	1	ADAMTS2	NM_014244	chr5	178470457	178705037	-	.	.	.	.	.	.	.	.	.	.	.	.	.	.	.	.	.	.	.	.
889	2,5	MAML1	NM_014757	chr5	179092456	179136893	+	BH609399	chr5	179110716	-	.	.	.	.	.	.	.	.	.	.	.	.	.	.	.	.
889	2,5	MAML1	NM_014757	chr5	179092456	179136893	+	BH609536	chr5	179110716	-	.	.	.	.	.	.	.	.	.	.	.	.	.	.	.	.
4315	1	LTC4S	NM_145867	chr5	179153591	179156119	+	.	.	.	.	.	.	.	.	.	.	.	.	.	.	.	.	.	.	.	.
4394	1	MGAT4B	NM_054013	chr5	179157203	179162481	-	.	.	.	.	.	.	.	.	.	.	.	.	.	.	.	.	.	.	.	.
336	3,75	MAPK9	NM_002752	chr5	179593200	179651677	-	.	.	.	.	.	.	.	.	.	.	.	.	.	.	.	.	.	.	.	.
2156	1,5	GFPT2	NM_005110	chr5	179660305	179712921	-	.	.	.	.	.	.	.	.	.	.	.	.	.	.	.	.	.	.	.	.
2389	1,5	SCGB3A1	NM_052863	chr5	179949710	179951093	-	.	.	.	.	.	.	.	.	.	.	.	.	.	.	.	.	.	.	.	.
140	5	FLT4	NM_182925	chr5	179961111	180009230	-	.	.	.	.	.	.	.	.	.	.	.	.	.	.	.	.	.	.	.	.
2252	1,5	MGAT1	NM_002406	chr5	180150146	180162654	-	.	.	.	.	.	.	.	.	.	.	.	.	.	.	.	.	.	.	.	.
3625	1	DUSP22	NM_020185	chr6	237100	296355	+	.	.	.	.	.	.	.	.	.	.	.	.	.	.	.	.	.	.	.	.
1478	2	IRF4	NM_001195286	chr6	336738	356443	+	.	.	.	.	.	.	.	.	.	.	.	.	.	.	.	.	.	.	.	.
4102	1	HUS1B	NM_148959	chr6	600938	601964	-	.	.	.	.	.	.	.	.	.	.	.	.	.	.	.	.	.	.	.	.
2590	1,25	FOXQ1	NM_033260	chr6	1257674	1259993	+	.	.	.	.	.	.	.	.	.	.	.	.	.	.	.	.	.	.	.	.
1410	2	FOXC1	NM_001453	chr6	1555679	1559128	+	.	.	.	.	.	.	.	.	.	.	.	.	.	.	.	.	.	.	.	.

Table S2

tumor associated genes								HIV				MLV				MMTV				MMTV(SIN)				MMTV(SIN)arrest			
rank	score	gene symbol	RefSEq	chrom	txStart	txEnd	strand	integrant	chr	position	strand	integrant	chr	position	strand	integrant	chr	position	strand	integrant	chr	position	strand	integrant	chr	position	strand
3909	1	GMDS	NM_001500	chr6	1569033	2190867	-	.	.	.	.	.	.	.	.	.	.	.	.	.	.	.	.	3051_283_3	chr6	1763022	+
1551	2	MYLK4	NM_001012418	chr6	2608861	2696153	-	.	.	.	.	.	.	.	.	.	.	.	.	.	.	.	.	.	.	.	.
5125	1	RIPK1	NM_003804	chr6	3022056	3060420	+	.	.	.	.	.	.	.	.	.	.	.	.	.	.	.	.	.	.	.	.
7607	0,25	TUBB2A	NM_001069	chr6	3098900	3102782	-	.	.	.	.	.	.	.	.	.	.	.	.	.	.	.	.	.	.	.	.
1145	2,25	PRPF4B	NM_003913	chr6	3966567	4010216	+	CL529526	chr6	3996297	+	.	.	.	.	.	.	.	.	.	.	.	.	.	.	.	.
3736	1	FARS2	NM_006567	chr6	5206582	5716815	+	BH609554	chr6	5455880	+	.	.	.	.	.	.	.	.	.	.	.	.	.	.	.	.
3707	1	F13A1	NM_000129	chr6	6089309	6265923	-	.	.	.	.	.	.	.	.	.	.	.	.	.	.	.	.	.	.	.	.
6583	0,5	LY86	NM_004271	chr6	6533932	6600215	+	.	.	.	.	.	.	.	.	.	.	.	.	.	.	.	.	.	.	.	.
6832	0,5	RREB1	NM_001003698	chr6	7053084	7197212	+	BH609566	chr6	7155842	-	.	.	.	.	.	.	.	.	.	.	.	.	.	.	.	.
5122	1	RIOK1	NM_153005	chr6	7347878	7363269	+	.	.	.	.	.	.	.	.	.	.	.	.	65_2_2173_8	chr6	7348548	-	.	.	.	.
3610	1	DSP	NM_004415	chr6	7486868	7531945	+	.	.	.	.	.	.	.	.	.	.	.	.	.	.	.	.	.	.	.	.
5883	0,75	BMP6	NM_001718	chr6	7672009	7826960	+	.	.	.	.	.	.	.	.	.	.	.	.	.	.	.	.	.	.	.	.
964	2,5	TFAP2A	NM_001032280	chr6	10504901	10520593	-	.	.	.	.	.	.	.	.	.	.	.	.	.	.	.	.	.	.	.	.
3869	1	GCNT2	NM_145655	chr6	10693978	10737587	+	.	.	.	.	.	.	.	.	.	.	.	.	.	.	.	.	.	.	.	.
4333	1	MAK	NM_001242957	chr6	10870941	10946774	-	.	.	.	.	.	.	.	.	.	.	.	.	.	.	.	.	.	.	.	.
250	4	ELOVL2	NM_017770	chr6	11088978	11152610	-	.	.	.	.	.	.	.	.	.	.	.	.	.	.	.	.	.	.	.	.
4555	1	NEDD9	NM_182966	chr6	11308591	11340901	-	.	.	.	.	.	.	.	.	.	.	.	.	.	.	.	.	.	.	.	.
461	3,25	EDN1	NM_001955	chr6	12398514	12405413	+	.	.	.	.	.	.	.	.	.	.	.	.	.	.	.	.	.	.	.	.
2319	1,5	PHACTR1	NM_001242648	chr6	12825022	13396054	+	.	.	.	.	.	.	.	.	.	.	.	.	.	.	.	.	.	.	.	.
3877	1	GFOD1	NM_001242629	chr6	13577480	13595848	-	.	.	.	.	.	.	.	.	.	.	.	.	.	.	.	.	.	.	.	.
957	2,5	SIRT5	NM_012241	chr6	13682837	13723369	+	.	.	.	.	.	.	.	.	.	.	.	.	.	.	.	.	.	.	.	.
5059	1	RANBP9	NM_005493	chr6	13729708	13819775	-	.	.	.	.	.	.	.	.	.	.	.	.	.	.	.	.	.	.	.	.
7240	0,25	CD83	NM_004233	chr6	14225843	14245127	+	.	.	.	.	.	.	.	.	.	.	.	.	.	.	.	.	.	.	.	.
4186	1	JARID2	NM_001267040	chr6	15357064	15630252	+	CL800001	chr6	15466253	-	.	.	.	.	.	.	.	.	.	.	.	.	.	.	.	.
4186	1	JARID2	NM_001267040	chr6	15357064	15630252	+	AY516995.1	chr6	15364234	-	.	.	.	.	.	.	.	.	.	.	.	.	.	.	.	.
4473	1	MYLIP	NM_013262	chr6	16237295	16256457	+	.	.	.	.	.	.	.	.	.	.	.	.	.	.	.	.	.	.	.	.
3912	1	GMPR	NM_006877	chr6	16346789	16403759	+	.	.	.	.	.	.	.	.	.	.	.	.	.	.	.	.	.	.	.	.
6128	0,5	CAP2	NM_006366	chr6	17501714	17666002	+	CL528965	chr6	17593554	+	.	.	.	.	.	.	.	.	.	.	.	.	.	.	.	.
909	2,5	NUP153	NM_005124	chr6	17723247	17814797	-	BH609549	chr6	17738608	-	AY516321	chr6	17793094	-	.	.	.	.	.	.	.	.	.	.	.	.
1351	2	DEK	NM_003472	chr6	18332378	18372778	-	.	.	.	.	.	.	.	.	.	.	.	.	.	.	.	.	.	.	.	.
5138	1	RNF144B	NM_182757	chr6	18495559	18577084	+	.	.	.	.	.	.	.	.	.	.	.	.	.	.	.	.	.	.	.	.
4107	1	ID4	NM_001546	chr6	19945579	19950410	+	.	.	.	.	.	.	.	.	.	.	.	.	.	.	.	.	.	.	.	.
817	2,5	E2F3	NM_001243076	chr6	20512012	20601924	+	.	.	.	.	.	.	.	.	.	.	.	.	.	.	.	.	.	.	.	.
2412	1,5	SOX4	NM_003107	chr6	21701950	21706828	+	.	.	.	.	.	.	.	.	.	.	.	.	.	.	.	.	.	.	.	.
4949	1	PRL	NM_001163558	chr6	22395451	22411061	-	.	.	.	.	.	.	.	.	.	.	.	.	.	.	.	.	.	.	.	.
6686	0,5	NRSN1	NM_080723	chr6	24234392	24255736	+	.	.	.	.	.	.	.	.	.	.	.	.	.	.	.	.	.	.	.	.
1429	2	GPLD1	NM_001503	chr6	24534040	24597829	-	.	.	.	.	.	.	.	.	.	.	.	.	.	.	.	.	.	.	.	.
2952	1	ALDH5A1	NM_001080	chr6	24603175	24645414	+	.	.	.	.	.	.	.	.	.	.	.	.	.	.	.	.	.	.	.	.
6517	0,5	KIAA0319	NM_014809	chr6	24652310	24754362	-	.	.	.	.	.	.	.	.	.	.	.	.	.	.	.	.	.	.	.	.
6341	0,5	FAM65B	NM_014722	chr6	24912491	25019174	-	.	.	.	.	AY516862	chr6	25011458	+	.	.	.	.	.	.	.	.	.	.	.	.
6341	0,5	FAM65B	NM_014722	chr6	24912491	25019174	-	.	.	.	.	AY516512	chr6	25010145	+	.	.	.	.	.	.	.	.	.	.	.	.
4304	1	LRRC16A	NM_001173977	chr6	25387634	25728737	+	CL800334	chr6	25536671	+	.	.	.	.	.	.	.	.	.	.	.	.	.	.	.	.
7531	0,25	SCGN	NM_006998	chr6	25760407	25809987	+	.	.	.	.	.	.	.	.	.	.	.	.	.	.	.	.	.	.	.	.
7049	0,5	TRIM38	NM_006355	chr6	26070895	26095536	+	.	.	.	.	.	.	.	.	.	.	.	.	.	.	.	.	.	.	.	.
4048	1	HIST1H1A	NM_005325	chr6	26125238	26126019	-	.	.	.	.	.	.	.	.	.	.	.	.	.	.	.	.	.	.	.	.
6427	0,5	HFE	NM_139006	chr6	26195487	26203448	+	.	.	.	.	.	.	.	.	.	.	.	.	.	.	.	.	.	.	.	.
6430	0,5	HIST1H4F	NM_003540	chr6	26348632	26349000	+	.	.	.	.	.	.	.	.	.	.	.	.	.	.	.	.	.	.	.	.
6431	0,5	HIST1H4H	NM_003543	chr6	26393332	26393706	-	.	.	.	.	.	.	.	.	.	.	.	.	.	.	.	.	.	.	.	.
4049	1	HIST1H2BJ	NM_021058	chr6	27208073	27208554	-	.	.	.	.	.	.	.	.	.	.	.	.	.	.	.	.	.	.	.	.
1449	2	HIST1H4I	NM_003495	chr6	27215066	27215436	+	.	.	.	.	.	.	.	.	.	.	.	.	.	.	.	.	.	.	.	.
5793	1	ZNF391	NM_001076781	chr6	27464502	27477206	+	.	.	.	.	.	.	.	.	.	.	.	.	.	.	.	.	.	.	.	.

Table S2

tumor associated genes								HIV				MLV				MMTV				MMTV(SIN)				MMTV(SIN)arrest			
rank	score	gene symbol	RefSEq	chrom	txStart	txEnd	strand	integrant	chr	position	strand	integrant	chr	position	strand	integrant	chr	position	strand	integrant	chr	position	strand	integrant	chr	position	strand
7127	0,5	ZNF184	NM_007149	chr6	27526499	27548876	-	.	.	.	.	.	.	.	.	.	.	.	.	.	.	.	.	.	.	.	.
2185	1,5	HIST1H1B	NM_005322	chr6	27942548	27943338	-	.	.	.	.	.	.	.	.	.	.	.	.	.	.	.	.	.	.	.	.
6432	0,5	HIST1H4L	NM_003546	chr6	27948904	27949268	-	.	.	.	.	.	.	.	.	.	.	.	.	.	.	.	.	.	.	.	.
7624	0,25	ZNF165	NM_003447	chr6	28156460	28165319	+	.	.	.	.	.	.	.	.	.	.	.	.	.	.	.	.	.	.	.	.
7131	0,5	ZNF323	NM_001135216	chr6	28400493	28411890	-	.	.	.	.	.	.	.	.	.	.	.	.	.	.	.	.	.	.	.	.
3962	1	GPX6	NM_182701	chr6	28579051	28591549	-	.	.	.	.	.	.	.	.	.	.	.	.	.	.	.	.	.	.	.	.
3961	1	GPX5	NM_001509	chr6	28601767	28610707	+	.	.	.	.	.	.	.	.	.	.	.	.	.	.	.	.	.	.	.	.
5552	1	TRIM27	NM_006510	chr6	28978757	28999747	-	.	.	.	.	.	.	.	.	.	.	.	.	.	.	.	.	.	.	.	.
7074	0,5	UBD	NM_006398	chr6	29631367	29635681	-	.	.	.	.	.	.	.	.	.	.	.	.	.	.	.	.	.	.	.	.
2593	1,25	GABBR1	NM_001470	chr6	29677983	29708941	-	.	.	.	.	.	.	.	.	.	.	.	.	.	.	.	.	.	.	.	.
2681	1,25	MOG	NM_206814	chr6	29732736	29748128	+	.	.	.	.	.	.	.	.	.	.	.	.	.	.	.	.	.	.	.	.
4054	1	HLA-F	NM_001098479	chr6	29799095	29803052	+	.	.	.	.	.	.	.	.	.	.	.	.	.	.	.	.	.	.	.	.
856	2,5	HLA-G	NM_002127	chr6	29902734	29906878	+	.	.	.	.	.	.	.	.	.	.	.	.	.	.	.	.	.	.	.	.
5866	1	ZNRD1	NM_170783	chr6	30137014	30140665	+	.	.	.	.	.	.	.	.	.	.	.	.	.	.	.	.	.	.	.	.
4907	1	PPP1R11	NM_021959	chr6	30142910	30146087	+	.	.	.	.	.	.	.	.	.	.	.	.	.	.	.	.	.	.	.	.
4053	1	HLA-E	NM_005516	chr6	30565161	30569961	+	.	.	.	.	.	.	.	.	.	.	.	.	.	.	.	.	.	.	.	.
6389	0,5	GNL1	NM_005275	chr6	30617133	30633350	-	.	.	.	.	.	.	.	.	.	.	.	.	.	.	.	.	.	.	.	.
4958	1	PRR3	NM_025263	chr6	30632464	30640452	+	.	.	.	.	.	.	.	.	.	.	.	.	.	.	.	.	.	.	.	.
4906	1	PPP1R10	NR_072994	chr6	30676155	30693063	-	.	.	.	.	.	.	.	.	.	.	.	.	.	.	.	.	.	.	.	.
6104	0,5	C6orf136	NM_001109938	chr6	30722794	30728966	+	.	.	.	.	.	.	.	.	.	.	.	.	.	.	.	.	.	.	.	.
397	3,5	MDC1	NM_014641	chr6	30775562	30793437	-	.	.	.	.	.	.	.	.	.	.	.	.	.	.	.	.	.	.	.	.
7068	0,5	TUBB	NM_178014	chr6	30796135	30801174	+	.	.	.	.	.	.	.	.	.	.	.	.	.	.	.	.	.	.	.	.
5921	0,75	IER3	NM_003897	chr6	30818954	30820306	-	.	.	.	.	.	.	.	.	.	.	.	.	.	.	.	.	.	.	.	.
558	3	DDR1	NM_001202521	chr6	30964443	30975912	+	.	.	.	.	.	.	.	.	.	.	.	.	.	.	.	.	.	.	.	.
1436	2	GTF2H4	NM_001517	chr6	30983955	30989859	+	.	.	.	.	.	.	.	.	.	.	.	.	.	.	.	.	.	.	.	.
5669	1	VARS2	NM_001167733	chr6	30989960	31002214	+	.	.	.	.	.	.	.	.	.	.	.	.	.	.	.	.	.	.	.	.
3235	1	C6orf15	NM_014070	chr6	31186978	31188311	-	.	.	.	.	.	.	.	.	.	.	.	.	.	.	.	.	.	.	.	.
7494	0,25	PSORS1C2	NM_014069	chr6	31213289	31215106	-	.	.	.	.	.	.	.	.	.	.	.	.	.	.	.	.	.	.	.	.
1662	2	POU5F1	NM_203289	chr6	31240092	31242594	-	CL799968	chr6	31240607	+	.	.	.	.	.	.	.	.	.	.	.	.	.	.	.	.
4051	1	HLA-B	NM_005514	chr6	31429627	31432968	-	.	.	.	.	.	.	.	.	.	.	.	.	.	.	.	.	.	.	.	.
1270	2	ATP6V1G2	NM_130463	chr6	31620206	31622604	-	.	.	.	.	.	.	.	.	.	.	.	.	.	.	.	.	.	.	.	.
7406	0,25	LTA	NM_000595	chr6	31648049	31650079	+	.	.	.	.	.	.	.	.	.	.	.	.	.	.	.	.	.	.	.	.
970	2,5	TNF	NM_000594	chr6	31651322	31654091	+	.	.	.	.	.	.	.	.	.	.	.	.	.	.	.	.	.	.	.	.
7157	0,25	AIF1	NM_032955	chr6	31691767	31692777	+	.	.	.	.	.	.	.	.	.	.	.	.	.	.	.	.	.	.	.	.
3472	1	CSNK2B	NM_001320	chr6	31741635	31745822	+	.	.	.	.	.	.	.	.	.	.	.	.	.	.	.	.	.	.	.	.
5892	0,75	CLIC1	NM_001288	chr6	31806336	31812320	-	.	.	.	.	.	.	.	.	.	.	.	.	.	.	.	.	.	.	.	.
4444	1	MSH5	NM_002441	chr6	31815703	31838434	+	.	.	.	.	.	.	.	.	.	.	.	.	.	.	.	.	.	.	.	.
5668	1	VARS	NM_006295	chr6	31853275	31871691	-	.	.	.	.	.	.	.	.	.	.	.	.	.	.	.	.	.	.	.	.
1458	2	HSPA1A	NM_005345	chr6	31891269	31893698	+	.	.	.	.	.	.	.	.	.	.	.	.	.	.	.	.	.	.	.	.
1459	2	HSPA1B	NM_005346	chr6	31903490	31906010	+	.	.	.	.	.	.	.	.	.	.	.	.	.	.	.	.	.	.	.	.
4567	1	NEU1	NM_000434	chr6	31934807	31938688	-	.	.	.	.	.	.	.	.	.	.	.	.	.	.	.	.	.	.	.	.
820	2,5	EHMT2	NM_006709	chr6	31955515	31973443	-	CL800816	[illegible]_qbl_h	3105917	-	.	.	.	.	.	.	.	.	.	.	.	.	.	.	.	.
6169	0,5	CFB	NM_001710	chr6	32021699	32027840	+	.	.	.	.	.	.	.	.	.	.	.	.	.	.	.	.	.	.	.	.
5379	1	STK19	NM_032454	chr6	32089495	32089939	+	.	.	.	.	.	.	.	.	.	.	.	.	.	.	.	.	.	.	.	.
554	3	CYP21A2	NM_001128590	chr6	32114071	32117426	+	.	.	.	.	.	.	.	.	.	.	.	.	.	.	.	.	.	.	.	.
6773	0,5	PRRT1	NM_030651	chr6	32224117	32227698	-	.	.	.	.	.	.	.	.	.	.	.	.	.	.	.	.	.	.	.	.
4930	1	PPT2	NM_138717	chr6	32229206	32239436	+	.	.	.	.	.	.	.	.	.	.	.	.	.	.	.	.	.	.	.	.
2925	1	AGPAT1	NM_032741	chr6	32243960	32253866	-	.	.	.	.	.	.	.	.	.	.	.	.	.	.	.	.	.	.	.	.
740	2,75	NOTCH4	NM_004557	chr6	32270597	32299822	-	.	.	.	.	.	.	.	.	.	.	.	.	.	.	.	.	.	.	.	.
1068	2,25	HLA-DRA	NM_019111	chr6	32515596	32520802	+	.	.	.	.	.	.	.	.	.	.	.	.	.	.	.	.	.	.	.	.
1067	2,25	HLA-DQA1	NM_002122	chr6	32713160	32719407	+	.	.	.	.	.	.	.	.	.	.	.	.	.	.	.	.	.	.	.	.

Table S2

tumor associated genes								HIV				MLV				MMTV				MMTV(SIN)				MMTV(SIN)arrest			
rank	score	gene symbol	RefSEq	chrom	txStart	txEnd	strand	integrant	chr	position	strand	integrant	chr	position	strand	integrant	chr	position	strand	integrant	chr	position	strand	integrant	chr	position	strand
1171	2,25	TAP2	NM_018833	chr6	32897587	32914525	-	.	.	.	.	.	.	.	.	.	.	.	.	.	.	.	.	.	.	.	.
5431	1	TAP1	NM_000593	chr6	32920963	32929726	-	.	.	.	.	.	.	.	.	.	.	.	.	.	.	.	.	.	.	.	.
7493	0,25	PSMB9	NM_002800	chr6	32929915	32935606	+	.	.	.	.	.	.	.	.	.	.	.	.	.	.	.	.	.	.	.	.
3160	1	BRD2	NM_005104	chr6	33044414	33057260	+	.	.	.	.	.	.	.	.	.	.	.	.	.	.	.	.	.	.	.	.
7348	0,25	HLA-DOA	NM_002119	chr6	33079937	33085367	-	.	.	.	.	.	.	.	.	.	.	.	.	.	.	.	.	.	.	.	.
4052	1	HLA-DPA1	NM_001242524	chr6	33140323	33156533	-	.	.	.	.	.	.	.	.	.	.	.	.	.	.	.	.	.	.	.	.
7349	0,25	HLA-DPB1	NM_002121	chr6	33151680	33165451	+	.	.	.	.	.	.	.	.	.	.	.	.	.	.	.	.	.	.	.	.
5172	1	RXRB	NM_021976	chr6	33269339	33276451	-	.	.	.	.	.	.	.	.	.	.	.	.	.	.	.	.	.	.	.	.
4092	1	HSD17B8	NM_014234	chr6	33280391	33282586	+	.	.	.	.	.	.	.	.	.	.	.	.	.	.	.	.	.	.	.	.
3107	1	B3GALT4	NM_003782	chr6	33352894	33354580	+	.	.	.	.	.	.	.	.	.	.	.	.	.	.	.	.	.	.	.	.
5112	1	RGL2	NM_004761	chr6	33367408	33375143	-	.	.	.	.	.	.	.	.	.	.	.	.	.	.	.	.	.	.	.	.
1343	2	DAXX	NM_001141970	chr6	33394312	33398771	-	.	.	.	.	.	.	.	.	.	.	.	.	.	.	.	.	.	.	.	.
6994	0,5	SYNGAP1	NM_006772	chr6	33495824	33529444	+	.	.	.	.	.	.	.	.	.	.	.	.	.	.	.	.	.	.	.	.
4183	1	ITPR3	NM_002224	chr6	33697133	33772326	+	CL529723	chr6	33701457	+	AY516424	chr6	33750589	+	.	.	.	.	.	.	.	.	.	.	.	.
6414	0,5	GRM4	NM_000841	chr6	34097600	34221892	-	.	.	.	.	.	.	.	.	.	.	.	.	.	.	.	.	.	.	.	.
264	4	HMGA1	NM_145901	chr6	34312627	34321986	+	.	.	.	.	.	.	.	.	.	.	.	.	.	.	.	.	.	.	.	.
2304	1,5	PACSIN1	NM_001199583	chr6	34590626	34612017	+	.	.	.	.	.	.	.	.	.	.	.	.	.	.	.	.	.	.	.	.
2413	1,5	SPDEF	NM_012391	chr6	34613556	34632088	-	.	.	.	.	.	.	.	.	.	.	.	.	.	.	.	.	.	.	.	.
2719	1,25	PPARD	NM_177435	chr6	35418312	35501151	+	.	.	.	.	.	.	.	.	.	.	.	.	.	.	.	.	.	.	.	.
138	5	FANCE	NM_021922	chr6	35528115	35542859	+	.	.	.	.	.	.	.	.	.	.	.	.	.	.	.	.	.	.	.	.
7013	0,5	TEAD3	NM_003214	chr6	35549351	35572839	-	.	.	.	.	.	.	.	.	.	.	.	.	.	.	.	.	.	.	.	.
6355	0,5	FKBP5	NM_001145776	chr6	35649339	35764670	-	BH609544	chr6	35711064	-	.	.	.	.	.	.	.	.	.	.	.	.	.	.	.	.
5352	1	SRPK1	NM_003137	chr6	35908788	35996935	-	.	.	.	.	.	.	.	.	.	.	.	.	.	.	.	.	.	.	.	.
6913	0,5	SLC26A8	NM_052961	chr6	36019268	36100391	-	.	.	.	.	.	.	.	.	.	.	.	.	.	.	.	.	.	.	.	.
616	3	MAPK14	NM_139012	chr6	36103431	36186991	+	CL800194	chr6	36131418	-	.	.	.	.	.	.	.	.	.	.	.	.	.	.	.	.
1521	2	MAPK13	NR_072996	chr6	36206238	36220279	+	.	.	.	.	.	.	.	.	.	.	.	.	.	.	.	.	.	.	.	.
5386	1	STK38	NM_007271	chr6	36569646	36623225	-	CL800599	chr6	36577013	+	.	.	.	.	.	.	.	.	.	.	.	.	.	.	.	.
40	7,75	CDKN1A	NM_000389	chr6	36754433	36763095	+	.	.	.	.	.	.	.	.	.	.	.	.	.	.	.	.	.	.	.	.
413	3,5	PIM1	NM_002648	chr6	37245899	37251182	+	.	.	.	.	.	.	.	.	.	.	.	.	.	.	.	.	.	.	.	.
1056	2,25	GLO1	NM_006708	chr6	38751679	38778930	-	.	.	.	.	.	.	.	.	.	.	.	.	.	.	.	.	.	.	.	.
1362	2	DNAH8	NM_001206927	chr6	38791094	39106552	+	.	.	.	.	.	.	.	.	.	.	.	.	.	.	.	.	.	.	.	.
6534	0,5	KIF6	NM_145027	chr6	39410853	39801159	-	.	.	.	.	.	.	.	.	.	.	.	.	.	.	.	.	.	.	.	.
6249	0,5	DAAM2	NM_001201427	chr6	39868136	39980631	+	.	.	.	.	.	.	.	.	.	.	.	.	.	.	.	.	.	.	.	.
4302	1	LRFN2	NM_020737	chr6	40467350	40663104	-	.	.	.	.	.	.	.	.	.	.	.	.	.	.	.	.	.	.	.	.
3005	1	APOBEC2	NM_006789	chr6	41128917	41140608	+	.	.	.	.	.	.	.	.	.	.	.	.	.	.	.	.	.	.	.	.
5970	0,75	TREM1	NM_001242590	chr6	41350976	41362435	-	.	.	.	.	.	.	.	.	.	.	.	.	.	.	.	.	.	.	.	.
689	3	TFEB	NM_001167827	chr6	41759693	41811975	-	.	.	.	.	.	.	.	.	.	.	.	.	.	.	.	.	.	.	.	.
4769	1	PGC	NM_001166424	chr6	41816553	41823117	-	.	.	.	.	.	.	.	.	.	.	.	.	.	.	.	.	.	.	.	.
202	4,25	CCND3	NM_001136126	chr6	42010648	42124588	-	CL529311	chr6	42035511	+	.	.	.	.	.	.	.	.	.	.	.	.	.	.	.	.
6771	0,5	PRPH2	NM_000322	chr6	42772310	42798336	-	.	.	.	.	.	.	.	.	.	.	.	.	.	.	.	.	.	.	.	.
4980	1	PTCRA	NM_001243170	chr6	42991704	43001553	+	.	.	.	.	.	.	.	.	.	.	.	.	.	.	.	.	.	.	.	.
2604	1,25	GNMT	NM_018960	chr6	43036477	43039596	+	.	.	.	.	.	.	.	.	.	.	.	.	.	.	.	.	.	.	.	.
2341	1,5	PPP2R5D	NM_180976	chr6	43060214	43088061	+	.	.	.	.	.	.	.	.	.	.	.	.	.	.	.	.	.	.	.	.
1149	2,25	PTK7	NM_152880	chr6	43151983	43237436	+	.	.	.	.	.	.	.	.	.	.	.	.	.	.	.	.	.	.	.	.
1967	1,75	SRF	NM_003131	chr6	43246897	43257222	+	.	.	.	.	.	.	.	.	.	.	.	.	.	.	.	.	.	.	.	.
3489	1	CUL9	NM_015089	chr6	43257899	43300303	+	AY517429.1	chr6	43287643	-	.	.	.	.	.	.	.	.	.	.	.	.	.	.	.	.
1826	2	TTBK1	NM_032538	chr6	43319199	43363975	+	.	.	.	.	.	.	.	.	.	.	.	.	.	.	.	.	.	.	.	.
2854	1	ABCC10	NM_001198934	chr6	43503269	43526141	+	.	.	.	.	.	.	.	.	.	.	.	.	.	.	.	.	.	.	.	.
3589	1	DLK2	NM_023932	chr6	43526067	43531264	-	.	.	.	.	.	.	.	.	.	.	.	.	.	.	.	.	.	.	.	.
1650	2	POLR1C	NM_203290	chr6	43592754	43597224	+	.	.	.	.	.	.	.	.	.	.	.	.	.	.	.	.	.	.	.	.
5716	1	XPO5	NM_020750	chr6	43598045	43651790	-	CL799822	chr6	43646513	+	.	.	.	.	.	.	.	.	.	.	.	.	.	.	.	.

Table S2

tumor associated genes								HIV				MLV				MMTV				MMTV(SIN)				MMTV(SIN)arrest			
rank	score	gene symbol	RefSEq	chrom	txStart	txEnd	strand	integrant	chr	position	strand	integrant	chr	position	strand	integrant	chr	position	strand	integrant	chr	position	strand	integrant	chr	position	strand
4864	1	POLH	NM_006502	chr6	43651855	43696238	+	CL799950	chr6	43665047	+	AY516820	chr6	43667589	-	.	.	.	.	.	.	.	.	.	.	.	.
4864	1	POLH	NM_006502	chr6	43651855	43696238	+	CL800407	chr6	43662075	+	AY516820	chr6	43667589	-	.	.	.	.	.	.	.	.	.	.	.	.
3992	1	GTPBP2	NM_019096	chr6	43696195	43704914	-	.	.	.	.	.	.	.	.	.	.	.	.	.	.	.	.	.	.	.	.
149	5	VEGFA	NM_001025368	chr6	43845923	43862201	+	.	.	.	.	.	.	.	.	.	.	.	.	.	.	.	.	.	.	.	.
5267	1	SLC29A1	NM_001078174	chr6	44295219	44309866	+	.	.	.	.	.	.	.	.	.	.	.	.	.	.	.	.	.	.	.	.
382	3,5	HSP90AB1	NM_007355	chr6	44322826	44329592	+	.	.	.	.	.	.	.	.	.	.	.	.	.	.	.	.	.	.	.	.
4575	1	NFKBIE	NM_004556	chr6	44333880	44341503	-	.	.	.	.	.	.	.	.	.	.	.	.	.	.	.	.	.	.	.	.
2846	1	AARS2	NM_020745	chr6	44374440	44389041	-	.	.	.	.	.	.	.	.	.	.	.	.	.	.	.	.	.	.	.	.
1302	2	CDC5L	NM_001253	chr6	44463228	44526139	+	.	.	.	.	.	.	.	.	.	.	.	.	.	.	.	.	.	.	.	.
674	3	RUNX2	NM_004348	chr6	45497891	45626797	+	CL800133	chr6	45414392	-	AY515890	chr6	45523382	+	.	.	.	.	.	.	.	.	.	.	.	.
1383	2	ENPP4	NM_014936	chr6	46205659	46222395	+	.	.	.	.	.	.	.	.	.	.	.	.	.	.	.	.	.	.	.	.
3683	1	ENPP5	NM_021572	chr6	46235720	46246676	-	.	.	.	.	.	.	.	.	.	.	.	.	.	.	.	.	.	.	.	.
5450	1	TDRD6	NM_001168359	chr6	46763570	46780015	+	.	.	.	.	.	.	.	.	.	.	.	.	.	.	.	.	.	.	.	.
4834	1	PLA2G7	NM_005084	chr6	46780011	46811110	-	.	.	.	.	.	.	.	.	.	.	.	.	.	.	.	.	.	.	.	.
6149	0,5	CD2AP	NM_012120	chr6	47553483	47702955	+	CL800357	chr6	47589322	+	AY515908	chr6	47567190	+	.	.	.	.	.	.	.	.	.	.	.	.
6149	0,5	CD2AP	NM_012120	chr6	47553483	47702955	+	BH609537	chr6	47569630	+	AY515908	chr6	47567190	+	.	.	.	.	.	.	.	.	.	.	.	.
6149	0,5	CD2AP	NM_012120	chr6	47553483	47702955	+	CL799705	chr6	47679872	+	AY515908	chr6	47567190	+	.	.	.	.	.	.	.	.	.	.	.	.
4463	1	MUT	NM_000255	chr6	49506031	49539000	-	.	.	.	.	.	.	.	.	.	.	.	.	.	.	.	.	.	.	.	.
3338	1	CENPQ	NM_018132	chr6	49539054	49568779	+	.	.	.	.	.	.	.	.	.	.	.	.	.	.	.	.	.	.	.	.
1610	2	PGK2	NM_138733	chr6	49861322	49863012	-	.	.	.	.	.	.	.	.	.	.	.	.	.	.	.	.	.	.	.	.
4817	1	PKHD1	NM_170724	chr6	51693605	52060382	-	.	.	.	.	.	.	.	.	.	.	.	.	.	.	.	.	2138_186_3	chr6	51857638	+
4371	1	MCM3	NM_001270472	chr6	52236770	52257638	-	CL800446	chr6	52252484	+	.	.	.	.	.	.	.	.	.	.	.	.	.	.	.	.
6716	0,5	PAQR8	NM_133367	chr6	52334884	52380534	+	CL529548	chr6	52357285	-	.	.	.	.	.	.	.	.	.	.	.	.	.	.	.	.
7045	0,5	TRAM2	NM_012288	chr6	52470158	52549821	-	.	.	.	.	AY516333	chr6	52487298	+	.	.	.	.	.	.	.	.	.	.	.	.
3979	1	GSTA2	NM_000846	chr6	52722843	52736320	-	.	.	.	.	.	.	.	.	.	.	.	.	.	.	.	.	.	.	.	.
1063	2,25	GSTA1	NM_145740	chr6	52764136	52776623	-	.	.	.	.	.	.	.	.	.	.	.	.	.	.	.	.	.	.	.	.
3981	1	GSTA5	NM_153699	chr6	52804499	52818852	-	.	.	.	.	.	.	.	.	.	.	.	.	.	.	.	.	.	.	.	.
3980	1	GSTA3	NM_000847	chr6	52869397	52882455	-	.	.	.	.	.	.	.	.	.	.	.	.	.	.	.	.	.	.	.	.
2176	1,5	GSTA4	NM_001512	chr6	52950704	52968137	-	.	.	.	.	AY516865	chr6	52966946	-	.	.	.	.	.	.	.	.	.	.	.	.
2198	1,5	ICK	NM_016513	chr6	52974056	53034559	-	.	.	.	.	.	.	.	.	.	.	.	.	.	.	.	.	.	.	.	.
3761	1	FBXO9	NM_033480	chr6	53038215	53073629	+	.	.	.	.	.	.	.	.	.	.	.	.	.	.	.	.	.	.	.	.
3673	1	ELOVL5	NM_001242831	chr6	53266846	53321936	-	CL529612	chr6	53302972	-	.	.	.	.	.	.	.	.	.	.	.	.	.	.	.	.
843	2,5	GCLC	NM_001498	chr6	53470098	53517886	-	.	.	.	.	.	.	.	.	.	.	.	.	.	.	.	.	.	.	.	.
6434	0,5	HMGCLL1	NM_001042406	chr6	55407129	55551971	-	.	.	.	.	.	.	.	.	.	.	.	.	.	.	.	.	.	.	.	.
7192	0,25	BMP5	NM_021073	chr6	55728195	55848334	-	.	.	.	.	.	.	.	.	.	.	.	.	.	.	.	.	.	.	.	.
460	3,25	DST	NM_015548	chr6	56430743	56615653	-	.	.	.	.	.	.	.	.	.	.	.	.	.	.	.	.	.	.	.	.
4939	1	PRIM2	NM_000947	chr6	57290380	57621335	+	CL800448	chr6	57572618	-	.	.	.	.	.	.	.	.	.	.	.	.	.	.	.	.
2217	1,5	KHDRBS2	NM_152688	chr6	62447823	63054059	-	CL800189	chr6	62796629	-	.	.	.	.	.	.	.	.	.	.	.	.	.	.	.	.
2730	1,25	PTP4A1	NM_003463	chr6	64339878	64351448	+	.	.	.	.	.	.	.	.	.	.	.	.	.	.	.	.	.	.	.	.
127	5	BAI3	NM_001704	chr6	69402352	70156124	+	.	.	.	.	.	.	.	.	.	.	.	.	330_391	chr6	69766805	+	.	.	.	.
3414	1	COL19A1	NM_001858	chr6	70633168	70978878	+	.	.	.	.	.	.	.	.	.	.	.	.	.	.	.	.	.	.	.	.
3111	1	B3GAT2	NM_080742	chr6	71627789	71723509	-	.	.	.	.	.	.	.	.	.	.	.	.	.	.	.	.	.	.	.	.
6819	0,5	RIMS1	NM_001168408	chr6	72979234	73169566	+	CL528974	chr6	72979670	-	AY516199	chr6	73042239	+	.	.	.	.	.	.	.	.	.	.	.	.
4203	1	KCNQ5	NM_001160133	chr6	73388291	73965294	+	CL529580	chr6	73428678	-	.	.	.	.	.	.	.	.	24_1_1342_4	chr6	73714906	+	.	.	.	.
2566	1,25	DDX43	NM_018665	chr6	74161005	74184010	+	.	.	.	.	.	.	.	.	.	.	.	.	.	.	.	.	.	.	.	.
1375	2	EEF1A1	NM_001402	chr6	74282193	74287476	-	.	.	.	.	.	.	.	.	.	.	.	.	.	.	.	.	.	.	.	.
3295	1	CD109	NM_133493	chr6	74462234	74594761	+	.	.	.	.	.	.	.	.	.	.	.	.	.	.	.	.	.	.	.	.
3439	1	COX7A2	NM_001865	chr6	76004110	76010364	-	.	.	.	.	.	.	.	.	.	.	.	.	.	.	.	.	.	.	.	.
1745	2	SENP6	NM_015571	chr6	76368341	76484714	+	BH609556	chr6	76410880	+	.	.	.	.	.	.	.	.	.	.	.	.	.	.	.	.
4480	1	MYO6	NM_004999	chr6	76515628	76685974	+	.	.	.	.	.	.	.	.	.	.	.	.	.	.	.	.	.	.	.	.
6478	0,5	IMPG1	NM_001563	chr6	76687781	76839055	-	.	.	.	.	.	.	.	.	.	.	.	.	.	.	.	.	.	.	.	.

Table S2

tumor associated genes								HIV				MLV				MMTV				MMTV(SIN)				MMTV(SIN)arrest			
rank	score	gene symbol	RefSEq	chrom	txStart	txEnd	strand	integrant	chr	position	strand	integrant	chr	position	strand	integrant	chr	position	strand	integrant	chr	position	strand	integrant	chr	position	strand
7362	0,25	HTR1B	NM_000863	chr6	78228666	78229839	-	.	.	.	.	.	.	.	.	.	.	.	.	.	.	.	.	.	.	.	.
4163	1	IRAK1BP1	NM_001010844	chr6	79633907	79665039	+	.	.	.	.	.	.	.	.	.	.	.	.	.	.	.	.	.	.	.	.
1130	2,25	PHIP	NM_017934	chr6	79700854	79844730	-	CL800597	chr6	79748417	-	.	.	.	.	.	.	.	.	.	.	.	.	1988_177_1	chr6	79791063	-
1130	2,25	PHIP	NM_017934	chr6	79700854	79844730	-	AY517006.1	chr6	79778958	+	.	.	.	.	.	.	.	.	.	.	.	.	1988_177_1	chr6	79791063	-
765	2,75	TTK	NM_001166691	chr6	80771040	80808963	+	CL529490	chr6	80781571	-	.	.	.	.	.	.	.	.	.	.	.	.	.	.	.	.
3135	1	BCKDHB	NM_000056	chr6	80873062	81112706	+	.	.	.	.	.	.	.	.	.	.	.	.	.	.	.	.	.	.	.	.
6338	0,5	FAM46A	NM_017633	chr6	82512165	82519147	-	.	.	.	.	.	.	.	.	.	.	.	.	.	.	.	.	.	.	.	.
4106	1	IBTK	NM_015525	chr6	82936674	83014167	-	.	.	.	.	.	.	.	.	.	.	.	.	.	.	.	.	.	.	.	.
2808	1,25	TPBG	NM_006670	chr6	83129641	83133852	+	.	.	.	.	.	.	.	.	.	.	.	.	.	.	.	.	.	.	.	.
4775	1	PGM3	NM_015599	chr6	83931311	83959731	-	BH609557	chr6	83944169	+	.	.	.	.	.	.	.	.	.	.	.	.	.	.	.	.
4775	1	PGM3	NM_015599	chr6	83931311	83959731	-	CL528975	chr6	83943952	-	.	.	.	.	.	.	.	.	.	.	.	.	.	.	.	.
2250	1,5	ME1	NM_002395	chr6	83976828	84197657	-	CL528976	chr6	84001324	-	.	.	.	.	.	.	.	.	.	.	.	.	.	.	.	.
6941	0,5	SNAP91	NM_001242793	chr6	84319323	84475846	-	.	.	.	.	.	.	.	.	.	.	.	.	.	.	.	.	.	.	.	.
5127	1	RIPPLY2	NM_001009994	chr6	84619703	84623953	+	.	.	.	.	.	.	.	.	.	.	.	.	.	.	.	.	.	.	.	.
3500	1	CYB5R4	NM_016230	chr6	84626088	84726865	+	.	.	.	.	.	.	.	.	.	.	.	.	.	.	.	.	.	.	.	.
6626	0,5	MRAP2	NM_138409	chr6	84800138	84857324	+	.	.	.	.	.	.	.	.	.	.	.	.	.	.	.	.	.	.	.	.
1586	2	NT5E	NM_001204813	chr6	86216020	86262228	+	.	.	.	.	.	.	.	.	.	.	.	.	.	.	.	.	.	.	.	.
6457	0,5	HTR1E	NM_000865	chr6	87703742	87783116	+	.	.	.	.	.	.	.	.	.	.	.	.	.	.	.	.	.	.	.	.
2548	1,25	CGA	NM_000735	chr6	87851934	87861584	-	.	.	.	.	.	.	.	.	.	.	.	.	.	.	.	.	.	.	.	.
5065	1	RARS2	NM_020320	chr6	88280814	88356454	-	CL528978	chr6	88319422	+	.	.	.	.	.	.	.	.	.	.	.	.	.	.	.	.
2081	1,5	CNR1	NM_016083	chr6	88906303	88932486	-	.	.	.	.	.	.	.	.	.	.	.	.	.	.	.	.	.	.	.	.
3124	1	BACH2	NM_001170794	chr6	90692967	91063348	-	CL799858	chr6	91001100	+	.	.	.	.	.	.	.	.	.	.	.	.	.	.	.	.
2658	1,25	MAP3K7	NM_145333	chr6	91280012	91353741	-	CL799867	chr6	91349751	-	.	.	.	.	.	.	.	.	.	.	.	.	.	.	.	.
567	3	EPHA7	NM_004440	chr6	94006458	94186021	-	.	.	.	.	.	.	.	.	.	.	.	.	.	.	.	.	.	.	.	.
1414	2	FUT9	NM_006581	chr6	96570565	96770209	+	.	.	.	.	.	.	.	.	.	.	.	.	.	.	.	.	.	.	.	.
3424	1	COQ3	NM_017421	chr6	99924068	99948803	-	.	.	.	.	.	.	.	.	.	.	.	.	.	.	.	.	.	.	.	.
6603	0,5	MCHR2	NM_032503	chr6	100474506	100548835	-	.	.	.	.	.	.	.	.	.	.	.	.	.	.	.	.	.	.	.	.
5242	1	SIM1	NM_005068	chr6	100943470	101018272	-	.	.	.	.	.	.	.	.	.	.	.	.	.	.	.	.	.	.	.	.
1257	2	ASCC3	NM_006828	chr6	101063328	101435945	-	CL529450	chr6	101416738	+	.	.	.	.	.	.	.	.	371_194	chr6	101387341	+	.	.	.	.
1257	2	ASCC3	NM_006828	chr6	101063328	101435945	-	CL800156	chr6	101322716	+	.	.	.	.	.	.	.	.	371_194	chr6	101387341	+	.	.	.	.
1257	2	ASCC3	NM_006828	chr6	101063328	101435945	-	CL800056	chr6	101335553	-	.	.	.	.	.	.	.	.	371_194	chr6	101387341	+	.	.	.	.
1257	2	ASCC3	NM_006828	chr6	101063328	101435945	-	CL799813	chr6	101151067	-	.	.	.	.	.	.	.	.	371_194	chr6	101387341	+	.	.	.	.
1257	2	ASCC3	NM_006828	chr6	101063328	101435945	-	AY516913.1	chr6	101158786	-	.	.	.	.	.	.	.	.	371_194	chr6	101387341	+	.	.	.	.
1138	2,25	POPDC3	NR_024539	chr6	105712467	105730823	-	.	.	.	.	.	.	.	.	.	.	.	.	.	.	.	.	.	.	.	.
7489	0,25	PREP	NM_002726	chr6	105832134	105957692	-	.	.	.	.	.	.	.	.	.	.	.	.	.	.	.	.	.	.	.	.
1230	2	AIM1	NM_001624	chr6	107066422	107125027	+	.	.	.	.	.	.	.	.	.	.	.	.	.	.	.	.	.	.	.	.
908	2,5	NR2E1	NM_003269	chr6	108593907	108616706	+	.	.	.	.	.	.	.	.	.	.	.	.	.	.	.	.	.	.	.	.
65	6,5	FOXO3	NM_201559	chr6	108987718	109112664	+	.	.	.	.	.	.	.	.	.	.	.	.	.	.	.	.	.	.	.	.
7092	0,5	WASF1	NM_001024934	chr6	110527714	110607900	-	CL528982	chr6	110590859	+	.	.	.	.	.	.	.	.	.	.	.	.	.	.	.	.
1347	2	DDO	NM_003649	chr6	110820075	110843446	-	.	.	.	.	.	.	.	.	.	.	.	.	.	.	.	.	.	.	.	.
5259	1	SLC22A16	NM_033125	chr6	110852585	110904537	-	.	.	.	.	.	.	.	.	.	.	.	.	.	.	.	.	.	.	.	.
1244	2	AMD1	NM_001033059	chr6	111302680	111323606	+	.	.	.	.	.	.	.	.	.	.	.	.	.	.	.	.	.	.	.	.
3991	1	GTF3C6	NM_138408	chr6	111386455	111395784	+	.	.	.	.	.	.	.	.	.	.	.	.	.	.	.	.	.	.	.	.
2770	1,25	SLC16A10	NM_018593	chr6	111515473	111651299	+	.	.	.	.	.	.	.	.	.	.	.	.	.	.	.	.	.	.	.	.
5097	1	REV3L	NM_002912	chr6	111726926	111911107	-	CL800689	chr6	111773220	+	.	.	.	.	.	.	.	.	.	.	.	.	.	.	.	.
1047	2,25	FYN	NM_002037	chr6	112088227	112301348	-	AY516916.1	chr6	112242336	+	AY516769	chr6	112184413	-	.	.	.	.	.	.	.	.	.	.	.	.
1047	2,25	FYN	NM_002037	chr6	112088227	112301348	-	AY517356.1	chr6	112279292	-	AY516769	chr6	112184413	-	.	.	.	.	.	.	.	.	.	.	.	.
5700	1	WISP3	NM_003880	chr6	112481970	112497580	+	.	.	.	.	.	.	.	.	.	.	.	.	.	.	.	.	.	.	.	.
2228	1,5	LAMA4	NM_001105206	chr6	112535826	112682610	-	.	.	.	.	.	.	.	.	.	.	.	.	.	.	.	.	.	.	.	.
2244	1,5	MARCKS	NM_002356	chr6	114285219	114291345	+	.	.	.	.	.	.	.	.	.	.	.	.	.	.	.	.	.	.	.	.
182	4,5	HDAC2	NR_033441	chr6	114364012	114399052	-	AY517201.1	chr6	114381903	-	.	.	.	.	.	.	.	.	.	.	.	.	.	.	.	.

Table S2

tumor associated genes								HIV				MLV				MMTV				MMTV(SIN)				MMTV(SIN)arrest			
rank	score	gene symbol	RefSEq	chrom	txStart	txEnd	strand	integrant	chr	position	strand	integrant	chr	position	strand	integrant	chr	position	strand	integrant	chr	position	strand	integrant	chr	position	strand
4084	1	HS3ST5	NM_153612	chr6	114483442	114490734	-	.	.	.	.	.	.	.	.	.	.	.	.	.	.	.	.	.	.	.	.
2591	1,25	FRK	NM_002031	chr6	116369385	116488614	-	.	.	.	.	.	.	.	.	.	.	.	.	.	.	.	.	.	.	.	.
7056	0,5	TSPYL1	NM_003309	chr6	116702714	116707973	-	.	.	.	.	.	.	.	.	.	.	.	.	.	.	.	.	.	.	.	.
2111	1,5	DSE	NM_001080976	chr6	116707975	116866135	+	.	.	.	.	.	.	.	.	.	.	.	.	.	.	.	.	.	.	.	.
4244	1	KPNA5	NM_002269	chr6	117109059	117169723	+	CL529510	chr6	117149035	-	.	.	.	.	.	.	.	.	.	.	.	.	.	.	.	.
229	4,25	ROS1	NM_002944	chr6	117716222	117853711	-	.	.	.	.	.	.	.	.	.	.	.	.	686_128	chr6	117852470	+	.	.	.	.
1059	2,25	GOPC	NM_020399	chr6	117988125	118030398	-	.	.	.	.	.	.	.	.	.	.	.	.	.	.	.	.	.	.	.	.
7482	0,25	PLN	NM_002667	chr6	118976134	118988280	+	.	.	.	.	.	.	.	.	.	.	.	.	.	.	.	.	.	.	.	.
7175	0,25	ASF1A	NM_014034	chr6	119263627	119272034	+	.	.	.	.	.	.	.	.	.	.	.	.	.	.	.	.	.	.	.	.
7415	0,25	MCM9	NM_153255	chr6	119273460	119298026	-	.	.	.	.	.	.	.	.	.	.	.	.	.	.	.	.	.	.	.	.
3726	1	FAM184A	NM_001100411	chr6	119322694	119512057	-	.	.	.	.	.	.	.	.	D5_GVJ7CIC0	chr6	119324582	-	.	.	.	.	.	.	.	.
7426	0,25	MIR548B	NR_030315	chr6	119431910	119432007	-	.	.	.	.	.	.	.	.	.	.	.	.	.	.	.	.	.	.	.	.
4336	1	MAN1A1	NM_005907	chr6	119540064	119712630	-	CL528984	chr6	119594972	-	.	.	.	.	.	.	.	.	.	.	.	.	.	.	.	.
3236	1	C6orf170	NM_152730	chr6	121442325	121697343	-	.	.	.	.	.	.	.	.	.	.	.	.	.	.	.	.	.	.	.	.
469	3,25	GJA1	NM_000165	chr6	121798443	121812572	+	.	.	.	.	.	.	.	.	.	.	.	.	.	.	.	.	.	.	.	.
6874	0,5	SERINC1	NM_020755	chr6	122806191	122834651	-	.	.	.	.	.	.	.	.	.	.	.	.	.	.	.	.	.	.	.	.
1629	2	PKIB	NM_001270395	chr6	123015572	123089217	+	.	.	.	.	.	.	.	.	.	.	.	.	.	.	.	.	.	.	.	.
1907	1,75	FABP7	NM_001446	chr6	123142344	123146917	+	.	.	.	.	.	.	.	.	.	.	.	.	.	.	.	.	.	.	.	.
5391	1	STL	NR_026876	chr6	125271087	125325872	-	.	.	.	.	.	.	.	.	.	.	.	.	.	.	.	.	.	.	.	.
5141	1	RNF217	NM_152553	chr6	125346212	125446360	+	CL799947	chr6	125351597	-	.	.	.	.	.	.	.	.	.	.	.	.	.	.	.	.
5528	1	TPD52L1	NM_001003397	chr6	125516577	125626343	+	.	.	.	.	.	.	.	.	.	.	.	.	.	.	.	.	.	.	.	.
4036	1	HEY2	NM_012259	chr6	126112424	126124108	+	.	.	.	.	.	.	.	.	.	.	.	.	.	.	.	.	1516_151_1	chr6	126118362	-
2271	1,5	NCOA7	NM_181782	chr6	126153601	126294869	+	.	.	.	.	.	.	.	.	.	.	.	.	.	.	.	.	.	.	.	.
6834	0,5	RSPO3	NM_032784	chr6	127481740	127559877	+	.	.	.	.	.	.	.	.	.	.	.	.	.	.	.	.	.	.	.	.
658	3	PTPRK	NM_002844	chr6	128331616	128883512	-	BH609871	chr6	128453386	+	AY515943	chr6	128335745	-	.	.	.	.	2051_116	chr6	128741180	-	.	.	.	.
1930	1,75	LAMA2	NM_001079823	chr6	129245978	129879403	+	CL528986	chr6	129800541	+	AY515942	chr6	129547920	-	.	.	.	.	.	.	.	.	3087_288_1	chr6	129583617	-
1930	1,75	LAMA2	NM_001079823	chr6	129245978	129879403	+	CL529718	chr6	129815209	-	AY515942	chr6	129547920	-	.	.	.	.	.	.	.	.	3087_288_1	chr6	129583617	-
2502	1,25	ARG1	NM_001244438	chr6	131936036	131947165	+	.	.	.	.	.	.	.	.	.	.	.	.	.	.	.	.	.	.	.	.
3682	1	ENPP3	NM_005021	chr6	132000134	132110243	+	.	.	.	.	.	.	.	.	.	.	.	.	.	.	.	.	.	.	.	.
3681	1	ENPP1	NM_006208	chr6	132170848	132257988	+	CL529424	chr6	132241509	+	.	.	.	.	.	.	.	.	.	.	.	.	.	.	.	.
3481	1	CTGF	NM_001901	chr6	132311009	132314211	-	.	.	.	.	.	.	.	.	.	.	.	.	.	.	.	.	.	.	.	.
1939	1,75	MOXD1	NM_015529	chr6	132658886	132764357	-	.	.	.	.	.	.	.	.	.	.	.	.	.	.	.	.	.	.	.	.
5677	1	VNN1	NM_004666	chr6	133043689	133076887	-	.	.	.	.	.	.	.	.	.	.	.	.	.	.	.	.	.	.	.	.
5679	1	VNN3	NR_028290	chr6	133085618	133097597	-	.	.	.	.	.	.	.	.	.	.	.	.	.	.	.	.	.	.	.	.
5678	1	VNN2	NM_078488	chr6	133106701	133126291	-	.	.	.	.	.	.	.	.	.	.	.	.	.	.	.	.	.	.	.	.
1904	1,75	EYA4	NM_172103	chr6	133604187	133894951	+	.	.	.	.	.	.	.	.	_439_1_290	chr6	133622251	+	.	.	.	.	.	.	.	.
7580	0,25	TCF21	NM_198392	chr6	134251951	134258368	+	.	.	.	.	.	.	.	.	.	.	.	.	.	.	.	.	.	.	.	.
5437	1	TBPL1	NM_004865	chr6	134315993	134350331	+	.	.	.	.	.	.	.	.	.	.	.	.	.	.	.	.	.	.	.	.
5268	1	SLC2A12	NM_145176	chr6	134350411	134415482	-	.	.	.	.	.	.	.	.	.	.	.	.	.	.	.	.	.	.	.	.
679	3	SGK1	NM_001143676	chr6	134532076	134680889	-	.	.	.	.	.	.	.	.	.	.	.	.	.	.	.	.	.	.	.	.
625	3	MYB	NM_001161659	chr6	135544145	135582004	+	AY516946.1	chr6	135553837	-	.	.	.	.	.	.	.	.	.	.	.	.	606_112_1	chr6	135547863	-
2933	1	AHI1	NM_017651	chr6	135646802	135860596	-	AY516959.1	chr6	135794110	+	.	.	.	.	.	.	.	.	.	.	.	.	1824_167_2	chr6	135849823	+
4737	1	PDE7B	NM_018945	chr6	136214526	136558402	+	CL528988	chr6	136261608	-	.	.	.	.	.	.	.	.	.	.	.	.	.	.	.	.
2241	1,5	MAP7	NM_001198616	chr6	136705111	136913650	-	.	.	.	.	AY516012	chr6	136829553	-	.	.	.	.	.	.	.	.	.	.	.	.
1518	2	MAP3K5	NM_005923	chr6	136919879	137155349	-	BH609563	chr6	137015151	-	.	.	.	.	.	.	.	.	.	.	.	.	.	.	.	.
6466	0,5	IFNGR1	NM_000416	chr6	137560313	137582260	-	.	.	.	.	.	.	.	.	.	.	.	.	.	.	.	.	.	.	.	.
2443	1,5	TNFAIP3	NM_001270507	chr6	138230017	138246144	+	.	.	.	.	.	.	.	.	.	.	.	.	.	.	.	.	.	.	.	.
6525	0,5	KIAA1244	NM_020340	chr6	138524745	138707493	+	.	.	.	.	.	.	.	.	.	.	.	.	.	.	.	.	.	.	.	.
3373	1	CITED2	NM_006079	chr6	139735084	139737480	-	.	.	.	.	.	.	.	.	.	.	.	.	.	.	.	.	.	.	.	.
1119	2,25	NMBR	NM_002511	chr6	142438437	142451629	-	.	.	.	.	.	.	.	.	.	.	.	.	.	.	.	.	.	.	.	.
6433	0,5	HIVEP2	NM_006734	chr6	143114296	143308031	-	CL529489	chr6	143146352	-	.	.	.	.	.	.	.	.	.	.	.	.	.	.	.	.

Table S2

tumor associated genes								HIV				MLV				MMTV				MMTV(SIN)				MMTV(SIN)arrest			
rank	score	gene symbol	RefSEq	chrom	txStart	txEnd	strand	integrant	chr	position	strand	integrant	chr	position	strand	integrant	chr	position	strand	integrant	chr	position	strand	integrant	chr	position	strand
3814	1	FUCA2	NM_032020	chr6	143857641	143874713	-	.	.	.	.	.	.	.	.	.	.	.	.	.	.	.	.	.	.	.	.
4777	1	PHACTR2	NM_001100166	chr6	143971009	144194015	+	.	.	.	.	.	.	.	.	.	.	.	.	.	.	.	.	.	.	.	.
493	3,25	PLAGL1	NM_001080951	chr6	144303129	144427428	-	.	.	.	.	.	.	.	.	.	.	.	.	.	.	.	.	.	.	.	.
377	3,5	GRM1	NM_001114329	chr6	146390474	146800424	+	.	.	.	.	.	.	.	.	.	.	.	.	.	.	.	.	.	.	.	.
5951	0,75	RAB32	NM_006834	chr6	146906520	146917779	+	.	.	.	.	.	.	.	.	.	.	.	.	.	.	.	.	.	.	.	.
6986	0,5	STXBP5	NM_001127715	chr6	147567186	147753305	+	CL800039	chr6	147703932	+	.	.	.	.	.	.	.	.	.	.	.	.	.	.	.	.
5958	0,75	SASH1	NM_015278	chr6	148705421	148914877	+	.	.	.	.	.	.	.	.	.	.	.	.	.	.	.	.	.	.	.	.
5663	1	UST	NM_005715	chr6	149109963	149439819	+	BH609567	chr6	149308231	+	.	.	.	.	.	.	.	.	.	.	.	.	.	.	.	.
6989	0,5	SUMO4	NM_001002255	chr6	149763187	149763875	+	.	.	.	.	.	.	.	.	.	.	.	.	.	.	.	.	.	.	.	.
478	3,25	LATS1	NR_073033	chr6	150020981	150079254	-	.	.	.	.	.	.	.	.	.	.	.	.	.	.	.	.	.	.	.	.
2295	1,5	NUP43	NM_198887	chr6	150087149	150109381	-	.	.	.	.	.	.	.	.	.	.	.	.	.	.	.	.	.	.	.	.
6574	0,5	LRP11	NM_032832	chr6	150181586	150227173	-	.	.	.	.	.	.	.	.	.	.	.	.	.	.	.	.	.	.	.	.
5053	1	RAET1E	NM_001243328	chr6	150246203	150258888	-	.	.	.	.	.	.	.	.	.	.	.	.	.	.	.	.	.	.	.	.
5638	1	ULBP1	NM_025218	chr6	150326835	150336539	+	.	.	.	.	.	.	.	.	.	.	.	.	.	.	.	.	.	.	.	.
4911	1	PPP1R14C	NM_030949	chr6	150505880	150613221	+	.	.	.	.	.	.	.	.	.	.	.	.	.	.	.	.	.	.	.	.
4453	1	MTHFD1L	NM_001242768	chr6	151229161	151464716	+	.	.	.	.	.	.	.	.	.	.	.	.	.	.	.	.	.	.	.	.
708	2,75	AKAP12	NM_005100	chr6	151602826	151721390	+	.	.	.	.	.	.	.	.	.	.	.	.	.	.	.	.	.	.	.	.
177	4,5	ESR1	NM_001122740	chr6	152170146	152466101	+	.	.	.	.	.	.	.	.	.	.	.	.	.	.	.	.	.	.	.	.
5419	1	SYNE1	NM_033071	chr6	152484511	152999679	-	.	.	.	.	.	.	.	.	.	.	.	.	.	.	.	.	.	.	.	.
1181	2,25	VIP	NM_003381	chr6	153113624	153122596	+	.	.	.	.	.	.	.	.	.	.	.	.	.	.	.	.	.	.	.	.
6351	0,5	FBXO5	NM_012177	chr6	153333350	153345896	-	.	.	.	.	.	.	.	.	.	.	.	.	.	.	.	.	.	.	.	.
6480	0,5	IPCEF1	NM_001130699	chr6	154517309	154692907	-	CL799847	chr6	154633610	+	AY516091	chr6	154655334	+	.	.	.	.	.	.	.	.	.	.	.	.
3405	1	CNKSR3	NM_173515	chr6	154768124	154873445	-	.	.	.	.	.	.	.	.	.	.	.	.	.	.	.	.	.	.	.	.
7021	0,5	TIAM2	NM_012454	chr6	155453114	155620549	+	.	.	.	.	.	.	.	.	.	.	.	.	.	.	.	.	.	.	.	.
758	2,75	SYNJ2	NM_003898	chr6	158322875	158440195	+	.	.	.	.	AY516827	chr6	158383263	-	.	.	.	.	.	.	.	.	3255_329_1	chr6	158421796	+
3988	1	GTF2H5	NM_207118	chr6	158509366	158540364	+	.	.	.	.	.	.	.	.	.	.	.	.	.	.	.	.	.	.	.	.
1905	1,75	EZR	NM_001111077	chr6	159106760	159160444	-	.	.	.	.	.	.	.	.	.	.	.	.	.	.	.	.	.	.	.	.
755	2,75	SOD2	NM_001024466	chr6	160020138	160034343	-	.	.	.	.	.	.	.	.	.	.	.	.	.	.	.	.	.	.	.	.
2877	1	ACAT2	NM_005891	chr6	160102978	160120077	+	.	.	.	.	.	.	.	.	.	.	.	.	.	.	.	.	.	.	.	.
6757	0,5	PNLDC1	NM_173516	chr6	160141290	160161725	+	.	.	.	.	.	.	.	.	.	.	.	.	.	.	.	.	.	.	.	.
4359	1	MAS1	NM_002377	chr6	160247963	160249097	+	.	.	.	.	.	.	.	.	.	.	.	.	.	.	.	.	.	.	.	.
215	4,25	IGF2R	NM_000876	chr6	160310120	160447573	+	.	.	.	.	.	.	.	.	.	.	.	.	.	.	.	.	.	.	.	.
5257	1	SLC22A1	NM_003057	chr6	160462852	160499740	+	.	.	.	.	.	.	.	.	.	.	.	.	.	.	.	.	.	.	.	.
5260	1	SLC22A2	NM_003058	chr6	160557783	160599953	-	.	.	.	.	.	.	.	.	.	.	.	.	.	.	.	.	.	.	.	.
2771	1,25	SLC22A3	NM_021977	chr6	160689394	160793601	+	.	.	.	.	.	.	.	.	.	.	.	.	.	.	.	.	.	.	.	.
4296	1	LPA	NM_005577	chr6	160872504	161007397	-	.	.	.	.	.	.	.	.	.	.	.	.	.	.	.	.	.	.	.	.
4842	1	PLG	NM_000301	chr6	161043214	161095075	+	.	.	.	.	.	.	.	.	.	.	.	.	.	.	.	.	.	.	.	.
1517	2	MAP3K4	NM_006724	chr6	161332811	161458407	+	CL528994	chr6	161403935	-	.	.	.	.	.	.	.	.	.	.	.	.	.	.	.	.
2927	1	AGPAT4	NM_020133	chr6	161471046	161615097	-	.	.	.	.	.	.	.	.	.	.	.	.	.	.	.	.	.	.	.	.
490	3,25	PARK2	NM_004562	chr6	161688579	163068824	-	.	.	.	.	.	.	.	.	.	.	.	.	101_1_595_21	chr6	162587669	-	.	.	.	.
1707	2	QKI	NM_006775	chr6	163755664	163919618	+	CL800718	chr6	163809810	+	.	.	.	.	.	.	.	.	48_1_2026_6	chr6	163886652	-	.	.	.	.
1707	2	QKI	NM_006775	chr6	163755664	163919618	+	AY517219.1	chr6	163835471	-	.	.	.	.	.	.	.	.	48_1_2026_6	chr6	163886652	-	.	.	.	.
1707	2	QKI	NM_006775	chr6	163755664	163919618	+	CL799538	chr6	163906379	+	.	.	.	.	.	.	.	.	48_1_2026_6	chr6	163886652	-	.	.	.	.
1949	1,75	PDE10A	NR_045597	chr6	165660765	165995578	-	AY517278.1	chr6	165884146	+	.	.	.	.	.	.	.	.	.	.	.	.	2650_230_1	chr6	165720169	-
7578	0,25	T	NM_001270484	chr6	166491135	166502147	-	.	.	.	.	.	.	.	.	.	.	.	.	.	.	.	.	.	.	.	.
7492	0,25	PRR18	NM_175922	chr6	166639157	166641861	-	.	.	.	.	.	.	.	.	.	.	.	.	.	.	.	.	.	.	.	.
6880	0,5	SFT2D1	NM_145169	chr6	166653505	166675981	-	.	.	.	.	.	.	.	.	.	.	.	.	.	.	.	.	.	.	.	.
230	4,25	RPS6KA2	NM_021135	chr6	166742843	166960716	-	.	.	.	.	.	.	.	.	.	.	.	.	63_3_2150_8	chr6	167150781	-	.	.	.	.
5132	1	RNASET2	NM_003730	chr6	167262993	167290067	-	.	.	.	.	.	.	.	.	.	.	.	.	.	.	.	.	.	.	.	.
1404	2	FGFR1OP	NM_194429	chr6	167332805	167374056	+	CL528995	chr6	167346460	+	.	.	.	.	.	.	.	.	.	.	.	.	.	.	.	.
1404	2	FGFR1OP	NM_194429	chr6	167332805	167374056	+	CL800162	chr6	167348510	+	.	.	.	.	.	.	.	.	.	.	.	.	.	.	.	.

Table S2

tumor associated genes								HIV				MLV				MMTV				MMTV(SIN)				MMTV(SIN)arrest			
rank	score	gene symbol	RefSEq	chrom	txStart	txEnd	strand	integrant	chr	position	strand	integrant	chr	position	strand	integrant	chr	position	strand	integrant	chr	position	strand	integrant	chr	position	strand
3293	1	CCR6	NM_031409	chr6	167456230	167472619	+	.	.	.	.	.	.	.	.	.	.	.	.	.	.	.	.	.	.	.	.
623	3	MLLT4	NM_001207008	chr6	167970519	168115549	+	.	.	.	.	.	.	.	.	.	.	.	.	.	.	.	.	.	.	.	.
6532	0,5	KIF25	NM_005355	chr6	168161401	168188618	+	.	.	.	.	.	.	.	.	.	.	.	.	.	.	.	.	.	.	.	.
2800	1,25	THBS2	NM_003247	chr6	169357799	169396062	-	.	.	.	.	.	.	.	.	.	.	.	.	.	.	.	.	.	.	.	.
562	3	DLL1	NM_005618	chr6	170433218	170441622	-	.	.	.	.	.	.	.	.	.	.	.	.	.	.	.	.	.	.	.	.
1794	2	TBP	NM_003194	chr6	170705345	170723883	+	.	.	.	.	.	.	.	.	.	.	.	.	.	.	.	.	.	.	.	.
67	6,5	PDGFA	NM_002607	chr7	503422	526007	-	.	.	.	.	.	.	.	.	.	.	.	.	.	.	.	.	3151_305_1	chr7	507241	+
1685	2	PRKAR1B	NM_002735	chr7	555359	718687	-	.	.	.	.	AY516502	chr7	610321	+	.	.	.	.	.	.	.	.	.	.	.	.
4026	1	HEATR2	NM_017802	chr7	732863	792642	+	.	.	.	.	.	.	.	.	.	.	.	.	.	.	.	.	.	.	.	.
5993	0,5	ADAP1	NM_006869	chr7	904062	960815	-	.	.	.	.	.	.	.	.	.	.	.	.	.	.	.	.	.	.	.	.
3432	1	COX19	NM_001031617	chr7	971011	981761	-	.	.	.	.	.	.	.	.	.	.	.	.	.	.	.	.	.	.	.	.
3246	1	C7orf50	NM_001134395	chr7	1003148	1144419	-	CL528998	chr7	1087438	-	.	.	.	.	.	.	.	.	.	.	.	.	.	.	.	.
3949	1	GPR146	NM_138445	chr7	1063666	1065423	+	.	.	.	.	.	.	.	.	.	.	.	.	.	.	.	.	.	.	.	.
5741	1	ZFAND2A	NM_182491	chr7	1159068	1166381	-	.	.	.	.	.	.	.	.	.	.	.	.	.	.	.	.	.	.	.	.
4397	1	MICALL2	NM_182924	chr7	1440520	1465635	-	.	.	.	.	.	.	.	.	.	.	.	.	.	.	.	.	.	.	.	.
4154	1	INTS1	NM_001080453	chr7	1476438	1510544	-	CL528999	chr7	1492103	-	.	.	.	.	.	.	.	.	.	.	.	.	.	.	.	.
4327	1	MAFK	NM_002360	chr7	1536893	1549205	+	.	.	.	.	.	.	.	.	.	.	.	.	.	.	.	.	.	.	.	.
4974	1	PSMG3	NM_032302	chr7	1573494	1576155	-	.	.	.	.	.	.	.	.	.	.	.	.	.	.	.	.	.	.	.	.
1097	2,25	MAD1L1	NM_001013836	chr7	1821953	2239109	-	CL800447	chr7	1988410	+	.	.	.	.	.	.	.	.	.	.	.	.	.	.	.	.
3812	1	FTSJ2	NM_013393	chr7	2240451	2248359	-	.	.	.	.	.	.	.	.	.	.	.	.	.	.	.	.	.	.	.	.
489	3,25	NUDT1	NM_198953	chr7	2249065	2257306	+	.	.	.	.	.	.	.	.	.	.	.	.	.	.	.	.	.	.	.	.
5312	1	SNX8	NM_013321	chr7	2257930	2320625	-	.	.	.	.	.	.	.	.	.	.	.	.	.	.	.	.	.	.	.	.
3667	1	EIF3B	NM_003751	chr7	2360999	2386903	+	.	.	.	.	.	.	.	.	.	.	.	.	.	.	.	.	.	.	.	.
544	3	CHST12	NM_001243794	chr7	2409720	2440742	+	.	.	.	.	.	.	.	.	.	.	.	.	.	.	.	.	.	.	.	.
878	2,5	LFNG	NM_001166355	chr7	2518688	2534589	+	.	.	.	.	.	.	.	.	.	.	.	.	.	.	.	.	.	.	.	.
4161	1	IQCE	NM_152558	chr7	2565157	2620894	+	.	.	.	.	.	.	.	.	.	.	.	.	.	.	.	.	.	.	.	.
5587	1	TTYH3	NM_025250	chr7	2638128	2670962	+	.	.	.	.	.	.	.	.	.	.	.	.	.	.	.	.	.	.	.	.
1425	2	GNA12	NM_007353	chr7	2734266	2850485	-	.	.	.	.	.	.	.	.	.	.	.	.	.	.	.	.	.	.	.	.
537	3	CARD11	NM_032415	chr7	2912235	3050035	-	.	.	.	.	.	.	.	.	.	.	.	.	.	.	.	.	.	.	.	.
2589	1,25	FOXK1	NM_001037165	chr7	4688455	4777600	+	.	.	.	.	.	.	.	.	.	.	.	.	.	.	.	.	.	.	.	.
1715	2	RADIL	NM_018059	chr7	4805265	4889861	-	.	.	.	.	.	.	.	.	.	.	.	.	.	.	.	.	.	.	.	.
7461	0,25	PAPOLB	NM_020144	chr7	4863894	4868151	-	.	.	.	.	.	.	.	.	.	.	.	.	.	.	.	.	.	.	.	.
6618	0,5	MMD2	NM_001270375	chr7	4912145	4965370	-	.	.	.	.	.	.	.	.	.	.	.	.	.	.	.	.	.	.	.	.
2749	1,25	RBAK	NM_021163	chr7	5052078	5075645	+	.	.	.	.	.	.	.	.	.	.	.	.	.	.	.	.	.	.	.	.
2648	1,25	LOC389458	NR_015343	chr7	5078216	5079380	+	.	.	.	.	.	.	.	.	.	.	.	.	.	.	.	.	.	.	.	.
2828	1,25	WIPI2	NM_001033518	chr7	5196360	5240012	+	CL800755	chr7	5214466	+	.	.	.	.	.	.	.	.	.	.	.	.	.	.	.	.
2772	1,25	SLC29A4	NM_001040661	chr7	5289086	5310230	+	.	.	.	.	.	.	.	.	.	.	.	.	.	.	.	.	.	.	.	.
2805	1,25	TNRC18	NM_001080495	chr7	5312948	5429703	-	.	.	.	.	.	.	.	.	.	.	.	.	.	.	.	.	.	.	.	.
1396	2	FBXL18	NM_024963	chr7	5481953	5519925	-	.	.	.	.	.	.	.	.	.	.	.	.	.	.	.	.	.	.	.	.
1205	2	ACTB	NM_001101	chr7	5533304	5536758	-	.	.	.	.	.	.	.	.	.	.	.	.	.	.	.	.	.	.	.	.
3808	1	FSCN1	NM_003088	chr7	5598961	5612813	+	.	.	.	.	.	.	.	.	.	.	.	.	.	.	.	.	.	.	.	.
5140	1	RNF216	NM_207111	chr7	5626197	5787887	-	BH609576	chr7	5780229	+	.	.	.	.	.	.	.	.	.	.	.	.	.	.	.	.
5140	1	RNF216	NM_207111	chr7	5626197	5787887	-	CL800080	chr7	5771251	+	.	.	.	.	.	.	.	.	.	.	.	.	.	.	.	.
68	6,5	PMS2	NM_000535	chr7	5979395	6015263	-	.	.	.	.	.	.	.	.	.	.	.	.	.	.	.	.	.	.	.	.
1378	2	EIF2AK1	NM_014413	chr7	6028403	6065386	-	.	.	.	.	.	.	.	.	.	.	.	.	.	.	.	.	.	.	.	.
1847	2	USP42	NM_032172	chr7	6111075	6167720	+	.	.	.	.	.	.	.	.	.	.	.	.	.	.	.	.	.	.	.	.
3520	1	CYTH3	NM_004227	chr7	6167936	6278767	-	.	.	.	.	.	.	.	.	.	.	.	.	.	.	.	.	.	.	.	.
660	3	RAC1	NM_018890	chr7	6380650	6410123	+	.	.	.	.	.	.	.	.	.	.	.	.	.	.	.	.	.	.	.	.
3525	1	DAGLB	NM_139179	chr7	6415271	6454168	-	.	.	.	.	.	.	.	.	.	.	.	.	.	.	.	.	.	.	.	.
1488	2	KDELR2	NM_001100603	chr7	6467236	6490374	-	.	.	.	.	.	.	.	.	.	.	.	.	.	.	.	.	.	.	.	.
5740	1	ZDHHC4	NM_018106	chr7	6583589	6595135	+	.	.	.	.	.	.	.	.	.	.	.	.	.	.	.	.	.	.	.	.

Table S2

tumor associated genes								HIV				MLV				MMTV				MMTV(SIN)				MMTV(SIN)arrest			
rank	score	gene symbol	RefSEq	chrom	txStart	txEnd	strand	integrant	chr	position	strand	integrant	chr	position	strand	integrant	chr	position	strand	integrant	chr	position	strand	integrant	chr	position	strand
3238	1	C7orf26	NM_024067	chr7	6596439	6614880	+	.	.	.	.	.	.	.	.	.	.	.	.	.	.	.	.	.	.	.	.
5757	1	ZNF12	NM_016265	chr7	6694588	6713091	-	.	.	.	.	.	.	.	.	.	.	.	.	.	.	.	.	.	.	.	.
5171	1	RSPH10B	NM_173565	chr7	6760264	6804921	+	.	.	.	.	.	.	.	.	.	.	.	.	.	.	.	.	.	.	.	.
4401	1	MIOS	NM_019005	chr7	7573140	7613635	+	.	.	.	.	.	.	.	.	.	.	.	.	.	.	.	.	.	.	.	.
671	3	RPA3	NM_002947	chr7	7643099	7724763	-	.	.	.	.	.	.	.	.	.	.	.	.	.	.	.	.	.	.	.	.
3900	1	GLCCI1	NM_138426	chr7	7974898	8095234	+	.	.	.	.	.	.	.	.	.	.	.	.	.	.	.	.	.	.	.	.
6461	0,5	ICA1	NM_022307	chr7	8119339	8268207	-	.	.	.	.	.	.	.	.	.	.	.	.	.	.	.	.	.	.	.	.
1567	2	NDUFA4	NM_002489	chr7	10938104	10946338	-	.	.	.	.	.	.	.	.	.	.	.	.	.	.	.	.	.	.	.	.
4781	1	PHF14	NM_014660	chr7	10980023	11113901	+	AY517207.1	chr7	11048892	+	.	.	.	.	.	.	.	.	.	.	.	.	.	.	.	.
4781	1	PHF14	NM_014660	chr7	10980023	11113901	+	CL800390	chr7	11085678	-	.	.	.	.	.	.	.	.	.	.	.	.	.	.	.	.
7020	0,5	THSD7A	NM_015204	chr7	11376586	11838349	-	.	.	.	.	.	.	.	.	.	.	.	.	.	.	.	.	.	.	.	.
5486	1	TMEM106B	NM_001134232	chr7	12217372	12243415	+	CL529635	chr7	12221434	-	.	.	.	.	.	.	.	.	.	.	.	.	.	.	.	.
6853	0,5	SCIN	NM_001112706	chr7	12576727	12659753	+	.	.	.	.	.	.	.	.	.	.	.	.	.	.	.	.	.	.	.	.
3034	1	ARL4A	NM_001195396	chr7	12693435	12697083	+	.	.	.	.	.	.	.	.	.	.	.	.	.	.	.	.	.	.	.	.
178	4,5	ETV1	NM_001163149	chr7	13897380	13997390	-	.	.	.	.	.	.	.	.	.	.	.	.	.	.	.	.	.	.	.	.
174	4,5	DGKB	NM_004080	chr7	14151198	14847600	-	.	.	.	.	.	.	.	.	.	.	.	.	.	.	.	.	.	.	.	.
1108	2,25	MEOX2	NM_005924	chr7	15617361	15692833	-	.	.	.	.	.	.	.	.	.	.	.	.	.	.	.	.	.	.	.	.
6011	0,5	ANKMY2	NM_020319	chr7	16605925	16651967	-	BH609575	chr7	16631667	+	.	.	.	.	.	.	.	.	.	.	.	.	.	.	.	.
7605	0,25	TSPAN13	NM_014399	chr7	16759875	16790686	+	.	.	.	.	.	.	.	.	.	.	.	.	.	.	.	.	.	.	.	.
524	3	AHR	NM_001621	chr7	17304800	17352300	+	.	.	.	.	.	.	.	.	.	.	.	.	.	.	.	.	.	.	.	.
6947	0,5	SNX13	NM_015132	chr7	17796909	17946656	-	AY517259.1	chr7	17825832	+	.	.	.	.	.	.	.	.	.	.	.	.	.	.	.	.
4955	1	PRPS1L1	NM_175886	chr7	18032924	18034011	-	.	.	.	.	.	.	.	.	.	.	.	.	.	.	.	.	.	.	.	.
1445	2	HDAC9	NM_001204147	chr7	18515424	18674991	+	CL529001	chr7	18488201	-	AY516636	chr7	18516010	-	.	.	.	.	.	.	.	.	.	.	.	.
1445	2	HDAC9	NM_001204147	chr7	18515424	18674991	+	CL799810	chr7	18460422	-	AY516636	chr7	18516010	-	.	.	.	.	.	.	.	.	.	.	.	.
1445	2	HDAC9	NM_001204147	chr7	18515424	18674991	+	CL800827	chr7	18697585	+	AY516636	chr7	18516010	-	.	.	.	.	.	.	.	.	.	.	.	.
1445	2	HDAC9	NM_001204147	chr7	18515424	18674991	+	CL529002	chr7	18880105	-	AY516636	chr7	18516010	-	.	.	.	.	.	.	.	.	.	.	.	.
5972	0,75	TWIST1	NM_000474	chr7	19121615	19123820	-	.	.	.	.	.	.	.	.	.	.	.	.	.	.	.	.	.	.	.	.
5927	0,75	ITGB8	NM_002214	chr7	20337249	20421907	+	.	.	.	.	.	.	.	.	.	.	.	.	.	.	.	.	.	.	.	.
2852	1	ABCB5	NM_001163942	chr7	20653490	20666542	+	.	.	.	.	.	.	.	.	.	.	.	.	.	.	.	.	.	.	.	.
3595	1	DNAH11	NM_003777	chr7	21549357	21907982	+	.	.	.	.	.	.	.	.	.	.	.	.	.	.	.	.	.	.	.	.
596	3	IL6	NM_000600	chr7	22733290	22738146	+	.	.	.	.	.	.	.	.	.	.	.	.	.	.	.	.	.	.	.	.
6696	0,5	NUPL2	NM_007342	chr7	23187970	23207155	+	.	.	.	.	.	.	.	.	.	.	.	.	.	.	.	.	.	.	.	.
5915	0,75	GPNMB	NM_001005340	chr7	23252840	23281254	+	.	.	.	.	.	.	.	.	.	.	.	.	.	.	.	.	.	.	.	.
4122	1	IGF2BP3	NM_006547	chr7	23316352	23476520	-	.	.	.	.	.	.	.	.	.	.	.	.	.	.	.	.	.	.	.	.
5382	1	STK31	NM_001260505	chr7	23716310	23838655	+	.	.	.	.	.	.	.	.	.	.	.	.	.	.	.	.	.	.	.	.
1121	2,25	NPY	NM_000905	chr7	24290331	24298009	+	.	.	.	.	.	.	.	.	.	.	.	.	.	.	.	.	.	.	.	.
6709	0,5	OSBPL3	NM_145320	chr7	24802688	24986285	-	CL529524	chr7	24846057	+	.	.	.	.	.	.	.	.	.	.	.	.	.	.	.	.
1023	2,25	CYCS	NM_018947	chr7	25124794	25131505	-	.	.	.	.	.	.	.	.	.	.	.	.	.	.	.	.	.	.	.	.
3242	1	C7orf31	NM_138811	chr7	25140840	25186342	-	.	.	.	.	.	.	.	.	.	.	.	.	.	.	.	.	.	.	.	.
6668	0,5	NFE2L3	NM_004289	chr7	26158371	26193281	+	.	.	.	.	.	.	.	.	.	.	.	.	.	.	.	.	.	.	.	.
1070	2,25	HNRNPA2B1	NM_002137	chr7	26196080	26206938	-	.	.	.	.	.	.	.	.	.	.	.	.	.	.	.	.	.	.	.	.
6945	0,5	SNX10	NR_037670	chr7	26298039	26380474	+	.	.	.	.	.	.	.	.	.	.	.	.	.	.	.	.	.	.	.	.
6894	0,5	SKAP2	NM_003930	chr7	26673212	26870866	-	CL529003	chr7	26836503	-	.	.	.	.	.	.	.	.	.	.	.	.	.	.	.	.
2615	1,25	HOXA1	NM_005522	chr7	27099138	27102150	-	.	.	.	.	.	.	.	.	.	.	.	.	.	.	.	.	.	.	.	.
6445	0,5	HOXA2	NM_006735	chr7	27106497	27108919	-	.	.	.	.	.	.	.	.	.	.	.	.	.	.	.	.	.	.	.	.
4068	1	HOXA3	NM_153631	chr7	27112333	27133164	-	.	.	.	.	.	.	.	.	.	.	.	.	.	.	.	.	.	.	.	.
6446	0,5	HOXA4	NM_002141	chr7	27134650	27136924	-	.	.	.	.	.	.	.	.	.	.	.	.	.	.	.	.	.	.	.	.
5917	0,75	HOXA5	NM_019102	chr7	27147195	27149812	-	.	.	.	.	.	.	.	.	.	.	.	.	.	.	.	.	.	.	.	.
2190	1,5	HOXA7	NM_006896	chr7	27159862	27162821	-	.	.	.	.	.	.	.	.	.	.	.	.	.	.	.	.	.	.	.	.
183	4,5	HOXA9	NM_152739	chr7	27168581	27171674	-	.	.	.	.	.	.	.	.	.	.	.	.	.	.	.	.	.	.	.	.
2189	1,5	HOXA10	NM_018951	chr7	27176734	27180480	-	.	.	.	.	.	.	.	.	.	.	.	.	.	.	.	.	.	.	.	.

Table S2

tumor associated genes								HIV				MLV				MMTV				MMTV(SIN)				MMTV(SIN)arrest			
rank	score	gene symbol	RefSEq	chrom	txStart	txEnd	strand	integrant	chr	position	strand	integrant	chr	position	strand	integrant	chr	position	strand	integrant	chr	position	strand	integrant	chr	position	strand
473	3,25	HOXA11	NM_005523	chr7	27187300	27191360	-	.	.	.	.	.	.	.	.	.	.	.	.	.	.	.	.	.	.	.	.
587	3	HOXA13	NM_000522	chr7	27203023	27206250	-	.	.	.	.	.	.	.	.	.	.	.	.	.	.	.	.	.	.	.	.
4041	1	HIBADH	NM_152740	chr7	27531583	27669145	-	.	.	.	.	.	.	.	.	.	.	.	.	.	.	.	.	.	.	.	.
1487	2	JAZF1	NM_175061	chr7	27836717	28186962	-	.	.	.	.	.	.	.	.	.	.	.	.	.	.	.	.	.	.	.	.
6216	0,5	CREB5	NM_182898	chr7	28418668	28832036	+	.	.	.	.	.	.	.	.	.	.	.	.	.	.	.	.	3182_311_1	chr7	28714514	-
4839	1	PLEKHA8	NM_032639	chr7	30034501	30096884	+	.	.	.	.	.	.	.	.	.	.	.	.	.	.	.	.	.	.	.	.
6107	0,5	C7orf41	NM_152793	chr7	30141076	30168906	+	.	.	.	.	.	.	.	.	.	.	.	.	.	.	.	.	.	.	.	.
2597	1,25	GARS	NM_002047	chr7	30600705	30640173	+	.	.	.	.	.	.	.	.	.	.	.	.	.	.	.	.	.	.	.	.
4149	1	INMT	NM_006774	chr7	30758275	30763743	+	.	.	.	.	.	.	.	.	.	.	.	.	.	.	.	.	.	.	.	.
1249	2	AQP1	NM_001185060	chr7	30927439	30931656	+	.	.	.	.	.	.	.	.	.	.	.	.	.	.	.	.	.	.	.	.
7154	0,25	ADCYAP1R1	NM_001199637	chr7	31058600	31117618	+	.	.	.	.	.	.	.	.	.	.	.	.	.	.	.	.	.	.	.	.
6665	0,5	NEUROD6	NM_022728	chr7	31343604	31347063	-	.	.	.	.	.	.	.	.	.	.	.	.	.	.	.	.	.	.	.	.
4731	1	PDE1C	NM_001191056	chr7	31795771	32076991	-	.	.	.	.	.	.	.	.	.	.	.	.	.	.	.	.	.	.	.	.
1407	2	FKBP9	NM_007270	chr7	32963529	33013068	+	.	.	.	.	.	.	.	.	.	.	.	.	.	.	.	.	.	.	.	.
4634	1	NT5C3	NM_001166118	chr7	33020249	33047302	-	.	.	.	.	.	.	.	.	.	.	.	.	.	.	.	.	.	.	.	.
1997	1,5	ANLN	NM_018685	chr7	36395956	36459925	+	.	.	.	.	.	.	.	.	.	.	.	.	.	.	.	.	.	.	.	.
953	2,5	SFRP4	NM_003014	chr7	37912059	37923050	-	.	.	.	.	.	.	.	.	.	.	.	.	.	.	.	.	.	.	.	.
5374	1	STARD3NL	NM_032016	chr7	38184332	38236797	+	.	.	.	.	.	.	.	.	.	.	.	.	.	.	.	.	.	.	.	.
7160	0,25	AMPH	NM_139316	chr7	38389821	38637692	-	.	.	.	.	.	.	.	.	.	.	.	.	.	.	.	.	.	.	.	.
4884	1	POU6F2	NM_007252	chr7	38984133	39470915	+	.	.	.	.	.	.	.	.	.	.	.	.	.	.	.	.	.	.	.	.
5057	1	RALA	NM_005402	chr7	39629676	39714248	+	.	.	.	.	.	.	.	.	.	.	.	.	.	.	.	.	.	.	.	.
1081	2,25	INHBA	NM_002192	chr7	41695125	41709231	-	.	.	.	.	.	.	.	.	.	.	.	.	.	.	.	.	.	.	.	.
847	2,5	GLI3	NM_000168	chr7	41967072	42243143	-	.	.	.	.	.	.	.	.	.	.	.	.	.	.	.	.	102_77_1	chr7	42198877	-
4970	1	PSMA2	NM_002787	chr7	42922986	42938330	-	.	.	.	.	.	.	.	.	.	.	.	.	.	.	.	.	.	.	.	.
854	2,5	HECW1	NM_015052	chr7	43118722	43569463	+	.	.	.	.	AY516734	chr7	43257069	-	.	.	.	.	.	.	.	.	.	.	.	.
854	2,5	HECW1	NM_015052	chr7	43118722	43569463	+	.	.	.	.	AY516735	chr7	43257069	-	.	.	.	.	.	.	.	.	.	.	.	.
5377	1	STK17A	NM_004760	chr7	43589216	43633503	+	AY517047.1	chr7	43617264	+	.	.	.	.	.	.	.	.	.	.	.	.	.	.	.	.
3151	1	BLVRA	NM_001253823	chr7	43764796	43813466	+	CL800356	chr7	43811401	-	.	.	.	.	.	.	.	.	.	.	.	.	.	.	.	.
4764	1	PGAM2	NM_000290	chr7	44068850	44071711	-	.	.	.	.	.	.	.	.	.	.	.	.	.	.	.	.	.	.	.	.
1137	2,25	POLM	NM_013284	chr7	44078371	44088654	-	.	.	.	.	.	.	.	.	.	.	.	.	.	.	.	.	.	.	.	.
1642	2	POLD2	NM_001127218	chr7	44120803	44129694	-	.	.	.	.	.	.	.	.	.	.	.	.	.	.	.	.	.	.	.	.
1423	2	GCK	NM_000162	chr7	44150394	44195547	-	.	.	.	.	.	.	.	.	.	.	.	.	.	.	.	.	.	.	.	.
799	2,5	CAMK2B	NM_001220	chr7	44223273	44331755	-	.	.	.	.	.	.	.	.	.	.	.	.	.	.	.	.	.	.	.	.
4643	1	NUDCD3	NM_015332	chr7	44388489	44496910	-	BH609572	chr7	44456832	+	.	.	.	.	.	.	.	.	.	.	.	.	.	.	.	.
3555	1	DDX56	NM_019082	chr7	44571540	44580662	-	.	.	.	.	.	.	.	.	.	.	.	.	.	.	.	.	.	.	.	.
4658	1	OGDH	NM_001003941	chr7	44612645	44682720	+	CL529486	chr7	44640503	-	.	.	.	.	.	.	.	.	.	.	.	.	.	.	.	.
4658	1	OGDH	NM_001003941	chr7	44612645	44682720	+	CL799523	chr7	44697613	-	.	.	.	.	.	.	.	.	.	.	.	.	.	.	.	.
4658	1	OGDH	NM_001003941	chr7	44612645	44682720	+	CL529004	chr7	44696589	+	.	.	.	.	.	.	.	.	.	.	.	.	.	.	.	.
5949	0,75	PPIA	NM_021130	chr7	44802765	44809241	+	.	.	.	.	.	.	.	.	.	.	.	.	.	.	.	.	.	.	.	.
4478	1	MYO1G	NM_033054	chr7	44968784	44985229	-	.	.	.	.	.	.	.	.	.	.	.	.	.	.	.	.	.	.	.	.
354	3,5	ADCY1	NM_021116	chr7	45580649	45729239	+	.	.	.	.	.	.	.	.	.	.	.	.	.	.	.	.	.	.	.	.
2199	1,5	IGFBP1	NM_000596	chr7	45894483	45899792	+	.	.	.	.	.	.	.	.	.	.	.	.	.	.	.	.	.	.	.	.
268	4	IGFBP3	NM_000598	chr7	45918368	45927396	-	.	.	.	.	.	.	.	.	.	.	.	.	.	.	.	.	.	.	.	.
2197	1,5	HUS1	NR_037917	chr7	47969409	47985747	-	.	.	.	.	.	.	.	.	.	.	.	.	.	.	.	.	.	.	.	.
2822	1,25	UPP1	NM_181597	chr7	48094879	48114855	+	.	.	.	.	.	.	.	.	.	.	.	.	.	.	.	.	.	.	.	.
515	3	ABCA13	NM_152701	chr7	48208388	48657637	+	.	.	.	.	.	.	.	.	.	.	.	.	.	.	.	.	3127_298_1	chr7	48227957	+
7618	0,25	VWC2	NM_198570	chr7	49783802	49922684	+	.	.	.	.	.	.	.	.	.	.	.	.	.	.	.	.	.	.	.	.
1466	2	IKZF1	NM_001220776	chr7	50314923	50440292	+	.	.	.	.	.	.	.	.	.	.	.	.	.	.	.	.	.	.	.	.
3543	1	DDC	NM_001242886	chr7	50493627	50596262	-	.	.	.	.	.	.	.	.	.	.	.	.	.	.	.	.	.	.	.	.
728	2,75	GRB10	NM_001001550	chr7	50625253	50740492	-	.	.	.	.	.	.	.	.	.	.	.	.	.	.	.	.	.	.	.	.
2762	1,25	SEC61G	NM_014302	chr7	54787433	54794433	-	.	.	.	.	.	.	.	.	.	.	.	.	.	.	.	.	.	.	.	.

Table S2

tumor associated genes								HIV				MLV				MMTV				MMTV(SIN)				MMTV(SIN)arrest			
rank	score	gene symbol	RefSEq	chrom	txStart	txEnd	strand	integrant	chr	position	strand	integrant	chr	position	strand	integrant	chr	position	strand	integrant	chr	position	strand	integrant	chr	position	strand
3	15,75	EGFR	NM_201282	chr7	55054218	55203822	+	.	.	.	.	AY516339	chr7	55109025	-	.	.	.	.	.	.	.	.	.	.	.	.
2646	1,25	LANCL2	NM_018697	chr7	55400634	55468929	+	CL800649	chr7	55446828	-	.	.	.	.	.	.	.	.	.	.	.	.	.	.	.	.
2153	1,5	GBAS	NM_001202469	chr7	55999763	56035369	+	.	.	.	.	.	.	.	.	.	.	.	.	.	.	.	.	.	.	.	.
1697	2	PSPH	NM_004577	chr7	56046237	56086762	-	.	.	.	.	.	.	.	.	.	.	.	.	.	.	.	.	.	.	.	.
5410	1	SUMF2	NM_001042469	chr7	56099410	56115859	+	.	.	.	.	.	.	.	.	.	.	.	.	.	.	.	.	.	.	.	.
4787	1	PHKG1	NM_001258459	chr7	56115469	56128183	-	.	.	.	.	.	.	.	.	.	.	.	.	.	.	.	.	.	.	.	.
7634	0,25	ZNF92	NM_007139	chr7	64476202	64503433	+	.	.	.	.	.	.	.	.	.	.	.	.	.	.	.	.	.	.	.	.
3996	1	GUSB	NM_000181	chr7	65063107	65084736	-	.	.	.	.	.	.	.	.	.	.	.	.	.	.	.	.	.	.	.	.
787	2,5	ASL	NM_001024944	chr7	65178268	65195764	+	.	.	.	.	.	.	.	.	.	.	.	.	.	.	.	.	.	.	.	.
1739	2	SBDS	NM_016038	chr7	66090124	66098023	-	.	.	.	.	.	.	.	.	.	.	.	.	.	.	.	.	.	.	.	.
2460	1,5	WBSCR17	NM_022479	chr7	70235724	70816520	+	.	.	.	.	.	.	.	.	.	.	.	.	.	.	.	.	.	.	.	.
6762	0,5	POM121	NM_172020	chr7	71999407	72056779	+	.	.	.	.	.	.	.	.	.	.	.	.	.	.	.	.	.	.	.	.
6367	0,5	FZD9	NM_003508	chr7	72486044	72488386	+	.	.	.	.	.	.	.	.	.	.	.	.	.	.	.	.	.	.	.	.
7185	0,25	BCL7B	NM_001197244	chr7	72588618	72610001	-	.	.	.	.	.	.	.	.	.	.	.	.	.	.	.	.	.	.	.	.
5687	1	WBSCR22	NR_045512	chr7	72735833	72750487	+	.	.	.	.	.	.	.	.	.	.	.	.	.	.	.	.	.	.	.	.
5395	1	STX1A	NM_001165903	chr7	72751470	72771953	-	.	.	.	.	.	.	.	.	.	.	.	.	.	.	.	.	.	.	.	.
1312	2	CLDN3	NM_001306	chr7	72821262	72822536	-	.	.	.	.	.	.	.	.	.	.	.	.	.	.	.	.	.	.	.	.
1013	2,25	CLDN4	NM_001305	chr7	72883128	72884951	+	.	.	.	.	.	.	.	.	.	.	.	.	.	.	.	.	.	.	.	.
1381	2	ELN	NM_001081754	chr7	73080362	73122172	+	.	.	.	.	.	.	.	.	.	.	.	.	.	.	.	.	.	.	.	.
4283	1	LIMK1	NM_001204426	chr7	73145421	73174791	+	.	.	.	.	AY516872	chr7	73150915	-	.	.	.	.	.	.	.	.	.	.	.	.
5098	1	RFC2	NM_181471	chr7	73283767	73306674	-	.	.	.	.	.	.	.	.	.	.	.	.	.	.	.	.	.	.	.	.
6191	0,5	CLIP2	NM_003388	chr7	73341740	73458209	+	.	.	.	.	.	.	.	.	.	.	.	.	.	.	.	.	.	.	.	.
4507	1	NCF1	NM_000265	chr7	73826244	73841595	+	.	.	.	.	.	.	.	.	.	.	.	.	.	.	.	.	.	.	.	.
1066	2,25	HIP1	NM_001243198	chr7	75000554	75206219	-	.	.	.	.	.	.	.	.	.	.	.	.	.	.	.	.	.	.	.	.
1106	2,25	MDH2	NM_005918	chr7	75515328	75533866	+	.	.	.	.	.	.	.	.	.	.	.	.	.	.	.	.	.	.	.	.
1073	2,25	HSPB1	NM_001540	chr7	75769810	75771550	+	.	.	.	.	.	.	.	.	.	.	.	.	.	.	.	.	.	.	.	.
5727	1	YWHAG	NM_012479	chr7	75794043	75826278	-	.	.	.	.	.	.	.	.	.	.	.	.	.	.	.	.	.	.	.	.
5867	1	ZP3	NM_007155	chr7	75864776	75909324	+	.	.	.	.	.	.	.	.	.	.	.	.	.	.	.	.	.	.	.	.
3615	1	DTX2	NM_001102596	chr7	75947762	75973248	+	.	.	.	.	.	.	.	.	.	.	.	.	.	.	.	.	.	.	.	.
7477	0,25	PION	NM_017439	chr7	76778003	76883653	-	.	.	.	.	.	.	.	.	.	.	.	.	.	.	.	.	.	.	.	.
4993	1	PTPN12	NM_001131008	chr7	77005287	77107324	+	CL800367	chr7	77073012	-	.	.	.	.	.	.	.	.	.	.	.	.	.	.	.	.
4331	1	MAGI2	NM_012301	chr7	77484309	78920826	-	.	.	.	.	.	.	.	.	_284_1_185	chr7	78223190	+	.	.	.	.	.	.	.	.
376	3,5	GNAI1	NM_002069	chr7	79602075	79686661	+	.	.	.	.	.	.	.	.	.	.	.	.	.	.	.	.	.	.	.	.
3919	1	GNAT3	NM_001102386	chr7	79925922	79979178	-	.	.	.	.	AY516385	chr7	79945582	+	.	.	.	.	.	.	.	.	.	.	.	.
3919	1	GNAT3	NM_001102386	chr7	79925922	79979178	-	.	.	.	.	AY516145	chr7	79941813	+	.	.	.	.	.	.	.	.	.	.	.	.
1294	2	CD36	NM_001127443	chr7	80113580	80141670	+	.	.	.	.	AY516281	chr7	80109777	+	.	.	.	.	.	.	.	.	.	.	.	.
1743	2	SEMA3C	NM_006379	chr7	80209789	80386603	-	.	.	.	.	.	.	.	.	.	.	.	.	.	.	.	.	.	.	.	.
213	4,25	HGF	NM_001010931	chr7	81209942	81237388	-	CL529008	chr7	81225869	-	AY516177	chr7	81172672	-	.	.	.	.	.	.	.	.	.	.	.	.
3263	1	CACNA2D1	NM_000722	chr7	81417353	81910967	-	CL800757	chr7	81493712	+	AY516182	chr7	81721958	-	.	.	.	.	.	.	.	.	.	.	.	.
6724	0,5	PCLO	NM_014510	chr7	82287731	82630133	-	.	.	.	.	.	.	.	.	.	.	.	.	.	.	.	.	.	.	.	.
6867	0,5	SEMA3E	NM_001178129	chr7	82831157	83108683	-	.	.	.	.	.	.	.	.	.	.	.	.	.	.	.	.	.	.	.	.
6866	0,5	SEMA3A	NM_006080	chr7	83425594	83662153	-	.	.	.	.	AY516474	chr7	83475933	-	.	.	.	.	.	.	.	.	.	.	.	.
5207	1	SEMA3D	NM_152754	chr7	84462807	84589183	-	.	.	.	.	.	.	.	.	.	.	.	.	.	.	.	.	.	.	.	.
851	2,5	GRM3	NM_000840	chr7	86111165	86332128	+	.	.	.	.	.	.	.	.	.	.	.	.	.	.	.	.	.	.	.	.
7285	0,25	DMTF1	NM_021145	chr7	86619805	86663584	+	.	.	.	.	.	.	.	.	.	.	.	.	.	.	.	.	.	.	.	.
2851	1	ABCB4	NM_000443	chr7	86869296	86942955	-	.	.	.	.	.	.	.	.	.	.	.	.	.	.	.	.	.	.	.	.
769	2,5	ABCB1	NM_000927	chr7	86971114	87180575	-	.	.	.	.	.	.	.	.	.	.	.	.	.	.	.	.	.	.	.	.
6255	0,5	DBF4	NM_006716	chr7	87343479	87376792	+	.	.	.	.	.	.	.	.	.	.	.	.	.	.	.	.	.	.	.	.
1209	2	ADAM22	NM_021723	chr7	87401501	87670140	+	.	.	.	.	.	.	.	.	.	.	.	.	.	.	.	.	.	.	.	.
7573	0,25	SRI	NM_198901	chr7	87672367	87694244	-	.	.	.	.	.	.	.	.	.	.	.	.	.	.	.	.	.	.	.	.
2421	1,5	STEAP1	NM_012449	chr7	89621624	89632077	+	.	.	.	.	.	.	.	.	.	.	.	.	.	.	.	.	.	.	.	.

Table S2

tumor associated genes								HIV				MLV				MMTV				MMTV(SIN)				MMTV(SIN)arrest			
rank	score	gene symbol	RefSeq	chrom	txStart	txEnd	strand	integrant	chr	position	strand	integrant	chr	position	strand	integrant	chr	position	strand	integrant	chr	position	strand	integrant	chr	position	strand
6982	0,5	STEAP2	NM_001040665	chr7	89679109	89704928	+	.	.	.	.	.	.	.	.	.	.	.	.	.	.	.	.	.	.	.	.
1415	2	FZD1	NM_003505	chr7	90731718	90736068	+	.	.	.	.	.	.	.	.	.	.	.	.	.	.	.	.	.	.	.	.
525	3	AKAP9	NM_005751	chr7	91408124	91577923	+	.	.	.	.	.	.	.	.	.	.	.	.	s434_115	chr7	91461994	+	.	.	.	.
1341	2	CYP51A1	NM_000786	chr7	91579398	91601776	-	.	.	.	.	.	.	.	.	.	.	.	.	.	.	.	.	.	.	.	.
1496	2	KRIT1	NM_194456	chr7	91666218	91713350	-	.	.	.	.	.	.	.	.	.	.	.	.	.	.	.	.	.	.	.	.
2986	1	ANKIB1	NM_019004	chr7	91713483	91868634	+	.	.	.	.	.	.	.	.	.	.	.	.	.	.	.	.	.	.	.	.
7326	0,25	GATAD1	NR_052016	chr7	91914697	91927317	+	.	.	.	.	.	.	.	.	.	.	.	.	.	.	.	.	.	.	.	.
7474	0,25	PEX1	NM_000466	chr7	91954272	91995781	-	.	.	.	.	.	.	.	.	.	.	.	.	.	.	.	.	.	.	.	.
54	7	CDK6	NM_001259	chr7	92072170	92301167	-	CL529670	chr7	92226006	+	.	.	.	.	.	.	.	.	.	.	.	.	.	.	.	.
54	7	CDK6	NM_001259	chr7	92072170	92301167	-	AY516915.1	chr7	92104548	-	.	.	.	.	.	.	.	.	.	.	.	.	.	.	.	.
54	7	CDK6	NM_001259	chr7	92072170	92301167	-	CL529669	chr7	92135079	+	.	.	.	.	.	.	.	.	.	.	.	.	.	.	.	.
54	7	CDK6	NM_001259	chr7	92072170	92301167	-	AY517016.1	chr7	92227823	+	.	.	.	.	.	.	.	.	.	.	.	.	.	.	.	.
2530	1,25	CALCR	NM_001742	chr7	92891734	93041978	-	.	.	.	.	.	.	.	.	.	.	.	.	.	.	.	.	.	.	.	.
966	2,5	TFPI2	NM_006528	chr7	93353680	93358001	-	.	.	.	.	.	.	.	.	.	.	.	.	.	.	.	.	.	.	.	.
3927	1	GNGT1	NM_021955	chr7	93373755	93378421	+	.	.	.	.	.	.	.	.	.	.	.	.	.	.	.	.	.	.	.	.
3924	1	GNG11	NM_004126	chr7	93388951	93393762	+	.	.	.	.	.	.	.	.	.	.	.	.	.	.	.	.	.	.	.	.
6057	0,5	BET1	NM_005868	chr7	93458935	93471626	-	.	.	.	.	.	.	.	.	.	.	.	.	.	.	.	.	.	.	.	.
547	3	COL1A2	NM_000089	chr7	93861808	93898480	+	.	.	.	.	.	.	.	.	.	.	.	.	.	.	.	.	.	.	.	.
3280	1	CASD1	NM_022900	chr7	93977105	94024264	+	.	.	.	.	.	.	.	.	.	.	.	.	.	.	.	.	.	.	.	.
6729	0,5	PEG10	NM_015068	chr7	94123572	94136942	+	.	.	.	.	.	.	.	.	.	.	.	.	.	.	.	.	.	.	.	.
4920	1	PPP1R9A	NM_017650	chr7	94374884	94763663	+	.	.	.	.	.	.	.	.	.	.	.	.	.	.	.	.	.	.	.	.
4876	1	PON1	NM_000446	chr7	94765604	94791820	-	.	.	.	.	.	.	.	.	.	.	.	.	.	.	.	.	.	.	.	.
4878	1	PON3	NM_000940	chr7	94827119	94863623	-	.	.	.	.	.	.	.	.	.	.	.	.	.	.	.	.	.	.	.	.
4877	1	PON2	NM_000305	chr7	94872109	94902320	-	.	.	.	.	.	.	.	.	.	.	.	.	.	.	.	.	.	.	.	.
6727	0,5	PDK4	NM_002612	chr7	95050744	95063861	-	.	.	.	.	.	.	.	.	.	.	.	.	.	.	.	.	.	.	.	.
2115	1,5	DYNC1I1	NM_001135556	chr7	95239753	95565672	+	.	.	.	.	.	.	.	.	.	.	.	.	.	.	.	.	.	.	.	.
6908	0,5	SLC25A13	NR_027662	chr7	95587467	95789395	-	.	.	.	.	.	.	.	.	.	.	.	.	.	.	.	.	.	.	.	.
5233	1	SHFM1	NM_006304	chr7	96156014	96177139	-	.	.	.	.	.	.	.	.	.	.	.	.	.	.	.	.	.	.	.	.
7280	0,25	DLX5	NM_005221	chr7	96487637	96492079	-	.	.	.	.	.	.	.	.	.	.	.	.	.	.	.	.	.	.	.	.
1790	2	TAC1	NM_003182	chr7	97199206	97207720	+	.	.	.	.	.	.	.	.	.	.	.	.	.	.	.	.	.	.	.	.
1258	2	ASNS	NM_183356	chr7	97319364	97339790	-	.	.	.	.	.	.	.	.	.	.	.	.	.	.	.	.	.	.	.	.
6612	0,5	MGC72080	NR_002822	chr7	97433843	97439574	-	.	.	.	.	.	.	.	.	.	.	.	.	.	.	.	.	.	.	.	.
1095	2,25	LMTK2	NM_014916	chr7	97574132	97676880	+	.	.	.	.	.	.	.	.	.	.	.	.	.	.	.	.	.	.	.	.
3145	1	BHLHA15	NM_177455	chr7	97679501	97680207	+	.	.	.	.	.	.	.	.	.	.	.	.	.	.	.	.	.	.	.	.
5453	1	TECPR1	NM_015395	chr7	97683982	97719404	-	.	.	.	.	.	.	.	.	.	.	.	.	.	.	.	.	.	.	.	.
3162	1	BRI3	NM_001159491	chr7	97748914	97760211	+	.	.	.	.	.	.	.	.	.	.	.	.	.	.	.	.	.	.	.	.
3126	1	BAIAP2L1	NM_018842	chr7	97758897	97868363	-	.	.	.	.	AY516263	chr7	97821517	-	.	.	.	.	.	.	.	.	.	.	.	.
2285	1,5	NPTX2	NM_002523	chr7	98084532	98097117	+	.	.	.	.	.	.	.	.	.	.	.	.	.	.	.	.	.	.	.	.
7030	0,5	TMEM130	NM_001134451	chr7	98282046	98305609	-	.	.	.	.	.	.	.	.	.	.	.	.	.	.	.	.	.	.	.	.
94	6	TRRAP	NM_001244580	chr7	98314048	98448802	+	.	.	.	.	.	.	.	.	.	.	.	.	.	.	.	.	.	.	.	.
1767	2	SMURF1	NM_020429	chr7	98462993	98579679	-	.	.	.	.	.	.	.	.	.	.	.	.	.	.	.	.	.	.	.	.
3045	1	ARPC1A	NM_006409	chr7	98761431	98801821	+	.	.	.	.	.	.	.	.	.	.	.	.	.	.	.	.	.	.	.	.
2005	1,5	ARPC1B	NM_005720	chr7	98810233	98830340	+	.	.	.	.	.	.	.	.	.	.	.	.	.	.	.	.	.	.	.	.
4727	1	PDAP1	NM_014891	chr7	98830234	98844241	-	.	.	.	.	.	.	.	.	.	.	.	.	.	.	.	.	.	.	.	.
3165	1	BUD31	NM_003910	chr7	98844536	98855175	+	.	.	.	.	.	.	.	.	.	.	.	.	.	.	.	.	.	.	.	.
4978	1	PTCD1	NM_015545	chr7	98852297	98874398	-	.	.	.	.	.	.	.	.	.	.	.	.	.	.	.	.	.	.	.	.
3452	1	CPSF4	NM_006693	chr7	98874498	98892932	+	.	.	.	.	.	.	.	.	.	.	.	.	.	.	.	.	.	.	.	.
1265	2	ATP5J2	NM_001003714	chr7	98893719	98901760	-	.	.	.	.	.	.	.	.	.	.	.	.	.	.	.	.	.	.	.	.
5794	1	ZNF394	NM_032164	chr7	98928789	98935813	-	.	.	.	.	.	.	.	.	.	.	.	.	.	.	.	.	.	.	.	.
5752	1	ZKSCAN5	NM_014569	chr7	98940508	98969381	+	.	.	.	.	.	.	.	.	.	.	.	.	.	.	.	.	.	.	.	.
5842	1	ZNF655	NM_138494	chr7	98993980	99012013	+	.	.	.	.	.	.	.	.	.	.	.	.	.	.	.	.	.	.	.	.

Table S2

tumor associated genes								HIV				MLV				MMTV				MMTV(SIN)				MMTV(SIN)arrest			
rank	score	gene symbol	RefSEq	chrom	txStart	txEnd	strand	integrant	chr	position	strand	integrant	chr	position	strand	integrant	chr	position	strand	integrant	chr	position	strand	integrant	chr	position	strand
5811	1	ZNF498	NM_145115	chr7	99052506	99067966	+	.	.	.	.	.	.	.	.	.	.	.	.	.	.	.	.	.	.	.	.
3513	1	CYP3A5	NR_033807	chr7	99083748	99115557	-	.	.	.	.	.	.	.	.	.	.	.	.	.	.	.	.	.	.	.	.
3514	1	CYP3A7	NM_000765	chr7	99140595	99170755	-	.	.	.	.	.	.	.	.	.	.	.	.	.	.	.	.	.	.	.	.
555	3	CYP3A4	NM_017460	chr7	99192518	99219747	-	.	.	.	.	.	.	.	.	.	.	.	.	.	.	.	.	.	.	.	.
3512	1	CYP3A43	NM_057096	chr7	99263571	99302109	+	.	.	.	.	.	.	.	.	.	.	.	.	.	.	.	.	.	.	.	.
5556	1	TRIM4	NM_033017	chr7	99325965	99355159	-	.	.	.	.	.	.	.	.	.	.	.	.	.	.	.	.	.	.	.	.
6046	0,5	AZGP1	NM_001185	chr7	99402285	99411671	-	.	.	.	.	.	.	.	.	.	.	.	.	.	.	.	.	.	.	.	.
5870	1	ZSCAN21	NM_145914	chr7	99485352	99500600	+	.	.	.	.	.	.	.	.	.	.	.	.	.	.	.	.	.	.	.	.
2087	1,5	COPS6	NM_006833	chr7	99524518	99527758	+	.	.	.	.	.	.	.	.	.	.	.	.	.	.	.	.	.	.	.	.
2669	1,25	MCM7	NM_182776	chr7	99528339	99536316	-	.	.	.	.	.	.	.	.	.	.	.	.	.	.	.	.	.	.	.	.
2997	1	AP4M1	NM_004722	chr7	99537065	99542739	+	.	.	.	.	.	.	.	.	.	.	.	.	.	.	.	.	.	.	.	.
5427	1	TAF6	NR_033792	chr7	99542628	99554931	-	.	.	.	.	.	.	.	.	.	.	.	.	.	.	.	.	.	.	.	.
3409	1	CNPY4	NM_152755	chr7	99555200	99561064	+	.	.	.	.	.	.	.	.	.	.	.	.	.	.	.	.	.	.	.	.
4366	1	MBLAC1	NM_203397	chr7	99562255	99564057	+	.	.	.	.	.	.	.	.	.	.	.	.	.	.	.	.	.	.	.	.
3247	1	C7orf59	NM_001008395	chr7	99584465	99589769	+	.	.	.	.	.	.	.	.	.	.	.	.	.	.	.	.	.	.	.	.
3244	1	C7orf43	NM_018275	chr7	99589978	99594238	-	.	.	.	.	.	.	.	.	.	.	.	.	.	.	.	.	.	.	.	.
3837	1	GAL3ST4	NM_024637	chr7	99594800	99604309	-	.	.	.	.	.	.	.	.	.	.	.	.	.	.	.	.	.	.	.	.
687	3	STAG3	NM_012447	chr7	99613473	99649946	+	.	.	.	.	.	.	.	.	.	.	.	.	.	.	.	.	.	.	.	.
5008	1	PVRIG	NM_024070	chr7	99654806	99657047	+	.	.	.	.	.	.	.	.	.	.	.	.	.	.	.	.	.	.	.	.
5329	1	SPDYE3	NM_001004351	chr7	99743260	99757755	+	.	.	.	.	.	.	.	.	.	.	.	.	.	.	.	.	.	.	.	.
4811	1	PILRB	NR_036569	chr7	99771623	99803390	+	AY517075.1	chr7	99800774	+	.	.	.	.	.	.	.	.	.	.	.	.	.	.	.	.
4810	1	PILRA	NM_013439	chr7	99809003	99835658	+	.	.	.	.	.	.	.	.	.	.	.	.	.	.	.	.	.	.	.	.
5736	1	ZCWPW1	NM_017984	chr7	99836430	99864367	-	.	.	.	.	.	.	.	.	.	.	.	.	.	.	.	.	.	.	.	.
4380	1	MEPCE	NM_001194992	chr7	99864348	99869685	+	.	.	.	.	.	.	.	.	.	.	.	.	.	.	.	.	.	.	.	.
1825	2	TSC22D4	NM_030935	chr7	99902077	99914838	-	.	.	.	.	.	.	.	.	.	.	.	.	.	.	.	.	.	.	.	.
2921	1	AGFG2	NM_006076	chr7	99974769	100003779	+	.	.	.	.	.	.	.	.	.	.	.	.	.	.	.	.	.	.	.	.
4301	1	LRCH4	NM_002319	chr7	100009569	100021712	-	.	.	.	.	.	.	.	.	.	.	.	.	.	.	.	.	.	.	.	.
1397	2	FBXO24	NM_012172	chr7	100025131	100036676	+	.	.	.	.	.	.	.	.	.	.	.	.	.	.	.	.	.	.	.	.
2705	1,25	PCOLCE	NM_002593	chr7	100037817	100043734	+	.	.	.	.	.	.	.	.	.	.	.	.	.	.	.	.	.	.	.	.
4426	1	MOSPD3	NM_023948	chr7	100048049	100050936	+	.	.	.	.	.	.	.	.	.	.	.	.	.	.	.	.	.	.	.	.
5463	1	TFR2	NM_003227	chr7	100055974	100077109	-	.	.	.	.	.	.	.	.	.	.	.	.	.	.	.	.	.	.	.	.
1426	2	GNB2	NM_005273	chr7	100109298	100114728	+	.	.	.	.	.	.	.	.	.	.	.	.	.	.	.	.	.	.	.	.
3889	1	GIGYF1	NM_022574	chr7	100115065	100124806	-	.	.	.	.	.	.	.	.	.	.	.	.	.	.	.	.	.	.	.	.
4879	1	POP7	NM_005837	chr7	100141611	100143059	+	.	.	.	.	.	.	.	.	.	.	.	.	.	.	.	.	.	.	.	.
136	5	EPO	NM_000799	chr7	100156358	100159259	+	.	.	.	.	.	.	.	.	.	.	.	.	.	.	.	.	.	.	.	.
175	4,5	EPHB4	NM_004444	chr7	100238122	100263079	-	.	.	.	.	.	.	.	.	.	.	.	.	.	.	.	.	.	.	.	.
1823	2	TRIP6	NM_003302	chr7	100302885	100309012	+	.	.	.	.	.	.	.	.	.	.	.	.	.	.	.	.	.	.	.	.
2879	1	ACHE	NM_015831	chr7	100325550	100331477	-	.	.	.	.	.	.	.	.	.	.	.	.	.	.	.	.	.	.	.	.
952	2,5	SERPINE1	NM_000602	chr7	100557089	100569267	+	AY517095.1	chr7	100560971	-	.	.	.	.	.	.	.	.	.	.	.	.	.	.	.	.
2326	1,5	PLOD3	NM_001084	chr7	100635977	100647731	-	.	.	.	.	.	.	.	.	.	.	.	.	.	.	.	.	.	.	.	.
1333	2	CUX1	NM_001202545	chr7	101245903	101713970	+	CL800200	chr7	101556973	+	.	.	.	.	.	.	.	.	.	.	.	.	.	.	.	.
6884	0,5	SH2B2	NM_020979	chr7	101715124	101748898	+	.	.	.	.	.	.	.	.	.	.	.	.	.	.	.	.	.	.	.	.
1655	2	POLR2J	NM_006234	chr7	101900552	101906386	-	.	.	.	.	.	.	.	.	.	.	.	.	.	.	.	.	.	.	.	.
6575	0,5	LRRC17	NM_001031692	chr7	102340579	102372792	+	.	.	.	.	.	.	.	.	.	.	.	.	.	.	.	.	.	.	.	.
2370	1,5	RELN	NM_173054	chr7	102899466	103417199	-	.	.	.	.	.	.	.	.	.	.	.	.	.	.	.	.	.	.	.	.
4411	1	MLL5	NM_018682	chr7	104441872	104541768	+	AY517168.1	chr7	104500911	-	.	.	.	.	.	.	.	.	.	.	.	.	.	.	.	.
4411	1	MLL5	NM_018682	chr7	104441872	104541768	+	CL800536	chr7	104466761	+	.	.	.	.	.	.	.	.	.	.	.	.	.	.	.	.
2787	1,25	SRPK2	NM_182691	chr7	104544058	104696713	-	BH609584	chr7	104758823	-	.	.	.	.	.	.	.	.	.	.	.	.	.	.	.	.
6821	0,5	RINT1	NM_021930	chr7	104959767	104995360	+	.	.	.	.	.	.	.	.	.	.	.	.	.	.	.	.	.	.	.	.
6999	0,5	SYPL1	NM_006754	chr7	105518049	105540329	-	.	.	.	.	.	.	.	.	.	.	.	.	.	.	.	.	.	.	.	.
1943	1,75	NAMPT	NM_005746	chr7	105675967	105712874	-	.	.	.	.	.	.	.	.	.	.	.	.	.	.	.	.	.	.	.	.

Table S2

tumor associated genes								HIV				MLV				MMTV				MMTV(SIN)				MMTV(SIN)arrest			
rank	score	gene symbol	RefSEq	chrom	txStart	txEnd	strand	integrant	chr	position	strand	integrant	chr	position	strand	integrant	chr	position	strand	integrant	chr	position	strand	integrant	chr	position	strand
17	9,75	PIK3CG	NM_002649	chr7	106293159	106334828	+	.	.	.	.	.	.	.	.	.	.	.	.	.	.	.	.	.	.	.	.
1956	1,75	PRKAR2B	NM_002736	chr7	106472413	106589492	+	.	.	.	.	.	.	.	.	.	.	.	.	.	.	.	.	.	.	.	.
6396	0,5	GPR22	NM_005295	chr7	106897737	106903361	+	.	.	.	.	.	.	.	.	.	.	.	.	.	.	.	.	.	.	.	.
6912	0,5	SLC26A4	NM_000441	chr7	107088315	107145488	+	.	.	.	.	.	.	.	.	.	.	.	.	.	.	.	.	.	.	.	.
3587	1	DLD	NM_000108	chr7	107318821	107348879	+	AY516986.1	chr7	107346916	+	.	.	.	.	.	.	.	.	.	.	.	.	.	.	.	.
4254	1	LAMB1	NM_002291	chr7	107351481	107431040	-	.	.	.	.	.	.	.	.	.	.	.	.	.	.	.	.	.	.	.	.
4255	1	LAMB4	NM_007356	chr7	107451231	107558037	-	.	.	.	.	.	.	.	.	.	.	.	.	.	.	.	.	.	.	.	.
4618	1	NRCAM	NM_005010	chr7	107575306	107884077	-	.	.	.	.	.	.	.	.	.	.	.	.	.	.	.	.	3336_390_1	chr7	107864478	-
4310	1	LRRN3	NM_001099658	chr7	110518297	110552745	+	.	.	.	.	.	.	.	.	.	.	.	.	.	.	.	.	.	.	.	.
3601	1	DOCK4	NM_014705	chr7	111153399	111633698	-	.	.	.	.	.	.	.	.	.	.	.	.	.	.	.	.	.	.	.	.
4120	1	IFRD1	NM_001007245	chr7	111850434	111904494	+	BH609588	chr7	111892824	+	.	.	.	.	.	.	.	.	.	.	.	.	.	.	.	.
225	4,25	PPP1R3A	NM_002711	chr7	113304117	113346318	-	.	.	.	.	.	.	.	.	.	.	.	.	.	.	.	.	.	.	.	.
760	2,75	TES	NM_015641	chr7	115637782	115686073	+	.	.	.	.	.	.	.	.	.	.	.	.	.	.	.	.	.	.	.	.
2053	1,5	CAV2	NM_198212	chr7	115926890	115935831	+	.	.	.	.	.	.	.	.	.	.	.	.	.	.	.	.	.	.	.	.
172	4,5	CAV1	NM_001753	chr7	115952074	115988475	+	.	.	.	.	.	.	.	.	.	.	.	.	.	.	.	.	.	.	.	.
8	11,75	MET	NM_001127500	chr7	116099694	116225676	+	.	.	.	.	.	.	.	.	.	.	.	.	.	.	.	.	.	.	.	.
7224	0,25	CAPZA2	NM_006136	chr7	116289798	116346549	+	.	.	.	.	.	.	.	.	.	.	.	.	.	.	.	.	.	.	.	.
6977	0,5	ST7	NM_021908	chr7	116380616	116651197	+	.	.	.	.	.	.	.	.	.	.	.	.	.	.	.	.	.	.	.	.
5976	0,75	WNT2	NM_003391	chr7	116703921	116750579	-	.	.	.	.	.	.	.	.	.	.	.	.	.	.	.	.	.	.	.	.
7177	0,25	ASZ1	NM_130768	chr7	116790511	116854813	-	.	.	.	.	AY516201	chr7	116813614	-	.	.	.	.	.	.	.	.	.	.	.	.
7243	0,25	CFTR	NM_000492	chr7	116907252	117095954	+	.	.	.	.	.	.	.	.	.	.	.	.	.	.	.	.	.	.	.	.
5930	0,75	KCND2	NM_012281	chr7	119700957	120177623	+	.	.	.	.	.	.	.	.	.	.	.	.	.	.	.	.	.	.	.	.
5971	0,75	TSPAN12	NM_012338	chr7	120214609	120285413	-	.	.	.	.	.	.	.	.	.	.	.	.	.	.	.	.	.	.	.	.
5924	0,75	ING3	NM_198267	chr7	120378052	120384270	+	.	.	.	.	.	.	.	.	.	.	.	.	.	.	.	.	.	.	.	.
2830	1,25	WNT16	NM_016087	chr7	120752656	120768394	+	.	.	.	.	.	.	.	.	.	.	.	.	.	.	.	.	.	.	.	.
7307	0,25	FAM3C	NM_001040020	chr7	120776140	120823658	-	.	.	.	.	.	.	.	.	.	.	.	.	.	.	.	.	.	.	.	.
2352	1,5	PTPRZ1	NM_001206839	chr7	121300394	121489326	+	.	.	.	.	.	.	.	.	.	.	.	.	.	.	.	.	.	.	.	.
2848	1	AASS	NM_005763	chr7	121500833	121571580	-	.	.	.	.	.	.	.	.	.	.	.	.	.	.	.	.	.	.	.	.
6118	0,5	CADPS2	NM_001167940	chr7	121745713	122314049	-	.	.	.	.	.	.	.	.	.	.	.	.	.	.	.	.	.	.	.	.
4528	1	NDUFA5	NM_005000	chr7	122968318	122985194	-	.	.	.	.	.	.	.	.	.	.	.	.	.	.	.	.	.	.	.	.
5686	1	WASL	NM_003941	chr7	123109232	123176352	-	CL800075	chr7	123121571	+	.	.	.	.	.	.	.	.	.	.	.	.	.	.	.	.
5323	1	SPAM1	NM_003117	chr7	123352521	123398697	+	.	.	.	.	.	.	.	.	.	.	.	.	.	.	.	.	.	.	.	.
6398	0,5	GPR37	NM_005302	chr7	124173349	124192917	-	.	.	.	.	.	.	.	.	.	.	.	.	.	.	.	.	.	.	.	.
4881	1	POT1	NR_003102	chr7	124249675	124357273	-	CL800346	chr7	124273642	+	.	.	.	.	.	.	.	.	.	.	.	.	.	.	.	.
3018	1	ARF5	NM_001662	chr7	127015641	127018995	+	.	.	.	.	.	.	.	.	.	.	.	.	.	.	.	.	.	.	.	.
6577	0,5	LRRC4	NM_022143	chr7	127454359	127458238	-	.	.	.	.	.	.	.	.	.	.	.	.	.	.	.	.	.	.	.	.
1931	1,75	LEP	NM_000230	chr7	127668566	127684918	+	.	.	.	.	.	.	.	.	.	.	.	.	.	.	.	.	.	.	.	.
1471	2	IMPDH1	NM_183243	chr7	127819566	127837272	-	.	.	.	.	.	.	.	.	.	.	.	.	.	.	.	.	.	.	.	.
4383	1	METTL2B	NM_018396	chr7	127904018	127930214	+	.	.	.	.	.	.	.	.	.	.	.	.	.	.	.	.	.	.	.	.
179	4,5	FLNC	NM_001127487	chr7	128257718	128286564	+	.	.	.	.	.	.	.	.	.	.	.	.	.	.	.	.	.	.	.	.
3093	1	ATP6V1F	NM_004231	chr7	128290092	128293139	+	.	.	.	.	.	.	.	.	.	.	.	.	.	.	.	.	.	.	.	.
5518	1	TNPO3	NR_034053	chr7	128381469	128482463	-	.	.	.	.	.	.	.	.	.	.	.	.	.	.	.	.	3208_314_1	chr7	128418497	+
345	3,75	SMO	NM_005631	chr7	128615948	128640621	+	.	.	.	.	.	.	.	.	.	.	.	.	.	.	.	.	.	.	.	.
2494	1,25	AHCYL2	NM_001130723	chr7	128795199	128857288	+	.	.	.	.	.	.	.	.	.	.	.	.	.	.	.	.	.	.	.	.
5603	1	UBE2H	NM_001202498	chr7	129257808	129380019	-	.	.	.	.	.	.	.	.	.	.	.	.	.	.	.	.	.	.	.	.
7253	0,25	CPA4	NM_001163446	chr7	129720209	129751256	+	.	.	.	.	.	.	.	.	.	.	.	.	.	.	.	.	.	.	.	.
6608	0,5	MEST	NM_177525	chr7	129918405	129933374	+	.	.	.	.	.	.	.	.	.	.	.	.	.	.	.	.	.	.	.	.
3705	1	EXOC4	NM_021807	chr7	132588362	133401053	+	BH609591	chr7	132889453	+	.	.	.	.	.	.	.	.	.	.	.	.	.	.	.	.
3705	1	EXOC4	NM_021807	chr7	132588362	133401053	+	CL800387	chr7	133390981	+	.	.	.	.	.	.	.	.	.	.	.	.	.	.	.	.
1509	2	LRGUK	NM_144648	chr7	133462644	133599473	+	.	.	.	.	.	.	.	.	_334_1_206	chr7	133510570	+	.	.	.	.	.	.	.	.
2496	1,25	AKR1B1	NM_001628	chr7	133777646	133794428	-	.	.	.	.	.	.	.	.	.	.	.	.	.	.	.	.	.	.	.	.

Table S2

tumor associated genes								HIV				MLV				MMTV				MMTV(SIN)				MMTV(SIN)arrest			
rank	score	gene symbol	RefSEq	chrom	txStart	txEnd	strand	integrant	chr	position	strand	integrant	chr	position	strand	integrant	chr	position	strand	integrant	chr	position	strand	integrant	chr	position	strand
2497	1,25	AKR1B10	NM_020299	chr7	133862883	133876706	+	.	.	.	.	.	.	.	.	.	.	.	.	.	.	.	.	.	.	.	.
3156	1	BPGM	NM_001724	chr7	133982070	134015107	+	.	.	.	.	.	.	.	.	.	.	.	.	.	.	.	.	.	.	.	.
6123	0,5	CALD1	NM_033157	chr7	134114703	134306020	+	.	.	.	.	AY516356	chr7	134222082	-	.	.	.	.	.	.	.	.	1007_127_1	chr7	134249425	+
5698	1	WDR91	NM_014149	chr7	134519129	134546856	-	.	.	.	.	.	.	.	.	.	.	.	.	.	.	.	.	.	.	.	.
3408	1	CNOT4	NM_001190850	chr7	134697086	134845415	-	BH609577	chr7	134779001	-	.	.	.	.	.	.	.	.	.	.	.	.	.	.	.	.
4650	1	NUP205	NM_015135	chr7	134893201	134984039	+	.	.	.	.	.	.	.	.	.	.	.	.	.	.	.	.	.	.	.	.
4988	1	PTN	NM_002825	chr7	136562634	136679086	-	.	.	.	.	.	.	.	.	.	.	.	.	.	.	.	.	.	.	.	.
249	4	DGKI	NM_004717	chr7	136724924	137182149	-	AY517423.1	chr7	137014141	+	.	.	.	.	.	.	.	.	.	.	.	.	2525_219_1	chr7	137109365	+
3456	1	CREB3L2	NM_194071	chr7	137210264	137337387	-	.	.	.	.	.	.	.	.	.	.	.	.	.	.	.	.	.	.	.	.
1235	2	AKR1D1	NM_001190906	chr7	137411717	137453590	+	.	.	.	.	.	.	.	.	.	.	.	.	.	.	.	.	.	.	.	.
764	2,75	TRIM24	NM_003852	chr7	137795618	137920872	+	.	.	.	.	.	.	.	.	.	.	.	.	.	.	.	.	.	.	.	.
1267	2	ATP6V0A4	NM_130840	chr7	138041578	138133481	-	.	.	.	.	.	.	.	.	.	.	.	.	.	.	.	.	.	.	.	.
4226	1	KIAA1549	NM_020910	chr7	138166666	138316604	-	CL800814	chr7	138286277	+	.	.	.	.	.	.	.	.	.	.	.	.	.	.	.	.
7115	0,5	ZC3HAV1	NM_020119	chr7	138378805	138445005	-	.	.	.	.	.	.	.	.	.	.	.	.	.	.	.	.	.	.	.	.
6188	0,5	CLEC2L	NM_001080511	chr7	138859213	138880271	+	.	.	.	.	.	.	.	.	.	.	.	.	.	.	.	.	.	.	.	.
263	4	HIPK2	NM_001113239	chr7	138896855	139124162	-	.	.	.	.	.	.	.	.	.	.	.	.	.	.	.	.	.	.	.	.
4187	1	JHDM1D	NM_030647	chr7	139431014	139523210	-	.	.	.	.	.	.	.	.	.	.	.	.	.	.	.	.	.	.	.	.
5274	1	SLC37A3	NM_032295	chr7	139680020	139744780	-	.	.	.	.	.	.	.	.	.	.	.	.	.	.	.	.	.	.	.	.
4407	1	MKRN1	NM_001145125	chr7	139802451	139825838	-	.	.	.	.	.	.	.	.	.	.	.	.	.	.	.	.	.	.	.	.
2104	1,5	DENND2A	NM_015689	chr7	139864688	139948811	-	.	.	.	.	.	.	.	.	.	.	.	.	.	.	.	.	.	.	.	.
1214	2	ADCK2	NM_052853	chr7	140019421	140041377	+	.	.	.	.	.	.	.	.	.	.	.	.	.	.	.	.	.	.	.	.
1568	2	NDUFB2	NM_004546	chr7	140042949	140052915	+	AY517454.1	chr7	140051337	-	.	.	.	.	.	.	.	.	.	.	.	.	.	.	.	.
22	9	BRAF	NM_004333	chr7	140080281	140271033	-	CL529704	chr7	140188725	-	.	.	.	.	.	.	.	.	.	.	.	.	.	.	.	.
4441	1	MRPS33	NM_016071	chr7	140352429	140360948	-	.	.	.	.	.	.	.	.	.	.	.	.	.	.	.	.	.	.	.	.
2922	1	AGK	NM_018238	chr7	140897546	141000678	+	.	.	.	.	.	.	.	.	.	.	.	.	.	.	.	.	.	.	.	.
5699	1	WEE2	NM_001105558	chr7	141054621	141077540	+	.	.	.	.	.	.	.	.	.	.	.	.	.	.	.	.	.	.	.	.
4390	1	MGAM	NM_004668	chr7	141342147	141453016	+	.	.	.	.	.	.	.	.	.	.	.	.	.	.	.	.	.	.	.	.
6775	0,5	PRSS1	NM_002769	chr7	142136892	142140501	+	.	.	.	.	.	.	.	.	.	.	.	.	.	.	.	.	.	.	.	.
105	5,5	EPHB6	NM_004445	chr7	142262913	142278969	+	.	.	.	.	.	.	.	.	.	.	.	.	.	.	.	.	.	.	.	.
5567	1	TRPV6	NM_018646	chr7	142279081	142293612	-	.	.	.	.	.	.	.	.	.	.	.	.	.	.	.	.	.	.	.	.
5566	1	TRPV5	NM_019841	chr7	142315769	142340942	-	.	.	.	.	.	.	.	.	.	.	.	.	.	.	.	.	.	.	.	.
2177	1,5	GSTK1	NM_001143679	chr7	142670643	142676344	+	.	.	.	.	.	.	.	.	.	.	.	.	.	.	.	.	.	.	.	.
1885	1,75	CASP2	NM_032982	chr7	142695429	142714911	+	CL529406	chr7	142698566	-	.	.	.	.	.	.	.	.	.	.	.	.	.	.	.	.
7145	0,5	ZYX	NM_001010972	chr7	142788481	142798327	+	.	.	.	.	.	.	.	.	.	.	.	.	.	.	.	.	.	.	.	.
825	2,5	EPHA1	NM_005232	chr7	142798326	142816107	-	.	.	.	.	.	.	.	.	.	.	.	.	.	.	.	.	.	.	.	.
3030	1	ARHGEF5	NM_005435	chr7	143683421	143708658	+	.	.	.	.	.	.	.	.	.	.	.	.	.	.	.	.	.	.	.	.
5532	1	TPK1	NM_001042482	chr7	143779966	144164079	-	.	.	.	.	.	.	.	.	.	.	.	.	.	.	.	.	.	.	.	.
2084	1,5	CNTNAP2	NM_014141	chr7	145444385	147749021	+	CL800294	chr7	146073258	+	AY516714	chr7	145461772	-	.	.	.	.	.	.	.	.	1051_128_1	chr7	147427286	-
1043	2,25	EZH2	NM_001203249	chr7	148135396	148211534	-	BH609593	chr7	148166889	-	.	.	.	.	.	.	.	.	.	.	.	.	.	.	.	.
2314	1,5	PDIA4	NM_004911	chr7	148331086	148356715	-	.	.	.	.	.	.	.	.	.	.	.	.	.	.	.	.	.	.	.	.
5858	1	ZNF786	NM_152411	chr7	148397665	148418802	-	.	.	.	.	.	.	.	.	.	.	.	.	.	.	.	.	.	.	.	.
5800	1	ZNF425	NM_001001661	chr7	148430810	148454371	-	.	.	.	.	.	.	.	.	.	.	.	.	.	.	.	.	.	.	.	.
5806	1	ZNF467	NM_207336	chr7	149092384	149101228	-	.	.	.	.	.	.	.	.	.	.	.	.	.	.	.	.	.	.	.	.
6795	0,5	RARRES2	NM_002889	chr7	149666339	149669696	-	.	.	.	.	.	.	.	.	.	.	.	.	.	.	.	.	.	.	.	.
7033	0,5	TMEM176A	NM_018487	chr7	150128786	150133141	+	.	.	.	.	.	.	.	.	.	.	.	.	.	.	.	.	.	.	.	.
1199	2	ABP1	NM_001091	chr7	150180505	150189312	+	.	.	.	.	.	.	.	.	.	.	.	.	.	.	.	.	.	.	.	.
82	6,25	NOS3	NM_000603	chr7	150319076	150342620	+	.	.	.	.	.	.	.	.	.	.	.	.	.	.	.	.	.	.	.	.
1197	2	ABCB8	NM_007188	chr7	150356442	150375802	+	.	.	.	.	.	.	.	.	.	.	.	.	.	.	.	.	.	.	.	.
2065	1,5	CDK5	NM_004935	chr7	150381831	150385985	-	.	.	.	.	.	.	.	.	.	.	.	.	.	.	.	.	.	.	.	.
2134	1,5	FASTK	NM_006712	chr7	150404640	150408903	-	.	.	.	.	.	.	.	.	.	.	.	.	.	.	.	.	.	.	.	.
298	4	RHEB	NM_005614	chr7	150794030	150847943	-	.	.	.	.	.	.	.	.	.	.	.	.	.	.	.	.	.	.	.	.

Table S2

tumor associated genes								HIV				MLV				MMTV				MMTV(SIN)				MMTV(SIN)arrest			
rank	score	gene symbol	RefSEq	chrom	txStart	txEnd	strand	integrant	chr	position	strand	integrant	chr	position	strand	integrant	chr	position	strand	integrant	chr	position	strand	integrant	chr	position	strand
4941	1	PRKAG2	NM_001040633	chr7	150884133	151142890	-	.	.	.	.	.	.	.	.	.	.	.	.	.	.	.	.	1973_176_1	chr7	151133480	+
1419	2	GALNTL5	NR_033169	chr7	151284396	151347952	+	.	.	.	.	.	.	.	.	.	.	.	.	.	.	.	.	.	.	.	.
279	4	MLL3	NM_170606	chr7	151462942	151764023	-	AY517031.1	chr7	151570316	-	.	.	.	.	.	.	.	.	.	.	.	.	.	.	.	.
1184	2,25	XRCC2	NM_005431	chr7	151974519	152004183	-	.	.	.	.	.	.	.	.	.	.	.	.	.	.	.	.	.	.	.	.
1206	2	ACTR3B	NR_073001	chr7	152087766	152183397	+	.	.	.	.	.	.	.	.	.	.	.	.	.	.	.	.	.	.	.	.
2571	1,25	DPP6	NM_130797	chr7	153380709	154316928	+	.	.	.	.	.	.	.	.	.	.	.	.	1536_119	chr7	153339901	+	1872_170_1	chr7	153327095	-
1597	2	PAXIP1	NM_007349	chr7	154366332	154425615	-	.	.	.	.	.	.	.	.	.	.	.	.	.	.	.	.	.	.	.	.
4100	1	HTR5A	NM_024012	chr7	154492966	154510035	+	.	.	.	.	.	.	.	.	.	.	.	.	.	.	.	.	.	.	.	.
1756	2	SHH	NM_000193	chr7	155288318	155297728	-	.	.	.	.	.	.	.	.	.	.	.	.	.	.	.	.	.	.	.	.
1541	2	MNX1	NM_005515	chr7	156490307	156496108	-	.	.	.	.	.	.	.	.	.	.	.	.	.	.	.	.	.	.	.	.
1833	2	UBE3C	NM_014671	chr7	156624415	156754827	+	.	.	.	.	.	.	.	.	.	.	.	.	.	.	.	.	.	.	.	.
2351	1,5	PTPRN2	NM_130842	chr7	157024510	158073243	-	.	.	.	.	.	.	.	.	.	.	.	.	s702_132	chr7	157633804	+	.	.	.	.
2351	1,5	PTPRN2	NM_130842	chr7	157024510	158073243	-	.	.	.	.	.	.	.	.	.	.	.	.	1402_182	chr7	157633256	+	.	.	.	.
2351	1,5	PTPRN2	NM_130842	chr7	157024510	158073243	-	.	.	.	.	.	.	.	.	.	.	.	.	1549_178	chr7	157634434	+	.	.	.	.
3756	1	FBXO25	NM_183421	chr8	346807	409875	+	CL800820	chr8	408565	+	.	.	.	.	.	.	.	.	.	.	.	.	.	.	.	.
6267	0,5	DLGAP2	NM_004745	chr8	1436975	1644049	+	.	.	.	.	.	.	.	.	.	.	.	.	.	.	.	.	.	.	.	.
3029	1	ARHGEF10	NM_014629	chr8	1759555	1894214	+	.	.	.	.	.	.	.	.	.	.	.	.	.	.	.	.	.	.	.	.
4197	1	KBTBD11	NM_014867	chr8	1909450	1942516	+	.	.	.	.	.	.	.	.	.	.	.	.	.	.	.	.	.	.	.	.
6644	0,5	MYOM2	NM_003970	chr8	1980564	2080787	+	.	.	.	.	.	.	.	.	.	.	.	.	.	.	.	.	.	.	.	.
993	2,25	ANGPT2	NM_001118887	chr8	6344580	6408192	-	.	.	.	.	.	.	.	.	.	.	.	.	.	.	.	.	.	.	.	.
7272	0,25	DEFB1	NM_005218	chr8	6715508	6722939	-	.	.	.	.	.	.	.	.	.	.	.	.	.	.	.	.	.	.	.	.
4916	1	PPP1R3B	NM_001201329	chr8	9031173	9046562	-	.	.	.	.	.	.	.	.	.	.	.	.	.	.	.	.	.	.	.	.
5152	1	RP1L1	NM_178857	chr8	10501269	10550027	-	.	.	.	.	.	.	.	.	.	.	.	.	.	.	.	.	.	.	.	.
5319	1	SOX7	NM_031439	chr8	10618687	10625432	-	.	.	.	.	.	.	.	.	.	.	.	.	.	.	.	.	.	.	.	.
4459	1	MTMR9	NM_015458	chr8	11179409	11223064	+	.	.	.	.	.	.	.	.	.	.	.	.	.	.	.	.	.	.	.	.
2514	1,25	BLK	NM_001715	chr8	11388929	11459517	+	.	.	.	.	.	.	.	.	.	.	.	.	.	.	.	.	.	.	.	.
1052	2,25	GATA4	NM_002052	chr8	11599125	11654918	+	.	.	.	.	.	.	.	.	.	.	.	.	.	.	.	.	.	.	.	.
4557	1	NEIL2	NM_001135746	chr8	11664580	11682263	+	.	.	.	.	.	.	.	.	.	.	.	.	.	.	.	.	.	.	.	.
3767	1	FDFT1	NM_004462	chr8	11697598	11734227	+	.	.	.	.	.	.	.	.	.	.	.	.	.	.	.	.	.	.	.	.
1020	2,25	CTSB	NM_147782	chr8	11737442	11763055	-	.	.	.	.	AY515899	chr8	11758011	+	.	.	.	.	.	.	.	.	.	.	.	.
720	2,75	DLC1	NM_182643	chr8	12985242	13416800	-	CL799730	chr8	13139124	-	.	.	.	.	.	.	.	.	.	.	.	.	.	.	.	.
696	3	TUSC3	NM_178234	chr8	15441966	15668529	+	.	.	.	.	.	.	.	.	.	.	.	.	.	.	.	.	.	.	.	.
5935	0,75	MSR1	NM_138716	chr8	16009757	16094671	-	.	.	.	.	.	.	.	.	.	.	.	.	.	.	.	.	.	.	.	.
3772	1	FGF20	NM_019851	chr8	16894704	16904045	-	.	.	.	.	.	.	.	.	.	.	.	.	.	.	.	.	.	.	.	.
3653	1	EFHA2	NM_181723	chr8	16929117	17024519	+	CL799692	chr8	17016584	+	.	.	.	.	.	.	.	.	.	.	.	.	.	.	.	.
6635	0,5	MTMR7	NM_004686	chr8	17198676	17315411	-	.	.	.	.	.	.	.	.	.	.	.	.	.	.	.	.	.	.	.	.
2707	1,25	PDGFRL	NM_006207	chr8	17478214	17544922	+	.	.	.	.	.	.	.	.	.	.	.	.	.	.	.	.	.	.	.	.
637	3	PCM1	NM_006197	chr8	17824645	17931737	+	CL800554	chr8	17895130	+	.	.	.	.	.	.	.	.	.	.	.	.	.	.	.	.
3051	1	ASAH1	NM_177924	chr8	17958204	17986159	-	.	.	.	.	.	.	.	.	.	.	.	.	.	.	.	.	.	.	.	.
2691	1,25	NAT1	NM_001160170	chr8	18111897	18125478	+	.	.	.	.	.	.	.	.	.	.	.	.	.	.	.	.	.	.	.	.
902	2,5	NAT2	NM_000015	chr8	18293034	18303003	+	.	.	.	.	.	.	.	.	.	.	.	.	.	.	.	.	.	.	.	.
1694	2	PSD3	NM_206909	chr8	18429092	18710685	-	.	.	.	.	.	.	.	.	.	.	.	.	.	.	.	.	.	.	.	.
3467	1	CSGALNACT1	NR_024040	chr8	19305951	19584541	-	CL800179	chr8	19570257	-	AY516807	chr8	19482820	+	.	.	.	.	.	.	.	.	.	.	.	.
3467	1	CSGALNACT1	NR_024040	chr8	19305951	19584541	-	CL800179	chr8	19570257	-	AY516331	chr8	19438078	-	.	.	.	.	.	.	.	.	.	.	.	.
1932	1,75	LPL	NM_000237	chr8	19840861	19869050	+	.	.	.	.	.	.	.	.	.	.	.	.	.	.	.	.	.	.	.	.
2013	1,5	ATP6V1B2	NM_001693	chr8	20098983	20123487	+	.	.	.	.	.	.	.	.	.	.	.	.	.	.	.	.	.	.	.	.
7408	0,25	LZTS1	NM_021020	chr8	20147955	20157083	-	.	.	.	.	.	.	.	.	.	.	.	.	.	.	.	.	.	.	.	.
6281	0,5	DOK2	NM_003974	chr8	21822329	21827151	-	.	.	.	.	.	.	.	.	.	.	.	.	.	.	.	.	.	.	.	.
4599	1	NPM2	NM_182795	chr8	21938299	21950354	+	CL529018	chr8	21946258	-	.	.	.	.	.	.	.	.	.	.	.	.	.	.	.	.
3771	1	FGF17	NM_003867	chr8	21956373	21962265	+	.	.	.	.	.	.	.	.	.	.	.	.	.	.	.	.	.	.	.	.
6313	0,5	EPB49	NM_001978	chr8	21972631	21995982	+	.	.	.	.	.	.	.	.	.	.	.	.	.	.	.	.	.	.	.	.

Table S2

tumor associated genes								HIV				MLV				MMTV				MMTV(SIN)				MMTV(SIN)arrest			
rank	score	gene symbol	RefSEq	chrom	txStart	txEnd	strand	integrant	chr	position	strand	integrant	chr	position	strand	integrant	chr	position	strand	integrant	chr	position	strand	integrant	chr	position	strand
4079	1	HR	NM_005144	chr8	22027876	22044510	-	.	.	.	.	.	.	.	.	.	.	.	.	.	.	.	.	.	.	.	.
6555	0,5	LGI3	NM_139278	chr8	22060287	22070289	-	.	.	.	.	.	.	.	.	.	.	.	.	.	.	.	.	.	.	.	.
6882	0,5	SFTPC	NM_003018	chr8	22075128	22077936	+	.	.	.	.	.	.	.	.	.	.	.	.	.	.	.	.	.	.	.	.
3152	1	BMP1	NM_001199	chr8	22078597	22114941	+	.	.	.	.	.	.	.	.	.	.	.	.	.	.	.	.	.	.	.	.
6742	0,5	PHYHIP	NM_001099335	chr8	22133160	22145796	-	.	.	.	.	.	.	.	.	.	.	.	.	.	.	.	.	.	.	.	.
4870	1	POLR3D	NM_001722	chr8	22158563	22164625	+	.	.	.	.	.	.	.	.	.	.	.	.	.	.	.	.	.	.	.	.
1674	2	PPP3CC	NM_005605	chr8	22354427	22454602	+	.	.	.	.	.	.	.	.	.	.	.	.	.	.	.	.	.	.	.	.
6301	0,5	EGR3	NM_001199881	chr8	22601118	22606038	-	.	.	.	.	.	.	.	.	.	.	.	.	.	.	.	.	.	.	.	.
4749	1	PEBP4	NM_144962	chr8	22626709	22841366	-	.	.	.	.	.	.	.	.	.	.	.	.	.	.	.	.	.	.	.	.
7513	0,25	RHOBTB2	NM_001160037	chr8	22909305	22933655	+	.	.	.	.	.	.	.	.	.	.	.	.	.	.	.	.	.	.	.	.
971	2,5	TNFRSF10B	NM_003842	chr8	22933592	22982645	-	.	.	.	.	.	.	.	.	.	.	.	.	.	.	.	.	.	.	.	.
2802	1,25	TNFRSF10C	NM_003841	chr8	23016271	23030895	+	.	.	.	.	.	.	.	.	.	.	.	.	.	.	.	.	.	.	.	.
972	2,5	TNFRSF10D	NM_003840	chr8	23049045	23077488	-	.	.	.	.	.	.	.	.	.	.	.	.	.	.	.	.	.	.	.	.
2445	1,5	TNFRSF10A	NM_003844	chr8	23104914	23138625	-	.	.	.	.	.	.	.	.	.	.	.	.	.	.	.	.	.	.	.	.
3688	1	ENTPD4	NM_004901	chr8	23342609	23371189	-	.	.	.	.	.	.	.	.	.	.	.	.	.	.	.	.	.	.	.	.
4581	1	NKX3-1	NM_001256339	chr8	23592150	23596347	-	.	.	.	.	.	.	.	.	.	.	.	.	.	.	.	.	.	.	.	.
1210	2	ADAM28	NM_014265	chr8	24207524	24268671	+	CL800419	chr8	24259720	-	.	.	.	.	.	.	.	.	.	.	.	.	.	.	.	.
5937	0,75	NEFM	NM_005382	chr8	24827178	24832511	+	.	.	.	.	.	.	.	.	.	.	.	.	.	.	.	.	.	.	.	.
2276	1,5	NEFL	NM_006158	chr8	24864385	24870048	-	.	.	.	.	.	.	.	.	.	.	.	.	.	.	.	.	.	.	.	.
3602	1	DOCK5	NM_024940	chr8	25098203	25326536	+	.	.	.	.	.	.	.	.	.	.	.	.	.	.	.	.	.	.	.	.
470	3,25	GNRH1	NM_001083111	chr8	25332690	25338473	-	.	.	.	.	.	.	.	.	.	.	.	.	.	.	.	.	.	.	.	.
6159	0,5	CDCA2	NM_152562	chr8	25372429	25421342	+	.	.	.	.	.	.	.	.	.	.	.	.	.	.	.	.	.	.	.	.
1670	2	PPP2R2A	NM_001177591	chr8	26206648	26286112	+	.	.	.	.	.	.	.	.	.	.	.	.	.	.	.	.	.	.	.	.
2517	1,25	BNIP3L	NM_004331	chr8	26296439	26326561	+	.	.	.	.	.	.	.	.	.	.	.	.	.	.	.	.	.	.	.	.
2424	1,5	STMN4	NM_030795	chr8	27149730	27171820	-	.	.	.	.	.	.	.	.	.	.	.	.	.	.	.	.	.	.	.	.
497	3,25	PTK2B	NM_173175	chr8	27238997	27372825	+	BH609598	chr8	27337174	+	.	.	.	.	.	.	.	.	.	.	.	.	.	.	.	.
1386	2	EPHX2	NM_001256484	chr8	27404601	27458356	+	.	.	.	.	.	.	.	.	.	.	.	.	.	.	.	.	.	.	.	.
1014	2,25	CLU	NR_038335	chr8	27510350	27524871	-	.	.	.	.	.	.	.	.	.	.	.	.	.	.	.	.	.	.	.	.
3698	1	ESCO2	NM_001017420	chr8	27687976	27718343	+	.	.	.	.	.	.	.	.	.	.	.	.	.	.	.	.	.	.	.	.
2306	1,5	PBK	NM_018492	chr8	27723056	27751268	-	.	.	.	.	.	.	.	.	.	.	.	.	.	.	.	.	.	.	.	.
5193	1	SCARA5	NM_173833	chr8	27783317	27906288	-	.	.	.	.	.	.	.	.	.	.	.	.	.	.	.	.	.	.	.	.
6759	0,5	PNOC	NM_006228	chr8	28230567	28256787	+	.	.	.	.	.	.	.	.	.	.	.	.	.	.	.	.	.	.	.	.
838	2,5	FZD3	NM_145866	chr8	28407640	28487704	+	.	.	.	.	.	.	.	.	.	.	.	.	.	.	.	.	.	.	.	.
2129	1,5	EXTL3	NM_001440	chr8	28615071	28667121	+	.	.	.	.	.	.	.	.	.	.	.	.	.	.	.	.	.	.	.	.
2573	1,25	DUSP4	NM_057158	chr8	29246497	29262241	-	.	.	.	.	.	.	.	.	.	.	.	.	.	.	.	.	.	.	.	.
3977	1	GSR	NM_001195104	chr8	30655119	30705028	-	CL799659	chr8	30665141	-	AY516046	chr8	30701041	+	.	.	.	.	.	.	.	.	.	.	.	.
292	4	PPP2CB	NM_001009552	chr8	30762667	30789894	-	.	.	.	.	.	.	.	.	.	.	.	.	.	.	.	.	.	.	.	.
6783	0,5	PURG	NM_013357	chr8	31007898	31009859	-	.	.	.	.	.	.	.	.	.	.	.	.	.	.	.	.	.	.	.	.
71	6,5	WRN	NM_000553	chr8	31010319	31150819	+	CL529459	chr8	31054870	+	.	.	.	.	.	.	.	.	.	.	.	.	.	.	.	.
370	3,5	DUSP26	NM_024025	chr8	33568392	33576981	-	.	.	.	.	.	.	.	.	.	.	.	.	.	.	.	.	.	.	.	.
6786	0,5	RAB11FIP1	NM_001002814	chr8	37835622	37876173	-	.	.	.	.	.	.	.	.	.	.	.	.	.	.	.	.	.	.	.	.
1040	2,25	EIF4EBP1	NM_004095	chr8	38007176	38037040	+	.	.	.	.	.	.	.	.	.	.	.	.	.	.	.	.	.	.	.	.
3056	1	ASH2L	NM_001105214	chr8	38082467	38116755	+	.	.	.	.	.	.	.	.	.	.	.	.	.	.	.	.	.	.	.	.
5372	1	STAR	NM_000349	chr8	38119374	38127757	-	.	.	.	.	.	.	.	.	.	.	.	.	.	.	.	.	.	.	.	.
4888	1	PPAPDC1B	NM_032483	chr8	38241414	38245895	-	.	.	.	.	.	.	.	.	.	.	.	.	.	.	.	.	.	.	.	.
1182	2,25	WHSC1L1	NM_017778	chr8	38293091	38358947	-	CL799678	chr8	38339425	+	.	.	.	.	.	.	.	.	.	.	.	.	.	.	.	.
58	6,75	FGFR1	NM_015850	chr8	38387812	38445509	-	CL529021	chr8	38402432	+	.	.	.	.	.	.	.	.	.	.	.	.	.	.	.	.
5423	1	TACC1	NM_001122824	chr8	38763878	38829703	+	CL799684	chr8	38818633	-	.	.	.	.	.	.	.	.	.	.	.	.	.	.	.	.
7152	0,25	ADAM9	NR_027639	chr8	38973661	39081936	+	.	.	.	.	.	.	.	.	.	.	.	.	.	.	.	.	.	.	.	.
4111	1	IDO1	NM_002164	chr8	39890484	39905466	+	.	.	.	.	.	.	.	.	.	.	.	.	.	.	.	.	.	.	.	.
4112	1	IDO2	NM_194294	chr8	39911630	39993067	+	.	.	.	.	.	.	.	.	.	.	.	.	s1741_281	chr8	39928141	+	.	.	.	.

Table S2

tumor associated genes								HIV				MLV				MMTV				MMTV(SIN)				MMTV(SIN)arrest			
rank	score	gene symbol	RefSEq	chrom	txStart	txEnd	strand	integrant	chr	position	strand	integrant	chr	position	strand	integrant	chr	position	strand	integrant	chr	position	strand	integrant	chr	position	strand
6109	0,5	C8orf4	NM_020130	chr8	40130143	40131984	+	.	.	.	.	.	.	.	.	.	.	.	.	.	.	.	.	.	.	.	.
7124	0,5	ZMAT4	NM_024645	chr8	40507267	40874500	-	CL800843	chr8	40797500	+	.	.	.	.	.	.	.	.	.	.	.	.	.	.	.	.
2395	1,5	SFRP1	NM_003012	chr8	41238632	41286147	-	.	.	.	.	.	.	.	.	.	.	.	.	.	.	.	.	.	.	.	.
2928	1	AGPAT6	NM_178819	chr8	41554863	41601677	+	.	.	.	.	.	.	.	.	.	.	.	.	.	.	.	.	.	.	.	.
2983	1	ANK1	NM_020477	chr8	41629900	41774297	-	.	.	.	.	.	.	.	.	.	.	.	.	.	.	.	.	.	.	.	.
494	3,25	PLAT	NM_000930	chr8	42151392	42184351	-	.	.	.	.	.	.	.	.	.	.	.	.	.	.	.	.	.	.	.	.
862	2,5	IKBKB	NM_001190720	chr8	42247976	42309328	+	.	.	.	.	.	.	.	.	.	.	.	.	.	.	.	.	3110_293_1	chr8	42277786	+
2716	1,25	POLB	NM_002690	chr8	42315129	42348488	+	.	.	.	.	.	.	.	.	.	.	.	.	.	.	.	.	.	.	.	.
3585	1	DKK4	NM_014420	chr8	42350742	42353831	-	.	.	.	.	.	.	.	.	.	.	.	.	.	.	.	.	.	.	.	.
3792	1	FNTA	NR_033698	chr8	43030598	43060088	+	CL529638	chr8	43044241	-	.	.	.	.	.	.	.	.	.	.	.	.	.	.	.	.
4038	1	HGSNAT	NM_152419	chr8	43114748	43177127	+	.	.	.	.	.	.	.	.	.	.	.	.	.	.	.	.	.	.	.	.
6166	0,5	CEBPD	NM_005195	chr8	48812028	48813279	-	.	.	.	.	.	.	.	.	.	.	.	.	.	.	.	.	.	.	.	.
110	5,5	PRKDC	NM_006904	chr8	48848221	49035296	-	.	.	.	.	.	.	.	.	.	.	.	.	.	.	.	.	.	.	.	.
5609	1	UBE2V2	NM_003350	chr8	49083547	49137007	+	.	.	.	.	.	.	.	.	.	.	.	.	.	.	.	.	.	.	.	.
6940	0,5	SNAI2	NM_003068	chr8	49992793	49996552	-	.	.	.	.	.	.	.	.	.	.	.	.	.	.	.	.	.	.	.	.
6704	0,5	OPRK1	NM_000912	chr8	54300828	54326747	-	.	.	.	.	.	.	.	.	.	.	.	.	.	.	.	.	.	.	.	.
2014	1,5	ATP6V1H	NM_213620	chr8	54790655	54918155	-	.	.	.	.	.	.	.	.	.	.	.	.	.	.	.	.	.	.	.	.
5115	1	RGS20	NM_170587	chr8	54926920	55034417	+	.	.	.	.	.	.	.	.	.	.	.	.	.	.	.	.	.	.	.	.
1796	2	TCEA1	NM_201437	chr8	55041668	55097561	-	.	.	.	.	.	.	.	.	.	.	.	.	.	.	.	.	.	.	.	.
4321	1	LYPLA1	NM_006330	chr8	55121490	55177130	-	CL799752	chr8	55130751	-	.	.	.	.	.	.	.	.	.	.	.	.	.	.	.	.
5967	0,75	SOX17	NM_022454	chr8	55533047	55536009	+	.	.	.	.	.	.	.	.	.	.	.	.	.	.	.	.	.	.	.	.
7106	0,5	XKR4	NM_052898	chr8	56177570	56601264	+	.	.	.	.	.	.	.	.	_286_1_186	chr8	56201717	-	.	.	.	.	.	.	.	.
887	2,5	LYN	NM_002350	chr8	56954939	57087560	+	.	.	.	.	.	.	.	.	.	.	.	.	.	.	.	.	.	.	.	.
7520	0,25	RPS20	NM_001146227	chr8	57143292	57149694	-	.	.	.	.	.	.	.	.	.	.	.	.	.	.	.	.	.	.	.	.
2262	1,5	MOS	NM_005372	chr8	57188054	57189095	-	.	.	.	.	.	.	.	.	.	.	.	.	.	.	.	.	.	.	.	.
747	2,75	PLAG1	NM_001114634	chr8	57236021	57286413	-	CL800115	chr8	57247618	-	.	.	.	.	.	.	.	.	.	.	.	.	.	.	.	.
6863	0,5	SDR16C5	NM_138969	chr8	57375123	57395795	-	.	.	.	.	.	.	.	.	.	.	.	.	.	.	.	.	.	.	.	.
7473	0,25	PENK	NM_006211	chr8	57516066	57521147	-	.	.	.	.	.	.	.	.	.	.	.	.	.	.	.	.	.	.	.	.
4145	1	IMPAD1	NM_017813	chr8	58033041	58068984	-	.	.	.	.	.	.	.	.	.	.	.	.	.	.	.	.	.	.	.	.
6327	0,5	FAM110B	NM_147189	chr8	59069666	59224831	+	.	.	.	.	.	.	.	.	.	.	.	.	.	.	.	.	.	.	.	.
3519	1	CYP7A1	NM_000780	chr8	59565290	59575274	-	.	.	.	.	.	.	.	.	.	.	.	.	.	.	.	.	.	.	.	.
5523	1	TOX	NM_014729	chr8	59880530	60194321	-	CL800713	chr8	59963436	+	.	.	.	.	.	.	.	.	.	.	.	.	.	.	.	.
5523	1	TOX	NM_014729	chr8	59880530	60194321	-	BH609601	chr8	60138218	-	.	.	.	.	.	.	.	.	.	.	.	.	.	.	.	.
2528	1,25	CA8	NM_004056	chr8	61263976	61356508	-	.	.	.	.	.	.	.	.	.	.	.	.	.	.	.	.	.	.	.	.
2738	1,25	RAB2A	NM_001242644	chr8	61592022	61698757	+	.	.	.	.	.	.	.	.	.	.	.	.	.	.	.	.	.	.	.	.
3349	1	CHD7	NM_017780	chr8	61753877	61943140	+	.	.	.	.	.	.	.	.	.	.	.	.	.	.	.	.	.	.	.	.
1259	2	ASPH	NM_001164754	chr8	62698822	62789753	-	CL800048	chr8	62781509	-	.	.	.	.	.	.	.	.	.	.	.	.	.	.	.	.
3882	1	GGH	NM_003878	chr8	64090192	64114164	-	.	.	.	.	.	.	.	.	.	.	.	.	.	.	.	.	.	.	.	.
7062	0,5	TTPA	NM_000370	chr8	64134601	64161166	-	.	.	.	.	.	.	.	.	.	.	.	.	.	.	.	.	.	.	.	.
2025	1,5	BHLHE22	NM_152414	chr8	65655348	65658745	+	.	.	.	.	.	.	.	.	.	.	.	.	.	.	.	.	.	.	.	.
4736	1	PDE7A	NM_002603	chr8	66789122	66863883	-	CL529682	chr8	66832415	-	.	.	.	.	.	.	.	.	.	.	.	.	.	.	.	.
6218	0,5	CRH	NM_000756	chr8	67251165	67253400	-	.	.	.	.	.	.	.	.	.	.	.	.	.	.	.	.	.	.	.	.
523	3	ADHFE1	NM_144650	chr8	67507271	67543598	+	.	.	.	.	.	.	.	.	.	.	.	.	.	.	.	.	.	.	.	.
6110	0,5	C8orf46	NM_152765	chr8	67568044	67593313	+	.	.	.	.	.	.	.	.	.	.	.	.	.	.	.	.	.	.	.	.
2397	1,5	SGK3	NM_170709	chr8	67849969	67936811	+	AY517043.1	chr8	67926929	-	.	.	.	.	.	.	.	.	.	.	.	.	.	.	.	.
1016	2,25	COPS5	NM_006837	chr8	68117868	68137116	-	.	.	.	.	.	.	.	.	.	.	.	.	.	.	.	.	.	.	.	.
3473	1	CSPP1	NM_024790	chr8	68139156	68271403	+	BH609602	chr8	68216270	-	.	.	.	.	.	.	.	.	.	.	.	.	.	.	.	.
1680	2	PREX2	NM_025170	chr8	69027156	69180208	+	.	.	.	.	.	.	.	.	D5_GVJ7CIC0	chr8	69070979	-	.	.	.	.	.	.	.	.
688	3	SULF1	NM_001128206	chr8	70567581	70735701	+	.	.	.	.	.	.	.	.	.	.	.	.	.	.	.	.	.	.	.	.
6932	0,5	SLCO5A1	NM_001146009	chr8	70747121	70907958	-	.	.	.	.	.	.	.	.	.	.	.	.	.	.	.	.	.	.	.	.
186	4,5	NCOA2	NM_006540	chr8	71186820	71478574	-	.	.	.	.	AY516461	chr8	71288997	-	.	.	.	.	.	.	.	.	.	.	.	.

Table S2

tumor associated genes								HIV				MLV				MMTV				MMTV(SIN)				MMTV(SIN)arrest			
rank	score	gene symbol	RefSEq	chrom	txStart	txEnd	strand	integrant	chr	position	strand	integrant	chr	position	strand	integrant	chr	position	strand	integrant	chr	position	strand	integrant	chr	position	strand
2130	1,5	EYA1	NM_172059	chr8	72272221	72431533	-	.	.	.	.	AY515936	chr8	72379175	-	.	.	.	.	.	.	.	.	.	.	.	.
6492	0,5	KCNB2	NM_004770	chr8	73612179	74013138	+	.	.	.	.	.	.	.	.	.	.	.	.	.	.	.	.	.	.	.	.
5457	1	TERF1	NM_003218	chr8	74083650	74122541	+	.	.	.	.	.	.	.	.	.	.	.	.	.	.	.	.	.	.	.	.
7508	0,25	RDH10	NM_172037	chr8	74369390	74400074	+	.	.	.	.	.	.	.	.	.	.	.	.	.	.	.	.	.	.	.	.
6980	0,5	STAU2	NM_001164384	chr8	74624390	74822497	-	CL529764	chr8	74648450	-	.	.	.	.	.	.	.	.	.	.	.	.	.	.	.	.
4320	1	LY96	NM_001195797	chr8	75066117	75103861	+	.	.	.	.	.	.	.	.	.	.	.	.	.	.	.	.	.	.	.	.
4791	1	PI15	NM_015886	chr8	75899326	75929819	+	.	.	.	.	.	.	.	.	.	.	.	.	.	.	.	.	.	.	.	.
3459	1	CRISPLD1	NM_031461	chr8	76059262	76109350	+	.	.	.	.	.	.	.	.	.	.	.	.	.	.	.	.	.	.	.	.
4064	1	HNF4G	NM_004133	chr8	76614757	76641616	+	.	.	.	.	.	.	.	.	.	.	.	.	.	.	.	.	.	.	.	.
7120	0,5	ZFHX4	NM_024721	chr8	77756069	77942076	+	.	.	.	.	AY516024	chr8	77885616	+	.	.	.	.	.	.	.	.	.	.	.	.
4818	1	PKIA	NM_006823	chr8	79590890	79680057	+	CL529716	chr8	79605251	+	.	.	.	.	.	.	.	.	.	.	.	.	.	.	.	.
2791	1,25	STMN2	NM_001199214	chr8	80685603	80740965	+	.	.	.	.	.	.	.	.	.	.	.	.	.	.	.	.	.	.	.	.
4035	1	HEY1	NM_012258	chr8	80838799	80842653	-	.	.	.	.	.	.	.	.	.	.	.	.	.	.	.	.	.	.	.	.
7304	0,25	FABP5	NM_001444	chr8	82355272	82359567	+	CL529664	chr8	82359539	+	.	.	.	.	.	.	.	.	.	.	.	.	.	.	.	.
384	3,5	IMPA1	NM_005536	chr8	82731705	82761144	-	.	.	.	.	.	.	.	.	.	.	.	.	.	.	.	.	.	.	.	.
2118	1,5	E2F5	NM_001951	chr8	86276870	86314006	+	.	.	.	.	.	.	.	.	.	.	.	.	.	.	.	.	.	.	.	.
3252	1	CA13	NM_198584	chr8	86344967	86383554	+	.	.	.	.	.	.	.	.	.	.	.	.	.	.	.	.	.	.	.	.
2524	1,25	CA1	NM_001128831	chr8	86427709	86477594	-	.	.	.	.	.	.	.	.	.	.	.	.	.	.	.	.	.	.	.	.
1883	1,75	CA3	NM_005181	chr8	86538307	86548519	+	.	.	.	.	.	.	.	.	.	.	.	.	.	.	.	.	.	.	.	.
2525	1,25	CA2	NM_000067	chr8	86563382	86580973	+	.	.	.	.	.	.	.	.	.	.	.	.	.	.	.	.	.	.	.	.
4969	1	PSKH2	NM_033126	chr8	87129806	87150967	-	.	.	.	.	.	.	.	.	.	.	.	.	.	.	.	.	.	.	.	.
3087	1	ATP6V0D2	NM_152565	chr8	87180254	87235570	+	.	.	.	.	.	.	.	.	.	.	.	.	.	.	.	.	.	.	.	.
5709	1	WWP1	NM_007013	chr8	87424109	87549294	+	AY517193.1	chr8	87529852	-	.	.	.	.	.	.	.	.	.	.	.	.	.	.	.	.
5709	1	WWP1	NM_007013	chr8	87424109	87549294	+	CL800345	chr8	87548736	+	.	.	.	.	.	.	.	.	.	.	.	.	.	.	.	.
5709	1	WWP1	NM_007013	chr8	87424109	87549294	+	CL800082	chr8	87492431	+	.	.	.	.	.	.	.	.	.	.	.	.	.	.	.	.
5709	1	WWP1	NM_007013	chr8	87424109	87549294	+	CL529624	chr8	87462003	+	.	.	.	.	.	.	.	.	.	.	.	.	.	.	.	.
5709	1	WWP1	NM_007013	chr8	87424109	87549294	+	CL529625	chr8	87463620	-	.	.	.	.	.	.	.	.	.	.	.	.	.	.	.	.
6619	0,5	MMP16	NM_005941	chr8	89118575	89408833	-	.	.	.	.	.	.	.	.	.	.	.	.	.	.	.	.	.	.	.	.
5126	1	RIPK2	NM_003821	chr8	90839109	90872433	+	CL529025	chr8	90863427	-	.	.	.	.	.	.	.	.	.	.	.	.	.	.	.	.
143	5	NBN	NM_002485	chr8	91014739	91066075	-	.	.	.	.	.	.	.	.	.	.	.	.	.	.	.	.	.	.	.	.
6119	0,5	CALB1	NM_004929	chr8	91140013	91164283	-	.	.	.	.	.	.	.	.	.	.	.	.	.	.	.	.	.	.	.	.
302	4	RUNX1T1	NM_175634	chr8	93036370	93184630	-	CL529027	chr8	93127715	-	.	.	.	.	.	.	.	.	.	.	.	.	.	.	.	.
6381	0,5	GEM	NM_181702	chr8	95330660	95343723	-	.	.	.	.	.	.	.	.	.	.	.	.	.	.	.	.	.	.	.	.
662	3	RAD54B	NM_012415	chr8	95453363	95556519	-	.	.	.	.	.	.	.	.	.	.	.	.	.	.	.	.	.	.	.	.
5526	1	TP53INP1	NM_033285	chr8	96007375	96030791	-	.	.	.	.	.	.	.	.	.	.	.	.	.	.	.	.	.	.	.	.
844	2,5	GDF6	NM_001001557	chr8	97223733	97242196	-	.	.	.	.	.	.	.	.	.	.	.	.	.	.	.	.	.	.	.	.
5646	1	UQCRB	NM_001254752	chr8	97308079	97317038	-	.	.	.	.	.	.	.	.	.	.	.	.	.	.	.	.	.	.	.	.
4981	1	PTDSS1	NM_014754	chr8	97343342	97415950	+	.	.	.	.	.	.	.	.	.	.	.	.	.	.	.	.	.	.	.	.
1741	2	SDC2	NM_002998	chr8	97575057	97693213	+	.	.	.	.	.	.	.	.	.	.	.	.	.	.	.	.	.	.	.	.
7057	0,5	TSPYL5	NM_033512	chr8	98354889	98359352	-	.	.	.	.	.	.	.	.	.	.	.	.	.	.	.	.	.	.	.	.
6509	0,5	KCNS2	NM_020697	chr8	99508425	99512199	+	.	.	.	.	.	.	.	.	.	.	.	.	.	.	.	.	.	.	.	.
5381	1	STK3	NM_006281	chr8	99536034	99907085	-	.	.	.	.	.	.	.	.	.	.	.	.	.	.	.	.	2602_226_1	chr8	99926629	-
1850	2	VPS13B	NM_152564	chr8	100094669	100958990	+	CL800350	chr8	100107422	-	.	.	.	.	.	.	.	.	.	.	.	.	.	.	.	.
1850	2	VPS13B	NM_152564	chr8	100094669	100958990	+	CL529471	chr8	100121961	+	.	.	.	.	.	.	.	.	.	.	.	.	.	.	.	.
1850	2	VPS13B	NM_152564	chr8	100094669	100958990	+	CL529327	chr8	100597640	+	.	.	.	.	.	.	.	.	.	.	.	.	.	.	.	.
550	3	COX6C	NM_004374	chr8	100959398	100975418	-	.	.	.	.	.	.	.	.	.	.	.	.	.	.	.	.	.	.	.	.
926	2,5	POLR2K	NM_005034	chr8	101232014	101235406	+	.	.	.	.	.	.	.	.	.	.	.	.	.	.	.	.	.	.	.	.
6016	0,5	ANKRD46	NM_198401	chr8	101602175	101641190	-	.	.	.	.	.	.	.	.	.	.	.	.	.	.	.	.	.	.	.	.
6712	0,5	PABPC1	NM_002568	chr8	101784319	101803491	-	BH609605	chr8	101797642	-	.	.	.	.	.	.	.	.	.	.	.	.	.	.	.	.
7112	0,5	YWHAZ	NM_001135702	chr8	101999979	102031975	-	.	.	.	.	.	.	.	.	.	.	.	.	.	.	.	.	.	.	.	.
6649	0,5	NCALD	NM_001040624	chr8	102767945	103205738	-	.	.	.	.	.	.	.	.	.	.	.	.	.	.	.	.	.	.	.	.

Table S2

tumor associated genes								HIV				MLV				MMTV				MMTV(SIN)				MMTV(SIN)arrest			
rank	score	gene symbol	RefSEq	chrom	txStart	txEnd	strand	integrant	chr	position	strand	integrant	chr	position	strand	integrant	chr	position	strand	integrant	chr	position	strand	integrant	chr	position	strand
673	3	RRM2B	NM_001172478	chr8	103285904	103320522	-	.	.	.	.	.	.	.	.	.	.	.	.	.	.	.	.	.	.	.	.
700	3	UBR5	NM_015902	chr8	103333677	103494093	-	CL529574	chr8	103341459	+	.	.	.	.	.	.	.	.	.	.	.	.	.	.	.	.
700	3	UBR5	NM_015902	chr8	103333677	103494093	-	BH609606	chr8	103488764	+	.	.	.	.	.	.	.	.	.	.	.	.	.	.	.	.
3090	1	ATP6V1C1	NM_001695	chr8	104102423	104154461	+	.	.	.	.	.	.	.	.	.	.	.	.	.	.	.	.	.	.	.	.
839	2,5	FZD6	NM_001164615	chr8	104379836	104414270	+	.	.	.	.	.	.	.	.	.	.	.	.	.	.	.	.	.	.	.	.
2098	1,5	CTHRC1	NM_001256099	chr8	104453836	104464408	+	.	.	.	.	.	.	.	.	.	.	.	.	.	.	.	.	.	.	.	.
948	2,5	RIMS2	NM_014677	chr8	104900591	105335832	+	.	.	.	.	.	.	.	.	.	.	.	.	.	.	.	.	1630_156_4	chr8	104913854	-
2110	1,5	DPYS	NM_001385	chr8	105460827	105548453	-	.	.	.	.	.	.	.	.	.	.	.	.	.	.	.	.	.	.	.	.
1867	2	ZFPM2	NM_012082	chr8	106400322	106885943	+	.	.	.	.	.	.	.	.	.	.	.	.	s1738_279	chr8	106770741	-	1665_159_1	chr8	106631644	-
783	2,5	ANGPT1	NM_001146	chr8	108330885	108579430	-	CL800683	chr8	108376391	-	.	.	.	.	.	.	.	.	.	.	.	.	.	.	.	.
2383	1,5	RSPO2	NM_178565	chr8	108980719	109165089	-	.	.	.	.	.	.	.	.	.	.	.	.	.	.	.	.	2391_207_2	chr8	108992611	-
7299	0,25	EIF3E	NM_001568	chr8	109283147	109330135	-	AY517184.1	chr8	109305000	+	.	.	.	.	.	.	.	.	.	.	.	.	.	.	.	.
7299	0,25	EIF3E	NM_001568	chr8	109283147	109330135	-	AY516971.1	chr8	109290797	+	.	.	.	.	.	.	.	.	.	.	.	.	.	.	.	.
7451	0,25	NUDCD1	NM_032869	chr8	110322323	110415526	-	.	.	.	.	.	.	.	.	.	.	.	.	.	.	.	.	.	.	.	.
2575	1,25	EBAG9	NM_004215	chr8	110621485	110646567	+	.	.	.	.	.	.	.	.	.	.	.	.	.	.	.	.	.	.	.	.
6510	0,5	KCNV1	NM_014379	chr8	111048408	111056135	-	.	.	.	.	.	.	.	.	.	.	.	.	.	.	.	.	.	.	.	.
244	4	CSMD3	NM_198124	chr8	113304332	114458558	-	.	.	.	.	.	.	.	.	.	.	.	.	.	.	.	.	.	.	.	.
1824	2	TRPS1	NM_014112	chr8	116489899	116750402	-	CL800809	chr8	116569210	+	.	.	.	.	.	.	.	.	.	.	.	.	.	.	.	.
942	2,5	RAD21	NM_006265	chr8	117927353	117956286	-	CL800002	chr8	117938062	+	.	.	.	.	.	.	.	.	.	.	.	.	.	.	.	.
64	6,5	EXT1	NM_000127	chr8	118880782	119193239	-	CL529513	chr8	119170174	+	AY516196	chr8	119098953	+	.	.	.	.	.	.	.	.	.	.	.	.
6846	0,5	SAMD12	NM_207506	chr8	119459407	119703365	-	.	.	.	.	.	.	.	.	.	.	.	.	.	.	.	.	.	.	.	.
1975	1,75	TNFRSF11B	NM_002546	chr8	120004976	120033564	-	.	.	.	.	.	.	.	.	.	.	.	.	.	.	.	.	.	.	.	.
2237	1,5	MAL2	NM_052886	chr8	120289790	120327095	+	.	.	.	.	.	.	.	.	.	.	.	.	.	.	.	.	.	.	.	.
404	3,5	NOV	NM_002514	chr8	120497732	120505859	+	.	.	.	.	.	.	.	.	.	.	.	.	.	.	.	.	.	.	.	.
323	3,75	ENPP2	NR_045555	chr8	120638497	120674429	-	.	.	.	.	AY516405	chr8	120697964	+	.	.	.	.	.	.	.	.	.	.	.	.
5426	1	TAF2	NM_003184	chr8	120812194	120914255	-	.	.	.	.	.	.	.	.	.	.	.	.	.	.	.	.	.	.	.	.
3412	1	COL14A1	NM_021110	chr8	121206532	121453454	+	.	.	.	.	.	.	.	.	.	.	.	.	.	.	.	.	.	.	.	.
5310	1	SNTB1	NM_021021	chr8	121617165	121893490	-	.	.	.	.	.	.	.	.	.	.	.	.	.	.	.	.	.	.	.	.
4017	1	HAS2	NM_005328	chr8	122694451	122722811	-	.	.	.	.	.	.	.	.	.	.	.	.	.	.	.	.	.	.	.	.
7310	0,25	FAM83A	NM_032899	chr8	124263932	124290388	+	.	.	.	.	.	.	.	.	.	.	.	.	.	.	.	.	.	.	.	.
4543	1	NDUFB9	NM_005005	chr8	125620523	125631408	+	.	.	.	.	.	.	.	.	.	.	.	.	.	.	.	.	.	.	.	.
4460	1	MTSS1	NM_014751	chr8	125632208	125809911	-	.	.	.	.	.	.	.	.	.	.	.	.	65_1_2174_8	chr8	125776874	+	.	.	.	.
5346	1	SQLE	NM_003129	chr8	126079900	126103707	+	.	.	.	.	.	.	.	.	.	.	.	.	.	.	.	.	.	.	.	.
45	7,5	MYC	NM_002467	chr8	128817496	128822862	+	.	.	.	.	.	.	.	.	.	.	.	.	.	.	.	.	.	.	.	.
6339	0,5	FAM49B	NR_046359	chr8	130921020	131021300	-	CL529029	chr8	130952218	+	AY515930	chr8	130993170	+	.	.	.	.	.	.	.	.	.	.	.	.
6339	0,5	FAM49B	NR_046359	chr8	130921020	131021300	-	AY517424.1	chr8	131001798	+	AY515930	chr8	130993170	+	.	.	.	.	.	.	.	.	.	.	.	.
6339	0,5	FAM49B	NR_046359	chr8	130921020	131021300	-	CL800038	chr8	131052367	-	AY515930	chr8	130993170	+	.	.	.	.	.	.	.	.	.	.	.	.
6339	0,5	FAM49B	NR_046359	chr8	130921020	131021300	-	BH609608	chr8	130957028	-	AY515930	chr8	130993170	+	.	.	.	.	.	.	.	.	.	.	.	.
1222	2	ADCY8	NM_001115	chr8	131861728	132122017	-	.	.	.	.	.	.	.	.	_189_1_159	chr8	132097350	-	.	.	.	.	.	.	.	.
6508	0,5	KCNQ3	NM_001204824	chr8	133202286	133528691	-	.	.	.	.	.	.	.	.	.	.	.	.	.	.	.	.	.	.	.	.
1173	2,25	TG	NM_003235	chr8	133948386	134216325	+	.	.	.	.	.	.	.	.	.	.	.	.	01_1_2477_12	chr8	134053791	-	.	.	.	.
5975	0,75	WISP1	NM_001204869	chr8	134272463	134313114	+	.	.	.	.	.	.	.	.	.	.	.	.	.	.	.	.	.	.	.	.
1778	2	ST3GAL1	NM_003033	chr8	134536272	134653365	-	.	.	.	.	.	.	.	.	.	.	.	.	.	.	.	.	.	.	.	.
295	4	PTK2	NM_001199649	chr8	141737662	142080594	-	.	.	.	.	.	.	.	.	.	.	.	.	.	.	.	.	3181_310_1	chr8	141750072	-
2732	1,25	PTP4A3	NM_007079	chr8	142501188	142510802	+	.	.	.	.	.	.	.	.	.	.	.	.	.	.	.	.	.	.	.	.
792	2,5	BAI1	NM_001702	chr8	143542378	143623370	+	.	.	.	.	.	.	.	.	.	.	.	.	.	.	.	.	.	.	.	.
4966	1	PSCA	NM_005672	chr8	143758875	143761147	+	.	.	.	.	AY516772	chr8	143760638	-	.	.	.	.	.	.	.	.	.	.	.	.
2160	1,5	GML	NM_002066	chr8	143913218	143925264	+	.	.	.	.	.	.	.	.	.	.	.	.	.	.	.	.	.	.	.	.
1336	2	CYP11B1	NM_001026213	chr8	143950774	143958238	-	.	.	.	.	.	.	.	.	.	.	.	.	.	.	.	.	.	.	.	.
3504	1	CYP11B2	NM_000498	chr8	143988976	143996261	-	.	.	.	.	.	.	.	.	.	.	.	.	.	.	.	.	.	.	.	.
4318	1	LY6H	NM_001130478	chr8	144310705	144313428	-	.	.	.	.	.	.	.	.	.	.	.	.	.	.	.	.	.	.	.	.

Table S2

tumor associated genes								HIV				MLV				MMTV				MMTV(SIN)				MMTV(SIN)arrest			
rank	score	gene symbol	RefSEq	chrom	txStart	txEnd	strand	integrant	chr	position	strand	integrant	chr	position	strand	integrant	chr	position	strand	integrant	chr	position	strand	integrant	chr	position	strand
5014	1	PYCRL	NM_023078	chr8	144757225	144762927	-	.	.	.	.	.	.	.	.	.	.	.	.	.	.	.	.	.	.	.	.
5579	1	TSTA3	NM_003313	chr8	144765930	144770875	-	.	.	.	.	.	.	.	.	.	.	.	.	.	.	.	.	.	.	.	.
2660	1,25	MAPK15	NM_139021	chr8	144870494	144876621	+	.	.	.	.	.	.	.	.	.	.	.	.	.	.	.	.	.	.	.	.
4665	1	OPLAH	NM_017570	chr8	145178154	145187572	-	CL529033	chr8	145184753	+	.	.	.	.	.	.	.	.	.	.	.	.	.	.	.	.
3937	1	GPAA1	NM_003801	chr8	145209511	145213107	+	.	.	.	.	.	.	.	.	.	.	.	.	.	.	.	.	.	.	.	.
3502	1	CYC1	NM_001916	chr8	145221947	145224416	+	.	.	.	.	.	.	.	.	.	.	.	.	.	.	.	.	.	.	.	.
6063	0,5	BOP1	NM_015201	chr8	145456863	145485928	-	CL799658	chr8	145482815	+	.	.	.	.	.	.	.	.	.	.	.	.	.	.	.	.
2616	1,25	HSF1	NM_005526	chr8	145486077	145509193	+	.	.	.	.	.	.	.	.	.	.	.	.	.	.	.	.	.	.	.	.
3564	1	DGAT1	NM_012079	chr8	145509054	145521375	-	.	.	.	.	.	.	.	.	.	.	.	.	.	.	.	.	.	.	.	.
3751	1	FBXL6	NM_012162	chr8	145549895	145552991	-	.	.	.	.	.	.	.	.	.	.	.	.	.	.	.	.	.	.	.	.
2908	1	ADCK5	NM_174922	chr8	145568538	145589261	+	CL800745	chr8	145585859	-	.	.	.	.	.	.	.	.	.	.	.	.	.	.	.	.
2908	1	ADCK5	NM_174922	chr8	145568538	145589261	+	CL529005	chr8	145580157	+	.	.	.	.	.	.	.	.	.	.	.	.	.	.	.	.
4230	1	KIFC2	NM_145754	chr8	145662545	145670307	+	.	.	.	.	.	.	.	.	.	.	.	.	.	.	.	.	.	.	.	.
4913	1	PPP1R16A	NM_032902	chr8	145692916	145698312	+	.	.	.	.	.	.	.	.	.	.	.	.	.	.	.	.	.	.	.	.
1061	2,25	GPT	NM_005309	chr8	145700272	145703363	+	.	.	.	.	.	.	.	.	.	.	.	.	.	.	.	.	.	.	.	.
228	4,25	RECQL4	NM_004260	chr8	145707474	145714018	-	CL800788	chr8	145712094	+	.	.	.	.	.	.	.	.	.	.	.	.	.	.	.	.
7227	0,25	CBWD1	NM_001145356	chr9	111037	169075	-	.	.	.	.	.	.	.	.	.	.	.	.	.	.	.	.	.	.	.	.
6114	0,5	C9orf66	NM_152569	chr9	203107	205893	-	.	.	.	.	.	.	.	.	.	.	.	.	.	.	.	.	.	.	.	.
2570	1,25	DOCK8	NM_203447	chr9	204864	455259	+	.	.	.	.	.	.	.	.	.	.	.	.	.	.	.	.	.	.	.	.
7381	0,25	KANK1	NM_153186	chr9	696805	736106	+	AY517189.1	chr9	668521	-	.	.	.	.	.	.	.	.	.	.	.	.	.	.	.	.
7282	0,25	DMRT1	NM_021951	chr9	831689	959090	+	.	.	.	.	.	.	.	.	.	.	.	.	.	.	.	.	.	.	.	.
6270	0,5	DMRT3	NM_021240	chr9	966963	981732	+	.	.	.	.	.	.	.	.	.	.	.	.	.	.	.	.	.	.	.	.
7283	0,25	DMRT2	NM_006557	chr9	1040345	1047554	+	.	.	.	.	.	.	.	.	.	.	.	.	.	.	.	.	.	.	.	.
1167	2,25	SMARCA2	NM_003070	chr9	2005341	2183623	+	.	.	.	.	.	.	.	.	.	.	.	.	.	.	.	.	.	.	.	.
1849	2	VLDLR	NM_001018056	chr9	2611792	2644485	+	.	.	.	.	.	.	.	.	.	.	.	.	.	.	.	.	.	.	.	.
6511	0,5	KCNV2	NM_133497	chr9	2707525	2720037	+	.	.	.	.	.	.	.	.	.	.	.	.	.	.	.	.	.	.	.	.
6515	0,5	KIAA0020	NM_014878	chr9	2794154	2834130	-	.	.	.	.	.	.	.	.	.	.	.	.	.	.	.	.	.	.	.	.
5961	0,75	SLC1A1	NM_004170	chr9	4480426	4577469	+	.	.	.	.	.	.	.	.	.	.	.	.	.	.	.	.	.	.	.	.
4889	1	PPAPDC2	NM_203453	chr9	4652293	4655272	+	.	.	.	.	.	.	.	.	.	.	.	.	.	.	.	.	.	.	.	.
2938	1	AK3	NM_001199856	chr9	4699556	4716227	-	.	.	.	.	.	.	.	.	.	.	.	.	.	.	.	.	.	.	.	.
5082	1	RCL1	NM_005772	chr9	4782833	4851064	+	.	.	.	.	.	.	.	.	.	.	.	.	.	.	.	.	.	.	.	.
33	8,25	JAK2	NM_004972	chr9	4975244	5118183	+	.	.	.	.	.	.	.	.	.	.	.	.	.	.	.	.	.	.	.	.
5128	1	RLN2	NM_005059	chr9	5289867	5294580	-	.	.	.	.	.	.	.	.	.	.	.	.	.	.	.	.	.	.	.	.
4408	1	MLANA	NM_005511	chr9	5880908	5899822	+	.	.	.	.	.	.	.	.	.	.	.	.	.	.	.	.	.	.	.	.
6475	0,5	IL33	NM_001199640	chr9	6205785	6247983	+	.	.	.	.	.	.	.	.	.	.	.	.	.	.	.	.	.	.	.	.
1840	2	UHRF2	NR_046386	chr9	6403150	6497051	+	.	.	.	.	.	.	.	.	.	.	.	.	.	.	.	.	.	.	.	.
3902	1	GLDC	NM_000170	chr9	6522463	6635692	-	.	.	.	.	.	.	.	.	.	.	.	.	.	.	.	.	.	.	.	.
4209	1	KDM4C	NM_001146694	chr9	6747640	7160920	+	CL529458	chr9	7054388	+	.	.	.	.	.	.	.	.	.	.	.	.	.	.	.	.
296	4	PTPRD	NM_001171025	chr9	8304245	8723946	-	BH609613	chr9	9529773	+	.	.	.	.	.	.	.	.	.	.	.	.	.	.	.	.
1830	2	TYRP1	NM_000550	chr9	12683385	12700266	+	.	.	.	.	.	.	.	.	.	.	.	.	.	.	.	.	.	.	.	.
4427	1	MPDZ	NM_003829	chr9	13095702	13269563	-	.	.	.	.	.	.	.	.	.	.	.	.	.	.	.	.	.	.	.	.
3803	1	FREM1	NM_001177704	chr9	14724663	14769531	-	.	.	.	.	.	.	.	.	.	.	.	.	.	.	.	.	.	.	.	.
5304	1	SNAPC3	NM_001039697	chr9	15412781	15451627	+	.	.	.	.	.	.	.	.	.	.	.	.	.	.	.	.	.	.	.	.
1696	2	PSIP1	NM_033222	chr9	15454064	15501003	-	CL800603	chr9	15468584	+	.	.	.	.	.	.	.	.	.	.	.	.	.	.	.	.
2027	1,5	BNC2	NM_017637	chr9	16399500	16860786	-	.	.	.	.	.	.	.	.	.	.	.	.	s1861_327	chr9	16563499	+	.	.	.	.
427	3,5	SH3GL2	NM_003026	chr9	17568952	17787122	+	.	.	.	.	.	.	.	.	.	.	.	.	.	.	.	.	.	.	.	.
1211	2	ADAMTSL1	NM_001040272	chr9	18464078	18900947	+	.	.	.	.	.	.	.	.	.	.	.	.	.	.	.	.	1770_164_1	chr9	18723082	-
1730	2	RPS6	NM_001010	chr9	19366253	19370235	-	.	.	.	.	.	.	.	.	.	.	.	.	.	.	.	.	.	.	.	.
2486	1,25	ACER2	NM_001010887	chr9	19398924	19442500	+	.	.	.	.	.	.	.	.	.	.	.	.	.	.	.	.	.	.	.	.
7552	0,25	SLC24A2	NM_001193288	chr9	19497449	19777017	-	.	.	.	.	.	.	.	.	.	.	.	.	.	.	.	.	.	.	.	.
1538	2	MLLT3	NM_004529	chr9	20334967	20612514	-	.	.	.	.	AY516371	chr9	20598051	+	.	.	.	.	s1701_266	chr9	20485907	+	.	.	.	.

Table S2

tumor associated genes								HIV				MLV				MMTV				MMTV(SIN)				MMTV(SIN)arrest			
rank	score	gene symbol	RefSEq	chrom	txStart	txEnd	strand	integrant	chr	position	strand	integrant	chr	position	strand	integrant	chr	position	strand	integrant	chr	position	strand	integrant	chr	position	strand
1538	2	MLLT3	NM_004529	chr9	20334967	20612514	-	.	.	.	.	AY516195	chr9	20610185	+	.	.	.	.	s1701_266	chr9	20485907	+	.	.	.	.
1699	2	PTPLAD2	NM_001010915	chr9	20996364	21021635	-	.	.	.	.	.	.	.	.	.	.	.	.	.	.	.	.	.	.	.	.
1074	2,25	IFNB1	NM_002176	chr9	21067103	21067943	-	.	.	.	.	.	.	.	.	.	.	.	.	.	.	.	.	.	.	.	.
6465	0,5	IFNA21	NM_002175	chr9	21155635	21156659	-	.	.	.	.	.	.	.	.	.	.	.	.	.	.	.	.	.	.	.	.
4237	1	KLHL9	NM_018847	chr9	21321017	21325429	-	.	.	.	.	.	.	.	.	.	.	.	.	.	.	.	.	.	.	.	.
1464	2	IFNA2	NM_000605	chr9	21374253	21375396	-	.	.	.	.	.	.	.	.	.	.	.	.	.	.	.	.	.	.	.	.
7367	0,25	IFNE	NM_176891	chr9	21470838	21472312	-	.	.	.	.	.	.	.	.	.	.	.	.	.	.	.	.	.	.	.	.
7424	0,25	MIR31	NR_029505	chr9	21502113	21502184	-	.	.	.	.	.	.	.	.	.	.	.	.	.	.	.	.	.	.	.	.
1940	1,75	MTAP	NM_002451	chr9	21792634	21855969	+	CL529035	chr9	21795426	+	.	.	.	.	.	.	.	.	.	.	.	.	.	.	.	.
1	16,75	CDKN2A	NM_000077	chr9	21957750	21965132	-	.	.	.	.	.	.	.	.	.	.	.	.	.	.	.	.	.	.	.	.
27	8,5	CDKN2B	NM_004936	chr9	21992901	21999312	-	.	.	.	.	.	.	.	.	.	.	.	.	.	.	.	.	.	.	.	.
7284	0,25	DMRTA1	NM_022160	chr9	22436839	22442472	+	.	.	.	.	.	.	.	.	.	.	.	.	.	.	.	.	.	.	.	.
1901	1,75	ELAVL2	NM_001171195	chr9	23680102	23811843	-	.	.	.	.	.	.	.	.	.	.	.	.	.	.	.	.	.	.	.	.
7608	0,25	TUSC1	NM_001004125	chr9	25666386	25668856	-	.	.	.	.	.	.	.	.	.	.	.	.	.	.	.	.	.	.	.	.
6747	0,5	PLAA	NM_001031689	chr9	26893367	26937468	-	.	.	.	.	.	.	.	.	.	.	.	.	.	.	.	.	.	.	.	.
7369	0,25	IFT74	NM_001099224	chr9	26946370	27026796	+	CL800577	chr9	27041497	-	AY516131	chr9	26938991	-	.	.	.	.	.	.	.	.	.	.	.	.
7405	0,25	LRRC19	NM_022901	chr9	26983134	26995691	-	.	.	.	.	.	.	.	.	.	.	.	.	.	.	.	.	.	.	.	.
196	4,5	TEK	NM_000459	chr9	27099146	27220172	+	.	.	.	.	.	.	.	.	.	.	.	.	.	.	.	.	3295_347_1	chr9	27198526	-
6111	0,5	C9orf11	NM_020641	chr9	27274653	27287137	-	.	.	.	.	.	.	.	.	.	.	.	.	.	.	.	.	.	.	.	.
7368	0,25	IFNK	NM_020124	chr9	27514311	27516496	+	.	.	.	.	.	.	.	.	.	.	.	.	.	.	.	.	.	.	.	.
6115	0,5	C9orf72	NM_018325	chr9	27536542	27563491	-	.	.	.	.	.	.	.	.	.	.	.	.	.	.	.	.	.	.	.	.
880	2,5	LINGO2	NM_001258282	chr9	27938083	29202998	-	CL800527	chr9	29192154	-	.	.	.	.	.	.	.	.	.	.	.	.	.	.	.	.
880	2,5	LINGO2	NM_001258282	chr9	27938083	29202998	-	CL800103	chr9	28851999	-	.	.	.	.	.	.	.	.	.	.	.	.	.	.	.	.
2487	1,25	ACO1	NM_002197	chr9	32374600	32440832	+	.	.	.	.	.	.	.	.	.	.	.	.	.	.	.	.	.	.	.	.
2806	1,25	TOPORS	NM_001195622	chr9	32530541	32542626	-	.	.	.	.	.	.	.	.	.	.	.	.	.	.	.	.	.	.	.	.
4541	1	NDUFB6	NM_001199987	chr9	32543523	32563182	-	.	.	.	.	.	.	.	.	.	.	.	.	.	.	.	.	.	.	.	.
1170	2,25	TAF1L	NM_153809	chr9	32619451	32625667	-	.	.	.	.	.	.	.	.	.	.	.	.	.	.	.	.	.	.	.	.
3013	1	APTX	NR_036578	chr9	32962603	32991610	-	.	.	.	.	.	.	.	.	.	.	.	.	.	.	.	.	.	.	.	.
3117	1	B4GALT1	NM_001497	chr9	33100638	33157356	-	.	.	.	.	.	.	.	.	.	.	.	.	.	.	.	.	.	.	.	.
5333	1	SPINK4	NM_014471	chr9	33230195	33238565	+	.	.	.	.	.	.	.	.	.	.	.	.	.	.	.	.	.	.	.	.
6047	0,5	BAG1	NM_001172415	chr9	33242469	33254759	-	.	.	.	.	.	.	.	.	.	.	.	.	.	.	.	.	.	.	.	.
4577	1	NFX1	NM_002504	chr9	33280417	33361155	+	.	.	.	.	.	.	.	.	.	.	.	.	.	.	.	.	.	.	.	.
6777	0,5	PRSS3	NM_002771	chr9	33785558	33789229	+	.	.	.	.	.	.	.	.	.	.	.	.	.	.	.	.	.	.	.	.
4645	1	NUDT2	NM_147173	chr9	34319503	34333711	+	.	.	.	.	.	.	.	.	.	.	.	.	.	.	.	.	.	.	.	.
6113	0,5	C9orf24	NM_147169	chr9	34369016	34371598	-	.	.	.	.	.	.	.	.	.	.	.	.	.	.	.	.	.	.	.	.
6196	0,5	CNTFR	NM_147164	chr9	34541429	34579735	-	.	.	.	.	.	.	.	.	.	.	.	.	.	.	.	.	.	.	.	.
1050	2,25	GALT	NM_001258332	chr9	34636585	34640595	+	.	.	.	.	.	.	.	.	.	.	.	.	.	.	.	.	.	.	.	.
2535	1,25	CCL21	NM_002989	chr9	34699001	34700147	-	.	.	.	.	.	.	.	.	.	.	.	.	.	.	.	.	.	.	.	.
257	4	FANCG	NM_004629	chr9	35063834	35070013	-	.	.	.	.	.	.	.	.	.	.	.	.	.	.	.	.	.	.	.	.
1622	2	PIGO	NM_152850	chr9	35078684	35086598	-	.	.	.	.	.	.	.	.	.	.	.	.	.	.	.	.	.	.	.	.
5458	1	TESK1	NM_006285	chr9	35595280	35600038	+	.	.	.	.	.	.	.	.	.	.	.	.	.	.	.	.	.	.	.	.
2043	1,5	CA9	NM_001216	chr9	35663914	35671154	+	.	.	.	.	.	.	.	.	.	.	.	.	.	.	.	.	.	.	.	.
7599	0,25	TPM2	NM_003289	chr9	35672922	35680053	-	.	.	.	.	.	.	.	.	.	.	.	.	.	.	.	.	.	.	.	.
1812	2	TLN1	NM_006289	chr9	35687333	35722392	-	CL529037	chr9	35709708	-	.	.	.	.	.	.	.	.	.	.	.	.	.	.	.	.
4601	1	NPR2	NM_003995	chr9	35782405	35799728	+	.	.	.	.	.	.	.	.	.	.	.	.	.	.	.	.	.	.	.	.
7510	0,25	RECK	NM_021111	chr9	36026909	36114452	+	.	.	.	.	.	.	.	.	.	.	.	.	.	.	.	.	.	.	.	.
2078	1,5	CLTA	NM_001076677	chr9	36180852	36202059	+	.	.	.	.	.	.	.	.	.	.	.	.	.	.	.	.	.	.	.	.
1427	2	GNE	NM_001190384	chr9	36204438	36248496	-	.	.	.	.	.	.	.	.	.	.	.	.	.	.	.	.	.	.	.	.
6825	0,5	RNF38	NM_022781	chr9	36326398	36390296	-	.	.	.	.	.	.	.	.	.	.	.	.	.	.	.	.	.	.	.	.
1107	2,25	MELK	NM_001256692	chr9	36562858	36667680	+	.	.	.	.	.	.	.	.	.	.	.	.	.	.	.	.	.	.	.	.
744	2,75	PAX5	NM_016734	chr9	36828530	37024476	-	.	.	.	.	.	.	.	.	.	.	.	.	.	.	.	.	.	.	.	.

Table S2

tumor associated genes								HIV				MLV				MMTV				MMTV(SIN)				MMTV(SIN)arrest			
rank	score	gene symbol	RefSEq	chrom	txStart	txEnd	strand	integrant	chr	position	strand	integrant	chr	position	strand	integrant	chr	position	strand	integrant	chr	position	strand	integrant	chr	position	strand
3967	1	GRHPR	NM_012203	chr9	37412706	37426986	+	.	.	.	.	.	.	.	.	.	.	.	.	.	.	.	.	.	.	.	.
4868	1	POLR1E	NM_022490	chr9	37475944	37493693	+	.	.	.	.	.	.	.	.	.	.	.	.	.	.	.	.	.	.	.	.
3753	1	FBXO10	NM_012166	chr9	37500888	37566250	-	.	.	.	.	.	.	.	.	.	.	.	.	.	.	.	.	3162_306_1	chr9	37510621	+
6888	0,5	SHB	NM_003028	chr9	37905894	38059210	-	.	.	.	.	.	.	.	.	.	.	.	.	.	.	.	.	.	.	.	.
2948	1	ALDH1B1	NM_000692	chr9	38382660	38388662	+	.	.	.	.	.	.	.	.	.	.	.	.	.	.	.	.	.	.	.	.
918	2,5	PIP5K1B	NM_003558	chr9	70510435	70813911	+	CL800622	chr9	70562766	-	.	.	.	.	.	.	.	.	.	.	.	.	.	.	.	.
936	2,5	PRKACG	NM_002732	chr9	70817268	70818859	-	.	.	.	.	.	.	.	.	.	.	.	.	.	.	.	.	.	.	.	.
3823	1	FXN	NM_001161706	chr9	70840298	70904914	+	.	.	.	.	.	.	.	.	.	.	.	.	.	.	.	.	.	.	.	.
6022	0,5	APBA1	NM_001163	chr9	71232268	71477095	-	.	.	.	.	.	.	.	.	.	.	.	.	.	.	.	.	.	.	.	.
7051	0,5	TRPM3	NM_024971	chr9	72339785	72673794	-	.	.	.	.	AY516361	chr9	72394661	+	.	.	.	.	.	.	.	.	.	.	.	.
374	3,5	GDA	NM_001242505	chr9	73954086	74056960	+	.	.	.	.	.	.	.	.	.	.	.	.	.	.	.	.	.	.	.	.
443	3,25	ALDH1A1	NM_000689	chr9	74705397	74758053	-	CL799999	chr9	74745388	-	.	.	.	.	.	.	.	.	.	.	.	.	.	.	.	.
1874	1,75	ANXA1	NM_000700	chr9	74956600	74975127	+	.	.	.	.	AY516484	chr9	74956661	-	.	.	.	.	.	.	.	.	.	.	.	.
1728	2	RORB	NM_006914	chr9	76302071	76491937	+	.	.	.	.	.	.	.	.	.	.	.	.	.	.	.	.	.	.	.	.
7052	0,5	TRPM6	NM_001177310	chr9	76527230	76692456	-	.	.	.	.	.	.	.	.	.	.	.	.	.	.	.	.	.	.	.	.
4721	1	PCSK5	NM_001190482	chr9	77695379	78167075	+	CL529041	chr9	78164979	+	.	.	.	.	.	.	.	.	.	.	.	.	.	.	.	.
5103	1	RFK	NM_018339	chr9	78190252	78199264	-	.	.	.	.	.	.	.	.	.	.	.	.	.	.	.	.	.	.	.	.
1911	1,75	GCNT1	NM_001097633	chr9	78263887	78312152	+	.	.	.	.	.	.	.	.	.	.	.	.	.	.	.	.	.	.	.	.
4965	1	PRUNE2	NM_015225	chr9	78416111	78710823	-	.	.	.	.	.	.	.	.	_292_1_188	chr9	78584657	-	.	.	.	.	.	.	.	.
3915	1	GNA14	NM_004297	chr9	79227814	79453052	-	.	.	.	.	.	.	.	.	.	.	.	.	.	.	.	.	.	.	.	.
2165	1,5	GNAQ	NM_002072	chr9	79525010	79836039	-	CL529043	chr9	79614721	-	.	.	.	.	.	.	.	.	.	.	.	.	.	.	.	.
2726	1,25	PSAT1	NM_058179	chr9	80101878	80134829	+	.	.	.	.	.	.	.	.	.	.	.	.	.	.	.	.	.	.	.	.
5475	1	TLE4	NM_007005	chr9	81376697	81531476	+	.	.	.	.	.	.	.	.	.	.	.	.	.	.	.	.	.	.	.	.
1175	2,25	TLE1	NM_005077	chr9	83388417	83493416	-	.	.	.	.	.	.	.	.	.	.	.	.	.	.	.	.	.	.	.	.
3896	1	GKAP1	NM_001135953	chr9	85544155	85622572	-	.	.	.	.	.	.	.	.	.	.	.	.	.	.	.	.	.	.	.	.
631	3	NTRK2	NM_001007097	chr9	86474445	86620441	+	.	.	.	.	.	.	.	.	.	.	.	.	.	.	.	.	.	.	.	.
5999	0,5	AGTPBP1	NM_015239	chr9	87351273	87546764	-	CL529044	chr9	87502549	+	.	.	.	.	.	.	.	.	.	.	.	.	.	.	.	.
5999	0,5	AGTPBP1	NM_015239	chr9	87351273	87546764	-	CL529533	chr9	87473307	-	.	.	.	.	.	.	.	.	.	.	.	.	.	.	.	.
841	2,5	GAS1	NM_002048	chr9	88749096	88751924	-	.	.	.	.	.	.	.	.	.	.	.	.	.	.	.	.	.	.	.	.
458	3,25	DAPK1	NM_004938	chr9	89302575	89513369	+	.	.	.	.	.	.	.	.	.	.	.	.	.	.	.	.	.	.	.	.
7527	0,25	S1PR3	NM_005226	chr9	90796143	90809889	+	.	.	.	.	.	.	.	.	.	.	.	.	.	.	.	.	.	.	.	.
6889	0,5	SHC3	NM_016848	chr9	90810505	90983502	-	.	.	.	.	.	.	.	.	.	.	.	.	.	.	.	.	.	.	.	.
3377	1	CKS2	NM_001827	chr9	91115932	91121438	+	.	.	.	.	.	.	.	.	.	.	.	.	.	.	.	.	.	.	.	.
5912	0,75	GADD45G	NM_006705	chr9	91409746	91411289	+	.	.	.	.	.	.	.	.	.	.	.	.	.	.	.	.	.	.	.	.
6265	0,5	DIRAS2	NM_017594	chr9	92411933	92444928	-	.	.	.	.	.	.	.	.	.	.	.	.	.	.	.	.	.	.	.	.
757	2,75	SYK	NM_001135052	chr9	92603832	92700663	+	.	.	.	.	.	.	.	.	.	.	.	.	.	.	.	.	.	.	.	.
2015	1,5	AUH	NM_001698	chr9	93015917	93164027	-	CL529735	chr9	93061444	+	.	.	.	.	.	.	.	.	.	.	.	.	.	.	.	.
2015	1,5	AUH	NM_001698	chr9	93015917	93164027	-	CL529736	chr9	93131507	-	.	.	.	.	.	.	.	.	.	.	.	.	.	.	.	.
4574	1	NFIL3	NM_005384	chr9	93211147	93225965	-	.	.	.	.	.	.	.	.	.	.	.	.	.	.	.	.	.	.	.	.
670	3	ROR2	NM_004560	chr9	93524698	93752265	-	.	.	.	.	.	.	.	.	.	.	.	.	.	.	.	.	.	.	.	.
4104	1	IARS	NM_002161	chr9	94012445	94095859	-	CL529497	chr9	94091765	-	.	.	.	.	.	.	.	.	.	.	.	.	.	.	.	.
4104	1	IARS	NM_002161	chr9	94012445	94095859	-	CL529045	chr9	94088885	-	.	.	.	.	.	.	.	.	.	.	.	.	.	.	.	.
3337	1	CENPP	NM_001012267	chr9	94127561	94417258	+	CL799574	chr9	94185628	-	.	.	.	.	.	.	.	.	.	.	.	.	.	.	.	.
7455	0,25	OGN	NM_014057	chr9	94186069	94206758	-	.	.	.	.	.	.	.	.	.	.	.	.	.	.	.	.	.	.	.	.
1590	2	OMD	NM_005014	chr9	94216347	94226657	-	.	.	.	.	.	.	.	.	.	.	.	.	.	.	.	.	.	.	.	.
5879	0,75	ASPN	NM_001193335	chr9	94258309	94284665	-	.	.	.	.	.	.	.	.	.	.	.	.	.	.	.	.	.	.	.	.
2119	1,5	ECM2	NM_001197296	chr9	94295649	94338195	-	.	.	.	.	.	.	.	.	.	.	.	.	.	.	.	.	.	.	.	.
4160	1	IPPK	NM_022755	chr9	94415286	94472368	-	.	.	.	.	.	.	.	.	.	.	.	.	.	.	.	.	.	.	.	.
6059	0,5	BICD2	NM_015250	chr9	94513465	94566904	-	.	.	.	.	.	.	.	.	.	.	.	.	.	.	.	.	.	.	.	.
1857	2	WNK2	NM_006648	chr9	94987032	95122675	+	.	.	.	.	.	.	.	.	.	.	.	.	.	.	.	.	.	.	.	.
4989	1	PTPDC1	NM_001253830	chr9	95832911	95911960	+	.	.	.	.	.	.	.	.	.	.	.	.	.	.	.	.	.	.	.	.

Table S2

tumor associated genes								HIV				MLV				MMTV				MMTV(SIN)				MMTV(SIN)arrest			
rank	score	gene symbol	RefSEq	chrom	txStart	txEnd	strand	integrant	chr	position	strand	integrant	chr	position	strand	integrant	chr	position	strand	integrant	chr	position	strand	integrant	chr	position	strand
3746	1	FBP2	NM_003837	chr9	96360823	96395896	-	.	.	.	.	.	.	.	.	.	.	.	.	.	.	.	.	.	.	.	.
3745	1	FBP1	NM_001127628	chr9	96405237	96442352	-	.	.	.	.	.	.	.	.	.	.	.	.	.	.	.	.	.	.	.	.
256	4	FANCC	NM_000136	chr9	96901156	97119812	-	.	.	.	.	AY516338	chr9	97117075	+	.	.	.	.	.	.	.	.	.	.	.	.
83	6,25	PTCH1	NM_001083603	chr9	97245084	97319068	-	.	.	.	.	.	.	.	.	.	.	.	.	.	.	.	.	.	.	.	.
1454	2	HSD17B3	NM_000197	chr9	98037409	98104255	-	.	.	.	.	.	.	.	.	.	.	.	.	.	.	.	.	.	.	.	.
7133	0,5	ZNF367	NM_153695	chr9	98188045	98220490	-	.	.	.	.	.	.	.	.	.	.	.	.	.	.	.	.	.	.	.	.
1296	2	CDC14B	NM_033331	chr9	98302215	98421933	-	CL529046	chr9	98334359	-	.	.	.	.	.	.	.	.	.	.	.	.	.	.	.	.
7136	0,5	ZNF510	NM_014930	chr9	98557967	98580149	-	.	.	.	.	.	.	.	.	.	.	.	.	.	.	.	.	.	.	.	.
5451	1	TDRD7	NM_014290	chr9	99214122	99298226	+	.	.	.	.	.	.	.	.	.	.	.	.	.	.	.	.	.	.	.	.
6650	0,5	NCBP1	NM_002486	chr9	99435525	99475850	+	CL800553	chr9	99443747	-	.	.	.	.	.	.	.	.	.	.	.	.	.	.	.	.
6650	0,5	NCBP1	NM_002486	chr9	99435525	99475850	+	CL529047	chr9	99445488	+	.	.	.	.	.	.	.	.	.	.	.	.	.	.	.	.
125	5,25	XPA	NM_000380	chr9	99477011	99499512	-	.	.	.	.	.	.	.	.	.	.	.	.	.	.	.	.	.	.	.	.
4494	1	NANS	NM_018946	chr9	99858779	99885186	+	.	.	.	.	.	.	.	.	.	.	.	.	.	.	.	.	.	.	.	.
2594	1,25	GABBR2	NM_005458	chr9	100090184	100511300	-	.	.	.	.	.	.	.	.	.	.	.	.	.	.	.	.	.	.	.	.
309	4	TGFBR1	NM_001130916	chr9	100907232	100956294	+	.	.	.	.	.	.	.	.	.	.	.	.	.	.	.	.	.	.	.	.
2959	1	ALG2	NR_024532	chr9	101018527	101024067	-	.	.	.	.	.	.	.	.	.	.	.	.	.	.	.	.	.	.	.	.
1584	2	NR4A3	NM_173200	chr9	101623957	101668994	+	.	.	.	.	.	.	.	.	.	.	.	.	.	.	.	.	.	.	.	.
5485	1	TMEFF1	NM_003692	chr9	102275340	102379739	+	.	.	.	.	.	.	.	.	.	.	.	.	.	.	.	.	.	.	.	.
1274	2	BAAT	NM_001127610	chr9	103162519	103185622	-	.	.	.	.	.	.	.	.	.	.	.	.	.	.	.	.	.	.	.	.
5768	1	ZNF189	NM_003452	chr9	103200983	103212763	+	.	.	.	.	.	.	.	.	.	.	.	.	.	.	.	.	.	.	.	.
1239	2	ALDOB	NM_000035	chr9	103222662	103237883	-	.	.	.	.	.	.	.	.	.	.	.	.	.	.	.	.	.	.	.	.
3970	1	GRIN3A	NM_133445	chr9	103371454	103540683	-	.	.	.	.	.	.	.	.	.	.	.	.	.	.	.	.	.	.	.	.
1675	2	PPP3R2	NM_147180	chr9	103393717	103397104	-	.	.	.	.	.	.	.	.	.	.	.	.	.	.	.	.	.	.	.	.
1764	2	SMC2	NM_006444	chr9	105896361	105943521	+	AY517133.1	chr9	105901679	+	.	.	.	.	.	.	.	.	.	.	.	.	.	.	.	.
199	4,25	ABCA1	NM_005502	chr9	106583104	106730348	-	CL529049	chr9	106593483	-	.	.	.	.	.	.	.	.	.	.	.	.	.	.	.	.
1792	2	TAL2	NM_005421	chr9	107464558	107465206	+	.	.	.	.	.	.	.	.	.	.	.	.	.	.	.	.	.	.	.	.
2742	1,25	RAD23B	NM_002874	chr9	109085337	109134296	+	AY516917.1	chr9	109109533	-	.	.	.	.	.	.	.	.	.	.	.	.	.	.	.	.
2742	1,25	RAD23B	NM_002874	chr9	109085337	109134296	+	CL799878	chr9	109117037	-	.	.	.	.	.	.	.	.	.	.	.	.	.	.	.	.
875	2,5	KLF4	NM_004235	chr9	109286953	109291868	-	.	.	.	.	.	.	.	.	.	.	.	.	.	.	.	.	.	.	.	.
2896	1	ACTL7B	NM_006686	chr9	110656689	110658096	-	.	.	.	.	.	.	.	.	.	.	.	.	.	.	.	.	.	.	.	.
2895	1	ACTL7A	NM_006687	chr9	110664423	110665856	+	.	.	.	.	.	.	.	.	.	.	.	.	.	.	.	.	.	.	.	.
595	3	IKBKAP	NM_003640	chr9	110669620	110736429	-	.	.	.	.	.	.	.	.	.	.	.	.	.	.	.	.	.	.	.	.
3483	1	CTNNAL1	NM_003798	chr9	110744671	110815585	-	.	.	.	.	.	.	.	.	.	.	.	.	.	.	.	.	.	.	.	.
3691	1	EPB41L4B	NM_018424	chr9	111041832	111122842	-	.	.	.	.	.	.	.	.	.	.	.	.	.	.	.	.	.	.	.	.
4999	1	PTPN3	NM_001145369	chr9	111177794	111253485	-	BH609618	chr9	111252082	+	.	.	.	.	.	.	.	.	.	.	.	.	.	.	.	.
4691	1	PALM2-AKAP2	NM_007203	chr9	111582397	111974612	+	.	.	.	.	.	.	.	.	.	.	.	.	.	.	.	.	.	.	.	.
2940	1	AKAP2	NM_001198656	chr9	111850698	111974612	+	.	.	.	.	.	.	.	.	.	.	.	.	.	.	.	.	.	.	.	.
2817	1,25	TXN	NM_001244938	chr9	112045912	112058741	-	.	.	.	.	.	.	.	.	.	.	.	.	.	.	.	.	.	.	.	.
484	3,25	MUSK	NM_001166281	chr9	112470871	112603099	+	.	.	.	.	.	.	.	.	.	.	.	.	.	.	.	.	.	.	.	.
883	2,5	LPAR1	NM_057159	chr9	112675874	112840186	-	.	.	.	.	.	.	.	.	.	.	.	.	.	.	.	.	.	.	.	.
1428	2	GNG10	NM_001198664	chr9	113463671	113472347	+	.	.	.	.	.	.	.	.	.	.	.	.	.	.	.	.	.	.	.	.
2818	1,25	UGCG	NM_003358	chr9	113699026	113735254	+	.	.	.	.	.	.	.	.	.	.	.	.	.	.	.	.	.	.	.	.
5745	1	ZFP37	NM_003408	chr9	114843994	114858817	-	.	.	.	.	.	.	.	.	.	.	.	.	.	.	.	.	.	.	.	.
2404	1,5	SLC31A2	NM_001860	chr9	114953058	114966243	+	.	.	.	.	.	.	.	.	.	.	.	.	.	.	.	.	.	.	.	.
2774	1,25	SLC31A1	NM_001859	chr9	115023628	115066593	+	.	.	.	.	.	.	.	.	.	.	.	.	.	.	.	.	.	.	.	.
4954	1	PRPF4	NM_001244926	chr9	115077734	115095006	+	.	.	.	.	.	.	.	.	.	.	.	.	.	.	.	.	.	.	.	.
6069	0,5	BSPRY	NM_017688	chr9	115151632	115173334	+	.	.	.	.	.	.	.	.	.	.	.	.	.	.	.	.	.	.	.	.
1236	2	ALAD	NM_000031	chr9	115188412	115203439	-	.	.	.	.	.	.	.	.	.	.	.	.	.	.	.	.	.	.	.	.
645	3	POLE3	NR_027261	chr9	115209339	115212776	-	.	.	.	.	.	.	.	.	.	.	.	.	.	.	.	.	.	.	.	.
780	2,5	AMBP	NM_001633	chr9	115862228	115880573	-	.	.	.	.	.	.	.	.	.	.	.	.	.	.	.	.	.	.	.	.
1269	2	ATP6V1G1	NM_004888	chr9	116389814	116400973	+	.	.	.	.	.	.	.	.	.	.	.	.	.	.	.	.	.	.	.	.

Table S2

tumor associated genes								HIV				MLV				MMTV				MMTV(SIN)				MMTV(SIN)arrest			
rank	score	gene symbol	RefSEq	chrom	txStart	txEnd	strand	integrant	chr	position	strand	integrant	chr	position	strand	integrant	chr	position	strand	integrant	chr	position	strand	integrant	chr	position	strand
6116	0,5	C9orf91	NM_153045	chr9	116413526	116448524	+	.	.	.	.	.	.	.	.	.	.	.	.	.	.	.	.	.	.	.	.
2804	1,25	TNFSF8	NM_001252290	chr9	116695443	116732696	-	.	.	.	.	.	.	.	.	.	.	.	.	.	.	.	.	.	.	.	.
434	3,5	TNC	NM_002160	chr9	116821675	116920357	-	.	.	.	.	.	.	.	.	.	.	.	.	.	.	.	.	.	.	.	.
3556	1	DEC1	NM_017418	chr9	116943917	117204744	+	.	.	.	.	.	.	.	.	.	.	.	.	.	.	.	.	.	.	.	.
4696	1	PAPPA	NM_002581	chr9	117955891	118204421	+	.	.	.	.	.	.	.	.	.	.	.	.	.	.	.	.	.	.	.	.
7603	0,25	TRIM32	NM_001099679	chr9	118489401	118503400	+	.	.	.	.	.	.	.	.	.	.	.	.	.	.	.	.	.	.	.	.
968	2,5	TLR4	NM_003266	chr9	119506273	119519590	+	.	.	.	.	.	.	.	.	.	.	.	.	.	.	.	.	.	.	.	.
1028	2,25	DBC1	NM_014618	chr9	120968728	121171560	-	.	.	.	.	.	.	.	.	.	.	.	.	.	.	.	.	.	.	.	.
1399	2	FBXW2	NM_012164	chr9	122559074	122595561	-	CL800370	chr9	122563642	-	.	.	.	.	.	.	.	.	.	.	.	.	.	.	.	.
5021	1	RAB14	NM_016322	chr9	122980235	123004186	-	.	.	.	.	.	.	.	.	.	.	.	.	.	.	.	.	.	.	.	.
583	3	GSN	NM_001127667	chr9	123070200	123134941	+	.	.	.	.	.	.	.	.	.	.	.	.	.	.	.	.	.	.	.	.
4531	1	NDUFA8	NM_014222	chr9	123946158	123961919	-	.	.	.	.	.	.	.	.	.	.	.	.	.	.	.	.	.	.	.	.
4281	1	LHX6	NM_014368	chr9	124004676	124030912	-	.	.	.	.	.	.	.	.	.	.	.	.	.	.	.	.	.	.	.	.
4986	1	PTGS1	NM_000962	chr9	124173049	124197802	+	.	.	.	.	.	.	.	.	.	.	.	.	.	.	.	.	.	.	.	.
5047	1	RABGAP1	NM_012197	chr9	124743108	124906968	+	.	.	.	.	.	.	.	.	.	.	.	.	.	.	.	.	.	.	.	.
4280	1	LHX2	NM_004789	chr9	125813709	125835263	+	.	.	.	.	.	.	.	.	.	.	.	.	.	.	.	.	.	.	.	.
4562	1	NEK6	NM_001145001	chr9	126060016	126154540	+	.	.	.	.	.	.	.	.	.	.	.	.	.	.	.	.	.	.	.	.
1585	2	NR5A1	NM_004959	chr9	126283335	126309520	-	.	.	.	.	.	.	.	.	.	.	.	.	.	.	.	.	.	.	.	.
4617	1	NR6A1	NM_033334	chr9	126324523	126573397	-	.	.	.	.	.	.	.	.	.	.	.	.	.	.	.	.	.	.	.	.
4928	1	PPP6C	NM_001123369	chr9	126948672	126992039	-	.	.	.	.	.	.	.	.	.	.	.	.	.	.	.	.	.	.	.	.
4094	1	HSPA5	NM_005347	chr9	127036947	127043487	-	.	.	.	.	.	.	.	.	.	.	.	.	.	.	.	.	.	.	.	.
1103	2,25	MAPKAP1	NM_001006619	chr9	127239493	127509334	-	.	.	.	.	.	.	.	.	.	.	.	.	.	.	.	.	.	.	.	.
6331	0,5	FAM125B	NM_033446	chr9	128128943	128309141	+	.	.	.	.	.	.	.	.	_257_1_176	chr9	128232945	+	.	.	.	.	.	.	.	.
7114	0,5	ZBTB43	NM_001135776	chr9	128607105	128640308	+	.	.	.	.	.	.	.	.	.	.	.	.	.	.	.	.	.	.	.	.
6792	0,5	RALGPS1	NM_001190730	chr9	128716873	128980631	+	.	.	.	.	.	.	.	.	.	.	.	.	.	.	.	.	.	.	.	.
3857	1	GARNL3	NM_032293	chr9	129066576	129195649	+	.	.	.	.	.	.	.	.	.	.	.	.	.	.	.	.	.	.	.	.
5269	1	SLC2A8	NM_014580	chr9	129199285	129209986	+	.	.	.	.	.	.	.	.	.	.	.	.	.	.	.	.	.	.	.	.
6985	0,5	STXBP1	NM_001032221	chr9	129414306	129494816	+	.	.	.	.	.	.	.	.	.	.	.	.	.	.	.	.	192_87_1	chr9	129457271	-
715	2,75	CDK9	NM_001261	chr9	129588125	129592873	+	.	.	.	.	.	.	.	.	.	.	.	.	.	.	.	.	.	.	.	.
3798	1	FPGS	NM_004957	chr9	129604974	129616377	+	.	.	.	.	.	.	.	.	.	.	.	.	.	.	.	.	.	.	.	.
1902	1,75	ENG	NM_000118	chr9	129617111	129656868	-	.	.	.	.	.	.	.	.	.	.	.	.	.	.	.	.	.	.	.	.
2937	1	AK1	NM_000476	chr9	129668579	129679843	-	.	.	.	.	.	.	.	.	.	.	.	.	.	.	.	.	.	.	.	.
5366	1	ST6GALNAC6	NM_013443	chr9	129687421	129701692	-	.	.	.	.	.	.	.	.	.	.	.	.	.	.	.	.	.	.	.	.
5364	1	ST6GALNAC4	NM_175040	chr9	129709985	129719126	-	.	.	.	.	.	.	.	.	.	.	.	.	.	.	.	.	.	.	.	.
4814	1	PIP5KL1	NM_173492	chr9	129723628	129729591	-	.	.	.	.	.	.	.	.	.	.	.	.	.	.	.	.	.	.	.	.
4983	1	PTGES2	NM_025072	chr9	129922792	129930562	-	.	.	.	.	.	.	.	.	.	.	.	.	.	.	.	.	.	.	.	.
7395	0,25	LCN2	NM_005564	chr9	129951552	129955555	+	.	.	.	.	.	.	.	.	.	.	.	.	.	.	.	.	.	.	.	.
6184	0,5	CIZ1	NM_001257975	chr9	129968164	129994204	-	.	.	.	.	.	.	.	.	.	.	.	.	.	.	.	.	.	.	.	.
814	2,5	DNM1	NM_004408	chr9	130005483	130057348	+	.	.	.	.	.	.	.	.	.	.	.	.	.	.	.	.	.	.	.	.
6390	0,5	GOLGA2	NM_004486	chr9	130057928	130078089	-	.	.	.	.	.	.	.	.	.	.	.	.	.	.	.	.	.	.	.	.
1772	2	SPTAN1	NM_003127	chr9	130354657	130435765	+	BH609620	chr9	130413397	-	.	.	.	.	.	.	.	.	.	.	.	.	.	.	.	.
1772	2	SPTAN1	NM_003127	chr9	130354657	130435765	+	CL799588	chr9	130369975	-	.	.	.	.	.	.	.	.	.	.	.	.	.	.	.	.
1748	2	SET	NM_001122821	chr9	130485754	130498496	+	.	.	.	.	AY516384	chr9	130486325	-	.	.	.	.	.	.	.	.	.	.	.	.
4822	1	PKN3	NM_013355	chr9	130504622	130523020	+	.	.	.	.	.	.	.	.	.	.	.	.	.	.	.	.	.	.	.	.
3604	1	DOLK	NM_014908	chr9	130747629	130749833	-	.	.	.	.	.	.	.	.	.	.	.	.	.	.	.	.	.	.	.	.
4649	1	NUP188	NM_015354	chr9	130749797	130809195	+	CL800233	chr9	130751989	-	.	.	.	.	.	.	.	.	.	.	.	.	.	.	.	.
4649	1	NUP188	NM_015354	chr9	130749797	130809195	+	CL799985	chr9	130766457	-	.	.	.	.	.	.	.	.	.	.	.	.	.	.	.	.
4649	1	NUP188	NM_015354	chr9	130749797	130809195	+	CL799834	chr9	130766032	-	.	.	.	.	.	.	.	.	.	.	.	.	.	.	.	.
4649	1	NUP188	NM_015354	chr9	130749797	130809195	+	CL800493	chr9	130793757	-	.	.	.	.	.	.	.	.	.	.	.	.	.	.	.	.
3455	1	CRAT	NM_001257363	chr9	130896893	130912891	-	.	.	.	.	.	.	.	.	.	.	.	.	.	.	.	.	.	.	.	.
652	3	PPP2R4	NM_178000	chr9	130913413	130951046	+	.	.	.	.	.	.	.	.	.	.	.	.	.	.	.	.	.	.	.	.

Table S2

tumor associated genes								HIV				MLV				MMTV				MMTV(SIN)				MMTV(SIN)arrest			
rank	score	gene symbol	RefSEq	chrom	txStart	txEnd	strand	integrant	chr	position	strand	integrant	chr	position	strand	integrant	chr	position	strand	integrant	chr	position	strand	integrant	chr	position	strand
4114	1	IER5L	NM_203434	chr9	130977651	130980361	-	.	.	.	.	.	.	.	.	.	.	.	.	.	.	.	.	.	.	.	.
1147	2,25	PTGES	NM_004878	chr9	131540435	131555165	-	.	.	.	.	.	.	.	.	.	.	.	.	.	.	.	.	.	.	.	.
5655	1	USP20	NM_001110303	chr9	131637516	131683928	+	.	.	.	.	.	.	.	.	.	.	.	.	1399_73	chr9	131668494	-	.	.	.	.
1409	2	FNBP1	NM_015033	chr9	131689286	131845294	-	CL799949	chr9	131732668	+	.	.	.	.	.	.	.	.	.	.	.	.	.	.	.	.
1409	2	FNBP1	NM_015033	chr9	131689286	131845294	-	CL799820	chr9	131736295	-	.	.	.	.	.	.	.	.	.	.	.	.	.	.	.	.
1409	2	FNBP1	NM_015033	chr9	131689286	131845294	-	CL800567	chr9	131714141	-	.	.	.	.	.	.	.	.	.	.	.	.	.	.	.	.
3058	1	ASS1	NM_054012	chr9	132309914	132366482	+	.	.	.	.	.	.	.	.	.	.	.	.	.	.	.	.	.	.	.	.
52	7	ABL1	NM_005157	chr9	132700651	132752883	+	.	.	.	.	.	.	.	.	.	.	.	.	.	.	.	.	.	.	.	.
4256	1	LAMC3	NM_006059	chr9	132874324	132958267	+	.	.	.	.	.	.	.	.	.	.	.	.	.	.	.	.	.	.	.	.
338	3,75	NUP214	NM_005085	chr9	132990801	133098912	+	.	.	.	.	.	.	.	.	.	.	.	.	99_1_474_19	chr9	133046238	+	.	.	.	.
4890	1	PPAPDC3	NM_032728	chr9	133154901	133174470	+	.	.	.	.	.	.	.	.	.	.	.	.	.	.	.	.	.	.	.	.
5616	1	UCK1	NM_031432	chr9	133389003	133396483	-	.	.	.	.	.	.	.	.	.	.	.	.	.	.	.	.	.	.	.	.
2745	1,25	RAPGEF1	NM_005312	chr9	133441977	133602746	-	.	.	.	.	.	.	.	.	.	.	.	.	.	.	.	.	.	.	.	.
6687	0,5	NTNG2	NM_032536	chr9	134027154	134108041	+	.	.	.	.	.	.	.	.	.	.	.	.	.	.	.	.	.	.	.	.
5216	1	SETX	NM_015046	chr9	134126647	134220193	-	.	.	.	.	.	.	.	.	.	.	.	.	.	.	.	.	.	.	.	.
3990	1	GTF3C4	NM_012204	chr9	134535548	134555291	+	.	.	.	.	.	.	.	.	.	.	.	.	.	.	.	.	.	.	.	.
165	4,75	TSC1	NM_001162426	chr9	134756555	134809841	-	CL529730	chr9	134789365	+	.	.	.	.	.	.	.	.	.	.	.	.	.	.	.	.
3326	1	CEL	NM_001807	chr9	134927185	134937069	+	.	.	.	.	.	.	.	.	.	.	.	.	.	.	.	.	.	.	.	.
1716	2	RALGDS	NM_001042368	chr9	134962927	135014409	-	.	.	.	.	.	.	.	.	.	.	.	.	.	.	.	.	.	.	.	.
3863	1	GBGT1	NM_021996	chr9	135018160	135029122	-	CL799977	chr9	135020932	-	.	.	.	.	.	.	.	.	.	.	.	.	.	.	.	.
2862	1	ABO	NM_020469	chr9	135120383	135140451	-	CL800273	chr9	135130929	+	.	.	.	.	.	.	.	.	.	.	.	.	.	.	.	.
2565	1,25	DBH	NM_000787	chr9	135491305	135514287	+	.	.	.	.	.	.	.	.	.	.	.	.	.	.	.	.	.	.	.	.
5181	1	SARDH	NM_001134707	chr9	135518504	135593300	-	.	.	.	.	.	.	.	.	.	.	.	.	.	.	.	.	.	.	.	.
2456	1,5	VAV2	NM_003371	chr9	135616836	135847267	-	.	.	.	.	.	.	.	.	.	.	.	.	.	.	.	.	.	.	.	.
3161	1	BRD3	NM_007371	chr9	135885266	135922962	-	.	.	.	.	.	.	.	.	.	.	.	.	.	.	.	.	.	.	.	.
2759	1,25	RXRA	NM_002957	chr9	136358136	136472252	+	.	.	.	.	.	.	.	.	.	.	.	.	101_1_609_22	chr9	136405790	-	.	.	.	.
3417	1	COL5A1	NM_000093	chr9	136673472	136876509	+	.	.	.	.	.	.	.	.	.	.	.	.	.	.	.	.	.	.	.	.
6699	0,5	OLFM1	NM_014279	chr9	137106909	137152851	+	.	.	.	.	.	.	.	.	.	.	.	.	.	.	.	.	.	.	.	.
4269	1	LCN9	NM_001001676	chr9	137694988	137697576	+	.	.	.	.	.	.	.	.	.	.	.	.	.	.	.	.	.	.	.	.
6952	0,5	SOHLH1	NM_001101677	chr9	137725075	137731195	-	.	.	.	.	.	.	.	.	.	.	.	.	.	.	.	.	.	.	.	.
6862	0,5	SDCCAG3	NM_001039708	chr9	138416194	138424875	-	CL799986	chr9	138422345	+	.	.	.	.	.	.	.	.	.	.	.	.	.	.	.	.
603	3	INPP5E	NM_019892	chr9	138442887	138454077	-	.	.	.	.	.	.	.	.	.	.	.	.	.	.	.	.	.	.	.	.
43	7,75	NOTCH1	NM_017617	chr9	138508716	138560059	-	CL800450	chr9	138520429	-	.	.	.	.	.	.	.	.	.	.	.	.	.	.	.	.
1229	2	AGPAT2	NM_001012727	chr9	138687415	138701732	-	CL800308	chr9	138697270	-	.	.	.	.	.	.	.	.	.	.	.	.	.	.	.	.
1616	2	PHPT1	NM_014172	chr9	138863076	138865311	+	.	.	.	.	.	.	.	.	.	.	.	.	.	.	.	.	.	.	.	.
4334	1	MAMDC4	NM_206920	chr9	138866639	138875072	+	.	.	.	.	.	.	.	.	.	.	.	.	.	.	.	.	.	.	.	.
3645	1	EDF1	NM_153200	chr9	138877053	138880559	-	.	.	.	.	.	.	.	.	.	.	.	.	.	.	.	.	.	.	.	.
976	2,5	TRAF2	NM_021138	chr9	138900785	138940888	+	CL799614	chr9	138907897	+	.	.	.	.	.	.	.	.	.	.	.	.	.	.	.	.
6552	0,5	LCN12	NM_178536	chr9	138966588	138969770	+	.	.	.	.	.	.	.	.	.	.	.	.	.	.	.	.	.	.	.	.
6781	0,5	PTGDS	NM_000954	chr9	138991776	138996015	+	.	.	.	.	.	.	.	.	.	.	.	.	.	.	.	.	.	.	.	.
2484	1,25	ABCA2	NM_212533	chr9	139021506	139043195	-	.	.	.	.	.	.	.	.	.	.	.	.	.	.	.	.	.	.	.	.
3822	1	FUT7	NM_004479	chr9	139044446	139047113	-	.	.	.	.	.	.	.	.	.	.	.	.	.	.	.	.	.	.	.	.
3687	1	ENTPD2	NM_001246	chr9	139062371	139068324	-	.	.	.	.	.	.	.	.	.	.	.	.	.	.	.	.	.	.	.	.
4338	1	MAN1B1	NM_016219	chr9	139101199	139123460	+	BH609622	chr9	139102722	-	.	.	.	.	.	.	.	.	.	.	.	.	.	.	.	.
7289	0,25	DPP7	NM_013379	chr9	139124812	139129016	-	.	.	.	.	.	.	.	.	.	.	.	.	.	.	.	.	.	.	.	.
6411	0,5	GRIN1	NM_007327	chr9	139153429	139183035	+	.	.	.	.	.	.	.	.	.	.	.	.	.	.	.	.	.	.	.	.
444	3,25	ANAPC2	NM_013366	chr9	139189056	139202878	-	.	.	.	.	.	.	.	.	.	.	.	.	.	.	.	.	.	.	.	.
3690	1	ENTPD8	NM_001033113	chr9	139448636	139455722	-	.	.	.	.	.	.	.	.	.	.	.	.	.	.	.	.	.	.	.	.
6628	0,5	MRPL41	NM_032477	chr9	139566129	139566828	+	.	.	.	.	.	.	.	.	.	.	.	.	.	.	.	.	.	.	.	.
1377	2	EHMT1	NM_001145527	chr9	139633264	139798666	+	CL529247	chr9	139697074	+	.	.	.	.	.	.	.	.	.	.	.	.	.	.	.	.
1377	2	EHMT1	NM_001145527	chr9	139633264	139798666	+	CL529337	chr9	139675479	-	.	.	.	.	.	.	.	.	.	.	.	.	.	.	.	.

Table S2

tumor associated genes								HIV				MLV				MMTV				MMTV(SIN)				MMTV(SIN)arrest			
rank	score	gene symbol	RefSEq	chrom	txStart	txEnd	strand	integrant	chr	position	strand	integrant	chr	position	strand	integrant	chr	position	strand	integrant	chr	position	strand	integrant	chr	position	strand
3259	1	CACNA1B	NM_001243812	chr9	139892061	140138897	+	.	.	.	.	.	.	.	.	.	.	.	.	.	.	.	.	.	.	.	.
5754	1	ZMYND11	NM_001202468	chr10	215933	285729	+	.	.	.	.	.	.	.	.	.	.	.	.	.	.	.	.	.	.	.	.
1358	2	DIP2C	NM_014974	chr10	310129	725608	-	.	.	.	.	.	.	.	.	.	.	.	.	73_1_2760_19	chr10	516695	+	.	.	.	.
1437	2	GTPBP4	NM_012341	chr10	1024348	1053708	+	.	.	.	.	.	.	.	.	.	.	.	.	.	.	.	.	.	.	.	.
1462	2	IDI2	NM_033261	chr10	1054846	1061799	-	.	.	.	.	.	.	.	.	.	.	.	.	.	.	.	.	.	.	.	.
1461	2	IDI1	NM_004508	chr10	1075963	1085061	-	.	.	.	.	.	.	.	.	.	.	.	.	.	.	.	.	.	.	.	.
5693	1	WDR37	NM_014023	chr10	1092775	1168237	+	.	.	.	.	.	.	.	.	.	.	.	.	.	.	.	.	.	.	.	.
1989	1,5	ADARB2	NM_018702	chr10	1213252	1769670	-	.	.	.	.	.	.	.	.	.	.	.	.	.	.	.	.	.	.	.	.
638	3	PFKP	NM_001242339	chr10	3100818	3168997	+	.	.	.	.	.	.	.	.	.	.	.	.	.	.	.	.	.	.	.	.
217	4,25	KLF6	NM_001300	chr10	3808187	3817473	-	.	.	.	.	.	.	.	.	.	.	.	.	.	.	.	.	.	.	.	.
778	2,5	AKR1C1	NM_001353	chr10	4995453	5010158	+	.	.	.	.	.	.	.	.	.	.	.	.	.	.	.	.	.	.	.	.
987	2,25	AKR1C2	NM_001354	chr10	5019967	5050225	-	.	.	.	.	.	.	.	.	.	.	.	.	.	.	.	.	.	.	.	.
526	3	AKR1C3	NM_003739	chr10	5126567	5139878	+	.	.	.	.	.	.	.	.	.	.	.	.	.	.	.	.	.	.	.	.
1234	2	AKR1C4	NM_001818	chr10	5228797	5250910	+	.	.	.	.	.	.	.	.	.	.	.	.	.	.	.	.	.	.	.	.
6663	0,5	NET1	NM_001047160	chr10	5444513	5491019	+	CL800845	chr10	5456512	-	.	.	.	.	.	.	.	.	.	.	.	.	.	.	.	.
6470	0,5	IL15RA	NM_172200	chr10	6034339	6059580	-	.	.	.	.	.	.	.	.	.	.	.	.	.	.	.	.	.	.	.	.
332	3,75	IL2RA	NM_000417	chr10	6092662	6144339	-	.	.	.	.	.	.	.	.	.	.	.	.	.	.	.	.	.	.	.	.
4759	1	PFKFB3	NM_001145443	chr10	6226848	6317513	+	.	.	.	.	.	.	.	.	.	.	.	.	.	.	.	.	.	.	.	.
1143	2,25	PRKCQ	NM_006257	chr10	6509110	6662260	-	CL800615	chr10	6582503	+	.	.	.	.	.	.	.	.	.	.	.	.	.	.	.	.
2633	1,25	KIN	NM_012311	chr10	7832930	7869996	-	.	.	.	.	.	.	.	.	.	.	.	.	.	.	.	.	.	.	.	.
3073	1	ATP5C1	NM_001001973	chr10	7870098	7889768	+	.	.	.	.	.	.	.	.	.	.	.	.	.	.	.	.	.	.	.	.
842	2,5	GATA3	NM_002051	chr10	8136672	8157170	+	CL529491	chr10	8152329	-	.	.	.	.	.	.	.	.	.	.	.	.	.	.	.	.
7614	0,25	USP6NL	NM_014688	chr10	11542514	11693685	-	.	.	.	.	.	.	.	.	.	.	.	.	.	.	.	.	941_125_2	chr10	11658549	-
6295	0,5	ECHDC3	NM_024693	chr10	11824361	11846071	+	.	.	.	.	.	.	.	.	.	.	.	.	.	.	.	.	.	.	.	.
1842	2	UPF2	NM_080599	chr10	12002026	12125175	-	.	.	.	.	.	.	.	.	.	.	.	.	.	.	.	.	.	.	.	.
2105	1,5	DHTKD1	NM_018706	chr10	12150939	12205230	+	.	.	.	.	.	.	.	.	.	.	.	.	.	.	.	.	.	.	.	.
2391	1,5	SEC61A2	NR_024577	chr10	12211645	12251963	+	.	.	.	.	.	.	.	.	.	.	.	.	.	.	.	.	.	.	.	.
4646	1	NUDT5	NM_014142	chr10	12249578	12278149	-	.	.	.	.	.	.	.	.	.	.	.	.	.	.	.	.	.	.	.	.
6158	0,5	CDC123	NM_006023	chr10	12277966	12332595	+	.	.	.	.	.	.	.	.	.	.	.	.	.	.	.	.	.	.	.	.
2048	1,5	CAMK1D	NM_020397	chr10	12431588	12908143	+	.	.	.	.	.	.	.	.	.	.	.	.	393_118	chr10	12550031	+	1929_172_1	chr10	12588125	-
6140	0,5	CCDC3	NM_031455	chr10	12978630	13083710	-	.	.	.	.	.	.	.	.	.	.	.	.	.	.	.	.	.	.	.	.
2668	1,25	MCM10	NM_182751	chr10	13243559	13293110	+	.	.	.	.	.	.	.	.	.	.	.	.	.	.	.	.	.	.	.	.
2764	1,25	SEPHS1	NM_012247	chr10	13399443	13430304	-	.	.	.	.	.	.	.	.	.	.	.	.	.	.	.	.	.	.	.	.
4953	1	PRPF18	NM_003675	chr10	13668944	13712874	+	.	.	.	.	.	.	.	.	.	.	.	.	.	.	.	.	.	.	.	.
508	3,25	SUV39H2	NM_001193424	chr10	14960787	14986310	+	.	.	.	.	.	.	.	.	.	.	.	.	.	.	.	.	.	.	.	.
3539	1	DCLRE1C	NM_001033858	chr10	14988876	15036100	-	.	.	.	.	.	.	.	.	.	.	.	.	.	.	.	.	.	.	.	.
4661	1	OLAH	NM_001039702	chr10	15125900	15155857	+	.	.	.	.	.	.	.	.	.	.	.	.	.	.	.	.	.	.	.	.
869	2,5	ITGA8	NM_003638	chr10	15599093	15801776	-	.	.	.	.	.	.	.	.	.	.	.	.	.	.	.	.	.	.	.	.
6086	0,5	C1QL3	NM_001010908	chr10	16595747	16604010	-	.	.	.	.	.	.	.	.	.	.	.	.	.	.	.	.	.	.	.	.
7522	0,25	RSU1	NM_012425	chr10	16672622	16899459	-	.	.	.	.	.	.	.	.	.	.	.	.	.	.	.	.	.	.	.	.
1979	1,75	VIM	NM_003380	chr10	17310263	17319598	+	.	.	.	.	.	.	.	.	.	.	.	.	.	.	.	.	.	.	.	.
4990	1	PTPLA	NM_014241	chr10	17671963	17699379	-	.	.	.	.	.	.	.	.	.	.	.	.	.	.	.	.	.	.	.	.
5370	1	STAM	NM_003473	chr10	17726129	17798827	+	.	.	.	.	.	.	.	.	.	.	.	.	.	.	.	.	.	.	.	.
3264	1	CACNB2	NM_201571	chr10	18469747	18870694	+	.	.	.	.	.	.	.	.	.	.	.	.	.	.	.	.	3308_352_1	chr10	18769066	+
3037	1	ARL5B	NM_178815	chr10	18988318	19006946	+	.	.	.	.	.	.	.	.	.	.	.	.	.	.	.	.	.	.	.	.
4848	1	PLXDC2	NM_032812	chr10	20145377	20609121	+	CL529058	chr10	20186325	-	.	.	.	.	.	.	.	.	43_1_1933_6	chr10	20213614	+	.	.	.	.
6657	0,5	NEBL	NM_006393	chr10	21108908	21226537	-	.	.	.	.	.	.	.	.	.	.	.	.	.	.	.	.	.	.	.	.
1537	2	MLLT10	NM_001195627	chr10	21863106	21886120	+	CL529528	chr10	21974092	-	.	.	.	.	.	.	.	.	.	.	.	.	.	.	.	.
2515	1,25	BMI1	NM_005180	chr10	22650144	22660420	+	.	.	.	.	.	.	.	.	.	.	.	.	.	.	.	.	.	.	.	.
492	3,25	PIP4K2A	NM_005028	chr10	22863771	23043509	-	.	.	.	.	.	.	.	.	.	.	.	.	.	.	.	.	.	.	.	.
6632	0,5	MSRB2	NM_012228	chr10	23424432	23450948	+	.	.	.	.	.	.	.	.	.	.	.	.	.	.	.	.	.	.	.	.

Table S2

tumor associated genes								HIV				MLV				MMTV				MMTV(SIN)				MMTV(SIN)arrest			
rank	score	gene symbol	RefSEq	chrom	txStart	txEnd	strand	integrant	chr	position	strand	integrant	chr	position	strand	integrant	chr	position	strand	integrant	chr	position	strand	integrant	chr	position	strand
6523	0,5	KIAA1217	NM_001098500	chr10	24023680	24876778	+	.	.	.	.	.	.	.	.	.	.	.	.	.	.	.	.	.	.	.	.
4963	1	PRTFDC1	NM_020200	chr10	25177559	25281539	-	.	.	.	.	.	.	.	.	.	.	.	.	.	.	.	.	.	.	.	.
848	2,5	GPR158	NM_020752	chr10	25504295	25931163	+	.	.	.	.	.	.	.	.	.	.	.	.	.	.	.	.	.	.	.	.
2267	1,5	MYO3A	NM_017433	chr10	26263007	26541471	+	.	.	.	.	.	.	.	.	.	.	.	.	.	.	.	.	.	.	.	.
1910	1,75	GAD2	NM_000818	chr10	26545241	26633497	+	.	.	.	.	.	.	.	.	.	.	.	.	.	.	.	.	.	.	.	.
784	2,5	APBB1IP	NM_019043	chr10	26767271	26896738	+	CL529674	chr10	26785058	+	.	.	.	.	.	.	.	.	.	.	.	.	.	.	.	.
706	2,75	ABI1	NM_001178124	chr10	27075530	27190022	-	CL529059	chr10	27141211	+	.	.	.	.	.	.	.	.	.	.	.	.	.	.	.	.
4362	1	MASTL	NM_001172303	chr10	27483758	27515854	+	.	.	.	.	.	.	.	.	.	.	.	.	.	.	.	.	.	.	.	.
5022	1	RAB18	NM_001256412	chr10	27833108	27871172	+	CL529339	chr10	27869329	+	.	.	.	.	.	.	.	.	.	.	.	.	.	.	.	.
6624	0,5	MPP7	NM_173496	chr10	28379928	28611073	-	.	.	.	.	AY516153	chr10	28530862	+	.	.	.	.	.	.	.	.	.	.	.	.
6049	0,5	BAMBI	NM_012342	chr10	29006429	29011874	+	.	.	.	.	.	.	.	.	.	.	.	.	.	.	.	.	.	.	.	.
4323	1	LYZL1	NM_032517	chr10	29617995	29640164	+	.	.	.	.	.	.	.	.	.	.	.	.	.	.	.	.	.	.	.	.
5416	1	SVIL	NM_021738	chr10	29786282	29963907	-	.	.	.	.	.	.	.	.	.	.	.	.	.	.	.	.	.	.	.	.
1100	2,25	MAP3K8	NM_005204	chr10	30762955	30790768	+	CL529555	chr10	30772700	-	.	.	.	.	.	.	.	.	.	.	.	.	.	.	.	.
2468	1,5	ZEB1	NM_001174093	chr10	31648106	31858748	+	CL800581	chr10	31828823	-	.	.	.	.	.	.	.	.	.	.	.	.	.	.	.	.
6314	0,5	EPC1	NM_025209	chr10	32597864	32676119	-	.	.	.	.	.	.	.	.	.	.	.	.	.	.	.	.	.	.	.	.
1085	2,25	ITGB1	NM_133376	chr10	33229251	33286828	-	.	.	.	.	.	.	.	.	.	.	.	.	.	.	.	.	.	.	.	.
630	3	NRP1	NM_001024628	chr10	33526552	33663839	-	.	.	.	.	.	.	.	.	.	.	.	.	.	.	.	.	.	.	.	.
4699	1	PARD3	NM_001184794	chr10	34641193	35144259	-	BH609493	chr10	35106065	-	.	.	.	.	.	.	.	.	.	.	.	.	.	.	.	.
3893	1	GJD4	NM_153368	chr10	35934343	35937869	+	.	.	.	.	.	.	.	.	.	.	.	.	.	.	.	.	.	.	.	.
1048	2,25	FZD8	NM_031866	chr10	35967182	35970368	-	.	.	.	.	.	.	.	.	.	.	.	.	.	.	.	.	.	.	.	.
7129	0,5	ZNF248	NM_001267606	chr10	38157698	38186570	-	.	.	.	.	.	.	.	.	.	.	.	.	.	.	.	.	.	.	.	.
5789	1	ZNF33A	NM_006954	chr10	38339583	38389001	+	.	.	.	.	.	.	.	.	.	.	.	.	.	.	.	.	.	.	.	.
5792	1	ZNF37A	NM_003421	chr10	38423269	38452284	+	.	.	.	.	.	.	.	.	.	.	.	.	.	.	.	.	.	.	.	.
6062	0,5	BMS1	NM_014753	chr10	42597959	42650391	+	.	.	.	.	.	.	.	.	.	.	.	.	.	.	.	.	.	.	.	.
35	8,25	RET	NM_020975	chr10	42892522	42945803	+	.	.	.	.	.	.	.	.	.	.	.	.	.	.	.	.	.	.	.	.
3468	1	CSGALNACT2	NM_018590	chr10	42953939	43000755	+	CL799583	chr10	42967078	+	.	.	.	.	.	.	.	.	.	.	.	.	.	.	.	.
6797	0,5	RASGEF1A	NM_145313	chr10	43009989	43082373	-	.	.	.	.	.	.	.	.	.	.	.	.	.	.	.	.	.	.	.	.
2560	1,25	CXCL12	NM_199168	chr10	44192515	44200551	-	.	.	.	.	.	.	.	.	.	.	.	.	.	.	.	.	.	.	.	.
1995	1,5	ALOX5	NM_001256154	chr10	45189629	45261573	+	.	.	.	.	.	.	.	.	.	.	.	.	.	.	.	.	.	.	.	.
4995	1	PTPN20A	NM_001042395	chr10	45970128	46061009	-	.	.	.	.	.	.	.	.	.	.	.	.	.	.	.	.	.	.	.	.
2994	1	ANXA8	NM_001040084	chr10	47875230	47891374	+	.	.	.	.	.	.	.	.	.	.	.	.	.	.	.	.	.	.	.	.
5913	0,75	GDF10	NM_004962	chr10	48045793	48059144	-	.	.	.	.	.	.	.	.	.	.	.	.	.	.	.	.	.	.	.	.
4996	1	PTPN20B	NM_001042358	chr10	48357047	48447587	-	.	.	.	.	.	.	.	.	.	.	.	.	.	.	.	.	.	.	.	.
6362	0,5	FRMPD2	NM_001018071	chr10	49034607	49152947	-	.	.	.	.	.	.	.	.	.	.	.	.	.	.	.	.	.	.	.	.
617	3	MAPK8	NM_002750	chr10	49279692	49313189	+	.	.	.	.	.	.	.	.	.	.	.	.	.	.	.	.	.	.	.	.
176	4,5	ERCC6	NM_000124	chr10	50334496	50417153	-	.	.	.	.	.	.	.	.	.	.	.	.	.	.	.	.	.	.	.	.
451	3,25	CHAT	NM_020984	chr10	50487146	50543156	+	.	.	.	.	.	.	.	.	.	.	.	.	.	.	.	.	.	.	.	.
2298	1,5	OGDHL	NM_018245	chr10	50612692	50640431	-	.	.	.	.	.	.	.	.	.	.	.	.	.	.	.	.	.	.	.	.
7462	0,25	PARG	NM_003631	chr10	50696330	51041337	-	.	.	.	.	.	.	.	.	.	.	.	.	.	.	.	.	.	.	.	.
486	3,25	NCOA4	NM_001145263	chr10	51235113	51260740	+	.	.	.	.	.	.	.	.	.	.	.	.	.	.	.	.	.	.	.	.
5225	1	SGMS1	NM_147156	chr10	51735350	52053743	-	AY516920.1	chr10	51828799	+	AY516351	chr10	52034567	-	.	.	.	.	.	.	.	.	.	.	.	.
4944	1	PRKG1	NM_006258	chr10	52504239	53728116	+	.	.	.	.	AY516604	chr10	52592798	-	.	.	.	.	.	.	.	.	3293_346_2	chr10	53040609	-
4944	1	PRKG1	NM_006258	chr10	52504239	53728116	+	.	.	.	.	AY516604	chr10	52592798	-	.	.	.	.	.	.	.	.	645_114_2	chr10	52835351	-
1899	1,75	DKK1	NM_012242	chr10	53744046	53747423	+	.	.	.	.	.	.	.	.	.	.	.	.	.	.	.	.	.	.	.	.
4706	1	PCDH15	NM_001142767	chr10	55250865	56231057	-	AY517319.1	chr10	55352213	-	AY516618	chr10	56017174	-	.	.	.	.	.	.	.	.	.	.	.	.
2483	1,5	ZWINT	NM_001005413	chr10	57787204	57791040	-	.	.	.	.	.	.	.	.	.	.	.	.	.	.	.	.	.	.	.	.
1475	2	IPMK	NM_152230	chr10	59621283	59697700	-	CL529470	chr10	59624467	+	.	.	.	.	.	.	.	.	.	.	.	.	.	.	.	.
5598	1	UBE2D1	NM_003338	chr10	59764744	59800519	+	.	.	.	.	.	.	.	.	.	.	.	.	.	.	.	.	.	.	.	.
1803	2	TFAM	NM_003201	chr10	59814908	59828996	+	.	.	.	.	.	.	.	.	.	.	.	.	.	.	.	.	.	.	.	.
1292	2	CCDC6	NM_005436	chr10	61218511	61336420	-	.	.	.	.	.	.	.	.	.	.	.	.	.	.	.	.	.	.	.	.

Table S2

tumor associated genes								HIV				MLV				MMTV				MMTV(SIN)				MMTV(SIN)arrest			
rank	score	gene symbol	RefSEq	chrom	txStart	txEnd	strand	integrant	chr	position	strand	integrant	chr	position	strand	integrant	chr	position	strand	integrant	chr	position	strand	integrant	chr	position	strand
6010	0,5	ANK3	NM_001149	chr10	61456061	61570780	-	CL529341	chr10	62067129	-	AY516587	chr10	61854181	+	.	.	.	.	.	.	.	.	.	.	.	.
6010	0,5	ANK3	NM_001149	chr10	61456061	61570780	-	CL529061	chr10	62122677	+	AY516587	chr10	61854181	+	.	.	.	.	.	.	.	.	.	.	.	.
7132	0,5	ZNF365	NM_199450	chr10	63803921	63910054	+	.	.	.	.	.	.	.	.	.	.	.	.	.	.	.	.	.	.	.	.
2120	1,5	EGR2	NM_000399	chr10	64241761	64246132	-	.	.	.	.	.	.	.	.	.	.	.	.	.	.	.	.	.	.	.	.
4188	1	JMJD1C	NM_004241	chr10	64596993	64698988	-	.	.	.	.	.	.	.	.	.	.	.	.	.	.	.	.	.	.	.	.
1331	2	CTNNA3	NM_001127384	chr10	67349730	69095422	-	.	.	.	.	.	.	.	.	.	.	.	.	.	.	.	.	.	.	.	.
428	3,5	SIRT1	NM_012238	chr10	69314432	69348153	+	.	.	.	.	.	.	.	.	.	.	.	.	.	.	.	.	.	.	.	.
6039	0,5	ATOH7	NM_145178	chr10	69660357	69661876	-	.	.	.	.	.	.	.	.	.	.	.	.	.	.	.	.	.	.	.	.
6440	0,5	HNRNPH3	NM_012207	chr10	69761773	69772959	+	.	.	.	.	.	.	.	.	.	.	.	.	.	.	.	.	.	.	.	.
6838	0,5	RUFY2	NM_017987	chr10	69773280	69836952	-	.	.	.	.	.	.	.	.	.	.	.	.	.	.	.	.	.	.	.	.
3594	1	DNA2	NM_001080449	chr10	69843826	69901736	-	.	.	.	.	.	.	.	.	.	.	.	.	.	.	.	.	.	.	.	.
1801	2	TET1	NM_030625	chr10	69990122	70124245	+	.	.	.	.	.	.	.	.	.	.	.	.	.	.	.	.	.	.	.	.
3551	1	DDX50	NM_024045	chr10	70331039	70376609	+	.	.	.	.	.	.	.	.	.	.	.	.	.	.	.	.	.	.	.	.
5969	0,75	SRGN	NM_002727	chr10	70517833	70534573	+	.	.	.	.	.	.	.	.	.	.	.	.	.	.	.	.	.	.	.	.
1450	2	HK1	NM_033498	chr10	70699761	70831643	+	.	.	.	.	.	.	.	.	.	.	.	.	.	.	.	.	1940_173_1	chr10	70710902	+
5425	1	TACR2	NM_001057	chr10	70833963	70846680	-	.	.	.	.	.	.	.	.	.	.	.	.	.	.	.	.	.	.	.	.
5571	1	TSPAN15	NM_012339	chr10	70881231	70937429	+	.	.	.	.	.	.	.	.	.	.	.	.	.	.	.	.	.	.	.	.
6074	0,5	C10orf35	NM_145306	chr10	71060008	71063361	+	.	.	.	.	.	.	.	.	.	.	.	.	.	.	.	.	.	.	.	.
3999	1	H2AFY2	NM_018649	chr10	71482362	71542046	+	.	.	.	.	.	.	.	.	.	.	.	.	.	.	.	.	.	.	.	.
2386	1,5	SAR1A	NM_001142648	chr10	71579966	71600291	-	.	.	.	.	.	.	.	.	.	.	.	.	.	.	.	.	.	.	.	.
1954	1,75	PPA1	NM_021129	chr10	71632591	71663196	-	.	.	.	.	.	.	.	.	.	.	.	.	.	.	.	.	.	.	.	.
907	2,5	NODAL	NM_018055	chr10	71861697	71871471	-	.	.	.	.	.	.	.	.	.	.	.	.	.	.	.	.	.	.	.	.
6767	0,5	PRF1	NM_005041	chr10	72027109	72032537	-	.	.	.	.	.	.	.	.	.	.	.	.	.	.	.	.	.	.	.	.
6075	0,5	C10orf54	NM_022153	chr10	73177318	73203343	-	.	.	.	.	.	.	.	.	.	.	.	.	.	.	.	.	.	.	.	.
3310	1	CDH23	NM_001171935	chr10	73241645	73245710	+	.	.	.	.	.	.	.	.	.	.	.	.	.	.	.	.	.	.	.	.
2074	1,5	CHST3	NM_004273	chr10	73394125	73443328	+	.	.	.	.	.	.	.	.	.	.	.	.	.	.	.	.	.	.	.	.
1346	2	DDIT4	NM_019058	chr10	73703682	73705803	+	.	.	.	.	.	.	.	.	.	.	.	.	.	.	.	.	.	.	.	.
4827	1	PLA2G12B	NM_032562	chr10	74364943	74384516	-	.	.	.	.	.	.	.	.	.	.	.	.	.	.	.	.	.	.	.	.
4681	1	P4HA1	NM_001017962	chr10	74436985	74526738	-	CL529065	chr10	74502721	+	.	.	.	.	.	.	.	.	.	.	.	.	.	.	.	.
2993	1	ANXA7	NM_004034	chr10	74805194	74843847	-	.	.	.	.	.	.	.	.	.	.	.	.	.	.	.	.	.	.	.	.
654	3	PPP3CB	NM_001142354	chr10	74866568	74925788	-	CL800619	chr10	74879311	-	.	.	.	.	.	.	.	.	.	.	.	.	.	.	.	.
7082	0,5	USP54	NM_152586	chr10	74927301	75005439	-	.	.	.	.	.	.	.	.	.	.	.	.	.	.	.	.	.	.	.	.
3816	1	FUT11	NM_173540	chr10	75202054	75205982	+	.	.	.	.	.	.	.	.	.	.	.	.	.	.	.	.	.	.	.	.
4520	1	NDST2	NM_003635	chr10	75231674	75241595	-	.	.	.	.	.	.	.	.	.	.	.	.	.	.	.	.	.	.	.	.
800	2,5	CAMK2G	NM_172170	chr10	75242264	75304355	-	.	.	.	.	.	.	.	.	.	.	.	.	.	.	.	.	.	.	.	.
1135	2,25	PLAU	NM_002658	chr10	75340867	75347264	+	.	.	.	.	.	.	.	.	.	.	.	.	.	.	.	.	.	.	.	.
7617	0,25	VCL	NM_003373	chr10	75427877	75549920	+	.	.	.	.	.	.	.	.	.	.	.	.	.	.	.	.	.	.	.	.
1225	2	ADK	NM_001202450	chr10	75580948	76139068	+	AY517340.1	chr10	75759033	+	.	.	.	.	.	.	.	.	.	.	.	.	.	.	.	.
3616	1	DUPD1	NM_001003892	chr10	76467599	76488278	-	.	.	.	.	.	.	.	.	.	.	.	.	.	.	.	.	.	.	.	.
3618	1	DUSP13	NM_001007272	chr10	76524195	76538976	-	.	.	.	.	.	.	.	.	.	.	.	.	.	.	.	.	.	.	.	.
6503	0,5	KCNMA1	NM_002247	chr10	78314640	79067583	-	AY517332.1	chr10	79027424	-	.	.	.	.	.	.	.	.	.	.	.	.	.	.	.	.
1656	2	POLR3A	NM_007055	chr10	79404912	79459304	-	.	.	.	.	.	.	.	.	.	.	.	.	.	.	.	.	.	.	.	.
7542	0,25	SFTPA1	NM_001164645	chr10	81360646	81365151	+	.	.	.	.	.	.	.	.	.	.	.	.	.	.	.	.	.	.	.	.
4363	1	MAT1A	NM_000429	chr10	82021555	82039414	-	.	.	.	.	.	.	.	.	.	.	.	.	.	.	.	.	.	.	.	.
5570	1	TSPAN14	NM_030927	chr10	82204017	82272371	+	.	.	.	.	.	.	.	.	.	.	.	.	.	.	.	.	.	.	.	.
6684	0,5	NRG3	NM_001165973	chr10	83627422	84736915	+	.	.	.	.	.	.	.	.	.	.	.	.	.	.	.	.	.	.	.	.
6383	0,5	GHITM	NM_014394	chr10	85889164	85903291	+	.	.	.	.	.	.	.	.	.	.	.	.	.	.	.	.	.	.	.	.
3968	1	GRID1	NM_017551	chr10	87349291	88116230	-	.	.	.	.	AY516128	chr10	87392838	-	.	.	.	.	.	.	.	.	2839_255_1	chr10	87727671	-
6703	0,5	OPN4	NM_001030015	chr10	88404293	88416196	+	.	.	.	.	.	.	.	.	.	.	.	.	.	.	.	.	.	.	.	.
318	3,75	BMPR1A	NM_004329	chr10	88506375	88674925	+	.	.	.	.	.	.	.	.	.	.	.	.	.	.	.	.	.	.	.	.
305	4	SNCG	NM_003087	chr10	88708267	88712997	+	.	.	.	.	.	.	.	.	.	.	.	.	.	.	.	.	.	.	.	.

Table S2

tumor associated genes								HIV				MLV				MMTV				MMTV(SIN)				MMTV(SIN)arrest			
rank	score	gene symbol	RefSEq	chrom	txStart	txEnd	strand	integrant	chr	position	strand	integrant	chr	position	strand	integrant	chr	position	strand	integrant	chr	position	strand	integrant	chr	position	strand
3166	1	C10orf116	NM_006829	chr10	88718167	88720646	+	.	.	.	.	.	.	.	.	.	.	.	.	.	.	.	.	.	.	.	.
3906	1	GLUD1	NM_005271	chr10	88799938	88844756	-	.	.	.	.	.	.	.	.	.	.	.	.	.	.	.	.	.	.	.	.
277	4	MINPP1	NM_001178117	chr10	89254202	89303198	+	.	.	.	.	.	.	.	.	.	.	.	.	.	.	.	.	.	.	.	.
912	2,5	PAPSS2	NM_001015880	chr10	89409455	89497442	+	.	.	.	.	.	.	.	.	.	.	.	.	.	.	.	.	.	.	.	.
2505	1,25	ATAD1	NM_032810	chr10	89502854	89567897	-	CL800582	chr10	89526877	+	.	.	.	.	.	.	.	.	.	.	.	.	.	.	.	.
2	16,75	PTEN	NM_000314	chr10	89613174	89718512	+	CL529637	chr10	89679993	+	.	.	.	.	.	.	.	.	.	.	.	.	.	.	.	.
5144	1	RNLS	NM_001031709	chr10	90033838	90333062	-	.	.	.	.	.	.	.	.	.	.	.	.	.	.	.	.	.	.	.	.
5371	1	STAMBPL1	NM_020799	chr10	90629923	90673239	+	.	.	.	.	.	.	.	.	.	.	.	.	.	.	.	.	.	.	.	.
78	6,25	FAS	NM_152872	chr10	90740267	90765522	+	CL529740	chr10	90756728	-	.	.	.	.	.	.	.	.	.	.	.	.	.	.	.	.
6171	0,5	CH25H	NM_003956	chr10	90955673	90957051	-	.	.	.	.	.	.	.	.	.	.	.	.	.	.	.	.	.	.	.	.
4287	1	LIPA	NM_000235	chr10	90963309	91001640	-	.	.	.	.	.	.	.	.	.	.	.	.	.	.	.	.	.	.	.	.
6464	0,5	IFIT1	NM_001270928	chr10	91142282	91156224	+	.	.	.	.	.	.	.	.	.	.	.	.	.	.	.	.	.	.	.	.
4693	1	PANK1	NM_148977	chr10	91329233	91395309	-	.	.	.	.	.	.	.	.	.	.	.	.	.	.	.	.	.	.	.	.
4917	1	PPP1R3C	NM_005398	chr10	93378176	93382838	-	.	.	.	.	.	.	.	.	.	.	.	.	.	.	.	.	.	.	.	.
1816	2	TNKS2	NM_025235	chr10	93548130	93615212	+	.	.	.	.	.	.	.	.	.	.	.	.	.	.	.	.	.	.	.	.
6210	0,5	CPEB3	NM_014912	chr10	93798376	94040855	-	.	.	.	.	.	.	.	.	.	.	.	.	.	.	.	.	.	.	.	.
2222	1,5	KIF11	NM_004523	chr10	94342804	94405132	+	CL529068	chr10	94385935	-	.	.	.	.	.	.	.	.	.	.	.	.	.	.	.	.
4039	1	HHEX	NM_002729	chr10	94439660	94445388	+	.	.	.	.	.	.	.	.	.	.	.	.	.	.	.	.	.	.	.	.
2562	1,25	CYP26A1	NM_000783	chr10	94823636	94827631	+	.	.	.	.	.	.	.	.	.	.	.	.	.	.	.	.	.	.	.	.
6643	0,5	MYOF	NM_013451	chr10	95056175	95232064	-	.	.	.	.	.	.	.	.	.	.	.	.	.	.	.	.	.	.	.	.
3341	1	CEP55	NM_018131	chr10	95246378	95278839	+	.	.	.	.	.	.	.	.	.	.	.	.	.	.	.	.	.	.	.	.
5079	1	RBP4	NM_006744	chr10	95341582	95350983	-	.	.	.	.	.	.	.	.	.	.	.	.	.	.	.	.	.	.	.	.
1092	2,25	LGI1	NM_005097	chr10	95507555	95547906	+	.	.	.	.	.	.	.	.	.	.	.	.	.	.	.	.	.	.	.	.
1634	2	PLCE1	NM_001165979	chr10	95838819	96078138	+	.	.	.	.	.	.	.	.	5_GVJ7CIC0	chr10	95770987	+	.	.	.	.	2572_223_1	chr10	95899551	-
1634	2	PLCE1	NM_001165979	chr10	95838819	96078138	+	.	.	.	.	.	.	.	.	_175_1_155	chr10	95770987	+	.	.	.	.	2572_223_1	chr10	95899551	-
3506	1	CYP2C18	NM_000772	chr10	96433240	96485937	+	.	.	.	.	.	.	.	.	.	.	.	.	.	.	.	.	1314_139_1	chr10	96468127	-
3507	1	CYP2C19	NM_000769	chr10	96512452	96602661	+	.	.	.	.	.	.	.	.	.	.	.	.	.	.	.	.	.	.	.	.
3509	1	CYP2C9	NM_000771	chr10	96688404	96739138	+	.	.	.	.	.	.	.	.	.	.	.	.	.	.	.	.	.	.	.	.
3508	1	CYP2C8	NM_001198853	chr10	96786518	96819244	-	.	.	.	.	.	.	.	.	.	.	.	.	.	.	.	.	.	.	.	.
6954	0,5	SORBS1	NM_024991	chr10	97061519	97311161	-	.	.	.	.	.	.	.	.	.	.	.	.	.	.	.	.	.	.	.	.
2946	1	ALDH18A1	NM_001017423	chr10	97355675	97406557	-	.	.	.	.	.	.	.	.	.	.	.	.	.	.	.	.	.	.	.	.
3686	1	ENTPD1	NM_001098175	chr10	97461525	97627013	+	.	.	.	.	.	.	.	.	.	.	.	.	.	.	.	.	.	.	.	.
3149	1	BLNK	NR_047681	chr10	97941444	98021263	-	.	.	.	.	.	.	.	.	.	.	.	.	.	.	.	.	.	.	.	.
7288	0,25	DNTT	NM_004088	chr10	98054074	98088311	+	.	.	.	.	.	.	.	.	.	.	.	.	.	.	.	.	.	.	.	.
6702	0,5	OPALIN	NM_033207	chr10	98092964	98109082	-	.	.	.	.	.	.	.	.	.	.	.	.	.	.	.	.	.	.	.	.
2712	1,25	PIK3AP1	NM_152309	chr10	98343058	98470269	-	.	.	.	.	.	.	.	.	.	.	.	.	.	.	.	.	.	.	.	.
6933	0,5	SLIT1	NM_003061	chr10	98747784	98935673	-	.	.	.	.	.	.	.	.	.	.	.	.	.	.	.	.	.	.	.	.
7169	0,25	ARHGAP19	NM_001256423	chr10	98971919	99020437	-	.	.	.	.	.	.	.	.	.	.	.	.	.	.	.	.	.	.	.	.
3802	1	FRAT1	NM_005479	chr10	99069011	99071662	+	.	.	.	.	.	.	.	.	.	.	.	.	.	.	.	.	.	.	.	.
4763	1	PGAM1	NM_002629	chr10	99176016	99183188	+	.	.	.	.	.	.	.	.	.	.	.	.	.	.	.	.	.	.	.	.
4418	1	MMS19	NM_022362	chr10	99208070	99248356	-	.	.	.	.	.	.	.	.	.	.	.	.	.	.	.	.	.	.	.	.
4792	1	PI4K2A	NM_018425	chr10	99390432	99426177	+	.	.	.	.	.	.	.	.	.	.	.	.	.	.	.	.	.	.	.	.
5219	1	SFRP5	NM_003015	chr10	99516497	99521746	-	.	.	.	.	.	.	.	.	.	.	.	.	.	.	.	.	.	.	.	.
6219	0,5	CRTAC1	NM_018058	chr10	99614746	99780575	-	.	.	.	.	.	.	.	.	_383_1_232	chr10	99671118	-	94_1_2801_21	chr10	99700327	+	.	.	.	.
4078	1	HPSE2	NM_001166246	chr10	100206823	100985622	-	.	.	.	.	.	.	.	.	.	.	.	.	.	.	.	.	.	.	.	.
6195	0,5	CNNM1	NM_020348	chr10	101078845	101144077	+	.	.	.	.	.	.	.	.	.	.	.	.	.	.	.	.	.	.	.	.
2168	1,5	GOT1	NM_002079	chr10	101146616	101180520	-	.	.	.	.	.	.	.	.	.	.	.	.	.	.	.	.	.	.	.	.
3689	1	ENTPD7	NM_020354	chr10	101409252	101460992	+	BH609640	chr10	101433798	+	.	.	.	.	.	.	.	.	.	.	.	.	.	.	.	.
3430	1	COX15	NM_078470	chr10	101458494	101482413	-	.	.	.	.	.	.	.	.	.	.	.	.	.	.	.	.	.	.	.	.
6231	0,5	CUTC	NM_015960	chr10	101481947	101505884	+	.	.	.	.	.	.	.	.	.	.	.	.	.	.	.	.	.	.	.	.
716	2,75	CHUK	NM_001278	chr10	101938113	101979334	-	.	.	.	.	.	.	.	.	.	.	.	.	.	.	.	.	.	.	.	.

Table S2

tumor associated genes								HIV				MLV				MMTV				MMTV(SIN)				MMTV(SIN)arrest			
rank	score	gene symbol	RefSEq	chrom	txStart	txEnd	strand	integrant	chr	position	strand	integrant	chr	position	strand	integrant	chr	position	strand	integrant	chr	position	strand	integrant	chr	position	strand
6746	0,5	PKD2L1	NM_001253837	chr10	102037892	102080233	-	.	.	.	.	.	.	.	.	.	.	.	.	.	.	.	.	.	.	.	.
5194	1	SCD	NM_005063	chr10	102096761	102114578	+	.	.	.	.	.	.	.	.	.	.	.	.	.	.	.	.	2104_183_1	chr10	102113615	-
702	3	WNT8B	NM_003393	chr10	102212801	102233389	+	.	.	.	.	.	.	.	.	.	.	.	.	.	.	.	.	.	.	.	.
1569	2	NDUFB8	NM_005004	chr10	102273486	102279626	-	.	.	.	.	.	.	.	.	.	.	.	.	.	.	.	.	.	.	.	.
4044	1	HIF1AN	NM_017902	chr10	102285630	102303671	+	.	.	.	.	.	.	.	.	.	.	.	.	.	.	.	.	.	.	.	.
310	4	TLX1	NM_001195517	chr10	102881050	102887536	+	.	.	.	.	.	.	.	.	.	.	.	.	.	.	.	.	.	.	.	.
2521	1,25	BTRC	NM_033637	chr10	103103779	103307068	+	.	.	.	.	.	.	.	.	.	.	.	.	.	.	.	.	.	.	.	.
4866	1	POLL	NM_001174084	chr10	103328628	103338017	-	.	.	.	.	.	.	.	.	.	.	.	.	.	.	.	.	.	.	.	.
2141	1,5	FGF8	NM_006119	chr10	103519876	103525749	-	.	.	.	.	.	.	.	.	.	.	.	.	.	.	.	.	.	.	.	.
1678	2	PPRC1	NM_015062	chr10	103882776	103900080	+	.	.	.	.	.	.	.	.	.	.	.	.	.	.	.	.	.	.	.	.
628	3	NFKB2	NR_048560	chr10	104143856	104152276	+	.	.	.	.	.	.	.	.	.	.	.	.	.	.	.	.	.	.	.	.
6778	0,5	PSD	NM_002779	chr10	104152365	104168891	-	.	.	.	.	.	.	.	.	.	.	.	.	.	.	.	.	.	.	.	.
6347	0,5	FBXL15	NM_024326	chr10	104169560	104172883	+	.	.	.	.	.	.	.	.	.	.	.	.	.	.	.	.	.	.	.	.
5986	0,5	ACTR1A	NM_005736	chr10	104228975	104252502	-	.	.	.	.	.	.	.	.	.	.	.	.	.	.	.	.	.	.	.	.
346	3,75	SUFU	NM_001178133	chr10	104253708	104369210	+	CL529069	chr10	104343986	-	.	.	.	.	.	.	.	.	.	.	.	.	.	.	.	.
553	3	CYP17A1	NM_000102	chr10	104580277	104587280	-	.	.	.	.	.	.	.	.	.	.	.	.	.	.	.	.	.	.	.	.
7251	0,25	CNNM2	NM_017649	chr10	104668064	104828334	+	.	.	.	.	.	.	.	.	.	.	.	.	.	.	.	.	.	.	.	.
2290	1,5	NT5C2	NM_001134373	chr10	104837763	104943053	-	CL529447	chr10	104907314	-	.	.	.	.	.	.	.	.	.	.	.	.	.	.	.	.
4146	1	INA	NM_032727	chr10	105026909	105040098	+	.	.	.	.	.	.	.	.	.	.	.	.	.	.	.	.	.	.	.	.
2429	1,5	TAF5	NM_006951	chr10	105117713	105138812	+	.	.	.	.	.	.	.	.	.	.	.	.	.	.	.	.	.	.	.	.
4729	1	PDCD11	NM_014976	chr10	105146401	105196009	+	.	.	.	.	.	.	.	.	.	.	.	.	.	.	.	.	.	.	.	.
1575	2	NEURL	NM_004210	chr10	105243724	105342299	+	.	.	.	.	.	.	.	.	.	.	.	.	.	.	.	.	.	.	.	.
6886	0,5	SH3PXD2A	NM_014631	chr10	105343773	105605154	-	.	.	.	.	.	.	.	.	.	.	.	.	.	.	.	.	.	.	.	.
5292	1	SLK	NM_014720	chr10	105717459	105777332	+	CL799691	chr10	105742494	+	.	.	.	.	.	.	.	.	.	.	.	.	.	.	.	.
5292	1	SLK	NM_014720	chr10	105717459	105777332	+	AY517051.1	chr10	105748346	+	.	.	.	.	.	.	.	.	.	.	.	.	.	.	.	.
3413	1	COL17A1	NM_000494	chr10	105781035	105835628	-	.	.	.	.	.	.	.	.	.	.	.	.	.	.	.	.	.	.	.	.
3982	1	GSTO2	NM_183239	chr10	106018620	106049166	+	.	.	.	.	.	.	.	.	.	.	.	.	.	.	.	.	.	.	.	.
5315	1	SORCS3	NM_014978	chr10	106390848	107014983	+	.	.	.	.	.	.	.	.	.	.	.	.	30_97	chr10	106548093	-	.	.	.	.
5994	0,5	ADD3	NM_019903	chr10	111755715	111885313	+	.	.	.	.	.	.	.	.	.	.	.	.	.	.	.	.	.	.	.	.
7442	0,25	MXI1	NM_005962	chr10	111975751	112037113	+	CL529071	chr10	111994042	+	.	.	.	.	.	.	.	.	.	.	.	.	.	.	.	.
3629	1	DUSP5	NM_004419	chr10	112247614	112261292	+	.	.	.	.	.	.	.	.	.	.	.	.	.	.	.	.	.	.	.	.
7467	0,25	PDCD4	NM_014456	chr10	112621542	112649754	+	.	.	.	.	.	.	.	.	.	.	.	.	.	.	.	.	.	.	.	.
3938	1	GPAM	NM_001244949	chr10	113899611	113933527	-	.	.	.	.	.	.	.	.	.	.	.	.	.	.	.	.	.	.	.	.
1203	2	ACSL5	NM_203379	chr10	114123905	114178128	+	.	.	.	.	.	.	.	.	.	.	.	.	.	.	.	.	.	.	.	.
1798	2	TCF7L2	NM_001198529	chr10	114699998	114917426	+	.	.	.	.	.	.	.	.	.	.	.	.	.	.	.	.	.	.	.	.
7340	0,25	HABP2	NM_001177660	chr10	115300579	115339350	+	.	.	.	.	.	.	.	.	.	.	.	.	.	.	.	.	.	.	.	.
2287	1,5	NRAP	NM_006175	chr10	115338572	115413819	-	.	.	.	.	.	.	.	.	.	.	.	.	.	.	.	.	.	.	.	.
2051	1,5	CASP7	NM_033339	chr10	115429417	115480658	+	.	.	.	.	.	.	.	.	.	.	.	.	.	.	.	.	.	.	.	.
3537	1	DCLRE1A	NM_014881	chr10	115584473	115603849	-	AY516931.1	chr10	115584810	+	.	.	.	.	.	.	.	.	.	.	.	.	.	.	.	.
2918	1	AFAP1L2	NM_032550	chr10	116044572	116154505	-	.	.	.	.	.	.	.	.	.	.	.	.	.	.	.	.	.	.	.	.
2861	1	ABLIM1	NM_001003407	chr10	116180858	116434404	-	.	.	.	.	.	.	.	.	.	.	.	.	.	.	.	.	.	.	.	.
791	2,5	ATRNL1	NM_207303	chr10	116843113	117698486	+	BH609642	chr10	117371717	+	.	.	.	.	.	.	.	.	778_248	chr10	117491302	-	.	.	.	.
3879	1	GFRA1	NM_145793	chr10	117806431	118021809	-	.	.	.	.	.	.	.	.	.	.	.	.	.	.	.	.	.	.	.	.
4857	1	PNLIP	NM_000936	chr10	118295417	118317357	+	.	.	.	.	.	.	.	.	.	.	.	.	.	.	.	.	.	.	.	.
4858	1	PNLIPRP1	NM_006229	chr10	118340479	118358676	+	.	.	.	.	.	.	.	.	.	.	.	.	.	.	.	.	.	.	.	.
6452	0,5	HSPA12A	NM_025015	chr10	118420692	118492075	-	.	.	.	.	.	.	.	.	.	.	.	.	07_1_2514_12	chr10	118489119	+	.	.	.	.
873	2,5	KIAA1598	NM_001258299	chr10	118649812	118755078	-	.	.	.	.	.	.	.	.	.	.	.	.	.	.	.	.	.	.	.	.
7550	0,25	SLC18A2	NM_003054	chr10	118990573	119028931	+	.	.	.	.	.	.	.	.	.	.	.	.	.	.	.	.	.	.	.	.
3677	1	EMX2	NM_001165924	chr10	119291945	119299047	+	.	.	.	.	.	.	.	.	.	.	.	.	.	.	.	.	.	.	.	.
6787	0,5	RAB11FIP2	NM_014904	chr10	119754416	119796104	-	.	.	.	.	.	.	.	.	.	.	.	.	.	.	.	.	.	.	.	.
6646	0,5	NANOS1	NM_199461	chr10	120779217	120783844	+	.	.	.	.	.	.	.	.	.	.	.	.	.	.	.	.	.	.	.	.

Table S2

tumor associated genes								HIV				MLV				MMTV				MMTV(SIN)				MMTV(SIN)arrest			
rank	score	gene symbol	RefSEq	chrom	txStart	txEnd	strand	integrant	chr	position	strand	integrant	chr	position	strand	integrant	chr	position	strand	integrant	chr	position	strand	integrant	chr	position	strand
2578	1,25	EIF3A	NM_003750	chr10	120784530	120830324	-	CL529245	chr10	120805636	+	.	.	.	.	.	.	.	.	.	.	.	.	.	.	.	.
3973	1	GRK5	NM_005308	chr10	120957186	121205121	+	.	.	.	.	AY516353	chr10	121085878	+	.	.	.	.	.	.	.	.	.	.	.	.
1474	2	INPP5F	NM_001243194	chr10	121568758	121578652	+	.	.	.	.	.	.	.	.	.	.	.	.	.	.	.	.	2763_245_1	chr10	121540726	+
4887	1	PPAPDC1A	NM_001030059	chr10	122206455	122339357	+	.	.	.	.	.	.	.	.	.	.	.	.	.	.	.	.	.	.	.	.
79	6,25	FGFR2	NM_001144917	chr10	123227833	123347962	-	.	.	.	.	.	.	.	.	.	.	.	.	.	.	.	.	.	.	.	.
2427	1,5	TACC2	NM_206862	chr10	123738678	124004047	+	.	.	.	.	.	.	.	.	.	.	.	.	.	.	.	.	.	.	.	.
6751	0,5	PLEKHA1	NM_001001974	chr10	124124083	124181861	+	CL529345	chr10	124162564	-	.	.	.	.	.	.	.	.	.	.	.	.	.	.	.	.
6459	0,5	HTRA1	NM_002775	chr10	124211030	124264414	+	.	.	.	.	.	.	.	.	.	.	.	.	.	.	.	.	.	.	.	.
5900	0,75	DMBT1	NM_007329	chr10	124310170	124393242	+	.	.	.	.	.	.	.	.	.	.	.	.	.	.	.	.	.	.	.	.
1698	2	PSTK	NM_153336	chr10	124729545	124739896	+	.	.	.	.	.	.	.	.	.	.	.	.	.	.	.	.	.	.	.	.
2872	1	ACADSB	NM_001609	chr10	124758418	124807796	+	.	.	.	.	.	.	.	.	.	.	.	.	.	.	.	.	.	.	.	.
2522	1,25	BUB3	NM_001007793	chr10	124903749	124914876	+	.	.	.	.	.	.	.	.	.	.	.	.	.	.	.	.	.	.	.	.
6397	0,5	GPR26	NM_153442	chr10	125415860	125446903	+	.	.	.	.	.	.	.	.	.	.	.	.	.	.	.	.	.	.	.	.
4653	1	OAT	NM_001171814	chr10	126075861	126097535	-	.	.	.	.	.	.	.	.	.	.	.	.	.	.	.	.	.	.	.	.
1502	2	LHPP	NM_001167880	chr10	126140330	126292700	+	.	.	.	.	.	.	.	.	.	.	.	.	.	.	.	.	.	.	.	.
1394	2	FAM53B	NM_014661	chr10	126297852	126422920	-	.	.	.	.	.	.	.	.	.	.	.	.	.	.	.	.	.	.	.	.
4382	1	METTL10	NM_212554	chr10	126437395	126470429	-	.	.	.	.	.	.	.	.	.	.	.	.	.	.	.	.	.	.	.	.
3725	1	FAM175B	NM_032182	chr10	126480343	126515229	+	.	.	.	.	.	.	.	.	.	.	.	.	.	.	.	.	.	.	.	.
5868	1	ZRANB1	NM_017580	chr10	126620681	126665995	+	.	.	.	.	.	.	.	.	.	.	.	.	.	.	.	.	.	.	.	.
3480	1	CTBP2	NM_022802	chr10	126666407	126706443	-	.	.	.	.	.	.	.	.	.	.	.	.	.	.	.	.	.	.	.	.
1278	2	C10orf137	NM_015608	chr10	127398073	127442702	+	.	.	.	.	.	.	.	.	.	.	.	.	.	.	.	.	.	.	.	.
2454	1,5	UROS	NM_000375	chr10	127467136	127501827	-	.	.	.	.	.	.	.	.	.	.	.	.	.	.	.	.	.	.	.	.
3133	1	BCCIP	NM_078469	chr10	127502093	127521366	+	.	.	.	.	.	.	.	.	.	.	.	.	.	.	.	.	.	.	.	.
3579	1	DHX32	NM_018180	chr10	127514898	127559874	-	.	.	.	.	.	.	.	.	.	.	.	.	.	.	.	.	.	.	.	.
519	3	ADAM12	NM_003474	chr10	127692894	128067117	-	.	.	.	.	.	.	.	.	.	.	.	.	.	.	.	.	.	.	.	.
2032	1,5	C10orf90	NM_001004298	chr10	128103563	128200000	-	.	.	.	.	.	.	.	.	.	.	.	.	.	.	.	.	.	.	.	.
369	3,5	DOCK1	NM_001380	chr10	128584012	129140770	+	.	.	.	.	.	.	.	.	.	.	.	.	s1601_247	chr10	128906448	-	.	.	.	.
3396	1	CLRN3	NM_152311	chr10	129566103	129581201	-	.	.	.	.	.	.	.	.	.	.	.	.	.	.	.	.	.	.	.	.
1701	2	PTPRE	NM_006504	chr10	129595314	129774154	+	.	.	.	.	.	.	.	.	.	.	.	.	.	.	.	.	.	.	.	.
894	2,5	MKI67	NM_001145966	chr10	129784913	129814458	-	.	.	.	.	.	.	.	.	.	.	.	.	.	.	.	.	.	.	.	.
398	3,5	MGMT	NM_002412	chr10	131155443	131455773	+	.	.	.	.	.	.	.	.	.	.	.	.	.	.	.	.	.	.	.	.
1424	2	GLRX3	NM_006541	chr10	131824628	131867922	+	.	.	.	.	.	.	.	.	.	.	.	.	.	.	.	.	.	.	.	.
1797	2	TCERG1L	NM_174937	chr10	132780644	132999974	-	.	.	.	.	.	.	.	.	.	.	.	.	.	.	.	.	.	.	.	.
1671	2	PPP2R2D	NR_033191	chr10	133597949	133620043	+	.	.	.	.	.	.	.	.	.	.	.	.	.	.	.	.	.	.	.	.
2516	1,25	BNIP3	NM_004052	chr10	133631193	133645425	-	.	.	.	.	.	.	.	.	.	.	.	.	.	.	.	.	.	.	.	.
2211	1,5	JAKMIP3	NM_001105521	chr10	133768302	133848303	+	.	.	.	.	.	.	.	.	.	.	.	.	.	.	.	.	938_125_1	chr10	133836182	-
1900	1,75	DPYSL4	NM_006426	chr10	133850403	133869270	+	.	.	.	.	.	.	.	.	.	.	.	.	.	.	.	.	.	.	.	.
1784	2	STK32C	NM_173575	chr10	133870985	133971467	-	.	.	.	.	.	.	.	.	.	.	.	.	.	.	.	.	.	.	.	.
4306	1	LRRC27	NM_001143757	chr10	133995730	134045000	+	.	.	.	.	.	.	.	.	.	.	.	.	.	.	.	.	.	.	.	.
5010	1	PWWP2B	NM_138499	chr10	134060691	134081348	+	.	.	.	.	.	.	.	.	.	.	.	.	.	.	.	.	.	.	.	.
386	3,5	INPP5A	NM_005539	chr10	134201342	134446974	+	.	.	.	.	.	.	.	.	.	.	.	.	.	.	.	.	.	.	.	.
2282	1,5	NKX6-2	NM_177400	chr10	134448309	134449527	-	.	.	.	.	.	.	.	.	.	.	.	.	.	.	.	.	.	.	.	.
2169	1,5	GPR123	NM_001083909	chr10	134751398	134795169	+	.	.	.	.	.	.	.	.	.	.	.	.	.	.	.	.	.	.	.	.
2226	1,5	KNDC1	NM_152643	chr10	134823960	134889906	+	.	.	.	.	.	.	.	.	.	.	.	.	.	.	.	.	.	.	.	.
7151	0,25	ADAM8	NM_001109	chr10	134925910	134940397	-	.	.	.	.	.	.	.	.	.	.	.	.	.	.	.	.	.	.	.	.
5589	1	TUBGCP2	NR_046330	chr10	134942123	134975831	-	.	.	.	.	.	.	.	.	.	.	.	.	.	.	.	.	.	.	.	.
5813	1	ZNF511	NM_145806	chr10	134972412	134976656	+	.	.	.	.	.	.	.	.	.	.	.	.	.	.	.	.	.	.	.	.
6124	0,5	CALY	NM_015722	chr10	134988917	135000465	-	.	.	.	.	.	.	.	.	.	.	.	.	.	.	.	.	.	.	.	.
1373	2	ECHS1	NM_004092	chr10	135025976	135036898	-	.	.	.	.	.	.	.	.	.	.	.	.	.	.	.	.	.	.	.	.
2703	1,25	PAOX	NM_152911	chr10	135042730	135055188	+	.	.	.	.	.	.	.	.	.	.	.	.	.	.	.	.	.	.	.	.
6965	0,5	SPRN	NM_001012508	chr10	135084159	135088111	-	.	.	.	.	.	.	.	.	.	.	.	.	.	.	.	.	.	.	.	.

Table S2

tumor associated genes								HIV				MLV				MMTV				MMTV(SIN)				MMTV(SIN)arrest			
rank	score	gene symbol	RefSEq	chrom	txStart	txEnd	strand	integrant	chr	position	strand	integrant	chr	position	strand	integrant	chr	position	strand	integrant	chr	position	strand	integrant	chr	position	strand
2563	1,25	CYP2E1	NM_000773	chr10	135190856	135202610	+	.	.	.	.	.	.	.	.	.	.	.	.	.	.	.	.	.	.	.	.
6993	0,5	SYCE1	NM_001143764	chr10	135218337	135229128	-	.	.	.	.	.	.	.	.	.	.	.	.	.	.	.	.	.	.	.	.
7454	0,25	ODF3	NM_053280	chr11	186760	190258	+	.	.	.	.	.	.	.	.	.	.	.	.	.	.	.	.	.	.	.	.
7186	0,25	BET1L	NM_001098787	chr11	192923	197422	-	.	.	.	.	.	.	.	.	.	.	.	.	.	.	.	.	.	.	.	.
7514	0,25	RIC8A	NM_021932	chr11	198529	205110	+	.	.	.	.	.	.	.	.	.	.	.	.	.	.	.	.	.	.	.	.
7546	0,25	SIRT3	NM_001017524	chr11	205029	226362	-	CL529521	chr11	209079	+	.	.	.	.	.	.	.	.	.	.	.	.	.	.	.	.
4972	1	PSMD13	NM_175932	chr11	226807	242984	+	.	.	.	.	.	.	.	.	.	.	.	.	.	.	.	.	.	.	.	.
7446	0,25	NLRP6	NM_138329	chr11	268569	275304	+	.	.	.	.	.	.	.	.	.	.	.	.	.	.	.	.	.	.	.	.
6037	0,5	ATHL1	NM_025092	chr11	279137	285688	+	.	.	.	.	.	.	.	.	.	.	.	.	.	.	.	.	.	.	.	.
7365	0,25	IFITM5	NM_001025295	chr11	288202	289526	-	.	.	.	.	.	.	.	.	.	.	.	.	.	.	.	.	.	.	.	.
5923	0,75	IFITM2	NM_006435	chr11	298106	299410	+	.	.	.	.	.	.	.	.	.	.	.	.	.	.	.	.	.	.	.	.
5922	0,75	IFITM1	NM_003641	chr11	303990	305272	+	.	.	.	.	.	.	.	.	.	.	.	.	.	.	.	.	.	.	.	.
4118	1	IFITM3	NR_049759	chr11	309672	310914	-	.	.	.	.	.	.	.	.	.	.	.	.	.	.	.	.	.	.	.	.
7480	0,25	PKP3	NM_007183	chr11	384216	394908	+	.	.	.	.	.	.	.	.	.	.	.	.	.	.	.	.	.	.	.	.
4982	1	PTDSS2	NM_030783	chr11	440279	481387	+	.	.	.	.	.	.	.	.	.	.	.	.	.	.	.	.	.	.	.	.
60	6,75	HRAS	NM_001130442	chr11	522241	525550	-	.	.	.	.	.	.	.	.	.	.	.	.	.	.	.	.	.	.	.	.
2748	1,25	RASSF7	NM_003475	chr11	551449	554025	+	.	.	.	.	.	.	.	.	.	.	.	.	.	.	.	.	.	.	.	.
7377	0,25	IRF7	NM_004031	chr11	602554	605728	-	.	.	.	.	.	.	.	.	.	.	.	.	.	.	.	.	.	.	.	.
5429	1	TALDO1	NM_006755	chr11	737431	755024	+	.	.	.	.	.	.	.	.	.	.	.	.	.	.	.	.	.	.	.	.
6909	0,5	SLC25A22	NM_001191061	chr11	780474	788269	-	.	.	.	.	.	.	.	.	.	.	.	.	.	.	.	.	.	.	.	.
5887	0,75	CD151	NM_139030	chr11	822951	828835	+	.	.	.	.	.	.	.	.	.	.	.	.	.	.	.	.	.	.	.	.
927	2,5	POLR2L	NM_021128	chr11	829720	832529	-	.	.	.	.	.	.	.	.	.	.	.	.	.	.	.	.	.	.	.	.
7606	0,25	TSPAN4	NM_001025235	chr11	832940	857116	+	.	.	.	.	.	.	.	.	.	.	.	.	.	.	.	.	.	.	.	.
2000	1,5	AP2A2	NM_001242837	chr11	915808	1002245	+	CL800811	chr11	997880	-	.	.	.	.	.	.	.	.	.	.	.	.	.	.	.	.
2686	1,25	MUC2	NM_002457	chr11	1064874	1094417	+	.	.	.	.	.	.	.	.	.	.	.	.	.	.	.	.	.	.	.	.
7441	0,25	MUC5B	NM_002458	chr11	1200870	1239982	+	.	.	.	.	.	.	.	.	.	.	.	.	.	.	.	.	.	.	.	.
7041	0,5	TOLLIP	NM_019009	chr11	1252173	1287468	-	.	.	.	.	.	.	.	.	.	.	.	.	.	.	.	.	.	.	.	.
2520	1,25	BRSK2	NM_001256630	chr11	1388957	1440495	+	.	.	.	.	.	.	.	.	.	.	.	.	.	.	.	.	.	.	.	.
2114	1,5	DUSP8	NM_004420	chr11	1531856	1549726	-	.	.	.	.	.	.	.	.	.	.	.	.	.	.	.	.	.	.	.	.
1021	2,25	CTSD	NM_001909	chr11	1730557	1741798	-	.	.	.	.	.	.	.	.	.	.	.	.	.	.	.	.	.	.	.	.
2610	1,25	H19	NR_002196	chr11	1972981	1975641	-	.	.	.	.	.	.	.	.	.	.	.	.	.	.	.	.	.	.	.	.
860	2,5	IGF2	NM_000612	chr11	2106922	2116780	-	.	.	.	.	.	.	.	.	.	.	.	.	.	.	.	.	.	.	.	.
4151	1	INS	NM_000207	chr11	2137584	2139015	-	.	.	.	.	.	.	.	.	.	.	.	.	.	.	.	.	.	.	.	.
5464	1	TH	NM_000360	chr11	2141734	2149611	-	.	.	.	.	.	.	.	.	.	.	.	.	.	.	.	.	.	.	.	.
7174	0,25	ASCL2	NM_005170	chr11	2246303	2248758	-	.	.	.	.	.	.	.	.	.	.	.	.	.	.	.	.	.	.	.	.
7239	0,25	CD81	NM_004356	chr11	2355122	2375225	+	.	.	.	.	.	.	.	.	.	.	.	.	.	.	.	.	.	.	.	.
7384	0,25	KCNQ1	NR_040711	chr11	2439241	2826916	+	.	.	.	.	AY516651	chr11	2803603	-	.	.	.	.	.	.	.	.	.	.	.	.
6506	0,5	KCNQ1DN	NR_024627	chr11	2847838	2849911	+	.	.	.	.	.	.	.	.	.	.	.	.	.	.	.	.	.	.	.	.
807	2,5	CDKN1C	NM_001122630	chr11	2861023	2863571	-	.	.	.	.	.	.	.	.	.	.	.	.	.	.	.	.	.	.	.	.
7551	0,25	SLC22A18	NM_002555	chr11	2880087	2903052	+	.	.	.	.	.	.	.	.	.	.	.	.	.	.	.	.	.	.	.	.
7475	0,25	PHLDA2	NM_003311	chr11	2906078	2907226	-	.	.	.	.	.	.	.	.	.	.	.	.	.	.	.	.	.	.	.	.
538	3	CARS	NM_001014437	chr11	2978727	3035257	-	.	.	.	.	.	.	.	.	.	.	.	.	.	.	.	.	.	.	.	.
408	3,5	NUP98	NM_139132	chr11	3652815	3775598	-	.	.	.	.	.	.	.	.	.	.	.	.	.	.	.	.	1738_162_1	chr11	3745576	+
1723	2	RHOG	NM_001665	chr11	3804783	3818789	-	.	.	.	.	.	.	.	.	.	.	.	.	.	.	.	.	.	.	.	.
504	3,25	RRM1	NM_001033	chr11	4072499	4116682	+	.	.	.	.	.	.	.	.	.	.	.	.	.	.	.	.	.	.	.	.
4668	1	OR51E1	NM_152430	chr11	4621731	4633292	+	.	.	.	.	.	.	.	.	.	.	.	.	.	.	.	.	.	.	.	.
4020	1	HBB	NM_000518	chr11	5203271	5204877	-	.	.	.	.	.	.	.	.	.	.	.	.	.	.	.	.	2795_249_1	chr11	5203419	-
6423	0,5	HBD	NM_000519	chr11	5210634	5212434	-	.	.	.	.	.	.	.	.	.	.	.	.	.	.	.	.	.	.	.	.
5614	1	UBQLNL	NM_145053	chr11	5492198	5494532	-	.	.	.	.	.	.	.	.	.	.	.	.	.	.	.	.	.	.	.	.
7048	0,5	TRIM22	NM_001199573	chr11	5667392	5688669	+	.	.	.	.	.	.	.	.	.	.	.	.	.	.	.	.	.	.	.	.
1008	2,25	CCKBR	NM_176875	chr11	6237479	6249933	+	.	.	.	.	.	.	.	.	.	.	.	.	.	.	.	.	.	.	.	.

Table S2

tumor associated genes								HIV				MLV				MMTV				MMTV(SIN)				MMTV(SIN)arrest			
rank	score	gene symbol	RefSEq	chrom	txStart	txEnd	strand	integrant	chr	position	strand	integrant	chr	position	strand	integrant	chr	position	strand	integrant	chr	position	strand	integrant	chr	position	strand
1141	2,25	PRKCDBP	NM_145040	chr11	6296751	6298316	-	.	.	.	.	.	.	.	.	.	.	.	.	.	.	.	.	.	.	.	.
6024	0,5	APBB1	NM_001257321	chr11	6372930	6383467	-	.	.	.	.	.	.	.	.	.	.	.	.	.	.	.	.	.	.	.	.
2450	1,5	TRIM3	NM_001248006	chr11	6426418	6452265	-	.	.	.	.	.	.	.	.	.	.	.	.	.	.	.	.	.	.	.	.
598	3	ILK	NM_001014795	chr11	6581782	6588675	+	.	.	.	.	.	.	.	.	.	.	.	.	.	.	.	.	.	.	.	.
7625	0,25	ZNF215	NM_013250	chr11	6904229	6935854	+	.	.	.	.	.	.	.	.	.	.	.	.	.	.	.	.	.	.	.	.
6237	0,5	CYB5R2	NM_016229	chr11	7642901	7651397	-	.	.	.	.	.	.	.	.	.	.	.	.	.	.	.	.	.	.	.	.
6706	0,5	OR5P2	NM_153444	chr11	7774096	7775065	-	.	.	.	.	.	.	.	.	.	.	.	.	.	.	.	.	.	.	.	.
4586	1	NLRP10	NM_176821	chr11	7937731	7941635	-	.	.	.	.	.	.	.	.	.	.	.	.	.	.	.	.	.	.	.	.
7064	0,5	TUB	NM_177972	chr11	8059484	8084230	+	.	.	.	.	.	.	.	.	.	.	.	.	.	.	.	.	.	.	.	.
1504	2	LMO1	NM_002315	chr11	8202426	8242001	-	.	.	.	.	.	.	.	.	.	.	.	.	.	.	.	.	.	.	.	.
5384	1	STK33	NM_030906	chr11	8369993	8572079	-	.	.	.	.	.	.	.	.	.	.	.	.	.	.	.	.	.	.	.	.
3052	1	ASCL3	NM_020646	chr11	8915694	8921156	-	.	.	.	.	.	.	.	.	.	.	.	.	.	.	.	.	.	.	.	.
6685	0,5	NRIP3	NM_020645	chr11	8958698	8982172	-	.	.	.	.	.	.	.	.	.	.	.	.	.	.	.	.	.	.	.	.
4158	1	IPO7	NM_006391	chr11	9362744	9426250	+	.	.	.	.	.	.	.	.	.	.	.	.	.	.	.	.	.	.	.	.
1853	2	WEE1	NM_001143976	chr11	9552809	9567889	+	.	.	.	.	.	.	.	.	.	.	.	.	.	.	.	.	.	.	.	.
6992	0,5	SWAP70	NM_015055	chr11	9642203	9731083	+	CL799600	chr11	9681169	+	.	.	.	.	.	.	.	.	.	.	.	.	.	.	.	.
313	3,75	ADM	NM_001124	chr11	10283217	10285499	+	.	.	.	.	.	.	.	.	.	.	.	.	.	.	.	.	.	.	.	.
1996	1,5	AMPD3	NM_001172431	chr11	10428443	10485702	+	.	.	.	.	.	.	.	.	.	.	.	.	.	.	.	.	.	.	.	.
7407	0,25	LYVE1	NM_006691	chr11	10535988	10546941	-	.	.	.	.	.	.	.	.	.	.	.	.	.	.	.	.	.	.	.	.
6630	0,5	MRVI1	NM_001206881	chr11	10551213	10672111	-	.	.	.	.	.	.	.	.	.	.	.	.	.	.	.	.	.	.	.	.
3852	1	GALNTL4	NM_198516	chr11	11248996	11600137	-	.	.	.	.	.	.	.	.	.	.	.	.	22_1_2820_24	chr11	11553784	+	.	.	.	.
2106	1,5	DKK3	NM_001018057	chr11	11941118	11986762	-	.	.	.	.	.	.	.	.	.	.	.	.	.	.	.	.	.	.	.	.
4395	1	MICAL2	NM_014632	chr11	12088713	12241907	+	CL800212	chr11	12120968	+	.	.	.	.	.	.	.	.	.	.	.	.	.	.	.	.
5452	1	TEAD1	NM_021961	chr11	12652544	12922860	+	.	.	.	.	.	.	.	.	_351_1_216	chr11	12686687	-	.	.	.	.	.	.	.	.
6032	0,5	ARNTL	NM_001030273	chr11	13255900	13365388	+	.	.	.	.	.	.	.	.	.	.	.	.	.	.	.	.	.	.	.	.
1879	1,75	BTBD10	NM_032320	chr11	13366131	13441414	-	CL529076	chr11	13435652	-	.	.	.	.	.	.	.	.	.	.	.	.	.	.	.	.
1879	1,75	BTBD10	NM_032320	chr11	13366131	13441414	-	CL529075	chr11	13399969	+	.	.	.	.	.	.	.	.	.	.	.	.	.	.	.	.
2729	1,25	PTH	NM_000315	chr11	13470176	13474143	-	.	.	.	.	.	.	.	.	.	.	.	.	.	.	.	.	.	.	.	.
6963	0,5	SPON1	NM_006108	chr11	13940759	14246255	+	.	.	.	.	.	.	.	.	.	.	.	.	.	.	.	.	.	.	.	.
1735	2	RRAS2	NM_001102669	chr11	14256041	14336649	-	.	.	.	.	.	.	.	.	.	.	.	.	.	.	.	.	1763_163_1	chr11	14284166	+
4732	1	PDE3B	NM_000922	chr11	14621844	14850180	+	CL529627	chr11	14686929	-	.	.	.	.	.	.	.	.	.	.	.	.	.	.	.	.
6121	0,5	CALCA	NM_001033953	chr11	14944790	14950408	-	.	.	.	.	.	.	.	.	.	.	.	.	.	.	.	.	.	.	.	.
4152	1	INSC	NM_001042536	chr11	15093059	15225330	+	.	.	.	.	.	.	.	.	.	.	.	.	.	.	.	.	.	.	.	.
1623	2	PIK3C2A	NM_002645	chr11	17064699	17147930	-	.	.	.	.	.	.	.	.	_264_1_178	chr11	17077516	-	.	.	.	.	.	.	.	.
5981	0,5	ABCC8	NM_000352	chr11	17371007	17455025	-	.	.	.	.	.	.	.	.	.	.	.	.	.	.	.	.	.	.	.	.
900	2,5	MYOD1	NM_002478	chr11	17697685	17700254	+	.	.	.	.	.	.	.	.	.	.	.	.	.	.	.	.	.	.	.	.
6493	0,5	KCNC1	NM_004976	chr11	17714070	17755953	+	.	.	.	.	.	.	.	.	.	.	.	.	.	.	.	.	.	.	.	.
2809	1,25	TPH1	NM_004179	chr11	17998659	18018911	-	.	.	.	.	.	.	.	.	.	.	.	.	.	.	.	.	.	.	.	.
7528	0,25	SAA2	NM_030754	chr11	18223350	18226797	-	.	.	.	.	.	.	.	.	.	.	.	.	.	.	.	.	.	.	.	.
6844	0,5	SAA1	NM_001178006	chr11	18244383	18248099	+	.	.	.	.	.	.	.	.	.	.	.	.	.	.	.	.	.	.	.	.
2178	1,5	GTF2H1	NM_001142307	chr11	18300391	18345166	+	.	.	.	.	.	.	.	.	.	.	.	.	.	.	.	.	.	.	.	.
273	4	LDHA	NM_001165414	chr11	18374388	18386341	+	.	.	.	.	.	.	.	.	.	.	.	.	.	.	.	.	.	.	.	.
4273	1	LDHC	NM_002301	chr11	18390428	18429369	+	.	.	.	.	.	.	.	.	.	.	.	.	.	.	.	.	.	.	.	.
4271	1	LDHAL6A	NM_144972	chr11	18433949	18457723	+	.	.	.	.	.	.	.	.	.	.	.	.	.	.	.	.	.	.	.	.
2451	1,5	TSG101	NM_006292	chr11	18458433	18505079	-	CL799589	chr11	18471868	-	.	.	.	.	.	.	.	.	.	.	.	.	.	.	.	.
4125	1	IGSF22	NM_173588	chr11	18682427	18704353	-	.	.	.	.	.	.	.	.	.	.	.	.	.	.	.	.	.	.	.	.
5001	1	PTPN5	NM_032781	chr11	18706050	18769965	-	.	.	.	.	.	.	.	.	.	.	.	.	.	.	.	.	.	.	.	.
7436	0,25	MRGPRX2	NM_054030	chr11	19032578	19038804	-	.	.	.	.	.	.	.	.	.	.	.	.	.	.	.	.	.	.	.	.
6293	0,5	E2F8	NM_001256371	chr11	19202185	19219743	-	.	.	.	.	.	.	.	.	.	.	.	.	.	.	.	.	.	.	.	.
6456	0,5	HTATIP2	NM_001098523	chr11	20342262	20345776	+	.	.	.	.	.	.	.	.	.	.	.	.	.	.	.	.	.	.	.	.
1689	2	PRMT3	NM_001145167	chr11	20365651	20487455	+	.	.	.	.	.	.	.	.	.	.	.	.	.	.	.	.	.	.	.	.

Table S2

tumor associated genes								HIV				MLV				MMTV				MMTV(SIN)				MMTV(SIN)arrest			
rank	score	gene symbol	RefSEq	chrom	txStart	txEnd	strand	integrant	chr	position	strand	integrant	chr	position	strand	integrant	chr	position	strand	integrant	chr	position	strand	integrant	chr	position	strand
6662	0,5	NELL1	NM_006157	chr11	20647692	21553805	+	.	.	.	.	.	.	.	.	.	.	.	.	100_1_509_17	chr11	21366134	-	1124_130_1	chr11	20819295	-
1998	1,5	ANO5	NM_001142649	chr11	22171297	22261489	+	.	.	.	.	.	.	.	.	.	.	.	.	.	.	.	.	.	.	.	.
7549	0,25	SLC17A6	NM_020346	chr11	22316242	22357622	+	.	.	.	.	.	.	.	.	.	.	.	.	.	.	.	.	.	.	.	.
116	5,25	FANCF	NM_022725	chr11	22600654	22603963	-	.	.	.	.	.	.	.	.	.	.	.	.	.	.	.	.	.	.	.	.
7325	0,25	GAS2	NM_001143830	chr11	22644735	22791123	+	.	.	.	.	.	.	.	.	.	.	.	.	.	.	.	.	.	.	.	.
6582	0,5	LUZP2	NM_001252008	chr11	24475091	25060762	+	.	.	.	.	.	.	.	.	.	.	.	.	.	.	.	.	.	.	.	.
2990	1	ANO3	NM_031418	chr11	26310253	26641412	+	.	.	.	.	.	.	.	.	.	.	.	.	.	.	.	.	.	.	.	.
1875	1,75	BBOX1	NM_003986	chr11	27019084	27105930	+	.	.	.	.	.	.	.	.	.	.	.	.	.	.	.	.	.	.	.	.
6141	0,5	CCDC34	NM_030771	chr11	27316636	27341371	-	.	.	.	.	.	.	.	.	.	.	.	.	.	.	.	.	.	.	.	.
2511	1,25	BDNF	NM_170735	chr11	27633017	27637772	-	.	.	.	.	.	.	.	.	.	.	.	.	.	.	.	.	.	.	.	.
5928	0,75	KCNA4	NM_002233	chr11	29987863	29995153	-	.	.	.	.	.	.	.	.	.	.	.	.	.	.	.	.	.	.	.	.
4432	1	MPPED2	NM_001145399	chr11	30362615	30564506	-	.	.	.	.	.	.	.	.	.	.	.	.	.	.	.	.	.	.	.	.
2580	1,25	ELP4	NM_019040	chr11	31487872	31761905	+	CL800617	chr11	31649064	+	.	.	.	.	.	.	.	.	.	.	.	.	.	.	.	.
7463	0,25	PAX6	NM_001258465	chr11	31762915	31789266	-	.	.	.	.	.	.	.	.	.	.	.	.	.	.	.	.	.	.	.	.
62	6,75	WT1	NM_001198552	chr11	32365897	32408939	-	AY517234.1	chr11	32389738	+	.	.	.	.	.	.	.	.	.	.	.	.	.	.	.	.
4046	1	HIPK3	NM_005734	chr11	33235743	33332515	+	.	.	.	.	.	.	.	.	.	.	.	.	.	.	.	.	.	.	.	.
7238	0,25	CD59	NM_001127223	chr11	33681131	33700849	-	.	.	.	.	.	.	.	.	.	.	.	.	.	.	.	.	.	.	.	.
2139	1,5	FBXO3	NM_033406	chr11	33724866	33752647	-	.	.	.	.	.	.	.	.	.	.	.	.	.	.	.	.	.	.	.	.
159	4,75	LMO2	NM_005574	chr11	33836698	33870412	-	.	.	.	.	.	.	.	.	.	.	.	.	.	.	.	.	.	.	.	.
2533	1,25	CAT	NM_001752	chr11	34417047	34450183	+	.	.	.	.	.	.	.	.	.	.	.	.	.	.	.	.	.	.	.	.
7298	0,25	EHF	NM_001206615	chr11	34599163	34641410	+	.	.	.	.	.	.	.	.	.	.	.	.	.	.	.	.	.	.	.	.
151	4,75	CD44	NM_001001392	chr11	35116992	35210525	+	.	.	.	.	.	.	.	.	.	.	.	.	.	.	.	.	.	.	.	.
5962	0,75	SLC1A2	NM_001252652	chr11	35229327	35398186	-	.	.	.	.	.	.	.	.	.	.	.	.	.	.	.	.	.	.	.	.
6714	0,5	PAMR1	NM_015430	chr11	35409951	35503752	-	.	.	.	.	.	.	.	.	_306_1_195	chr11	35413066	-	.	.	.	.	.	.	.	.
3780	1	FJX1	NM_014344	chr11	35596310	35598997	+	.	.	.	.	.	.	.	.	.	.	.	.	.	.	.	.	.	.	.	.
5539	1	TRAF6	NM_004620	chr11	36461892	36488439	-	.	.	.	.	.	.	.	.	.	.	.	.	.	.	.	.	2585_225_1	chr11	36484066	-
5054	1	RAG1	NM_000448	chr11	36546138	36557886	+	.	.	.	.	.	.	.	.	.	.	.	.	.	.	.	.	.	.	.	.
5055	1	RAG2	NM_000536	chr11	36570068	36576405	-	.	.	.	.	.	.	.	.	.	.	.	.	.	.	.	.	.	.	.	.
6578	0,5	LRRC4C	NM_001258419	chr11	40092325	41437762	-	.	.	.	.	.	.	.	.	.	.	.	.	.	.	.	.	.	.	.	.
7165	0,25	API5	NM_001243747	chr11	43290080	43322656	+	.	.	.	.	.	.	.	.	.	.	.	.	.	.	.	.	.	.	.	.
4089	1	HSD17B12	NM_016142	chr11	43658718	43834745	+	CL529441	chr11	43665416	+	.	.	.	.	.	.	.	.	.	.	.	.	.	.	.	.
2967	1	ALKBH3	NM_139178	chr11	43858932	43898401	+	.	.	.	.	.	.	.	.	.	.	.	.	.	.	.	.	.	.	.	.
255	4	EXT2	NM_001178083	chr11	44073674	44223556	+	.	.	.	.	.	.	.	.	.	.	.	.	.	.	.	.	.	.	.	.
1888	1,75	CD82	NM_002231	chr11	44543716	44597891	+	.	.	.	.	.	.	.	.	.	.	.	.	.	.	.	.	.	.	.	.
7001	0,5	SYT13	NM_020826	chr11	45218428	45264460	-	.	.	.	.	.	.	.	.	.	.	.	.	.	.	.	.	.	.	.	.
3360	1	CHST1	NM_003654	chr11	45625814	45643782	-	.	.	.	.	.	.	.	.	.	.	.	.	.	.	.	.	.	.	.	.
6220	0,5	CRY2	NM_021117	chr11	45825532	45861375	+	.	.	.	.	.	.	.	.	.	.	.	.	.	.	.	.	.	.	.	.
2663	1,25	MAPK8IP1	NM_005456	chr11	45863622	45884592	+	.	.	.	.	.	.	.	.	.	.	.	.	.	.	.	.	.	.	.	.
133	5	DGKZ	NM_001105540	chr11	46339720	46358680	+	.	.	.	.	.	.	.	.	.	.	.	.	.	.	.	.	.	.	.	.
2670	1,25	MDK	NM_001270550	chr11	46358909	46361963	+	.	.	.	.	.	.	.	.	.	.	.	.	.	.	.	.	.	.	.	.
248	4	DDB2	NM_000107	chr11	47193068	47217345	+	.	.	.	.	.	.	.	.	.	.	.	.	.	.	.	.	.	.	.	.
2888	1	ACP2	NM_001131064	chr11	47223645	47227033	-	.	.	.	.	.	.	.	.	.	.	.	.	1034_346	chr11	47226153	-	.	.	.	.
4607	1	NR1H3	NM_001130101	chr11	47236043	47247160	+	.	.	.	.	.	.	.	.	.	.	.	.	.	.	.	.	.	.	.	.
611	3	MADD	NM_130475	chr11	47248246	47308158	+	.	.	.	.	.	.	.	.	.	.	.	.	.	.	.	.	.	.	.	.
2784	1,25	SPI1	NM_003120	chr11	47332984	47356703	-	.	.	.	.	.	.	.	.	.	.	.	.	.	.	.	.	.	.	.	.
1700	2	PTPMT1	NM_175732	chr11	47543557	47551589	+	.	.	.	.	.	.	.	.	.	.	.	.	.	.	.	.	.	.	.	.
2274	1,5	NDUFS3	NM_004551	chr11	47557137	47562691	+	.	.	.	.	.	.	.	.	.	.	.	.	.	.	.	.	.	.	.	.
6087	0,5	C1QTNF4	NM_031909	chr11	47567791	47572537	-	.	.	.	.	.	.	.	.	.	.	.	.	.	.	.	.	.	.	.	.
2292	1,5	NUP160	NM_015231	chr11	47756245	47826633	-	CL800140	chr11	47790188	+	.	.	.	.	.	.	.	.	.	.	.	.	.	.	.	.
938	2,5	PTPRJ	NM_001098503	chr11	47958685	48110842	+	.	.	.	.	.	.	.	.	.	.	.	.	.	.	.	.	.	.	.	.
5909	0,75	FOLH1	NM_004476	chr11	49124762	49186798	-	.	.	.	.	.	.	.	.	.	.	.	.	.	.	.	.	.	.	.	.

Table S2

tumor associated genes								HIV				MLV				MMTV				MMTV(SIN)				MMTV(SIN)arrest			
rank	score	gene symbol	RefSEq	chrom	txStart	txEnd	strand	integrant	chr	position	strand	integrant	chr	position	strand	integrant	chr	position	strand	integrant	chr	position	strand	integrant	chr	position	strand
6025	0,5	APLNR	NR_027991	chr11	56757627	56761503	-	.	.	.	.	.	.	.	.	.	.	.	.	.	.	.	.	.	.	.	.
6974	0,5	SSRP1	NM_003146	chr11	56850034	56859927	-	.	.	.	.	.	.	.	.	.	.	.	.	.	.	.	.	.	.	.	.
4937	1	PRG2	NM_002728	chr11	56911409	56914706	-	.	.	.	.	.	.	.	.	.	.	.	.	.	.	.	.	.	.	.	.
5606	1	UBE2L6	NM_198183	chr11	57075703	57092379	-	.	.	.	.	.	.	.	.	.	.	.	.	.	.	.	.	.	.	.	.
1747	2	SERPING1	NM_001032295	chr11	57122280	57138902	+	.	.	.	.	.	.	.	.	.	.	.	.	.	.	.	.	.	.	.	.
7110	0,5	YPEL4	NM_145008	chr11	57169135	57173993	-	.	.	.	.	.	.	.	.	.	.	.	.	.	.	.	.	.	.	.	.
809	2,5	CLP1	NM_001142597	chr11	57181791	57185913	+	.	.	.	.	.	.	.	.	.	.	.	.	.	.	.	.	.	.	.	.
1332	2	CTNND1	NM_001085459	chr11	57285809	57343228	+	CL529077	chr11	57292696	+	.	.	.	.	.	.	.	.	.	.	.	.	.	.	.	.
6705	0,5	OR5B17	NM_001005489	chr11	57882173	57883118	-	.	.	.	.	.	.	.	.	.	.	.	.	.	.	.	.	.	.	.	.
6329	0,5	FAM111B	NM_198947	chr11	58631233	58651464	+	.	.	.	.	.	.	.	.	.	.	.	.	.	.	.	.	.	.	.	.
6328	0,5	FAM111A	NM_022074	chr11	58668827	58679087	+	.	.	.	.	.	.	.	.	.	.	.	.	.	.	.	.	.	.	.	.
1367	2	DTX4	NM_015177	chr11	58696387	58732636	+	.	.	.	.	.	.	.	.	.	.	.	.	.	.	.	.	.	.	.	.
4442	1	MS4A4A	NM_024021	chr11	59804589	59833021	+	.	.	.	.	.	.	.	.	.	.	.	.	.	.	.	.	.	.	.	.
2682	1,25	MS4A1	NM_021950	chr11	59979857	59994801	+	.	.	.	.	.	.	.	.	.	.	.	.	.	.	.	.	.	.	.	.
3301	1	CD6	NM_006725	chr11	60495688	60544424	+	BH609651	chr11	60503243	-	.	.	.	.	.	.	.	.	.	.	.	.	.	.	.	.
7237	0,25	CD5	NM_014207	chr11	60626505	60651899	+	.	.	.	.	.	.	.	.	.	.	.	.	.	.	.	.	.	.	.	.
557	3	DDB1	NM_001923	chr11	60823494	60857260	-	.	.	.	.	.	.	.	.	.	.	.	.	.	.	.	.	2435_211_1	chr11	60834694	-
3526	1	DAK	NM_015533	chr11	60857229	60872807	+	.	.	.	.	.	.	.	.	.	.	.	.	.	.	.	.	.	.	.	.
1044	2,25	FEN1	NM_004111	chr11	61316684	61321290	+	.	.	.	.	.	.	.	.	.	.	.	.	.	.	.	.	.	.	.	.
3713	1	FADS1	NM_013402	chr11	61323672	61341105	-	CL529079	chr11	61325979	+	.	.	.	.	.	.	.	.	.	.	.	.	.	.	.	.
3714	1	FADS2	NM_004265	chr11	61352288	61391401	+	AY517213.1	chr11	61360564	+	.	.	.	.	.	.	.	.	.	.	.	.	.	.	.	.
6056	0,5	BEST1	NM_001139443	chr11	61473931	61487642	+	.	.	.	.	.	.	.	.	.	.	.	.	.	.	.	.	.	.	.	.
3810	1	FTH1	NM_002032	chr11	61488332	61491708	-	.	.	.	.	.	.	.	.	.	.	.	.	.	.	.	.	.	.	.	.
4147	1	INCENP	NM_020238	chr11	61648020	61677211	+	.	.	.	.	.	.	.	.	.	.	.	.	.	.	.	.	.	.	.	.
5196	1	SCGB2A1	NM_002407	chr11	61732715	61737987	+	.	.	.	.	.	.	.	.	.	.	.	.	.	.	.	.	.	.	.	.
7530	0,25	SCGB2A2	NM_002411	chr11	61794205	61797204	+	.	.	.	.	.	.	.	.	.	.	.	.	.	.	.	.	.	.	.	.
3057	1	ASRGL1	NM_001083926	chr11	61861349	61917463	+	.	.	.	.	.	.	.	.	.	.	.	.	.	.	.	.	.	.	.	.
4451	1	MTA2	NM_004739	chr11	62117250	62125888	-	.	.	.	.	.	.	.	.	.	.	.	.	.	.	.	.	.	.	.	.
3112	1	B3GAT3	NM_012200	chr11	62139343	62146223	-	.	.	.	.	.	.	.	.	.	.	.	.	.	.	.	.	.	.	.	.
840	2,5	GANAB	NM_198334	chr11	62148873	62170680	-	.	.	.	.	.	.	.	.	.	.	.	.	.	.	.	.	.	.	.	.
7198	0,25	BSCL2	NR_037949	chr11	62214309	62233667	-	.	.	.	.	.	.	.	.	.	.	.	.	.	.	.	.	.	.	.	.
1057	2,25	GNG3	NM_012202	chr11	62231641	62233254	+	.	.	.	.	.	.	.	.	.	.	.	.	.	.	.	.	.	.	.	.
6443	0,5	HNRNPUL2	NM_001079559	chr11	62236672	62251433	-	.	.	.	.	.	.	.	.	.	.	.	.	.	.	.	.	.	.	.	.
1653	2	POLR2G	NM_002696	chr11	62285586	62290763	+	.	.	.	.	.	.	.	.	.	.	.	.	.	.	.	.	.	.	.	.
7595	0,25	TMEM179B	NM_199337	chr11	62311449	62314448	+	.	.	.	.	.	.	.	.	.	.	.	.	.	.	.	.	.	.	.	.
5396	1	STX5	NM_001244666	chr11	62330907	62356139	-	.	.	.	.	.	.	.	.	.	.	.	.	.	.	.	.	.	.	.	.
7557	0,25	SLC3A2	NR_037193	chr11	62405746	62412931	+	.	.	.	.	.	.	.	.	.	.	.	.	.	.	.	.	.	.	.	.
6907	0,5	SLC22A6	NM_153276	chr11	62500644	62509071	-	.	.	.	.	.	.	.	.	.	.	.	.	.	.	.	.	.	.	.	.
7500	0,25	RARRES3	NM_004585	chr11	63060848	63070506	+	.	.	.	.	.	.	.	.	.	.	.	.	.	.	.	.	.	.	.	.
1952	1,75	PLA2G16	NM_007069	chr11	63098519	63138485	-	.	.	.	.	.	.	.	.	.	.	.	.	.	.	.	.	.	.	.	.
2384	1,5	RTN3	NM_006054	chr11	63205497	63283939	+	CL800061	chr11	63253406	+	.	.	.	.	.	.	.	.	.	.	.	.	.	.	.	.
2384	1,5	RTN3	NM_006054	chr11	63205497	63283939	+	CL799570	chr11	63257921	-	.	.	.	.	.	.	.	.	.	.	.	.	.	.	.	.
4357	1	MARK2	NM_001039469	chr11	63362975	63435068	+	.	.	.	.	.	.	.	.	.	.	.	.	.	.	.	.	.	.	.	.
6805	0,5	RCOR2	NM_173587	chr11	63435268	63440892	-	.	.	.	.	.	.	.	.	.	.	.	.	.	.	.	.	.	.	.	.
3443	1	COX8A	NM_004074	chr11	63498654	63500591	+	.	.	.	.	.	.	.	.	.	.	.	.	.	.	.	.	.	.	.	.
6358	0,5	FLRT1	NM_013280	chr11	63627937	63643221	+	.	.	.	.	.	.	.	.	.	.	.	.	.	.	.	.	.	.	.	.
5672	1	VEGFB	NM_003377	chr11	63758631	63763312	+	.	.	.	.	.	.	.	.	.	.	.	.	.	.	.	.	.	.	.	.
2336	1,5	PPP1R14B	NM_138689	chr11	63768526	63770989	-	.	.	.	.	.	.	.	.	.	.	.	.	.	.	.	.	.	.	.	.
1632	2	PLCB3	NM_001184883	chr11	63775570	63793500	+	.	.	.	.	.	.	.	.	.	.	.	.	.	.	.	.	.	.	.	.
1001	2,25	BAD	NM_004322	chr11	63793875	63808752	-	.	.	.	.	.	.	.	.	.	.	.	.	.	.	.	.	.	.	.	.
1390	2	ESRRA	NM_004451	chr11	63829619	63840786	+	CL800222	chr11	63832605	-	.	.	.	.	.	.	.	.	.	.	.	.	.	.	.	.

Table S2

tumor associated genes								HIV				MLV				MMTV				MMTV(SIN)				MMTV(SIN)arrest			
rank	score	gene symbol	RefSEq	chrom	txStart	txEnd	strand	integrant	chr	position	strand	integrant	chr	position	strand	integrant	chr	position	strand	integrant	chr	position	strand	integrant	chr	position	strand
1731	2	RPS6KA4	NM_003942	chr11	63883200	63896263	+	.	.	.	.	.	.	.	.	.	.	.	.	.	.	.	.	.	.	.	.
2288	1,5	NRXN2	NM_138734	chr11	64130221	64167363	-	.	.	.	.	.	.	.	.	.	.	.	.	.	.	.	.	.	.	.	.
2354	1,5	PYGM	NM_001164716	chr11	64270436	64284763	-	.	.	.	.	.	.	.	.	.	.	.	.	.	.	.	.	.	.	.	.
1519	2	MAP4K2	NM_004579	chr11	64313184	64327289	-	.	.	.	.	.	.	.	.	.	.	.	.	.	.	.	.	.	.	.	.
184	4,5	MEN1	NM_130800	chr11	64327561	64334611	-	.	.	.	.	.	.	.	.	.	.	.	.	.	.	.	.	.	.	.	.
1301	2	CDC42BPG	NM_017525	chr11	64348237	64368617	-	.	.	.	.	.	.	.	.	.	.	.	.	.	.	.	.	.	.	.	.
4923	1	PPP2R5B	NM_006244	chr11	64448718	64458526	+	.	.	.	.	.	.	.	.	.	.	.	.	.	.	.	.	.	.	.	.
5480	1	TM7SF2	NM_003273	chr11	64635916	64640283	+	.	.	.	.	.	.	.	.	.	.	.	.	.	.	.	.	.	.	.	.
6629	0,5	MRPL49	NM_004927	chr11	64646291	64651417	+	.	.	.	.	.	.	.	.	.	.	.	.	.	.	.	.	.	.	.	.
6129	0,5	CAPN1	NM_001198868	chr11	64705261	64736053	+	BH609652	chr11	64716058	-	.	.	.	.	.	.	.	.	.	.	.	.	.	.	.	.
1641	2	POLA2	NM_002689	chr11	64786007	64821664	+	CL800576	chr11	64806317	+	.	.	.	.	.	.	.	.	.	.	.	.	.	.	.	.
1641	2	POLA2	NM_002689	chr11	64786007	64821664	+	CL800120	chr11	64794478	+	.	.	.	.	.	.	.	.	.	.	.	.	.	.	.	.
1641	2	POLA2	NM_002689	chr11	64786007	64821664	+	CL799764	chr11	64801024	-	.	.	.	.	.	.	.	.	.	.	.	.	.	.	.	.
2655	1,25	MALAT1	NR_002819	chr11	65021808	65030515	+	.	.	.	.	.	.	.	.	.	.	.	.	.	.	.	.	.	.	.	.
675	3	SCYL1	NM_020680	chr11	65049123	65062758	+	.	.	.	.	.	.	.	.	.	.	.	.	.	.	.	.	.	.	.	.
4344	1	MAP3K11	NM_002419	chr11	65121801	65138296	-	CL799565	chr11	65126228	+	.	.	.	.	.	.	.	.	.	.	.	.	.	.	.	.
667	3	RELA	NM_021975	chr11	65177642	65187019	-	.	.	.	.	.	.	.	.	.	.	.	.	.	.	.	.	.	.	.	.
4462	1	MUS81	NM_025128	chr11	65384447	65390490	+	.	.	.	.	.	.	.	.	.	.	.	.	.	.	.	.	.	.	.	.
3651	1	EFEMP2	NM_016938	chr11	65390487	65396981	-	.	.	.	.	.	.	.	.	.	.	.	.	.	.	.	.	.	.	.	.
1046	2,25	FOSL1	NM_005438	chr11	65416267	65424573	-	.	.	.	.	.	.	.	.	.	.	.	.	.	.	.	.	.	.	.	.
6050	0,5	BANF1	NM_003860	chr11	65526125	65528193	+	.	.	.	.	.	.	.	.	.	.	.	.	.	.	.	.	.	.	.	.
2376	1,5	RIN1	NM_004292	chr11	65856117	65860576	-	.	.	.	.	.	.	.	.	.	.	.	.	.	.	.	.	.	.	.	.
7195	0,25	BRMS1	NM_001024957	chr11	65861379	65869158	-	.	.	.	.	.	.	.	.	.	.	.	.	.	.	.	.	.	.	.	.
2017	1,5	B3GNT1	NM_006876	chr11	65869418	65871737	-	.	.	.	.	.	.	.	.	.	.	.	.	.	.	.	.	.	.	.	.
4752	1	PELI3	NM_145065	chr11	65990911	66001384	+	.	.	.	.	.	.	.	.	.	.	.	.	.	.	.	.	.	.	.	.
6285	0,5	DPP3	NM_001256670	chr11	66004059	66033706	+	.	.	.	.	.	.	.	.	.	.	.	.	.	.	.	.	.	.	.	.
5073	1	RBM14	NM_006328	chr11	66140628	66153973	+	.	.	.	.	.	.	.	.	.	.	.	.	.	.	.	.	.	.	.	.
6968	0,5	SPTBN2	NM_006946	chr11	66209295	66245446	-	.	.	.	.	.	.	.	.	.	.	.	.	.	.	.	.	.	.	.	.
1599	2	PC	NM_000920	chr11	66372572	66482423	-	.	.	.	.	.	.	.	.	.	.	.	.	.	.	.	.	.	.	.	.
7000	0,5	SYT12	NM_001177880	chr11	66546765	66574910	+	.	.	.	.	.	.	.	.	.	.	.	.	.	.	.	.	.	.	.	.
1722	2	RHOD	NM_014578	chr11	66580864	66596064	+	.	.	.	.	.	.	.	.	.	.	.	.	.	.	.	.	.	.	.	.
6513	0,5	KDM2A	NM_012308	chr11	66643315	66782126	+	CL529537	chr11	66688150	-	.	.	.	.	.	.	.	.	.	.	.	.	.	.	.	.
6513	0,5	KDM2A	NM_012308	chr11	66643315	66782126	+	BH609655	chr11	66706356	-	.	.	.	.	.	.	.	.	.	.	.	.	.	.	.	.
6513	0,5	KDM2A	NM_012308	chr11	66643315	66782126	+	CL800353	chr11	66702704	+	.	.	.	.	.	.	.	.	.	.	.	.	.	.	.	.
6513	0,5	KDM2A	NM_012308	chr11	66643315	66782126	+	BH609656	chr11	66724976	-	.	.	.	.	.	.	.	.	.	.	.	.	.	.	.	.
6513	0,5	KDM2A	NM_012308	chr11	66643315	66782126	+	CL529083	chr11	66687889	+	.	.	.	.	.	.	.	.	.	.	.	.	.	.	.	.
6513	0,5	KDM2A	NM_012308	chr11	66643315	66782126	+	CL800342	chr11	66744113	+	.	.	.	.	.	.	.	.	.	.	.	.	.	.	.	.
6513	0,5	KDM2A	NM_012308	chr11	66643315	66782126	+	AY516895.1	chr11	66724843	+	.	.	.	.	.	.	.	.	.	.	.	.	.	.	.	.
6513	0,5	KDM2A	NM_012308	chr11	66643315	66782126	+	AY517198.1	chr11	66734687	+	.	.	.	.	.	.	.	.	.	.	.	.	.	.	.	.
6513	0,5	KDM2A	NM_012308	chr11	66643315	66782126	+	CL800125	chr11	66725366	+	.	.	.	.	.	.	.	.	.	.	.	.	.	.	.	.
2915	1	ADRBK1	NM_001619	chr11	66790480	66810605	+	CL799722	chr11	66794660	-	AY516659	chr11	66797595	+	.	.	.	.	.	.	.	.	.	.	.	.
6973	0,5	SSH3	NM_017857	chr11	66827494	66836654	+	.	.	.	.	.	.	.	.	.	.	.	.	.	.	.	.	502_107_1	chr11	66835115	+
922	2,5	POLD4	NR_046411	chr11	66874811	66877643	-	.	.	.	.	.	.	.	.	.	.	.	.	.	.	.	.	.	.	.	.
6187	0,5	CLCF1	NM_001166212	chr11	66888214	66898224	-	.	.	.	.	.	.	.	.	.	.	.	.	.	.	.	.	.	.	.	.
2743	1,25	RAD9A	NM_004584	chr11	66915998	66922459	+	CL800759	chr11	66921418	+	.	.	.	.	.	.	.	.	.	.	.	.	.	.	.	.
1667	2	PPP1CA	NM_001008709	chr11	66922227	66925952	-	.	.	.	.	.	.	.	.	.	.	.	.	.	.	.	.	.	.	.	.
672	3	RPS6KB2	NM_003952	chr11	66952510	66959455	+	.	.	.	.	.	.	.	.	.	.	.	.	.	.	.	.	.	.	.	.
5004	1	PTPRCAP	NM_005608	chr11	66959556	66961729	-	.	.	.	.	.	.	.	.	.	.	.	.	.	.	.	.	.	.	.	.
471	3,25	GSTP1	NM_000852	chr11	67107641	67110700	+	.	.	.	.	.	.	.	.	.	.	.	.	.	.	.	.	.	.	.	.
4552	1	NDUFV1	NM_001166102	chr11	67130898	67136588	+	.	.	.	.	.	.	.	.	.	.	.	.	.	.	.	.	.	.	.	.
4647	1	NUDT8	NM_001243750	chr11	67151985	67153984	-	.	.	.	.	.	.	.	.	.	.	.	.	.	.	.	.	.	.	.	.

Table S2

tumor associated genes								HIV				MLV				MMTV				MMTV(SIN)				MMTV(SIN)arrest			
rank	score	gene symbol	RefSEq	chrom	txStart	txEnd	strand	integrant	chr	position	strand	integrant	chr	position	strand	integrant	chr	position	strand	integrant	chr	position	strand	integrant	chr	position	strand
2898	1	ACY3	NM_080658	chr11	67166601	67174706	-	.	.	.	.	.	.	.	.	.	.	.	.	.	.	.	.	.	.	.	.
1237	2	ALDH3B2	NM_000695	chr11	67186208	67198678	-	.	.	.	.	.	.	.	.	.	.	.	.	.	.	.	.	.	.	.	.
1994	1,5	ALDH3B1	NM_001161473	chr11	67532623	67553319	+	.	.	.	.	.	.	.	.	.	.	.	.	.	.	.	.	.	.	.	.
4551	1	NDUFS8	NM_002496	chr11	67554659	67560690	+	.	.	.	.	.	.	.	.	.	.	.	.	.	.	.	.	.	.	.	.
2433	1,5	TCIRG1	NM_006019	chr11	67563037	67574942	+	.	.	.	.	.	.	.	.	.	.	.	.	.	.	.	.	.	.	.	.
1309	2	CHKA	NM_212469	chr11	67576901	67645434	-	CL800050	chr11	67625407	-	.	.	.	.	.	.	.	.	.	.	.	.	.	.	.	.
5413	1	SUV420H1	NM_016028	chr11	67689761	67737360	-	CL529084	chr11	67705713	+	.	.	.	.	.	.	.	.	.	.	.	.	.	.	.	.
884	2,5	LRP5	NM_002335	chr11	67836683	67973319	+	.	.	.	.	AY515990	chr11	67965764	-	.	.	.	.	.	.	.	.	.	.	.	.
1324	2	CPT1A	NM_001876	chr11	68278926	68365975	-	.	.	.	.	.	.	.	.	.	.	.	.	.	.	.	.	.	.	.	.
53	7	CCND1	NM_053056	chr11	69165053	69178423	+	.	.	.	.	.	.	.	.	.	.	.	.	.	.	.	.	.	.	.	.
2586	1,25	FGF4	NM_002007	chr11	69296977	69299352	-	.	.	.	.	.	.	.	.	.	.	.	.	.	.	.	.	.	.	.	.
2585	1,25	FGF3	NM_005247	chr11	69333916	69343129	-	.	.	.	.	.	.	.	.	.	.	.	.	.	.	.	.	.	.	.	.
465	3,25	FADD	NM_003824	chr11	69726916	69731156	+	.	.	.	.	.	.	.	.	.	.	.	.	.	.	.	.	.	.	.	.
4896	1	PPFIA1	NR_045286	chr11	69794453	69908255	+	.	.	.	.	.	.	.	.	.	.	.	.	.	.	.	.	.	.	.	.
3486	1	CTTN	NM_138565	chr11	69922259	69960338	+	.	.	.	.	.	.	.	.	.	.	.	.	.	.	.	.	.	.	.	.
6887	0,5	SHANK2	NM_012309	chr11	69991608	70613456	-	.	.	.	.	.	.	.	.	.	.	.	.	.	.	.	.	.	.	.	.
3572	1	DHCR7	NM_001163817	chr11	70823104	70837125	-	.	.	.	.	.	.	.	.	.	.	.	.	.	.	.	.	.	.	.	.
4488	1	NADSYN1	NM_018161	chr11	70841864	70890229	+	.	.	.	.	.	.	.	.	.	.	.	.	.	.	.	.	.	.	.	.
1123	2,25	NUMA1	NM_006185	chr11	71391558	71469221	-	AY517242.1	chr11	71445357	+	.	.	.	.	.	.	.	.	.	.	.	.	.	.	.	.
3794	1	FOLR2	NM_001113535	chr11	71605466	71610642	+	.	.	.	.	.	.	.	.	.	.	.	.	.	.	.	.	.	.	.	.
387	3,5	INPPL1	NM_001567	chr11	71613529	71627836	+	.	.	.	.	.	.	.	.	.	.	.	.	.	.	.	.	.	.	.	.
1603	2	PDE2A	NM_001243784	chr11	71964831	72031152	-	.	.	.	.	.	.	.	.	.	.	.	.	.	.	.	.	.	.	.	.
6711	0,5	P2RY2	NM_002564	chr11	72606991	72625043	+	.	.	.	.	.	.	.	.	.	.	.	.	.	.	.	.	.	.	.	.
6753	0,5	PLEKHB1	NM_021200	chr11	73034870	73051512	+	.	.	.	.	.	.	.	.	.	.	.	.	.	.	.	.	.	.	.	.
5040	1	RAB6A	NM_002869	chr11	73064330	73149849	-	.	.	.	.	.	.	.	.	.	.	.	.	.	.	.	.	.	.	.	.
5973	0,75	UCP2	NM_003355	chr11	73363363	73371537	-	.	.	.	.	.	.	.	.	.	.	.	.	.	.	.	.	.	.	.	.
651	3	PPME1	NM_016147	chr11	73560015	73643396	+	.	.	.	.	.	.	.	.	.	.	.	.	.	.	.	.	.	.	.	.
4682	1	P4HA3	NM_182904	chr11	73655349	73700347	-	.	.	.	.	.	.	.	.	.	.	.	.	.	.	.	.	.	.	.	.
6737	0,5	PGM2L1	NM_173582	chr11	73719008	73787150	-	.	.	.	.	.	.	.	.	.	.	.	.	.	.	.	.	.	.	.	.
1643	2	POLD3	NM_006591	chr11	73981222	74031753	+	.	.	.	.	.	.	.	.	.	.	.	.	.	.	.	.	.	.	.	.
4569	1	NEU3	NM_006656	chr11	74377597	74396391	+	.	.	.	.	.	.	.	.	.	.	.	.	1594_291	chr11	74382900	+	.	.	.	.
2006	1,5	ARRB1	NM_004041	chr11	74648813	74740523	-	.	.	.	.	.	.	.	.	.	.	.	.	.	.	.	.	.	.	.	.
5164	1	RPS3	NM_001005	chr11	74788182	74795605	+	.	.	.	.	.	.	.	.	.	.	.	.	.	.	.	.	.	.	.	.
6877	0,5	SERPINH1	NM_001207014	chr11	74950748	74961497	+	.	.	.	.	.	.	.	.	.	.	.	.	.	.	.	.	.	.	.	.
3565	1	DGAT2	NM_032564	chr11	75157425	75190229	+	.	.	.	.	.	.	.	.	.	.	.	.	.	.	.	.	.	.	.	.
5664	1	UVRAG	NM_003369	chr11	75203859	75532930	+	CL800129	chr11	75497533	-	.	.	.	.	.	.	.	.	.	.	.	.	.	.	.	.
5664	1	UVRAG	NM_003369	chr11	75203859	75532930	+	CL529435	chr11	75424459	+	.	.	.	.	.	.	.	.	.	.	.	.	.	.	.	.
701	3	WNT11	NM_004626	chr11	75575017	75595222	-	.	.	.	.	.	.	.	.	.	.	.	.	.	.	.	.	.	.	.	.
4946	1	PRKRIR	NM_004705	chr11	75738651	75769528	-	.	.	.	.	.	.	.	.	.	.	.	.	.	.	.	.	.	.	.	.
3168	1	C11orf30	NM_020193	chr11	75833716	75940237	+	AY517369.1	chr11	75854544	-	.	.	.	.	.	.	.	.	.	.	.	.	.	.	.	.
3168	1	C11orf30	NM_020193	chr11	75833716	75940237	+	CL799828	chr11	75895011	-	.	.	.	.	.	.	.	.	.	.	.	.	.	.	.	.
3168	1	C11orf30	NM_020193	chr11	75833716	75940237	+	BH609661	chr11	75908212	-	.	.	.	.	.	.	.	.	.	.	.	.	.	.	.	.
3168	1	C11orf30	NM_020193	chr11	75833716	75940237	+	CL800612	chr11	75911140	+	.	.	.	.	.	.	.	.	.	.	.	.	.	.	.	.
3168	1	C11orf30	NM_020193	chr11	75833716	75940237	+	CL799679	chr11	75844905	-	.	.	.	.	.	.	.	.	.	.	.	.	.	.	.	.
2878	1	ACER3	NM_018367	chr11	76249564	76412498	+	.	.	.	.	.	.	.	.	.	.	.	.	.	.	.	.	.	.	.	.
3115	1	B3GNT6	NM_138706	chr11	76423082	76430653	+	.	.	.	.	.	.	.	.	.	.	.	.	.	.	.	.	.	.	.	.
633	3	PAK1	NM_002576	chr11	76710707	76862756	-	.	.	.	.	.	.	.	.	.	.	.	.	.	.	.	.	.	.	.	.
4545	1	NDUFC2	NM_001204055	chr11	77457040	77468913	-	.	.	.	.	.	.	.	.	.	.	.	.	.	.	.	.	.	.	.	.
2963	1	ALG8	NM_024079	chr11	77489635	77528347	-	.	.	.	.	.	.	.	.	.	.	.	.	.	.	.	.	.	.	.	.
6369	0,5	GAB2	NM_012296	chr11	77603983	77730574	-	.	.	.	.	.	.	.	.	.	.	.	.	.	.	.	.	.	.	.	.
4497	1	NARS2	NM_001243251	chr11	77824654	77963557	-	CL799558	chr11	77918952	-	.	.	.	.	.	.	.	.	.	.	.	.	.	.	.	.

Table S2

tumor associated genes								HIV				MLV				MMTV				MMTV(SIN)				MMTV(SIN)arrest			
rank	score	gene symbol	RefSEq	chrom	txStart	txEnd	strand	integrant	chr	position	strand	integrant	chr	position	strand	integrant	chr	position	strand	integrant	chr	position	strand	integrant	chr	position	strand
5028	1	RAB30	NM_014488	chr11	82370125	82460532	-	.	.	.	.	.	.	.	.	.	.	.	.	49_1_2031_7	chr11	82430209	+	.	.	.	.
6723	0,5	PCF11	NM_015885	chr11	82545784	82574483	+	.	.	.	.	.	.	.	.	.	.	.	.	.	.	.	.	.	.	.	.
3588	1	DLG2	NM_001142702	chr11	82843703	83071116	-	.	.	.	.	.	.	.	.	_285_1_186	chr11	84935783	+	.	.	.	.	.	.	.	.
1617	2	PICALM	NM_001008660	chr11	85345861	85457787	-	.	.	.	.	.	.	.	.	.	.	.	.	.	.	.	.	.	.	.	.
3647	1	EED	NM_152991	chr11	85633453	85667433	+	BH609662	chr11	85656536	-	.	.	.	.	.	.	.	.	.	.	.	.	.	.	.	.
2671	1,25	ME3	NM_001014811	chr11	85829797	86060888	-	.	.	.	.	.	.	.	.	.	.	.	.	.	.	.	.	.	.	.	.
6776	0,5	PRSS23	NM_007173	chr11	86189138	86199921	+	.	.	.	.	.	.	.	.	.	.	.	.	.	.	.	.	.	.	.	.
7320	0,25	FZD4	NM_012193	chr11	86334364	86344088	-	.	.	.	.	.	.	.	.	.	.	.	.	.	.	.	.	.	.	.	.
7591	0,25	TMEM135	NM_001168724	chr11	86426533	86717524	+	.	.	.	.	.	.	.	.	.	.	.	.	.	.	.	.	.	.	.	.
1710	2	RAB38	NM_022337	chr11	87486062	87548283	-	CL799996	chr11	87490086	-	.	.	.	.	.	.	.	.	.	.	.	.	.	.	.	.
6228	0,5	CTSC	NM_148170	chr11	87693628	87710589	-	AY516982.1	chr11	87693382	+	.	.	.	.	.	.	.	.	.	.	.	.	.	.	.	.
1433	2	GRM5	NM_000842	chr11	87877391	88436464	-	.	.	.	.	.	.	.	.	.	.	.	.	.	.	.	.	.	.	.	.
5594	1	TYR	NM_000372	chr11	88550687	88668575	+	.	.	.	.	.	.	.	.	.	.	.	.	.	.	.	.	.	.	.	.
5941	0,75	NOX4	NM_016931	chr11	88697169	88864301	-	.	.	.	.	.	.	.	.	.	.	.	.	.	.	.	.	.	.	.	.
7316	0,25	FOLH1B	NM_153696	chr11	89032112	89071534	+	.	.	.	.	.	.	.	.	.	.	.	.	.	.	.	.	.	.	.	.
3169	1	C11orf54	NM_014039	chr11	93114440	93135895	+	CL529088	chr11	93133660	+	.	.	.	.	.	.	.	.	.	.	.	.	.	.	.	.
6403	0,5	GPR83	NM_016540	chr11	93750124	93774233	-	.	.	.	.	.	.	.	.	.	.	.	.	.	.	.	.	.	.	.	.
108	5,5	MRE11A	NM_005591	chr11	93790114	93866688	-	.	.	.	.	.	.	.	.	.	.	.	.	.	.	.	.	.	.	.	.
3819	1	FUT4	NM_002033	chr11	93916664	93922712	+	.	.	.	.	.	.	.	.	.	.	.	.	.	.	.	.	.	.	.	.
4210	1	KDM4D	NM_018039	chr11	94346492	94372324	+	.	.	.	.	.	.	.	.	.	.	.	.	.	.	.	.	.	.	.	.
3732	1	FAM76B	NM_144664	chr11	95141753	95162602	-	.	.	.	.	.	.	.	.	.	.	.	.	.	.	.	.	.	.	.	.
4458	1	MTMR2	NM_016156	chr11	95205691	95297019	-	.	.	.	.	.	.	.	.	_457_1_326	chr11	95211320	-	.	.	.	.	.	.	.	.
614	3	MAML2	NM_032427	chr11	95351087	95715992	-	CL529592	chr11	95356730	+	AY516486	chr11	95358698	-	.	.	.	.	.	.	.	.	.	.	.	.
1318	2	CNTN5	NM_001243271	chr11	98396915	99685643	+	.	.	.	.	AY516747	chr11	99150614	+	.	.	.	.	66_1_2179_8	chr11	98623705	+	1151_132_1	chr11	99040159	+
339	3,75	PGR	NM_000926	chr11	100405564	100505004	-	.	.	.	.	.	.	.	.	.	.	.	.	.	.	.	.	.	.	.	.
2463	1,5	YAP1	NM_001195044	chr11	101486401	101609364	+	.	.	.	.	.	.	.	.	.	.	.	.	.	.	.	.	.	.	.	.
316	3,75	BIRC3	NM_001165	chr11	101693390	101715344	+	.	.	.	.	.	.	.	.	.	.	.	.	.	.	.	.	.	.	.	.
7189	0,25	BIRC2	NM_001256163	chr11	101723314	101754611	+	.	.	.	.	.	.	.	.	.	.	.	.	.	.	.	.	.	.	.	.
5487	1	TMEM123	NM_052932	chr11	101772265	101828985	-	.	.	.	.	.	.	.	.	.	.	.	.	.	.	.	.	.	.	.	.
5934	0,75	MMP7	NM_002423	chr11	101896448	101906688	-	.	.	.	.	.	.	.	.	.	.	.	.	.	.	.	.	.	.	.	.
4416	1	MMP20	NM_004771	chr11	101952775	102001273	-	.	.	.	.	.	.	.	.	.	.	.	.	.	.	.	.	.	.	.	.
4417	1	MMP8	NM_002424	chr11	102087735	102100895	-	.	.	.	.	.	.	.	.	.	.	.	.	.	.	.	.	.	.	.	.
2259	1,5	MMP10	NM_002425	chr11	102146442	102156569	-	.	.	.	.	.	.	.	.	.	.	.	.	.	.	.	.	.	.	.	.
2677	1,25	MMP1	NM_002421	chr11	102165850	102174176	-	.	.	.	.	.	.	.	.	.	.	.	.	.	.	.	.	.	.	.	.
2679	1,25	MMP3	NM_002422	chr11	102211737	102219552	-	.	.	.	.	.	.	.	.	.	.	.	.	.	.	.	.	.	.	.	.
7430	0,25	MMP12	NM_002426	chr11	102238673	102250974	-	.	.	.	.	.	.	.	.	.	.	.	.	.	.	.	.	.	.	.	.
4415	1	MMP13	NM_002427	chr11	102318930	102331673	-	.	.	.	.	.	.	.	.	.	.	.	.	.	.	.	.	.	.	.	.
2313	1,5	PDGFD	NM_033135	chr11	103283123	103540237	-	CL800458	chr11	103524484	-	.	.	.	.	.	.	.	.	.	.	.	.	.	.	.	.
801	2,5	CASP4	NM_033306	chr11	104318803	104332632	-	.	.	.	.	.	.	.	.	.	.	.	.	.	.	.	.	.	.	.	.
6132	0,5	CASP5	NM_001136112	chr11	104370176	104399105	-	.	.	.	.	.	.	.	.	.	.	.	.	.	.	.	.	.	.	.	.
1290	2	CASP1	NM_001257118	chr11	104401446	104411094	-	.	.	.	.	.	.	.	.	.	.	.	.	.	.	.	.	.	.	.	.
3276	1	CARD16	NM_001017534	chr11	104417262	104421261	-	.	.	.	.	.	.	.	.	.	.	.	.	.	.	.	.	.	.	.	.
1431	2	GRIA4	NM_000829	chr11	104986009	105358029	+	.	.	.	.	.	.	.	.	.	.	.	.	.	.	.	.	.	.	.	.
2847	1	AASDHPPT	NM_015423	chr11	105453501	105474629	+	.	.	.	.	.	.	.	.	.	.	.	.	.	.	.	.	.	.	.	.
1438	2	GUCY1A2	NM_000855	chr11	106049947	106394381	-	CL529092	chr11	106069344	-	.	.	.	.	.	.	.	.	.	.	.	.	.	.	.	.
6306	0,5	ELMOD1	NM_001130037	chr11	106967026	107042715	+	.	.	.	.	.	.	.	.	.	.	.	.	.	.	.	.	.	.	.	.
5964	0,75	SLN	NM_003063	chr11	107083310	107087997	-	.	.	.	.	.	.	.	.	.	.	.	.	.	.	.	.	.	.	.	.
2876	1	ACAT1	NM_000019	chr11	107497467	107524101	+	.	.	.	.	.	.	.	.	.	.	.	.	.	.	.	.	.	.	.	.
4598	1	NPAT	NM_002519	chr11	107533327	107598575	-	.	.	.	.	.	.	.	.	.	.	.	.	.	.	.	.	.	.	.	.
10	11,25	ATM	NM_000051	chr11	107598768	107745036	+	.	.	.	.	.	.	.	.	.	.	.	.	.	.	.	.	.	.	.	.
1031	2,25	DDX10	NM_004398	chr11	108041025	108316858	+	CL529685	chr11	108128790	+	.	.	.	.	.	.	.	.	.	.	.	.	.	.	.	.

Table S2

tumor associated genes								HIV				MLV				MMTV				MMTV(SIN)				MMTV(SIN)arrest			
rank	score	gene symbol	RefSEq	chrom	txStart	txEnd	strand	integrant	chr	position	strand	integrant	chr	position	strand	integrant	chr	position	strand	integrant	chr	position	strand	integrant	chr	position	strand
7509	0,25	RDX	NM_001260496	chr11	109571495	109672647	-	.	.	.	.	.	.	.	.	.	.	.	.	.	.	.	.	.	.	.	.
929	2,5	POU2AF1	NM_006235	chr11	110728189	110755367	-	.	.	.	.	.	.	.	.	.	.	.	.	.	.	.	.	.	.	.	.
681	3	SIK2	NM_015191	chr11	110978379	111102842	+	BH609663	chr11	111026988	+	.	.	.	.	.	.	.	.	.	.	.	.	.	.	.	.
226	4,25	PPP2R1B	NM_002716	chr11	111113814	111142379	-	.	.	.	.	.	.	.	.	.	.	.	.	.	.	.	.	.	.	.	.
2964	1	ALG9	NM_001077692	chr11	111158128	111247214	-	.	.	.	.	.	.	.	.	.	.	.	.	.	.	.	.	.	.	.	.
7255	0,25	CRYAB	NM_001885	chr11	111284559	111287683	-	.	.	.	.	.	.	.	.	.	.	.	.	.	.	.	.	.	.	.	.
1922	1,75	HSPB2	NM_001541	chr11	111288669	111290027	+	.	.	.	.	.	.	.	.	.	.	.	.	.	.	.	.	.	.	.	.
5899	0,75	DIXDC1	NM_001037954	chr11	111313136	111398517	+	.	.	.	.	.	.	.	.	.	.	.	.	.	.	.	.	.	.	.	.
3586	1	DLAT	NM_001931	chr11	111400747	111440212	+	.	.	.	.	.	.	.	.	.	.	.	.	.	.	.	.	.	.	.	.
304	4	SDHD	NM_003002	chr11	111462780	111471728	+	.	.	.	.	.	.	.	.	.	.	.	.	.	.	.	.	.	.	.	.
1924	1,75	IL18	NM_001243211	chr11	111519183	111540050	-	.	.	.	.	.	.	.	.	.	.	.	.	.	.	.	.	.	.	.	.
5006	1	PTS	NM_000317	chr11	111602297	111609905	+	.	.	.	.	.	.	.	.	.	.	.	.	.	.	.	.	.	.	.	.
98	5,75	NCAM1	NM_181351	chr11	112337178	112654368	+	.	.	.	.	.	.	.	.	.	.	.	.	.	.	.	.	.	.	.	.
2987	1	ANKK1	NM_178510	chr11	112763722	112776350	+	.	.	.	.	.	.	.	.	.	.	.	.	.	.	.	.	.	.	.	.
2572	1,25	DRD2	NM_000795	chr11	112785526	112851211	-	.	.	.	.	.	.	.	.	.	.	.	.	.	.	.	.	.	.	.	.
2839	1,25	ZW10	NM_004724	chr11	113109114	113149695	-	.	.	.	.	.	.	.	.	.	.	.	.	.	.	.	.	.	.	.	.
5656	1	USP28	NM_020886	chr11	113173806	113251466	-	CL800147	chr11	113220191	-	.	.	.	.	.	.	.	.	.	.	.	.	.	.	.	.
441	3,5	ZBTB16	NM_006006	chr11	113435640	113626607	+	.	.	.	.	.	.	.	.	.	.	.	.	.	.	.	.	.	.	.	.
1945	1,75	NNMT	NM_006169	chr11	113671744	113688448	+	.	.	.	.	.	.	.	.	.	.	.	.	.	.	.	.	.	.	.	.
2529	1,25	CADM1	NM_014333	chr11	114549554	114880451	-	CL529094	chr11	114841007	+	.	.	.	.	.	.	.	.	.	.	.	.	.	.	.	.
7166	0,25	APOA1	NM_000039	chr11	116211678	116213548	-	.	.	.	.	.	.	.	.	.	.	.	.	.	.	.	.	.	.	.	.
286	4	PAFAH1B2	NM_001184746	chr11	116520209	116552341	+	.	.	.	.	.	.	.	.	.	.	.	.	.	.	.	.	.	.	.	.
7006	0,5	TAGLN	NM_003186	chr11	116575249	116580718	+	.	.	.	.	.	.	.	.	.	.	.	.	.	.	.	.	.	.	.	.
913	2,5	PCSK7	NM_004716	chr11	116580997	116608021	-	.	.	.	.	.	.	.	.	.	.	.	.	.	.	.	.	.	.	.	.
815	2,5	DSCAML1	NM_020693	chr11	116803698	117173186	-	.	.	.	.	.	.	.	.	.	.	.	.	.	.	.	.	.	.	.	.
6366	0,5	FXYD6	NM_001164831	chr11	117212900	117253411	-	.	.	.	.	.	.	.	.	.	.	.	.	.	.	.	.	.	.	.	.
6858	0,5	SCN4B	NM_001142348	chr11	117509301	117528840	-	.	.	.	.	.	.	.	.	.	.	.	.	.	.	.	.	.	.	.	.
6857	0,5	SCN2B	NM_004588	chr11	117538728	117552547	-	.	.	.	.	.	.	.	.	.	.	.	.	.	.	.	.	.	.	.	.
6150	0,5	CD3E	NM_000733	chr11	117680504	117692100	+	.	.	.	.	.	.	.	.	.	.	.	.	.	.	.	.	.	.	.	.
2011	1,5	ATP5L	NM_006476	chr11	117777313	117785772	+	.	.	.	.	.	.	.	.	.	.	.	.	.	.	.	.	.	.	.	.
278	4	MLL	NM_001197104	chr11	117812414	117902749	+	AY516902.1	chr11	117825151	-	.	.	.	.	.	.	.	.	.	.	.	.	.	.	.	.
5546	1	TREH	NM_007180	chr11	118034151	118055591	-	.	.	.	.	.	.	.	.	.	.	.	.	.	.	.	.	.	.	.	.
1032	2,25	DDX6	NM_001257191	chr11	118123681	118166867	-	.	.	.	.	.	.	.	.	.	.	.	.	.	.	.	.	.	.	.	.
4056	1	HMBS	NM_001258208	chr11	118460796	118469469	+	.	.	.	.	.	.	.	.	.	.	.	.	.	.	.	.	.	.	.	.
2611	1,25	H2AFX	NM_002105	chr11	118469794	118471387	-	.	.	.	.	.	.	.	.	.	.	.	.	.	.	.	.	.	.	.	.
563	3	DPAGT1	NM_001382	chr11	118472422	118477995	-	.	.	.	.	.	.	.	.	.	.	.	.	.	.	.	.	.	.	.	.
6098	0,5	C2CD2L	NM_014807	chr11	118483302	118493037	+	.	.	.	.	.	.	.	.	.	.	.	.	.	.	.	.	.	.	.	.
2858	1	ABCG4	NM_022169	chr11	118524959	118538584	+	.	.	.	.	.	.	.	.	.	.	.	.	.	.	.	.	.	.	.	.
87	6	CBL	NM_005188	chr11	118582195	118684069	+	CL800684	chr11	118606109	+	.	.	.	.	.	.	.	.	.	.	.	.	.	.	.	.
87	6	CBL	NM_005188	chr11	118582195	118684069	+	BH609666	chr11	118605970	+	.	.	.	.	.	.	.	.	.	.	.	.	.	.	.	.
5554	1	TRIM29	NM_012101	chr11	119487203	119514073	-	.	.	.	.	.	.	.	.	.	.	.	.	.	.	.	.	.	.	.	.
4883	1	POU2F3	NM_014352	chr11	119616112	119695863	+	.	.	.	.	.	.	.	.	.	.	.	.	.	.	.	.	.	.	.	.
1254	2	ARHGEF12	NM_001198665	chr11	119712827	119865855	+	AY517294.1	chr11	119716024	+	.	.	.	.	.	.	.	.	.	.	.	.	.	.	.	.
5454	1	TECTA	NM_005422	chr11	120478584	120566725	+	.	.	.	.	.	.	.	.	.	.	.	.	.	.	.	.	.	.	.	.
5191	1	SC5DL	NM_006918	chr11	120668597	120689329	+	.	.	.	.	.	.	.	.	.	.	.	.	.	.	.	.	.	.	.	.
5317	1	SORL1	NM_003105	chr11	120828121	121009681	+	.	.	.	.	.	.	.	.	.	.	.	.	.	.	.	.	.	.	.	.
729	2,75	HSPA8	NM_006597	chr11	122433409	122438111	-	.	.	.	.	.	.	.	.	.	.	.	.	.	.	.	.	.	.	.	.
1740	2	SCN3B	NM_001040151	chr11	123005104	123030525	-	.	.	.	.	.	.	.	.	.	.	.	.	.	.	.	.	.	.	.	.
5683	1	VWA5A	NM_014622	chr11	123491320	123522828	+	.	.	.	.	.	.	.	.	.	.	.	.	.	.	.	.	.	.	.	.
4669	1	OR8D2	NM_001002918	chr11	123694367	123695303	-	.	.	.	.	.	.	.	.	.	.	.	.	.	.	.	.	.	.	.	.
5322	1	SPA17	NM_017425	chr11	124048949	124069897	+	.	.	.	.	.	.	.	.	.	.	.	.	.	.	.	.	.	.	.	.

Table S2

tumor associated genes								HIV				MLV				MMTV				MMTV(SIN)				MMTV(SIN)arrest			
rank	score	gene symbol	RefSEq	chrom	txStart	txEnd	strand	integrant	chr	position	strand	integrant	chr	position	strand	integrant	chr	position	strand	integrant	chr	position	strand	integrant	chr	position	strand
4621	1	NRGN	NM_006176	chr11	124115038	124122312	+	.	.	.	.	.	.	.	.	.	.	.	.	.	.	.	.	.	.	.	.
75	6,25	CHEK1	NM_001274	chr11	125001460	125032252	+	.	.	.	.	.	.	.	.	.	.	.	.	.	.	.	.	.	.	.	.
6260	0,5	DDX25	NM_013264	chr11	125279470	125298215	+	.	.	.	.	.	.	.	.	.	.	.	.	.	.	.	.	.	.	.	.
5359	1	ST3GAL4	NM_001254757	chr11	125730749	125789746	+	CL529095	chr11	125746703	+	.	.	.	.	.	.	.	.	.	.	.	.	.	.	.	.
6536	0,5	KIRREL3	NM_001161707	chr11	125806046	126375976	-	.	.	.	.	.	.	.	.	.	.	.	.	.	.	.	.	.	.	.	.
568	3	ETS1	NM_001143820	chr11	127833865	127962663	-	.	.	.	.	.	.	.	.	.	.	.	.	.	.	.	.	.	.	.	.
576	3	FLI1	NM_001167681	chr11	128069020	128188372	+	CL800606	chr11	128188310	+	.	.	.	.	.	.	.	.	.	.	.	.	.	.	.	.
6499	0,5	KCNJ1	NM_153765	chr11	128213124	128242478	-	.	.	.	.	.	.	.	.	.	.	.	.	.	.	.	.	.	.	.	.
5525	1	TP53AIP1	NM_001195195	chr11	128309836	128318504	-	.	.	.	.	.	.	.	.	.	.	.	.	.	.	.	.	.	.	.	.
3127	1	BARX2	NM_003658	chr11	128751090	128827384	+	.	.	.	.	.	.	.	.	.	.	.	.	.	.	.	.	.	.	.	.
3002	1	APLP2	NM_001142276	chr11	129444925	129519916	+	.	.	.	.	.	.	.	.	.	.	.	.	.	.	.	.	.	.	.	.
1969	1,75	ST14	NM_021978	chr11	129534891	129585467	+	.	.	.	.	.	.	.	.	.	.	.	.	.	.	.	.	.	.	.	.
7153	0,25	ADAMTS8	NM_007037	chr11	129780027	129803749	-	.	.	.	.	.	.	.	.	.	.	.	.	.	.	.	.	.	.	.	.
2299	1,5	OPCML	NM_002545	chr11	131790084	132318247	-	.	.	.	.	.	.	.	.	.	.	.	.	.	.	.	.	.	.	.	.
7380	0,25	JAM3	NM_001205329	chr11	133444029	133526862	+	.	.	.	.	.	.	.	.	.	.	.	.	.	.	.	.	.	.	.	.
2868	1	ACAD8	NM_014384	chr11	133628643	133640956	+	.	.	.	.	.	.	.	.	.	.	.	.	.	.	.	.	.	.	.	.
710	2,75	B3GAT1	NM_054025	chr11	133753607	133787022	-	.	.	.	.	.	.	.	.	.	.	.	.	.	.	.	.	.	.	.	.
4162	1	IQSEC3	NM_001170738	chr12	46309	157886	+	.	.	.	.	.	.	.	.	.	.	.	.	.	.	.	.	.	.	.	.
6922	0,5	SLC6A12	NM_001122847	chr12	169503	193124	-	.	.	.	.	.	.	.	.	.	.	.	.	.	.	.	.	.	.	.	.
5283	1	SLC6A13	NM_001190997	chr12	200047	242300	-	.	.	.	.	.	.	.	.	.	.	.	.	.	.	.	.	.	.	.	.
2216	1,5	KDM5A	NM_001042603	chr12	259483	368881	-	.	.	.	.	.	.	.	.	.	.	.	.	.	.	.	.	.	.	.	.
5701	1	WNK1	NM_018979	chr12	732349	890879	+	CL800805	chr12	834116	+	.	.	.	.	.	.	.	.	.	.	.	.	.	.	.	.
5701	1	WNK1	NM_018979	chr12	732349	890879	+	CL529097	chr12	840983	-	.	.	.	.	.	.	.	.	.	.	.	.	.	.	.	.
5701	1	WNK1	NM_018979	chr12	732349	890879	+	CL800054	chr12	803375	-	.	.	.	.	.	.	.	.	.	.	.	.	.	.	.	.
5701	1	WNK1	NM_018979	chr12	732349	890879	+	CL800044	chr12	806116	+	.	.	.	.	.	.	.	.	.	.	.	.	.	.	.	.
661	3	RAD52	NM_134424	chr12	891515	929124	-	.	.	.	.	.	.	.	.	.	.	.	.	.	.	.	.	.	.	.	.
1387	2	ERC1	NR_027946	chr12	970664	1475360	+	CL799688	chr12	985692	+	AY516139	chr12	1298674	-	.	.	.	.	112_175	chr12	1054015	+	.	.	.	.
1860	2	WNT5B	NM_032642	chr12	1608672	1626639	+	.	.	.	.	.	.	.	.	.	.	.	.	.	.	.	.	.	.	.	.
6580	0,5	LRTM2	NM_001163926	chr12	1799693	1816179	+	.	.	.	.	.	.	.	.	.	.	.	.	.	.	.	.	.	.	.	.
3260	1	CACNA1C	NM_199460	chr12	2032676	2677376	+	CL800053	chr12	2526981	-	.	.	.	.	.	.	.	.	.	.	.	.	.	.	.	.
7055	0,5	TSPAN9	NM_006675	chr12	3056781	3265991	+	.	.	.	.	.	.	.	.	.	.	.	.	s1452_223	chr12	3194974	+	.	.	.	.
7055	0,5	TSPAN9	NM_006675	chr12	3056781	3265991	+	.	.	.	.	.	.	.	.	.	.	.	.	s1216_186	chr12	3094669	+	.	.	.	.
937	2,5	PRMT8	NM_019854	chr12	3470624	3573399	+	.	.	.	.	.	.	.	.	.	.	.	.	.	.	.	.	.	.	.	.
44	7,5	CCND2	NM_001759	chr12	4253162	4284783	+	.	.	.	.	.	.	.	.	.	.	.	.	.	.	.	.	.	.	.	.
3773	1	FGF23	NM_020638	chr12	4347653	4359155	-	.	.	.	.	.	.	.	.	.	.	.	.	.	.	.	.	.	.	.	.
3774	1	FGF6	NM_020996	chr12	4413568	4425041	-	.	.	.	.	.	.	.	.	.	.	.	.	.	.	.	.	.	.	.	.
5051	1	RAD51AP1	NM_001130862	chr12	4518210	4539474	+	.	.	.	.	.	.	.	.	.	.	.	.	.	.	.	.	.	.	.	.
3636	1	DYRK4	NM_003845	chr12	4569504	4593302	+	.	.	.	.	.	.	.	.	.	.	.	.	.	.	.	.	.	.	.	.
2941	1	AKAP3	NM_006422	chr12	4594936	4624604	-	.	.	.	.	.	.	.	.	.	.	.	.	s1457_224	chr12	4597139	+	.	.	.	.
4532	1	NDUFA9	NM_005002	chr12	4628524	4666981	+	.	.	.	.	.	.	.	.	.	.	.	.	.	.	.	.	.	.	.	.
4198	1	KCNA1	NM_000217	chr12	4889333	4897683	+	.	.	.	.	.	.	.	.	.	.	.	.	.	.	.	.	.	.	.	.
7382	0,25	KCNA5	NM_002234	chr12	5023345	5026215	+	.	.	.	.	.	.	.	.	.	.	.	.	.	.	.	.	.	.	.	.
4637	1	NTF3	NM_001102654	chr12	5411540	5474726	+	.	.	.	.	.	.	.	.	.	.	.	.	.	.	.	.	.	.	.	.
2826	1,25	VWF	NM_000552	chr12	5928300	6104097	-	.	.	.	.	.	.	.	.	.	.	.	.	.	.	.	.	.	.	.	.
713	2,75	CD9	NM_001769	chr12	6179742	6217698	+	.	.	.	.	.	.	.	.	.	.	.	.	.	.	.	.	.	.	.	.
762	2,75	TNFRSF1A	NM_001065	chr12	6308183	6321544	-	.	.	.	.	.	.	.	.	.	.	.	.	.	.	.	.	.	.	.	.
3855	1	GAPDH	NM_001256799	chr12	6514669	6517798	+	.	.	.	.	.	.	.	.	.	.	.	.	.	.	.	.	.	.	.	.
6172	0,5	CHD4	NM_001273	chr12	6549508	6586812	-	.	.	.	.	.	.	.	.	.	.	.	.	.	.	.	.	.	.	.	.
867	2,5	ING4	NM_001127585	chr12	6629964	6642569	-	CL800842	chr12	6634884	+	.	.	.	.	.	.	.	.	.	.	.	.	.	.	.	.
705	3	ZNF384	NM_001039920	chr12	6645903	6668999	-	CL800014	chr12	6659176	+	.	.	.	.	.	.	.	.	.	.	.	.	.	.	.	.
705	3	ZNF384	NM_001039920	chr12	6645903	6668999	-	CL800372	chr12	6661651	+	.	.	.	.	.	.	.	.	.	.	.	.	.	.	.	.

Table S2

tumor associated genes								HIV				MLV				MMTV				MMTV(SIN)				MMTV(SIN)arrest			
rank	score	gene symbol	RefSEq	chrom	txStart	txEnd	strand	integrant	chr	position	strand	integrant	chr	position	strand	integrant	chr	position	strand	integrant	chr	position	strand	integrant	chr	position	strand
3300	1	CD4	NM_001195014	chr12	6768898	6800237	+	.	.	.	.	.	.	.	.	.	.	.	.	.	.	.	.	.	.	.	.
6554	0,5	LEPREL2	NM_014262	chr12	6807832	6819279	+	.	.	.	.	.	.	.	.	.	.	.	.	.	.	.	.	.	.	.	.
3922	1	GNB3	NM_002075	chr12	6819635	6826818	+	.	.	.	.	.	.	.	.	.	.	.	.	.	.	.	.	.	.	.	.
5660	1	USP5	NM_001098536	chr12	6831545	6846056	+	.	.	.	.	.	.	.	.	.	.	.	.	.	.	.	.	.	.	.	.
5531	1	TPI1	NM_001159287	chr12	6846844	6850371	+	.	.	.	.	.	.	.	.	.	.	.	.	.	.	.	.	.	.	.	.
1042	2,25	ENO2	NM_001975	chr12	6893874	6903120	+	.	.	.	.	.	.	.	.	.	.	.	.	.	.	.	.	.	.	.	.
3060	1	ATN1	NM_001940	chr12	6907740	6921745	+	.	.	.	.	.	.	.	.	.	.	.	.	.	.	.	.	.	.	.	.
422	3,5	PTPN6	NM_080548	chr12	6926000	6940740	+	.	.	.	.	.	.	.	.	.	.	.	.	.	.	.	.	.	.	.	.
4779	1	PHB2	NM_001144831	chr12	6944775	6950177	-	.	.	.	.	.	.	.	.	.	.	.	.	.	.	.	.	.	.	.	.
5884	0,75	C1S	NM_001734	chr12	7038240	7048596	+	.	.	.	.	.	.	.	.	.	.	.	.	.	.	.	.	.	.	.	.
6088	0,5	C1RL	NM_016546	chr12	7138286	7153145	-	.	.	.	.	.	.	.	.	.	.	.	.	.	.	.	.	.	.	.	.
3297	1	CD163	NM_203416	chr12	7514678	7547681	-	.	.	.	.	.	.	.	.	.	.	.	.	.	.	.	.	.	.	.	.
3004	1	APOBEC1	NM_001644	chr12	7693262	7709769	-	.	.	.	.	.	.	.	.	.	.	.	.	.	.	.	.	.	.	.	.
3873	1	GDF3	NM_020634	chr12	7733647	7739627	-	.	.	.	.	.	.	.	.	.	.	.	.	.	.	.	.	.	.	.	.
3386	1	CLEC4C	NM_130441	chr12	7773277	7793336	-	.	.	.	.	.	.	.	.	.	.	.	.	.	.	.	.	.	.	.	.
2773	1,25	SLC2A3	NM_006931	chr12	7963090	7980159	-	.	.	.	.	.	.	.	.	.	.	.	.	.	.	.	.	.	.	.	.
6659	0,5	NECAP1	NR_024260	chr12	8126073	8141640	+	.	.	.	.	.	.	.	.	.	.	.	.	.	.	.	.	2388_207_1	chr12	8140103	+
6344	0,5	FAM90A1	NM_018088	chr12	8265122	8271481	-	.	.	.	.	.	.	.	.	.	.	.	.	.	.	.	.	.	.	.	.
6189	0,5	CLEC4E	NM_014358	chr12	8577167	8584825	-	.	.	.	.	.	.	.	.	.	.	.	.	.	.	.	.	.	.	.	.
2935	1	AICDA	NM_020661	chr12	8646028	8656709	-	.	.	.	.	.	.	.	.	.	.	.	.	.	.	.	.	.	.	.	.
7420	0,25	MFAP5	NM_003480	chr12	8689806	8706700	-	.	.	.	.	.	.	.	.	.	.	.	.	.	.	.	.	.	.	.	.
983	2,25	A2M	NM_000014	chr12	9111570	9159825	-	.	.	.	.	.	.	.	.	.	.	.	.	.	.	.	.	.	.	.	.
5015	1	PZP	NM_002864	chr12	9192702	9252233	-	.	.	.	.	.	.	.	.	.	.	.	.	.	.	.	.	.	.	.	.
6154	0,5	CD69	NM_001781	chr12	9796348	9804764	-	.	.	.	.	.	.	.	.	.	.	.	.	.	.	.	.	.	.	.	.
3384	1	CLEC2B	NM_005127	chr12	9896234	9913725	-	.	.	.	.	.	.	.	.	.	.	.	.	.	.	.	.	.	.	.	.
6700	0,5	OLR1	NM_001172632	chr12	10202165	10216057	-	.	.	.	.	.	.	.	.	.	.	.	.	.	.	.	.	.	.	.	.
6370	0,5	GABARAPL1	NM_031412	chr12	10256755	10266991	+	.	.	.	.	.	.	.	.	.	.	.	.	.	.	.	.	.	.	.	.
1929	1,75	KLRK1	NM_007360	chr12	10416218	10433920	-	.	.	.	.	.	.	.	.	.	.	.	.	.	.	.	.	.	.	.	.
6541	0,5	KLRC4	NM_013431	chr12	10451249	10453623	-	.	.	.	.	.	.	.	.	.	.	.	.	.	.	.	.	.	.	.	.
2637	1,25	KLRC3	NM_007333	chr12	10459449	10464461	-	.	.	.	.	.	.	.	.	.	.	.	.	.	.	.	.	.	.	.	.
7390	0,25	KLRC2	NM_002260	chr12	10474470	10479859	-	.	.	.	.	.	.	.	.	.	.	.	.	.	.	.	.	.	.	.	.
2636	1,25	KLRC1	NM_213657	chr12	10489903	10498482	-	.	.	.	.	.	.	.	.	.	.	.	.	.	.	.	.	.	.	.	.
961	2,5	STYK1	NM_018423	chr12	10662804	10718158	-	.	.	.	.	.	.	.	.	.	.	.	.	.	.	.	.	.	.	.	.
7008	0,5	TAS2R50	NM_176890	chr12	11029778	11030778	-	.	.	.	.	.	.	.	.	.	.	.	.	.	.	.	.	.	.	.	.
6764	0,5	PRB2	NM_006248	chr12	11435740	11439765	-	.	.	.	.	.	.	.	.	.	.	.	.	.	.	.	.	.	.	.	.
569	3	ETV6	NM_001987	chr12	11694054	11939592	+	.	.	.	.	.	.	.	.	.	.	.	.	.	.	.	.	.	.	.	.
885	2,5	LRP6	NM_002336	chr12	12160227	12311078	-	CL529100	chr12	12183512	+	.	.	.	.	.	.	.	.	.	.	.	.	.	.	.	.
3621	1	DUSP16	NM_030640	chr12	12517482	12606715	-	.	.	.	.	.	.	.	.	.	.	.	.	.	.	.	.	.	.	.	.
101	5,5	CDKN1B	NM_004064	chr12	12761568	12766572	+	.	.	.	.	.	.	.	.	.	.	.	.	.	.	.	.	.	.	.	.
3549	1	DDX47	NM_016355	chr12	12857546	12874182	+	.	.	.	.	.	.	.	.	.	.	.	.	.	.	.	.	.	.	.	.
7334	0,25	GPRC5A	NM_003979	chr12	12935222	12957867	+	.	.	.	.	.	.	.	.	.	.	.	.	.	.	.	.	.	.	.	.
6526	0,5	KIAA1467	NM_020853	chr12	13088581	13127650	+	.	.	.	.	.	.	.	.	.	.	.	.	.	.	.	.	.	.	.	.
5905	0,75	EMP1	NM_001423	chr12	13240868	13260975	+	.	.	.	.	.	.	.	.	.	.	.	.	.	.	.	.	.	.	.	.
850	2,5	GRIN2B	NM_000834	chr12	13605676	14024289	-	.	.	.	.	.	.	.	.	.	.	.	.	.	.	.	.	.	.	.	.
6749	0,5	PLBD1	NM_024829	chr12	14547863	14612058	-	.	.	.	.	.	.	.	.	.	.	.	.	.	.	.	.	.	.	.	.
1439	2	GUCY2C	NM_004963	chr12	14656834	14740786	-	.	.	.	.	.	.	.	.	.	.	.	.	.	.	.	.	.	.	.	.
6613	0,5	MGP	NM_000900	chr12	14925381	14930120	-	.	.	.	.	.	.	.	.	.	.	.	.	.	.	.	.	.	.	.	.
3027	1	ARHGDIB	NM_001175	chr12	14986216	15005829	-	.	.	.	.	.	.	.	.	.	.	.	.	.	.	.	.	.	.	.	.
2312	1,5	PDE6H	NM_006205	chr12	15017222	15026066	+	.	.	.	.	.	.	.	.	.	.	.	.	.	.	.	.	.	.	.	.
5095	1	RERG	NM_032918	chr12	15151982	15265678	-	.	.	.	.	.	.	.	.	.	.	.	.	.	.	.	.	.	.	.	.
2735	1,25	PTPRO	NM_030667	chr12	15366457	15642532	+	.	.	.	.	.	.	.	.	.	.	.	.	.	.	.	.	.	.	.	.

Table S2

tumor associated genes								HIV				MLV				MMTV				MMTV(SIN)				MMTV(SIN)arrest			
rank	score	gene symbol	RefSEq	chrom	txStart	txEnd	strand	integrant	chr	position	strand	integrant	chr	position	strand	integrant	chr	position	strand	integrant	chr	position	strand	integrant	chr	position	strand
6319	0,5	EPS8	NM_004447	chr12	15664341	15833777	-	.	.	.	.	.	.	.	.	.	.	.	.	.	.	.	.	.	.	.	.
2792	1,25	STRAP	NM_007178	chr12	15926554	15947677	+	.	.	.	.	.	.	.	.	.	.	.	.	.	.	.	.	.	.	.	.
3559	1	DERA	NM_015954	chr12	15955452	16081582	+	.	.	.	.	.	.	.	.	.	.	.	.	.	.	.	.	.	.	.	.
2675	1,25	MGST1	NM_145791	chr12	16391978	16408611	+	.	.	.	.	.	.	.	.	.	.	.	.	.	.	.	.	.	.	.	.
4293	1	LMO3	NM_001243612	chr12	16592572	16650171	-	.	.	.	.	.	.	.	.	.	.	.	.	.	.	.	.	.	.	.	.
6807	0,5	RERGL	NM_024730	chr12	18125069	18134381	-	.	.	.	.	.	.	.	.	.	.	.	.	.	.	.	.	.	.	.	.
1624	2	PIK3C2G	NM_004570	chr12	18305740	18692619	+	.	.	.	.	.	.	.	.	.	.	.	.	.	.	.	.	365_99_1	chr12	18385910	+
1635	2	PLCZ1	NM_033123	chr12	18727376	18782260	-	.	.	.	.	.	.	.	.	.	.	.	.	.	.	.	.	.	.	.	.
5288	1	SLCO1C1	NM_001145946	chr12	20739555	20797587	+	.	.	.	.	.	.	.	.	.	.	.	.	.	.	.	.	.	.	.	.
5287	1	SLCO1A2	NM_134431	chr12	21308800	21439638	-	.	.	.	.	.	.	.	.	.	.	.	.	.	.	.	.	.	.	.	.
7364	0,25	IAPP	NM_000415	chr12	21417068	21424181	+	.	.	.	.	.	.	.	.	.	.	.	.	.	.	.	.	.	.	.	.
5090	1	RECQL	NM_002907	chr12	21513110	21545870	-	.	.	.	.	.	.	.	.	.	.	.	.	.	.	.	.	.	.	.	.
479	3,25	LDHB	NM_002300	chr12	21679541	21702056	-	.	.	.	.	.	.	.	.	.	.	.	.	.	.	.	.	.	.	.	.
6192	0,5	CMAS	NM_018686	chr12	22090376	22109873	+	.	.	.	.	.	.	.	.	.	.	.	.	.	.	.	.	.	.	.	.
5367	1	ST8SIA1	NM_003034	chr12	22237591	22378915	-	.	.	.	.	.	.	.	.	.	.	.	.	105_1_735_25	chr12	22329345	-	.	.	.	.
1391	2	ETNK1	NM_018638	chr12	22669342	22734875	+	.	.	.	.	.	.	.	.	.	.	.	.	.	.	.	.	.	.	.	.
7566	0,25	SOX5	NM_006940	chr12	23576497	23993904	-	CL800595	chr12	24533325	-	.	.	.	.	.	.	.	.	.	.	.	.	.	.	.	.
1876	1,75	BCAT1	NM_001178092	chr12	24854224	24993660	-	.	.	.	.	.	.	.	.	.	.	.	.	.	.	.	.	.	.	.	.
7403	0,25	LRMP	NM_006152	chr12	25096447	25152536	+	.	.	.	.	.	.	.	.	.	.	.	.	.	.	.	.	.	.	.	.
7225	0,25	CASC1	NM_001204102	chr12	25152489	25239361	-	.	.	.	.	.	.	.	.	.	.	.	.	.	.	.	.	.	.	.	.
16	9,75	KRAS	NM_004985	chr12	25249446	25295121	-	CL529632	chr12	25282092	+	.	.	.	.	.	.	.	.	.	.	.	.	.	.	.	.
7505	0,25	RASSF8	NM_007211	chr12	26003230	26124092	+	.	.	.	.	.	.	.	.	.	.	.	.	.	.	.	.	.	.	.	.
606	3	ITPR2	NM_002223	chr12	26379551	26877398	-	CL529546	chr12	26471577	-	.	.	.	.	.	.	.	.	.	.	.	.	.	.	.	.
5387	1	STK38L	NM_015000	chr12	27288344	27370157	+	.	.	.	.	.	.	.	.	.	.	.	.	.	.	.	.	.	.	.	.
1148	2,25	PTHLH	NM_198965	chr12	28002283	28016183	-	.	.	.	.	.	.	.	.	.	.	.	.	.	.	.	.	.	.	.	.
4159	1	IPO8	NM_001190995	chr12	30673181	30721445	-	.	.	.	.	.	.	.	.	.	.	.	.	.	.	.	.	.	.	.	.
3599	1	DNM1L	NM_012062	chr12	32723403	32789851	+	CL529506	chr12	32746257	+	.	.	.	.	.	.	.	.	.	.	.	.	.	.	.	.
5721	1	YARS2	NM_001040436	chr12	32790744	32800154	-	.	.	.	.	.	.	.	.	.	.	.	.	.	.	.	.	.	.	.	.
2955	1	ALG10	NM_032834	chr12	34066482	34072503	+	.	.	.	.	.	.	.	.	.	.	.	.	.	.	.	.	.	.	.	.
3449	1	CPNE8	NM_153634	chr12	37332268	37585687	-	CL800079	chr12	37558080	-	.	.	.	.	.	.	.	.	.	.	.	.	.	.	.	.
3449	1	CPNE8	NM_153634	chr12	37332268	37585687	-	CL800741	chr12	37374076	+	.	.	.	.	.	.	.	.	.	.	.	.	.	.	.	.
3449	1	CPNE8	NM_153634	chr12	37332268	37585687	-	CL800737	chr12	37334767	+	.	.	.	.	.	.	.	.	.	.	.	.	.	.	.	.
770	2,5	ABCD2	NM_005164	chr12	38231288	38300110	-	.	.	.	.	.	.	.	.	.	.	.	.	.	.	.	.	.	.	.	.
7555	0,25	SLC2A13	NM_052885	chr12	38435089	38785928	-	.	.	.	.	.	.	.	.	.	.	.	.	.	.	.	.	.	.	.	.
1511	2	LRRK2	NM_198578	chr12	38905079	39049353	+	.	.	.	.	.	.	.	.	.	.	.	.	.	.	.	.	.	.	.	.
717	2,75	CNTN1	NM_001256063	chr12	39372510	39652786	+	.	.	.	.	.	.	.	.	_183_1_157	chr12	39449228	+	.	.	.	.	.	.	.	.
4748	1	PDZRN4	NM_001164595	chr12	39868516	40254659	+	.	.	.	.	.	.	.	.	.	.	.	.	.	.	.	.	133_82_1	chr12	40005465	+
7491	0,25	PRICKLE1	NM_153026	chr12	41138406	41269839	-	.	.	.	.	.	.	.	.	.	.	.	.	s980_160	chr12	41143119	+	.	.	.	.
521	3	ADAMTS20	NM_025003	chr12	42034278	42231991	-	.	.	.	.	.	.	.	.	.	.	.	.	.	.	.	.	.	.	.	.
2628	1,25	IRAK4	NM_016123	chr12	42439013	42469613	+	.	.	.	.	.	.	.	.	.	.	.	.	.	.	.	.	.	.	.	.
2816	1,25	TWF1	NM_002822	chr12	42473792	42486445	-	.	.	.	.	.	.	.	.	.	.	.	.	.	.	.	.	.	.	.	.
2277	1,5	NELL2	NM_001145108	chr12	43188324	43556514	-	CL529728	chr12	43216535	-	.	.	.	.	.	.	.	.	.	.	.	.	.	.	.	.
2277	1,5	NELL2	NM_001145108	chr12	43188324	43556514	-	CL529729	chr12	43475807	-	.	.	.	.	.	.	.	.	.	.	.	.	.	.	.	.
7164	0,25	ANO6	NM_001142679	chr12	43896036	44120454	+	.	.	.	.	.	.	.	.	.	.	.	.	.	.	.	.	.	.	.	.
1965	1,75	SLC38A1	NM_001077484	chr12	44863107	44949475	-	.	.	.	.	.	.	.	.	.	.	.	.	.	.	.	.	.	.	.	.
6007	0,5	AMIGO2	NM_181847	chr12	45755756	45760001	-	.	.	.	.	.	.	.	.	.	.	.	.	.	.	.	.	.	.	.	.
585	3	HDAC7	NM_001098416	chr12	46462773	46500030	-	.	.	.	.	.	.	.	.	.	.	.	.	.	.	.	.	1213_134_1	chr12	46466915	+
350	3,75	VDR	NM_001017536	chr12	46521586	46585081	-	CL529530	chr12	46573627	+	.	.	.	.	.	.	.	.	.	.	.	.	.	.	.	.
6201	0,5	COL2A1	NM_001844	chr12	46653014	46684552	-	.	.	.	.	.	.	.	.	.	.	.	.	.	.	.	.	.	.	.	.
1744	2	SENP1	NM_001267594	chr12	46722947	46786358	-	.	.	.	.	.	.	.	.	.	.	.	.	.	.	.	.	.	.	.	.
1609	2	PFKM	NM_001166688	chr12	46802696	46826454	+	.	.	.	.	.	.	.	.	.	.	.	.	.	.	.	.	.	.	.	.

Table S2

tumor associated genes								HIV				MLV				MMTV				MMTV(SIN)				MMTV(SIN)arrest			
rank	score	gene symbol	RefSEq	chrom	txStart	txEnd	strand	integrant	chr	position	strand	integrant	chr	position	strand	integrant	chr	position	strand	integrant	chr	position	strand	integrant	chr	position	strand
7173	0,25	ASB8	NM_024095	chr12	46827838	46837644	-	.	.	.	.	.	.	.	.	.	.	.	.	.	.	.	.	.	.	.	.
6420	0,5	H1FNT	NM_181788	chr12	47009029	47010329	+	.	.	.	.	.	.	.	.	.	.	.	.	.	.	.	.	.	.	.	.
4253	1	LALBA	NM_002289	chr12	47247733	47250096	-	.	.	.	.	.	.	.	.	.	.	.	.	.	.	.	.	.	.	.	.
3291	1	CCNT1	NM_001240	chr12	47368507	47397048	-	CL529596	chr12	47389026	+	.	.	.	.	.	.	.	.	.	.	.	.	.	.	.	.
1220	2	ADCY6	NM_020983	chr12	47446247	47469087	-	CL800509	chr12	47447878	+	.	.	.	.	.	.	.	.	.	.	.	.	.	.	.	.
560	3	DDX23	NM_004818	chr12	47509805	47532224	-	.	.	.	.	.	.	.	.	.	.	.	.	.	.	.	.	.	.	.	.
2380	1,5	RND1	NM_014470	chr12	47537182	47545920	-	.	.	.	.	.	.	.	.	.	.	.	.	.	.	.	.	.	.	.	.
3016	1	ARF3	NM_001659	chr12	47616258	47637519	-	CL800512	chr12	47634478	+	.	.	.	.	.	.	.	.	.	.	.	.	.	.	.	.
980	2,5	WNT10B	NM_003394	chr12	47645389	47651908	-	.	.	.	.	.	.	.	.	.	.	.	.	.	.	.	.	.	.	.	.
2829	1,25	WNT1	NM_005430	chr12	47658502	47662663	+	.	.	.	.	.	.	.	.	.	.	.	.	.	.	.	.	.	.	.	.
6259	0,5	DDN	NM_015086	chr12	47675199	47679355	-	.	.	.	.	.	.	.	.	.	.	.	.	.	.	.	.	.	.	.	.
4940	1	PRKAG1	NM_001206709	chr12	47682321	47698896	-	.	.	.	.	.	.	.	.	.	.	.	.	.	.	.	.	.	.	.	.
4410	1	MLL2	NM_003482	chr12	47699024	47735374	-	.	.	.	.	.	.	.	.	.	.	.	.	.	.	.	.	.	.	.	.
7065	0,5	TUBA1B	NM_006082	chr12	47807832	47811571	-	.	.	.	.	.	.	.	.	.	.	.	.	.	.	.	.	.	.	.	.
7066	0,5	TUBA1C	NM_032704	chr12	47945131	47953380	+	.	.	.	.	.	.	.	.	.	.	.	.	.	.	.	.	.	.	.	.
5563	1	TROAP	NM_001100620	chr12	48003237	48004438	+	.	.	.	.	.	.	.	.	.	.	.	.	.	.	.	.	.	.	.	.
6495	0,5	KCNH3	NM_012284	chr12	48219206	48238344	+	.	.	.	.	.	.	.	.	.	.	.	.	.	.	.	.	.	.	.	.
3941	1	GPD1	NM_005276	chr12	48783868	48791370	+	.	.	.	.	.	.	.	.	.	.	.	.	.	.	.	.	.	.	.	.
1262	2	ATF1	NM_005171	chr12	49444055	49501210	+	.	.	.	.	AY516564	chr12	49465732	-	.	.	.	.	.	.	.	.	.	.	.	.
3848	1	GALNT6	NM_007210	chr12	50032099	50071467	-	.	.	.	.	.	.	.	.	.	.	.	.	.	.	.	.	.	.	.	.
6859	0,5	SCN8A	NM_014191	chr12	50271286	50492915	+	.	.	.	.	.	.	.	.	.	.	.	.	.	.	.	.	.	.	.	.
5989	0,5	ACVRL1	NM_000020	chr12	50587468	50603412	+	.	.	.	.	.	.	.	.	.	.	.	.	.	.	.	.	.	.	.	.
773	2,5	ACVR1B	NM_004302	chr12	50631717	50677130	+	CL529104	chr12	50636672	+	.	.	.	.	.	.	.	.	.	.	.	.	.	.	.	.
6408	0,5	GRASP	NM_181711	chr12	50687014	50695938	+	.	.	.	.	.	.	.	.	.	.	.	.	.	.	.	.	.	.	.	.
2699	1,25	NR4A1	NM_173157	chr12	50731452	50739558	+	.	.	.	.	.	.	.	.	.	.	.	.	.	.	.	.	.	.	.	.
2641	1,25	KRT7	NM_005556	chr12	50913220	50928976	+	.	.	.	.	.	.	.	.	.	.	.	.	.	.	.	.	.	.	.	.
4250	1	KRT73	NM_175068	chr12	51287620	51298610	-	.	.	.	.	.	.	.	.	.	.	.	.	.	.	.	.	.	.	.	.
4248	1	KRT1	NM_006121	chr12	51354786	51360458	-	.	.	.	.	.	.	.	.	.	.	.	.	.	.	.	.	.	.	.	.
4249	1	KRT4	NM_002272	chr12	51486593	51494167	-	.	.	.	.	.	.	.	.	.	.	.	.	.	.	.	.	.	.	.	.
2642	1,25	KRT8	NM_001256282	chr12	51577237	51606520	-	.	.	.	.	.	.	.	.	.	.	.	.	.	.	.	.	.	.	.	.
6542	0,5	KRT18	NM_000224	chr12	51629109	51632952	+	.	.	.	.	.	.	.	.	.	.	.	.	.	.	.	.	.	.	.	.
3669	1	EIF4B	NM_001417	chr12	51686328	51722260	+	.	.	.	.	AY516229	chr12	51687588	-	.	.	.	.	.	.	.	.	.	.	.	.
5455	1	TENC1	NM_170754	chr12	51730101	51744429	+	.	.	.	.	.	.	.	.	.	.	.	.	.	.	.	.	.	.	.	.
6967	0,5	SPRYD3	NM_032840	chr12	51744366	51759471	-	.	.	.	.	.	.	.	.	.	.	.	.	.	.	.	.	.	.	.	.
1077	2,25	IGFBP6	NM_002178	chr12	51777702	51782395	+	.	.	.	.	.	.	.	.	.	.	.	.	.	.	.	.	.	.	.	.
5313	1	SOAT2	NM_003578	chr12	51783540	51804590	+	.	.	.	.	.	.	.	.	.	.	.	.	.	.	.	.	.	.	.	.
3465	1	CSAD	NM_001244706	chr12	51837713	51844544	-	.	.	.	.	.	.	.	.	.	.	.	.	.	.	.	.	.	.	.	.
4178	1	ITGB7	NM_000889	chr12	51871373	51887267	-	.	.	.	.	.	.	.	.	.	.	.	.	.	.	.	.	.	.	.	.
5063	1	RARG	NM_001042728	chr12	51890616	51900464	-	.	.	.	.	.	.	.	.	.	.	.	.	.	.	.	.	.	.	.	.
4756	1	PFDN5	NM_002624	chr12	51975501	51979501	+	.	.	.	.	.	.	.	.	.	.	.	.	.	.	.	.	.	.	.	.
5977	0,5	AAAS	NM_001173466	chr12	51987506	52001679	-	.	.	.	.	.	.	.	.	.	.	.	.	.	.	.	.	.	.	.	.
232	4,25	SP1	NM_001251825	chr12	52060245	52096493	+	CL800629	chr12	52069940	+	.	.	.	.	.	.	.	.	.	.	.	.	3213_316_1	chr12	52064552	+
6006	0,5	AMHR2	NM_001164691	chr12	52103905	52111585	+	.	.	.	.	.	.	.	.	.	.	.	.	.	.	.	.	.	.	.	.
4345	1	MAP3K12	NM_001193511	chr12	52160542	52179711	-	.	.	.	.	.	.	.	.	.	.	.	.	.	.	.	.	.	.	.	.
3076	1	ATP5G2	NM_005176	chr12	52345210	52356376	-	.	.	.	.	.	.	.	.	.	.	.	.	.	.	.	.	.	.	.	.
3269	1	CALCOCO1	NM_001143682	chr12	52391169	52407574	-	.	.	.	.	.	.	.	.	.	.	.	.	.	.	.	.	.	.	.	.
588	3	HOXC13	NM_017410	chr12	52618842	52626595	+	.	.	.	.	.	.	.	.	.	.	.	.	.	.	.	.	.	.	.	.
4072	1	HOXC11	NM_014212	chr12	52653176	52656470	+	.	.	.	.	AY516760	chr12	52655353	+	.	.	.	.	.	.	.	.	.	.	.	.
6448	0,5	HOXC10	NM_017409	chr12	52665212	52670329	+	.	.	.	.	.	.	.	.	.	.	.	.	.	.	.	.	.	.	.	.
4073	1	HOXC6	NM_004503	chr12	52708460	52710874	+	.	.	.	.	.	.	.	.	.	.	.	.	.	.	.	.	.	.	.	.
5300	1	SMUG1	NM_001243791	chr12	52861505	52869045	-	.	.	.	.	.	.	.	.	.	.	.	.	.	.	.	.	.	.	.	.

Table S2

tumor associated genes								HIV				MLV				MMTV				MMTV(SIN)				MMTV(SIN)arrest			
rank	score	gene symbol	RefSEq	chrom	txStart	txEnd	strand	integrant	chr	position	strand	integrant	chr	position	strand	integrant	chr	position	strand	integrant	chr	position	strand	integrant	chr	position	strand
3285	1	CBX5	NM_001127322	chr12	52910996	52939596	-	.	.	.	.	.	.	.	.	.	.	.	.	.	.	.	.	.	.	.	.
7353	0,25	HNRNPA1	NM_002136	chr12	52960754	52965297	+	.	.	.	.	.	.	.	.	.	.	.	.	.	.	.	.	.	.	.	.
868	2,5	ITGA5	NM_002205	chr12	53075311	53099317	-	.	.	.	.	.	.	.	.	.	.	.	.	.	.	.	.	.	.	.	.
6725	0,5	PDE1B	NM_000924	chr12	53229443	53259290	+	.	.	.	.	.	.	.	.	.	.	.	.	.	.	.	.	.	.	.	.
1668	2	PPP1R1A	NM_006741	chr12	53259290	53268710	-	.	.	.	.	.	.	.	.	.	.	.	.	.	.	.	.	.	.	.	.
1110	2,25	METTL7B	NM_152637	chr12	54361596	54364661	+	.	.	.	.	.	.	.	.	.	.	.	.	.	.	.	.	.	.	.	.
4174	1	ITGA7	NM_001144996	chr12	54364620	54387953	-	.	.	.	.	.	.	.	.	.	.	.	.	.	.	.	.	.	.	.	.
5087	1	RDH5	NM_001199771	chr12	54400417	54404793	+	.	.	.	.	.	.	.	.	.	.	.	.	.	.	.	.	.	.	.	.
6153	0,5	CD63	NM_001780	chr12	54405493	54409189	-	.	.	.	.	.	.	.	.	.	.	.	.	.	.	.	.	.	.	.	.
6379	0,5	GDF11	NM_005811	chr12	54423330	54432932	+	.	.	.	.	.	.	.	.	.	.	.	.	.	.	.	.	.	.	.	.
2260	1,5	MMP19	NM_002429	chr12	54515480	54523002	-	.	.	.	.	.	.	.	.	.	.	.	.	.	.	.	.	.	.	.	.
1034	2,25	DGKA	NM_001345	chr12	54611212	54634074	+	.	.	.	.	.	.	.	.	.	.	.	.	.	.	.	.	.	.	.	.
131	5	CDK2	NM_001798	chr12	54646822	54652835	+	.	.	.	.	.	.	.	.	.	.	.	.	.	.	.	.	.	.	.	.
5038	1	RAB5B	NM_001252036	chr12	54653963	54676734	+	.	.	.	.	.	.	.	.	.	.	.	.	.	.	.	.	.	.	.	.
5411	1	SUOX	NM_001032386	chr12	54677309	54685576	+	.	.	.	.	.	.	.	.	.	.	.	.	.	.	.	.	.	.	.	.
42	7,75	ERBB3	NM_001005915	chr12	54760158	54765668	+	.	.	.	.	.	.	.	.	.	.	.	.	.	.	.	.	.	.	.	.
5294	1	SMARCC2	NM_139067	chr12	54841902	54869618	-	.	.	.	.	.	.	.	.	.	.	.	.	.	.	.	.	.	.	.	.
3464	1	CS	NM_004077	chr12	54951749	54980442	-	.	.	.	.	.	.	.	.	.	.	.	.	.	.	.	.	.	.	.	.
7575	0,25	STAT2	NM_198332	chr12	55021648	55040304	-	CL799747	chr12	55032449	-	.	.	.	.	.	.	.	.	.	.	.	.	.	.	.	.
2438	1,5	TIMELESS	NM_003920	chr12	55096423	55129467	-	.	.	.	.	.	.	.	.	.	.	.	.	.	.	.	.	.	.	.	.
2158	1,5	GLS2	NM_013267	chr12	55151002	55168448	-	.	.	.	.	.	.	.	.	.	.	.	.	.	.	.	.	.	.	.	.
3072	1	ATP5B	NM_001686	chr12	55318225	55326119	-	.	.	.	.	.	.	.	.	.	.	.	.	.	.	.	.	.	.	.	.
1554	2	NACA	NM_001113203	chr12	55392477	55405593	-	.	.	.	.	.	.	.	.	.	.	.	.	.	.	.	.	.	.	.	.
4938	1	PRIM1	NM_000946	chr12	55411630	55432413	-	.	.	.	.	.	.	.	.	.	.	.	.	.	.	.	.	.	.	.	.
4090	1	HSD17B6	NM_003725	chr12	55443374	55467841	+	.	.	.	.	.	.	.	.	.	.	.	.	.	.	.	.	.	.	.	.
7005	0,5	TAC3	NM_013251	chr12	55690047	55696611	-	.	.	.	.	.	.	.	.	.	.	.	.	.	.	.	.	.	.	.	.
960	2,5	STAT6	NM_001178081	chr12	55775454	55791463	-	.	.	.	.	.	.	.	.	.	.	.	.	.	.	.	.	.	.	.	.
2649	1,25	LRP1	NM_002332	chr12	55808548	55893392	+	.	.	.	.	.	.	.	.	.	.	.	.	.	.	.	.	.	.	.	.
5235	1	SHMT2	NM_001166359	chr12	55910377	55914985	+	.	.	.	.	.	.	.	.	.	.	.	.	.	.	.	.	.	.	.	.
4527	1	NDUFA4L2	NM_020142	chr12	55914952	55920742	-	.	.	.	.	.	.	.	.	.	.	.	.	.	.	.	.	.	.	.	.
5368	1	STAC3	NM_145064	chr12	55923508	55931236	-	.	.	.	.	.	.	.	.	.	.	.	.	.	.	.	.	.	.	.	.
7374	0,25	INHBC	NM_005538	chr12	56114809	56130876	+	.	.	.	.	.	.	.	.	.	.	.	.	.	.	.	.	.	.	.	.
2205	1,5	INHBE	NM_031479	chr12	56135362	56138058	+	.	.	.	.	.	.	.	.	.	.	.	.	.	.	.	.	.	.	.	.
375	3,5	GLI1	NM_001160045	chr12	56140184	56152314	+	.	.	.	.	.	.	.	.	.	.	.	.	.	.	.	.	.	.	.	.
7171	0,25	ARHGAP9	NM_001080156	chr12	56152304	56157995	-	.	.	.	.	.	.	.	.	.	.	.	.	.	.	.	.	.	.	.	.
1104	2,25	MARS	NM_004990	chr12	56168002	56196705	+	.	.	.	.	.	.	.	.	.	.	.	.	.	.	.	.	.	.	.	.
321	3,75	DDIT3	NM_001195057	chr12	56196637	56200567	-	.	.	.	.	.	.	.	.	.	.	.	.	.	.	.	.	.	.	.	.
4365	1	MBD6	NM_052897	chr12	56202925	56210198	+	.	.	.	.	.	.	.	.	.	.	.	.	.	.	.	.	.	.	.	.
7269	0,25	DCTN2	NM_006400	chr12	56210099	56227381	-	.	.	.	.	.	.	.	.	.	.	.	.	.	.	.	.	.	.	.	.
5932	0,75	KIF5A	NM_004984	chr12	56230113	56264821	+	.	.	.	.	.	.	.	.	.	.	.	.	.	.	.	.	2562_221_1	chr12	56262714	-
414	3,5	PIP4K2C	NM_001146259	chr12	56271208	56283478	+	.	.	.	.	.	.	.	.	.	.	.	.	.	.	.	.	.	.	.	.
6291	0,5	DTX3	NM_178502	chr12	56284870	56289850	+	.	.	.	.	.	.	.	.	.	.	.	.	.	.	.	.	.	.	.	.
7553	0,25	SLC26A10	NM_133489	chr12	56299959	56306201	+	.	.	.	.	.	.	.	.	.	.	.	.	.	.	.	.	.	.	.	.
2020	1,5	B4GALNT1	NM_001478	chr12	56305817	56313252	-	.	.	.	.	.	.	.	.	.	.	.	.	.	.	.	.	.	.	.	.
7458	0,25	OS9	NM_001261422	chr12	56374004	56401607	+	CL529665	chr12	56393728	+	.	.	.	.	.	.	.	.	.	.	.	.	.	.	.	.
167	4,5	AGAP2	NM_001122772	chr12	56404342	56418296	-	.	.	.	.	.	.	.	.	.	.	.	.	.	.	.	.	.	.	.	.
1178	2,25	TSPAN31	NM_005981	chr12	56425050	56428293	+	.	.	.	.	.	.	.	.	.	.	.	.	.	.	.	.	.	.	.	.
9	11,5	CDK4	NM_000075	chr12	56427776	56432497	-	.	.	.	.	.	.	.	.	.	.	.	.	.	.	.	.	.	.	.	.
7414	0,25	MARCH9	NM_138396	chr12	56435147	56440460	+	.	.	.	.	.	.	.	.	.	.	.	.	.	.	.	.	.	.	.	.
1337	2	CYP27B1	NM_000785	chr12	56442383	56447243	-	.	.	.	.	.	.	.	.	.	.	.	.	.	.	.	.	.	.	.	.
7417	0,25	METTL1	NM_005371	chr12	56448617	56452181	-	.	.	.	.	.	.	.	.	.	.	.	.	.	.	.	.	.	.	.	.

Table S2

tumor associated genes								HIV				MLV				MMTV				MMTV(SIN)				MMTV(SIN)arrest			
rank	score	gene symbol	RefSEq	chrom	txStart	txEnd	strand	integrant	chr	position	strand	integrant	chr	position	strand	integrant	chr	position	strand	integrant	chr	position	strand	integrant	chr	position	strand
2810	1,25	TSFM	NM_001172695	chr12	56462794	56477637	+	.	.	.	.	.	.	.	.	.	.	.	.	.	.	.	.	.	.	.	.
6045	0,5	AVIL	NM_006576	chr12	56477426	56496119	-	.	.	.	.	.	.	.	.	.	.	.	.	.	.	.	.	.	.	.	.
6227	0,5	CTDSP2	NM_005730	chr12	56499976	56527014	-	.	.	.	.	.	.	.	.	.	.	.	.	.	.	.	.	.	.	.	.
7423	0,25	MIR26A2	NR_029847	chr12	56504658	56504742	-	.	.	.	.	.	.	.	.	.	.	.	.	.	.	.	.	.	.	.	.
2834	1,25	XRCC6BP1	NM_033276	chr12	56621711	56637319	+	.	.	.	.	.	.	.	.	.	.	.	.	.	.	.	.	.	.	.	.
6903	0,5	SLC16A7	NM_001270623	chr12	58276087	58469902	+	CL800647	chr12	58462530	-	.	.	.	.	.	.	.	.	.	.	.	.	.	.	.	.
6903	0,5	SLC16A7	NM_001270623	chr12	58276087	58469902	+	CL800646	chr12	58284462	-	.	.	.	.	.	.	.	.	.	.	.	.	.	.	.	.
2583	1,25	FAM19A2	NM_178539	chr12	60388295	60872887	-	CL800204	chr12	60511936	-	.	.	.	.	.	.	.	.	.	.	.	.	.	.	.	.
7613	0,25	USP15	NM_001252079	chr12	60940387	61016741	+	.	.	.	.	.	.	.	.	.	.	.	.	.	.	.	.	.	.	.	.
7432	0,25	MON2	NM_015026	chr12	61146863	61277630	+	CL799644	chr12	61272669	-	.	.	.	.	.	.	.	.	.	.	.	.	.	.	.	.
7201	0,25	C12orf61	NM_175895	chr12	61281797	61283481	-	.	.	.	.	.	.	.	.	.	.	.	.	.	.	.	.	.	.	.	.
7429	0,25	MIRLET7I	NR_029661	chr12	61283732	61283816	+	.	.	.	.	.	.	.	.	.	.	.	.	.	.	.	.	.	.	.	.
2333	1,5	PPM1H	NM_020700	chr12	61324030	61614932	-	.	.	.	.	AY516231	chr12	61383003	+	.	.	.	.	.	.	.	.	.	.	.	.
7179	0,25	AVPR1A	NM_000706	chr12	61822805	61832857	-	.	.	.	.	.	.	.	.	.	.	.	.	.	.	.	.	.	.	.	.
7290	0,25	DPY19L2	NM_173812	chr12	62238959	62348621	-	CL529107	chr12	62304758	-	.	.	.	.	.	.	.	.	.	.	.	.	.	.	.	.
6970	0,5	SRGAP1	NM_020762	chr12	62524807	62827880	+	.	.	.	.	.	.	.	.	.	.	.	.	.	.	.	.	.	.	.	.
7203	0,25	C12orf66	NM_152440	chr12	62872685	62902343	-	.	.	.	.	.	.	.	.	.	.	.	.	.	.	.	.	.	.	.	.
7621	0,25	XPOT	NM_007235	chr12	63084419	63128730	+	.	.	.	.	.	.	.	.	.	.	.	.	.	.	.	.	.	.	.	.
963	2,5	TBK1	NM_013254	chr12	63132106	63182166	+	CL800829	chr12	63180761	-	.	.	.	.	.	.	.	.	.	.	.	.	.	.	.	.
7503	0,25	RASSF3	NR_040718	chr12	63290559	63377614	+	.	.	.	.	.	.	.	.	.	.	.	.	.	.	.	.	.	.	.	.
2605	1,25	GNS	NM_002076	chr12	63393488	63439493	-	.	.	.	.	.	.	.	.	.	.	.	.	.	.	.	.	.	.	.	.
1855	2	WIF1	NM_007191	chr12	63730670	63801613	-	.	.	.	.	.	.	.	.	.	.	.	.	.	.	.	.	.	.	.	.
4275	1	LEMD3	NM_001167614	chr12	63849617	63928408	+	.	.	.	.	.	.	.	.	.	.	.	.	.	.	.	.	.	.	.	.
7437	0,25	MSRB3	NM_198080	chr12	63958689	64146954	+	.	.	.	.	.	.	.	.	.	.	.	.	.	.	.	.	.	.	.	.
379	3,5	HMGA2	NM_003483	chr12	64504506	64646338	+	.	.	.	.	.	.	.	.	.	.	.	.	.	.	.	.	.	.	.	.
7399	0,25	LLPH	NM_032338	chr12	64803115	64810800	-	.	.	.	.	.	.	.	.	.	.	.	.	.	.	.	.	.	.	.	.
7026	0,5	TMBIM4	NM_016056	chr12	64816983	64850074	-	.	.	.	.	.	.	.	.	.	.	.	.	.	.	.	.	.	.	.	.
2207	1,5	IRAK3	NM_001142523	chr12	64869244	64934661	+	.	.	.	.	.	.	.	.	.	.	.	.	.	.	.	.	.	.	.	.
4028	1	HELB	NM_033647	chr12	64982601	65023690	+	.	.	.	.	.	.	.	.	.	.	.	.	.	.	.	.	.	.	.	.
7336	0,25	GRIP1	NM_021150	chr12	65027477	65359192	-	.	.	.	.	.	.	.	.	.	.	.	.	.	.	.	.	.	.	.	.
6127	0,5	CAND1	NM_018448	chr12	65949327	65994655	+	.	.	.	.	.	.	.	.	.	.	.	.	.	.	.	.	.	.	.	.
2116	1,5	DYRK2	NM_006482	chr12	66328778	66342711	+	.	.	.	.	.	.	.	.	.	.	.	.	.	.	.	.	.	.	.	.
214	4,25	IFNG	NM_000619	chr12	66834816	66839788	-	.	.	.	.	.	.	.	.	.	.	.	.	.	.	.	.	.	.	.	.
7498	0,25	RAP1B	NM_001251917	chr12	67290885	67340653	+	AY517227.1	chr12	67331527	-	.	.	.	.	.	.	.	.	.	.	.	.	.	.	.	.
2700	1,25	NUP107	NM_020401	chr12	67366997	67422740	+	.	.	.	.	.	.	.	.	.	.	.	.	.	.	.	.	.	.	.	.
1165	2,25	SLC35E3	NM_018656	chr12	67426202	67446120	+	.	.	.	.	.	.	.	.	.	.	.	.	.	.	.	.	.	.	.	.
12	10,75	MDM2	NM_002392	chr12	67488237	67525587	+	.	.	.	.	.	.	.	.	.	.	.	.	.	.	.	.	1312_139_1	chr12	67511427	+
2090	1,5	CPM	NM_001005502	chr12	67531222	67613246	-	.	.	.	.	.	.	.	.	.	.	.	.	.	.	.	.	.	.	.	.
6586	0,5	LYZ	NM_000239	chr12	68028400	68034280	+	.	.	.	.	.	.	.	.	.	.	.	.	.	.	.	.	.	.	.	.
5722	1	YEATS4	NM_006530	chr12	68039798	68070843	+	.	.	.	.	.	.	.	.	.	.	.	.	.	.	.	.	.	.	.	.
836	2,5	FRS2	NM_006654	chr12	68150395	68259829	+	.	.	.	.	.	.	.	.	.	.	.	.	.	.	.	.	.	.	.	.
6143	0,5	CCT2	NM_001198842	chr12	68265728	68281624	+	.	.	.	.	.	.	.	.	.	.	.	.	.	.	.	.	.	.	.	.
7404	0,25	LRRC10	NM_201550	chr12	68288611	68291209	-	.	.	.	.	.	.	.	.	.	.	.	.	.	.	.	.	.	.	.	.
6504	0,5	KCNMB4	NM_014505	chr12	69046328	69114339	+	.	.	.	.	.	.	.	.	.	.	.	.	.	.	.	.	.	.	.	.
227	4,25	PTPRB	NM_001206971	chr12	69196898	69289891	-	.	.	.	.	.	.	.	.	.	.	.	.	.	.	.	.	.	.	.	.
2736	1,25	PTPRR	NM_130846	chr12	69318119	69434655	-	.	.	.	.	.	.	.	.	.	.	.	.	.	.	.	.	.	.	.	.
5530	1	TPH2	NM_173353	chr12	70618892	70712488	+	.	.	.	.	.	.	.	.	.	.	.	.	.	.	.	.	.	.	.	.
4199	1	KCNC2	NM_139136	chr12	73720124	73889795	-	.	.	.	.	.	.	.	.	.	.	.	.	.	.	.	.	.	.	.	.
4247	1	KRR1	NM_007043	chr12	74177685	74191685	-	.	.	.	.	.	.	.	.	.	.	.	.	.	.	.	.	.	.	.	.
6741	0,5	PHLDA1	NM_007350	chr12	74705493	74711823	-	.	.	.	.	.	.	.	.	.	.	.	.	.	.	.	.	.	.	.	.
6226	0,5	CSRP2	NM_001321	chr12	75776626	75796930	-	.	.	.	.	.	.	.	.	.	.	.	.	.	.	.	.	.	.	.	.

Table S2

tumor associated genes								HIV				MLV				MMTV				MMTV(SIN)				MMTV(SIN)arrest			
rank	score	gene symbol	RefSEq	chrom	txStart	txEnd	strand	integrant	chr	position	strand	integrant	chr	position	strand	integrant	chr	position	strand	integrant	chr	position	strand	integrant	chr	position	strand
6292	0,5	E2F7	NM_203394	chr12	75939156	75983491	-	.	.	.	.	.	.	.	.	.	.	.	.	.	.	.	.	.	.	.	.
283	4	NAV3	NM_014903	chr12	76749199	77130921	+	.	.	.	.	.	.	.	.	.	.	.	.	.	.	.	.	.	.	.	.
2794	1,25	SYT1	NM_005639	chr12	77782579	78369919	+	BH609683	chr12	78002906	+	.	.	.	.	_139_1_136	chr12	77783442	+	.	.	.	.	.	.	.	.
4908	1	PPP1R12A	NM_001244992	chr12	78691473	78852862	-	CL529478	chr12	78844436	-	.	.	.	.	.	.	.	.	.	.	.	.	.	.	.	.
4908	1	PPP1R12A	NM_001244992	chr12	78691473	78852862	-	CL529476	chr12	78713993	+	.	.	.	.	.	.	.	.	.	.	.	.	.	.	.	.
4908	1	PPP1R12A	NM_001244992	chr12	78691473	78852862	-	CL529477	chr12	78727358	-	.	.	.	.	.	.	.	.	.	.	.	.	.	.	.	.
4908	1	PPP1R12A	NM_001244992	chr12	78691473	78852862	-	CL800665	chr12	78715633	-	.	.	.	.	.	.	.	.	.	.	.	.	.	.	.	.
7398	0,25	LIN7A	NM_004664	chr12	79715301	79855825	-	.	.	.	.	.	.	.	.	.	.	.	.	.	.	.	.	.	.	.	.
2893	1	ACSS3	NM_024560	chr12	79995939	80173713	+	.	.	.	.	.	.	.	.	.	.	.	.	.	.	.	.	.	.	.	.
930	2,5	PPFIA2	NM_001220479	chr12	80176175	80287324	-	.	.	.	.	.	.	.	.	.	.	.	.	.	.	.	.	.	.	.	.
6688	0,5	NTS	NM_006183	chr12	84792203	84800901	+	.	.	.	.	.	.	.	.	.	.	.	.	.	.	.	.	.	.	.	.
6610	0,5	MGAT4C	NM_013244	chr12	84897167	85756812	-	.	.	.	.	.	.	.	.	.	.	.	.	.	.	.	.	.	.	.	.
2634	1,25	KITLG	NM_003994	chr12	87410700	87498381	-	.	.	.	.	.	.	.	.	.	.	.	.	.	.	.	.	.	.	.	.
3630	1	DUSP6	NM_022652	chr12	88265967	88270427	-	.	.	.	.	.	.	.	.	.	.	.	.	.	.	.	.	.	.	.	.
2149	1,5	GALNT4	NM_003774	chr12	88437320	88442714	-	.	.	.	.	.	.	.	.	.	.	.	.	.	.	.	.	.	.	.	.
7178	0,25	ATP2B1	NM_001682	chr12	88505956	88573975	-	AY517100.1	chr12	88571776	-	.	.	.	.	.	.	.	.	.	.	.	.	.	.	.	.
7178	0,25	ATP2B1	NM_001682	chr12	88505956	88573975	-	CL800055	chr12	88517301	+	.	.	.	.	.	.	.	.	.	.	.	.	.	.	.	.
4317	1	LUM	NM_002345	chr12	90021362	90029673	-	.	.	.	.	.	.	.	.	.	.	.	.	.	.	.	.	.	.	.	.
556	3	DCN	NM_133503	chr12	90063165	90097490	-	.	.	.	.	.	.	.	.	.	.	.	.	.	.	.	.	.	.	.	.
535	3	BTG1	NM_001731	chr12	91058184	91063804	-	.	.	.	.	.	.	.	.	.	.	.	.	.	.	.	.	.	.	.	.
5607	1	UBE2N	NM_003348	chr12	92326218	92360157	-	.	.	.	.	.	.	.	.	.	.	.	.	.	.	.	.	.	.	.	.
306	4	SOCS2	NM_001270468	chr12	92488887	92494652	+	.	.	.	.	.	.	.	.	.	.	.	.	.	.	.	.	.	.	.	.
4525	1	NDUFA12	NM_001258338	chr12	93889234	93921620	-	AY517387.1	chr12	93918283	+	.	.	.	.	.	.	.	.	.	.	.	.	.	.	.	.
4610	1	NR2C1	NM_001032287	chr12	93948315	93991535	-	.	.	.	.	.	.	.	.	.	.	.	.	.	.	.	.	.	.	.	.
2673	1,25	METAP2	NM_006838	chr12	94391952	94433744	+	AY517044.1	chr12	94414320	-	.	.	.	.	.	.	.	.	.	.	.	.	.	.	.	.
5659	1	USP44	NM_032147	chr12	94435017	94469394	-	.	.	.	.	.	.	.	.	.	.	.	.	.	.	.	.	.	.	.	.
2974	1	AMDHD1	NM_152435	chr12	94861201	94886501	+	.	.	.	.	.	.	.	.	.	.	.	.	.	.	.	.	.	.	.	.
4009	1	HAL	NM_001258333	chr12	94890570	94914274	-	.	.	.	.	.	.	.	.	.	.	.	.	.	.	.	.	.	.	.	.
995	2,25	APAF1	NM_181861	chr12	97563208	97653342	+	.	.	.	.	.	.	.	.	.	.	.	.	.	.	.	.	.	.	.	.
6017	0,5	ANKS1B	NM_152788	chr12	97652699	98902563	-	.	.	.	.	AY516111	chr12	98829720	+	_289_1_187	chr12	97767596	+	64_1_2161_8	chr12	98481357	+	.	.	.	.
6905	0,5	SLC17A8	NM_139319	chr12	99274987	99339968	+	.	.	.	.	.	.	.	.	.	.	.	.	.	.	.	.	.	.	.	.
2698	1,25	NR1H4	NM_001206992	chr12	99421268	99481776	+	.	.	.	.	.	.	.	.	.	.	.	.	.	.	.	.	.	.	.	.
6018	0,5	ANO4	NM_178826	chr12	99712504	100046550	+	.	.	.	.	.	.	.	.	_447_1_297	chr12	99803484	-	.	.	.	.	.	.	.	.
5282	1	SLC5A8	NM_145913	chr12	100074124	100128147	-	.	.	.	.	.	.	.	.	.	.	.	.	.	.	.	.	.	.	.	.
3032	1	ARL1	NM_001177	chr12	100311033	100325703	-	.	.	.	.	.	.	.	.	.	.	.	.	.	.	.	.	.	.	.	.
4469	1	MYBPC1	NM_001254719	chr12	100512839	100603789	+	.	.	.	.	.	.	.	.	.	.	.	.	.	.	.	.	.	.	.	.
3358	1	CHPT1	NM_020244	chr12	100615547	100646977	+	.	.	.	.	.	.	.	.	.	.	.	.	.	.	.	.	.	.	.	.
2294	1,5	NUP37	NM_024057	chr12	100992102	101036491	-	CL799571	chr12	101017427	-	.	.	.	.	.	.	.	.	.	.	.	.	.	.	.	.
475	3,25	IGF1	NM_001111283	chr12	101313774	101398508	-	.	.	.	.	.	.	.	.	.	.	.	.	.	.	.	.	.	.	.	.
4687	1	PAH	NM_000277	chr12	101756233	101835511	-	.	.	.	.	.	.	.	.	.	.	.	.	.	.	.	.	.	.	.	.
5878	0,75	ASCL1	NM_004316	chr12	101875581	101878424	+	.	.	.	.	.	.	.	.	.	.	.	.	.	.	.	.	.	.	.	.
1457	2	HSP90B1	NM_003299	chr12	102848241	102865838	+	.	.	.	.	.	.	.	.	.	.	.	.	.	.	.	.	.	.	.	.
5448	1	TDG	NM_003211	chr12	102883722	102906786	+	.	.	.	.	.	.	.	.	.	.	.	.	.	.	.	.	.	.	.	.
5593	1	TXNRD1	NM_003330	chr12	103204589	103268216	+	.	.	.	.	AY516454	chr12	103199827	-	.	.	.	.	.	.	.	.	.	.	.	.
3663	1	EID3	NM_001008394	chr12	103221639	103223112	+	.	.	.	.	.	.	.	.	.	.	.	.	.	.	.	.	.	.	.	.
3361	1	CHST11	NM_018413	chr12	103374821	103679922	+	.	.	.	.	.	.	.	.	.	.	.	.	.	.	.	.	.	.	.	.
2291	1,5	NUAK1	NM_014840	chr12	104981254	105057941	-	.	.	.	.	.	.	.	.	.	.	.	.	.	.	.	.	2895_259_2	chr12	104996880	-
6185	0,5	CKAP4	NM_006825	chr12	105155788	105165843	-	.	.	.	.	.	.	.	.	.	.	.	.	.	.	.	.	.	.	.	.
1657	2	POLR3B	NM_018082	chr12	105275565	105428106	+	.	.	.	.	.	.	.	.	.	.	.	.	.	.	.	.	.	.	.	.
3053	1	ASCL4	NM_203436	chr12	106692291	106694551	+	.	.	.	.	.	.	.	.	.	.	.	.	.	.	.	.	.	.	.	.
7102	0,5	WSCD2	NM_014653	chr12	107047640	107168443	+	.	.	.	.	.	.	.	.	.	.	.	.	.	.	.	.	.	.	.	.

Table S2

tumor associated genes								HIV				MLV				MMTV				MMTV(SIN)				MMTV(SIN)arrest			
rank	score	gene symbol	RefSEq	chrom	txStart	txEnd	strand	integrant	chr	position	strand	integrant	chr	position	strand	integrant	chr	position	strand	integrant	chr	position	strand	integrant	chr	position	strand
7533	0,25	SELPLG	NM_001206609	chr12	107539808	107549983	-	.	.	.	.	.	.	.	.	.	.	.	.	.	.	.	.	.	.	.	.
3527	1	DAO	NM_001917	chr12	107797985	107818839	+	.	.	.	.	.	.	.	.	.	.	.	.	.	.	.	.	.	.	.	.
6991	0,5	SVOP	NM_018711	chr12	107828786	107943228	-	.	.	.	.	.	.	.	.	.	.	.	.	.	.	.	.	.	.	.	.
2966	1	ALKBH2	NM_001145375	chr12	108010375	108015676	-	.	.	.	.	.	.	.	.	.	.	.	.	.	.	.	.	.	.	.	.
1977	1,75	UNG	NM_003362	chr12	108020305	108033181	+	.	.	.	.	.	.	.	.	.	.	.	.	.	.	.	.	.	.	.	.
2867	1	ACACB	NM_001093	chr12	108061584	108190413	+	.	.	.	.	.	.	.	.	.	.	.	.	.	.	.	.	.	.	.	.
4413	1	MMAB	NR_038118	chr12	108475903	108495741	-	.	.	.	.	.	.	.	.	.	.	.	.	.	.	.	.	.	.	.	.
1549	2	MVK	NM_000431	chr12	108495882	108519454	+	.	.	.	.	.	.	.	.	.	.	.	.	.	.	.	.	.	.	.	.
3892	1	GIT2	NM_057170	chr12	108851989	108918577	-	.	.	.	.	.	.	.	.	.	.	.	.	.	.	.	.	.	.	.	.
2980	1	ANAPC7	NM_016238	chr12	109295087	109325918	-	.	.	.	.	.	.	.	.	.	.	.	.	.	.	.	.	.	.	.	.
1714	2	RAD9B	NM_152442	chr12	109424387	109454274	+	.	.	.	.	.	.	.	.	.	.	.	.	.	.	.	.	.	.	.	.
4931	1	PPTC7	NM_139283	chr12	109456619	109505447	-	.	.	.	.	.	.	.	.	.	.	.	.	.	.	.	.	.	.	.	.
2720	1,25	PPP1CC	NM_002710	chr12	109641995	109665166	-	AY516967.1	chr12	109649823	+	.	.	.	.	.	.	.	.	.	.	.	.	.	.	.	.
1753	2	SH2B3	NM_005475	chr12	110328134	110373810	+	.	.	.	.	.	.	.	.	.	.	.	.	.	.	.	.	.	.	.	.
3158	1	BRAP	NM_006768	chr12	110564332	110608173	-	.	.	.	.	.	.	.	.	.	.	.	.	.	.	.	.	.	.	.	.
779	2,5	ALDH2	NM_001204889	chr12	110689073	110732172	+	.	.	.	.	.	.	.	.	.	.	.	.	.	.	.	.	.	.	.	.
2665	1,25	MAPKAPK5	NM_139078	chr12	110764661	110815611	+	.	.	.	.	.	.	.	.	.	.	.	.	.	.	.	.	.	.	.	.
111	5,5	PTPN11	NM_002834	chr12	111340918	111432100	+	CL529114	chr12	111426664	+	.	.	.	.	.	.	.	.	.	.	.	.	.	.	.	.
111	5,5	PTPN11	NM_002834	chr12	111340918	111432100	+	CL800669	chr12	111366521	-	.	.	.	.	.	.	.	.	.	.	.	.	.	.	.	.
7518	0,25	RPH3A	NM_001143854	chr12	111713931	111821067	+	.	.	.	.	.	.	.	.	.	.	.	.	94_1_2417_11	chr12	111786360	-	.	.	.	.
816	2,5	DTX1	NM_004416	chr12	111980044	112020216	+	.	.	.	.	.	.	.	.	.	.	.	.	.	.	.	.	.	.	.	.
6796	0,5	RASAL1	NM_001193520	chr12	112021006	112058427	-	.	.	.	.	.	.	.	.	.	.	.	.	.	.	.	.	.	.	.	.
1350	2	DDX54	NM_001111322	chr12	112079360	112107667	-	.	.	.	.	.	.	.	.	.	.	.	.	.	.	.	.	.	.	.	.
5203	1	SDS	NM_006843	chr12	112314633	112326075	-	.	.	.	.	.	.	.	.	.	.	.	.	.	.	.	.	.	.	.	.
5441	1	TBX5	NM_181486	chr12	113276117	113328351	-	.	.	.	.	.	.	.	.	.	.	.	.	.	.	.	.	.	.	.	.
5439	1	TBX3	NM_016569	chr12	113592441	113606352	-	.	.	.	.	.	.	.	.	.	.	.	.	.	.	.	.	.	.	.	.
4375	1	MED13L	NM_015335	chr12	114880763	115199374	-	CL800211	chr12	115005421	+	.	.	.	.	.	.	.	.	.	.	.	.	.	.	.	.
4375	1	MED13L	NM_015335	chr12	114880763	115199374	-	BH609689	chr12	115090811	-	.	.	.	.	.	.	.	.	.	.	.	.	.	.	.	.
6826	0,5	RNFT2	NM_001109903	chr12	115660478	115775819	+	.	.	.	.	.	.	.	.	_318_1_200	chr12	115681276	-	.	.	.	.	.	.	.	.
3762	1	FBXW8	NM_153348	chr12	115833143	115953336	+	.	.	.	.	.	.	.	.	.	.	.	.	.	.	.	.	.	.	.	.
7014	0,5	TESC	NM_017899	chr12	115961110	116021634	-	.	.	.	.	.	.	.	.	.	.	.	.	.	.	.	.	.	.	.	.
2138	1,5	FBXO21	NM_015002	chr12	116065967	116112683	-	.	.	.	.	.	.	.	.	.	.	.	.	.	.	.	.	.	.	.	.
488	3,25	NOS1	NM_001204213	chr12	116130329	116227240	-	.	.	.	.	.	.	.	.	.	.	.	.	.	.	.	.	.	.	.	.
1497	2	KSR2	NM_173598	chr12	116375199	116890411	-	.	.	.	.	.	.	.	.	.	.	.	.	.	.	.	.	.	.	.	.
5101	1	RFC5	NM_001206801	chr12	116938888	116954425	+	.	.	.	.	.	.	.	.	.	.	.	.	.	.	.	.	.	.	.	.
7472	0,25	PEBP1	NM_002567	chr12	117058252	117067773	+	.	.	.	.	.	.	.	.	.	.	.	.	.	.	.	.	.	.	.	.
2430	1,5	TAOK3	NM_016281	chr12	117071988	117295133	-	AY517195.1	chr12	117264230	-	AY516878	chr12	117279476	-	.	.	.	.	.	.	.	.	.	.	.	.
2430	1,5	TAOK3	NM_016281	chr12	117071988	117295133	-	CL800267	chr12	117071992	-	AY516878	chr12	117279476	-	.	.	.	.	.	.	.	.	.	.	.	.
6455	0,5	HSPB8	NM_014365	chr12	118100977	118116934	+	.	.	.	.	.	.	.	.	.	.	.	.	.	.	.	.	.	.	.	.
1683	2	PRKAB1	NM_006253	chr12	118590143	118603812	+	.	.	.	.	.	.	.	.	.	.	.	.	.	.	.	.	.	.	.	.
363	3,5	CIT	NM_007174	chr12	118607977	118799478	-	.	.	.	.	.	.	.	.	.	.	.	.	.	.	.	.	.	.	.	.
5031	1	RAB35	NM_006861	chr12	119017281	119039026	-	.	.	.	.	.	.	.	.	.	.	.	.	.	.	.	.	.	.	.	.
6377	0,5	GCN1L1	NM_006836	chr12	119049396	119116896	-	.	.	.	.	.	.	.	.	.	.	.	.	.	.	.	.	.	.	.	.
6829	0,5	RPLP0	NM_001002	chr12	119118885	119123397	-	.	.	.	.	.	.	.	.	.	.	.	.	.	.	.	.	.	.	.	.
1705	2	PXN	NM_001080855	chr12	119132624	119187957	-	.	.	.	.	.	.	.	.	.	.	.	.	.	.	.	.	.	.	.	.
4828	1	PLA2G1B	NM_000928	chr12	119244296	119249975	-	.	.	.	.	.	.	.	.	.	.	.	.	.	.	.	.	.	.	.	.
4445	1	MSI1	NM_002442	chr12	119263515	119291366	-	.	.	.	.	.	.	.	.	.	.	.	.	.	.	.	.	.	.	.	.
3436	1	COX6A1	NM_004373	chr12	119360275	119362928	+	.	.	.	.	.	.	.	.	.	.	.	.	.	.	.	.	.	.	.	.
3425	1	COQ5	NM_032314	chr12	119425464	119451347	-	.	.	.	.	.	.	.	.	.	.	.	.	.	.	.	.	.	.	.	.
3257	1	CABP1	NM_004276	chr12	119572692	119589510	+	.	.	.	.	.	.	.	.	.	.	.	.	.	.	.	.	.	.	.	.
6615	0,5	MLEC	NM_014730	chr12	119609331	119624050	+	.	.	.	.	.	.	.	.	.	.	.	.	.	.	.	.	2737_242_1	chr12	119619604	-

Table S2

tumor associated genes								HIV				MLV				MMTV				MMTV(SIN)				MMTV(SIN)arrest			
rank	score	gene symbol	RefSEq	chrom	txStart	txEnd	strand	integrant	chr	position	strand	integrant	chr	position	strand	integrant	chr	position	strand	integrant	chr	position	strand	integrant	chr	position	strand
2871	1	ACADS	NM_000017	chr12	119647953	119662194	+	.	.	.	.	.	.	.	.	.	.	.	.	.	.	.	.	.	.	.	.
472	3,25	HNF1A	NM_000545	chr12	119900931	119924697	+	.	.	.	.	.	.	.	.	.	.	.	.	.	.	.	.	.	.	.	.
911	2,5	P2RX7	NR_033955	chr12	120055013	120108737	+	.	.	.	.	.	.	.	.	.	.	.	.	.	.	.	.	.	.	.	.
1884	1,75	CAMKK2	NM_153500	chr12	120159877	120220494	-	.	.	.	.	.	.	.	.	.	.	.	.	.	.	.	.	.	.	.	.
235	4	ANAPC5	NM_016237	chr12	120230430	120274648	-	.	.	.	.	.	.	.	.	.	.	.	.	.	.	.	.	1094_130_1	chr12	120275076	+
5118	1	RHOF	NM_019034	chr12	120700042	120715977	-	.	.	.	.	.	.	.	.	.	.	.	.	.	.	.	.	.	.	.	.
4076	1	HPD	NM_002150	chr12	120761815	120781152	-	.	.	.	.	.	.	.	.	.	.	.	.	.	.	.	.	.	.	.	.
794	2,5	BCL7A	NM_020993	chr12	120944243	120984333	+	.	.	.	.	.	.	.	.	.	.	.	.	.	.	.	.	.	.	.	.
6616	0,5	MLXIP	NM_014938	chr12	121001034	121194920	+	.	.	.	.	.	.	.	.	.	.	.	.	.	.	.	.	.	.	.	.
6474	0,5	IL31	NM_001014336	chr12	121222529	121224699	-	.	.	.	.	.	.	.	.	.	.	.	.	.	.	.	.	.	.	.	.
2019	1,5	B3GNT4	NM_030765	chr12	121254180	121258037	+	.	.	.	.	.	.	.	.	.	.	.	.	.	.	.	.	.	.	.	.
2567	1,25	DIABLO	NM_019887	chr12	121258161	121278021	-	.	.	.	.	.	.	.	.	.	.	.	.	.	.	.	.	.	.	.	.
3389	1	CLIP1	NM_198240	chr12	121321933	121473069	-	.	.	.	.	AY516552	chr12	121451008	-	.	.	.	.	.	.	.	.	.	.	.	.
1495	2	KNTC1	NM_014708	chr12	121577761	121676900	+	CL800729	chr12	121619921	-	.	.	.	.	.	.	.	.	.	.	.	.	.	.	.	.
4816	1	PITPNM2	NM_020845	chr12	122033979	122160928	-	.	.	.	.	.	.	.	.	.	.	.	.	.	.	.	.	.	.	.	.
7433	0,25	MPHOSPH9	NM_022782	chr12	122206898	122272394	-	CL800163	chr12	122257849	+	.	.	.	.	.	.	.	.	.	.	.	.	.	.	.	.
3314	1	CDK2AP1	NM_001270433	chr12	122311469	122320734	-	.	.	.	.	.	.	.	.	.	.	.	.	.	.	.	.	.	.	.	.
5188	1	SBNO1	NM_018183	chr12	122339608	122400941	-	.	.	.	.	.	.	.	.	.	.	.	.	.	.	.	.	.	.	.	.
5214	1	SETD8	NM_020382	chr12	122434656	122459853	+	.	.	.	.	.	.	.	.	.	.	.	.	.	.	.	.	.	.	.	.
3554	1	DDX55	NM_020936	chr12	122652624	122671435	+	.	.	.	.	.	.	.	.	.	.	.	.	.	.	.	.	.	.	.	.
3987	1	GTF2H3	NM_001516	chr12	122684333	122711287	+	.	.	.	.	.	.	.	.	.	.	.	.	.	.	.	.	.	.	.	.
3083	1	ATP6V0A2	NM_012463	chr12	122762817	122812254	+	.	.	.	.	.	.	.	.	.	.	.	.	.	.	.	.	.	.	.	.
403	3,5	NCOR2	NM_006312	chr12	123374909	123617963	-	.	.	.	.	.	.	.	.	.	.	.	.	.	.	.	.	.	.	.	.
1981	1,5	AACS	NM_023928	chr12	124115877	124193824	+	.	.	.	.	.	.	.	.	.	.	.	.	.	.	.	.	.	.	.	.
7031	0,5	TMEM132B	NM_052907	chr12	124377114	124709542	+	.	.	.	.	.	.	.	.	.	.	.	.	.	.	.	.	.	.	.	.
3904	1	GLT1D1	NM_144669	chr12	127904033	128035462	+	.	.	.	.	.	.	.	.	.	.	.	.	.	.	.	.	.	.	.	.
7032	0,5	TMEM132D	NM_133448	chr12	128122223	128954165	-	.	.	.	.	.	.	.	.	.	.	.	.	.	.	.	.	.	.	.	.
1416	2	FZD10	NM_007197	chr12	129212956	129216238	+	.	.	.	.	.	.	.	.	.	.	.	.	.	.	.	.	.	.	.	.
6818	0,5	RIMBP2	NM_015347	chr12	129446633	129568363	-	.	.	.	.	.	.	.	.	.	.	.	.	110_1_944_29	chr12	129546369	+	.	.	.	.
943	2,5	RAN	NM_006325	chr12	129922569	129926779	+	.	.	.	.	.	.	.	.	.	.	.	.	.	.	.	.	.	.	.	.
3947	1	GPR133	NM_198827	chr12	130004404	130191961	+	.	.	.	.	.	.	.	.	.	.	.	.	.	.	.	.	.	.	.	.
2821	1,25	ULK1	NM_003565	chr12	130945231	130973660	+	.	.	.	.	.	.	.	.	.	.	.	.	.	.	.	.	.	.	.	.
7497	0,25	PUS1	NM_001002020	chr12	130979697	130994359	+	.	.	.	.	.	.	.	.	.	.	.	.	.	.	.	.	.	.	.	.
251	4	EP400	NM_015409	chr12	131000417	131130964	+	CL799936	chr12	131014695	+	.	.	.	.	.	.	.	.	1587_228	chr12	131097151	-	.	.	.	.
251	4	EP400	NM_015409	chr12	131000417	131130964	+	AY517351.1	chr12	131015901	+	.	.	.	.	.	.	.	.	1587_228	chr12	131097151	-	.	.	.	.
3552	1	DDX51	NM_175066	chr12	131187092	131194833	-	.	.	.	.	.	.	.	.	.	.	.	.	.	.	.	.	.	.	.	.
6376	0,5	GALNT9	NM_001122636	chr12	131246869	131415978	-	.	.	.	.	.	.	.	.	.	.	.	.	.	.	.	.	.	.	.	.
290	4	POLE	NM_006231	chr12	131710420	131774018	-	CL799884	chr12	131742234	+	.	.	.	.	.	.	.	.	.	.	.	.	.	.	.	.
290	4	POLE	NM_006231	chr12	131710420	131774018	-	CL799978	chr12	131766493	+	.	.	.	.	.	.	.	.	.	.	.	.	.	.	.	.
6391	0,5	GOLGA3	NM_005895	chr12	131855567	131915361	-	.	.	.	.	.	.	.	.	.	.	.	.	.	.	.	.	.	.	.	.
7244	0,25	CHFR	NM_018223	chr12	131927010	131974277	-	.	.	.	.	.	.	.	.	.	.	.	.	.	.	.	.	.	.	.	.
1818	2	TPTE2	NM_001141968	chr13	18895018	19008903	-	.	.	.	.	.	.	.	.	.	.	.	.	.	.	.	.	.	.	.	.
4429	1	MPHOSPH8	NM_017520	chr13	19105787	19145599	+	.	.	.	.	.	.	.	.	.	.	.	.	.	.	.	.	.	.	.	.
4975	1	PSPC1	NR_003272	chr13	19146891	19255159	-	.	.	.	.	.	.	.	.	.	.	.	.	.	.	.	.	.	.	.	.
5753	1	ZMYM5	NM_001039649	chr13	19309592	19335776	-	.	.	.	.	.	.	.	.	.	.	.	.	.	.	.	.	.	.	.	.
704	3	ZMYM2	NM_003453	chr13	19430809	19563968	+	.	.	.	.	.	.	.	.	.	.	.	.	.	.	.	.	.	.	.	.
6384	0,5	GJB6	NM_001110219	chr13	19694100	19704534	-	.	.	.	.	.	.	.	.	.	.	.	.	.	.	.	.	.	.	.	.
2093	1,5	CRYL1	NM_015974	chr13	19875805	19998012	-	.	.	.	.	.	.	.	.	.	.	.	.	.	.	.	.	.	.	.	.
4121	1	IFT88	NM_006531	chr13	20039207	20163576	+	.	.	.	.	.	.	.	.	.	.	.	.	.	.	.	.	.	.	.	.
4131	1	IL17D	NM_138284	chr13	20175481	20195237	+	.	.	.	.	.	.	.	.	.	.	.	.	.	.	.	.	.	.	.	.
4486	1	N6AMT2	NM_174928	chr13	20201072	20246057	-	.	.	.	.	.	.	.	.	.	.	.	.	.	.	.	.	.	.	.	.

Table S2

tumor associated genes								HIV				MLV				MMTV				MMTV(SIN)				MMTV(SIN)arrest			
rank	score	gene symbol	RefSEq	chrom	txStart	txEnd	strand	integrant	chr	position	strand	integrant	chr	position	strand	integrant	chr	position	strand	integrant	chr	position	strand	integrant	chr	position	strand
5715	1	XPO4	NM_022459	chr13	20249467	20374913	-	BH609691	chr13	20271986	-	.	.	.	.	.	.	.	.	.	.	.	.	.	.	.	.
737	2,75	LATS2	NM_014572	chr13	20445175	20533722	-	.	.	.	.	.	.	.	.	.	.	.	.	.	.	.	.	.	.	.	.
5179	1	SAP18	NM_005870	chr13	20612652	20621224	+	.	.	.	.	.	.	.	.	.	.	.	.	.	.	.	.	.	.	.	.
4437	1	MRP63	NM_024026	chr13	20648371	20651220	+	.	.	.	.	.	.	.	.	.	.	.	.	.	.	.	.	.	.	.	.
5737	1	ZDHHC20	NM_153251	chr13	20844709	20931508	-	.	.	.	.	.	.	.	.	.	.	.	.	.	.	.	.	.	.	.	.
3652	1	EFHA1	NM_152726	chr13	20964827	21076355	-	CL529649	chr13	20975504	-	.	.	.	.	.	.	.	.	.	.	.	.	.	.	.	.
3652	1	EFHA1	NM_152726	chr13	20964827	21076355	-	CL529650	chr13	21052759	+	.	.	.	.	.	.	.	.	.	.	.	.	.	.	.	.
3652	1	EFHA1	NM_152726	chr13	20964827	21076355	-	AY516992.1	chr13	21048406	-	.	.	.	.	.	.	.	.	.	.	.	.	.	.	.	.
468	3,25	FGF9	NM_002010	chr13	21143214	21176640	+	.	.	.	.	.	.	.	.	.	.	.	.	.	.	.	.	.	.	.	.
1738	2	SACS	NM_014363	chr13	22800964	22905841	-	.	.	.	.	.	.	.	.	.	.	.	.	.	.	.	.	.	.	.	.
2447	1,5	TNFRSF19	NM_001204458	chr13	23051498	23148244	+	.	.	.	.	.	.	.	.	.	.	.	.	.	.	.	.	.	.	.	.
4403	1	MIPEP	NM_005932	chr13	23202327	23361587	-	.	.	.	.	AY516516	chr13	23340728	+	.	.	.	.	.	.	.	.	.	.	.	.
5324	1	SPATA13	NM_001166271	chr13	23632860	23779212	+	.	.	.	.	.	.	.	.	.	.	.	.	.	.	.	.	.	.	.	.
3215	1	C1QTNF9	NM_178540	chr13	23781715	23794669	+	.	.	.	.	.	.	.	.	.	.	.	.	.	.	.	.	.	.	.	.
1595	2	PARP4	NM_006437	chr13	23893068	23984948	-	.	.	.	.	.	.	.	.	.	.	.	.	.	.	.	.	.	.	.	.
3063	1	ATP12A	NM_001185085	chr13	24152548	24183923	+	.	.	.	.	.	.	.	.	.	.	.	.	.	.	.	.	.	.	.	.
3332	1	CENPJ	NM_018451	chr13	24354411	24395027	-	.	.	.	.	.	.	.	.	.	.	.	.	.	.	.	.	.	.	.	.
2131	1,5	FAM123A	NM_152704	chr13	24640671	24643857	-	.	.	.	.	.	.	.	.	.	.	.	.	.	.	.	.	.	.	.	.
1548	2	MTMR6	NM_004685	chr13	24718340	24759704	-	.	.	.	.	.	.	.	.	.	.	.	.	.	.	.	.	.	.	.	.
910	2,5	NUPL1	NM_014089	chr13	24773665	24814561	+	.	.	.	.	.	.	.	.	.	.	.	.	.	.	.	.	.	.	.	.
1271	2	ATP8A2	NM_016529	chr13	24844208	25493420	+	.	.	.	.	.	.	.	.	.	.	.	.	604_114	chr13	25219892	+	631_113_2	chr13	24893374	+
5143	1	RNF6	NM_183044	chr13	25684904	25693894	-	.	.	.	.	.	.	.	.	.	.	.	.	.	.	.	.	.	.	.	.
542	3	CDK8	NM_001260	chr13	25726755	25876569	+	.	.	.	.	.	.	.	.	.	.	.	.	.	.	.	.	.	.	.	.
2459	1,5	WASF3	NM_006646	chr13	26029839	26161082	+	.	.	.	.	.	.	.	.	.	.	.	.	.	.	.	.	.	.	.	.
3946	1	GPR12	NM_005288	chr13	26227338	26232922	-	.	.	.	.	.	.	.	.	.	.	.	.	.	.	.	.	.	.	.	.
5653	1	USP12	NM_182488	chr13	26538286	26644033	-	.	.	.	.	.	.	.	.	.	.	.	.	.	.	.	.	.	.	.	.
5161	1	RPL21	NM_000982	chr13	26723691	26728702	+	.	.	.	.	.	.	.	.	.	.	.	.	.	.	.	.	.	.	.	.
3989	1	GTF3A	NM_002097	chr13	26896680	26907846	+	.	.	.	.	.	.	.	.	.	.	.	.	.	.	.	.	.	.	.	.
4456	1	MTIF3	NM_152912	chr13	26907775	26922739	-	.	.	.	.	.	.	.	.	.	.	.	.	.	.	.	.	.	.	.	.
1506	2	LNX2	NM_153371	chr13	27018049	27092720	-	.	.	.	.	.	.	.	.	.	.	.	.	.	.	.	.	.	.	.	.
646	3	POLR1D	NM_152705	chr13	27093915	27139559	+	.	.	.	.	.	.	.	.	.	.	.	.	.	.	.	.	.	.	.	.
3984	1	GSX1	NM_145657	chr13	27264779	27266089	+	.	.	.	.	.	.	.	.	.	.	.	.	.	.	.	.	.	.	.	.
7471	0,25	PDX1	NM_000209	chr13	27392167	27398451	+	.	.	.	.	.	.	.	.	.	.	.	.	.	.	.	.	.	.	.	.
204	4,25	CDX2	NM_001265	chr13	27434204	27441505	-	.	.	.	.	.	.	.	.	.	.	.	.	.	.	.	.	.	.	.	.
96	5,75	FLT3	NM_004119	chr13	27475410	27572729	-	.	.	.	.	.	.	.	.	.	.	.	.	.	.	.	.	.	.	.	.
4692	1	PAN3	NM_175854	chr13	27610642	27767475	+	CL800410	chr13	27658591	-	.	.	.	.	.	.	.	.	.	.	.	.	.	.	.	.
4692	1	PAN3	NM_175854	chr13	27610642	27767475	+	AY516898.1	chr13	27647784	-	.	.	.	.	.	.	.	.	.	.	.	.	.	.	.	.
4692	1	PAN3	NM_175854	chr13	27610642	27767475	+	CL799766	chr13	27747288	-	.	.	.	.	.	.	.	.	.	.	.	.	.	.	.	.
118	5,25	FLT1	NM_001160031	chr13	27871180	27967265	-	.	.	.	.	.	.	.	.	.	.	.	.	.	.	.	.	.	.	.	.
4875	1	POMP	NM_015932	chr13	28131140	28151093	+	.	.	.	.	.	.	.	.	.	.	.	.	.	.	.	.	.	.	.	.
5285	1	SLC7A1	NM_003045	chr13	28981550	29067825	-	.	.	.	.	.	.	.	.	.	.	.	.	.	.	.	.	.	.	.	.
5611	1	UBL3	NM_007106	chr13	29236544	29322820	-	.	.	.	.	.	.	.	.	.	.	.	.	.	.	.	.	.	.	.	.
4196	1	KATNAL1	NM_032116	chr13	29674766	29779624	-	.	.	.	.	.	.	.	.	.	.	.	.	.	.	.	.	.	.	.	.
380	3,5	HMGB1	NM_002128	chr13	29930878	29938081	-	.	.	.	.	.	.	.	.	.	.	.	.	.	.	.	.	.	.	.	.
5662	1	USPL1	NM_005800	chr13	30089829	30131686	+	.	.	.	.	.	.	.	.	.	.	.	.	.	.	.	.	.	.	.	.
4098	1	HSPH1	NM_006644	chr13	30608762	30634117	-	.	.	.	.	.	.	.	.	.	.	.	.	.	.	.	.	.	.	.	.
3110	1	B3GALTL	NM_194318	chr13	30672111	30804411	+	.	.	.	.	.	.	.	.	.	.	.	.	.	.	.	.	.	.	.	.
3806	1	FRY	NM_023037	chr13	31503436	31768776	+	CL799621	chr13	31663115	+	.	.	.	.	.	.	.	.	.	.	.	.	.	.	.	.
3806	1	FRY	NM_023037	chr13	31503436	31768776	+	CL800598	chr13	31554323	-	.	.	.	.	.	.	.	.	.	.	.	.	.	.	.	.
20	9,25	BRCA2	NM_000059	chr13	31787616	31871809	+	CL799599	chr13	31838174	+	.	.	.	.	.	.	.	.	.	.	.	.	.	.	.	.
4484	1	N4BP2L1	NM_052818	chr13	31872859	31900315	-	CL799626	chr13	31888078	+	.	.	.	.	.	.	.	.	.	.	.	.	.	.	.	.

Table S2

tumor associated genes								HIV				MLV				MMTV				MMTV(SIN)				MMTV(SIN)arrest			
rank	score	gene symbol	RefSEq	chrom	txStart	txEnd	strand	integrant	chr	position	strand	integrant	chr	position	strand	integrant	chr	position	strand	integrant	chr	position	strand	integrant	chr	position	strand
4485	1	N4BP2L2	NM_014887	chr13	31989029	32010932	-	CL799618	chr13	32006988	-	.	.	.	.	.	.	.	.	.	.	.	.	.	.	.	.
4746	1	PDS5B	NM_015032	chr13	32058563	32250158	+	CL529485	chr13	32128794	-	.	.	.	.	.	.	.	.	.	.	.	.	.	.	.	.
5373	1	STARD13	NM_178006	chr13	32575271	32757901	-	.	.	.	.	AY516419	chr13	32753851	+	.	.	.	.	.	.	.	.	.	.	.	.
5099	1	RFC3	NM_181558	chr13	33290205	33438695	+	.	.	.	.	.	.	.	.	.	.	.	.	.	.	.	.	.	.	.	.
2269	1,5	NBEA	NM_015678	chr13	34414423	35144873	+	CL799754	chr13	34588042	-	AY516365	chr13	35043981	+	_381_1_232	chr13	35067855	+	.	.	.	.	.	.	.	.
2269	1,5	NBEA	NM_015678	chr13	34414423	35144873	+	CL800264	chr13	34691845	+	AY516365	chr13	35043981	+	_381_1_232	chr13	35067855	+	.	.	.	.	.	.	.	.
2269	1,5	NBEA	NM_015678	chr13	34414423	35144873	+	CL799540	chr13	34646063	+	AY516365	chr13	35043981	+	_381_1_232	chr13	35067855	+	.	.	.	.	.	.	.	.
2234	1,5	MAB21L1	NM_005584	chr13	34945925	34948832	-	.	.	.	.	.	.	.	.	.	.	.	.	.	.	.	.	.	.	.	.
1898	1,75	DCLK1	NM_001195430	chr13	35318036	35327998	-	.	.	.	.	.	.	.	.	.	.	.	.	.	.	.	.	.	.	.	.
1009	2,25	CCNA1	NM_001111046	chr13	35903966	35915019	+	.	.	.	.	.	.	.	.	.	.	.	.	.	.	.	.	.	.	.	.
6936	0,5	SMAD9	NM_001127217	chr13	36316967	36392409	-	.	.	.	.	.	.	.	.	.	.	.	.	.	.	.	.	.	.	.	.
2961	1	ALG5	NM_001142364	chr13	36421907	36471504	-	.	.	.	.	.	.	.	.	.	.	.	.	.	.	.	.	2429_210_4	chr13	36438692	+
1327	2	CSNK1A1L	NM_145203	chr13	36575396	36577801	-	.	.	.	.	.	.	.	.	.	.	.	.	.	.	.	.	.	.	.	.
648	3	POSTN	NM_001135936	chr13	37034718	37070981	-	.	.	.	.	.	.	.	.	.	.	.	.	.	.	.	.	.	.	.	.
5621	1	UFM1	NM_016617	chr13	37821941	37835143	+	.	.	.	.	.	.	.	.	.	.	.	.	.	.	.	.	.	.	.	.
879	2,5	LHFP	NM_005780	chr13	38815028	39075356	-	.	.	.	.	.	.	.	.	.	.	.	.	.	.	.	.	.	.	.	.
119	5,25	FOXO1	NM_002015	chr13	40027800	40138734	-	.	.	.	.	.	.	.	.	.	.	.	.	.	.	.	.	.	.	.	.
822	2,5	ELF1	NM_172373	chr13	40404054	40491508	-	.	.	.	.	AY516503	chr13	40430927	+	.	.	.	.	.	.	.	.	.	.	.	.
719	2,75	DGKH	NM_178009	chr13	41520842	41701891	+	CL529362	chr13	41650137	-	.	.	.	.	.	.	.	.	.	.	.	.	2667_234_2	chr13	41533911	+
6003	0,5	AKAP11	NM_016248	chr13	41744288	41795403	+	.	.	.	.	.	.	.	.	.	.	.	.	.	.	.	.	.	.	.	.
5511	1	TNFSF11	NM_033012	chr13	42034871	42080149	+	.	.	.	.	.	.	.	.	.	.	.	.	.	.	.	.	.	.	.	.
1035	2,25	DNAJC15	NM_013238	chr13	42495361	42581306	+	CL799536	chr13	42543729	-	.	.	.	.	.	.	.	.	.	.	.	.	.	.	.	.
6875	0,5	SERP2	NM_001010897	chr13	43845977	43869850	+	.	.	.	.	.	.	.	.	.	.	.	.	.	.	.	.	.	.	.	.
2215	1,5	KCTD4	NM_198404	chr13	44664987	44673175	-	.	.	.	.	.	.	.	.	.	.	.	.	.	.	.	.	.	.	.	.
7254	0,25	CPB2	NM_001872	chr13	45525322	45577212	-	.	.	.	.	.	.	.	.	.	.	.	.	.	.	.	.	.	.	.	.
393	3,5	LCP1	NM_002298	chr13	45598058	45654460	-	.	.	.	.	.	.	.	.	.	.	.	.	.	.	.	.	.	.	.	.
2195	1,5	HTR2A	NM_001165947	chr13	46303677	46369212	-	.	.	.	.	.	.	.	.	.	.	.	.	.	.	.	.	.	.	.	.
5398	1	SUCLA2	NM_003850	chr13	47414791	47473463	-	.	.	.	.	.	.	.	.	.	.	.	.	.	.	.	.	.	.	.	.
7379	0,25	ITM2B	NM_021999	chr13	47705274	47734233	+	.	.	.	.	.	.	.	.	.	.	.	.	.	.	.	.	.	.	.	.
6	13,25	RB1	NM_000321	chr13	47775883	47954027	+	CL800699	chr13	47821099	-	.	.	.	.	.	.	.	.	.	.	.	.	.	.	.	.
5080	1	RCBTB2	NM_001268	chr13	47961099	48005317	-	.	.	.	.	.	.	.	.	.	.	.	.	.	.	.	.	.	.	.	.
5215	1	SETDB2	NM_031915	chr13	48916429	48967140	+	.	.	.	.	.	.	.	.	.	.	.	.	.	.	.	.	.	.	.	.
6739	0,5	PHF11	NM_001040444	chr13	48968528	49001118	+	.	.	.	.	.	.	.	.	.	.	.	.	.	.	.	.	.	.	.	.
3033	1	ARL11	NM_138450	chr13	49100435	49106009	+	.	.	.	.	.	.	.	.	.	.	.	.	.	.	.	.	.	.	.	.
7375	0,25	INTS6	NM_001039938	chr13	50921621	50925276	-	.	.	.	.	.	.	.	.	.	.	.	.	.	.	.	.	.	.	.	.
3096	1	ATP7B	NM_001243182	chr13	51404805	51483631	-	.	.	.	.	.	.	.	.	.	.	.	.	.	.	.	.	.	.	.	.
2956	1	ALG11	NR_036571	chr13	51484523	51501781	+	.	.	.	.	.	.	.	.	.	.	.	.	.	.	.	.	.	.	.	.
4561	1	NEK5	NM_199289	chr13	51536900	51601215	-	.	.	.	.	.	.	.	.	.	.	.	.	.	.	.	.	.	.	.	.
4559	1	NEK3	NM_001146099	chr13	51604779	51631997	-	.	.	.	.	.	.	.	.	.	.	.	.	.	.	.	.	.	.	.	.
4709	1	PCDH8	NM_002590	chr13	52316109	52320776	-	.	.	.	.	.	.	.	.	.	.	.	.	.	.	.	.	.	.	.	.
7456	0,25	OLFM4	NM_006418	chr13	52500876	52524197	+	.	.	.	.	.	.	.	.	.	.	.	.	.	.	.	.	.	.	.	.
7275	0,25	DIAPH3	NM_030932	chr13	59246297	59485344	-	.	.	.	.	.	.	.	.	.	.	.	.	.	.	.	.	.	.	.	.
7583	0,25	TDRD3	NM_001146071	chr13	59868591	60046014	+	CL799701	chr13	59968604	-	.	.	.	.	.	.	.	.	.	.	.	.	.	.	.	.
6719	0,5	PCDH20	NM_022843	chr13	60881819	60887656	-	.	.	.	.	.	.	.	.	.	.	.	.	.	.	.	.	.	.	.	.
2309	1,5	PCDH9	NM_020403	chr13	65774966	66702469	-	AY516983.1	chr13	65943620	+	.	.	.	.	.	.	.	.	1547_109	chr13	65808459	-	2153_187_1	chr13	66691819	-
2309	1,5	PCDH9	NM_020403	chr13	65774966	66702469	-	AY516983.1	chr13	65943620	+	.	.	.	.	.	.	.	.	1547_109	chr13	65808459	-	2807_251_4	chr13	66110920	-
2309	1,5	PCDH9	NM_020403	chr13	65774966	66702469	-	AY517042.1	chr13	65847942	+	.	.	.	.	.	.	.	.	1547_109	chr13	65808459	-	2153_187_1	chr13	66691819	-
2309	1,5	PCDH9	NM_020403	chr13	65774966	66702469	-	AY517042.1	chr13	65847942	+	.	.	.	.	.	.	.	.	1547_109	chr13	65808459	-	2807_251_4	chr13	66110920	-
4234	1	KLF5	NM_001730	chr13	72531142	72549677	+	.	.	.	.	.	.	.	.	.	.	.	.	.	.	.	.	.	.	.	.
5436	1	TBC1D4	NM_014832	chr13	74756809	74954251	-	.	.	.	.	.	.	.	.	.	.	.	.	.	.	.	.	.	.	.	.
882	2,5	LMO7	NM_015842	chr13	75232797	75332007	+	.	.	.	.	.	.	.	.	.	.	.	.	.	.	.	.	.	.	.	.

Table S2

tumor associated genes								HIV				MLV				MMTV				MMTV(SIN)				MMTV(SIN)arrest			
rank	score	gene symbol	RefSEq	chrom	txStart	txEnd	strand	integrant	chr	position	strand	integrant	chr	position	strand	integrant	chr	position	strand	integrant	chr	position	strand	integrant	chr	position	strand
3749	1	FBXL3	NM_012158	chr13	76477389	76499332	-	.	.	.	.	.	.	.	.	.	.	.	.	.	.	.	.	.	.	.	.
1550	2	MYCBP2	NM_015057	chr13	76516792	76799178	-	AY517017.1	chr13	76679134	-	.	.	.	.	.	.	.	.	.	.	.	.	.	.	.	.
1550	2	MYCBP2	NM_015057	chr13	76516792	76799178	-	AY517098.1	chr13	76678870	-	.	.	.	.	.	.	.	.	.	.	.	.	.	.	.	.
819	2,5	EDNRB	NM_000115	chr13	77367616	77447665	-	.	.	.	.	.	.	.	.	.	.	.	.	.	.	.	.	.	.	.	.
7485	0,25	POU4F1	NM_006237	chr13	78071230	78075696	-	.	.	.	.	.	.	.	.	.	.	.	.	.	.	.	.	.	.	.	.
5142	1	RNF219	NM_024546	chr13	78086421	78131315	-	AY517040.1	chr13	78091571	-	.	.	.	.	.	.	.	.	.	.	.	.	1515_151_1	chr13	78108838	+
5075	1	RBM26	NM_022118	chr13	78792099	78877924	-	.	.	.	.	.	.	.	.	.	.	.	.	.	.	.	.	.	.	.	.
2272	1,5	NDFIP2	NM_001161407	chr13	78953259	79028213	+	CL529610	chr13	78987693	-	.	.	.	.	.	.	.	.	02_1_2491_12	chr13	79014580	+	.	.	.	.
114	5,5	SPRY2	NM_005842	chr13	79808112	79813087	-	.	.	.	.	.	.	.	.	.	.	.	.	.	.	.	.	.	.	.	.
2406	1,5	SLITRK1	NM_052910	chr13	83349343	83354529	-	.	.	.	.	.	.	.	.	.	.	.	.	.	.	.	.	.	.	.	.
5291	1	SLITRK6	NM_032229	chr13	85264922	85271484	-	.	.	.	.	.	.	.	.	.	.	.	.	.	.	.	.	.	.	.	.
6934	0,5	SLITRK5	NM_015567	chr13	87122870	87129871	+	.	.	.	.	.	.	.	.	.	.	.	.	.	.	.	.	.	.	.	.
5914	0,75	GPC5	NM_004466	chr13	90848935	92317488	+	.	.	.	.	.	.	.	.	_214_1_166	chr13	91305043	-	110_1_982_30	chr13	92189291	+	1827_168_1	chr13	92120058	-
5914	0,75	GPC5	NM_004466	chr13	90848935	92317488	+	.	.	.	.	.	.	.	.	_214_1_166	chr13	91305043	-	110_1_982_30	chr13	92189291	+	3176_309_1	chr13	91382905	+
1345	2	DCT	NM_001922	chr13	93889841	93929937	-	.	.	.	.	.	.	.	.	.	.	.	.	.	.	.	.	.	.	.	.
1804	2	TGDS	NM_014305	chr13	94024308	94046512	-	.	.	.	.	.	.	.	.	.	.	.	.	.	.	.	.	.	.	.	.
3954	1	GPR180	NM_180989	chr13	94052104	94084900	+	AY517063.1	chr13	94077555	-	.	.	.	.	.	.	.	.	.	.	.	.	.	.	.	.
3954	1	GPR180	NM_180989	chr13	94052104	94084900	+	BH609696	chr13	94079310	-	.	.	.	.	.	.	.	.	.	.	.	.	.	.	.	.
2783	1,25	SOX21	NM_007084	chr13	94159879	94162390	-	.	.	.	.	.	.	.	.	.	.	.	.	.	.	.	.	.	.	.	.
986	2,25	ABCC4	NM_001105515	chr13	94546025	94751688	-	.	.	.	.	.	.	.	.	.	.	.	.	.	.	.	.	.	.	.	.
3382	1	CLDN10	NM_006984	chr13	95002947	95030011	+	.	.	.	.	.	.	.	.	.	.	.	.	.	.	.	.	.	.	.	.
3637	1	DZIP1	NM_014934	chr13	95028456	95094961	-	.	.	.	.	.	.	.	.	.	.	.	.	.	.	.	.	.	.	.	.
3597	1	DNAJC3	NM_006260	chr13	95127393	95245244	+	.	.	.	.	.	.	.	.	.	.	.	.	.	.	.	.	.	.	.	.
1453	2	HS6ST3	NM_153456	chr13	95541093	96289817	+	.	.	.	.	AY516542	chr13	95691084	+	.	.	.	.	.	.	.	.	2381_206_1	chr13	96146820	-
2247	1,5	MBNL2	NM_144778	chr13	96672550	96844375	+	.	.	.	.	.	.	.	.	_267_1_179	chr13	96760184	-	.	.	.	.	.	.	.	.
665	3	RAP2A	NM_021033	chr13	96884475	96918253	+	.	.	.	.	.	.	.	.	.	.	.	.	.	.	.	.	2258_196_1	chr13	96901174	-
3735	1	FARP1	NM_005766	chr13	97593434	97900024	+	.	.	.	.	.	.	.	.	.	.	.	.	.	.	.	.	.	.	.	.
1783	2	STK24	NM_001032296	chr13	97900455	98027397	-	.	.	.	.	.	.	.	.	.	.	.	.	.	.	.	.	.	.	.	.
3603	1	DOCK9	NM_001130049	chr13	98310435	98536661	-	.	.	.	.	.	.	.	.	.	.	.	.	.	.	.	.	.	.	.	.
5597	1	UBAC2	NM_001144072	chr13	98650679	98836754	+	.	.	.	.	.	.	.	.	.	.	.	.	.	.	.	.	.	.	.	.
6395	0,5	GPR183	NM_004951	chr13	98744789	98757750	-	.	.	.	.	.	.	.	.	.	.	.	.	.	.	.	.	.	.	.	.
5481	1	TM9SF2	NM_004800	chr13	98951728	99013277	+	AY517410.1	chr13	99010335	-	.	.	.	.	.	.	.	.	.	.	.	.	.	.	.	.
1315	2	CLYBL	NM_206808	chr13	99056919	99347389	+	.	.	.	.	.	.	.	.	.	.	.	.	.	.	.	.	.	.	.	.
5749	1	ZIC5	NM_033132	chr13	99413275	99422179	-	.	.	.	.	.	.	.	.	.	.	.	.	.	.	.	.	.	.	.	.
1187	2,25	ZIC2	NM_007129	chr13	99432026	99437020	+	.	.	.	.	.	.	.	.	.	.	.	.	.	.	.	.	.	.	.	.
1600	2	PCCA	NM_000282	chr13	99539269	99980692	+	.	.	.	.	.	.	.	.	_186_1_158	chr13	99820905	+	.	.	.	.	.	.	.	.
5507	1	TMTC4	NM_032813	chr13	100054090	100125104	-	.	.	.	.	.	.	.	.	.	.	.	.	.	.	.	.	.	.	.	.
4492	1	NALCN	NM_052867	chr13	100504130	100866814	-	.	.	.	.	.	.	.	.	.	.	.	.	.	.	.	.	.	.	.	.
4179	1	ITGBL1	NM_004791	chr13	100902966	101166795	+	.	.	.	.	AY516554	chr13	101100719	-	.	.	.	.	.	.	.	.	.	.	.	.
832	2,5	FGF14	NM_175929	chr13	101171205	101852125	-	.	.	.	.	.	.	.	.	.	.	.	.	104_1_718_23	chr13	101450007	+	1782_165_1	chr13	101492414	-
5533	1	TPP2	NM_003291	chr13	102047286	102129524	+	.	.	.	.	.	.	.	.	.	.	.	.	.	.	.	.	.	.	.	.
4205	1	KDELC1	NM_024089	chr13	102234631	102249405	-	.	.	.	.	.	.	.	.	.	.	.	.	.	.	.	.	.	.	.	.
3148	1	BIVM	NM_001159596	chr13	102249399	102291889	+	.	.	.	.	.	.	.	.	.	.	.	.	.	.	.	.	.	.	.	.
77	6,25	ERCC5	NM_000123	chr13	102296191	102326352	+	.	.	.	.	.	.	.	.	.	.	.	.	.	.	.	.	.	.	.	.
5252	1	SLC10A2	NM_000452	chr13	102494348	102517197	-	.	.	.	.	.	.	.	.	.	.	.	.	.	.	.	.	.	.	.	.
6300	0,5	EFNB2	NM_004093	chr13	105940079	105985389	-	.	.	.	.	.	.	.	.	.	.	.	.	.	.	.	.	.	.	.	.
3023	1	ARGLU1	NM_018011	chr13	105993662	106018515	-	.	.	.	.	.	.	.	.	.	.	.	.	.	.	.	.	.	.	.	.
3721	1	FAM155A	NM_001080396	chr13	106618879	107317461	-	.	.	.	.	.	.	.	.	.	.	.	.	.	.	.	.	.	.	.	.
1094	2,25	LIG4	NM_206937	chr13	107657792	107665883	-	CL529365	chr13	107668356	+	.	.	.	.	.	.	.	.	.	.	.	.	.	.	.	.
2860	1	ABHD13	NM_032859	chr13	107668763	107684604	+	.	.	.	.	.	.	.	.	.	.	.	.	.	.	.	.	.	.	.	.
4475	1	MYO16	NM_015011	chr13	108046500	108658356	+	.	.	.	.	.	.	.	.	_391_1_237	chr13	108059021	-	.	.	.	.	.	.	.	.

Table S2

tumor associated genes								HIV				MLV				MMTV				MMTV(SIN)				MMTV(SIN)arrest			
rank	score	gene symbol	RefSEq	chrom	txStart	txEnd	strand	integrant	chr	position	strand	integrant	chr	position	strand	integrant	chr	position	strand	integrant	chr	position	strand	integrant	chr	position	strand
1083	2,25	IRS2	NM_003749	chr13	109204184	109236915	-	CL529449	chr13	109226956	+	.	.	.	.	.	.	.	.	.	.	.	.	.	.	.	.
3415	1	COL4A1	NM_001845	chr13	109599310	109757497	-	.	.	.	.	.	.	.	.	.	.	.	.	.	.	.	.	.	.	.	.
2085	1,5	COL4A2	NM_001846	chr13	109757631	109963374	+	.	.	.	.	AY516579	chr13	109838671	-	.	.	.	.	.	.	.	.	.	.	.	.
3278	1	CARKD	NR_040104	chr13	110065807	110090343	+	.	.	.	.	.	.	.	.	.	.	.	.	.	.	.	.	.	.	.	.
1288	2	CARS2	NM_024537	chr13	110091757	110156481	-	CL800400	chr13	110115944	+	.	.	.	.	.	.	.	.	.	.	.	.	.	.	.	.
732	2,75	ING1	NM_005537	chr13	110165359	110171422	+	.	.	.	.	.	.	.	.	.	.	.	.	.	.	.	.	.	.	.	.
2988	1	ANKRD10	NM_017664	chr13	110328887	110365417	-	.	.	.	.	.	.	.	.	.	.	.	.	.	.	.	.	.	.	.	.
786	2,5	ARHGEF7	NM_003899	chr13	110604061	110756082	+	.	.	.	.	.	.	.	.	.	.	.	.	.	.	.	.	.	.	.	.
2411	1,5	SOX1	NM_005986	chr13	111769913	111774021	+	.	.	.	.	.	.	.	.	.	.	.	.	.	.	.	.	.	.	.	.
5590	1	TUBGCP3	NM_006322	chr13	112187328	112290482	-	.	.	.	.	.	.	.	.	.	.	.	.	.	.	.	.	.	.	.	.
1263	2	ATP11A	NM_032189	chr13	112392643	112589483	+	.	.	.	.	AY516108	chr13	112425997	+	.	.	.	.	.	.	.	.	.	.	.	.
2248	1,5	MCF2L	NM_001112732	chr13	112671535	112802054	+	.	.	.	.	.	.	.	.	.	.	.	.	s1004_163	chr13	112731957	-	.	.	.	.
3710	1	F7	NM_019616	chr13	112808102	112822996	+	.	.	.	.	.	.	.	.	.	.	.	.	.	.	.	.	.	.	.	.
4715	1	PCID2	NM_001127202	chr13	112879853	112911030	-	.	.	.	.	AY516614	chr13	112886297	-	.	.	.	.	.	.	.	.	.	.	.	.
813	2,5	CUL4A	NM_003589	chr13	112911086	112967393	+	.	.	.	.	.	.	.	.	.	.	.	.	.	.	.	.	.	.	.	.
272	4	LAMP1	NM_005561	chr13	112999469	113025742	+	.	.	.	.	.	.	.	.	.	.	.	.	.	.	.	.	.	.	.	.
3975	1	GRTP1	NM_024719	chr13	113026505	113066464	-	.	.	.	.	.	.	.	.	.	.	.	.	.	.	.	.	.	.	.	.
1990	1,5	ADPRHL1	NM_199162	chr13	113124260	113151458	-	.	.	.	.	.	.	.	.	.	.	.	.	.	.	.	.	.	.	.	.
3541	1	DCUN1D2	NM_001014283	chr13	113158134	113193024	-	.	.	.	.	.	.	.	.	.	.	.	.	.	.	.	.	.	.	.	.
5482	1	TMCO3	NM_017905	chr13	113193308	113252545	+	.	.	.	.	.	.	.	.	.	.	.	.	.	.	.	.	.	.	.	.
965	2,5	TFDP1	NM_007111	chr13	113287056	113343789	+	.	.	.	.	.	.	.	.	.	.	.	.	.	.	.	.	.	.	.	.
3070	1	ATP4B	NM_000705	chr13	113351122	113360514	-	.	.	.	.	.	.	.	.	.	.	.	.	.	.	.	.	.	.	.	.
3971	1	GRK1	NM_002929	chr13	113369597	113373973	+	.	.	.	.	.	.	.	.	.	.	.	.	.	.	.	.	.	.	.	.
2151	1,5	GAS6	NM_001143945	chr13	113574925	113590421	+	.	.	.	.	.	.	.	.	.	.	.	.	.	.	.	.	.	.	.	.
1717	2	RASA3	NM_007368	chr13	113765295	113916197	-	.	.	.	.	AY516728	chr13	113847244	-	.	.	.	.	.	.	.	.	.	.	.	.
1297	2	CDC16	NM_003903	chr13	114018463	114056252	+	.	.	.	.	.	.	.	.	.	.	.	.	.	.	.	.	.	.	.	.
4667	1	OR4Q3	NM_172194	chr14	19285426	19286368	+	.	.	.	.	.	.	.	.	.	.	.	.	.	.	.	.	.	.	.	.
5582	1	TTC5	NM_138376	chr14	19827140	19843993	-	CL529401	chr14	19828664	-	.	.	.	.	.	.	.	.	.	.	.	.	.	.	.	.
540	3	CCNB1IP1	NM_182852	chr14	19849366	19871311	-	.	.	.	.	.	.	.	.	.	.	.	.	.	.	.	.	.	.	.	.
288	4	PARP2	NM_005484	chr14	19881612	19895903	+	.	.	.	.	.	.	.	.	.	.	.	.	.	.	.	.	.	.	.	.
5456	1	TEP1	NM_007110	chr14	19903665	19951419	-	.	.	.	.	.	.	.	.	.	.	.	.	.	.	.	.	.	.	.	.
4672	1	OSGEP	NM_017807	chr14	19985046	19993107	-	.	.	.	.	.	.	.	.	.	.	.	.	.	.	.	.	.	.	.	.
314	3,75	APEX1	NM_001244249	chr14	19993129	19995771	+	.	.	.	.	.	.	.	.	.	.	.	.	.	.	.	.	.	.	.	.
5498	1	TMEM55B	NM_144568	chr14	19995851	19999477	-	.	.	.	.	.	.	.	.	.	.	.	.	.	.	.	.	.	.	.	.
1246	2	ANG	NM_001145	chr14	20222175	20232185	+	.	.	.	.	.	.	.	.	.	.	.	.	.	.	.	.	.	.	.	.
2379	1,5	RNASE4	NM_002937	chr14	20222211	20238598	+	.	.	.	.	.	.	.	.	.	.	.	.	.	.	.	.	.	.	.	.
1727	2	RNASE1	NM_002933	chr14	20339354	20340876	-	.	.	.	.	.	.	.	.	.	.	.	.	.	.	.	.	.	.	.	.
2378	1,5	RNASE2	NM_002934	chr14	20493469	20494434	+	.	.	.	.	.	.	.	.	.	.	.	.	.	.	.	.	.	.	.	.
1565	2	NDRG2	NM_016250	chr14	20554761	20563775	-	.	.	.	.	.	.	.	.	.	.	.	.	.	.	.	.	.	.	.	.
5534	1	TPPP2	NM_173846	chr14	20568184	20570172	+	.	.	.	.	.	.	.	.	.	.	.	.	.	.	.	.	.	.	.	.
5769	1	ZNF219	NM_001101672	chr14	20628044	20637013	-	.	.	.	.	.	.	.	.	.	.	.	.	.	.	.	.	.	.	.	.
4066	1	HNRNPC	NM_031314	chr14	20747135	20807478	-	CL529119	chr14	20788002	-	.	.	.	.	.	.	.	.	.	.	.	.	.	.	.	.
5158	1	RPGRIP1	NM_020366	chr14	20825975	20889300	+	.	.	.	.	AY516777	chr14	20846853	-	.	.	.	.	.	.	.	.	.	.	.	.
5412	1	SUPT16H	NM_007192	chr14	20889470	20922265	-	.	.	.	.	.	.	.	.	.	.	.	.	.	.	.	.	.	.	.	.
543	3	CHD8	NM_020920	chr14	20923192	20975297	-	.	.	.	.	.	.	.	.	.	.	.	.	.	.	.	.	.	.	.	.
5027	1	RAB2B	NM_032846	chr14	20997018	21014972	-	.	.	.	.	.	.	.	.	.	.	.	.	.	.	.	.	.	.	.	.
5524	1	TOX4	NM_014828	chr14	21015174	21037159	+	.	.	.	.	.	.	.	.	.	.	.	.	.	.	.	.	.	.	.	.
4384	1	METTL3	NM_019852	chr14	21036121	21049297	-	.	.	.	.	.	.	.	.	.	.	.	.	.	.	.	.	.	.	.	.
5177	1	SALL2	NM_005407	chr14	21059071	21075177	-	.	.	.	.	.	.	.	.	.	.	.	.	.	.	.	.	.	.	.	.
3524	1	DAD1	NM_001344	chr14	22103646	22127983	-	.	.	.	.	.	.	.	.	.	.	.	.	.	.	.	.	.	.	.	.
2678	1,25	MMP14	NM_004995	chr14	22375632	22386643	+	.	.	.	.	.	.	.	.	.	.	.	.	.	.	.	.	.	.	.	.

Table S2

tumor associated genes								HIV				MLV				MMTV				MMTV(SIN)				MMTV(SIN)arrest			
rank	score	gene symbol	RefSEq	chrom	txStart	txEnd	strand	integrant	chr	position	strand	integrant	chr	position	strand	integrant	chr	position	strand	integrant	chr	position	strand	integrant	chr	position	strand
6573	0,5	LRP10	NM_014045	chr14	22410799	22417131	+	.	.	.	.	.	.	.	.	.	.	.	.	.	.	.	.	.	.	.	.
1690	2	PRMT5	NM_006109	chr14	22459572	22468501	-	.	.	.	.	.	.	.	.	.	.	.	.	.	.	.	.	.	.	.	.
6167	0,5	CEBPE	NM_001805	chr14	22656354	22658660	-	.	.	.	.	.	.	.	.	.	.	.	.	.	.	.	.	.	.	.	.
1276	2	BCL2L2	NM_001199839	chr14	22845908	22850808	+	.	.	.	.	.	.	.	.	.	.	.	.	.	.	.	.	.	.	.	.
3401	1	CMTM5	NM_138460	chr14	22915856	22918821	+	.	.	.	.	.	.	.	.	.	.	.	.	.	.	.	.	.	.	.	.
6637	0,5	MYH7	NM_000257	chr14	22951786	22974710	-	.	.	.	.	.	.	.	.	.	.	.	.	.	.	.	.	.	.	.	.
2437	1,5	THTPA	NR_046052	chr14	23051858	23098630	+	.	.	.	.	.	.	.	.	.	.	.	.	.	.	.	.	.	.	.	.
3576	1	DHRS2	NM_005794	chr14	23175412	23184688	+	.	.	.	.	.	.	.	.	.	.	.	.	.	.	.	.	.	.	.	.
2091	1,5	CPNE6	NM_006032	chr14	23610595	23617136	+	.	.	.	.	.	.	.	.	.	.	.	.	.	.	.	.	.	.	.	.
4717	1	PCK2	NM_004563	chr14	23633322	23643179	+	.	.	.	.	.	.	.	.	.	.	.	.	.	.	.	.	.	.	.	.
5089	1	REC8	NM_005132	chr14	23711073	23719303	+	.	.	.	.	.	.	.	.	.	.	.	.	.	.	.	.	.	.	.	.
4157	1	IPO4	NR_051979	chr14	23719264	23727964	-	.	.	.	.	.	.	.	.	.	.	.	.	.	.	.	.	.	.	.	.
5577	1	TSSK4	NM_174944	chr14	23744765	23747294	+	.	.	.	.	.	.	.	.	.	.	.	.	.	.	.	.	.	.	.	.
3913	1	GMPR2	NM_001002000	chr14	23771487	23778287	+	.	.	.	.	.	.	.	.	.	.	.	.	.	.	.	.	.	.	.	.
7585	0,25	TGM1	NM_000359	chr14	23788159	23802256	-	.	.	.	.	.	.	.	.	.	.	.	.	.	.	.	.	.	.	.	.
3575	1	DHRS1	NM_138452	chr14	23829643	23838506	-	.	.	.	.	.	.	.	.	.	.	.	.	.	.	.	.	.	.	.	.
4314	1	LTB4R	NM_181657	chr14	23852152	23857082	+	.	.	.	.	.	.	.	.	.	.	.	.	.	.	.	.	.	.	.	.
1218	2	ADCY4	NM_001198568	chr14	23857394	23874117	-	.	.	.	.	.	.	.	.	.	.	.	.	.	.	.	.	.	.	.	.
2377	1,5	RIPK3	NM_006871	chr14	23875066	23879082	-	.	.	.	.	.	.	.	.	.	.	.	.	.	.	.	.	.	.	.	.
4573	1	NFATC4	NM_001136022	chr14	23905984	23918650	+	.	.	.	.	.	.	.	.	.	.	.	.	.	.	.	.	.	.	.	.
7339	0,25	GZMB	NM_004131	chr14	24170001	24173272	-	.	.	.	.	.	.	.	.	.	.	.	.	.	.	.	.	.	.	.	.
6987	0,5	STXBP6	NM_014178	chr14	24351143	24588935	-	.	.	.	.	.	.	.	.	.	.	.	.	.	.	.	.	.	.	.	.
1144	2,25	PRKD1	NM_002742	chr14	29115437	29466650	-	.	.	.	.	.	.	.	.	.	.	.	.	.	.	.	.	.	.	.	.
3411	1	COCH	NM_001135058	chr14	30413491	30429573	+	.	.	.	.	.	.	.	.	.	.	.	.	.	.	.	.	.	.	.	.
7576	0,25	STRN3	NM_001083893	chr14	30432755	30565358	-	.	.	.	.	.	.	.	.	.	.	.	.	.	.	.	.	.	.	.	.
3026	1	ARHGAP5	NM_001173	chr14	31616245	31698685	+	.	.	.	.	.	.	.	.	.	.	.	.	.	.	.	.	.	.	.	.
356	3,5	AKAP6	NM_004274	chr14	31868229	32372019	+	.	.	.	.	.	.	.	.	.	.	.	.	.	.	.	.	.	.	.	.
6679	0,5	NPAS3	NM_022123	chr14	32478209	33343133	+	.	.	.	.	.	.	.	.	.	.	.	.	.	.	.	.	.	.	.	.
3660	1	EGLN3	NM_022073	chr14	33463171	33490035	-	.	.	.	.	.	.	.	.	.	.	.	.	.	.	.	.	.	.	.	.
6951	0,5	SNX6	NM_152233	chr14	34100368	34169117	-	.	.	.	.	.	.	.	.	.	.	.	.	.	.	.	.	.	.	.	.
3129	1	BAZ1A	NM_182648	chr14	34291687	34414604	-	CL800035	chr14	34322903	+	.	.	.	.	.	.	.	.	.	.	.	.	.	.	.	.
4922	1	PPP2R3C	NM_017917	chr14	34624428	34661270	-	.	.	.	.	.	.	.	.	.	.	.	.	.	.	.	.	.	.	.	.
629	3	NFKBIA	NM_020529	chr14	34940466	34943711	-	.	.	.	.	.	.	.	.	.	.	.	.	.	.	.	.	.	.	.	.
487	3,25	NKX2-1	NM_001079668	chr14	36055352	36059181	-	.	.	.	.	.	.	.	.	.	.	.	.	.	.	.	.	.	.	.	.
5263	1	SLC25A21	NM_001171170	chr14	36216876	36711616	-	.	.	.	.	.	.	.	.	.	.	.	.	.	.	.	.	.	.	.	.
7317	0,25	FOXA1	NM_004496	chr14	37128507	37134076	-	.	.	.	.	.	.	.	.	.	.	.	.	.	.	.	.	.	.	.	.
2418	1,5	SSTR1	NM_001049	chr14	37746954	37752019	+	.	.	.	.	.	.	.	.	.	.	.	.	.	.	.	.	.	.	.	.
6572	0,5	LRFN5	NM_152447	chr14	41146513	41443504	+	.	.	.	.	.	.	.	.	.	.	.	.	.	.	.	.	.	.	.	.
3807	1	FSCB	NM_032135	chr14	44043101	44046249	-	.	.	.	.	.	.	.	.	.	.	.	.	.	.	.	.	.	.	.	.
1395	2	FANCM	NM_020937	chr14	44674885	44739843	+	.	.	.	.	.	.	.	.	.	.	.	.	.	.	.	.	.	.	.	.
4391	1	MGAT2	NM_002408	chr14	49157238	49159949	+	.	.	.	.	.	.	.	.	.	.	.	.	.	.	.	.	.	.	.	.
1644	2	POLE2	NM_002692	chr14	49180019	49224848	-	.	.	.	.	.	.	.	.	.	.	.	.	.	.	.	.	.	.	.	.
3019	1	ARF6	NM_001663	chr14	49429485	49433522	+	.	.	.	.	.	.	.	.	.	.	.	.	.	.	.	.	.	.	.	.
6956	0,5	SOS2	NM_006939	chr14	49653595	49767849	-	CL529629	chr14	49670734	+	.	.	.	.	.	.	.	.	.	.	.	.	.	.	.	.
4252	1	L2HGDH	NM_024884	chr14	49778901	49848697	-	.	.	.	.	.	.	.	.	.	.	.	.	.	.	.	.	.	.	.	.
3317	1	CDKL1	NM_004196	chr14	49866469	49932367	-	.	.	.	.	.	.	.	.	.	.	.	.	.	.	.	.	.	.	.	.
4352	1	MAP4K5	NM_198794	chr14	49954960	50069126	-	.	.	.	.	.	.	.	.	.	.	.	.	.	.	.	.	.	.	.	.
6038	0,5	ATL1	NM_015915	chr14	50096492	50169534	+	.	.	.	.	.	.	.	.	.	.	.	.	.	.	.	.	.	.	.	.
5185	1	SAV1	NM_021818	chr14	50170047	50204821	-	.	.	.	.	.	.	.	.	.	.	.	.	.	.	.	.	.	.	.	.
1576	2	NIN	NM_016350	chr14	50262296	50367589	-	.	.	.	.	.	.	.	.	.	.	.	.	.	.	.	.	.	.	.	.
2353	1,5	PYGL	NM_001163940	chr14	50441684	50480998	-	.	.	.	.	.	.	.	.	.	.	.	.	.	.	.	.	.	.	.	.

Table S2

tumor associated genes								HIV				MLV				MMTV				MMTV(SIN)				MMTV(SIN)arrest			
rank	score	gene symbol	RefSEq	chrom	txStart	txEnd	strand	integrant	chr	position	strand	integrant	chr	position	strand	integrant	chr	position	strand	integrant	chr	position	strand	integrant	chr	position	strand
3804	1	FRMD6	NM_001267046	chr14	51188325	51267194	+	.	.	.	.	.	.	.	.	.	.	.	.	.	.	.	.	.	.	.	.
6671	0,5	NID2	NM_007361	chr14	51541269	51605696	-	.	.	.	.	.	.	.	.	.	.	.	.	.	.	.	.	.	.	.	.
3932	1	GNPNAT1	NM_198066	chr14	52311660	52328136	-	.	.	.	.	.	.	.	.	.	.	.	.	.	.	.	.	.	.	.	.
3544	1	DDHD1	NM_001160147	chr14	52573207	52689796	-	.	.	.	.	.	.	.	.	.	.	.	.	.	.	.	.	.	.	.	.
317	3,75	BMP4	NM_130851	chr14	53486204	53491020	-	.	.	.	.	.	.	.	.	.	.	.	.	.	.	.	.	.	.	.	.
2543	1,25	CDKN3	NM_001130851	chr14	53933422	53956684	+	.	.	.	.	.	.	.	.	.	.	.	.	.	.	.	.	.	.	.	.
3868	1	GCH1	NM_001024024	chr14	54378473	54439292	-	CL800605	chr14	54419373	+	.	.	.	.	.	.	.	.	.	.	.	.	.	.	.	.
7093	0,5	WDHD1	NM_007086	chr14	54475405	54563569	-	CL800848	chr14	54478399	+	.	.	.	.	.	.	.	.	.	.	.	.	.	.	.	.
4355	1	MAPK1IP1L	NM_144578	chr14	54588114	54606665	+	.	.	.	.	.	.	.	.	.	.	.	.	.	.	.	.	.	.	.	.
394	3,5	LGALS3	NR_003225	chr14	54673453	54681901	+	.	.	.	.	.	.	.	.	.	.	.	.	.	.	.	.	.	.	.	.
3757	1	FBXO34	NM_017943	chr14	54807773	54890082	+	.	.	.	.	.	.	.	.	.	.	.	.	.	.	.	.	.	.	.	.
877	2,5	KTN1	NM_001079522	chr14	55116677	55221055	+	BH609700	chr14	55122527	-	.	.	.	.	.	.	.	.	.	.	.	.	.	.	.	.
4221	1	KIAA0586	NM_001244190	chr14	57963855	58085302	+	.	.	.	.	.	.	.	.	.	.	.	.	.	.	.	.	.	.	.	.
3523	1	DACT1	NR_046093	chr14	58170538	58184791	+	.	.	.	.	.	.	.	.	_230_1_171	chr14	58172170	+	.	.	.	.	.	.	.	.
3521	1	DAAM1	NM_001270520	chr14	58725133	58907876	+	.	.	.	.	.	.	.	.	.	.	.	.	.	.	.	.	.	.	.	.
3948	1	GPR135	NM_022571	chr14	58999992	59001812	-	.	.	.	.	.	.	.	.	.	.	.	.	.	.	.	.	.	.	.	.
3181	1	C14orf149	NM_144581	chr14	59009158	59020826	-	.	.	.	.	.	.	.	.	.	.	.	.	.	.	.	.	.	.	.	.
2758	1,25	RTN1	NM_206852	chr14	59132446	59167285	-	.	.	.	.	.	.	.	.	.	.	.	.	.	.	.	.	.	.	.	.
1356	2	DHRS7	NM_016029	chr14	59681251	59701964	-	.	.	.	.	.	.	.	.	.	.	.	.	.	.	.	.	.	.	.	.
1666	2	PPM1A	NM_177951	chr14	59785718	59825025	+	CL800614	chr14	59793574	+	.	.	.	.	.	.	.	.	.	.	.	.	.	.	.	.
5247	1	SIX1	NM_005982	chr14	60181169	60185908	-	.	.	.	.	.	.	.	.	.	.	.	.	.	.	.	.	.	.	.	.
1760	2	SIX4	NM_017420	chr14	60246008	60260605	-	.	.	.	.	.	.	.	.	.	.	.	.	.	.	.	.	.	.	.	.
898	2,5	MNAT1	NM_002431	chr14	60271211	60505151	+	CL529125	chr14	60346597	-	.	.	.	.	.	.	.	.	.	.	.	.	.	.	.	.
898	2,5	MNAT1	NM_002431	chr14	60271211	60505151	+	CL799917	chr14	60343573	-	.	.	.	.	.	.	.	.	.	.	.	.	.	.	.	.
5561	1	TRMT5	NM_020810	chr14	60507919	60517535	-	.	.	.	.	.	.	.	.	.	.	.	.	.	.	.	.	.	.	.	.
5275	1	SLC38A6	NM_001172702	chr14	60517584	60620204	+	.	.	.	.	.	.	.	.	.	.	.	.	.	.	.	.	.	.	.	.
1142	2,25	PRKCH	NM_006255	chr14	60858267	61087451	+	.	.	.	.	.	.	.	.	.	.	.	.	.	.	.	.	.	.	.	.
107	5,5	HIF1A	NM_181054	chr14	61231871	61284730	+	.	.	.	.	.	.	.	.	.	.	.	.	.	.	.	.	.	.	.	.
2409	1,5	SNAPC1	NM_003082	chr14	61298827	61332899	+	.	.	.	.	.	.	.	.	.	.	.	.	.	.	.	.	.	.	.	.
1725	2	RHOJ	NM_020663	chr14	62740854	62829983	+	.	.	.	.	.	.	.	.	.	.	.	.	.	.	.	.	.	.	.	.
1673	2	PPP2R5E	NM_006246	chr14	62911107	63079832	-	CL800173	chr14	63031487	+	.	.	.	.	.	.	.	.	.	.	.	.	.	.	.	.
5697	1	WDR89	NM_080666	chr14	63133509	63178394	-	.	.	.	.	.	.	.	.	.	.	.	.	.	.	.	.	.	.	.	.
680	3	SGPP1	NM_030791	chr14	63220687	63264509	-	.	.	.	.	.	.	.	.	.	.	.	.	.	.	.	.	.	.	.	.
5420	1	SYNE2	NM_182910	chr14	63752424	63762922	+	.	.	.	.	.	.	.	.	.	.	.	.	.	.	.	.	.	.	.	.
155	4,75	ESR2	NM_001437	chr14	63769499	63830881	-	.	.	.	.	.	.	.	.	.	.	.	.	.	.	.	.	.	.	.	.
1547	2	MTHFD1	NM_005956	chr14	63924511	63996478	+	.	.	.	.	.	.	.	.	.	.	.	.	.	.	.	.	.	.	.	.
2942	1	AKAP5	NM_004857	chr14	64001969	64010974	+	.	.	.	.	.	.	.	.	.	.	.	.	.	.	.	.	.	.	.	.
5731	1	ZBTB25	NM_006977	chr14	64023307	64040307	-	.	.	.	.	.	.	.	.	.	.	.	.	.	.	.	.	.	.	.	.
5730	1	ZBTB1	NM_014950	chr14	64041044	64070161	+	.	.	.	.	.	.	.	.	.	.	.	.	.	.	.	.	.	.	.	.
858	2,5	HSPA2	NM_021979	chr14	64076938	64079707	+	.	.	.	.	.	.	.	.	.	.	.	.	.	.	.	.	.	.	.	.
4840	1	PLEKHG3	NM_015549	chr14	64240945	64280813	+	.	.	.	.	.	.	.	.	.	.	.	.	.	.	.	.	.	.	.	.
2417	1,5	SPTB	NM_001024858	chr14	64282753	64359619	-	.	.	.	.	.	.	.	.	.	.	.	.	.	.	.	.	.	.	.	.
3368	1	CHURC1	NM_001204064	chr14	64450831	64471837	+	.	.	.	.	.	.	.	.	.	.	.	.	.	.	.	.	.	.	.	.
3959	1	GPX2	NR_046320	chr14	64475622	64479376	-	.	.	.	.	.	.	.	.	.	.	.	.	.	.	.	.	.	.	.	.
1708	2	RAB15	NM_198686	chr14	64482284	64508628	-	.	.	.	.	.	.	.	.	.	.	.	.	.	.	.	.	.	.	.	.
3793	1	FNTB	NM_002028	chr14	64523259	64599123	+	.	.	.	.	.	.	.	.	.	.	.	.	.	.	.	.	.	.	.	.
1526	2	MAX	NM_145113	chr14	64611597	64638980	-	.	.	.	.	.	.	.	.	.	.	.	.	.	.	.	.	.	.	.	.
1413	2	FUT8	NM_178155	chr14	64947062	65280592	+	AY517382.1	chr14	65031598	+	.	.	.	.	.	.	.	.	.	.	.	.	.	.	.	.
582	3	GPHN	NM_001024218	chr14	66043877	66718278	+	CL800469	chr14	66557314	-	.	.	.	.	.	.	.	.	.	.	.	.	.	.	.	.
582	3	GPHN	NM_001024218	chr14	66043877	66718278	+	CL800085	chr14	66278629	+	.	.	.	.	.	.	.	.	.	.	.	.	.	.	.	.
582	3	GPHN	NM_001024218	chr14	66043877	66718278	+	BH609874	chr14	66597256	-	.	.	.	.	.	.	.	.	.	.	.	.	.	.	.	.

Table S2

tumor associated genes								HIV				MLV				MMTV				MMTV(SIN)				MMTV(SIN)arrest			
rank	score	gene symbol	RefSEq	chrom	txStart	txEnd	strand	integrant	chr	position	strand	integrant	chr	position	strand	integrant	chr	position	strand	integrant	chr	position	strand	integrant	chr	position	strand
582	3	GPHN	NM_001024218	chr14	66043877	66718278	+	CL529127	chr14	66624445	-	.	.	.	.	.	.	.	.	.	.	.	.	.	.	.	.
582	3	GPHN	NM_001024218	chr14	66043877	66718278	+	CL529126	chr14	66164917	+	.	.	.	.	.	.	.	.	.	.	.	.	.	.	.	.
3731	1	FAM71D	NM_173526	chr14	66725862	66765020	+	.	.	.	.	.	.	.	.	.	.	.	.	.	.	.	.	.	.	.	.
4431	1	MPP5	NM_022474	chr14	66777764	66872531	+	.	.	.	.	.	.	.	.	.	.	.	.	s388_111	chr14	66829584	+	.	.	.	.
1268	2	ATP6V1D	NM_015994	chr14	66874333	66896473	-	.	.	.	.	.	.	.	.	.	.	.	.	.	.	.	.	.	.	.	.
1039	2,25	EIF2S1	NM_004094	chr14	66896786	66922986	+	.	.	.	.	.	.	.	.	.	.	.	.	.	.	.	.	.	.	.	.
4838	1	PLEK2	NM_016445	chr14	66923452	66948581	-	.	.	.	.	.	.	.	.	.	.	.	.	.	.	.	.	.	.	.	.
1619	2	PIGH	NM_004569	chr14	67125775	67136770	-	.	.	.	.	.	.	.	.	.	.	.	.	.	.	.	.	.	.	.	.
1252	2	ARG2	NM_001172	chr14	67156331	67188189	+	.	.	.	.	.	.	.	.	.	.	.	.	.	.	.	.	.	.	.	.
5682	1	VTI1B	NM_006370	chr14	67187618	67211355	-	.	.	.	.	.	.	.	.	.	.	.	.	.	.	.	.	.	.	.	.
1721	2	RDH11	NM_001252650	chr14	67213270	67232263	-	.	.	.	.	.	.	.	.	.	.	.	.	.	.	.	.	.	.	.	.
5084	1	RDH12	NM_152443	chr14	67238355	67270921	+	.	.	.	.	.	.	.	.	.	.	.	.	.	.	.	.	.	.	.	.
1868	2	ZFYVE26	NM_015346	chr14	67282989	67353059	-	.	.	.	.	.	.	.	.	.	.	.	.	.	.	.	.	.	.	.	.
5744	1	ZFP36L1	NM_001244701	chr14	68324124	68332713	-	.	.	.	.	.	.	.	.	.	.	.	.	.	.	.	.	.	.	.	.
2488	1,25	ACTN1	NM_001102	chr14	68410592	68515836	-	.	.	.	.	.	.	.	.	.	.	.	.	.	.	.	.	.	.	.	.
3703	1	EXD2	NR_034164	chr14	68727946	68780490	+	.	.	.	.	.	.	.	.	.	.	.	.	.	.	.	.	.	.	.	.
3850	1	GALNTL1	NM_001168368	chr14	68796433	68890943	+	.	.	.	.	.	.	.	.	.	.	.	.	.	.	.	.	.	.	.	.
2127	1,5	ERH	NM_004450	chr14	68916592	68934774	-	.	.	.	.	.	.	.	.	.	.	.	.	.	.	.	.	.	.	.	.
5278	1	SLC39A9	NM_001252151	chr14	68934848	68998860	+	.	.	.	.	.	.	.	.	.	.	.	.	.	.	.	.	.	.	.	.
6079	0,5	C14orf162	NR_024630	chr14	69106283	69107671	-	.	.	.	.	.	.	.	.	.	.	.	.	.	.	.	.	.	.	.	.
4215	1	KIAA0247	NM_014734	chr14	69148062	69251614	+	CL800108	chr14	69237824	+	.	.	.	.	.	.	.	.	.	.	.	.	.	.	.	.
1766	2	SMOC1	NM_001034852	chr14	69415866	69568836	+	.	.	.	.	.	.	.	.	.	.	.	.	177_317	chr14	69422606	-	.	.	.	.
2405	1,5	SLC8A3	NM_058240	chr14	69580686	69725540	-	.	.	.	.	.	.	.	.	.	.	.	.	.	.	.	.	.	.	.	.
3431	1	COX16	NM_001204090	chr14	69861550	69896201	-	.	.	.	.	.	.	.	.	.	.	.	.	.	.	.	.	.	.	.	.
5421	1	SYNJ2BP	NM_018373	chr14	69902965	69953560	-	.	.	.	.	.	.	.	.	.	.	.	.	.	.	.	.	.	.	.	.
2900	1	ADAM21	NM_003813	chr14	69988626	69996375	+	.	.	.	.	.	.	.	.	.	.	.	.	.	.	.	.	.	.	.	.
2899	1	ADAM20	NM_003814	chr14	70058830	70071485	-	.	.	.	.	.	.	.	.	.	.	.	.	.	.	.	.	.	.	.	.
4377	1	MED6	NM_005466	chr14	70120709	70137137	-	.	.	.	.	.	.	.	.	.	.	.	.	.	.	.	.	.	.	.	.
5583	1	TTC9	NM_015351	chr14	70178256	70211830	+	.	.	.	.	.	.	.	.	.	.	.	.	.	.	.	.	.	.	.	.
4350	1	MAP3K9	NM_033141	chr14	70264606	70345641	-	.	.	.	.	.	.	.	.	.	.	.	.	199_260	chr14	70326558	-	.	.	.	.
4718	1	PCNX	NM_014982	chr14	70443874	70651852	+	CL799918	chr14	70563259	-	.	.	.	.	.	.	.	.	.	.	.	.	.	.	.	.
2401	1,5	SIPA1L1	NM_015556	chr14	71065794	71275873	+	.	.	.	.	AY516672	chr14	71069840	+	.	.	.	.	.	.	.	.	.	.	.	.
1157	2,25	RGS6	NM_001204423	chr14	71468569	72102991	+	.	.	.	.	.	.	.	.	.	.	.	.	.	.	.	.	.	.	.	.
3605	1	DPF3	NM_012074	chr14	72206412	72430562	-	.	.	.	.	.	.	.	.	.	.	.	.	.	.	.	.	.	.	.	.
5746	1	ZFYVE1	NM_021260	chr14	72505911	72563673	-	CL529128	chr14	72536730	-	.	.	.	.	.	.	.	.	.	.	.	.	.	.	.	.
5074	1	RBM25	NM_021239	chr14	72594973	72657829	+	.	.	.	.	.	.	.	.	.	.	.	.	.	.	.	.	.	.	.	.
657	3	PSEN1	NM_007318	chr14	72672895	72760152	+	.	.	.	.	.	.	.	.	.	.	.	.	.	.	.	.	.	.	.	.
2305	1,5	PAPLN	NM_173462	chr14	72773957	72811100	+	.	.	.	.	.	.	.	.	.	.	.	.	.	.	.	.	.	.	.	.
1280	2	C14orf169	NM_024644	chr14	73027396	73029858	+	.	.	.	.	.	.	.	.	.	.	.	.	.	.	.	.	.	.	.	.
2884	1	ACOT6	NM_001037162	chr14	73153300	73156345	+	.	.	.	.	.	.	.	.	.	.	.	.	.	.	.	.	.	.	.	.
3598	1	DNAL1	NM_001201366	chr14	73181330	73240184	+	CL800265	chr14	73191947	-	.	.	.	.	.	.	.	.	.	.	.	.	.	.	.	.
3598	1	DNAL1	NM_001201366	chr14	73181330	73240184	+	CL800784	chr14	73216637	-	.	.	.	.	.	.	.	.	.	.	.	.	.	.	.	.
4859	1	PNMA1	NM_006029	chr14	73248238	73250881	-	.	.	.	.	.	.	.	.	.	.	.	.	.	.	.	.	.	.	.	.
3188	1	C14orf43	NM_194278	chr14	73251577	73296754	-	CL799995	chr14	73284271	-	.	.	.	.	.	.	.	.	.	.	.	.	.	.	.	.
4985	1	PTGR2	NM_001146154	chr14	73388286	73421921	+	.	.	.	.	.	.	.	.	.	.	.	.	.	.	.	.	.	.	.	.
5795	1	ZNF410	NR_040251	chr14	73423070	73468744	+	.	.	.	.	.	.	.	.	.	.	.	.	.	.	.	.	.	.	.	.
3723	1	FAM161B	NM_152445	chr14	73469447	73486870	-	.	.	.	.	.	.	.	.	.	.	.	.	.	.	.	.	.	.	.	.
1321	2	COQ6	NM_182480	chr14	73486389	73499566	+	.	.	.	.	.	.	.	.	.	.	.	.	.	.	.	.	.	.	.	.
1384	2	ENTPD5	NM_001249	chr14	73502933	73555779	-	BH609704	chr14	73540494	-	.	.	.	.	.	.	.	.	.	.	.	.	.	.	.	.
3189	1	C14orf45	NM_025057	chr14	73555811	73602548	+	.	.	.	.	.	.	.	.	.	.	.	.	.	.	.	.	.	.	.	.
1238	2	ALDH6A1	NM_005589	chr14	73596624	73620949	-	.	.	.	.	.	.	.	.	.	.	.	.	.	.	.	.	.	.	.	.

Table S2

tumor associated genes								HIV				MLV				MMTV				MMTV(SIN)				MMTV(SIN)arrest			
rank	score	gene symbol	RefSEq	chrom	txStart	txEnd	strand	integrant	chr	position	strand	integrant	chr	position	strand	integrant	chr	position	strand	integrant	chr	position	strand	integrant	chr	position	strand
4286	1	LIN52	NM_001024674	chr14	73621408	73736870	+	CL799762	chr14	73652838	-	.	.	.	.	.	.	.	.	.	.	.	.	.	.	.	.
517	3	ABCD4	NM_005050	chr14	73821732	73839520	-	.	.	.	.	.	.	.	.	.	.	.	.	.	.	.	.	.	.	.	.
2284	1,5	NPC2	NM_006432	chr14	74016395	74029837	-	.	.	.	.	.	.	.	.	.	.	.	.	.	.	.	.	.	.	.	.
4170	1	ISCA2	NM_194279	chr14	74030202	74032024	+	.	.	.	.	.	.	.	.	.	.	.	.	.	.	.	.	.	.	.	.
6581	0,5	LTBP2	NM_000428	chr14	74034638	74148787	-	.	.	.	.	.	.	.	.	.	.	.	.	1580_289	chr14	74059277	-	.	.	.	.
4216	1	KIAA0317	NM_001039479	chr14	74197707	74249560	-	.	.	.	.	.	.	.	.	.	.	.	.	.	.	.	.	.	.	.	.
3763	1	FCF1	NM_015962	chr14	74249602	74273143	+	.	.	.	.	.	.	.	.	.	.	.	.	.	.	.	.	.	.	.	.
5724	1	YLPM1	NM_019589	chr14	74299821	74373766	+	.	.	.	.	.	.	.	.	.	.	.	.	.	.	.	.	.	.	.	.
4952	1	PROX2	NM_001243007	chr14	74389488	74400290	-	.	.	.	.	.	.	.	.	.	.	.	.	.	.	.	.	.	.	.	.
1361	2	DLST	NM_001244883	chr14	74418346	74428347	+	.	.	.	.	.	.	.	.	.	.	.	.	.	.	.	.	.	.	.	.
1129	2,25	PGF	NM_001207012	chr14	74478285	74492220	-	.	.	.	.	.	.	.	.	.	.	.	.	.	.	.	.	.	.	.	.
3665	1	EIF2B2	NM_014239	chr14	74539364	74546047	+	.	.	.	.	.	.	.	.	.	.	.	.	.	.	.	.	.	.	.	.
622	3	MLH3	NM_001040108	chr14	74550219	74587988	-	.	.	.	.	.	.	.	.	.	.	.	.	.	.	.	.	.	.	.	.
1207	2	ACYP1	NM_203488	chr14	74589680	74600489	-	AY517336.1	chr14	74596609	-	.	.	.	.	.	.	.	.	.	.	.	.	.	.	.	.
1574	2	NEK9	NM_033116	chr14	74618570	74663531	-	.	.	.	.	.	.	.	.	.	.	.	.	.	.	.	.	.	.	.	.
5483	1	TMED10	NM_006827	chr14	74667923	74713102	-	.	.	.	.	.	.	.	.	.	.	.	.	.	.	.	.	.	.	.	.
158	4,75	FOS	NM_005252	chr14	74815233	74818690	+	.	.	.	.	.	.	.	.	.	.	.	.	.	.	.	.	.	.	.	.
6053	0,5	BATF	NM_006399	chr14	75058536	75083087	+	.	.	.	.	.	.	.	.	.	.	.	.	.	.	.	.	.	.	.	.
3174	1	C14orf1	NM_007176	chr14	75186985	75197291	-	.	.	.	.	.	.	.	.	.	.	.	.	.	.	.	.	.	.	.	.
5586	1	TTLL5	NM_015072	chr14	75197303	75491178	+	CL529368	chr14	75435220	+	.	.	.	.	.	.	.	.	.	.	.	.	.	.	.	.
348	3,75	TGFB3	NM_003239	chr14	75494194	75517845	-	.	.	.	.	.	.	.	.	.	.	.	.	.	.	.	.	.	.	.	.
3176	1	C14orf118	NM_017972	chr14	75688011	75738887	+	.	.	.	.	.	.	.	.	.	.	.	.	.	.	.	.	.	.	.	.
3699	1	ESRRB	NM_004452	chr14	75907442	76037933	+	.	.	.	.	.	.	.	.	.	.	.	.	.	.	.	.	.	.	.	.
5670	1	VASH1	NM_014909	chr14	76297987	76319116	+	.	.	.	.	.	.	.	.	.	.	.	.	.	.	.	.	.	.	.	.
2981	1	ANGEL1	NM_015305	chr14	76323338	76349036	-	.	.	.	.	.	.	.	.	.	.	.	.	.	.	.	.	.	.	.	.
4227	1	KIAA1737	NM_033426	chr14	76634330	76653383	+	.	.	.	.	.	.	.	.	.	.	.	.	.	.	.	.	.	.	.	.
5738	1	ZDHHC22	NM_174976	chr14	76667365	76677887	-	.	.	.	.	.	.	.	.	.	.	.	.	.	.	.	.	.	.	.	.
6669	0,5	NGB	NM_021257	chr14	76801586	76807408	-	.	.	.	.	.	.	.	.	.	.	.	.	.	.	.	.	.	.	.	.
2330	1,5	POMT2	NM_013382	chr14	76811051	76856978	-	.	.	.	.	.	.	.	.	.	.	.	.	.	.	.	.	.	.	.	.
1435	2	GSTZ1	NM_001513	chr14	76857458	76867693	+	.	.	.	.	.	.	.	.	.	.	.	.	.	.	.	.	.	.	.	.
5484	1	TMED8	NM_213601	chr14	76877866	76913149	-	.	.	.	.	.	.	.	.	.	.	.	.	.	.	.	.	.	.	.	.
2934	1	AHSA1	NM_012111	chr14	76994125	77005568	+	.	.	.	.	.	.	.	.	.	.	.	.	.	.	.	.	.	.	.	.
5345	1	SPTLC2	NM_004863	chr14	77042092	77152863	-	.	.	.	.	.	.	.	.	.	.	.	.	.	.	.	.	.	.	.	.
2965	1	ALKBH1	NM_006020	chr14	77208501	77244109	-	.	.	.	.	.	.	.	.	.	.	.	.	.	.	.	.	.	.	.	.
429	3,5	SNW1	NM_012245	chr14	77253696	77297250	-	.	.	.	.	.	.	.	.	.	.	.	.	.	.	.	.	.	.	.	.
3184	1	C14orf178	NM_001173978	chr14	77296925	77305838	+	.	.	.	.	.	.	.	.	.	.	.	.	.	.	.	.	.	.	.	.
1213	2	ADCK1	NM_020421	chr14	77336178	77470050	+	.	.	.	.	.	.	.	.	.	.	.	.	.	.	.	.	.	.	.	.
4626	1	NRXN3	NM_138970	chr14	78815406	79400513	+	CL529129	chr14	79049765	+	.	.	.	.	.	.	.	.	105_1_733_26	chr14	78152476	+	842_121_1	chr14	78111248	+
2568	1,25	DIO2	NM_013989	chr14	79733620	79747723	-	.	.	.	.	.	.	.	.	.	.	.	.	.	.	.	.	.	.	.	.
437	3,5	TSHR	NM_001018036	chr14	80491621	80645047	+	.	.	.	.	.	.	.	.	.	.	.	.	.	.	.	.	.	.	.	.
3985	1	GTF2A1	NR_048573	chr14	80711549	80757049	-	.	.	.	.	.	.	.	.	.	.	.	.	.	.	.	.	.	.	.	.
5392	1	STON2	NM_001256430	chr14	80796746	80963501	-	.	.	.	.	.	.	.	.	.	.	.	.	.	.	.	.	.	.	.	.
1742	2	SEL1L	NM_001244984	chr14	81034906	81069958	-	CL800466	chr14	81011893	-	.	.	.	.	.	.	.	.	.	.	.	.	.	.	.	.
3787	1	FLRT2	NM_013231	chr14	85066240	85164023	+	CL528778	chr14	85113259	-	.	.	.	.	.	.	.	.	.	.	.	.	.	.	.	.
3838	1	GALC	NM_000153	chr14	87469110	87529368	-	.	.	.	.	.	.	.	.	.	.	.	.	.	.	.	.	.	.	.	.
2171	1,5	GPR65	NM_003608	chr14	87541220	87550908	+	.	.	.	.	.	.	.	.	.	.	.	.	.	.	.	.	.	.	.	.
4997	1	PTPN21	NM_007039	chr14	88001874	88090876	-	.	.	.	.	.	.	.	.	.	.	.	.	.	.	.	.	.	.	.	.
5449	1	TDP1	NM_001008744	chr14	89491998	89580861	+	.	.	.	.	.	.	.	.	.	.	.	.	.	.	.	.	.	.	.	.
536	3	CALM1	NM_006888	chr14	89933079	89944372	+	.	.	.	.	.	.	.	.	.	.	.	.	.	.	.	.	.	.	.	.
7061	0,5	TTC7B	NM_001010854	chr14	90076684	90352514	-	CL800259	chr14	90212206	+	.	.	.	.	.	.	.	.	534_246	chr14	90322093	+	.	.	.	.
1732	2	RPS6KA5	NM_004755	chr14	90406922	90596746	-	CL799726	chr14	90419540	+	.	.	.	.	.	.	.	.	.	.	.	.	.	.	.	.

Table S2

tumor associated genes								HIV				MLV				MMTV				MMTV(SIN)				MMTV(SIN)arrest			
rank	score	gene symbol	RefSEq	chrom	txStart	txEnd	strand	integrant	chr	position	strand	integrant	chr	position	strand	integrant	chr	position	strand	integrant	chr	position	strand	integrant	chr	position	strand
3282	1	CATSPERB	NM_024764	chr14	91116870	91268166	-	.	.	.	.	.	.	.	.	.	.	.	.	.	.	.	.	.	.	.	.
2135	1,5	FBLN5	NM_006329	chr14	91405507	91483799	-	.	.	.	.	.	.	.	.	.	.	.	.	.	.	.	.	.	.	.	.
511	3,25	TRIP11	NM_004239	chr14	91503995	91576156	-	.	.	.	.	.	.	.	.	.	.	.	.	.	.	.	.	.	.	.	.
3102	1	ATXN3	NM_001164778	chr14	91594648	91642718	-	.	.	.	.	.	.	.	.	.	.	.	.	.	.	.	.	.	.	.	.
4535	1	NDUFB1	NM_004545	chr14	91652220	91657906	-	.	.	.	.	.	.	.	.	.	.	.	.	.	.	.	.	.	.	.	.
1058	2,25	GOLGA5	NM_005113	chr14	92330402	92376057	+	.	.	.	.	.	.	.	.	.	.	.	.	.	.	.	.	.	.	.	.
3351	1	CHGA	NM_001275	chr14	92459197	92471393	+	.	.	.	.	.	.	.	.	.	.	.	.	.	.	.	.	.	.	.	.
389	3,5	ITPK1	NM_001142594	chr14	92473011	92652016	-	.	.	.	.	.	.	.	.	.	.	.	.	.	.	.	.	.	.	.	.
6621	0,5	MOAP1	NM_022151	chr14	92718293	92721002	-	.	.	.	.	.	.	.	.	.	.	.	.	.	.	.	.	.	.	.	.
3444	1	COX8C	NM_182971	chr14	92883289	92884453	+	.	.	.	.	.	.	.	.	.	.	.	.	.	.	.	.	.	.	.	.
4115	1	IFI27	NM_005532	chr14	93646831	93652786	+	.	.	.	.	.	.	.	.	.	.	.	.	.	.	.	.	.	.	.	.
4927	1	PPP4R4	NM_020958	chr14	93710401	93763935	+	.	.	.	.	.	.	.	.	.	.	.	.	.	.	.	.	.	.	.	.
6876	0,5	SERPINA6	NM_001756	chr14	93840337	93859441	-	.	.	.	.	.	.	.	.	.	.	.	.	.	.	.	.	.	.	.	.
1963	1,75	SERPINA1	NM_001127707	chr14	93912836	93926782	-	CL529130	chr14	93913115	-	.	.	.	.	.	.	.	.	.	.	.	.	.	.	.	.
5211	1	SERPINA12	NM_173850	chr14	94023372	94053934	-	.	.	.	.	.	.	.	.	.	.	.	.	.	.	.	.	.	.	.	.
7537	0,25	SERPINA5	NM_000624	chr14	94117483	94129210	+	.	.	.	.	.	.	.	.	.	.	.	.	.	.	.	.	.	.	.	.
1964	1,75	SERPINA3	NM_001085	chr14	94148466	94160143	+	.	.	.	.	.	.	.	.	.	.	.	.	.	.	.	.	.	.	.	.
1357	2	DICER1	NM_030621	chr14	94622317	94677808	-	.	.	.	.	.	.	.	.	.	.	.	.	.	.	.	.	.	.	.	.
1799	2	TCL6	NR_028288	chr14	95187267	95209542	+	.	.	.	.	.	.	.	.	.	.	.	.	.	.	.	.	.	.	.	.
1172	2,25	TCL1A	NM_021966	chr14	95246056	95250286	-	.	.	.	.	.	.	.	.	.	.	.	.	.	.	.	.	.	.	.	.
5881	0,75	BDKRB2	NM_000623	chr14	95740887	95780419	+	.	.	.	.	.	.	.	.	.	.	.	.	.	.	.	.	.	.	.	.
2939	1	AK7	NM_152327	chr14	95928200	96025517	+	.	.	.	.	.	.	.	.	.	.	.	.	.	.	.	.	.	.	.	.
5680	1	VRK1	NM_003384	chr14	96333436	96417704	+	CL529131	chr14	96403827	-	.	.	.	.	.	.	.	.	.	.	.	.	.	.	.	.
793	2,5	BCL11B	NM_022898	chr14	98705377	98807575	-	.	.	.	.	.	.	.	.	.	.	.	.	.	.	.	.	.	.	.	.
7540	0,25	SETD3	NM_032233	chr14	98933835	99016979	-	.	.	.	.	.	.	.	.	.	.	.	.	.	.	.	.	.	.	.	.
6241	0,5	CYP46A1	NM_006668	chr14	99220507	99263391	+	.	.	.	.	.	.	.	.	.	.	.	.	.	.	.	.	.	.	.	.
2464	1,5	YY1	NM_003403	chr14	99774854	99814557	+	.	.	.	.	.	.	.	.	.	.	.	.	.	.	.	.	.	.	.	.
5684	1	WARS	NM_173701	chr14	99869877	99912433	-	.	.	.	.	.	.	.	.	.	.	.	.	.	.	.	.	.	.	.	.
3141	1	BEGAIN	NM_020836	chr14	100073236	100105884	-	.	.	.	.	.	.	.	.	.	.	.	.	.	.	.	.	.	.	.	.
7277	0,25	DLK1	NM_003836	chr14	100262954	100271220	+	.	.	.	.	.	.	.	.	.	.	.	.	.	.	.	.	.	.	.	.
7276	0,25	DIO3	NM_001362	chr14	101097440	101099542	+	.	.	.	.	.	.	.	.	.	.	.	.	.	.	.	.	.	.	.	.
4924	1	PPP2R5C	NM_002719	chr14	101345892	101464081	+	.	.	.	.	.	.	.	.	.	.	.	.	2384_260	chr14	101357280	-	.	.	.	.
55	7	HSP90AA1	NM_001017963	chr14	101616827	101675839	-	.	.	.	.	.	.	.	.	.	.	.	.	.	.	.	.	.	.	.	.
1300	2	CDC42BPB	NM_006035	chr14	102468468	102593495	-	AY517269.1	chr14	102498119	-	.	.	.	.	.	.	.	.	.	.	.	.	.	.	.	.
3671	1	EIF5	NM_183004	chr14	102870950	102881114	+	.	.	.	.	.	.	.	.	.	.	.	.	.	.	.	.	.	.	.	.
4358	1	MARK3	NM_001128921	chr14	102921453	103039919	+	BH609707	chr14	103010064	+	.	.	.	.	.	.	.	.	.	.	.	.	.	.	.	.
4358	1	MARK3	NM_001128921	chr14	102921453	103039919	+	CL529132	chr14	103026961	+	.	.	.	.	.	.	.	.	.	.	.	.	.	.	.	.
454	3,25	CKB	NM_001823	chr14	103055747	103058949	-	.	.	.	.	.	.	.	.	.	.	.	.	.	.	.	.	.	.	.	.
1185	2,25	XRCC3	NM_001100118	chr14	103233706	103251576	-	.	.	.	.	.	.	.	.	.	.	.	.	.	.	.	.	.	.	.	.
933	2,5	PPP1R13B	NM_015316	chr14	103269840	103383680	-	.	.	.	.	.	.	.	.	.	.	.	.	.	.	.	.	.	.	.	.
7204	0,25	C14orf180	NM_001008404	chr14	104117100	104127228	+	.	.	.	.	.	.	.	.	.	.	.	.	.	.	.	.	.	.	.	.
7594	0,25	TMEM179	NM_207379	chr14	104131464	104142142	-	.	.	.	.	.	.	.	.	.	.	.	.	.	.	.	.	.	.	.	.
7373	0,25	INF2	NM_001031714	chr14	104226987	104256992	+	.	.	.	.	.	.	.	.	.	.	.	.	.	.	.	.	.	.	.	.
1991	1,5	ADSSL1	NM_199165	chr14	104267272	104284692	+	.	.	.	.	.	.	.	.	.	.	.	.	.	.	.	.	.	.	.	.
7547	0,25	SIVA1	NM_021709	chr14	104290514	104297041	+	CL529134	chr14	104294444	+	.	.	.	.	.	.	.	.	.	.	.	.	.	.	.	.
36	8	AKT1	NM_001014431	chr14	104306731	104333125	-	.	.	.	.	.	.	.	.	.	.	.	.	.	.	.	.	.	.	.	.
7385	0,25	KIAA0284	NM_015005	chr14	104402694	104434152	+	.	.	.	.	.	.	.	.	.	.	.	.	.	.	.	.	.	.	.	.
6750	0,5	PLD4	NM_138790	chr14	104462231	104470618	+	.	.	.	.	.	.	.	.	.	.	.	.	.	.	.	.	.	.	.	.
6001	0,5	AHNAK2	NM_138420	chr14	104474635	104515739	-	.	.	.	.	.	.	.	.	.	.	.	.	.	.	.	.	.	.	.	.
1485	2	JAG2	NM_145159	chr14	104678362	104706206	-	.	.	.	.	.	.	.	.	.	.	.	.	.	.	.	.	.	.	.	.
6065	0,5	BRF1	NM_145685	chr14	104746667	104785267	-	.	.	.	.	.	.	.	.	.	.	.	.	.	.	.	.	.	.	.	.

Table S2

tumor associated genes								HIV				MLV				MMTV				MMTV(SIN)				MMTV(SIN)arrest			
rank	score	gene symbol	RefSEq	chrom	txStart	txEnd	strand	integrant	chr	position	strand	integrant	chr	position	strand	integrant	chr	position	strand	integrant	chr	position	strand	integrant	chr	position	strand
2676	1,25	MKRN3	NM_005664	chr15	21361546	21364259	+	.	.	.	.	.	.	.	.	.	.	.	.	.	.	.	.	.	.	.	.
1564	2	NDN	NM_002487	chr15	21481646	21483543	-	.	.	.	.	.	.	.	.	.	.	.	.	.	.	.	.	.	.	.	.
2780	1,25	SNRPN	NM_022807	chr15	22619886	22774822	+	.	.	.	.	.	.	.	.	.	.	.	.	.	.	.	.	.	.	.	.
5610	1	UBE3A	NM_000462	chr15	23133488	23235268	-	CL800369	chr15	23185234	+	.	.	.	.	.	.	.	.	.	.	.	.	.	.	.	.
6040	0,5	ATP10A	NM_024490	chr15	23474952	23659442	-	CL800695	chr15	23612739	+	.	.	.	.	.	.	.	.	.	.	.	.	.	.	.	.
7322	0,25	GABRB3	NM_001191321	chr15	24339786	24425330	-	.	.	.	.	.	.	.	.	.	.	.	.	.	.	.	.	.	.	.	.
2146	1,5	GABRA5	NM_000810	chr15	24662958	24777103	+	.	.	.	.	.	.	.	.	.	.	.	.	.	.	.	.	.	.	.	.
3834	1	GABRG3	NM_001270873	chr15	24799174	25400074	+	.	.	.	.	.	.	.	.	.	.	.	.	.	.	.	.	.	.	.	.
4655	1	OCA2	NM_000275	chr15	25673615	26018053	-	.	.	.	.	.	.	.	.	.	.	.	.	.	.	.	.	.	.	.	.
586	3	HERC2	NM_004667	chr15	26029777	26240893	-	.	.	.	.	.	.	.	.	.	.	.	.	.	.	.	.	.	.	.	.
7023	0,5	TJP1	NM_175610	chr15	27779648	27901998	-	.	.	.	.	.	.	.	.	.	.	.	.	.	.	.	.	.	.	.	.
2004	1,5	ARHGAP11A	NM_014783	chr15	30694982	30719160	+	.	.	.	.	.	.	.	.	.	.	.	.	.	.	.	.	.	.	.	.
6851	0,5	SCG5	NM_003020	chr15	30721161	30776590	+	.	.	.	.	.	.	.	.	.	.	.	.	.	.	.	.	.	.	.	.
7335	0,25	GREM1	NM_001191323	chr15	30797496	30814162	+	.	.	.	.	.	.	.	.	.	.	.	.	.	.	.	.	.	.	.	.
950	2,5	RYR3	NM_001036	chr15	31390468	31945595	+	.	.	.	.	.	.	.	.	.	.	.	.	.	.	.	.	.	.	.	.
2507	1,25	AVEN	NM_020371	chr15	31945719	32118595	-	.	.	.	.	.	.	.	.	.	.	.	.	.	.	.	.	.	.	.	.
4767	1	PGBD4	NM_152595	chr15	32181565	32183883	+	.	.	.	.	.	.	.	.	.	.	.	.	.	.	.	.	.	.	.	.
3195	1	C15orf29	NM_024713	chr15	32220166	32289589	-	.	.	.	.	.	.	.	.	.	.	.	.	.	.	.	.	.	.	.	.
5254	1	SLC12A6	NM_001042495	chr15	32309488	32417557	-	.	.	.	.	.	.	.	.	.	.	.	.	.	.	.	.	.	.	.	.
1282	2	C15orf55	NM_175741	chr15	32425357	32437223	+	.	.	.	.	.	.	.	.	.	.	.	.	.	.	.	.	.	.	.	.
6569	0,5	LPCAT4	NM_153613	chr15	32438380	32446687	-	.	.	.	.	.	.	.	.	.	.	.	.	.	.	.	.	.	.	.	.
6392	0,5	GOLGA8A	NR_027409	chr15	32458561	32516959	-	.	.	.	.	.	.	.	.	.	.	.	.	.	.	.	.	.	.	.	.
2894	1	ACTC1	NM_005159	chr15	32867588	32875219	-	.	.	.	.	.	.	.	.	.	.	.	.	.	.	.	.	.	.	.	.
1250	2	AQR	NM_014691	chr15	32935843	33049287	-	CL800438	chr15	32972185	+	.	.	.	.	.	.	.	.	.	.	.	.	.	.	.	.
5854	1	ZNF770	NM_014106	chr15	33057833	33067789	-	.	.	.	.	.	.	.	.	.	.	.	.	.	.	.	.	.	.	.	.
3100	1	ATPBD4	NM_080650	chr15	33450461	33625696	-	.	.	.	.	.	.	.	.	.	.	.	.	.	.	.	.	.	.	.	.
3196	1	C15orf41	NM_001130010	chr15	34659103	34889741	+	.	.	.	.	.	.	.	.	.	.	.	.	.	.	.	.	.	.	.	.
4379	1	MEIS2	NM_170677	chr15	34970513	35179633	-	.	.	.	.	.	.	.	.	.	.	.	.	.	.	.	.	.	.	.	.
7590	0,25	TMCO5A	NM_152453	chr15	36014748	36030915	+	.	.	.	.	.	.	.	.	.	.	.	.	.	.	.	.	.	.	.	.
2785	1,25	SPRED1	NM_152594	chr15	36332343	36436742	+	.	.	.	.	.	.	.	.	.	.	.	.	.	.	.	.	.	.	.	.
2584	1,25	FAM98B	NM_001042429	chr15	36533619	36561121	+	CL529709	chr15	36551466	+	.	.	.	.	.	.	.	.	.	.	.	.	.	.	.	.
1154	2,25	RASGRP1	NM_005739	chr15	36567593	36644299	-	.	.	.	.	.	.	.	.	.	.	.	.	.	.	.	.	.	.	.	.
7205	0,25	C15orf53	NM_207444	chr15	36776090	36779531	+	.	.	.	.	.	.	.	.	.	.	.	.	.	.	.	.	.	.	.	.
7206	0,25	C15orf54	NM_207445	chr15	37330176	37334340	+	.	.	.	.	.	.	.	.	.	.	.	.	.	.	.	.	.	.	.	.
233	4,25	THBS1	NM_003246	chr15	37660571	37676960	+	.	.	.	.	.	.	.	.	.	.	.	.	.	.	.	.	.	.	.	.
2592	1,25	FSIP1	NM_152597	chr15	37679523	37862331	-	.	.	.	.	.	.	.	.	.	.	.	.	.	.	.	.	.	.	.	.
2170	1,5	GPR176	NM_007223	chr15	37880222	38000385	-	.	.	.	.	.	.	.	.	.	.	.	.	.	.	.	.	.	.	.	.
1038	2,25	EIF2AK4	NM_001013703	chr15	38013638	38115089	+	.	.	.	.	.	.	.	.	.	.	.	.	.	.	.	.	.	.	.	.
2786	1,25	SRP14	NM_003134	chr15	38115182	38118681	-	.	.	.	.	.	.	.	.	.	.	.	.	.	.	.	.	.	.	.	.
100	5,5	BUB1B	NM_001211	chr15	38240501	38300629	+	.	.	.	.	.	.	.	.	.	.	.	.	.	.	.	.	.	.	.	.
409	3,5	PAK6	NM_001128629	chr15	38296920	38356980	+	.	.	.	.	.	.	.	.	.	.	.	.	.	.	.	.	.	.	.	.
642	3	PLCB2	NM_004573	chr15	38367389	38387466	-	.	.	.	.	.	.	.	.	.	.	.	.	.	.	.	.	.	.	.	.
3198	1	C15orf52	NM_207380	chr15	38410944	38420460	-	.	.	.	.	.	.	.	.	.	.	.	.	.	.	.	.	.	.	.	.
3193	1	C15orf23	NM_033286	chr15	38462213	38473781	+	.	.	.	.	.	.	.	.	.	.	.	.	.	.	.	.	.	.	.	.
1484	2	IVD	NM_002225	chr15	38484977	38500804	+	.	.	.	.	.	.	.	.	.	.	.	.	.	.	.	.	.	.	.	.
3125	1	BAHD1	NM_014952	chr15	38520702	38547733	+	.	.	.	.	.	.	.	.	.	.	.	.	.	.	.	.	.	.	.	.
1311	2	CHST14	NM_130468	chr15	38550451	38552649	+	.	.	.	.	.	.	.	.	.	.	.	.	.	.	.	.	.	.	.	.
3199	1	C15orf57	NM_001080791	chr15	38632589	38644544	-	.	.	.	.	.	.	.	.	.	.	.	.	.	.	.	.	.	.	.	.
5166	1	RPUSD2	NM_152260	chr15	38648828	38653953	+	.	.	.	.	.	.	.	.	.	.	.	.	.	.	.	.	.	.	.	.
446	3,25	CASC5	NM_170589	chr15	38673738	38742173	+	.	.	.	.	.	.	.	.	.	.	.	.	.	.	.	.	.	.	.	.
113	5,5	RAD51	NM_001164269	chr15	38774669	38811648	+	.	.	.	.	.	.	.	.	.	.	.	.	.	.	.	.	.	.	.	.

Table S2

tumor associated genes								HIV				MLV				MMTV				MMTV(SIN)				MMTV(SIN)arrest			
rank	score	gene symbol	RefSEq	chrom	txStart	txEnd	strand	integrant	chr	position	strand	integrant	chr	position	strand	integrant	chr	position	strand	integrant	chr	position	strand	integrant	chr	position	strand
5747	1	ZFYVE19	NM_001258421	chr15	38886565	38894059	+	.	.	.	.	.	.	.	.	.	.	.	.	.	.	.	.	.	.	.	.
4912	1	PPP1R14D	NM_001130143	chr15	38894934	38908199	-	.	.	.	.	.	.	.	.	.	.	.	.	.	.	.	.	.	.	.	.
5339	1	SPINT1	NM_003710	chr15	38923537	38937145	+	.	.	.	.	.	.	.	.	.	.	.	.	.	.	.	.	.	.	.	.
1360	2	DLL4	NM_019074	chr15	39008822	39018550	+	.	.	.	.	.	.	.	.	.	.	.	.	.	.	.	.	.	.	.	.
6323	0,5	EXD1	NM_152596	chr15	39262222	39310187	-	.	.	.	.	.	.	.	.	.	.	.	.	.	.	.	.	.	.	.	.
6698	0,5	OIP5	NM_007280	chr15	39388757	39412111	-	.	.	.	.	.	.	.	.	.	.	.	.	.	.	.	.	.	.	.	.
4534	1	NDUFAF1	NM_016013	chr15	39466838	39481950	-	.	.	.	.	.	.	.	.	.	.	.	.	.	.	.	.	.	.	.	.
605	3	ITPKA	NM_002220	chr15	39573347	39583049	+	.	.	.	.	.	.	.	.	.	.	.	.	.	.	.	.	.	.	.	.
481	3,25	LTK	NM_206961	chr15	39583131	39593377	-	.	.	.	.	.	.	.	.	.	.	.	.	.	.	.	.	.	.	.	.
978	2,5	TYRO3	NM_006293	chr15	39638511	39658828	+	.	.	.	.	.	.	.	.	.	.	.	.	.	.	.	.	.	.	.	.
621	3	MGA	NM_001080541	chr15	39739901	39849433	+	BH609875	chr15	39764404	-	.	.	.	.	.	.	.	.	.	.	.	.	.	.	.	.
396	3,5	MAPKBP1	NM_001265611	chr15	39853923	39907345	+	BH609712	chr15	39867658	-	.	.	.	.	.	.	.	.	.	.	.	.	.	.	.	.
4192	1	JMJD7	NM_001114632	chr15	39907574	39917077	+	.	.	.	.	.	.	.	.	.	.	.	.	.	.	.	.	.	.	.	.
4193	1	JMJD7-PLA2G4E	NM_005090	chr15	39907574	39927638	+	.	.	.	.	.	.	.	.	.	.	.	.	.	.	.	.	.	.	.	.
4833	1	PLA2G4B	NM_001114633	chr15	39918302	39927638	+	.	.	.	.	.	.	.	.	.	.	.	.	.	.	.	.	.	.	.	.
5344	1	SPTBN5	NM_016642	chr15	39927635	39973567	-	.	.	.	.	.	.	.	.	.	.	.	.	.	.	.	.	.	.	.	.
3854	1	GANC	NM_198141	chr15	40353657	40433156	+	.	.	.	.	.	.	.	.	.	.	.	.	.	.	.	.	.	.	.	.
6130	0,5	CAPN3	NM_173090	chr15	40484268	40491807	+	.	.	.	.	.	.	.	.	.	.	.	.	.	.	.	.	.	.	.	.
7121	0,5	ZFP106	NM_022473	chr15	40492312	40537022	-	.	.	.	.	.	.	.	.	.	.	.	.	.	.	.	.	.	.	.	.
5580	1	TTBK2	NM_173500	chr15	40823835	41000299	-	CL800023	chr15	40960130	+	.	.	.	.	.	.	.	.	.	.	.	.	1718_161_1	chr15	40913499	-
5580	1	TTBK2	NM_173500	chr15	40823835	41000299	-	CL800396	chr15	40976909	-	.	.	.	.	.	.	.	.	.	.	.	.	1718_161_1	chr15	40913499	-
4268	1	LCMT2	NM_014793	chr15	41407265	41410112	-	.	.	.	.	.	.	.	.	.	.	.	.	.	.	.	.	.	.	.	.
974	2,5	TP53BP1	NM_001141980	chr15	41486703	41572646	-	BH609713	chr15	41555570	+	.	.	.	.	.	.	.	.	.	.	.	.	.	.	.	.
2075	1,5	CKMT1B	NM_020990	chr15	41672543	41678896	+	.	.	.	.	.	.	.	.	.	.	.	.	.	.	.	.	.	.	.	.
3375	1	CKMT1A	NM_001015001	chr15	41772375	41778712	+	.	.	.	.	.	.	.	.	.	.	.	.	.	.	.	.	.	.	.	.
4744	1	PDIA3	NM_005313	chr15	41825881	41852096	+	.	.	.	.	.	.	.	.	.	.	.	.	.	.	.	.	.	.	.	.
5316	1	SORD	NM_003104	chr15	43102593	43154579	+	.	.	.	.	.	.	.	.	.	.	.	.	.	.	.	.	.	.	.	.
3859	1	GATM	NM_001482	chr15	43440613	43458272	-	.	.	.	.	.	.	.	.	.	.	.	.	.	.	.	.	.	.	.	.
6969	0,5	SQRDL	NM_021199	chr15	43714547	43770771	+	.	.	.	.	.	.	.	.	.	.	.	.	.	.	.	.	.	.	.	.
6871	0,5	SEMA6D	NM_153619	chr15	45797977	45853712	+	.	.	.	.	.	.	.	.	.	.	.	.	.	.	.	.	.	.	.	.
1371	2	DUT	NM_001948	chr15	46411656	46422862	+	.	.	.	.	.	.	.	.	.	.	.	.	.	.	.	.	.	.	.	.
6890	0,5	SHC4	NM_203349	chr15	46903225	47042933	-	.	.	.	.	.	.	.	.	.	.	.	.	.	.	.	.	.	.	.	.
3841	1	GALK2	NM_001001556	chr15	47235267	47409294	+	CL799998	chr15	47292728	-	.	.	.	.	.	.	.	.	.	.	.	.	.	.	.	.
3775	1	FGF7	NM_002009	chr15	47502666	47566815	+	.	.	.	.	.	.	.	.	.	.	.	.	.	.	.	.	.	.	.	.
2613	1,25	HDC	NM_002112	chr15	48321437	48345454	-	.	.	.	.	.	.	.	.	.	.	.	.	.	.	.	.	.	.	.	.
5661	1	USP8	NM_001128611	chr15	48503870	48580569	+	.	.	.	.	.	.	.	.	.	.	.	.	.	.	.	.	.	.	.	.
5565	1	TRPM7	NM_017672	chr15	48636643	48766304	-	.	.	.	.	.	.	.	.	.	.	.	.	.	.	.	.	.	.	.	.
206	4,25	CYP19A1	NM_000103	chr15	49287545	49418087	-	.	.	.	.	.	.	.	.	.	.	.	.	.	.	.	.	.	.	.	.
6385	0,5	GLDN	NM_181789	chr15	49421004	49487501	+	.	.	.	.	.	.	.	.	.	.	.	.	.	.	.	.	.	.	.	.
5195	1	SCG3	NM_001165257	chr15	49760841	49800515	+	.	.	.	.	.	.	.	.	.	.	.	.	.	.	.	.	.	.	.	.
2662	1,25	MAPK6	NM_002748	chr15	50098702	50145754	+	.	.	.	.	.	.	.	.	.	.	.	.	.	.	.	.	.	.	.	.
6054	0,5	BCL2L10	NM_020396	chr15	50189113	50192264	-	.	.	.	.	.	.	.	.	.	.	.	.	.	.	.	.	.	.	.	.
3923	1	GNB5	NM_006578	chr15	50200414	50259454	-	.	.	.	.	.	.	.	.	.	.	.	.	.	.	.	.	.	.	.	.
2268	1,5	MYO5C	NM_018728	chr15	50271806	50375287	-	BH609717	chr15	50285192	-	.	.	.	.	.	.	.	.	.	.	.	.	.	.	.	.
7077	0,5	UNC13C	NM_001080534	chr15	52092392	52708098	+	CL800688	chr15	52592585	-	.	.	.	.	.	.	.	.	.	.	.	.	.	.	.	.
2358	1,5	RAB27A	NM_183235	chr15	53282456	53369305	-	.	.	.	.	.	.	.	.	.	.	.	.	.	.	.	.	.	.	.	.
4796	1	PIGB	NM_004855	chr15	53398424	53435138	+	.	.	.	.	.	.	.	.	.	.	.	.	.	.	.	.	.	.	.	.
1571	2	NEDD4	NM_198400	chr15	53906413	53996621	-	CL529142	chr15	53953904	-	AY516336	chr15	53992781	+	.	.	.	.	.	.	.	.	.	.	.	.
147	5	TCF12	NM_207038	chr15	54998124	55368006	+	CL800481	chr15	55135178	-	.	.	.	.	.	.	.	.	.	.	.	.	.	.	.	.
147	5	TCF12	NM_207038	chr15	54998124	55368006	+	CL529535	chr15	55265790	+	.	.	.	.	.	.	.	.	.	.	.	.	.	.	.	.
2947	1	ALDH1A2	NM_170696	chr15	56032913	56145413	-	.	.	.	.	.	.	.	.	.	.	.	.	.	.	.	.	.	.	.	.

Table S2

tumor associated genes								HIV				MLV				MMTV				MMTV(SIN)				MMTV(SIN)arrest			
rank	score	gene symbol	RefSEq	chrom	txStart	txEnd	strand	integrant	chr	position	strand	integrant	chr	position	strand	integrant	chr	position	strand	integrant	chr	position	strand	integrant	chr	position	strand
5886	0,75	CCNB2	NM_004701	chr15	57184575	57204536	+	.	.	.	.	.	.	.	.	.	.	.	.	.	.	.	.	.	.	.	.
5936	0,75	MYO1E	NM_004998	chr15	57215459	57452363	-	.	.	.	.	.	.	.	.	.	.	.	.	s1740_280	chr15	57446086	+	.	.	.	.
4272	1	LDHAL6B	NM_033195	chr15	57286306	57288077	+	.	.	.	.	.	.	.	.	.	.	.	.	.	.	.	.	.	.	.	.
6342	0,5	FAM81A	NM_152450	chr15	57517663	57603043	+	.	.	.	.	.	.	.	.	.	.	.	.	.	.	.	.	.	.	.	.
3870	1	GCNT3	NM_004751	chr15	57691273	57699502	+	.	.	.	.	.	.	.	.	.	.	.	.	.	.	.	.	.	.	.	.
7193	0,25	BNIP2	NM_004330	chr15	57742353	57768934	-	.	.	.	.	.	.	.	.	.	.	.	.	.	.	.	.	.	.	.	.
1999	1,5	ANXA2	NM_001002857	chr15	58426641	58477477	-	.	.	.	.	.	.	.	.	.	.	.	.	.	.	.	.	.	.	.	.
5149	1	RORA	NM_134262	chr15	58567774	58671999	-	.	.	.	.	.	.	.	.	.	.	.	.	.	.	.	.	.	.	.	.
5045	1	RAB8B	NM_016530	chr15	61268780	61347026	+	.	.	.	.	.	.	.	.	.	.	.	.	.	.	.	.	.	.	.	.
1882	1,75	CA12	NM_206925	chr15	61402782	61461128	-	.	.	.	.	.	.	.	.	.	.	.	.	.	.	.	.	.	.	.	.
2182	1,5	HERC1	NM_003922	chr15	61687869	61913200	-	.	.	.	.	.	.	.	.	.	.	.	.	.	.	.	.	.	.	.	.
3528	1	DAPK2	NM_014326	chr15	61986287	62125574	-	.	.	.	.	.	.	.	.	.	.	.	.	.	.	.	.	.	.	.	.
7487	0,25	PPIB	NM_000942	chr15	62235066	62242407	-	.	.	.	.	.	.	.	.	.	.	.	.	.	.	.	.	.	.	.	.
3470	1	CSNK1G1	NM_022048	chr15	62244768	62435495	-	.	.	.	.	AY516341	chr15	62326465	-	.	.	.	.	.	.	.	.	.	.	.	.
3470	1	CSNK1G1	NM_022048	chr15	62244768	62435495	-	.	.	.	.	AY516646	chr15	62405632	+	.	.	.	.	.	.	.	.	.	.	.	.
6516	0,5	KIAA0101	NM_014736	chr15	62444263	62460755	-	.	.	.	.	.	.	.	.	.	.	.	.	.	.	.	.	.	.	.	.
4452	1	MTFMT	NM_139242	chr15	63080902	63109030	-	BH609720	chr15	63099240	+	.	.	.	.	.	.	.	.	.	.	.	.	.	.	.	.
6182	0,5	CILP	NM_003613	chr15	63275389	63290893	-	.	.	.	.	.	.	.	.	.	.	.	.	.	.	.	.	.	.	.	.
4991	1	PTPLAD1	NM_016395	chr15	63609879	63657746	+	.	.	.	.	.	.	.	.	.	.	.	.	.	.	.	.	.	.	.	.
5018	1	RAB11A	NM_001206836	chr15	63948850	63971383	+	.	.	.	.	.	.	.	.	.	.	.	.	.	.	.	.	.	.	.	.
120	5,25	MAP2K1	NM_002755	chr15	64466264	64570936	+	.	.	.	.	.	.	.	.	.	.	.	.	.	.	.	.	.	.	.	.
5873	1	ZWILCH	NR_003105	chr15	64584484	64628876	+	.	.	.	.	.	.	.	.	.	.	.	.	.	.	.	.	.	.	.	.
754	2,75	SMAD6	NM_001142861	chr15	64787983	64861391	+	.	.	.	.	.	.	.	.	.	.	.	.	.	.	.	.	.	.	.	.
164	4,75	SMAD3	NM_001145103	chr15	65217412	65274587	+	.	.	.	.	.	.	.	.	.	.	.	.	.	.	.	.	.	.	.	.
4343	1	MAP2K5	NM_001206804	chr15	65628395	65886509	+	BH609722	chr15	65803566	+	.	.	.	.	.	.	.	.	.	.	.	.	.	.	.	.
2321	1,5	PIAS1	NM_016166	chr15	66133625	66267458	+	.	.	.	.	.	.	.	.	.	.	.	.	.	.	.	.	.	.	.	.
7378	0,25	ITGA11	NM_001004439	chr15	66381095	66511546	-	.	.	.	.	.	.	.	.	.	.	.	.	.	.	.	.	.	.	.	.
3901	1	GLCE	NM_015554	chr15	67240026	67351598	+	.	.	.	.	.	.	.	.	.	.	.	.	.	.	.	.	.	.	.	.
5474	1	TLE3	NM_020908	chr15	68127596	68177310	-	.	.	.	.	.	.	.	.	.	.	.	.	.	.	.	.	.	.	.	.
6549	0,5	LARP6	NM_197958	chr15	68930821	68933552	-	.	.	.	.	.	.	.	.	.	.	.	.	.	.	.	.	.	.	.	.
4613	1	NR2E3	NM_014249	chr15	69889947	69897651	+	.	.	.	.	.	.	.	.	.	.	.	.	.	.	.	.	.	.	.	.
2183	1,5	HEXA	NM_000520	chr15	70422831	70455574	-	.	.	.	.	.	.	.	.	.	.	.	.	.	.	.	.	.	.	.	.
4565	1	NEO1	NM_001172624	chr15	71131877	71384600	+	.	.	.	.	.	.	.	.	.	.	.	.	.	.	.	.	.	.	.	.
6681	0,5	NPTN	NM_017455	chr15	71639396	71712806	-	CL800015	chr15	71702466	-	.	.	.	.	.	.	.	.	.	.	.	.	2143_186_1	chr15	71692606	-
6567	0,5	LOXL1	NM_005576	chr15	72005841	72031531	+	.	.	.	.	.	.	.	.	.	.	.	.	.	.	.	.	.	.	.	.
188	4,5	PML	NM_033244	chr15	72074066	72115788	+	CL529666	chr15	72083623	+	.	.	.	.	.	.	.	.	.	.	.	.	.	.	.	.
188	4,5	PML	NM_033244	chr15	72074066	72115788	+	AY517169.1	chr15	72079380	-	.	.	.	.	.	.	.	.	.	.	.	.	.	.	.	.
1335	2	CYP11A1	NM_001099773	chr15	72417155	72445606	-	.	.	.	.	.	.	.	.	.	.	.	.	.	.	.	.	.	.	.	.
3391	1	CLK3	NM_001130028	chr15	72694387	72709595	+	.	.	.	.	.	.	.	.	.	.	.	.	.	.	.	.	.	.	.	.
456	3,25	CYP1A1	NM_000499	chr15	72798935	72804930	-	.	.	.	.	.	.	.	.	.	.	.	.	.	.	.	.	.	.	.	.
457	3,25	CYP1A2	NM_000761	chr15	72828236	72835994	+	.	.	.	.	.	.	.	.	.	.	.	.	.	.	.	.	.	.	.	.
2097	1,5	CSK	NM_001127190	chr15	72861477	72882592	+	.	.	.	.	.	.	.	.	.	.	.	.	.	.	.	.	.	.	.	.
5640	1	ULK3	NM_001099436	chr15	72915511	72922605	-	.	.	.	.	.	.	.	.	.	.	.	.	.	.	.	.	.	.	.	.
4430	1	MPI	NM_002435	chr15	72969462	72977618	+	.	.	.	.	.	.	.	.	.	.	.	.	.	.	.	.	.	.	.	.
3435	1	COX5A	NM_004255	chr15	72999669	73017548	-	AY517212.1	chr15	73008160	+	.	.	.	.	.	.	.	.	.	.	.	.	.	.	.	.
5162	1	RPP25	NM_017793	chr15	73034495	73036828	-	.	.	.	.	.	.	.	.	.	.	.	.	.	.	.	.	.	.	.	.
6849	0,5	SCAMP5	NM_001178112	chr15	73074928	73100889	+	.	.	.	.	.	.	.	.	.	.	.	.	.	.	.	.	.	.	.	.
4893	1	PPCDC	NM_021823	chr15	73102979	73130120	+	.	.	.	.	.	.	.	.	.	.	.	.	.	.	.	.	.	.	.	.
4556	1	NEIL1	NR_046311	chr15	73427012	73434645	+	.	.	.	.	.	.	.	.	.	.	.	.	.	.	.	.	.	.	.	.
4340	1	MAN2C1	NM_001256495	chr15	73435185	73448021	-	.	.	.	.	.	.	.	.	.	.	.	.	.	.	.	.	.	.	.	.
1758	2	SIN3A	NM_001145358	chr15	73448772	73531140	-	.	.	.	.	.	.	.	.	.	.	.	.	.	.	.	.	.	.	.	.

Table S2

tumor associated genes								HIV				MLV				MMTV				MMTV(SIN)				MMTV(SIN)arrest			
rank	score	gene symbol	RefSEq	chrom	txStart	txEnd	strand	integrant	chr	position	strand	integrant	chr	position	strand	integrant	chr	position	strand	integrant	chr	position	strand	integrant	chr	position	strand
5003	1	PTPN9	NM_002833	chr15	73546514	73658680	-	.	.	.	.	.	.	.	.	.	.	.	.	.	.	.	.	.	.	.	.
6223	0,5	CSPG4	NM_001897	chr15	73753717	73792244	-	.	.	.	.	.	.	.	.	.	.	.	.	.	.	.	.	.	.	.	.
3755	1	FBXO22	NR_037623	chr15	73983254	74014663	+	.	.	.	.	.	.	.	.	.	.	.	.	.	.	.	.	.	.	.	.
4976	1	PSTPIP1	NM_003978	chr15	75074519	75116726	+	.	.	.	.	.	.	.	.	.	.	.	.	.	.	.	.	.	.	.	.
7054	0,5	TSPAN3	NM_001168412	chr15	75123414	75150625	-	CL529400	chr15	75147025	-	.	.	.	.	.	.	.	.	.	.	.	.	.	.	.	.
6560	0,5	LINGO1	NM_032808	chr15	75692423	75711764	-	.	.	.	.	.	.	.	.	.	.	.	.	.	.	.	.	.	.	.	.
4108	1	IDH3A	NM_005530	chr15	76228773	76249939	+	.	.	.	.	.	.	.	.	.	.	.	.	.	.	.	.	.	.	.	.
772	2,5	ACSBG1	NM_001199377	chr15	76250241	76314104	-	.	.	.	.	.	.	.	.	.	.	.	.	.	.	.	.	.	.	.	.
1363	2	DNAJA4	NM_018602	chr15	76343541	76361593	+	.	.	.	.	.	.	.	.	.	.	.	.	.	.	.	.	.	.	.	.
2554	1,25	CRABP1	NM_004378	chr15	76419720	76427627	+	.	.	.	.	.	.	.	.	.	.	.	.	.	.	.	.	.	.	.	.
7245	0,25	CHRNA5	NM_000745	chr15	76644916	76674666	+	.	.	.	.	.	.	.	.	.	.	.	.	.	.	.	.	.	.	.	.
3359	1	CHRNA3	NM_000743	chr15	76674701	76700692	-	.	.	.	.	.	.	.	.	.	.	.	.	.	.	.	.	.	.	.	.
5992	0,5	ADAMTS7	NM_014272	chr15	76838599	76890828	-	.	.	.	.	.	.	.	.	.	.	.	.	.	.	.	.	.	.	.	.
6623	0,5	MORF4L1	NM_001265604	chr15	76952385	76977136	+	.	.	.	.	.	.	.	.	.	.	.	.	.	.	.	.	.	.	.	.
5895	0,75	CTSH	NM_004390	chr15	77001146	77024475	-	.	.	.	.	.	.	.	.	.	.	.	.	.	.	.	.	.	.	.	.
945	2,5	RASGRF1	NM_002891	chr15	77039343	77170270	-	.	.	.	.	.	.	.	.	.	.	.	.	.	.	.	.	.	.	.	.
4455	1	MTHFS	NR_037654	chr15	77922943	77976394	-	.	.	.	.	.	.	.	.	.	.	.	.	.	.	.	.	.	.	.	.
1878	1,75	BCL2A1	NM_004049	chr15	78040286	78050698	-	.	.	.	.	.	.	.	.	.	.	.	.	.	.	.	.	.	.	.	.
3715	1	FAH	NM_000137	chr15	78232287	78265979	+	.	.	.	.	.	.	.	.	.	.	.	.	.	.	.	.	.	.	.	.
1255	2	ARNT2	NM_014862	chr15	78483746	78677332	+	.	.	.	.	.	.	.	.	.	.	.	.	.	.	.	.	2326_201_1	chr15	78511613	-
6522	0,5	KIAA1199	NM_018689	chr15	78858766	79031054	+	.	.	.	.	.	.	.	.	.	.	.	.	.	.	.	.	.	.	.	.
7371	0,25	IL16	NM_172217	chr15	79304694	79392159	+	.	.	.	.	.	.	.	.	.	.	.	.	.	.	.	.	.	.	.	.
6209	0,5	CPEB1	NM_030594	chr15	81009005	81113783	-	.	.	.	.	.	.	.	.	.	.	.	.	.	.	.	.	.	.	.	.
2398	1,5	SH3GL3	NM_003027	chr15	81907094	82078497	+	.	.	.	.	.	.	.	.	.	.	.	.	.	.	.	.	.	.	.	.
522	3	ADAMTSL3	NM_207517	chr15	82113841	82499597	+	.	.	.	.	.	.	.	.	.	.	.	.	.	.	.	.	.	.	.	.
5938	0,75	NMB	NM_205858	chr15	82999363	83002806	-	.	.	.	.	.	.	.	.	.	.	.	.	.	.	.	.	.	.	.	.
2970	1	ALPK3	NM_020778	chr15	83160914	83217717	+	.	.	.	.	.	.	.	.	.	.	.	.	.	.	.	.	.	.	.	.
4738	1	PDE8A	NM_173454	chr15	83326208	83483376	+	CL800815	chr15	83409180	+	.	.	.	.	.	.	.	.	.	.	.	.	.	.	.	.
777	2,5	AKAP13	NM_006738	chr15	83724850	84093593	+	BH609728	chr15	84066375	-	.	.	.	.	.	.	.	.	.	.	.	.	.	.	.	.
777	2,5	AKAP13	NM_006738	chr15	83724850	84093593	+	CL529607	chr15	84045967	-	.	.	.	.	.	.	.	.	.	.	.	.	.	.	.	.
777	2,5	AKAP13	NM_006738	chr15	83724850	84093593	+	BH609727	chr15	83760954	+	.	.	.	.	.	.	.	.	.	.	.	.	.	.	.	.
6538	0,5	KLHL25	NM_022480	chr15	84103562	84139193	-	.	.	.	.	.	.	.	.	.	.	.	.	.	.	.	.	.	.	.	.
61	6,75	NTRK3	NM_001243101	chr15	86220991	86600966	-	.	.	.	.	.	.	.	.	.	.	.	.	.	.	.	.	.	.	.	.
3561	1	DET1	NM_017996	chr15	86856717	86890916	-	.	.	.	.	.	.	.	.	.	.	.	.	.	.	.	.	.	.	.	.
2874	1	ACAN	NM_013227	chr15	87147677	87219589	+	.	.	.	.	.	.	.	.	.	.	.	.	.	.	.	.	.	.	.	.
1646	2	POLG	NM_002693	chr15	87660539	87679030	-	.	.	.	.	.	.	.	.	.	.	.	.	.	.	.	.	.	.	.	.
994	2,25	ANPEP	NM_001150	chr15	88129129	88159076	-	.	.	.	.	.	.	.	.	.	.	.	.	.	.	.	.	.	.	.	.
594	3	IDH2	NM_002168	chr15	88428215	88446712	-	.	.	.	.	.	.	.	.	.	.	.	.	.	.	.	.	.	.	.	.
6180	0,5	CIB1	NM_006384	chr15	88574480	88578283	-	.	.	.	.	.	.	.	.	.	.	.	.	.	.	.	.	.	.	.	.
6372	0,5	GABARAPL3	NR_028287	chr15	88690766	88693683	-	.	.	.	.	.	.	.	.	.	.	.	.	.	.	.	.	.	.	.	.
477	3,25	IQGAP1	NM_003870	chr15	88732476	88846479	+	CL529498	chr15	88736498	+	AY516764	chr15	88757899	-	.	.	.	.	.	.	.	.	.	.	.	.
86	6	BLM	NM_000057	chr15	89061582	89159690	+	.	.	.	.	.	.	.	.	.	.	.	.	.	.	.	.	.	.	.	.
327	3,75	FURIN	NM_002569	chr15	89212888	89227691	+	.	.	.	.	.	.	.	.	.	.	.	.	.	.	.	.	.	.	.	.
211	4,25	FES	NM_002005	chr15	89228691	89240010	+	.	.	.	.	.	.	.	.	.	.	.	.	.	.	.	.	.	.	.	.
7078	0,5	UNC45A	NM_001039675	chr15	89274413	89298327	+	.	.	.	.	.	.	.	.	.	.	.	.	.	.	.	.	.	.	.	.
6765	0,5	PRC1	NM_003981	chr15	89310271	89338885	-	.	.	.	.	.	.	.	.	.	.	.	.	.	.	.	.	.	.	.	.
5415	1	SV2B	NM_001167580	chr15	89444185	89645543	+	.	.	.	.	.	.	.	.	.	.	.	.	.	.	.	.	.	.	.	.
6335	0,5	FAM174B	NM_207446	chr15	90961682	91000035	-	.	.	.	.	.	.	.	.	.	.	.	.	.	.	.	.	.	.	.	.
741	2,75	NR2F2	NM_021005	chr15	94675114	94684496	+	.	.	.	.	.	.	.	.	.	.	.	.	.	.	.	.	.	.	.	.
3046	1	ARRDC4	NM_183376	chr15	96304936	96318072	+	.	.	.	.	.	.	.	.	.	.	.	.	.	.	.	.	.	.	.	.
267	4	IGF1R	NM_000875	chr15	97010283	97325282	+	.	.	.	.	AY516163	chr15	97258592	-	.	.	.	.	.	.	.	.	3050_283_1	chr15	97133594	+

Table S2

tumor associated genes								HIV				MLV				MMTV				MMTV(SIN)				MMTV(SIN)arrest			
rank	score	gene symbol	RefSEq	chrom	txStart	txEnd	strand	integrant	chr	position	strand	integrant	chr	position	strand	integrant	chr	position	strand	integrant	chr	position	strand	integrant	chr	position	strand
267	4	IGF1R	NM_000875	chr15	97010283	97325282	+	.	.	.	.	AY516685	chr15	97258251	-	.	.	.	.	.	.	.	.	3050_283_1	chr15	97133594	+
4378	1	MEF2A	NM_001130927	chr15	97923655	98074152	+	BH609730	chr15	98018603	-	.	.	.	.	.	.	.	.	.	.	.	.	.	.	.	.
527	3	ALDH1A3	NM_000693	chr15	99237531	99274353	+	.	.	.	.	.	.	.	.	.	.	.	.	.	.	.	.	.	.	.	.
4309	1	LRRK1	NM_024652	chr15	99276982	99427840	+	.	.	.	.	AY516208	chr15	99316026	-	.	.	.	.	.	.	.	.	.	.	.	.
1601	2	PCSK6	NM_138321	chr15	99670747	99847710	-	.	.	.	.	.	.	.	.	.	.	.	.	.	.	.	.	.	.	.	.
1661	2	POLR3K	NM_016310	chr16	36978	43632	-	.	.	.	.	.	.	.	.	.	.	.	.	.	.	.	.	.	.	.	.
4428	1	MPG	NM_001015052	chr16	68168	75850	+	.	.	.	.	.	.	.	.	.	.	.	.	.	.	.	.	.	.	.	.
2180	1,5	HBA2	NM_000517	chr16	162845	163709	+	.	.	.	.	.	.	.	.	.	.	.	.	.	.	.	.	.	.	.	.
6813	0,5	RGS11	NM_003834	chr16	258310	265915	-	.	.	.	.	.	.	.	.	.	.	.	.	.	.	.	.	.	.	.	.
3028	1	ARHGDIG	NM_001176	chr16	270606	273004	+	.	.	.	.	.	.	.	.	.	.	.	.	.	.	.	.	.	.	.	.
6726	0,5	PDIA2	NM_006849	chr16	273118	277210	+	.	.	.	.	.	.	.	.	.	.	.	.	.	.	.	.	.	.	.	.
1272	2	AXIN1	NM_003502	chr16	277440	342677	-	CL800168	chr16	341056	+	.	.	.	.	.	.	.	.	.	.	.	.	.	.	.	.
4590	1	NME4	NM_005009	chr16	387192	390755	+	.	.	.	.	.	.	.	.	.	.	.	.	.	.	.	.	.	.	.	.
6953	0,5	SOLH	NM_005632	chr16	517856	544637	+	.	.	.	.	.	.	.	.	.	.	.	.	.	.	.	.	.	.	.	.
4801	1	PIGQ	NM_148920	chr16	559968	574137	+	.	.	.	.	.	.	.	.	.	.	.	.	.	.	.	.	.	.	.	.
6789	0,5	RAB40C	NM_021168	chr16	580173	619274	+	CL799664	chr16	594574	-	.	.	.	.	.	.	.	.	.	.	.	.	.	.	.	.
6789	0,5	RAB40C	NM_021168	chr16	580173	619274	+	CL800070	chr16	593788	+	.	.	.	.	.	.	.	.	.	.	.	.	.	.	.	.
5394	1	STUB1	NM_005861	chr16	670115	672769	+	.	.	.	.	.	.	.	.	.	.	.	.	.	.	.	.	.	.	.	.
2136	1,5	FBXL16	NM_153350	chr16	682500	695826	-	.	.	.	.	.	.	.	.	.	.	.	.	.	.	.	.	.	.	.	.
4008	1	HAGHL	NM_032304	chr16	717266	719716	+	.	.	.	.	.	.	.	.	.	.	.	.	.	.	.	.	.	.	.	.
2683	1,25	MSLN	NM_001177355	chr16	750766	758866	+	.	.	.	.	.	.	.	.	.	.	.	.	.	.	.	.	.	.	.	.
7567	0,25	SOX8	NM_014587	chr16	971808	976980	+	.	.	.	.	.	.	.	.	.	.	.	.	.	.	.	.	.	.	.	.
1283	2	CACNA1H	NM_001005407	chr16	1143241	1211773	+	.	.	.	.	.	.	.	.	.	.	.	.	.	.	.	.	.	.	.	.
979	2,5	UBE2I	NM_194261	chr16	1299154	1317020	+	CL799760	chr16	1306205	+	.	.	.	.	.	.	.	.	.	.	.	.	.	.	.	.
1522	2	MAPK8IP3	NM_001040439	chr16	1696221	1760319	+	CL800262	chr16	1745359	-	.	.	.	.	.	.	.	.	.	.	.	.	.	.	.	.
1522	2	MAPK8IP3	NM_001040439	chr16	1696221	1760319	+	CL800490	chr16	1709163	-	.	.	.	.	.	.	.	.	.	.	.	.	.	.	.	.
1522	2	MAPK8IP3	NM_001040439	chr16	1696221	1760319	+	CL800753	chr16	1709120	+	.	.	.	.	.	.	.	.	.	.	.	.	.	.	.	.
7447	0,25	NME3	NM_002513	chr16	1760321	1761711	-	.	.	.	.	.	.	.	.	.	.	.	.	.	.	.	.	.	.	.	.
4007	1	HAGH	NM_001040427	chr16	1799104	1817196	-	.	.	.	.	.	.	.	.	.	.	.	.	.	.	.	.	.	.	.	.
4536	1	NDUFB10	NM_004548	chr16	1949517	1951977	+	.	.	.	.	.	.	.	.	.	.	.	.	.	.	.	.	.	.	.	.
3876	1	GFER	NM_005262	chr16	1974150	1977751	+	.	.	.	.	.	.	.	.	.	.	.	.	.	.	.	.	.	.	.	.
6996	0,5	SYNGR3	NM_004209	chr16	1979946	1984277	+	.	.	.	.	.	.	.	.	.	.	.	.	.	.	.	.	.	.	.	.
4638	1	NTHL1	NM_002528	chr16	2029816	2037868	-	CL799545	chr16	2032577	-	.	.	.	.	.	.	.	.	.	.	.	.	.	.	.	.
85	6,25	TSC2	NM_000548	chr16	2037990	2078714	+	CL800124	chr16	2058083	-	.	.	.	.	.	.	.	.	.	.	.	.	.	.	.	.
6745	0,5	PKD1	NM_001009944	chr16	2078711	2125900	-	CL800172	chr16	2087862	+	.	.	.	.	.	.	.	.	.	.	.	.	.	.	.	.
6788	0,5	RAB26	NM_014353	chr16	2138651	2144142	+	.	.	.	.	.	.	.	.	.	.	.	.	.	.	.	.	.	.	.	.
5540	1	TRAF7	NM_032271	chr16	2145799	2168131	+	.	.	.	.	.	.	.	.	.	.	.	.	.	.	.	.	.	.	.	.
6131	0,5	CASKIN1	NM_020764	chr16	2167184	2186466	-	.	.	.	.	.	.	.	.	.	.	.	.	.	.	.	.	.	.	.	.
3639	1	E4F1	NM_004424	chr16	2213567	2225744	+	CL799721	chr16	2217734	-	.	.	.	.	.	.	.	.	.	.	.	.	2036_179_5	chr16	2221339	+
984	2,25	ABCA3	NM_001089	chr16	2265879	2330748	-	.	.	.	.	.	.	.	.	.	.	.	.	.	.	.	.	.	.	.	.
3289	1	CCNF	NM_001761	chr16	2419395	2448860	+	CL800081	chr16	2432932	-	.	.	.	.	.	.	.	.	.	.	.	.	.	.	.	.
3289	1	CCNF	NM_001761	chr16	2419395	2448860	+	CL800478	chr16	2431851	+	.	.	.	.	.	.	.	.	.	.	.	.	.	.	.	.
3085	1	ATP6V0C	NM_001198569	chr16	2503727	2510225	+	.	.	.	.	.	.	.	.	.	.	.	.	.	.	.	.	.	.	.	.
745	2,75	PDPK1	NM_001261816	chr16	2527965	2593192	+	BH609744	chr16	2563345	-	.	.	.	.	.	.	.	.	.	.	.	.	.	.	.	.
745	2,75	PDPK1	NM_001261816	chr16	2527965	2593192	+	CL529153	chr16	2536602	+	.	.	.	.	.	.	.	.	.	.	.	.	.	.	.	.
745	2,75	PDPK1	NM_001261816	chr16	2527965	2593192	+	CL799929	chr16	2530816	+	.	.	.	.	.	.	.	.	.	.	.	.	.	.	.	.
6972	0,5	SRRM2	NM_016333	chr16	2742330	2761414	+	.	.	.	.	.	.	.	.	.	.	.	.	.	.	.	.	.	.	.	.
4961	1	PRSS21	NM_006799	chr16	2807164	2811724	+	.	.	.	.	.	.	.	.	.	.	.	.	.	.	.	.	.	.	.	.
4820	1	PKMYT1	NM_001258450	chr16	2962792	2970541	-	.	.	.	.	.	.	.	.	.	.	.	.	.	.	.	.	.	.	.	.
2446	1,5	TNFRSF12A	NM_016639	chr16	3010313	3012384	+	.	.	.	.	.	.	.	.	.	.	.	.	.	.	.	.	.	.	.	.
7250	0,25	CLUAP1	NM_024793	chr16	3499894	3529051	+	.	.	.	.	.	.	.	.	.	.	.	.	.	.	.	.	.	.	.	.

Table S2

tumor associated genes								HIV				MLV				MMTV				MMTV(SIN)				MMTV(SIN)arrest			
rank	score	gene symbol	RefSEq	chrom	txStart	txEnd	strand	integrant	chr	position	strand	integrant	chr	position	strand	integrant	chr	position	strand	integrant	chr	position	strand	integrant	chr	position	strand
7046	0,5	TRAP1	NM_016292	chr16	3648038	3707599	-	.	.	.	.	.	.	.	.	.	.	.	.	.	.	.	.	.	.	.	.
48	7,25	CREBBP	NM_004380	chr16	3715056	3870122	-	BH609749	chr16	3825323	+	.	.	.	.	.	.	.	.	.	.	.	.	.	.	.	.
48	7,25	CREBBP	NM_004380	chr16	3715056	3870122	-	CL529545	chr16	3855425	+	.	.	.	.	.	.	.	.	.	.	.	.	.	.	.	.
166	4,5	ADCY9	NM_001116	chr16	3952650	4106187	-	.	.	.	.	.	.	.	.	.	.	.	.	.	.	.	.	.	.	.	.
5901	0,75	DNAJA3	NM_005147	chr16	4415806	4446776	+	.	.	.	.	AY516235	chr16	4416610	+	.	.	.	.	.	.	.	.	.	.	.	.
4061	1	HMOX2	NM_001127205	chr16	4485859	4500349	+	CL800716	chr16	4483818	+	.	.	.	.	.	.	.	.	.	.	.	.	.	.	.	.
5148	1	ROGDI	NR_046480	chr16	4786963	4792952	-	.	.	.	.	.	.	.	.	.	.	.	.	.	.	.	.	.	.	.	.
7075	0,5	UBN1	NM_001079514	chr16	4837912	4872364	+	.	.	.	.	.	.	.	.	.	.	.	.	.	.	.	.	.	.	.	.
2954	1	ALG1	NM_019109	chr16	5061810	5077381	+	.	.	.	.	.	.	.	.	.	.	.	.	.	.	.	.	.	.	.	.
1982	1,5	ABAT	NM_001127448	chr16	8722073	8785933	+	.	.	.	.	.	.	.	.	.	.	.	.	.	.	.	.	.	.	.	.
4852	1	PMM2	NM_000303	chr16	8799170	8850695	+	.	.	.	.	.	.	.	.	.	.	.	.	.	.	.	.	.	.	.	.
439	3,5	USP7	NM_003470	chr16	8893451	8964842	-	.	.	.	.	.	.	.	.	.	.	.	.	.	.	.	.	.	.	.	.
849	2,5	GRIN2A	NM_001134408	chr16	9764032	10183425	-	.	.	.	.	.	.	.	.	.	.	.	.	.	.	.	.	.	.	.	.
6036	0,5	ATF7IP2	NM_001256160	chr16	10387412	10484996	+	.	.	.	.	.	.	.	.	.	.	.	.	.	.	.	.	.	.	.	.
453	3,25	CIITA	NM_000246	chr16	10878555	10926341	+	.	.	.	.	.	.	.	.	.	.	.	.	.	.	.	.	.	.	.	.
193	4,5	SOCS1	NM_003745	chr16	11255774	11257540	-	.	.	.	.	.	.	.	.	.	.	.	.	.	.	.	.	.	.	.	.
5955	0,75	RSL1D1	NM_015659	chr16	11835555	11852943	-	.	.	.	.	.	.	.	.	.	.	.	.	.	.	.	.	.	.	.	.
3976	1	GSPT1	NM_002094	chr16	11869485	11917326	-	CL799666	chr16	11892923	+	.	.	.	.	.	.	.	.	.	.	.	.	.	.	.	.
1176	2,25	TNFRSF17	NM_001192	chr16	11966464	11969426	+	.	.	.	.	.	.	.	.	.	.	.	.	.	.	.	.	.	.	.	.
209	4,25	ERCC4	NM_005236	chr16	13921514	13953706	+	.	.	.	.	.	.	.	.	.	.	.	.	.	.	.	.	.	.	.	.
4405	1	MKL2	NM_014048	chr16	14072696	14268131	+	CL800505	chr16	14150974	+	.	.	.	.	.	.	.	.	.	.	.	.	.	.	.	.
4405	1	MKL2	NM_014048	chr16	14072696	14268131	+	BH609740	chr16	14124493	+	.	.	.	.	.	.	.	.	.	.	.	.	.	.	.	.
7187	0,25	BFAR	NM_016561	chr16	14634168	14670594	+	CL799543	chr16	14661455	-	.	.	.	.	.	.	.	.	.	.	.	.	.	.	.	.
4825	1	PLA2G10	NM_003561	chr16	14673905	14696027	-	.	.	.	.	.	.	.	.	.	.	.	.	.	.	.	.	.	.	.	.
4636	1	NTAN1	NM_001270767	chr16	15039210	15057437	-	.	.	.	.	.	.	.	.	.	.	.	.	.	.	.	.	.	.	.	.
5170	1	RRN3	NM_018427	chr16	15061379	15095659	-	.	.	.	.	.	.	.	.	.	.	.	.	.	.	.	.	.	.	.	.
4434	1	MPV17L	NM_173803	chr16	15397111	15411044	+	.	.	.	.	.	.	.	.	.	.	.	.	.	.	.	.	.	.	.	.
4517	1	NDE1	NM_017668	chr16	15651583	15727709	+	CL529156	chr16	15698657	-	.	.	.	.	.	.	.	.	.	.	.	.	.	.	.	.
899	2,5	MYH11	NM_022844	chr16	15704492	15858388	-	.	.	.	.	.	.	.	.	.	.	.	.	.	.	.	.	.	.	.	.
985	2,25	ABCC1	NM_004996	chr16	15950934	16144431	+	CL800178	chr16	16105964	+	.	.	.	.	.	.	.	.	.	.	.	.	.	.	.	.
5718	1	XYLT1	NM_022166	chr16	17103681	17472239	-	.	.	.	.	.	.	.	.	.	.	.	.	.	.	.	.	.	.	.	.
1765	2	SMG1	NM_015092	chr16	18723675	18845227	-	CL529157	chr16	18820472	-	.	.	.	.	.	.	.	.	.	.	.	.	.	.	.	.
1765	2	SMG1	NM_015092	chr16	18723675	18845227	-	BH609583	chr16	18829341	+	.	.	.	.	.	.	.	.	.	.	.	.	.	.	.	.
1765	2	SMG1	NM_015092	chr16	18723675	18845227	-	BH609731	chr16	18829341	+	.	.	.	.	.	.	.	.	.	.	.	.	.	.	.	.
1765	2	SMG1	NM_015092	chr16	18723675	18845227	-	BH609733	chr16	18791440	-	.	.	.	.	.	.	.	.	.	.	.	.	.	.	.	.
3426	1	COQ7	NM_001190983	chr16	18986739	18998918	+	.	.	.	.	.	.	.	.	.	.	.	.	.	.	.	.	.	.	.	.
6406	0,5	GPRC5B	NM_016235	chr16	19777793	19803652	-	.	.	.	.	.	.	.	.	.	.	.	.	.	.	.	.	.	.	.	.
2891	1	ACSM1	NM_052956	chr16	20542059	20610079	-	.	.	.	.	.	.	.	.	.	.	.	.	.	.	.	.	.	.	.	.
6566	0,5	LOC81691	NM_001144924	chr16	20725267	20768491	+	.	.	.	.	.	.	.	.	.	.	.	.	.	.	.	.	.	.	.	.
6585	0,5	LYRM1	NM_001128302	chr16	20819575	20843834	+	CL529158	chr16	20842445	-	.	.	.	.	.	.	.	.	.	.	.	.	.	.	.	.
2094	1,5	CRYM	NM_001888	chr16	21177339	21221905	-	.	.	.	.	.	.	.	.	.	.	.	.	.	.	.	.	.	.	.	.
1844	2	UQCRC2	NM_003366	chr16	21872109	21902169	+	CL799724	chr16	21895836	-	.	.	.	.	.	.	.	.	.	.	.	.	.	.	.	.
1844	2	UQCRC2	NM_003366	chr16	21872109	21902169	+	BH609737	chr16	21884279	-	.	.	.	.	.	.	.	.	.	.	.	.	.	.	.	.
3648	1	EEF2K	NM_013302	chr16	22125092	22207567	+	.	.	.	.	.	.	.	.	.	.	.	.	.	.	.	.	.	.	.	.
4871	1	POLR3E	NR_047581	chr16	22216196	22253926	+	.	.	.	.	.	.	.	.	.	.	.	.	.	.	.	.	.	.	.	.
3321	1	CDR2	NM_001802	chr16	22264757	22293439	-	.	.	.	.	.	.	.	.	.	.	.	.	.	.	.	.	.	.	.	.
1920	1,75	HS3ST2	NM_006043	chr16	22733360	22835160	+	.	.	.	.	.	.	.	.	.	.	.	.	.	.	.	.	.	.	.	.
3640	1	EARS2	NM_001083614	chr16	23440834	23476197	-	.	.	.	.	.	.	.	.	.	.	.	.	.	.	.	.	.	.	.	.
4533	1	NDUFAB1	NM_005003	chr16	23499835	23515140	-	.	.	.	.	.	.	.	.	.	.	.	.	.	.	.	.	.	.	.	.
635	3	PALB2	NM_024675	chr16	23521983	23560179	-	.	.	.	.	.	.	.	.	.	.	.	.	.	.	.	.	.	.	.	.
495	3,25	PLK1	NM_005030	chr16	23597701	23609189	+	.	.	.	.	.	.	.	.	.	.	.	.	.	.	.	.	.	.	.	.

Table S2

tumor associated genes								HIV				MLV				MMTV				MMTV(SIN)				MMTV(SIN)arrest			
rank	score	gene symbol	RefSEq	chrom	txStart	txEnd	strand	integrant	chr	position	strand	integrant	chr	position	strand	integrant	chr	position	strand	integrant	chr	position	strand	integrant	chr	position	strand
2073	1,5	CHP2	NM_022097	chr16	23673448	23677757	+	.	.	.	.	.	.	.	.	.	.	.	.	.	.	.	.	.	.	.	.
419	3,5	PRKCB	NM_002738	chr16	23754800	24139433	+	.	.	.	.	.	.	.	.	.	.	.	.	.	.	.	.	.	.	.	.
6117	0,5	CACNG3	NM_006539	chr16	24174374	24281238	+	.	.	.	.	.	.	.	.	.	.	.	.	.	.	.	.	.	.	.	.
6799	0,5	RBBP6	NM_018703	chr16	24458408	24491684	+	.	.	.	.	.	.	.	.	.	.	.	.	.	.	.	.	.	.	.	.
6919	0,5	SLC5A11	NM_001258413	chr16	24764684	24830450	+	.	.	.	.	.	.	.	.	.	.	.	.	.	.	.	.	.	.	.	.
4267	1	LCMT1	NM_001032391	chr16	25030547	25097052	+	AY517210.1	chr16	25091399	-	.	.	.	.	.	.	.	.	.	.	.	.	.	.	.	.
4083	1	HS3ST4	NM_006040	chr16	25610847	26056510	+	.	.	.	.	.	.	.	.	.	.	.	.	.	.	.	.	.	.	.	.
6477	0,5	IL4R	NM_001257997	chr16	27232730	27283600	+	.	.	.	.	.	.	.	.	.	.	.	.	.	.	.	.	.	.	.	.
1470	2	IL21R	NM_181078	chr16	27320983	27370864	+	.	.	.	.	.	.	.	.	.	.	.	.	.	.	.	.	.	.	.	.
4220	1	KIAA0556	NM_015202	chr16	27468968	27699193	+	.	.	.	.	.	.	.	.	.	.	.	.	.	.	.	.	.	.	.	.
7107	0,5	XPO6	NM_015171	chr16	28016816	28130691	-	CL529711	chr16	28064186	-	.	.	.	.	.	.	.	.	.	.	.	.	.	.	.	.
7107	0,5	XPO6	NM_015171	chr16	28016816	28130691	-	AY516951.1	chr16	28076864	+	.	.	.	.	.	.	.	.	.	.	.	.	.	.	.	.
7107	0,5	XPO6	NM_015171	chr16	28016816	28130691	-	CL800748	chr16	28078358	+	.	.	.	.	.	.	.	.	.	.	.	.	.	.	.	.
7107	0,5	XPO6	NM_015171	chr16	28016816	28130691	-	CL529712	chr16	28083216	-	.	.	.	.	.	.	.	.	.	.	.	.	.	.	.	.
5187	1	SBK1	NM_001024401	chr16	28211340	28242671	+	.	.	.	.	.	.	.	.	.	.	.	.	.	.	.	.	.	.	.	.
7452	0,25	NUPR1	NM_001042483	chr16	28456162	28457996	-	.	.	.	.	.	.	.	.	.	.	.	.	.	.	.	.	.	.	.	.
5403	1	SULT1A2	NM_177528	chr16	28510764	28515302	-	.	.	.	.	.	.	.	.	.	.	.	.	.	.	.	.	.	.	.	.
5402	1	SULT1A1	NM_177536	chr16	28524408	28542408	-	.	.	.	.	.	.	.	.	.	.	.	.	.	.	.	.	.	.	.	.
804	2,5	CD19	NM_001770	chr16	28850760	28858169	+	.	.	.	.	.	.	.	.	.	.	.	.	.	.	.	.	.	.	.	.
5340	1	SPNS1	NM_032038	chr16	28893596	28903370	+	.	.	.	.	.	.	.	.	.	.	.	.	.	.	.	.	.	.	.	.
6962	0,5	SPN	NM_001030288	chr16	29581771	29589324	+	.	.	.	.	.	.	.	.	.	.	.	.	.	.	.	.	.	.	.	.
5017	1	QPRT	NM_014298	chr16	29597941	29616815	+	.	.	.	.	.	.	.	.	.	.	.	.	.	.	.	.	.	.	.	.
1941	1,75	MVP	NM_017458	chr16	29739215	29766861	+	.	.	.	.	AY516211	chr16	29743247	+	.	.	.	.	.	.	.	.	.	.	.	.
3313	1	CDIPT	NM_006319	chr16	29777178	29782079	-	.	.	.	.	.	.	.	.	.	.	.	.	.	.	.	.	.	.	.	.
7541	0,25	SEZ6L2	NM_001114100	chr16	29789980	29818086	-	.	.	.	.	.	.	.	.	.	.	.	.	.	.	.	.	.	.	.	.
6033	0,5	ASPHD1	NM_181718	chr16	29819647	29824878	+	.	.	.	.	.	.	.	.	.	.	.	.	.	.	.	.	.	.	.	.
5430	1	TAOK2	NM_016151	chr16	29892688	29907227	+	AY517343.1	chr16	29910974	+	.	.	.	.	.	.	.	.	.	.	.	.	.	.	.	.
5430	1	TAOK2	NM_016151	chr16	29892688	29907227	+	CL800561	chr16	29902181	-	.	.	.	.	.	.	.	.	.	.	.	.	.	.	.	.
6279	0,5	DOC2A	NM_003586	chr16	29924335	29929902	-	.	.	.	.	.	.	.	.	.	.	.	.	.	.	.	.	.	.	.	.
988	2,25	ALDOA	NM_001243177	chr16	29984494	29989242	+	.	.	.	.	.	.	.	.	.	.	.	.	.	.	.	.	.	.	.	.
1676	2	PPP4C	NM_002720	chr16	29994884	30004196	+	.	.	.	.	.	.	.	.	.	.	.	.	.	.	.	.	.	.	.	.
275	4	MAPK3	NM_002746	chr16	30032926	30042131	-	.	.	.	.	.	.	.	.	.	.	.	.	.	.	.	.	.	.	.	.
5404	1	SULT1A3	NM_177552	chr16	30118049	30123151	+	.	.	.	.	.	.	.	.	.	.	.	.	.	.	.	.	.	.	.	.
5405	1	SULT1A4	NM_001017390	chr16	30118049	30123151	+	.	.	.	.	.	.	.	.	.	.	.	.	.	.	.	.	.	.	.	.
1162	2,25	SEPHS2	NM_012248	chr16	30362452	30364725	-	.	.	.	.	.	.	.	.	.	.	.	.	.	.	.	.	.	.	.	.
5926	0,75	ITGAL	NM_001114380	chr16	30391483	30442007	+	AY516919.1	chr16	30401915	+	.	.	.	.	.	.	.	.	.	.	.	.	.	.	.	.
5926	0,75	ITGAL	NM_001114380	chr16	30391483	30442007	+	CL800159	chr16	30396020	-	.	.	.	.	.	.	.	.	.	.	.	.	.	.	.	.
5348	1	SRCAP	NM_006662	chr16	30617962	30658951	+	.	.	.	.	.	.	.	.	.	.	.	.	.	.	.	.	.	.	.	.
4788	1	PHKG2	NM_001172432	chr16	30667120	30680001	+	.	.	.	.	.	.	.	.	.	.	.	.	.	.	.	.	.	.	.	.
7138	0,5	ZNF629	NM_001080417	chr16	30697270	30706024	-	.	.	.	.	.	.	.	.	.	.	.	.	.	.	.	.	.	.	.	.
1749	2	SETD1A	NM_014712	chr16	30876115	30903482	+	CL529160	chr16	30895040	+	.	.	.	.	.	.	.	.	.	.	.	.	.	.	.	.
4093	1	HSD3B7	NM_025193	chr16	30904019	30907974	+	.	.	.	.	.	.	.	.	.	.	.	.	.	.	.	.	.	.	.	.
1873	2	ZNF668	NM_024706	chr16	30979664	30993142	-	.	.	.	.	.	.	.	.	.	.	.	.	.	.	.	.	.	.	.	.
5840	1	ZNF646	NM_014699	chr16	30993243	31002334	+	.	.	.	.	.	.	.	.	.	.	.	.	.	.	.	.	.	.	.	.
5675	1	VKORC1	NM_206824	chr16	31009675	31013777	-	.	.	.	.	.	.	.	.	.	.	.	.	.	.	.	.	.	.	.	.
4962	1	PRSS8	NM_002773	chr16	31050254	31054652	-	.	.	.	.	.	.	.	.	.	.	.	.	.	.	.	.	.	.	.	.
1412	2	FUS	NM_001170634	chr16	31098931	31113693	+	.	.	.	.	.	.	.	.	.	.	.	.	.	.	.	.	.	.	.	.
2737	1,25	PYCARD	NM_013258	chr16	31120307	31121598	-	.	.	.	.	.	.	.	.	.	.	.	.	.	.	.	.	.	.	.	.
6486	0,5	ITGAM	NM_001145808	chr16	31178788	31251714	+	.	.	.	.	.	.	.	.	.	.	.	.	.	.	.	.	.	.	.	.
3437	1	COX6A2	NM_005205	chr16	31346552	31347250	-	.	.	.	.	.	.	.	.	.	.	.	.	.	.	.	.	.	.	.	.
7559	0,25	SLC5A2	NM_003041	chr16	31401939	31409592	+	.	.	.	.	.	.	.	.	.	.	.	.	.	.	.	.	.	.	.	.

Table S2

tumor associated genes								HIV				MLV				MMTV				MMTV(SIN)				MMTV(SIN)arrest			
rank	score	gene symbol	RefSEq	chrom	txStart	txEnd	strand	integrant	chr	position	strand	integrant	chr	position	strand	integrant	chr	position	strand	integrant	chr	position	strand	integrant	chr	position	strand
2690	1,25	MYLK3	NM_182493	chr16	45293694	45339722	-	.	.	.	.	.	.	.	.	.	.	.	.	.	.	.	.	.	.	.	.
3957	1	GPT2	NM_001142466	chr16	45476602	45522702	+	.	.	.	.	.	.	.	.	.	.	.	.	.	.	.	.	.	.	.	.
2278	1,5	NETO2	NM_001201477	chr16	45672931	45735437	-	.	.	.	.	.	.	.	.	.	.	.	.	.	.	.	.	.	.	.	.
4786	1	PHKB	NM_001031835	chr16	46052710	46292935	+	CL529724	chr16	46069226	-	.	.	.	.	.	.	.	.	.	.	.	.	.	.	.	.
5980	0,5	ABCC12	NM_033226	chr16	46674384	46738182	-	.	.	.	.	.	.	.	.	.	.	.	.	.	.	.	.	.	.	.	.
2855	1	ABCC11	NM_145186	chr16	46758322	46826589	-	.	.	.	.	.	.	.	.	.	.	.	.	.	.	.	.	.	.	.	.
5239	1	SIAH1	NM_003031	chr16	46951954	46976730	-	.	.	.	.	.	.	.	.	.	.	.	.	.	.	.	.	.	.	.	.
1221	2	ADCY7	NM_001114	chr16	48879323	48909544	+	.	.	.	.	.	.	.	.	.	.	.	.	.	.	.	.	.	.	.	.
1946	1,75	NOD2	NM_022162	chr16	49288550	49324488	+	.	.	.	.	.	.	.	.	.	.	.	.	.	.	.	.	.	.	.	.
366	3,5	CYLD	NM_001042355	chr16	49333461	49393347	+	.	.	.	.	.	.	.	.	.	.	.	.	.	.	.	.	.	.	.	.
5176	1	SALL1	NM_001127892	chr16	49727386	49742009	-	.	.	.	.	.	.	.	.	.	.	.	.	.	.	.	.	.	.	.	.
2807	1,25	TOX3	NM_001146188	chr16	51029418	51139215	-	.	.	.	.	.	.	.	.	.	.	.	.	.	.	.	.	.	.	.	.
1308	2	CHD9	NM_025134	chr16	51646445	51918915	+	CL529163	chr16	51794486	+	.	.	.	.	_394_1_239	chr16	51695071	+	.	.	.	.	.	.	.	.
1719	2	RBL2	NM_005611	chr16	52025851	52083061	+	.	.	.	.	.	.	.	.	.	.	.	.	.	.	.	.	.	.	.	.
6484	0,5	IRX3	NM_024336	chr16	52874712	52877879	-	.	.	.	.	.	.	.	.	.	.	.	.	.	.	.	.	.	.	.	.
4168	1	IRX5	NM_005853	chr16	53522611	53525896	+	.	.	.	.	.	.	.	.	.	.	.	.	.	.	.	.	.	.	.	.
4169	1	IRX6	NM_024335	chr16	53915971	53922173	+	.	.	.	.	.	.	.	.	.	.	.	.	.	.	.	.	.	.	.	.
624	3	MMP2	NM_001127891	chr16	54072974	54098087	+	.	.	.	.	.	.	.	.	.	.	.	.	.	.	.	.	.	.	.	.
3343	1	CES1	NM_001025195	chr16	54394264	54424576	-	.	.	.	.	.	.	.	.	.	.	.	.	.	.	.	.	.	.	.	.
2164	1,5	GNAO1	NM_138736	chr16	54782751	54939612	+	.	.	.	.	.	.	.	.	.	.	.	.	.	.	.	.	.	.	.	.
781	2,5	AMFR	NM_001144	chr16	54952864	55016951	-	AY517152.1	chr16	54996705	-	.	.	.	.	.	.	.	.	.	.	.	.	.	.	.	.
781	2,5	AMFR	NM_001144	chr16	54952864	55016951	-	AY517149.1	chr16	54996705	-	.	.	.	.	.	.	.	.	.	.	.	.	.	.	.	.
6689	0,5	NUDT21	NM_007006	chr16	55020548	55042762	-	.	.	.	.	.	.	.	.	.	.	.	.	.	.	.	.	.	.	.	.
6634	0,5	MT3	NM_005954	chr16	55180767	55182501	+	.	.	.	.	.	.	.	.	.	.	.	.	.	.	.	.	.	.	.	.
4450	1	MT2A	NM_005953	chr16	55199978	55200910	+	.	.	.	.	.	.	.	.	.	.	.	.	.	.	.	.	.	.	.	.
6633	0,5	MT1M	NM_176870	chr16	55224034	55225399	+	.	.	.	.	.	.	.	.	.	.	.	.	.	.	.	.	.	.	.	.
4448	1	MT1G	NM_005950	chr16	55258153	55259478	-	.	.	.	.	.	.	.	.	.	.	.	.	.	.	.	.	.	.	.	.
4449	1	MT1H	NM_005951	chr16	55261226	55262542	+	.	.	.	.	.	.	.	.	.	.	.	.	.	.	.	.	.	.	.	.
6695	0,5	NUP93	NM_001242795	chr16	55373204	55436362	+	.	.	.	.	.	.	.	.	.	.	.	.	.	.	.	.	.	.	.	.
7228	0,25	CCL22	NM_002990	chr16	55950195	55957603	+	.	.	.	.	.	.	.	.	.	.	.	.	.	.	.	.	.	.	.	.
7262	0,25	CX3CL1	NM_002996	chr16	55963914	55976457	+	.	.	.	.	.	.	.	.	.	.	.	.	.	.	.	.	.	.	.	.
1651	2	POLR2C	NM_032940	chr16	56054051	56063422	+	CL800522	chr16	56057885	-	.	.	.	.	.	.	.	.	.	.	.	.	.	.	.	.
6399	0,5	GPR56	NM_001145771	chr16	56219919	56256445	+	.	.	.	.	.	.	.	.	.	.	.	.	.	.	.	.	.	.	.	.
1540	2	MMP15	NM_002428	chr16	56616782	56638305	+	.	.	.	.	.	.	.	.	.	.	.	.	.	.	.	.	.	.	.	.
718	2,75	CSNK2A2	NM_001896	chr16	56749312	56789283	-	.	.	.	.	.	.	.	.	.	.	.	.	.	.	.	.	.	.	.	.
6656	0,5	NDRG4	NM_001242833	chr16	57091547	57105024	+	.	.	.	.	.	.	.	.	.	.	.	.	.	.	.	.	.	.	.	.
3935	1	GOT2	NM_002080	chr16	57298535	57325747	-	.	.	.	.	.	.	.	.	.	.	.	.	.	.	.	.	.	.	.	.
6164	0,5	CDH8	NM_001796	chr16	60243415	60628240	-	.	.	.	.	.	.	.	.	.	.	.	.	.	.	.	.	.	.	.	.
1304	2	CDH11	NM_001797	chr16	63538183	63713420	-	.	.	.	.	.	.	.	.	.	.	.	.	.	.	.	.	.	.	.	.
3311	1	CDH5	NM_001795	chr16	64958025	64996190	+	.	.	.	.	.	.	.	.	.	.	.	.	.	.	.	.	.	.	.	.
1811	2	TK2	NM_004614	chr16	65099406	65141816	-	.	.	.	.	.	.	.	.	.	.	.	.	.	.	.	.	.	.	.	.
5893	0,75	CMTM3	NM_144601	chr16	65195728	65205296	+	.	.	.	.	.	.	.	.	.	.	.	.	.	.	.	.	.	.	.	.
2527	1,25	CA7	NM_001014435	chr16	65436341	65445550	+	.	.	.	.	.	.	.	.	.	.	.	.	.	.	.	.	.	.	.	.
1734	2	RRAD	NM_001128850	chr16	65513082	65516940	-	.	.	.	.	.	.	.	.	.	.	.	.	.	.	.	.	.	.	.	.
2547	1,25	CES3	NM_001185177	chr16	65552632	65566553	+	.	.	.	.	.	.	.	.	.	.	.	.	.	.	.	.	.	.	.	.
1291	2	CBFB	NM_001755	chr16	65620550	65692459	+	AY517062.1	chr16	65658378	-	.	.	.	.	.	.	.	.	.	.	.	.	.	.	.	.
1291	2	CBFB	NM_001755	chr16	65620550	65692459	+	CL799604	chr16	65680239	-	.	.	.	.	.	.	.	.	.	.	.	.	.	.	.	.
1291	2	CBFB	NM_001755	chr16	65620550	65692459	+	BH609751	chr16	65622829	+	.	.	.	.	.	.	.	.	.	.	.	.	.	.	.	.
1291	2	CBFB	NM_001755	chr16	65620550	65692459	+	CL529504	chr16	65630440	-	.	.	.	.	.	.	.	.	.	.	.	.	.	.	.	.
2449	1,5	TRADD	NM_003789	chr16	65745589	65751313	-	.	.	.	.	.	.	.	.	.	.	.	.	.	.	.	.	.	.	.	.
722	2,75	E2F4	NM_001950	chr16	65783568	65790322	+	BH609752	chr16	65789251	-	.	.	.	.	.	.	.	.	.	.	.	.	.	.	.	.

Table S2

tumor associated genes								HIV				MLV				MMTV				MMTV(SIN)				MMTV(SIN)arrest			
rank	score	gene symbol	RefSEq	chrom	txStart	txEnd	strand	integrant	chr	position	strand	integrant	chr	position	strand	integrant	chr	position	strand	integrant	chr	position	strand	integrant	chr	position	strand
6354	0,5	FHOD1	NM_013241	chr16	65820792	65838926	-	.	.	.	.	.	.	.	.	.	.	.	.	.	.	.	.	.	.	.	.
7044	0,5	TPPP3	NM_015964	chr16	65981212	65984922	-	.	.	.	.	.	.	.	.	.	.	.	.	.	.	.	.	.	.	.	.
4087	1	HSD11B2	NM_000196	chr16	66022536	66028955	+	.	.	.	.	.	.	.	.	.	.	.	.	.	.	.	.	.	.	.	.
3086	1	ATP6V0D1	NM_004691	chr16	66029417	66072590	-	.	.	.	.	.	.	.	.	.	.	.	.	.	.	.	.	.	.	.	.
7259	0,25	CTCF	NM_001191022	chr16	66153810	66230589	+	.	.	.	.	.	.	.	.	.	.	.	.	.	.	.	.	.	.	.	.
5129	1	RLTPR	NM_001013838	chr16	66236530	66248973	+	.	.	.	.	.	.	.	.	.	.	.	.	.	.	.	.	.	.	.	.
4700	1	PARD6A	NM_016948	chr16	66252351	66254182	+	.	.	.	.	.	.	.	.	.	.	.	.	.	.	.	.	.	.	.	.
3339	1	CENPT	NM_025082	chr16	66419560	66438862	-	.	.	.	.	.	.	.	.	.	.	.	.	.	.	.	.	.	.	.	.
7453	0,25	NUTF2	NM_005796	chr16	66438319	66462720	+	.	.	.	.	.	.	.	.	.	.	.	.	.	.	.	.	.	.	.	.
4968	1	PSKH1	NM_006742	chr16	66484675	66521082	+	AY516988.1	chr16	66495024	-	.	.	.	.	.	.	.	.	.	.	.	.	.	.	.	.
4265	1	LCAT	NM_000229	chr16	66531287	66535516	-	.	.	.	.	.	.	.	.	.	.	.	.	.	.	.	.	.	.	.	.
6898	0,5	SLC12A4	NM_001145962	chr16	66534877	66560098	-	.	.	.	.	.	.	.	.	.	.	.	.	.	.	.	.	.	.	.	.
7293	0,25	DUS2L	NM_017803	chr16	66614704	66670685	+	.	.	.	.	.	.	.	.	.	.	.	.	.	.	.	.	.	.	.	.
2279	1,5	NFATC3	NM_173163	chr16	66676769	66820663	+	AY517224.1	chr16	66723707	+	.	.	.	.	.	.	.	.	.	.	.	.	.	.	.	.
2279	1,5	NFATC3	NM_173163	chr16	66676769	66820663	+	CL800026	chr16	66770052	+	.	.	.	.	.	.	.	.	.	.	.	.	.	.	.	.
2279	1,5	NFATC3	NM_173163	chr16	66676769	66820663	+	BH609754	chr16	66709959	+	.	.	.	.	.	.	.	.	.	.	.	.	.	.	.	.
2279	1,5	NFATC3	NM_173163	chr16	66676769	66820663	+	BH609755	chr16	66727042	+	.	.	.	.	.	.	.	.	.	.	.	.	.	.	.	.
2279	1,5	NFATC3	NM_173163	chr16	66676769	66820663	+	CL799608	chr16	66726513	+	.	.	.	.	.	.	.	.	.	.	.	.	.	.	.	.
2324	1,5	PLA2G15	NM_012320	chr16	66836747	66852462	+	.	.	.	.	.	.	.	.	.	.	.	.	.	.	.	.	.	.	.	.
1692	2	PRMT7	NM_019023	chr16	66902377	66948670	+	.	.	.	.	.	.	.	.	.	.	.	.	.	.	.	.	.	.	.	.
6939	0,5	SMPD3	NM_018667	chr16	66949730	67039910	-	BH609756	chr16	66998892	+	.	.	.	.	.	.	.	.	.	.	.	.	.	.	.	.
2541	1,25	CDH3	NM_001793	chr16	67235651	67290458	+	.	.	.	.	.	.	.	.	.	.	.	.	.	.	.	.	.	.	.	.
39	7,75	CDH1	NM_004360	chr16	67328695	67426945	+	.	.	.	.	.	.	.	.	.	.	.	.	.	.	.	.	.	.	.	.
4018	1	HAS3	NM_138612	chr16	67697629	67710120	+	.	.	.	.	.	.	.	.	.	.	.	.	.	.	.	.	.	.	.	.
3372	1	CIRH1A	NM_032830	chr16	67723999	67760438	+	.	.	.	.	.	.	.	.	.	.	.	.	.	.	.	.	.	.	.	.
6944	0,5	SNTB2	NM_006750	chr16	67778550	67900456	+	CL529494	chr16	67785577	-	.	.	.	.	.	.	.	.	.	.	.	.	.	.	.	.
6944	0,5	SNTB2	NM_006750	chr16	67778550	67900456	+	BH609757	chr16	67847454	+	.	.	.	.	.	.	.	.	.	.	.	.	.	.	.	.
2796	1,25	TERF2	NM_005652	chr16	67946964	67977375	-	.	.	.	.	.	.	.	.	.	.	.	.	.	.	.	.	.	.	.	.
4603	1	NQO1	NM_000903	chr16	68300804	68318034	-	BH609759	chr16	68317584	-	.	.	.	.	.	.	.	.	.	.	.	.	.	.	.	.
5710	1	WWP2	NM_001270455	chr16	68353687	68501809	+	BH609760	chr16	68413794	-	.	.	.	.	.	.	.	.	.	.	.	.	.	.	.	.
2845	1	AARS	NM_001605	chr16	68843797	68880913	-	.	.	.	.	.	.	.	.	.	.	.	.	.	.	.	.	.	.	.	.
3547	1	DDX19A	NM_018332	chr16	68938324	68964782	+	.	.	.	.	.	.	.	.	.	.	.	.	.	.	.	.	.	.	.	.
5358	1	ST3GAL2	NM_006927	chr16	68970838	69030492	-	.	.	.	.	.	.	.	.	.	.	.	.	.	.	.	.	.	.	.	.
3815	1	FUK	NM_145059	chr16	69045998	69071678	+	.	.	.	.	AY516533	chr16	69068663	-	.	.	.	.	.	.	.	.	.	.	.	.
6878	0,5	SF3B3	NM_012426	chr16	69115191	69169072	+	.	.	.	.	AY516149	chr16	69115731	-	.	.	.	.	.	.	.	.	.	.	.	.
6476	0,5	IL34	NM_001172771	chr16	69171298	69252086	+	.	.	.	.	.	.	.	.	.	.	.	.	.	.	.	.	.	.	.	.
6120	0,5	CALB2	NR_027910	chr16	69950116	69981843	+	.	.	.	.	.	.	.	.	.	.	.	.	.	.	.	.	.	.	.	.
3364	1	CHST4	NM_001166395	chr16	70117654	70129994	+	.	.	.	.	.	.	.	.	.	.	.	.	.	.	.	.	.	.	.	.
5433	1	TAT	NM_000353	chr16	70158254	70168499	-	.	.	.	.	.	.	.	.	.	.	.	.	.	.	.	.	.	.	.	.
7142	0,5	ZNF821	NM_001201552	chr16	70451083	70475594	-	.	.	.	.	.	.	.	.	.	.	.	.	.	.	.	.	.	.	.	.
3574	1	DHODH	NM_001361	chr16	70600143	70616817	+	.	.	.	.	.	.	.	.	.	.	.	.	.	.	.	.	.	.	.	.
5919	0,75	HP	NM_005143	chr16	70646008	70652456	+	.	.	.	.	.	.	.	.	.	.	.	.	.	.	.	.	.	.	.	.
4077	1	HPR	NM_020995	chr16	70654625	70668646	+	.	.	.	.	.	.	.	.	.	.	.	.	.	.	.	.	.	.	.	.
846	2,5	GLG1	NM_001145667	chr16	73038826	73198543	-	CL529167	chr16	73076927	+	.	.	.	.	.	.	.	.	.	.	.	.	.	.	.	.
4409	1	MLKL	NM_152649	chr16	73263253	73292290	-	.	.	.	.	.	.	.	.	.	.	.	.	.	.	.	.	.	.	.	.
3711	1	FA2H	NM_024306	chr16	73304356	73366230	-	.	.	.	.	.	.	.	.	.	.	.	.	.	.	.	.	.	.	.	.
2230	1,5	LDHD	NM_194436	chr16	73703258	73708171	-	.	.	.	.	.	.	.	.	.	.	.	.	.	.	.	.	.	.	.	.
358	3,5	BCAR1	NM_001170716	chr16	73820428	73858208	-	.	.	.	.	.	.	.	.	.	.	.	.	.	.	.	.	.	.	.	.
3365	1	CHST6	NM_021615	chr16	74064522	74086427	-	AY517286.1	chr16	74075111	-	.	.	.	.	.	.	.	.	.	.	.	.	.	.	.	.
6371	0,5	GABARAPL2	NM_007285	chr16	74157749	74169280	+	.	.	.	.	.	.	.	.	.	.	.	.	.	.	.	.	.	.	.	.
4195	1	KARS	NM_001130089	chr16	74219122	74239086	-	.	.	.	.	.	.	.	.	.	.	.	.	.	.	.	.	.	.	.	.

Table S2

tumor associated genes								HIV				MLV				MMTV				MMTV(SIN)				MMTV(SIN)arrest			
rank	score	gene symbol	RefSEq	chrom	txStart	txEnd	strand	integrant	chr	position	strand	integrant	chr	position	strand	integrant	chr	position	strand	integrant	chr	position	strand	integrant	chr	position	strand
6198	0,5	CNTNAP4	NM_033401	chr16	74868676	75150636	+	.	.	.	.	.	.	.	.	.	.	.	.	.	.	.	.	.	.	.	.
2902	1	ADAMTS18	NM_199355	chr16	75873525	76026512	-	.	.	.	.	.	.	.	.	.	.	.	.	.	.	.	.	.	.	.	.
440	3,5	WWOX	NM_130791	chr16	76690827	76870094	+	CL529169	chr16	76721206	+	.	.	.	.	.	.	.	.	67_1_2189_8	chr16	76760157	-	.	.	.	.
440	3,5	WWOX	NM_130791	chr16	76690827	76870094	+	CL529694	chr16	77490195	-	.	.	.	.	.	.	.	.	67_1_2189_8	chr16	76760157	-	.	.	.	.
1098	2,25	MAF	NM_005360	chr16	78185245	78192123	-	.	.	.	.	.	.	.	.	.	.	.	.	.	.	.	.	.	.	.	.
3335	1	CENPN	NM_001270474	chr16	79597603	79622591	+	.	.	.	.	AY515875	chr16	79617510	+	.	.	.	.	.	.	.	.	.	.	.	.
3137	1	BCMO1	NM_017429	chr16	79829796	79882248	+	.	.	.	.	.	.	.	.	.	.	.	.	.	.	.	.	.	.	.	.
109	5,5	PLCG2	NM_002661	chr16	80370399	80549400	+	.	.	.	.	.	.	.	.	.	.	.	.	.	.	.	.	1418_145_4	chr16	80538557	-
591	3	HSD17B2	NM_002153	chr16	80626358	80689640	+	.	.	.	.	.	.	.	.	.	.	.	.	.	.	.	.	.	.	.	.
714	2,75	CDH13	NM_001220488	chr16	81217899	82387716	+	AY516948.1	chr16	81958147	+	.	.	.	.	.	.	.	.	.	.	.	.	.	.	.	.
4412	1	MLYCD	NM_012213	chr16	82490230	82507288	+	.	.	.	.	.	.	.	.	.	.	.	.	.	.	.	.	.	.	.	.
7459	0,25	OSGIN1	NM_182981	chr16	82544327	82557438	+	.	.	.	.	.	.	.	.	.	.	.	.	.	.	.	.	.	.	.	.
6658	0,5	NECAB2	NM_019065	chr16	82559737	82593880	+	.	.	.	.	.	.	.	.	.	.	.	.	.	.	.	.	.	.	.	.
6598	0,5	MBTPS1	NM_003791	chr16	82644869	82708018	-	.	.	.	.	.	.	.	.	.	.	.	.	.	.	.	.	.	.	.	.
5990	0,5	ADAD2	NM_139174	chr16	82782223	82788273	+	.	.	.	.	.	.	.	.	.	.	.	.	.	.	.	.	.	.	.	.
4219	1	KIAA0513	NM_014732	chr16	83618910	83685329	+	.	.	.	.	.	.	.	.	.	.	.	.	.	.	.	.	.	.	.	.
4214	1	KIAA0182	NM_001134473	chr16	84202529	84267313	+	CL800471	chr16	84264696	-	.	.	.	.	.	.	.	.	.	.	.	.	.	.	.	.
1322	2	COX4I1	NM_001861	chr16	84390673	84398108	+	.	.	.	.	.	.	.	.	.	.	.	.	.	.	.	.	.	.	.	.
3795	1	FOXC2	NM_005251	chr16	85158357	85160038	+	.	.	.	.	.	.	.	.	.	.	.	.	.	.	.	.	.	.	.	.
1166	2,25	SLC7A5	NM_003486	chr16	86421129	86460601	-	.	.	.	.	.	.	.	.	.	.	.	.	.	.	.	.	.	.	.	.
3253	1	CA5A	NM_001739	chr16	86479125	86527613	-	.	.	.	.	.	.	.	.	.	.	.	.	.	.	.	.	.	.	.	.
4464	1	MVD	NM_002461	chr16	87245848	87256996	-	.	.	.	.	.	.	.	.	.	.	.	.	.	.	.	.	.	.	.	.
5302	1	SNAI3	NM_178310	chr16	87271590	87280383	-	.	.	.	.	.	.	.	.	.	.	.	.	.	.	.	.	.	.	.	.
3012	1	APRT	NM_000485	chr16	87403377	87405843	-	.	.	.	.	.	.	.	.	.	.	.	.	.	.	.	.	.	.	.	.
1418	2	GALNS	NM_000512	chr16	87407642	87450875	-	.	.	.	.	.	.	.	.	.	.	.	.	.	.	.	.	.	.	.	.
240	4	CBFA2T3	NM_005187	chr16	87468763	87571005	-	.	.	.	.	.	.	.	.	.	.	.	.	.	.	.	.	.	.	.	.
6012	0,5	ANKRD11	NR_045839	chr16	87880566	88084470	-	CL799768	chr16	87879933	+	.	.	.	.	.	.	.	.	.	.	.	.	.	.	.	.
6012	0,5	ANKRD11	NR_045839	chr16	87880566	88084470	-	CL800477	chr16	88040035	+	.	.	.	.	.	.	.	.	.	.	.	.	.	.	.	.
6012	0,5	ANKRD11	NR_045839	chr16	87880566	88084470	-	CL800435	chr16	87934526	-	.	.	.	.	.	.	.	.	.	.	.	.	.	.	.	.
6212	0,5	CPNE7	NM_153636	chr16	88169676	88191155	+	AY517390.1	chr16	88186912	+	.	.	.	.	.	.	.	.	.	.	.	.	.	.	.	.
2542	1,25	CDK10	NR_027702	chr16	88280576	88290273	+	.	.	.	.	.	.	.	.	.	.	.	.	.	.	.	.	.	.	.	.
115	5,25	FANCA	NM_000135	chr16	88331459	88410566	-	CL529584	chr16	88377652	+	.	.	.	.	.	.	.	.	.	.	.	.	.	.	.	.
115	5,25	FANCA	NM_000135	chr16	88331459	88410566	-	BH609763	chr16	88350592	-	.	.	.	.	.	.	.	.	.	.	.	.	.	.	.	.
115	5,25	FANCA	NM_000135	chr16	88331459	88410566	-	AY516998.1	chr16	88334091	+	.	.	.	.	.	.	.	.	.	.	.	.	.	.	.	.
6599	0,5	MC1R	NM_002386	chr16	88511787	88514886	+	.	.	.	.	.	.	.	.	.	.	.	.	.	.	.	.	.	.	.	.
5159	1	RPH3AL	NM_006987	chr17	62179	202633	-	.	.	.	.	.	.	.	.	.	.	.	.	.	.	.	.	.	.	.	.
7308	0,25	FAM57A	NM_024792	chr17	582596	592825	+	.	.	.	.	.	.	.	.	.	.	.	.	.	.	.	.	.	.	.	.
7609	0,25	TUSC5	NM_172367	chr17	1129706	1151031	+	.	.	.	.	.	.	.	.	.	.	.	.	.	.	.	.	.	.	.	.
2092	1,5	CRK	NM_016823	chr17	1271396	1306311	-	.	.	.	.	.	.	.	.	.	.	.	.	.	.	.	.	.	.	.	.
4150	1	INPP5K	NM_016532	chr17	1344620	1366932	-	.	.	.	.	.	.	.	.	.	.	.	.	.	.	.	.	.	.	.	.
6770	0,5	PRPF8	NM_006445	chr17	1500672	1534926	-	CL529509	chr17	1514431	+	.	.	.	.	.	.	.	.	.	.	.	.	.	.	.	.
1960	1,75	RPA1	NM_002945	chr17	1680022	1749598	+	CL529170	chr17	1692111	-	.	.	.	.	.	.	.	.	.	.	.	.	.	.	.	.
6837	0,5	RTN4RL1	NM_178568	chr17	1784720	1874928	-	.	.	.	.	.	.	.	.	.	.	.	.	.	.	.	.	.	.	.	.
3606	1	DPH1	NM_001383	chr17	1880180	1893475	+	.	.	.	.	.	.	.	.	.	.	.	.	.	.	.	.	.	.	.	.
2702	1,25	OVCA2	NM_080822	chr17	1892026	1893475	+	.	.	.	.	.	.	.	.	.	.	.	.	.	.	.	.	.	.	.	.
4043	1	HIC1	NM_001098202	chr17	1906353	1909731	+	.	.	.	.	.	.	.	.	.	.	.	.	.	.	.	.	.	.	.	.
7431	0,25	MNT	NM_020310	chr17	2234103	2251008	-	.	.	.	.	.	.	.	.	.	.	.	.	.	.	.	.	.	.	.	.
1592	2	PAFAH1B1	NM_000430	chr17	2443672	2535659	+	BH609876	chr17	2483077	+	.	.	.	.	.	.	.	.	.	.	.	.	.	.	.	.
1592	2	PAFAH1B1	NM_000430	chr17	2443672	2535659	+	BH609405	chr17	2468928	+	.	.	.	.	.	.	.	.	.	.	.	.	.	.	.	.
1592	2	PAFAH1B1	NM_000430	chr17	2443672	2535659	+	CL529171	chr17	2488648	-	.	.	.	.	.	.	.	.	.	.	.	.	.	.	.	.
4223	1	KIAA0664	NM_015229	chr17	2539429	2561677	-	.	.	.	.	.	.	.	.	.	.	.	.	.	.	.	.	.	.	.	.

Table S2

tumor associated genes								HIV				MLV				MMTV				MMTV(SIN)				MMTV(SIN)arrest			
rank	score	gene symbol	RefSEq	chrom	txStart	txEnd	strand	integrant	chr	position	strand	integrant	chr	position	strand	integrant	chr	position	strand	integrant	chr	position	strand	integrant	chr	position	strand
2007	1,5	ASPA	NM_001128085	chr17	3324153	3349450	+	.	.	.	.	.	.	.	.	.	.	.	.	.	.	.	.	.	.	.	.
5236	1	SHPK	NM_013276	chr17	3458304	3486365	-	.	.	.	.	.	.	.	.	.	.	.	.	.	.	.	.	.	.	.	.
6710	0,5	P2RX5	NM_002561	chr17	3523270	3546447	-	.	.	.	.	.	.	.	.	.	.	.	.	.	.	.	.	.	.	.	.
4176	1	ITGAE	NM_002208	chr17	3564667	3651286	-	BH609775	chr17	3571751	+	.	.	.	.	.	.	.	.	.	.	.	.	.	.	.	.
4176	1	ITGAE	NM_002208	chr17	3564667	3651286	-	BH609773	chr17	3639698	+	.	.	.	.	.	.	.	.	.	.	.	.	.	.	.	.
4176	1	ITGAE	NM_002208	chr17	3564667	3651286	-	CL529748	chr17	3643199	+	.	.	.	.	.	.	.	.	.	.	.	.	.	.	.	.
1286	2	CAMKK1	NM_172206	chr17	3710365	3740786	-	.	.	.	.	.	.	.	.	.	.	.	.	.	.	.	.	.	.	.	.
1241	2	ALOX15	NM_001140	chr17	4480962	4491709	-	.	.	.	.	.	.	.	.	.	.	.	.	.	.	.	.	.	.	.	.
2318	1,5	PELP1	NR_046388	chr17	4521427	4554381	-	.	.	.	.	.	.	.	.	.	.	.	.	.	.	.	.	.	.	.	.
7588	0,25	TM4SF5	NM_003963	chr17	4621935	4633253	+	.	.	.	.	.	.	.	.	.	.	.	.	.	.	.	.	.	.	.	.
643	3	PLD2	NM_002663	chr17	4657352	4673694	+	.	.	.	.	.	.	.	.	.	.	.	.	.	.	.	.	.	.	.	.
4400	1	MINK1	NM_170663	chr17	4683302	4742135	+	.	.	.	.	.	.	.	.	.	.	.	.	.	.	.	.	.	.	.	.
3679	1	ENO3	NM_001976	chr17	4795128	4801150	+	.	.	.	.	.	.	.	.	.	.	.	.	.	.	.	.	.	.	.	.
6126	0,5	CAMTA2	NM_001171167	chr17	4812010	4831389	-	.	.	.	.	.	.	.	.	.	.	.	.	.	.	.	.	.	.	.	.
5743	1	ZFP3	NM_153018	chr17	4922477	4940393	+	.	.	.	.	.	.	.	.	.	.	.	.	.	.	.	.	.	.	.	.
1848	2	USP6	NM_004505	chr17	4972410	5019048	+	.	.	.	.	.	.	.	.	.	.	.	.	.	.	.	.	.	.	.	.
1711	2	RABEP1	NM_004703	chr17	5126281	5229856	+	CL800301	chr17	5180524	-	.	.	.	.	.	.	.	.	.	.	.	.	.	.	.	.
1711	2	RABEP1	NM_004703	chr17	5126281	5229856	+	CL800693	chr17	5147293	+	.	.	.	.	.	.	.	.	.	.	.	.	.	.	.	.
6694	0,5	NUP88	NM_002532	chr17	5230069	5263783	-	.	.	.	.	.	.	.	.	.	.	.	.	.	.	.	.	.	.	.	.
4404	1	MIS12	NM_001258220	chr17	5330417	5334854	+	.	.	.	.	.	.	.	.	.	.	.	.	.	.	.	.	.	.	.	.
1577	2	NLRP1	NM_033006	chr17	5358161	5428556	-	CL529173	chr17	5407837	-	.	.	.	.	.	.	.	.	.	.	.	.	.	.	.	.
1577	2	NLRP1	NM_033006	chr17	5358161	5428556	-	AY517216.1	chr17	5395957	+	.	.	.	.	.	.	.	.	.	.	.	.	.	.	.	.
5708	1	WSCD1	NM_015253	chr17	5914657	5968471	+	.	.	.	.	.	.	.	.	.	.	.	.	.	.	.	.	.	.	.	.
2133	1,5	FAM64A	NM_001195228	chr17	6288458	6295109	+	.	.	.	.	.	.	.	.	.	.	.	.	.	.	.	.	.	.	.	.
6900	0,5	SLC13A5	NM_177550	chr17	6528762	6557464	-	.	.	.	.	.	.	.	.	.	.	.	.	.	.	.	.	.	.	.	.
7104	0,5	XAF1	NM_199139	chr17	6599879	6619688	+	.	.	.	.	.	.	.	.	.	.	.	.	.	.	.	.	.	.	.	.
6266	0,5	DLG4	NM_001128827	chr17	7033933	7061652	-	.	.	.	.	.	.	.	.	.	.	.	.	.	.	.	.	.	.	.	.
2873	1	ACADVL	NM_000018	chr17	7063873	7069310	+	.	.	.	.	.	.	.	.	.	.	.	.	.	.	.	.	.	.	.	.
1041	2,25	EIF5A	NM_001970	chr17	7151579	7156506	+	.	.	.	.	.	.	.	.	.	.	.	.	.	.	.	.	.	.	.	.
2173	1,5	GPS2	NM_004489	chr17	7156701	7159382	-	.	.	.	.	.	.	.	.	.	.	.	.	.	.	.	.	.	.	.	.
2875	1	ACAP1	NM_014716	chr17	7180571	7195517	+	.	.	.	.	.	.	.	.	.	.	.	.	.	.	.	.	.	.	.	.
5515	1	TNK1	NM_001251902	chr17	7225016	7233816	+	.	.	.	.	.	.	.	.	.	.	.	.	.	.	.	.	.	.	.	.
7483	0,25	PLSCR3	NM_001201576	chr17	7233770	7238886	-	.	.	.	.	.	.	.	.	.	.	.	.	.	.	.	.	.	.	.	.
4584	1	NLGN2	NM_020795	chr17	7252225	7263903	+	.	.	.	.	.	.	.	.	.	.	.	.	.	.	.	.	.	.	.	.
923	2,5	POLR2A	NM_000937	chr17	7328421	7358659	+	CL529174	chr17	7333637	+	.	.	.	.	.	.	.	.	.	.	.	.	.	.	.	.
923	2,5	POLR2A	NM_000937	chr17	7328421	7358659	+	CL799769	chr17	7342628	-	.	.	.	.	.	.	.	.	.	.	.	.	.	.	.	.
2121	1,5	EIF4A1	NM_001204510	chr17	7416747	7423048	+	.	.	.	.	.	.	.	.	.	.	.	.	.	.	.	.	.	.	.	.
2539	1,25	CD68	NM_001251	chr17	7423528	7426153	+	.	.	.	.	.	.	.	.	.	.	.	.	.	.	.	.	.	.	.	.
2769	1,25	SHBG	NR_027463	chr17	7458106	7477425	+	.	.	.	.	.	.	.	.	.	.	.	.	.	.	.	.	.	.	.	.
1161	2,25	SAT2	NM_133491	chr17	7470280	7471919	-	.	.	.	.	.	.	.	.	.	.	.	.	.	.	.	.	.	.	.	.
6042	0,5	ATP1B2	NM_001678	chr17	7494978	7501814	+	.	.	.	.	.	.	.	.	.	.	.	.	.	.	.	.	.	.	.	.
4	15,75	TP53	NM_001126117	chr17	7512444	7519536	-	.	.	.	.	.	.	.	.	.	.	.	.	.	.	.	.	.	.	.	.
2577	1,25	EFNB3	NM_001406	chr17	7549244	7555418	+	.	.	.	.	.	.	.	.	.	.	.	.	.	.	.	.	.	.	.	.
4212	1	KDM6B	NM_001080424	chr17	7683959	7698843	+	.	.	.	.	.	.	.	.	.	.	.	.	.	.	.	.	.	.	.	.
1440	2	GUCY2D	NM_000180	chr17	7846712	7864383	+	.	.	.	.	.	.	.	.	.	.	.	.	.	.	.	.	.	.	.	.
4034	1	HES7	NM_001165967	chr17	7964632	7968135	-	.	.	.	.	.	.	.	.	.	.	.	.	.	.	.	.	.	.	.	.
1608	2	PER1	NM_002616	chr17	7984512	7996478	-	.	.	.	.	.	.	.	.	.	.	.	.	.	.	.	.	.	.	.	.
7084	0,5	VAMP2	NM_014232	chr17	8003189	8007018	-	.	.	.	.	.	.	.	.	.	.	.	.	.	.	.	.	.	.	.	.
999	2,25	AURKB	NM_001256834	chr17	8048773	8054669	-	.	.	.	.	.	.	.	.	.	.	.	.	.	.	.	.	.	.	.	.
4755	1	PFAS	NM_012393	chr17	8093320	8114534	+	.	.	.	.	.	.	.	.	.	.	.	.	.	.	.	.	.	.	.	.
4809	1	PIK3R6	NM_001010855	chr17	8646779	8711719	-	.	.	.	.	.	.	.	.	.	.	.	.	.	.	.	.	.	.	.	.

Table S2

tumor associated genes								HIV				MLV				MMTV				MMTV(SIN)				MMTV(SIN)arrest			
rank	score	gene symbol	RefSEq	chrom	txStart	txEnd	strand	integrant	chr	position	strand	integrant	chr	position	strand	integrant	chr	position	strand	integrant	chr	position	strand	integrant	chr	position	strand
1132	2,25	PIK3R5	NM_014308	chr17	8722957	8756559	-	.	.	.	.	.	.	.	.	.	.	.	.	.	.	.	.	.	.	.	.
5397	1	STX8	NR_033656	chr17	9094512	9420000	-	AY517190.1	chr17	9266243	-	.	.	.	.	.	.	.	.	.	.	.	.	.	.	.	.
7094	0,5	WDR16	NM_001080556	chr17	9420668	9487501	+	.	.	.	.	.	.	.	.	.	.	.	.	.	.	.	.	.	.	.	.
5658	1	USP43	NM_153210	chr17	9489578	9573728	+	.	.	.	.	.	.	.	.	.	.	.	.	.	.	.	.	.	.	.	.
6386	0,5	GLP2R	NM_004246	chr17	9670105	9733747	+	.	.	.	.	.	.	.	.	.	.	.	.	.	.	.	.	.	.	.	.
2750	1,25	RCVRN	NM_002903	chr17	9741751	9749409	-	.	.	.	.	.	.	.	.	.	.	.	.	.	.	.	.	.	.	.	.
1051	2,25	GAS7	NM_201432	chr17	9754650	9880789	-	.	.	.	.	.	.	.	.	.	.	.	.	.	.	.	.	.	.	.	.
4471	1	MYH1	NM_005963	chr17	10336348	10362584	-	.	.	.	.	.	.	.	.	.	.	.	.	.	.	.	.	.	.	.	.
4472	1	MYH2	NM_001100112	chr17	10365189	10393665	-	.	.	.	.	.	.	.	.	.	.	.	.	.	.	.	.	.	.	.	.
3596	1	DNAH9	NM_001372	chr17	11442472	11813790	+	.	.	.	.	.	.	.	.	.	.	.	.	.	.	.	.	419_103_1	chr17	11554385	-
90	6	MAP2K4	NM_003010	chr17	11864859	11987776	+	.	.	.	.	.	.	.	.	.	.	.	.	.	.	.	.	.	.	.	.
1921	1,75	HS3ST3A1	NM_006042	chr17	13339730	13445969	-	.	.	.	.	.	.	.	.	.	.	.	.	.	.	.	.	.	.	.	.
3429	1	COX10	NM_001303	chr17	13913443	14052721	+	.	.	.	.	.	.	.	.	.	.	.	.	.	.	.	.	.	.	.	.
2194	1,5	HS3ST3B1	NM_006041	chr17	14145230	14190217	+	.	.	.	.	.	.	.	.	.	.	.	.	.	.	.	.	.	.	.	.
5947	0,75	PMP22	NM_153322	chr17	15073821	15104818	-	.	.	.	.	.	.	.	.	.	.	.	.	.	.	.	.	.	.	.	.
7130	0,5	ZNF286A	NM_020652	chr17	15543615	15564825	+	.	.	.	.	.	.	.	.	.	.	.	.	.	.	.	.	.	.	.	.
1562	2	NCOR1	NM_006311	chr17	15874132	16059599	-	CL529451	chr17	15911520	+	.	.	.	.	.	.	.	.	.	.	.	.	.	.	.	.
1562	2	NCOR1	NM_006311	chr17	15874132	16059599	-	BH609765	chr17	16013566	-	.	.	.	.	.	.	.	.	.	.	.	.	.	.	.	.
1562	2	NCOR1	NM_006311	chr17	15874132	16059599	-	BH609766	chr17	16042868	-	.	.	.	.	.	.	.	.	.	.	.	.	.	.	.	.
1562	2	NCOR1	NM_006311	chr17	15874132	16059599	-	CL799771	chr17	15994824	+	.	.	.	.	.	.	.	.	.	.	.	.	.	.	.	.
1562	2	NCOR1	NM_006311	chr17	15874132	16059599	-	AY516979.1	chr17	15974752	-	.	.	.	.	.	.	.	.	.	.	.	.	.	.	.	.
4800	1	PIGL	NM_004278	chr17	16061233	16170298	+	.	.	.	.	.	.	.	.	.	.	.	.	.	.	.	.	.	.	.	.
3340	1	CENPV	NM_181716	chr17	16186572	16197537	-	.	.	.	.	.	.	.	.	.	.	.	.	.	.	.	.	.	.	.	.
5508	1	TNFRSF13B	NM_012452	chr17	16783122	16816127	-	.	.	.	.	.	.	.	.	.	.	.	.	.	.	.	.	.	.	.	.
575	3	FLCN	NM_144606	chr17	17065209	17081227	-	.	.	.	.	.	.	.	.	.	.	.	.	.	.	.	.	.	.	.	.
4635	1	NT5M	NM_020201	chr17	17147404	17191702	+	.	.	.	.	.	.	.	.	.	.	.	.	.	.	.	.	.	.	.	.
2747	1,25	RASD1	NM_001199989	chr17	17338477	17340434	-	.	.	.	.	.	.	.	.	.	.	.	.	.	.	.	.	.	.	.	.
4753	1	PEMT	NM_148172	chr17	17349601	17435742	-	.	.	.	.	.	.	.	.	.	.	.	.	.	.	.	.	.	.	.	.
6639	0,5	MYO15A	NM_016239	chr17	17952744	18023841	+	.	.	.	.	.	.	.	.	.	.	.	.	.	.	.	.	.	.	.	.
4289	1	LLGL1	NM_004140	chr17	18069660	18088913	+	.	.	.	.	.	.	.	.	.	.	.	.	.	.	.	.	.	.	.	.
5234	1	SHMT1	NM_004169	chr17	18171911	18207581	-	CL800246	chr17	18190594	-	.	.	.	.	.	.	.	.	.	.	.	.	.	.	.	.
6772	0,5	PRPSAP2	NM_002767	chr17	18702105	18775324	+	.	.	.	.	.	.	.	.	.	.	.	.	.	.	.	.	.	.	.	.
1935	1,75	MAPK7	NM_139032	chr17	19221626	19227450	+	.	.	.	.	.	.	.	.	.	.	.	.	.	.	.	.	.	.	.	.
7419	0,25	MFAP4	NM_002404	chr17	19227347	19231125	-	.	.	.	.	.	.	.	.	.	.	.	.	.	.	.	.	.	.	.	.
2950	1	ALDH3A2	NM_000382	chr17	19492655	19521500	+	.	.	.	.	.	.	.	.	.	.	.	.	.	.	.	.	.	.	.	.
2949	1	ALDH3A1	NM_000691	chr17	19581889	19592338	-	.	.	.	.	.	.	.	.	.	.	.	.	.	.	.	.	.	.	.	.
5639	1	ULK2	NM_014683	chr17	19614734	19711831	-	.	.	.	.	.	.	.	.	.	.	.	.	.	.	.	.	.	.	.	.
739	2,75	MAP2K3	NM_002756	chr17	21131940	21159144	+	.	.	.	.	.	.	.	.	.	.	.	.	.	.	.	.	.	.	.	.
6501	0,5	KCNJ12	NM_021012	chr17	21220291	21263772	+	.	.	.	.	.	.	.	.	.	.	.	.	.	.	.	.	.	.	.	.
2643	1,25	KSR1	NM_014238	chr17	22823162	22974845	+	.	.	.	.	AY516376	chr17	22885014	-	.	.	.	.	.	.	.	.	.	.	.	.
2643	1,25	KSR1	NM_014238	chr17	22823162	22974845	+	.	.	.	.	AY516739	chr17	22950587	-	.	.	.	.	.	.	.	.	.	.	.	.
99	5,75	NOS2	NM_000625	chr17	23107918	23151682	-	.	.	.	.	.	.	.	.	.	.	.	.	.	.	.	.	.	.	.	.
2695	1,25	NLK	NM_016231	chr17	23393814	23547531	+	.	.	.	.	.	.	.	.	.	.	.	.	.	.	.	.	.	.	.	.
7091	0,5	VTN	NM_000638	chr17	23718425	23721500	-	.	.	.	.	.	.	.	.	.	.	.	.	.	.	.	.	.	.	.	.
7318	0,25	FOXN1	NM_003593	chr17	23875085	23889302	+	.	.	.	.	.	.	.	.	.	.	.	.	.	.	.	.	.	.	.	.
289	4	PIGS	NM_033198	chr17	23904532	23923014	-	.	.	.	.	.	.	.	.	.	.	.	.	.	.	.	.	.	.	.	.
989	2,25	ALDOC	NM_005165	chr17	23924259	23928078	-	.	.	.	.	.	.	.	.	.	.	.	.	.	.	.	.	.	.	.	.
1769	2	SPAG5	NM_006461	chr17	23928709	23950183	-	CL800153	chr17	23946424	+	.	.	.	.	.	.	.	.	.	.	.	.	.	.	.	.
5030	1	RAB34	NM_001142625	chr17	24065425	24069413	-	.	.	.	.	.	.	.	.	.	.	.	.	.	.	.	.	.	.	.	.
4564	1	NEK8	NM_178170	chr17	24079958	24093911	+	.	.	.	.	.	.	.	.	.	.	.	.	.	.	.	.	.	.	.	.
2323	1,5	PIPOX	NM_016518	chr17	24394043	24408362	+	.	.	.	.	.	.	.	.	.	.	.	.	.	.	.	.	.	.	.	.

Table S2

tumor associated genes								HIV				MLV				MMTV				MMTV(SIN)				MMTV(SIN)arrest			
rank	score	gene symbol	RefSEq	chrom	txStart	txEnd	strand	integrant	chr	position	strand	integrant	chr	position	strand	integrant	chr	position	strand	integrant	chr	position	strand	integrant	chr	position	strand
5469	1	TIAF1	NM_004740	chr17	24424664	24426753	-	.	.	.	.	.	.	.	.	.	.	.	.	.	.	.	.	.	.	.	.
1793	2	TAOK1	NM_020791	chr17	24742068	24903047	+	.	.	.	.	.	.	.	.	.	.	.	.	.	.	.	.	3286_344_1	chr17	24875083	+
3891	1	GIT1	NM_014030	chr17	24924612	24940736	-	.	.	.	.	.	.	.	.	.	.	.	.	.	.	.	.	.	.	.	.
3427	1	CORO6	NM_032854	chr17	24965899	24972567	-	.	.	.	.	.	.	.	.	.	.	.	.	.	.	.	.	.	.	.	.
6034	0,5	ATAD5	NM_024857	chr17	26183148	26246421	+	CL800720	chr17	26224861	-	.	.	.	.	.	.	.	.	.	.	.	.	.	.	.	.
6034	0,5	ATAD5	NM_024857	chr17	26183148	26246421	+	CL800844	chr17	26241548	+	.	.	.	.	.	.	.	.	.	.	.	.	.	.	.	.
6034	0,5	ATAD5	NM_024857	chr17	26183148	26246421	+	AY517123.1	chr17	26229249	-	.	.	.	.	.	.	.	.	.	.	.	.	.	.	.	.
7	12,25	NF1	NM_000267	chr17	26446070	26728821	+	CL800011	chr17	26601244	+	.	.	.	.	.	.	.	.	.	.	.	.	.	.	.	.
1125	2,25	OMG	NM_002544	chr17	26645793	26648506	-	.	.	.	.	.	.	.	.	.	.	.	.	.	.	.	.	.	.	.	.
3702	1	EVI2A	NM_014210	chr17	26667553	26672893	-	.	.	.	.	.	.	.	.	.	.	.	.	.	.	.	.	.	.	.	.
5020	1	RAB11FIP4	NM_032932	chr17	26742767	26889352	+	.	.	.	.	.	.	.	.	.	.	.	.	.	.	.	.	.	.	.	.
1789	2	SUZ12	NM_015355	chr17	27288156	27352170	+	.	.	.	.	.	.	.	.	.	.	.	.	.	.	.	.	.	.	.	.
1892	1,75	CDK5R1	NM_003885	chr17	27838217	27842384	+	.	.	.	.	.	.	.	.	.	.	.	.	.	.	.	.	.	.	.	.
802	2,5	CCL2	NM_002982	chr17	29606408	29608333	+	.	.	.	.	.	.	.	.	.	.	.	.	.	.	.	.	.	.	.	.
610	3	LIG3	NM_002311	chr17	30331629	30354304	+	.	.	.	.	.	.	.	.	.	.	.	.	.	.	.	.	.	.	.	.
5102	1	RFFL	NR_037713	chr17	30360243	30440461	-	CL529496	chr17	30422044	+	.	.	.	.	.	.	.	.	.	.	.	.	.	.	.	.
5289	1	SLFN11	NM_001104587	chr17	30701440	30724833	-	.	.	.	.	.	.	.	.	.	.	.	.	.	.	.	.	.	.	.	.
1791	2	TAF15	NM_003487	chr17	31160571	31198359	+	.	.	.	.	.	.	.	.	.	.	.	.	.	.	.	.	.	.	.	.
2534	1,25	CCL18	NM_002988	chr17	31415755	31422954	+	.	.	.	.	.	.	.	.	.	.	.	.	.	.	.	.	.	.	.	.
2536	1,25	CCL4	NM_002984	chr17	31455332	31457127	+	.	.	.	.	.	.	.	.	.	.	.	.	.	.	.	.	.	.	.	.
7229	0,25	CCL4L1	NM_001001435	chr17	31562580	31564387	+	.	.	.	.	.	.	.	.	.	.	.	.	.	.	.	.	.	.	.	.
4476	1	MYO19	NM_001033580	chr17	31943029	31965418	-	.	.	.	.	.	.	.	.	.	.	.	.	.	.	.	.	.	.	.	.
4805	1	PIGW	NM_178517	chr17	31965515	31969263	+	.	.	.	.	.	.	.	.	.	.	.	.	.	.	.	.	.	.	.	.
2866	1	ACACA	NM_198839	chr17	32516039	32841015	-	CL800541	chr17	32616758	+	.	.	.	.	.	.	.	.	.	.	.	.	.	.	.	.
3619	1	DUSP14	NM_007026	chr17	32924063	32947701	+	.	.	.	.	.	.	.	.	.	.	.	.	.	.	.	.	.	.	.	.
3553	1	DDX52	NM_007010	chr17	33046475	33077606	-	.	.	.	.	.	.	.	.	.	.	.	.	.	.	.	.	.	.	.	.
4062	1	HNF1B	NM_001165923	chr17	33120546	33179209	-	.	.	.	.	.	.	.	.	.	.	.	.	.	.	.	.	.	.	.	.
1539	2	MLLT6	NM_005937	chr17	34115398	34139582	+	.	.	.	.	.	.	.	.	.	.	.	.	.	.	.	.	.	.	.	.
1626	2	PIP4K2B	NM_003559	chr17	34175469	34209684	-	.	.	.	.	.	.	.	.	.	.	.	.	.	.	.	.	.	.	.	.
1498	2	LASP1	NM_006148	chr17	34279637	34331549	+	.	.	.	.	.	.	.	.	.	.	.	.	.	.	.	.	.	.	.	.
2045	1,5	CACNB1	NM_199247	chr17	34586914	34607482	-	.	.	.	.	.	.	.	.	.	.	.	.	.	.	.	.	.	.	.	.
2251	1,5	MED1	NM_004774	chr17	34814063	34861053	-	.	.	.	.	.	.	.	.	.	.	.	.	.	.	.	.	.	.	.	.
6664	0,5	NEUROD2	NM_006160	chr17	35013546	35017701	-	.	.	.	.	.	.	.	.	.	.	.	.	.	.	.	.	.	.	.	.
2338	1,5	PPP1R1B	NM_181505	chr17	35038276	35046404	+	.	.	.	.	.	.	.	.	.	.	.	.	.	.	.	.	.	.	.	.
2329	1,5	PNMT	NM_002686	chr17	35078032	35080254	+	.	.	.	.	.	.	.	.	.	.	.	.	.	.	.	.	.	.	.	.
21	9,25	ERBB2	NM_001005862	chr17	35097918	35138441	+	.	.	.	.	.	.	.	.	.	.	.	.	.	.	.	.	.	.	.	.
3965	1	GRB7	NM_005310	chr17	35147687	35157064	+	.	.	.	.	.	.	.	.	.	.	.	.	.	.	.	.	.	.	.	.
1467	2	IKZF3	NR_047559	chr17	35167493	35273967	-	CL799682	chr17	35200088	+	.	.	.	.	.	.	.	.	.	.	.	.	.	.	.	.
1467	2	IKZF3	NR_047559	chr17	35167493	35273967	-	AY517150.1	chr17	35267044	-	.	.	.	.	.	.	.	.	.	.	.	.	.	.	.	.
1467	2	IKZF3	NR_047559	chr17	35167493	35273967	-	AY517153.1	chr17	35267044	-	.	.	.	.	.	.	.	.	.	.	.	.	.	.	.	.
1467	2	IKZF3	NR_047559	chr17	35167493	35273967	-	BH609780	chr17	35239056	+	.	.	.	.	.	.	.	.	.	.	.	.	.	.	.	.
810	2,5	CSF3	NM_001178147	chr17	35425139	35427592	+	.	.	.	.	.	.	.	.	.	.	.	.	.	.	.	.	.	.	.	.
2436	1,5	THRA	NM_001190919	chr17	35471971	35503646	+	.	.	.	.	.	.	.	.	.	.	.	.	.	.	.	.	.	.	.	.
4604	1	NR1D1	NM_021724	chr17	35502562	35510499	-	.	.	.	.	.	.	.	.	.	.	.	.	.	.	.	.	.	.	.	.
6794	0,5	RAPGEFL1	NM_016339	chr17	35587767	35605432	+	.	.	.	.	.	.	.	.	.	.	.	.	.	.	.	.	.	.	.	.
191	4,5	RARA	NM_001024809	chr17	35751796	35767421	+	CL800240	chr17	35761006	+	.	.	.	.	.	.	.	.	.	.	.	.	.	.	.	.
197	4,5	TOP2A	NM_001067	chr17	35798298	35827728	-	CL800195	chr17	35811269	-	.	.	.	.	.	.	.	.	.	.	.	.	.	.	.	.
2621	1,25	IGFBP4	NM_001552	chr17	35853201	35867508	+	.	.	.	.	.	.	.	.	.	.	.	.	.	.	.	.	.	.	.	.
7596	0,25	TNS4	NM_032865	chr17	35885605	35911380	-	.	.	.	.	AY516818	chr17	35895598	-	.	.	.	.	.	.	.	.	.	.	.	.
7230	0,25	CCR7	NM_001838	chr17	35963547	35975262	-	.	.	.	.	.	.	.	.	.	.	.	.	.	.	.	.	.	.	.	.
5295	1	SMARCE1	NM_003079	chr17	36037505	36057629	-	.	.	.	.	.	.	.	.	.	.	.	.	.	.	.	.	.	.	.	.

Table S2

tumor associated genes								HIV				MLV				MMTV				MMTV(SIN)				MMTV(SIN)arrest			
rank	score	gene symbol	RefSEq	chrom	txStart	txEnd	strand	integrant	chr	position	strand	integrant	chr	position	strand	integrant	chr	position	strand	integrant	chr	position	strand	integrant	chr	position	strand
2640	1,25	KRT20	NM_019010	chr17	36285666	36295021	-	.	.	.	.	.	.	.	.	.	.	.	.	.	.	.	.	.	.	.	.
6544	0,5	KRTAP4-12	NM_031854	chr17	36532870	36533945	-	.	.	.	.	.	.	.	.	.	.	.	.	.	.	.	.	.	.	.	.
7391	0,25	KRT13	NM_153490	chr17	36910758	36915391	-	.	.	.	.	.	.	.	.	.	.	.	.	.	.	.	.	.	.	.	.
2639	1,25	KRT19	NM_002276	chr17	36933394	36938167	-	.	.	.	.	.	.	.	.	.	.	.	.	.	.	.	.	.	.	.	.
4251	1	KRT9	NM_000226	chr17	36975619	36981836	-	.	.	.	.	.	.	.	.	.	.	.	.	.	.	.	.	.	.	.	.
1422	2	GAST	NM_000805	chr17	37122103	37125747	+	.	.	.	.	.	.	.	.	.	.	.	.	.	.	.	.	.	.	.	.
1088	2,25	JUP	NM_021991	chr17	37164384	37196490	-	.	.	.	.	.	.	.	.	.	.	.	.	.	.	.	.	.	.	.	.
2880	1	ACLY	NM_198830	chr17	37276704	37328798	-	.	.	.	.	.	.	.	.	.	.	.	.	.	.	.	.	.	.	.	.
4580	1	NKIRAS2	NM_001144929	chr17	37425612	37431182	+	.	.	.	.	.	.	.	.	.	.	.	.	.	.	.	.	.	.	.	.
3581	1	DHX58	NM_024119	chr17	37506949	37518277	-	.	.	.	.	.	.	.	.	.	.	.	.	.	.	.	.	.	.	.	.
5039	1	RAB5C	NM_001252039	chr17	37530519	37560588	-	.	.	.	.	.	.	.	.	.	.	.	.	.	.	.	.	.	.	.	.
1782	2	STAT5B	NM_012448	chr17	37604720	37681950	-	CL800613	chr17	37641703	+	.	.	.	.	.	.	.	.	.	.	.	.	.	.	.	.
1782	2	STAT5B	NM_012448	chr17	37604720	37681950	-	CL529378	chr17	37641496	+	.	.	.	.	.	.	.	.	.	.	.	.	.	.	.	.
1782	2	STAT5B	NM_012448	chr17	37604720	37681950	-	CL529377	chr17	37673997	-	.	.	.	.	.	.	.	.	.	.	.	.	.	.	.	.
1782	2	STAT5B	NM_012448	chr17	37604720	37681950	-	CL800231	chr17	37665523	+	.	.	.	.	.	.	.	.	.	.	.	.	.	.	.	.
1782	2	STAT5B	NM_012448	chr17	37604720	37681950	-	BH609782	chr17	37663646	-	.	.	.	.	.	.	.	.	.	.	.	.	.	.	.	.
1781	2	STAT5A	NM_003152	chr17	37693090	37717486	+	.	.	.	.	.	.	.	.	.	.	.	.	.	.	.	.	.	.	.	.
431	3,5	STAT3	NM_003150	chr17	37718868	37794039	-	CL529621	chr17	37761182	+	.	.	.	.	.	.	.	.	.	.	.	.	.	.	.	.
431	3,5	STAT3	NM_003150	chr17	37718868	37794039	-	CL529622	chr17	37761452	-	.	.	.	.	.	.	.	.	.	.	.	.	.	.	.	.
5005	1	PTRF	NM_012232	chr17	37807992	37828864	-	.	.	.	.	.	.	.	.	.	.	.	.	.	.	.	.	.	.	.	.
1266	2	ATP6V0A1	NM_005177	chr17	37864387	37928123	+	CL800226	chr17	37869452	+	AY516780	chr17	37926598	-	.	.	.	.	.	.	.	.	.	.	.	.
4490	1	NAGLU	NM_000263	chr17	37941476	37949992	+	.	.	.	.	.	.	.	.	.	.	.	.	.	.	.	.	.	.	.	.
590	3	HSD17B1	NM_000413	chr17	37957509	37960758	+	.	.	.	.	.	.	.	.	.	.	.	.	.	.	.	.	.	.	.	.
3410	1	COASY	NM_025233	chr17	37967617	37971825	+	.	.	.	.	.	.	.	.	.	.	.	.	.	.	.	.	.	.	.	.
4971	1	PSMC3IP	NM_001256014	chr17	37977853	37983260	-	.	.	.	.	.	.	.	.	.	.	.	.	.	.	.	.	.	.	.	.
1858	2	WNK4	NM_032387	chr17	38186174	38202610	+	.	.	.	.	.	.	.	.	.	.	.	.	.	.	.	.	.	.	.	.
3140	1	BECN1	NM_003766	chr17	38215675	38229836	-	.	.	.	.	.	.	.	.	.	.	.	.	.	.	.	.	.	.	.	.
2995	1	AOC2	NM_009590	chr17	38250134	38256251	+	.	.	.	.	.	.	.	.	.	.	.	.	.	.	.	.	.	.	.	.
1248	2	AOC3	NM_003734	chr17	38256726	38263666	+	.	.	.	.	.	.	.	.	.	.	.	.	.	.	.	.	.	.	.	.
3826	1	G6PC	NM_001270397	chr17	38306339	38319976	+	.	.	.	.	.	.	.	.	.	.	.	.	.	.	.	.	.	.	.	.
5133	1	RND2	NM_005440	chr17	38430783	38437584	+	.	.	.	.	.	.	.	.	.	.	.	.	.	.	.	.	.	.	.	.
18	9,5	BRCA1	NR_027676	chr17	38449837	38530866	-	.	.	.	.	.	.	.	.	.	.	.	.	.	.	.	.	.	.	.	.
4501	1	NBR2	NR_003108	chr17	38531125	38550651	+	.	.	.	.	.	.	.	.	.	.	.	.	.	.	.	.	.	.	.	.
1559	2	NBR1	NM_005899	chr17	38576771	38719233	+	.	.	.	.	.	.	.	.	.	.	.	.	.	.	.	.	.	.	.	.
3036	1	ARL4D	NM_001661	chr17	38831878	38834030	+	.	.	.	.	.	.	.	.	.	.	.	.	.	.	.	.	.	.	.	.
210	4,25	ETV4	NM_001079675	chr17	38960736	38979326	-	.	.	.	.	.	.	.	.	.	.	.	.	.	.	.	.	.	.	.	.
565	3	DUSP3	NM_004090	chr17	39199014	39211894	-	.	.	.	.	.	.	.	.	.	.	.	.	.	.	.	.	.	.	.	.
7434	0,25	MPP3	NR_003562	chr17	39233692	39266073	-	.	.	.	.	.	.	.	.	.	.	.	.	.	.	.	.	.	.	.	.
4491	1	NAGS	NM_153006	chr17	39437557	39441962	+	.	.	.	.	.	.	.	.	.	.	.	.	.	.	.	.	.	.	.	.
1443	2	HDAC5	NM_001015053	chr17	39509646	39556540	-	.	.	.	.	.	.	.	.	.	.	.	.	.	.	.	.	.	.	.	.
5264	1	SLC25A39	NM_001143780	chr17	39752518	39757743	-	.	.	.	.	.	.	.	.	.	.	.	.	.	.	.	.	.	.	.	.
1917	1,75	GRN	NM_002087	chr17	39778016	39785996	+	.	.	.	.	.	.	.	.	.	.	.	.	.	.	.	.	.	.	.	.
5925	0,75	ITGA2B	NM_000419	chr17	39805075	39822399	-	.	.	.	.	.	.	.	.	.	.	.	.	.	.	.	.	.	.	.	.
3824	1	FZD2	NM_001466	chr17	39990337	39994156	+	.	.	.	.	.	.	.	.	.	.	.	.	.	.	.	.	.	.	.	.
5991	0,5	ADAM11	NM_002390	chr17	40192093	40214740	+	.	.	.	.	.	.	.	.	.	.	.	.	.	.	.	.	.	.	.	.
2601	1,25	GJC1	NM_001080383	chr17	40231341	40263705	-	.	.	.	.	.	.	.	.	.	.	.	.	.	.	.	.	.	.	.	.
6429	0,5	HIGD1B	NM_016438	chr17	40280804	40283374	+	.	.	.	.	.	.	.	.	.	.	.	.	.	.	.	.	.	.	.	.
3656	1	EFTUD2	NM_001258354	chr17	40283180	40332519	-	.	.	.	.	.	.	.	.	.	.	.	.	.	.	.	.	.	.	.	.
581	3	GFAP	NM_001242376	chr17	40342560	40348446	-	.	.	.	.	.	.	.	.	.	.	.	.	.	.	.	.	.	.	.	.
1880	1,75	C1QL1	NM_006688	chr17	40392586	40401170	-	.	.	.	.	.	.	.	.	.	.	.	.	.	.	.	.	.	.	.	.
3532	1	DCAKD	NM_024819	chr17	40456231	40493999	-	CL799983	chr17	40476915	-	.	.	.	.	.	.	.	.	.	.	.	.	.	.	.	.

Table S2

tumor associated genes								HIV				MLV				MMTV				MMTV(SIN)				MMTV(SIN)arrest			
rank	score	gene symbol	RefSEq	chrom	txStart	txEnd	strand	integrant	chr	position	strand	integrant	chr	position	strand	integrant	chr	position	strand	integrant	chr	position	strand	integrant	chr	position	strand
4836	1	PLCD3	NM_133373	chr17	40544533	40565417	-	.	.	.	.	.	.	.	.	.	.	.	.	.	.	.	.	.	.	.	.
2142	1,5	FMNL1	NM_005892	chr17	40655074	40680466	+	.	.	.	.	AY516582	chr17	40661827	-	.	.	.	.	.	.	.	.	.	.	.	.
2656	1,25	MAP3K14	NM_003954	chr17	40696270	40750197	-	.	.	.	.	.	.	.	.	.	.	.	.	.	.	.	.	.	.	.	.
7170	0,25	ARHGAP27	NM_174919	chr17	40862500	40866065	-	.	.	.	.	.	.	.	.	.	.	.	.	.	.	.	.	.	.	.	.
2243	1,5	MAPT	NM_016834	chr17	41327541	41461546	+	.	.	.	.	.	.	.	.	.	.	.	.	.	.	.	.	.	.	.	.
4629	1	NSF	NR_040116	chr17	42023350	42189995	+	.	.	.	.	.	.	.	.	.	.	.	.	.	.	.	.	.	.	.	.
5704	1	WNT3	NM_030753	chr17	42196856	42251081	-	.	.	.	.	.	.	.	.	.	.	.	.	.	.	.	.	.	.	.	.
5707	1	WNT9B	NM_003396	chr17	42283966	42309436	+	.	.	.	.	.	.	.	.	.	.	.	.	.	.	.	.	.	.	.	.
6830	0,5	RPRML	NM_203400	chr17	42410520	42411613	-	.	.	.	.	.	.	.	.	.	.	.	.	.	.	.	.	.	.	.	.
1298	2	CDC27	NM_001114091	chr17	42550309	42621664	-	.	.	.	.	.	.	.	.	_426_1_267	chr17	42568677	-	.	.	.	.	.	.	.	.
333	3,75	ITGB3	NM_000212	chr17	42686206	42745076	+	.	.	.	.	.	.	.	.	.	.	.	.	.	.	.	.	.	.	.	.
4245	1	KPNB1	NM_002265	chr17	43082273	43116003	+	.	.	.	.	.	.	.	.	.	.	.	.	.	.	.	.	.	.	.	.
4860	1	PNPO	NM_018129	chr17	43373887	43381673	+	.	.	.	.	.	.	.	.	.	.	.	.	.	.	.	.	.	.	.	.
6207	0,5	COPZ2	NM_016429	chr17	43458531	43470151	-	.	.	.	.	.	.	.	.	.	.	.	.	.	.	.	.	.	.	.	.
6667	0,5	NFE2L1	NM_003204	chr17	43480684	43493906	+	.	.	.	.	.	.	.	.	.	.	.	.	.	.	.	.	.	.	.	.
6136	0,5	CBX1	NM_001127228	chr17	43502412	43533882	-	.	.	.	.	.	.	.	.	.	.	.	.	.	.	.	.	.	.	.	.
6946	0,5	SNX11	NM_013323	chr17	43539945	43555104	+	.	.	.	.	.	.	.	.	.	.	.	.	.	.	.	.	.	.	.	.
5249	1	SKAP1	NM_001075099	chr17	43565800	43862593	-	CL800093	chr17	43778055	+	.	.	.	.	_279_1_183	chr17	43737251	-	.	.	.	.	.	.	.	.
7356	0,25	HOXB1	NM_002144	chr17	43961805	43963271	-	.	.	.	.	.	.	.	.	.	.	.	.	.	.	.	.	.	.	.	.
6447	0,5	HOXB2	NM_002145	chr17	43975017	43977392	-	.	.	.	.	.	.	.	.	.	.	.	.	.	.	.	.	.	.	.	.
1919	1,75	HOXB3	NM_002146	chr17	43981230	44006809	-	.	.	.	.	.	.	.	.	.	.	.	.	.	.	.	.	.	.	.	.
7358	0,25	HOXB4	NM_024015	chr17	44007867	44010742	-	.	.	.	.	.	.	.	.	.	.	.	.	.	.	.	.	.	.	.	.
4069	1	HOXB5	NM_002147	chr17	44023617	44026102	-	.	.	.	.	.	.	.	.	.	.	.	.	.	.	.	.	.	.	.	.
4070	1	HOXB6	NM_018952	chr17	44028097	44037333	-	.	.	.	.	.	.	.	.	.	.	.	.	.	.	.	.	.	.	.	.
4071	1	HOXB7	NM_004502	chr17	44039593	44043382	-	.	.	.	.	.	.	.	.	.	.	.	.	.	.	.	.	.	.	.	.
7359	0,25	HOXB8	NM_024016	chr17	44044706	44047300	-	.	.	.	.	.	.	.	.	.	.	.	.	.	.	.	.	.	.	.	.
7357	0,25	HOXB13	NM_006361	chr17	44157124	44161110	-	.	.	.	.	.	.	.	.	.	.	.	.	.	.	.	.	.	.	.	.
3075	1	ATP5G1	NM_005175	chr17	44325146	44328231	+	.	.	.	.	.	.	.	.	.	.	.	.	.	.	.	.	.	.	.	.
1075	2,25	IGF2BP1	NM_006546	chr17	44429772	44488506	+	.	.	.	.	.	.	.	.	.	.	.	.	.	.	.	.	.	.	.	.
3928	1	GNGT2	NM_001198754	chr17	44638594	44642935	-	.	.	.	.	.	.	.	.	.	.	.	.	.	.	.	.	.	.	.	.
7147	0,25	ABI3	NM_001135186	chr17	44642587	44655586	+	.	.	.	.	.	.	.	.	.	.	.	.	.	.	.	.	.	.	.	.
1614	2	PHOSPHO1	NM_001143804	chr17	44655730	44663127	-	.	.	.	.	.	.	.	.	.	.	.	.	.	.	.	.	.	.	.	.
916	2,5	PHB	NM_002634	chr17	44836418	44847241	-	.	.	.	.	.	.	.	.	.	.	.	.	.	.	.	.	.	.	.	.
2694	1,25	NGFR	NM_002507	chr17	44927653	44947381	+	.	.	.	.	.	.	.	.	.	.	.	.	.	.	.	.	.	.	.	.
7279	0,25	DLX4	NM_001934	chr17	45405128	45407322	+	.	.	.	.	.	.	.	.	.	.	.	.	.	.	.	.	.	.	.	.
2630	1,25	ITGA3	NM_002204	chr17	45488338	45522848	+	.	.	.	.	.	.	.	.	.	.	.	.	.	.	.	.	.	.	.	.
2315	1,5	PDK2	NM_001199899	chr17	45528057	45543732	+	.	.	.	.	.	.	.	.	.	.	.	.	.	.	.	.	.	.	.	.
4921	1	PPP1R9B	NM_032595	chr17	45566099	45582876	-	.	.	.	.	.	.	.	.	.	.	.	.	.	.	.	.	.	.	.	.
88	6	COL1A1	NM_000088	chr17	45616455	45633999	-	.	.	.	.	.	.	.	.	.	.	.	.	.	.	.	.	.	.	.	.
5719	1	XYLT2	NM_022167	chr17	45778391	45793511	+	.	.	.	.	.	.	.	.	.	.	.	.	.	.	.	.	.	.	.	.
3676	1	EME1	NM_152463	chr17	45805579	45813819	+	.	.	.	.	.	.	.	.	.	.	.	.	.	.	.	.	.	.	.	.
4308	1	LRRC59	NM_018509	chr17	45813592	45829913	-	.	.	.	.	.	.	.	.	.	.	.	.	.	.	.	.	.	.	.	.
6317	0,5	EPN3	NM_017957	chr17	45965046	45976110	+	.	.	.	.	.	.	.	.	.	.	.	.	.	.	.	.	.	.	.	.
797	2,5	CACNA1G	NM_001256334	chr17	45993427	46059831	+	.	.	.	.	.	.	.	.	.	.	.	.	.	.	.	.	.	.	.	.
150	4,75	ABCC3	NM_001144070	chr17	46067216	46100287	+	.	.	.	.	AY516737	chr17	46093846	-	.	.	.	.	.	.	.	.	.	.	.	.
7597	0,25	TOB1	NM_001243877	chr17	46294585	46300338	-	.	.	.	.	.	.	.	.	.	.	.	.	.	.	.	.	.	.	.	.
1120	2,25	NME1	NM_000269	chr17	46585918	46594449	+	.	.	.	.	.	.	.	.	.	.	.	.	.	.	.	.	.	.	.	.
4589	1	NME2	NM_001018138	chr17	46599081	46604104	+	.	.	.	.	.	.	.	.	.	.	.	.	.	.	.	.	.	.	.	.
1881	1,75	CA10	NM_001082533	chr17	47062672	47592160	-	.	.	.	.	.	.	.	.	.	.	.	.	.	.	.	.	.	.	.	.
5521	1	TOM1L1	NM_005486	chr17	50333050	50394327	+	.	.	.	.	.	.	.	.	.	.	.	.	.	.	.	.	.	.	.	.
857	2,5	HLF	NM_002126	chr17	50697319	50757425	+	.	.	.	.	.	.	.	.	.	.	.	.	.	.	.	.	.	.	.	.

Table S2

tumor associated genes								HIV				MLV				MMTV				MMTV(SIN)				MMTV(SIN)arrest			
rank	score	gene symbol	RefSEq	chrom	txStart	txEnd	strand	integrant	chr	position	strand	integrant	chr	position	strand	integrant	chr	position	strand	integrant	chr	position	strand	integrant	chr	position	strand
6617	0,5	MMD	NM_012329	chr17	50824972	50854340	-	CL529733	chr17	50849578	+	.	.	.	.	.	.	.	.	.	.	.	.	.	.	.	.
7028	0,5	TMEM100	NM_001099640	chr17	51151986	51164481	-	.	.	.	.	.	.	.	.	.	.	.	.	70_1_2753_19	chr17	51152835	-	1169_133_2	chr17	51161679	+
561	3	DGKE	NM_003647	chr17	52266458	52301035	+	CL800257	chr17	52275974	-	.	.	.	.	.	.	.	.	.	.	.	.	.	.	.	.
5551	1	TRIM25	NM_005082	chr17	52320268	52346408	-	.	.	.	.	.	.	.	.	.	.	.	.	.	.	.	.	.	.	.	.
6860	0,5	SCPEP1	NM_021626	chr17	52410466	52439128	+	.	.	.	.	.	.	.	.	.	.	.	.	.	.	.	.	.	.	.	.
1545	2	MSI2	NM_138962	chr17	52688929	53112298	+	CL799886	chr17	52843722	-	.	.	.	.	.	.	.	.	.	.	.	.	1263_137_1	chr17	53025581	+
7087	0,5	VEZF1	NM_007146	chr17	53403908	53420614	-	.	.	.	.	.	.	.	.	.	.	.	.	.	.	.	.	.	.	.	.
3694	1	EPX	NM_000502	chr17	53625087	53637534	+	.	.	.	.	.	.	.	.	.	.	.	.	.	.	.	.	.	.	.	.
4298	1	LPO	NM_001160102	chr17	53670785	53700878	+	.	.	.	.	.	.	.	.	.	.	.	.	.	.	.	.	.	.	.	.
1113	2,25	MPO	NM_000250	chr17	53702215	53713295	-	.	.	.	.	.	.	.	.	.	.	.	.	.	.	.	.	.	.	.	.
6072	0,5	BZRAP1	NM_004758	chr17	53733586	53761151	-	.	.	.	.	.	.	.	.	.	.	.	.	.	.	.	.	.	.	.	.
1802	2	TEX14	NM_031272	chr17	53989036	54124415	-	.	.	.	.	.	.	.	.	.	.	.	.	.	.	.	.	.	.	.	.
5052	1	RAD51C	NM_058216	chr17	54124961	54166691	+	.	.	.	.	.	.	.	.	.	.	.	.	.	.	.	.	.	.	.	.
932	2,5	PPM1E	NR_048561	chr17	54188228	54417322	+	.	.	.	.	.	.	.	.	.	.	.	.	.	.	.	.	.	.	.	.
5555	1	TRIM37	NM_001005207	chr17	54414781	54539048	-	CL799695	chr17	54519099	+	AY516624	chr17	54425577	+	.	.	.	.	.	.	.	.	.	.	.	.
243	4	CLTC	NM_004859	chr17	55051831	55129099	+	CL800209	chr17	55119721	-	.	.	.	.	.	.	.	.	.	.	.	.	.	.	.	.
301	4	RPS6KB1	NM_003161	chr17	55325224	55382568	+	CL799634	chr17	55368171	+	.	.	.	.	.	.	.	.	.	.	.	.	.	.	.	.
2042	1,5	CA4	NM_000717	chr17	55582083	55591688	+	.	.	.	.	.	.	.	.	.	.	.	.	.	.	.	.	.	.	.	.
3011	1	APPBP2	NM_006380	chr17	55875301	55958362	-	.	.	.	.	.	.	.	.	.	.	.	.	.	.	.	.	.	.	.	.
931	2,5	PPM1D	NM_003620	chr17	56032325	56098422	+	.	.	.	.	.	.	.	.	.	.	.	.	.	.	.	.	.	.	.	.
3131	1	BCAS3	NM_017679	chr17	56109953	56824981	+	BH609791	chr17	56303571	+	AY515886	chr17	56473618	-	.	.	.	.	.	.	.	.	3290_345_1	chr17	56151021	+
3131	1	BCAS3	NM_017679	chr17	56109953	56824981	+	BH609790	chr17	56281047	+	AY515886	chr17	56473618	-	.	.	.	.	.	.	.	.	3290_345_1	chr17	56151021	+
3131	1	BCAS3	NM_017679	chr17	56109953	56824981	+	CL529708	chr17	56505224	-	AY515886	chr17	56473618	-	.	.	.	.	.	.	.	.	3290_345_1	chr17	56151021	+
7011	0,5	TBX2	NM_005994	chr17	56832038	56841609	+	.	.	.	.	.	.	.	.	.	.	.	.	.	.	.	.	.	.	.	.
5440	1	TBX4	NM_018488	chr17	56888588	56916446	+	.	.	.	.	.	.	.	.	.	.	.	.	.	.	.	.	.	.	.	.
239	4	BRIP1	NM_032043	chr17	57111328	57295702	-	.	.	.	.	.	.	.	.	.	.	.	.	1555_150	chr17	57205805	-	.	.	.	.
5477	1	TLK2	NM_001112707	chr17	57910117	58046573	+	.	.	.	.	.	.	.	.	.	.	.	.	.	.	.	.	.	.	.	.
6627	0,5	MRC2	NM_006039	chr17	58058493	58124694	+	.	.	.	.	.	.	.	.	.	.	.	.	.	.	.	.	.	.	.	.
7149	0,25	ACE	NM_152830	chr17	58915909	58929473	+	.	.	.	.	.	.	.	.	.	.	.	.	.	.	.	.	.	.	.	.
6496	0,5	KCNH6	NM_173092	chr17	58954426	58980070	+	.	.	.	.	.	.	.	.	.	.	.	.	.	.	.	.	.	.	.	.
1516	2	MAP3K3	NM_203351	chr17	59053532	59127402	+	.	.	.	.	.	.	.	.	.	.	.	.	.	.	.	.	.	.	.	.
6262	0,5	DDX42	NM_007372	chr17	59205298	59250409	+	.	.	.	.	.	.	.	.	.	.	.	.	s855_149	chr17	59211563	-	.	.	.	.
3887	1	GH2	NM_002059	chr17	59311303	59313034	-	.	.	.	.	.	.	.	.	.	.	.	.	.	.	.	.	.	.	.	.
3886	1	GH1	NM_022562	chr17	59348294	59349930	-	.	.	.	.	.	.	.	.	.	.	.	.	.	.	.	.	.	.	.	.
3302	1	CD79B	NM_001039933	chr17	59359829	59363436	-	.	.	.	.	.	.	.	.	.	.	.	.	.	.	.	.	.	.	.	.
6462	0,5	ICAM2	NM_001099786	chr17	59433686	59451726	-	.	.	.	.	.	.	.	.	.	.	.	.	.	.	.	.	.	.	.	.
464	3,25	ERN1	NM_001433	chr17	59474121	59561234	-	CL799665	chr17	59510166	-	.	.	.	.	.	.	.	.	.	.	.	.	.	.	.	.
1950	1,75	PECAM1	NM_000442	chr17	59750508	59817743	-	.	.	.	.	.	.	.	.	.	.	.	.	.	.	.	.	.	.	.	.
4863	1	POLG2	NM_007215	chr17	59904363	59923646	-	.	.	.	.	.	.	.	.	.	.	.	.	.	.	.	.	.	.	.	.
2103	1,5	DDX5	NM_004396	chr17	59924835	59932946	-	.	.	.	.	.	.	.	.	.	.	.	.	s856_149	chr17	59925483	-	.	.	.	.
5301	1	SMURF2	NM_022739	chr17	59971196	60088848	-	.	.	.	.	.	.	.	.	.	.	.	.	.	.	.	.	.	.	.	.
3914	1	GNA13	NM_006572	chr17	60435868	60483382	-	.	.	.	.	.	.	.	.	.	.	.	.	.	.	.	.	.	.	.	.
1273	2	AXIN2	NM_004655	chr17	60955144	60988202	-	.	.	.	.	.	.	.	.	.	.	.	.	.	.	.	.	.	.	.	.
162	4,75	PRKCA	NM_002737	chr17	61729387	62237324	+	CL529488	chr17	61943204	+	AY516608	chr17	61937121	-	.	.	.	.	.	.	.	.	.	.	.	.
3266	1	CACNG5	NM_145811	chr17	62303852	62311857	+	.	.	.	.	.	.	.	.	.	.	.	.	.	.	.	.	.	.	.	.
3265	1	CACNG4	NM_014405	chr17	62391441	62459980	+	.	.	.	.	.	.	.	.	.	.	.	.	.	.	.	.	.	.	.	.
6426	0,5	HELZ	NM_014877	chr17	62497015	62671781	-	.	.	.	.	.	.	.	.	.	.	.	.	.	.	.	.	.	.	.	.
6064	0,5	BPTF	NM_004459	chr17	63252241	63410956	+	CL800118	chr17	63255285	-	.	.	.	.	.	.	.	.	.	.	.	.	.	.	.	.
6064	0,5	BPTF	NM_004459	chr17	63252241	63410956	+	CL529183	chr17	63302145	+	.	.	.	.	.	.	.	.	.	.	.	.	.	.	.	.
4243	1	KPNA2	NM_002266	chr17	63462309	63473432	+	.	.	.	.	.	.	.	.	.	.	.	.	.	.	.	.	.	.	.	.
418	3,5	PRKAR1A	NM_212471	chr17	64019704	64040505	+	.	.	.	.	.	.	.	.	.	.	.	.	.	.	.	.	.	.	.	.

Table S2

tumor associated genes								HIV				MLV				MMTV				MMTV(SIN)				MMTV(SIN)arrest			
rank	score	gene symbol	RefSEq	chrom	txStart	txEnd	strand	integrant	chr	position	strand	integrant	chr	position	strand	integrant	chr	position	strand	integrant	chr	position	strand	integrant	chr	position	strand
1983	1,5	ABCA8	NM_007168	chr17	64375025	64463128	-	.	.	.	.	.	.	.	.	.	.	.	.	.	.	.	.	.	.	.	.
1194	2	ABCA9	NM_080283	chr17	64482367	64568731	-	.	.	.	.	.	.	.	.	.	.	.	.	.	.	.	.	.	.	.	.
1192	2	ABCA6	NM_080284	chr17	64586441	64649610	-	CL800500	chr17	64607794	-	.	.	.	.	.	.	.	.	.	.	.	.	.	.	.	.
2849	1	ABCA10	NM_080282	chr17	64655742	64752551	-	.	.	.	.	.	.	.	.	.	.	.	.	.	.	.	.	.	.	.	.
516	3	ABCA5	NM_172232	chr17	64752170	64834918	-	CL529505	chr17	64817525	+	.	.	.	.	.	.	.	.	.	.	.	.	.	.	.	.
890	2,5	MAP2K6	NM_002758	chr17	64922432	65050065	+	.	.	.	.	.	.	.	.	.	.	.	.	.	.	.	.	.	.	.	.
4201	1	KCNJ16	NM_018658	chr17	65582960	65643341	+	.	.	.	.	.	.	.	.	.	.	.	.	.	.	.	.	.	.	.	.
7383	0,25	KCNJ2	NM_000891	chr17	65677270	65687778	+	.	.	.	.	.	.	.	.	.	.	.	.	.	.	.	.	.	.	.	.
5968	0,75	SOX9	NM_000346	chr17	67628755	67634155	+	.	.	.	.	.	.	.	.	.	.	.	.	.	.	.	.	.	.	.	.
6975	0,5	SSTR2	NM_001050	chr17	68672754	68679657	+	.	.	.	.	.	.	.	.	.	.	.	.	.	.	.	.	.	.	.	.
2359	1,5	RAB37	NM_001163990	chr17	70244950	70255069	+	.	.	.	.	.	.	.	.	.	.	.	.	99_1_491_18	chr17	70181582	+	.	.	.	.
6931	0,5	SLC9A3R1	NM_004252	chr17	70256345	70277094	+	.	.	.	.	.	.	.	.	.	.	.	.	.	.	.	.	.	.	.	.
6412	0,5	GRIN2C	NM_000835	chr17	70349762	70367602	-	.	.	.	.	.	.	.	.	.	.	.	.	.	.	.	.	.	.	.	.
3078	1	ATP5H	NM_006356	chr17	70546549	70554669	-	.	.	.	.	.	.	.	.	.	.	.	.	.	.	.	.	.	.	.	.
4631	1	NT5C	NM_014595	chr17	70637914	70639485	-	.	.	.	.	.	.	.	.	.	.	.	.	.	.	.	.	.	.	.	.
6437	0,5	HN1	NM_001002033	chr17	70642938	70662369	-	.	.	.	.	.	.	.	.	.	.	.	.	.	.	.	.	.	.	.	.
2297	1,5	NUP85	NM_024844	chr17	70713191	70743449	+	.	.	.	.	.	.	.	.	.	.	.	.	.	.	.	.	.	.	.	.
97	5,75	GRB2	NM_002086	chr17	70825751	70913384	-	CL799945	chr17	70832956	+	.	.	.	.	.	.	.	.	.	.	.	.	.	.	.	.
97	5,75	GRB2	NM_002086	chr17	70825751	70913384	-	CL800287	chr17	70892745	+	.	.	.	.	.	.	.	.	.	.	.	.	.	.	.	.
97	5,75	GRB2	NM_002086	chr17	70825751	70913384	-	CL800654	chr17	70886744	+	.	.	.	.	.	.	.	.	.	.	.	.	.	.	.	.
5091	1	RECQL5	NM_001003716	chr17	71157388	71174864	-	CL799837	chr17	71168737	-	.	.	.	.	.	.	.	.	.	.	.	.	.	.	.	.
5091	1	RECQL5	NM_001003716	chr17	71157388	71174864	-	AY517222.1	chr17	71159836	-	.	.	.	.	.	.	.	.	.	.	.	.	.	.	.	.
2209	1,5	ITGB4	NM_001005731	chr17	71229110	71265494	+	.	.	.	.	.	.	.	.	.	.	.	.	.	.	.	.	.	.	.	.
3840	1	GALK1	NM_000154	chr17	71265612	71272875	-	.	.	.	.	.	.	.	.	.	.	.	.	.	.	.	.	.	.	.	.
4001	1	H3F3B	NM_005324	chr17	71284109	71287455	-	.	.	.	.	.	.	.	.	.	.	.	.	.	.	.	.	.	.	.	.
2885	1	ACOX1	NM_004035	chr17	71449186	71487110	-	CL800570	chr17	71481113	-	.	.	.	.	.	.	.	.	.	.	.	.	.	.	.	.
3315	1	CDK3	NM_001258	chr17	71508581	71513675	+	.	.	.	.	.	.	.	.	.	.	.	.	.	.	.	.	.	.	.	.
3853	1	GALR2	NM_003857	chr17	71582486	71585168	+	.	.	.	.	.	.	.	.	.	.	.	.	.	.	.	.	.	.	.	.
2414	1,5	SPHK1	NM_001142601	chr17	71892284	71895536	+	.	.	.	.	.	.	.	.	.	.	.	.	.	.	.	.	.	.	.	.
699	3	UBE2O	NM_022066	chr17	71897207	71960883	-	.	.	.	.	AY516106	chr17	71953078	-	.	.	.	.	.	.	.	.	.	.	.	.
2844	1	AANAT	NM_001166579	chr17	71961027	71977794	+	.	.	.	.	.	.	.	.	.	.	.	.	.	.	.	.	.	.	.	.
6976	0,5	ST6GALNAC1	NM_018414	chr17	72132439	72151489	-	.	.	.	.	.	.	.	.	.	.	.	.	.	.	.	.	.	.	.	.
4191	1	JMJD6	NM_015167	chr17	72226119	72234476	-	.	.	.	.	.	.	.	.	.	.	.	.	.	.	.	.	.	.	.	.
2253	1,5	MGAT5B	NM_144677	chr17	72376392	72458066	+	.	.	.	.	.	.	.	.	.	.	.	.	.	.	.	.	.	.	.	.
6995	0,5	SYNGR2	NM_004710	chr17	73676265	73680604	+	.	.	.	.	.	.	.	.	.	.	.	.	.	.	.	.	.	.	.	.
349	3,75	TK1	NM_003258	chr17	73681754	73694880	-	CL529404	chr17	73690090	+	.	.	.	.	.	.	.	.	.	.	.	.	.	.	.	.
2919	1	AFMID	NM_001145526	chr17	73694992	73715377	+	.	.	.	.	.	.	.	.	.	.	.	.	.	.	.	.	.	.	.	.
171	4,5	BIRC5	NM_001012271	chr17	73721871	73733311	+	.	.	.	.	.	.	.	.	.	.	.	.	.	.	.	.	.	.	.	.
5965	0,75	SOCS3	NM_003955	chr17	73864456	73867753	-	.	.	.	.	.	.	.	.	.	.	.	.	.	.	.	.	.	.	.	.
6243	0,5	CYTH1	NM_017456	chr17	74181724	74289971	-	AY516929.1	chr17	74260473	-	.	.	.	.	.	.	.	.	.	.	.	.	.	.	.	.
1974	1,75	TIMP2	NM_003255	chr17	74360653	74433067	-	.	.	.	.	.	.	.	.	.	.	.	.	.	.	.	.	.	.	.	.
1091	2,25	LGALS3BP	NM_005567	chr17	74478929	74487656	-	.	.	.	.	.	.	.	.	.	.	.	.	.	.	.	.	.	.	.	.
3273	1	CANT1	NM_001159772	chr17	74499392	74517494	-	.	.	.	.	.	.	.	.	.	.	.	.	.	.	.	.	.	.	.	.
3678	1	ENGASE	NM_001042573	chr17	74582613	74596280	+	.	.	.	.	.	.	.	.	.	.	.	.	.	.	.	.	.	.	.	.
3685	1	ENPP7	NM_178543	chr17	75319476	75330616	+	.	.	.	.	.	.	.	.	.	.	.	.	.	.	.	.	.	.	.	.
3828	1	GAA	NM_000152	chr17	75689949	75708274	+	.	.	.	.	.	.	.	.	.	.	.	.	.	.	.	.	.	.	.	.
4602	1	NPTX1	NM_002522	chr17	76055227	76064999	-	.	.	.	.	.	.	.	.	.	.	.	.	.	.	.	.	.	.	.	.
6048	0,5	BAIAP2	NM_017451	chr17	76623541	76705827	+	.	.	.	.	.	.	.	.	.	.	.	.	.	.	.	.	.	.	.	.
1190	2	AATK	NM_001080395	chr17	76705690	76754467	-	.	.	.	.	.	.	.	.	.	.	.	.	.	.	.	.	.	.	.	.
4735	1	PDE6G	NR_026872	chr17	77227893	77234012	-	.	.	.	.	.	.	.	.	.	.	.	.	.	.	.	.	.	.	.	.
1447	2	HGS	NM_004712	chr17	77261366	77279556	+	CL800341	chr17	77276884	-	.	.	.	.	.	.	.	.	.	.	.	.	.	.	.	.

Table S2

tumor associated genes								HIV				MLV				MMTV				MMTV(SIN)				MMTV(SIN)arrest			
rank	score	gene symbol	RefSEq	chrom	txStart	txEnd	strand	integrant	chr	position	strand	integrant	chr	position	strand	integrant	chr	position	strand	integrant	chr	position	strand	integrant	chr	position	strand
2303	1,5	P4HB	NM_000918	chr17	77394322	77411833	-	CL799707	chr17	77405099	+	.	.	.	.	.	.	.	.	.	.	.	.	.	.	.	.
1245	2	ANAPC11	NM_001002246	chr17	77442894	77451655	+	.	.	.	.	.	.	.	.	.	.	.	.	.	.	.	.	.	.	.	.
5246	1	SIRT7	NM_016538	chr17	77463106	77469350	-	.	.	.	.	.	.	.	.	.	.	.	.	.	.	.	.	.	.	.	.
5012	1	PYCR1	NM_006907	chr17	77483559	77488259	-	.	.	.	.	.	.	.	.	.	.	.	.	.	.	.	.	.	.	.	.
1260	2	ASPSCR1	NM_001251888	chr17	77528714	77568571	+	.	.	.	.	AY516363	chr17	77554536	-	.	.	.	.	.	.	.	.	.	.	.	.
499	3,25	RAC3	NM_005052	chr17	77582820	77585369	+	.	.	.	.	.	.	.	.	.	.	.	.	.	.	.	.	.	.	.	.
3542	1	DCXR	NM_001195218	chr17	77587045	77588862	-	.	.	.	.	.	.	.	.	.	.	.	.	.	.	.	.	.	.	.	.
5104	1	RFNG	NM_002917	chr17	77599066	77602939	-	.	.	.	.	.	.	.	.	.	.	.	.	.	.	.	.	.	.	.	.
466	3,25	FASN	NM_004104	chr17	77629502	77649395	-	.	.	.	.	.	.	.	.	.	.	.	.	.	.	.	.	.	.	.	.
6902	0,5	SLC16A3	NM_004207	chr17	77780175	77790664	+	.	.	.	.	.	.	.	.	.	.	.	.	.	.	.	.	.	.	.	.
1328	2	CSNK1D	NM_139062	chr17	77793825	77824883	-	.	.	.	.	.	.	.	.	.	.	.	.	.	.	.	.	.	.	.	.
5035	1	RAB40B	NM_006822	chr17	78208231	78249887	-	.	.	.	.	.	.	.	.	.	.	.	.	.	.	.	.	.	.	.	.
3790	1	FN3K	NM_022158	chr17	78286740	78302362	+	.	.	.	.	.	.	.	.	.	.	.	.	.	.	.	.	.	.	.	.
5654	1	USP14	NM_005151	chr18	148482	203739	+	CL800411	chr18	157988	+	.	.	.	.	.	.	.	.	.	.	.	.	.	.	.	.
6205	0,5	COLEC12	NM_130386	chr18	309354	490729	-	.	.	.	.	.	.	.	.	.	.	.	.	.	.	.	.	.	.	.	.
513	3,25	TYMS	NM_001071	chr18	647603	663499	+	.	.	.	.	.	.	.	.	.	.	.	.	.	.	.	.	.	.	.	.
1864	2	YES1	NM_005433	chr18	711591	802327	-	CL800128	chr18	731101	+	.	.	.	.	.	.	.	.	.	.	.	.	.	.	.	.
5876	0,75	ADCYAP1	NM_001099733	chr18	894943	902173	+	.	.	.	.	.	.	.	.	.	.	.	.	.	.	.	.	.	.	.	.
1563	2	NDC80	NM_006101	chr18	2561509	2606634	+	.	.	.	.	.	.	.	.	.	.	.	.	.	.	.	.	.	.	.	.
2581	1,25	EPB41L3	NM_012307	chr18	5382387	5533986	-	.	.	.	.	.	.	.	.	.	.	.	.	.	.	.	.	.	.	.	.
271	4	LAMA1	NM_005559	chr18	6931742	7107813	-	.	.	.	.	.	.	.	.	.	.	.	.	.	.	.	.	.	.	.	.
659	3	PTPRM	NM_002845	chr18	7557313	8396859	+	.	.	.	.	AY516557	chr18	7861232	+	.	.	.	.	.	.	.	.	.	.	.	.
4553	1	NDUFV2	NM_021074	chr18	9092627	9124343	+	.	.	.	.	.	.	.	.	.	.	.	.	.	.	.	.	.	.	.	.
1152	2,25	RALBP1	NM_006788	chr18	9465529	9528106	+	.	.	.	.	.	.	.	.	.	.	.	.	.	.	.	.	.	.	.	.
4925	1	PPP4R1	NR_052003	chr18	9536788	9604605	-	CL800778	chr18	9562711	+	.	.	.	.	.	.	.	.	.	.	.	.	.	.	.	.
5029	1	RAB31	NM_006868	chr18	9698227	9852553	+	.	.	.	.	AY515999	chr18	9746081	-	.	.	.	.	.	.	.	.	.	.	.	.
2998	1	APCDD1	NM_153000	chr18	10444624	10478698	+	.	.	.	.	.	.	.	.	.	.	.	.	.	.	.	.	.	.	.	.
3917	1	GNAL	NM_001261444	chr18	11847436	11875683	+	.	.	.	.	.	.	.	.	.	.	.	.	.	.	.	.	.	.	.	.
599	3	IMPA2	NM_014214	chr18	11971426	12020885	+	.	.	.	.	.	.	.	.	.	.	.	.	.	.	.	.	.	.	.	.
6181	0,5	CIDEA	NM_001279	chr18	12244317	12267594	+	.	.	.	.	.	.	.	.	.	.	.	.	.	.	.	.	.	.	.	.
7070	0,5	TUBB6	NM_032525	chr18	12298256	12316568	+	.	.	.	.	.	.	.	.	.	.	.	.	.	.	.	.	.	.	.	.
2733	1,25	PTPN2	NM_080423	chr18	12775476	12874334	-	CL800583	chr18	12804153	-	.	.	.	.	.	.	.	.	.	.	.	.	.	.	.	.
2392	1,5	SEH1L	NM_001013437	chr18	12937982	12977536	+	.	.	.	.	.	.	.	.	.	.	.	.	.	.	.	.	.	.	.	.
6080	0,5	C18orf1	NM_181481	chr18	13208785	13642753	+	.	.	.	.	AY516819	chr18	13496909	-	.	.	.	.	.	.	.	.	2576_223_1	chr18	13419363	+
752	2,75	ROCK1	NM_005406	chr18	16783700	16945810	-	CL799655	chr18	16898163	+	.	.	.	.	.	.	.	.	.	.	.	.	.	.	.	.
752	2,75	ROCK1	NM_005406	chr18	16783700	16945810	-	CL529502	chr18	16839648	-	.	.	.	.	.	.	.	.	.	.	.	.	.	.	.	.
752	2,75	ROCK1	NM_005406	chr18	16783700	16945810	-	CL800830	chr18	16813412	-	.	.	.	.	.	.	.	.	.	.	.	.	.	.	.	.
3697	1	ESCO1	NM_052911	chr18	17363259	17434691	-	CL529593	chr18	17363623	-	.	.	.	.	.	.	.	.	.	.	.	.	.	.	.	.
580	3	GATA6	NM_005257	chr18	18003401	18036489	+	.	.	.	.	.	.	.	.	.	.	.	.	.	.	.	.	.	.	.	.
5070	1	RBBP8	NM_203292	chr18	18767836	18860447	+	CL800266	chr18	18813441	-	.	.	.	.	.	.	.	.	.	.	.	.	.	.	.	.
3256	1	CABLES1	NR_023359	chr18	18968525	19094432	+	.	.	.	.	.	.	.	.	.	.	.	.	.	.	.	.	.	.	.	.
5124	1	RIOK3	NM_003831	chr18	19286784	19317097	+	.	.	.	.	.	.	.	.	.	.	.	.	.	.	.	.	.	.	.	.
2644	1,25	LAMA3	NM_000227	chr18	19706981	19789027	+	CL800551	chr18	19747179	-	AY516771	chr18	19555003	-	.	.	.	.	.	.	.	.	.	.	.	.
7220	0,25	CABYR	NM_012189	chr18	19972952	19995562	+	.	.	.	.	.	.	.	.	.	.	.	.	.	.	.	.	.	.	.	.
6708	0,5	OSBPL1A	NM_018030	chr18	19996008	20106194	-	.	.	.	.	.	.	.	.	.	.	.	.	.	.	.	.	.	.	.	.
1871	2	ZNF521	NM_015461	chr18	20895885	21186212	-	.	.	.	.	.	.	.	.	.	.	.	.	.	.	.	.	.	.	.	.
430	3,5	SS18	NM_005637	chr18	21850214	21924609	-	.	.	.	.	.	.	.	.	.	.	.	.	.	.	.	.	.	.	.	.
6027	0,5	AQP4	NM_004028	chr18	22686004	22696573	-	.	.	.	.	.	.	.	.	.	.	.	.	.	.	.	.	.	.	.	.
1891	1,75	CDH2	NM_001792	chr18	23784927	24011443	-	.	.	.	.	AY516847	chr18	23947608	-	.	.	.	.	.	.	.	.	.	.	.	.
5902	0,75	DSC2	NM_004949	chr18	26899939	26936386	-	.	.	.	.	.	.	.	.	.	.	.	.	.	.	.	.	.	.	.	.
6290	0,5	DSG1	NM_001942	chr18	27152049	27191391	+	.	.	.	.	.	.	.	.	.	.	.	.	.	.	.	.	.	.	.	.

Table S2

tumor associated genes								HIV				MLV				MMTV				MMTV(SIN)				MMTV(SIN)arrest			
rank	score	gene symbol	RefSEq	chrom	txStart	txEnd	strand	integrant	chr	position	strand	integrant	chr	position	strand	integrant	chr	position	strand	integrant	chr	position	strand	integrant	chr	position	strand
3608	1	DSG4	NM_177986	chr18	27210737	27247878	+	.	.	.	.	.	.	.	.	.	.	.	.	.	.	.	.	.	.	.	.
3607	1	DSG2	NM_001943	chr18	27332024	27382812	+	CL800714	chr18	27356404	-	.	.	.	.	.	.	.	.	.	.	.	.	.	.	.	.
2813	1,25	TTR	NM_000371	chr18	27425727	27432984	+	.	.	.	.	.	.	.	.	.	.	.	.	.	.	.	.	.	.	.	.
2021	1,5	B4GALT6	NM_004775	chr18	27456206	27518684	-	.	.	.	.	.	.	.	.	.	.	.	.	.	.	.	.	.	.	.	.
5135	1	RNF125	NM_017831	chr18	27852442	27907152	+	.	.	.	.	.	.	.	.	.	.	.	.	.	.	.	.	.	.	.	.
6607	0,5	MEP1B	NM_005925	chr18	28023984	28054364	+	.	.	.	.	.	.	.	.	.	.	.	.	.	.	.	.	.	.	.	.
4595	1	NOL4	NM_003787	chr18	29685061	30057513	-	.	.	.	.	.	.	.	.	.	.	.	.	.	.	.	.	.	.	.	.
7626	0,25	ZNF24	NM_006965	chr18	31166175	31178424	-	.	.	.	.	.	.	.	.	.	.	.	.	.	.	.	.	.	.	.	.
3844	1	GALNT1	NM_020474	chr18	31488530	31545796	+	AY517313.1	chr18	31524306	+	.	.	.	.	.	.	.	.	.	.	.	.	.	.	.	.
5277	1	SLC39A6	NM_012319	chr18	31942491	31963355	-	.	.	.	.	.	.	.	.	.	.	.	.	.	.	.	.	.	.	.	.
3779	1	FHOD3	NM_025135	chr18	32131699	32614016	+	.	.	.	.	.	.	.	.	.	.	.	.	.	.	.	.	.	.	.	.
222	4,25	PIK3C3	NM_002647	chr18	37789196	37915444	+	CL800520	chr18	37808304	-	.	.	.	.	.	.	.	.	.	.	.	.	.	.	.	.
1159	2,25	RIT2	NM_002930	chr18	38577189	38949655	-	.	.	.	.	.	.	.	.	.	.	.	.	.	.	.	.	.	.	.	.
7003	0,5	SYT4	NM_020783	chr18	39101854	39111613	-	.	.	.	.	.	.	.	.	.	.	.	.	.	.	.	.	.	.	.	.
5959	0,75	SLC14A1	NM_001146037	chr18	41560915	41586483	+	.	.	.	.	.	.	.	.	.	.	.	.	.	.	.	.	.	.	.	.
4977	1	PSTPIP2	NM_024430	chr18	41817499	41906248	-	.	.	.	.	.	.	.	.	.	.	.	.	.	.	.	.	.	.	.	.
3071	1	ATP5A1	NM_001001935	chr18	41918107	41932317	-	.	.	.	.	.	.	.	.	.	.	.	.	.	.	.	.	.	.	.	.
6081	0,5	C18orf25	NM_001008239	chr18	42007985	42100953	+	.	.	.	.	.	.	.	.	.	.	.	.	.	.	.	.	.	.	.	.
2789	1,25	ST8SIA5	NM_013305	chr18	42513078	42591037	-	.	.	.	.	.	.	.	.	.	.	.	.	.	.	.	.	.	.	.	.
4794	1	PIAS2	NM_173206	chr18	42649670	42751493	-	.	.	.	.	.	.	.	.	_291_1_187	chr18	42703214	-	.	.	.	.	.	.	.	.
163	4,75	SMAD2	NM_005901	chr18	43613463	43710968	-	CL799875	chr18	43677800	+	.	.	.	.	.	.	.	.	.	.	.	.	.	.	.	.
163	4,75	SMAD2	NM_005901	chr18	43613463	43710968	-	CL529741	chr18	43698414	-	.	.	.	.	.	.	.	.	.	.	.	.	.	.	.	.
163	4,75	SMAD2	NM_005901	chr18	43613463	43710968	-	CL529185	chr18	43678924	-	.	.	.	.	.	.	.	.	.	.	.	.	.	.	.	.
163	4,75	SMAD2	NM_005901	chr18	43613463	43710968	-	CL529663	chr18	43662303	+	.	.	.	.	.	.	.	.	.	.	.	.	.	.	.	.
682	3	SMAD7	NM_001190823	chr18	44700220	44723175	-	.	.	.	.	.	.	.	.	.	.	.	.	.	.	.	.	3226_320_1	chr18	44723645	-
2865	1	ACAA2	NM_006111	chr18	45563871	45594249	-	.	.	.	.	.	.	.	.	.	.	.	.	.	.	.	.	.	.	.	.
2666	1,25	MBD1	NM_001204139	chr18	46047249	46062142	-	.	.	.	.	.	.	.	.	.	.	.	.	.	.	.	.	.	.	.	.
7263	0,25	CXXC1	NM_014593	chr18	46062710	46068690	-	.	.	.	.	.	.	.	.	.	.	.	.	.	.	.	.	.	.	.	.
2661	1,25	MAPK4	NM_002747	chr18	46340481	46512194	+	.	.	.	.	.	.	.	.	.	.	.	.	.	.	.	.	.	.	.	.
4373	1	ME2	NM_002396	chr18	46659429	46730160	+	CL529719	chr18	46723255	+	.	.	.	.	.	.	.	.	.	.	.	.	.	.	.	.
26	8,75	SMAD4	NM_005359	chr18	46810580	46865409	+	.	.	.	.	.	.	.	.	.	.	.	.	.	.	.	.	.	.	.	.
1029	2,25	DCC	NM_005215	chr18	48120539	49316271	+	.	.	.	.	.	.	.	.	.	.	.	.	.	.	.	.	.	.	.	.
893	2,5	MBD2	NM_003927	chr18	49931968	50005156	-	CL799877	chr18	49981421	+	.	.	.	.	.	.	.	.	.	.	.	.	.	.	.	.
2717	1,25	POLI	NM_007195	chr18	50049846	50078602	+	.	.	.	.	.	.	.	.	.	.	.	.	.	.	.	.	.	.	.	.
5025	1	RAB27B	NM_004163	chr18	50646705	50713745	+	.	.	.	.	.	.	.	.	.	.	.	.	.	.	.	.	.	.	.	.
5445	1	TCF4	NM_001243228	chr18	51040559	51408043	-	CL799902	chr18	51400457	-	.	.	.	.	.	.	.	.	.	.	.	.	.	.	.	.
7097	0,5	WDR7	NM_015285	chr18	52469613	52848034	+	CL529407	chr18	52809885	-	.	.	.	.	.	.	.	.	.	.	.	.	2931_264_1	chr18	52796189	-
7097	0,5	WDR7	NM_015285	chr18	52469613	52848034	+	CL799568	chr18	52635770	-	.	.	.	.	.	.	.	.	.	.	.	.	2931_264_1	chr18	52796189	-
6701	0,5	ONECUT2	NM_004852	chr18	53253914	53309528	+	.	.	.	.	.	.	.	.	.	.	.	.	.	.	.	.	.	.	.	.
3769	1	FECH	NM_000140	chr18	53363070	53404967	-	.	.	.	.	.	.	.	.	.	.	.	.	.	.	.	.	.	.	.	.
4496	1	NARS	NM_004539	chr18	53418891	53440175	-	.	.	.	.	.	.	.	.	.	.	.	.	.	.	.	.	.	.	.	.
3098	1	ATP8B1	NM_005603	chr18	53464656	53621325	-	.	.	.	.	.	.	.	.	.	.	.	.	.	.	.	.	.	.	.	.
2275	1,5	NEDD4L	NM_001144967	chr18	53862607	54219752	+	.	.	.	.	.	.	.	.	.	.	.	.	.	.	.	.	.	.	.	.
990	2,25	ALPK2	NM_052947	chr18	54299461	54447169	-	.	.	.	.	.	.	.	.	.	.	.	.	.	.	.	.	.	.	.	.
613	3	MALT1	NM_006785	chr18	54489597	54568350	+	AY517081.1	chr18	54543774	+	.	.	.	.	.	.	.	.	.	.	.	.	.	.	.	.
2607	1,25	GRP	NM_002091	chr18	55038379	55048982	+	.	.	.	.	.	.	.	.	.	.	.	.	.	.	.	.	.	.	.	.
6561	0,5	LMAN1	NM_005570	chr18	55146035	55177488	-	.	.	.	.	.	.	.	.	.	.	.	.	.	.	.	.	.	.	.	.
6600	0,5	MC4R	NM_005912	chr18	56189543	56190981	-	.	.	.	.	.	.	.	.	.	.	.	.	.	.	.	.	.	.	.	.
3309	1	CDH20	NM_031891	chr18	57308754	57373345	+	.	.	.	.	.	.	.	.	.	.	.	.	.	.	.	.	2283_198_1	chr18	57356219	-
1621	2	PIGN	NM_012327	chr18	57862437	58005269	-	.	.	.	.	.	.	.	.	.	.	.	.	.	.	.	.	.	.	.	.
7038	0,5	TNFRSF11A	NM_003839	chr18	58143527	58204484	+	.	.	.	.	.	.	.	.	.	.	.	.	.	.	.	.	.	.	.	.

Table S2

tumor associated genes								HIV				MLV				MMTV				MMTV(SIN)				MMTV(SIN)arrest			
rank	score	gene symbol	RefSEq	chrom	txStart	txEnd	strand	integrant	chr	position	strand	integrant	chr	position	strand	integrant	chr	position	strand	integrant	chr	position	strand	integrant	chr	position	strand
129	5	BCL2	NM_000657	chr18	59136166	59137593	-	.	.	.	.	AY516170	chr18	59056337	-	.	.	.	.	.	.	.	.	.	.	.	.
607	3	KDSR	NM_002035	chr18	59145950	59185486	-	.	.	.	.	.	.	.	.	.	.	.	.	.	.	.	.	.	.	.	.
951	2,5	SERPINB5	NM_002639	chr18	59295123	59323298	+	.	.	.	.	.	.	.	.	.	.	.	.	.	.	.	.	.	.	.	.
7538	0,25	SERPINB13	NM_012397	chr18	59405513	59417413	+	.	.	.	.	.	.	.	.	.	.	.	.	.	.	.	.	.	.	.	.
7539	0,25	SERPINB4	NM_002974	chr18	59455472	59462482	-	.	.	.	.	.	.	.	.	.	.	.	.	.	.	.	.	.	.	.	.
2766	1,25	SERPINB3	NM_006919	chr18	59473410	59480177	-	.	.	.	.	.	.	.	.	.	.	.	.	.	.	.	.	.	.	.	.
5889	0,75	CDH19	NM_021153	chr18	62322300	62422196	-	.	.	.	.	.	.	.	.	.	.	.	.	.	.	.	.	.	.	.	.
6283	0,5	DOK6	NM_152721	chr18	65219263	65667302	+	.	.	.	.	.	.	.	.	.	.	.	.	.	.	.	.	.	.	.	.
2410	1,5	SOCS6	NM_004232	chr18	66107116	66148414	+	.	.	.	.	.	.	.	.	.	.	.	.	.	.	.	.	.	.	.	.
6135	0,5	CBLN2	NM_182511	chr18	68354894	68362703	-	.	.	.	.	.	.	.	.	.	.	.	.	.	.	.	.	.	.	.	.
4566	1	NETO1	NM_138999	chr18	68652254	68683914	-	.	.	.	.	.	.	.	.	.	.	.	.	.	.	.	.	.	.	.	.
2079	1,5	CNDP1	NM_032649	chr18	70352671	70403241	+	.	.	.	.	.	.	.	.	.	.	.	.	.	.	.	.	.	.	.	.
1936	1,75	MBP	NM_001025100	chr18	72853726	72973762	-	.	.	.	.	AY516643	chr18	72968897	-	.	.	.	.	.	.	.	.	.	.	.	.
2150	1,5	GALR1	NM_001480	chr18	73090995	73111084	+	.	.	.	.	.	.	.	.	.	.	.	.	.	.	.	.	.	.	.	.
905	2,5	NFATC1	NM_172387	chr18	75261313	75390311	+	.	.	.	.	.	.	.	.	.	.	.	.	.	.	.	.	.	.	.	.
649	3	PPAP2C	NM_003712	chr19	232043	242435	-	.	.	.	.	.	.	.	.	.	.	.	.	.	.	.	.	.	.	.	.
2257	1,5	MIER2	NM_017550	chr19	256574	295791	-	.	.	.	.	.	.	.	.	.	.	.	.	.	.	.	.	.	.	.	.
955	2,5	SHC2	NM_012435	chr19	367582	411996	-	.	.	.	.	.	.	.	.	.	.	.	.	.	.	.	.	.	.	.	.
1299	2	CDC34	NM_004359	chr19	482732	493087	+	.	.	.	.	.	.	.	.	.	.	.	.	.	.	.	.	.	.	.	.
445	3,25	BSG	NM_198591	chr19	522276	534493	+	.	.	.	.	.	.	.	.	.	.	.	.	.	.	.	.	.	.	.	.
928	2,5	POLRMT	NM_005035	chr19	568222	584568	-	.	.	.	.	.	.	.	.	.	.	.	.	.	.	.	.	.	.	.	.
5136	1	RNF126	NM_194460	chr19	598525	614233	-	.	.	.	.	.	.	.	.	.	.	.	.	.	.	.	.	.	.	.	.
837	2,5	FSTL3	NM_005860	chr19	627388	634392	+	.	.	.	.	.	.	.	.	.	.	.	.	.	.	.	.	.	.	.	.
421	3,5	PTBP1	NM_002819	chr19	748391	763327	+	.	.	.	.	.	.	.	.	.	.	.	.	.	.	.	.	.	.	.	.
1893	1,75	CFD	NM_001928	chr19	810664	814610	+	.	.	.	.	.	.	.	.	.	.	.	.	.	.	.	.	.	.	.	.
4376	1	MED16	NM_005481	chr19	818961	844218	-	.	.	.	.	.	.	.	.	.	.	.	.	.	.	.	.	.	.	.	.
4231	1	KISS1R	NM_032551	chr19	868341	872015	+	.	.	.	.	.	.	.	.	.	.	.	.	.	.	.	.	.	.	.	.
5690	1	WDR18	NM_024100	chr19	935327	945569	+	.	.	.	.	.	.	.	.	.	.	.	.	.	.	.	.	.	.	.	.
2036	1,5	C19orf6	NM_033420	chr19	960649	972141	-	.	.	.	.	.	.	.	.	.	.	.	.	.	.	.	.	.	.	.	.
6194	0,5	CNN2	NM_201277	chr19	977297	990064	+	.	.	.	.	.	.	.	.	.	.	.	.	.	.	.	.	.	.	.	.
1193	2	ABCA7	NM_019112	chr19	991101	1016570	+	AY517303.1	chr19	1009591	-	.	.	.	.	.	.	.	.	.	.	.	.	.	.	.	.
417	3,5	POLR2E	NM_002695	chr19	1037577	1046391	-	.	.	.	.	.	.	.	.	.	.	.	.	.	.	.	.	.	.	.	.
1062	2,25	GPX4	NM_002085	chr19	1054935	1057787	+	.	.	.	.	.	.	.	.	.	.	.	.	.	.	.	.	.	.	.	.
5189	1	SBNO2	NM_001100122	chr19	1058632	1083273	-	.	.	.	.	.	.	.	.	.	.	.	.	.	.	.	.	.	.	.	.
19	9,5	STK11	NM_000455	chr19	1156797	1179434	+	CL529189	chr19	1175041	+	.	.	.	.	.	.	.	.	.	.	.	.	.	.	.	.
3205	1	C19orf26	NM_152769	chr19	1180946	1188990	-	.	.	.	.	.	.	.	.	.	.	.	.	.	.	.	.	.	.	.	.
998	2,25	ATP5D	NM_001001975	chr19	1192748	1195824	+	.	.	.	.	.	.	.	.	.	.	.	.	.	.	.	.	.	.	.	.
4399	1	MIDN	NM_177401	chr19	1199551	1210142	+	.	.	.	.	.	.	.	.	.	.	.	.	.	.	.	.	.	.	.	.
3371	1	CIRBP	NM_001280	chr19	1220266	1224171	+	.	.	.	.	.	.	.	.	.	.	.	.	.	.	.	.	.	.	.	.
3203	1	C19orf24	NM_017914	chr19	1226519	1230243	+	.	.	.	.	.	.	.	.	.	.	.	.	.	.	.	.	.	.	.	.
4461	1	MUM1	NM_032853	chr19	1305975	1329430	+	CL800214	chr19	1326362	-	.	.	.	.	.	.	.	.	.	.	.	.	.	.	.	.
4461	1	MUM1	NM_032853	chr19	1305975	1329430	+	CL799534	chr19	1316007	-	.	.	.	.	.	.	.	.	.	.	.	.	.	.	.	.
4461	1	MUM1	NM_032853	chr19	1305975	1329430	+	CL800031	chr19	1308054	-	.	.	.	.	.	.	.	.	.	.	.	.	.	.	.	.
4461	1	MUM1	NM_032853	chr19	1305975	1329430	+	CL800278	chr19	1309562	-	.	.	.	.	.	.	.	.	.	.	.	.	.	.	.	.
4461	1	MUM1	NM_032853	chr19	1305975	1329430	+	CL799587	chr19	1311472	+	.	.	.	.	.	.	.	.	.	.	.	.	.	.	.	.
1570	2	NDUFS7	NM_024407	chr19	1334882	1346588	+	.	.	.	.	.	.	.	.	.	.	.	.	.	.	.	.	.	.	.	.
1421	2	GAMT	NM_000156	chr19	1348024	1352569	-	.	.	.	.	.	.	.	.	.	.	.	.	.	.	.	.	.	.	.	.
5163	1	RPS15	NM_001018	chr19	1389362	1391492	+	.	.	.	.	.	.	.	.	.	.	.	.	.	.	.	.	.	.	.	.
200	4,25	APC2	NM_005883	chr19	1401147	1424243	+	.	.	.	.	.	.	.	.	.	.	.	.	.	.	.	.	.	.	.	.
3204	1	C19orf25	NM_152482	chr19	1424199	1430228	-	.	.	.	.	.	.	.	.	.	.	.	.	.	.	.	.	.	.	.	.
4720	1	PCSK4	NM_017573	chr19	1432426	1441407	-	.	.	.	.	.	.	.	.	.	.	.	.	.	.	.	.	.	.	.	.

Table S2

tumor associated genes								HIV				MLV				MMTV				MMTV(SIN)				MMTV(SIN)arrest			
rank	score	gene symbol	RefSEq	chrom	txStart	txEnd	strand	integrant	chr	position	strand	integrant	chr	position	strand	integrant	chr	position	strand	integrant	chr	position	strand	integrant	chr	position	strand
5093	1	REEP6	NM_138393	chr19	1442164	1448924	+	.	.	.	.	.	.	.	.	.	.	.	.	.	.	.	.	.	.	.	.
2905	1	ADAMTSL5	NM_213604	chr19	1456016	1464188	-	.	.	.	.	.	.	.	.	.	.	.	.	.	.	.	.	.	.	.	.
4386	1	MEX3D	NM_001174118	chr19	1505667	1519057	-	.	.	.	.	.	.	.	.	.	.	.	.	.	.	.	.	.	.	.	.
2667	1,25	MBD3	NM_003926	chr19	1527677	1543652	-	CL799829	chr19	1540440	+	.	.	.	.	.	.	.	.	.	.	.	.	.	.	.	.
56	7	TCF3	NM_001136139	chr19	1560288	1601286	-	.	.	.	.	.	.	.	.	.	.	.	.	.	.	.	.	.	.	.	.
3099	1	ATP8B3	NM_138813	chr19	1733073	1763275	-	.	.	.	.	.	.	.	.	.	.	.	.	.	.	.	.	.	.	.	.
3716	1	FAM108A1	NM_001130111	chr19	1827974	1836518	-	.	.	.	.	.	.	.	.	.	.	.	.	.	.	.	.	.	.	.	.
2388	1,5	SCAMP4	NM_079834	chr19	1856372	1877012	+	.	.	.	.	.	.	.	.	.	.	.	.	.	.	.	.	.	.	.	.
245	4	CSNK1G2	NM_001319	chr19	1892160	1932336	+	CL529381	chr19	1915415	+	.	.	.	.	.	.	.	.	.	.	.	.	.	.	.	.
245	4	CSNK1G2	NM_001319	chr19	1892160	1932336	+	CL799767	chr19	1900327	+	.	.	.	.	.	.	.	.	.	.	.	.	.	.	.	.
245	4	CSNK1G2	NM_001319	chr19	1892160	1932336	+	CL799524	chr19	1912320	+	.	.	.	.	.	.	.	.	.	.	.	.	.	.	.	.
2029	1,5	BTBD2	NM_017797	chr19	1936446	1966702	-	CL800817	chr19	1954848	-	.	.	.	.	.	.	.	.	.	.	.	.	.	.	.	.
1533	2	MKNK2	NM_017572	chr19	1988469	2002243	-	.	.	.	.	.	.	.	.	.	.	.	.	.	.	.	.	.	.	.	.
2001	1,5	AP3D1	NM_001261826	chr19	2051986	2102556	-	.	.	.	.	.	.	.	.	.	.	.	.	.	.	.	.	.	.	.	.
103	5,5	DOT1L	NM_032482	chr19	2115147	2183577	+	AY517172.1	chr19	2156719	+	.	.	.	.	.	.	.	.	.	.	.	.	.	.	.	.
4841	1	PLEKHJ1	NM_018049	chr19	2184154	2187328	-	.	.	.	.	.	.	.	.	.	.	.	.	.	.	.	.	.	.	.	.
5217	1	SF3A2	NM_007165	chr19	2187815	2199678	+	.	.	.	.	.	.	.	.	.	.	.	.	.	.	.	.	.	.	.	.
782	2,5	AMH	NM_000479	chr19	2200112	2203072	+	.	.	.	.	.	.	.	.	.	.	.	.	.	.	.	.	.	.	.	.
1124	2,25	OAZ1	NM_004152	chr19	2220519	2224487	+	.	.	.	.	.	.	.	.	.	.	.	.	.	.	.	.	.	.	.	.
4312	1	LSM7	NM_016199	chr19	2272519	2279614	-	.	.	.	.	.	.	.	.	.	.	.	.	.	.	.	.	.	.	.	.
2415	1,5	SPPL2B	NM_152988	chr19	2279628	2306100	+	.	.	.	.	.	.	.	.	.	.	.	.	.	.	.	.	.	.	.	.
5470	1	TIMM13	NM_012458	chr19	2376621	2378875	-	.	.	.	.	.	.	.	.	.	.	.	.	.	.	.	.	.	.	.	.
881	2,5	LMNB2	NM_032737	chr19	2379162	2407966	-	.	.	.	.	.	.	.	.	.	.	.	.	.	.	.	.	.	.	.	.
2595	1,25	GADD45B	NM_015675	chr19	2427122	2429257	+	.	.	.	.	.	.	.	.	.	.	.	.	.	.	.	.	.	.	.	.
2166	1,5	GNG7	NM_052847	chr19	2462217	2653746	-	CL529382	chr19	2510785	+	AY516052	chr19	2483202	+	.	.	.	.	.	.	.	.	.	.	.	.
2166	1,5	GNG7	NM_052847	chr19	2462217	2653746	-	CL800175	chr19	2548669	+	AY516052	chr19	2483202	+	.	.	.	.	.	.	.	.	.	.	.	.
3583	1	DIRAS1	NM_145173	chr19	2665564	2672390	-	.	.	.	.	.	.	.	.	.	.	.	.	.	.	.	.	.	.	.	.
5276	1	SLC39A3	NM_144564	chr19	2683522	2691074	-	.	.	.	.	.	.	.	.	.	.	.	.	.	.	.	.	.	.	.	.
5228	1	SGTA	NM_003021	chr19	2705711	2734354	-	.	.	.	.	.	.	.	.	.	.	.	.	.	.	.	.	.	.	.	.
5468	1	THOP1	NM_003249	chr19	2736505	2764599	+	BH609813	chr19	2739379	+	.	.	.	.	.	.	.	.	.	.	.	.	.	.	.	.
2439	1,5	TLE2	NM_003260	chr19	2948635	2980165	-	.	.	.	.	.	.	.	.	.	.	.	.	.	.	.	.	.	.	.	.
2917	1	AES	NM_001130	chr19	3003907	3013371	-	.	.	.	.	.	.	.	.	.	.	.	.	.	.	.	.	.	.	.	.
2161	1,5	GNA11	NM_002067	chr19	3045407	3072454	+	.	.	.	.	.	.	.	.	.	.	.	.	.	.	.	.	.	.	.	.
2162	1,5	GNA15	NM_002068	chr19	3087190	3114766	+	.	.	.	.	.	.	.	.	.	.	.	.	.	.	.	.	.	.	.	.
6653	0,5	NCLN	NM_020170	chr19	3136874	3160573	+	.	.	.	.	.	.	.	.	.	.	.	.	.	.	.	.	.	.	.	.
6368	0,5	FZR1	NM_016263	chr19	3457294	3487755	+	.	.	.	.	.	.	.	.	.	.	.	.	.	.	.	.	.	.	.	.
2187	1,5	HMG20B	NM_006339	chr19	3523942	3530081	+	.	.	.	.	.	.	.	.	.	.	.	.	.	.	.	.	.	.	.	.
7579	0,25	TBXA2R	NM_001060	chr19	3545503	3557831	-	.	.	.	.	AY516367	chr19	3557124	-	.	.	.	.	.	.	.	.	.	.	.	.
7579	0,25	TBXA2R	NM_001060	chr19	3545503	3557831	-	.	.	.	.	AY516492	chr19	3555330	-	.	.	.	.	.	.	.	.	.	.	.	.
1628	2	PIP5K1C	NM_001195733	chr19	3581178	3651477	-	.	.	.	.	.	.	.	.	.	.	.	.	.	.	.	.	.	.	.	.
6023	0,5	APBA3	NM_004886	chr19	3701770	3712673	-	.	.	.	.	.	.	.	.	.	.	.	.	.	.	.	.	.	.	.	.
619	3	MATK	NM_139355	chr19	3728966	3737415	-	.	.	.	.	.	.	.	.	.	.	.	.	.	.	.	.	.	.	.	.
7122	0,5	ZFR2	NM_001145640	chr19	3802810	3820027	-	.	.	.	.	.	.	.	.	.	.	.	.	.	.	.	.	.	.	.	.
6035	0,5	ATCAY	NM_033064	chr19	3831617	3879080	+	.	.	.	.	.	.	.	.	.	.	.	.	.	.	.	.	.	.	.	.
2564	1,25	DAPK3	NM_001348	chr19	3909451	3920826	-	.	.	.	.	.	.	.	.	.	.	.	.	.	.	.	.	.	.	.	.
6297	0,5	EEF2	NM_001961	chr19	3927053	3936461	-	.	.	.	.	.	.	.	.	.	.	.	.	.	.	.	.	.	.	.	.
2711	1,25	PIAS4	NM_015897	chr19	3958748	3989067	+	.	.	.	.	.	.	.	.	.	.	.	.	.	.	.	.	.	.	.	.
1099	2,25	MAP2K2	NM_030662	chr19	4041319	4075126	-	BH609816	chr19	4068029	+	.	.	.	.	.	.	.	.	.	.	.	.	.	.	.	.
5245	1	SIRT6	NM_016539	chr19	4125105	4133596	-	.	.	.	.	.	.	.	.	.	.	.	.	.	.	.	.	.	.	.	.
6014	0,5	ANKRD24	NM_133475	chr19	4134350	4175811	+	.	.	.	.	.	.	.	.	.	.	.	.	.	.	.	.	.	.	.	.
1754	2	SH3GL1	NM_001199943	chr19	4311363	4351565	-	.	.	.	.	.	.	.	.	.	.	.	.	.	.	.	.	.	.	.	.

Table S2

tumor associated genes								HIV				MLV				MMTV				MMTV(SIN)				MMTV(SIN)arrest			
rank	score	gene symbol	RefSEq	chrom	txStart	txEnd	strand	integrant	chr	position	strand	integrant	chr	position	strand	integrant	chr	position	strand	integrant	chr	position	strand	integrant	chr	position	strand
3346	1	CHAF1A	NM_005483	chr19	4353659	4394394	+	BH609815	chr19	4388981	-	.	.	.	.	.	.	.	.	.	.	.	.	1004_126_1	chr19	4390600	+
7612	0,25	UHRF1	NM_001048201	chr19	4860509	4913165	+	.	.	.	.	.	.	.	.	.	.	.	.	.	.	.	.	.	.	.	.
872	2,5	KDM4B	NM_015015	chr19	4920123	5104608	+	.	.	.	.	.	.	.	.	.	.	.	.	.	.	.	.	.	.	.	.
939	2,5	PTPRS	NM_130853	chr19	5156518	5291814	-	.	.	.	.	.	.	.	.	.	.	.	.	.	.	.	.	.	.	.	.
6845	0,5	SAFB	NM_002967	chr19	5574045	5619489	+	CL799948	chr19	5588970	-	.	.	.	.	.	.	.	.	.	.	.	.	.	.	.	.
3821	1	FUT6	NM_000150	chr19	5781636	5790742	-	.	.	.	.	.	.	.	.	.	.	.	.	.	.	.	.	.	.	.	.
3818	1	FUT3	NM_001097640	chr19	5793898	5802485	-	.	.	.	.	.	.	.	.	.	.	.	.	.	.	.	.	.	.	.	.
3820	1	FUT5	NM_002034	chr19	5816836	5821551	-	.	.	.	.	.	.	.	.	.	.	.	.	.	.	.	.	.	.	.	.
4524	1	NDUFA11	NM_175614	chr19	5845680	5855024	-	.	.	.	.	.	.	.	.	.	.	.	.	.	.	.	.	.	.	.	.
2373	1,5	RFX2	NM_134433	chr19	5944174	6061664	-	CL800272	chr19	6032092	+	.	.	.	.	.	.	.	.	.	.	.	.	.	.	.	.
2373	1,5	RFX2	NM_134433	chr19	5944174	6061664	-	CL800310	chr19	6034463	-	.	.	.	.	.	.	.	.	.	.	.	.	.	.	.	.
1536	2	MLLT1	NM_005934	chr19	6161391	6230959	-	CL800224	chr19	6202369	+	AY516580	chr19	6225234	-	.	.	.	.	.	.	.	.	.	.	.	.
6416	0,5	GTF2F1	NM_002096	chr19	6330579	6344291	-	.	.	.	.	.	.	.	.	_423_1_263	chr19	6342368	-	.	.	.	.	.	.	.	.
6514	0,5	KHSRP	NM_003685	chr19	6364118	6375822	-	.	.	.	.	.	.	.	.	.	.	.	.	.	.	.	.	.	.	.	.
6911	0,5	SLC25A41	NM_173637	chr19	6377047	6384790	-	.	.	.	.	.	.	.	.	.	.	.	.	.	.	.	.	.	.	.	.
6910	0,5	SLC25A23	NM_024103	chr19	6391074	6410781	-	.	.	.	.	.	.	.	.	.	.	.	.	.	.	.	.	.	.	.	.
5513	1	TNFSF9	NM_003811	chr19	6482009	6486939	+	.	.	.	.	.	.	.	.	.	.	.	.	.	.	.	.	.	.	.	.
6155	0,5	CD70	NM_001252	chr19	6536849	6542163	-	.	.	.	.	.	.	.	.	.	.	.	.	.	.	.	.	.	.	.	.
1180	2,25	VAV1	NM_001258207	chr19	6723678	6808377	+	CL529418	chr19	6789529	+	AY516518	chr19	6782061	+	.	.	.	.	.	.	.	.	.	.	.	.
216	4,25	INSR	NM_001079817	chr19	7063265	7245011	-	CL799733	chr19	7109490	+	.	.	.	.	.	.	.	.	.	.	.	.	.	.	.	.
6029	0,5	ARHGEF18	NM_001130955	chr19	7410573	7443371	+	CL529190	chr19	7400503	-	.	.	.	.	.	.	.	.	.	.	.	.	.	.	.	.
5711	1	XAB2	NM_020196	chr19	7590410	7600439	-	.	.	.	.	.	.	.	.	.	.	.	.	.	.	.	.	.	.	.	.
2425	1,5	STXBP2	NM_001127396	chr19	7607990	7618759	+	.	.	.	.	.	.	.	.	.	.	.	.	.	.	.	.	.	.	.	.
6190	0,5	CLEC4G	NM_198492	chr19	7699842	7703057	-	.	.	.	.	.	.	.	.	.	.	.	.	.	.	.	.	.	.	.	.
891	2,5	MAP2K7	NM_145185	chr19	7874764	7885363	+	.	.	.	.	.	.	.	.	.	.	.	.	.	.	.	.	.	.	.	.
2122	1,5	ELAVL1	NM_001419	chr19	7929456	7976529	-	.	.	.	.	.	.	.	.	.	.	.	.	.	.	.	.	.	.	.	.
4530	1	NDUFA7	NM_005001	chr19	8282183	8292280	-	.	.	.	.	.	.	.	.	.	.	.	.	.	.	.	.	.	.	.	.
6009	0,5	ANGPTL4	NM_001039667	chr19	8335010	8345257	+	.	.	.	.	.	.	.	.	.	.	.	.	.	.	.	.	.	.	.	.
5019	1	RAB11B	NM_004218	chr19	8361204	8375317	+	.	.	.	.	.	.	.	.	.	.	.	.	.	.	.	.	.	.	.	.
6441	0,5	HNRNPM	NM_005968	chr19	8415802	8460002	+	CL799551	chr19	8419124	-	.	.	.	.	.	.	.	.	.	.	.	.	.	.	.	.
6441	0,5	HNRNPM	NM_005968	chr19	8415802	8460002	+	AY517154.1	chr19	8430545	+	.	.	.	.	.	.	.	.	.	.	.	.	.	.	.	.
6441	0,5	HNRNPM	NM_005968	chr19	8415802	8460002	+	AY517156.1	chr19	8423119	-	.	.	.	.	.	.	.	.	.	.	.	.	.	.	.	.
6640	0,5	MYO1F	NM_012335	chr19	8491673	8548331	-	.	.	.	.	.	.	.	.	.	.	.	.	.	.	.	.	.	.	.	.
2685	1,25	MUC16	NM_024690	chr19	8820519	8953018	-	.	.	.	.	.	.	.	.	.	.	.	.	.	.	.	.	.	.	.	.
5824	1	ZNF560	NM_152476	chr19	9438030	9470279	-	.	.	.	.	.	.	.	.	.	.	.	.	.	.	.	.	.	.	.	.
4813	1	PIN1	NR_038422	chr19	9806882	9821365	+	.	.	.	.	.	.	.	.	.	.	.	.	.	.	.	.	.	.	.	.
3418	1	COL5A3	NM_015719	chr19	9931236	9982147	-	.	.	.	.	.	.	.	.	.	.	.	.	.	.	.	.	.	.	.	.
5088	1	RDH8	NM_015725	chr19	9984924	9993954	+	.	.	.	.	.	.	.	.	.	.	.	.	.	.	.	.	.	.	.	.
3230	1	C3P1	NR_027300	chr19	10013031	10045813	+	.	.	.	.	.	.	.	.	.	.	.	.	.	.	.	.	.	.	.	.
2037	1,5	C19orf66	NM_018381	chr19	10057805	10064928	+	.	.	.	.	.	.	.	.	.	.	.	.	.	.	.	.	.	.	.	.
4886	1	PPAN	NM_020230	chr19	10077898	10082975	+	.	.	.	.	.	.	.	.	.	.	.	.	.	.	.	.	.	.	.	.
3668	1	EIF3G	NM_003755	chr19	10086689	10091599	-	.	.	.	.	.	.	.	.	.	.	.	.	.	.	.	.	.	.	.	.
41	7,75	DNMT1	NM_001379	chr19	10105021	10166755	-	AY516997.1	chr19	10151281	-	.	.	.	.	.	.	.	.	.	.	.	.	.	.	.	.
41	7,75	DNMT1	NM_001379	chr19	10105021	10166755	-	BH609806	chr19	10159085	+	.	.	.	.	.	.	.	.	.	.	.	.	.	.	.	.
41	7,75	DNMT1	NM_001379	chr19	10105021	10166755	-	BH609805	chr19	10145130	-	.	.	.	.	.	.	.	.	.	.	.	.	.	.	.	.
41	7,75	DNMT1	NM_001379	chr19	10105021	10166755	-	BH609746	chr19	10159085	+	.	.	.	.	.	.	.	.	.	.	.	.	.	.	.	.
4438	1	MRPL4	NM_146387	chr19	10223639	10231721	+	.	.	.	.	.	.	.	.	.	.	.	.	.	.	.	.	.	.	.	.
330	3,75	ICAM1	NM_000201	chr19	10242516	10258291	+	.	.	.	.	.	.	.	.	.	.	.	.	.	.	.	.	.	.	.	.
1460	2	ICAM5	NM_003259	chr19	10261654	10268454	+	.	.	.	.	.	.	.	.	.	.	.	.	.	.	.	.	.	.	.	.
3768	1	FDX1L	NM_001031734	chr19	10281890	10287691	-	.	.	.	.	.	.	.	.	.	.	.	.	.	.	.	.	.	.	.	.
383	3,5	ICAM3	NM_002162	chr19	10305451	10311345	-	.	.	.	.	.	.	.	.	.	.	.	.	.	.	.	.	.	.	.	.

Table S2

tumor associated genes								HIV				MLV				MMTV				MMTV(SIN)				MMTV(SIN)arrest			
rank	score	gene symbol	RefSEq	chrom	txStart	txEnd	strand	integrant	chr	position	strand	integrant	chr	position	strand	integrant	chr	position	strand	integrant	chr	position	strand	integrant	chr	position	strand
512	3,25	TYK2	NM_003331	chr19	10322203	10352248	-	BH609807	chr19	10344802	-	.	.	.	.	.	.	.	.	.	.	.	.	.	.	.	.
1890	1,75	CDC37	NM_007065	chr19	10362808	10375271	-	.	.	.	.	.	.	.	.	.	.	.	.	.	.	.	.	.	.	.	.
1604	2	PDE4A	NM_001243121	chr19	10388448	10441307	+	.	.	.	.	.	.	.	.	.	.	.	.	.	.	.	.	.	.	.	.
391	3,5	KEAP1	NM_012289	chr19	10457795	10474481	-	.	.	.	.	.	.	.	.	.	.	.	.	.	.	.	.	.	.	.	.
3059	1	ATG4D	NM_032885	chr19	10515646	10525094	+	.	.	.	.	.	.	.	.	.	.	.	.	.	.	.	.	.	.	.	.
4246	1	KRI1	NM_023008	chr19	10524760	10537702	-	BH609808	chr19	10528382	-	.	.	.	.	.	.	.	.	.	.	.	.	.	.	.	.
152	4,75	CDKN2D	NM_079421	chr19	10538137	10540655	-	.	.	.	.	.	.	.	.	.	.	.	.	.	.	.	.	.	.	.	.
5279	1	SLC44A2	NM_020428	chr19	10597170	10616235	+	.	.	.	.	.	.	.	.	.	.	.	.	.	.	.	.	.	.	.	.
2203	1,5	ILF3	NM_004516	chr19	10625936	10657443	+	CL529575	chr19	10629649	+	.	.	.	.	.	.	.	.	.	.	.	.	.	.	.	.
2356	1,5	QTRT1	NM_031209	chr19	10673111	10685043	+	.	.	.	.	.	.	.	.	.	.	.	.	.	.	.	.	.	.	.	.
1036	2,25	DNM2	NM_001005361	chr19	10689728	10803586	+	.	.	.	.	.	.	.	.	.	.	.	.	.	.	.	.	.	.	.	.
2440	1,5	TMED1	NM_006858	chr19	10804113	10807983	-	.	.	.	.	.	.	.	.	.	.	.	.	.	.	.	.	.	.	.	.
130	5	CARM1	NM_199141	chr19	10843252	10894448	+	.	.	.	.	.	.	.	.	.	.	.	.	.	.	.	.	.	.	.	.
5723	1	YIPF2	NM_024029	chr19	10894443	10900357	-	.	.	.	.	.	.	.	.	.	.	.	.	.	.	.	.	.	.	.	.
3211	1	C19orf52	NM_138358	chr19	10900423	10901916	+	.	.	.	.	.	.	.	.	.	.	.	.	.	.	.	.	.	.	.	.
38	8	SMARCA4	NM_001128848	chr19	10955827	11033958	+	.	.	.	.	.	.	.	.	.	.	.	.	.	.	.	.	.	.	.	.
1499	2	LDLR	NM_001195803	chr19	11061037	11105505	+	.	.	.	.	.	.	.	.	.	.	.	.	.	.	.	.	.	.	.	.
5325	1	SPC24	NM_182513	chr19	11118830	11127484	-	.	.	.	.	.	.	.	.	.	.	.	.	.	.	.	.	.	.	.	.
4194	1	KANK2	NM_015493	chr19	11135942	11166266	-	.	.	.	.	.	.	.	.	.	.	.	.	.	.	.	.	.	.	.	.
2108	1,5	DOCK6	NM_020812	chr19	11170968	11234168	-	BH609809	chr19	11221962	-	.	.	.	.	.	.	.	.	.	.	.	.	.	.	.	.
940	2,5	RAB3D	NM_004283	chr19	11293721	11311344	-	.	.	.	.	.	.	.	.	.	.	.	.	.	.	.	.	.	.	.	.
5494	1	TMEM205	NM_033408	chr19	11314451	11317981	-	.	.	.	.	.	.	.	.	.	.	.	.	.	.	.	.	.	.	.	.
4299	1	LPPR2	NM_022737	chr19	11327061	11337374	+	.	.	.	.	.	.	.	.	.	.	.	.	.	.	.	.	.	.	.	.
2126	1,5	EPOR	NM_000121	chr19	11348880	11356018	-	.	.	.	.	.	.	.	.	.	.	.	.	.	.	.	.	.	.	.	.
5113	1	RGL3	NM_001161616	chr19	11365731	11391018	-	.	.	.	.	.	.	.	.	.	.	.	.	.	.	.	.	.	.	.	.
1686	2	PRKCSH	NM_002743	chr19	11407268	11422782	+	.	.	.	.	.	.	.	.	.	.	.	.	.	.	.	.	.	.	.	.
2579	1,25	ELAVL3	NM_032281	chr19	11423142	11452803	-	.	.	.	.	.	.	.	.	.	.	.	.	.	.	.	.	.	.	.	.
5841	1	ZNF653	NM_138783	chr19	11455241	11477738	-	.	.	.	.	.	.	.	.	.	.	.	.	.	.	.	.	.	.	.	.
1202	2	ACP5	NM_001611	chr19	11546474	11549553	-	.	.	.	.	.	.	.	.	.	.	.	.	.	.	.	.	.	.	.	.
7128	0,5	ZNF20	NM_001203250	chr19	12103167	12112222	-	.	.	.	.	.	.	.	.	.	.	.	.	.	.	.	.	.	.	.	.
5803	1	ZNF442	NM_030824	chr19	12321184	12337475	-	.	.	.	.	.	.	.	.	.	.	.	.	.	.	.	.	.	.	.	.
2238	1,5	MAN2B1	NM_001173498	chr19	12618321	12638591	-	.	.	.	.	.	.	.	.	.	.	.	.	.	.	.	.	.	.	.	.
1087	2,25	JUNB	NM_002229	chr19	12763309	12765125	+	.	.	.	.	.	.	.	.	.	.	.	.	.	.	.	.	.	.	.	.
5131	1	RNASEH2A	NM_006397	chr19	12778427	12785462	+	AY517251.1	chr19	12785306	+	.	.	.	.	.	.	.	.	.	.	.	.	.	.	.	.
1525	2	MAST1	NM_014975	chr19	12810258	12846766	+	.	.	.	.	.	.	.	.	.	.	.	.	.	.	.	.	.	.	.	.
3867	1	GCDH	NM_000159	chr19	12862973	12871783	+	.	.	.	.	.	.	.	.	.	.	.	.	.	.	.	.	.	.	.	.
3737	1	FARSA	NM_004461	chr19	12894283	12905558	-	.	.	.	.	.	.	.	.	.	.	.	.	.	.	.	.	.	.	.	.
7222	0,25	CALR	NM_004343	chr19	12910413	12916304	+	.	.	.	.	.	.	.	.	.	.	.	.	.	.	.	.	.	.	.	.
5050	1	RAD23A	NR_072976	chr19	12917627	12925457	+	.	.	.	.	.	.	.	.	.	.	.	.	.	.	.	.	.	.	.	.
1512	2	LYL1	NM_005583	chr19	13070841	13074974	-	.	.	.	.	.	.	.	.	.	.	.	.	.	.	.	.	.	.	.	.
3258	1	CACNA1A	NM_001174080	chr19	13178256	13478274	-	.	.	.	.	.	.	.	.	.	.	.	.	.	.	.	.	901_124_1	chr19	13352997	+
6137	0,5	CC2D1A	NM_017721	chr19	13877955	13902693	+	.	.	.	.	.	.	.	.	.	.	.	.	.	.	.	.	.	.	.	.
6811	0,5	RFX1	NM_002918	chr19	13933341	13978134	-	.	.	.	.	.	.	.	.	.	.	.	.	.	.	.	.	.	.	.	.
2342	1,5	PRKACA	NM_207518	chr19	14063506	14085992	-	.	.	.	.	.	.	.	.	.	.	.	.	.	.	.	.	.	.	.	.
3054	1	ASF1B	NM_018154	chr19	14091320	14108440	-	.	.	.	.	.	.	.	.	.	.	.	.	.	.	.	.	.	.	.	.
6156	0,5	CD97	NM_001025160	chr19	14352955	14380537	+	.	.	.	.	.	.	.	.	.	.	.	.	.	.	.	.	.	.	.	.
2713	1,25	PKN1	NM_213560	chr19	14412085	14443679	+	.	.	.	.	.	.	.	.	.	.	.	.	.	.	.	.	.	.	.	.
7328	0,25	GIPC1	NM_202469	chr19	14449570	14467961	-	CL800283	chr19	14461909	-	.	.	.	.	.	.	.	.	.	.	.	.	.	.	.	.
6273	0,5	DNAJB1	NM_006145	chr19	14486581	14490201	-	.	.	.	.	.	.	.	.	.	.	.	.	.	.	.	.	.	.	.	.
4542	1	NDUFB7	NM_004146	chr19	14537889	14543889	-	.	.	.	.	.	.	.	.	.	.	.	.	.	.	.	.	.	.	.	.
5256	1	SLC1A6	NM_005071	chr19	14921990	14944730	-	.	.	.	.	.	.	.	.	.	.	.	.	.	.	.	.	.	.	.	.

Table S2

tumor associated genes								HIV				MLV				MMTV				MMTV(SIN)				MMTV(SIN)arrest			
rank	score	gene symbol	RefSEq	chrom	txStart	txEnd	strand	integrant	chr	position	strand	integrant	chr	position	strand	integrant	chr	position	strand	integrant	chr	position	strand	integrant	chr	position	strand
4143	1	ILVBL	NM_006844	chr19	15086784	15097577	-	.	.	.	.	.	.	.	.	.	.	.	.	.	.	.	.	.	.	.	.
160	4,75	NOTCH3	NM_000435	chr19	15131443	15172792	-	.	.	.	.	.	.	.	.	.	.	.	.	.	.	.	.	.	.	.	.
796	2,5	BRD4	NM_014299	chr19	15218846	15252262	-	.	.	.	.	.	.	.	.	.	.	.	.	.	.	.	.	.	.	.	.
6004	0,5	AKAP8L	NM_014371	chr19	15351858	15390833	-	CL800059	chr19	15364371	+	.	.	.	.	.	.	.	.	.	.	.	.	.	.	.	.
7099	0,5	WIZ	NM_021241	chr19	15393317	15421762	-	CL800441	chr19	15409883	+	.	.	.	.	.	.	.	.	.	.	.	.	3222_318_1	chr19	15393988	+
4773	1	PGLYRP2	NM_052890	chr19	15440456	15451315	-	.	.	.	.	.	.	.	.	.	.	.	.	.	.	.	.	.	.	.	.
975	2,5	TPM4	NM_001145160	chr19	16039316	16074813	+	.	.	.	.	.	.	.	.	.	.	.	.	.	.	.	.	.	.	.	.
4233	1	KLF2	NM_016270	chr19	16296650	16299339	+	.	.	.	.	.	.	.	.	.	.	.	.	.	.	.	.	.	.	.	.
6318	0,5	EPS15L1	NM_001258375	chr19	16333388	16443823	-	.	.	.	.	.	.	.	.	.	.	.	.	.	.	.	.	.	.	.	.
6174	0,5	CHERP	NM_006387	chr19	16489699	16514263	-	.	.	.	.	.	.	.	.	.	.	.	.	.	.	.	.	.	.	.	.
5244	1	SIN3B	NM_015260	chr19	16801208	16852164	+	.	.	.	.	.	.	.	.	.	.	.	.	.	.	.	.	.	.	.	.
3445	1	CPAMD8	NM_015692	chr19	16864761	16998625	-	.	.	.	.	.	.	.	.	.	.	.	.	.	.	.	.	.	.	.	.
6641	0,5	MYO9B	NM_001130065	chr19	17047590	17185104	+	CL800237	chr19	17136558	+	.	.	.	.	.	.	.	.	.	.	.	.	.	.	.	.
2286	1,5	NR2F6	NM_005234	chr19	17203693	17217151	-	CL529193	chr19	17212580	+	.	.	.	.	.	.	.	.	.	.	.	.	.	.	.	.
4847	1	PLVAP	NM_031310	chr19	17323263	17349137	-	.	.	.	.	.	.	.	.	.	.	.	.	.	.	.	.	.	.	.	.
6070	0,5	BST2	NM_004335	chr19	17374754	17377384	-	.	.	.	.	.	.	.	.	.	.	.	.	.	.	.	.	.	.	.	.
4772	1	PGLS	NM_012088	chr19	17483431	17493097	+	.	.	.	.	.	.	.	.	.	.	.	.	.	.	.	.	.	.	.	.
6387	0,5	GLT25D1	NM_024656	chr19	17527510	17554965	+	.	.	.	.	.	.	.	.	.	.	.	.	.	.	.	.	.	.	.	.
3113	1	B3GNT3	NM_014256	chr19	17766918	17785385	+	.	.	.	.	.	.	.	.	.	.	.	.	.	.	.	.	.	.	.	.
4153	1	INSL3	NM_001265587	chr19	17788321	17793383	-	.	.	.	.	.	.	.	.	.	.	.	.	.	.	.	.	.	.	.	.
334	3,75	JAK3	NM_000215	chr19	17796592	17819841	-	.	.	.	.	.	.	.	.	.	.	.	.	.	.	.	.	.	.	.	.
6505	0,5	KCNN1	NM_002248	chr19	17923110	17970930	+	.	.	.	.	.	.	.	.	.	.	.	.	.	.	.	.	.	.	.	.
4130	1	IL12RB1	NM_153701	chr19	18042371	18058697	-	.	.	.	.	.	.	.	.	.	.	.	.	.	.	.	.	.	.	.	.
2245	1,5	MAST3	NM_015016	chr19	18069602	18123499	+	.	.	.	.	.	.	.	.	.	.	.	.	.	.	.	.	.	.	.	.
224	4,25	PIK3R2	NM_005027	chr19	18125015	18142343	+	.	.	.	.	.	.	.	.	.	.	.	.	.	.	.	.	.	.	.	.
4116	1	IFI30	NM_006332	chr19	18145578	18149927	+	.	.	.	.	.	.	.	.	.	.	.	.	.	.	.	.	.	.	.	.
4435	1	MPV17L2	NM_032683	chr19	18165039	18168550	+	.	.	.	.	.	.	.	.	.	.	.	.	.	.	.	.	.	.	.	.
2360	1,5	RAB3A	NM_002866	chr19	18168610	18175874	-	.	.	.	.	.	.	.	.	.	.	.	.	.	.	.	.	.	.	.	.
4734	1	PDE4C	NM_000923	chr19	18179770	18220010	-	.	.	.	.	AY516849	chr19	18203938	-	.	.	.	.	.	.	.	.	.	.	.	.
734	2,75	JUND	NM_005354	chr19	18251562	18253432	-	.	.	.	.	.	.	.	.	.	.	.	.	.	.	.	.	.	.	.	.
4776	1	PGPEP1	NM_017712	chr19	18312407	18341763	+	.	.	.	.	.	.	.	.	.	.	.	.	.	.	.	.	.	.	.	.
1912	1,75	GDF15	NM_004864	chr19	18357967	18360986	+	.	.	.	.	.	.	.	.	.	.	.	.	.	.	.	.	.	.	.	.
5356	1	SSBP4	NM_001009998	chr19	18391145	18406372	+	.	.	.	.	.	.	.	.	.	.	.	.	.	.	.	.	.	.	.	.
4171	1	ISYNA1	NM_001253389	chr19	18406197	18409119	-	.	.	.	.	.	.	.	.	.	.	.	.	.	.	.	.	.	.	.	.
462	3,25	ELL	NM_006532	chr19	18414472	18493937	-	.	.	.	.	.	.	.	.	.	.	.	.	.	.	.	.	.	.	.	.
1406	2	FKBP8	NM_012181	chr19	18503567	18515383	-	.	.	.	.	.	.	.	.	.	.	.	.	.	.	.	.	.	.	.	.
5596	1	UBA52	NM_003333	chr19	18543613	18549270	+	.	.	.	.	.	.	.	.	.	.	.	.	.	.	.	.	.	.	.	.
2523	1,25	C19orf60	NM_001100419	chr19	18560494	18564147	+	.	.	.	.	.	.	.	.	.	.	.	.	.	.	.	.	.	.	.	.
3461	1	CRLF1	NM_004750	chr19	18565034	18578660	-	.	.	.	.	.	.	.	.	.	.	.	.	.	.	.	.	.	.	.	.
5499	1	TMEM59L	NM_012109	chr19	18584681	18592849	+	.	.	.	.	.	.	.	.	.	.	.	.	.	.	.	.	.	.	.	.
4236	1	KLHL26	NM_018316	chr19	18608837	18642302	+	.	.	.	.	.	.	.	.	.	.	.	.	.	.	.	.	.	.	.	.
552	3	CRTC1	NM_001098482	chr19	18655424	18754143	+	.	.	.	.	.	.	.	.	.	.	.	.	.	.	.	.	.	.	.	.
6206	0,5	COMP	NM_000095	chr19	18754582	18763114	-	.	.	.	.	.	.	.	.	.	.	.	.	.	.	.	.	.	.	.	.
2453	1,5	UPF1	NM_002911	chr19	18803743	18840039	+	.	.	.	.	.	.	.	.	.	.	.	.	.	.	.	.	.	.	.	.
3872	1	GDF1	NM_001492	chr19	18840360	18867953	-	.	.	.	.	.	.	.	.	.	.	.	.	.	.	.	.	.	.	.	.
3422	1	COPE	NM_199442	chr19	18871322	18891199	-	.	.	.	.	.	.	.	.	.	.	.	.	.	.	.	.	.	.	.	.
3550	1	DDX49	NR_033677	chr19	18891483	18900442	+	.	.	.	.	.	.	.	.	.	.	.	.	.	.	.	.	.	.	.	.
4067	1	HOMER3	NM_001145722	chr19	18901009	18912113	-	.	.	.	.	.	.	.	.	.	.	.	.	.	.	.	.	.	.	.	.
3041	1	ARMC6	NM_001199196	chr19	19005386	19029987	+	.	.	.	.	.	.	.	.	.	.	.	.	.	.	.	.	.	.	.	.
5265	1	SLC25A42	NM_178526	chr19	19035802	19084841	+	.	.	.	.	.	.	.	.	.	.	.	.	.	.	.	.	.	.	.	.
2442	1,5	TMEM161A	NM_001256766	chr19	19091424	19110310	-	.	.	.	.	.	.	.	.	.	.	.	.	.	.	.	.	.	.	.	.

Table S2

tumor associated genes								HIV				MLV				MMTV				MMTV(SIN)				MMTV(SIN)arrest			
rank	score	gene symbol	RefSEq	chrom	txStart	txEnd	strand	integrant	chr	position	strand	integrant	chr	position	strand	integrant	chr	position	strand	integrant	chr	position	strand	integrant	chr	position	strand
2374	1,5	RFXANK	NM_134440	chr19	19164007	19173678	+	.	.	.	.	.	.	.	.	.	.	.	.	.	.	.	.	.	.	.	.
4612	1	NR2C2AP	NM_176880	chr19	19173223	19175238	-	.	.	.	.	.	.	.	.	.	.	.	.	.	.	.	.	.	.	.	.
4502	1	NCAN	NM_004386	chr19	19183781	19224061	+	.	.	.	.	.	.	.	.	.	.	.	.	.	.	.	.	.	.	.	.
4013	1	HAPLN4	NM_023002	chr19	19227450	19234596	-	.	.	.	.	.	.	.	.	.	.	.	.	.	.	.	.	.	.	.	.
5479	1	TM6SF2	NM_001001524	chr19	19236173	19245074	-	.	.	.	.	.	.	.	.	.	.	.	.	.	.	.	.	.	.	.	.
2152	1,5	GATAD2A	NM_017660	chr19	19357641	19480741	+	.	.	.	.	.	.	.	.	.	.	.	.	.	.	.	.	.	.	.	.
5578	1	TSSK6	NM_032037	chr19	19486027	19487469	-	.	.	.	.	.	.	.	.	.	.	.	.	.	.	.	.	.	.	.	.
1118	2,25	NDUFA13	NM_015965	chr19	19488018	19500013	+	.	.	.	.	.	.	.	.	.	.	.	.	.	.	.	.	.	.	.	.
3369	1	CILP2	NM_153221	chr19	19510073	19518468	+	.	.	.	.	.	.	.	.	.	.	.	.	.	.	.	.	.	.	.	.
4297	1	LPAR2	NM_004720	chr19	19595465	19600039	-	.	.	.	.	.	.	.	.	.	.	.	.	.	.	.	.	.	.	.	.
1264	2	ATP13A1	NM_020410	chr19	19617009	19635503	-	.	.	.	.	.	.	.	.	.	.	.	.	.	.	.	.	.	.	.	.
5756	1	ZNF101	NM_033204	chr19	19640662	19652138	+	.	.	.	.	.	.	.	.	.	.	.	.	.	.	.	.	.	.	.	.
2838	1,25	ZNF14	NM_021030	chr19	19682280	19704921	-	.	.	.	.	.	.	.	.	.	.	.	.	.	.	.	.	.	.	.	.
5812	1	ZNF506	NM_001099269	chr19	19764519	19793560	-	.	.	.	.	.	.	.	.	.	.	.	.	.	.	.	.	.	.	.	.
5778	1	ZNF253	NM_021047	chr19	19837713	19865293	+	.	.	.	.	.	.	.	.	.	.	.	.	.	.	.	.	.	.	.	.
7632	0,25	ZNF737	NM_001159293	chr19	20512637	20540466	-	.	.	.	.	.	.	.	.	.	.	.	.	.	.	.	.	.	.	.	.
7630	0,25	ZNF626	NM_001076675	chr19	20594584	20636242	-	.	.	.	.	.	.	.	.	.	.	.	.	.	.	.	.	.	.	.	.
7144	0,5	ZNF85	NM_003429	chr19	20897898	20925343	+	CL529644	chr19	20916603	-	.	.	.	.	.	.	.	.	.	.	.	.	.	.	.	.
7629	0,25	ZNF430	NM_001172671	chr19	20995265	21034692	+	AY517034.1	chr19	21009080	-	.	.	.	.	.	.	.	.	.	.	.	.	.	.	.	.
7631	0,25	ZNF714	NM_182515	chr19	21056792	21099723	+	.	.	.	.	.	.	.	.	.	.	.	.	.	.	.	.	.	.	.	.
5802	1	ZNF431	NM_133473	chr19	21116679	21160645	+	.	.	.	.	.	.	.	.	.	.	.	.	.	.	.	.	.	.	.	.
5848	1	ZNF708	NM_021269	chr19	21265802	21304052	-	.	.	.	.	.	.	.	.	.	.	.	.	.	.	.	.	.	.	.	.
7135	0,5	ZNF493	NM_175910	chr19	21371760	21402136	+	.	.	.	.	.	.	.	.	.	.	.	.	.	.	.	.	.	.	.	.
7628	0,25	ZNF429	NM_001001415	chr19	21480276	21512919	+	.	.	.	.	.	.	.	.	.	.	.	.	.	.	.	.	.	.	.	.
1845	2	UQCRFS1	NM_006003	chr19	34390006	34395976	-	.	.	.	.	.	.	.	.	.	.	.	.	.	.	.	.	.	.	.	.
173	4,5	CCNE1	NM_001238	chr19	34994740	35007055	+	.	.	.	.	.	.	.	.	.	.	.	.	.	.	.	.	.	.	.	.
5120	1	RHPN2	NM_033103	chr19	38161337	38247664	-	.	.	.	.	.	.	.	.	.	.	.	.	.	.	.	.	.	.	.	.
6925	0,5	SLC7A10	NM_019849	chr19	38391409	38408596	-	.	.	.	.	.	.	.	.	.	.	.	.	.	.	.	.	.	.	.	.
205	4,25	CEBPA	NM_004364	chr19	38482679	38485270	-	.	.	.	.	.	.	.	.	.	.	.	.	.	.	.	.	.	.	.	.
5944	0,75	PEPD	NM_001166056	chr19	38569694	38704639	-	.	.	.	.	.	.	.	.	.	.	.	.	.	.	.	.	.	.	.	.
6518	0,5	KIAA0355	NM_014686	chr19	39437295	39538311	+	.	.	.	.	.	.	.	.	.	.	.	.	.	.	.	.	.	.	.	.
1060	2,25	GPI	NM_001184722	chr19	39547484	39585158	+	.	.	.	.	.	.	.	.	.	.	.	.	.	.	.	.	.	.	.	.
6855	0,5	SCN1B	NM_001037	chr19	40213431	40223193	+	.	.	.	.	.	.	.	.	.	.	.	.	.	.	.	.	.	.	.	.
2192	1,5	HPN	NM_002151	chr19	40223249	40249317	+	.	.	.	.	.	.	.	.	.	.	.	.	.	.	.	.	.	.	.	.
6556	0,5	LGI4	NM_139284	chr19	40307256	40318018	-	.	.	.	.	.	.	.	.	.	.	.	.	.	.	.	.	.	.	.	.
2144	1,5	FXYD1	NM_005031	chr19	40322231	40325794	+	.	.	.	.	.	.	.	.	.	.	.	.	.	.	.	.	.	.	.	.
5911	0,75	FXYD7	NM_022006	chr19	40325993	40337045	+	.	.	.	.	.	.	.	.	.	.	.	.	.	.	.	.	.	.	.	.
5910	0,75	FXYD5	NM_144779	chr19	40337511	40352628	+	.	.	.	.	.	.	.	.	.	.	.	.	.	.	.	.	.	.	.	.
7081	0,5	USF2	NM_207291	chr19	40451735	40462558	+	.	.	.	.	.	.	.	.	.	.	.	.	.	.	.	.	.	.	.	.
6421	0,5	HAMP	NM_021175	chr19	40465249	40467885	+	.	.	.	.	.	.	.	.	.	.	.	.	.	.	.	.	.	.	.	.
1933	1,75	MAG	NM_080600	chr19	40474828	40496549	+	.	.	.	.	.	.	.	.	.	.	.	.	.	.	.	.	.	.	.	.
6148	0,5	CD22	NM_001185101	chr19	40511911	40530104	+	.	.	.	.	.	.	.	.	.	.	.	.	.	.	.	.	.	.	.	.
3856	1	GAPDHS	NM_014364	chr19	40716153	40728061	+	.	.	.	.	.	.	.	.	.	.	.	.	.	.	.	.	.	.	.	.
2441	1,5	TMEM147	NM_032635	chr19	40728341	40730269	+	.	.	.	.	.	.	.	.	.	.	.	.	.	.	.	.	.	.	.	.
3069	1	ATP4A	NM_000704	chr19	40732934	40746400	-	.	.	.	.	.	.	.	.	.	.	.	.	.	.	.	.	.	.	.	.
4019	1	HAUS5	NM_015302	chr19	40795485	40808091	+	.	.	.	.	.	.	.	.	.	.	.	.	.	.	.	.	.	.	.	.
2368	1,5	RBM42	NM_024321	chr19	40811819	40820427	+	.	.	.	.	.	.	.	.	.	.	.	.	.	.	.	.	.	.	.	.
1323	2	COX6B1	NM_001863	chr19	40830964	40841526	+	.	.	.	.	.	.	.	.	.	.	.	.	.	.	.	.	.	.	.	.
1535	2	MLL4	NM_014727	chr19	40900760	40921619	+	.	.	.	.	.	.	.	.	.	.	.	.	.	.	.	.	.	.	.	.
1695	2	PSENEN	NM_172341	chr19	40928333	40929743	+	.	.	.	.	.	.	.	.	.	.	.	.	.	.	.	.	.	.	.	.
4285	1	LIN37	NM_019104	chr19	40931101	40937260	+	.	.	.	.	.	.	.	.	.	.	.	.	.	.	.	.	.	.	.	.

Table S2

tumor associated genes								HIV				MLV				MMTV				MMTV(SIN)				MMTV(SIN)arrest			
rank	score	gene symbol	RefSEq	chrom	txStart	txEnd	strand	integrant	chr	position	strand	integrant	chr	position	strand	integrant	chr	position	strand	integrant	chr	position	strand	integrant	chr	position	strand
4096	1	HSPB6	NM_144617	chr19	40937306	40939770	-	.	.	.	.	.	.	.	.	.	.	.	.	.	.	.	.	.	.	.	.
3212	1	C19orf55	NM_001039887	chr19	40940883	40951917	+	.	.	.	.	.	.	.	.	.	.	.	.	.	.	.	.	.	.	.	.
5595	1	TYROBP	NM_001173515	chr19	41087142	41091051	-	CL800401	chr19	41089579	+	.	.	.	.	.	.	.	.	.	.	.	.	.	.	.	.
6571	0,5	LRFN3	NM_024509	chr19	41119861	41127937	+	.	.	.	.	.	.	.	.	.	.	.	.	.	.	.	.	.	.	.	.
1654	2	POLR2I	NM_006233	chr19	41296450	41298046	-	.	.	.	.	.	.	.	.	.	.	.	.	.	.	.	.	.	.	.	.
2089	1,5	COX7A1	NM_001864	chr19	41333663	41335611	-	.	.	.	.	.	.	.	.	.	.	.	.	.	.	.	.	.	.	.	.
7125	0,5	ZNF146	NM_001099639	chr19	41397343	41421515	+	CL800164	chr19	41404231	+	.	.	.	.	.	.	.	.	.	.	.	.	.	.	.	.
7347	0,25	HKR1	NM_181786	chr19	42517419	42547197	+	.	.	.	.	.	.	.	.	.	.	.	.	.	.	.	.	.	.	.	.
5825	1	ZNF569	NM_152484	chr19	42593899	42650179	-	.	.	.	.	.	.	.	.	.	.	.	.	.	.	.	.	.	.	.	.
7137	0,5	ZNF540	NM_152606	chr19	42734112	42796919	+	.	.	.	.	.	.	.	.	.	.	.	.	.	.	.	.	.	.	.	.
6284	0,5	DPF1	NM_001135155	chr19	43393488	43406730	-	.	.	.	.	.	.	.	.	.	.	.	.	.	.	.	.	.	.	.	.
2335	1,5	PPP1R14A	NM_033256	chr19	43433716	43439071	-	.	.	.	.	.	.	.	.	.	.	.	.	.	.	.	.	.	.	.	.
1770	2	SPINT2	NM_021102	chr19	43446937	43475094	+	AY517183.1	chr19	43466962	-	.	.	.	.	.	.	.	.	.	.	.	.	.	.	.	.
3206	1	C19orf33	NM_033520	chr19	43486643	43487486	+	.	.	.	.	.	.	.	.	.	.	.	.	.	.	.	.	.	.	.	.
6964	0,5	SPRED3	NR_073032	chr19	43572771	43582363	+	.	.	.	.	.	.	.	.	.	.	.	.	.	.	.	.	.	.	.	.
1718	2	RASGRP4	NM_001146205	chr19	43591537	43608785	-	.	.	.	.	.	.	.	.	.	.	.	.	.	.	.	.	.	.	.	.
6841	0,5	RYR1	NM_001042723	chr19	43616179	43770044	+	.	.	.	.	.	.	.	.	.	.	.	.	.	.	.	.	.	.	.	.
4351	1	MAP4K1	NM_001042600	chr19	43770120	43800483	-	.	.	.	.	.	.	.	.	.	.	.	.	.	.	.	.	.	.	.	.
5874	0,75	ACTN4	NM_004924	chr19	43830106	43913011	+	BH609820	chr19	43841451	+	.	.	.	.	.	.	.	.	.	.	.	.	.	.	.	.
3642	1	ECH1	NM_001398	chr19	43997901	44014337	-	.	.	.	.	.	.	.	.	.	.	.	.	.	.	.	.	.	.	.	.
7354	0,25	HNRNPL	NM_001533	chr19	44018867	44032457	-	.	.	.	.	.	.	.	.	.	.	.	.	.	.	.	.	.	.	.	.
1759	2	SIRT2	NR_034146	chr19	44061034	44076635	-	.	.	.	.	.	.	.	.	.	.	.	.	.	.	.	.	.	.	.	.
2387	1,5	SARS2	NM_001145901	chr19	44097743	44113376	-	.	.	.	.	.	.	.	.	.	.	.	.	.	.	.	.	.	.	.	.
6348	0,5	FBXO17	NM_148169	chr19	44123881	44135167	-	.	.	.	.	.	.	.	.	.	.	.	.	.	.	.	.	.	.	.	.
4690	1	PAK4	NM_005884	chr19	44308259	44361886	+	.	.	.	.	.	.	.	.	.	.	.	.	.	.	.	.	.	.	.	.
6754	0,5	PLEKHG2	NM_022835	chr19	44595589	44610895	+	.	.	.	.	.	.	.	.	.	.	.	.	.	.	.	.	.	.	.	.
1359	2	DLL3	NM_016941	chr19	44681396	44690961	+	.	.	.	.	.	.	.	.	.	.	.	.	.	.	.	.	.	.	.	.
3634	1	DYRK1B	NM_006483	chr19	45007829	45016681	-	.	.	.	.	.	.	.	.	.	.	.	.	.	.	.	.	.	.	.	.
1400	2	FCGBP	NM_003890	chr19	45045802	45132373	-	.	.	.	.	.	.	.	.	.	.	.	.	.	.	.	.	.	.	.	.
2239	1,5	MAP3K10	NM_002446	chr19	45389490	45413322	+	.	.	.	.	.	.	.	.	.	.	.	.	.	.	.	.	.	.	.	.
47	7,25	AKT2	NM_001626	chr19	45428063	45483142	-	.	.	.	.	.	.	.	.	.	.	.	.	.	.	.	.	.	.	.	.
4047	1	HIPK4	NM_144685	chr19	45577017	45587934	-	.	.	.	.	.	.	.	.	.	.	.	.	.	.	.	.	.	.	.	.
2394	1,5	SERTAD1	NM_013376	chr19	45620248	45623772	-	.	.	.	.	.	.	.	.	.	.	.	.	.	.	.	.	.	.	.	.
2026	1,5	BLVRB	NM_000713	chr19	45645530	45663565	-	.	.	.	.	.	.	.	.	.	.	.	.	.	.	.	.	.	.	.	.
2907	1	ADCK4	NM_024876	chr19	45889273	45914630	-	.	.	.	.	.	.	.	.	.	.	.	.	.	.	.	.	.	.	.	.
4182	1	ITPKC	NM_025194	chr19	45914847	45938605	+	.	.	.	.	.	.	.	.	.	.	.	.	.	.	.	.	.	.	.	.
6614	0,5	MIA	NM_006533	chr19	45973121	45975238	+	.	.	.	.	.	.	.	.	.	.	.	.	.	.	.	.	.	.	.	.
5037	1	RAB4B	NM_016154	chr19	45975963	45994689	+	.	.	.	.	.	.	.	.	.	.	.	.	.	.	.	.	.	.	.	.
3659	1	EGLN2	NM_080732	chr19	45996887	46006186	+	.	.	.	.	.	.	.	.	.	.	.	.	.	.	.	.	.	.	.	.
1338	2	CYP2A6	NM_000762	chr19	46041282	46048192	-	.	.	.	.	.	.	.	.	.	.	.	.	.	.	.	.	.	.	.	.
1339	2	CYP2A7	NM_030589	chr19	46073183	46080497	-	.	.	.	.	.	.	.	.	.	.	.	.	.	.	.	.	.	.	.	.
1026	2,25	CYP2B6	NM_000767	chr19	46189043	46216141	+	.	.	.	.	.	.	.	.	.	.	.	.	.	.	.	.	.	.	.	.
1025	2,25	CYP2A13	NM_000766	chr19	46286195	46293940	+	.	.	.	.	.	.	.	.	.	.	.	.	.	.	.	.	.	.	.	.
3510	1	CYP2F1	NM_000774	chr19	46312192	46326121	+	.	.	.	.	.	.	.	.	.	.	.	.	.	.	.	.	.	.	.	.
3511	1	CYP2S1	NM_030622	chr19	46390954	46405284	+	.	.	.	.	.	.	.	.	.	.	.	.	.	.	.	.	.	.	.	.
315	3,75	AXL	NM_001699	chr19	46416947	46459511	+	.	.	.	.	.	.	.	.	.	.	.	.	.	.	.	.	.	.	.	.
1805	2	TGFB1	NM_000660	chr19	46528651	46551671	-	.	.	.	.	.	.	.	.	.	.	.	.	.	.	.	.	.	.	.	.
3134	1	BCKDHA	NM_000709	chr19	46595533	46622750	+	.	.	.	.	.	.	.	.	.	.	.	.	.	.	.	.	.	.	.	.
2544	1,25	CEACAM5	NM_004363	chr19	46904369	46926277	+	.	.	.	.	.	.	.	.	.	.	.	.	.	.	.	.	.	.	.	.
2545	1,25	CEACAM6	NM_002483	chr19	46951237	46967953	+	.	.	.	.	.	.	.	.	.	.	.	.	.	.	.	.	.	.	.	.
1295	2	CD79A	NM_001783	chr19	47073029	47077279	+	.	.	.	.	.	.	.	.	.	.	.	.	.	.	.	.	.	.	.	.

Table S2

tumor associated genes								HIV				MLV				MMTV				MMTV(SIN)				MMTV(SIN)arrest			
rank	score	gene symbol	RefSEq	chrom	txStart	txEnd	strand	integrant	chr	position	strand	integrant	chr	position	strand	integrant	chr	position	strand	integrant	chr	position	strand	integrant	chr	position	strand
3066	1	ATP1A3	NM_001256213	chr19	47162573	47189459	-	.	.	.	.	.	.	.	.	.	.	.	.	.	.	.	.	.	.	.	.
6410	0,5	GRIK5	NM_002088	chr19	47194307	47261797	-	.	.	.	.	.	.	.	.	.	.	.	.	.	.	.	.	.	.	.	.
545	3	CIC	NM_015125	chr19	47480656	47491789	+	.	.	.	.	.	.	.	.	.	.	.	.	.	.	.	.	.	.	.	.
1593	2	PAFAH1B3	NM_001145940	chr19	47493024	47498792	-	.	.	.	.	.	.	.	.	.	.	.	.	.	.	.	.	.	.	.	.
6605	0,5	MEGF8	NM_001410	chr19	47521600	47574761	+	.	.	.	.	.	.	.	.	.	.	.	.	.	.	.	.	.	.	.	.
2068	1,5	CEACAM1	NM_001024912	chr19	47703297	47724501	-	.	.	.	.	.	.	.	.	.	.	.	.	.	.	.	.	.	.	.	.
2727	1,25	PSG1	NM_001184825	chr19	48062452	48075711	-	.	.	.	.	.	.	.	.	.	.	.	.	.	.	.	.	.	.	.	.
7490	0,25	PRG1	NR_026881	chr19	48545047	48545540	+	.	.	.	.	.	.	.	.	.	.	.	.	.	.	.	.	.	.	.	.
4789	1	PHLDB3	NM_198850	chr19	48671094	48700825	-	.	.	.	.	.	.	.	.	.	.	.	.	.	.	.	.	.	.	.	.
3701	1	ETHE1	NM_014297	chr19	48702710	48723236	-	.	.	.	.	.	.	.	.	.	.	.	.	.	.	.	.	.	.	.	.
5826	1	ZNF575	NM_174945	chr19	48729179	48732124	+	.	.	.	.	.	.	.	.	.	.	.	.	.	.	.	.	.	.	.	.
703	3	XRCC1	NM_006297	chr19	48739303	48771570	-	.	.	.	.	.	.	.	.	.	.	.	.	.	.	.	.	.	.	.	.
4166	1	IRGQ	NM_001007561	chr19	48780358	48792127	-	.	.	.	.	.	.	.	.	.	.	.	.	.	.	.	.	.	.	.	.
5827	1	ZNF576	NM_024327	chr19	48792383	48796427	+	.	.	.	.	.	.	.	.	.	.	.	.	.	.	.	.	.	.	.	.
5801	1	ZNF428	NM_182498	chr19	48803215	48815854	-	BH609826	chr19	48806176	+	.	.	.	.	.	.	.	.	.	.	.	.	.	.	.	.
3268	1	CADM4	NM_145296	chr19	48818361	48835831	-	.	.	.	.	.	.	.	.	.	.	.	.	.	.	.	.	.	.	.	.
1953	1,75	PLAUR	NM_002659	chr19	48844562	48866338	-	.	.	.	.	.	.	.	.	.	.	.	.	.	.	.	.	.	.	.	.
6584	0,5	LYPD5	NM_182573	chr19	48991918	49016648	-	.	.	.	.	.	.	.	.	.	.	.	.	.	.	.	.	.	.	.	.
5781	1	ZNF283	NM_181845	chr19	49023312	49044890	+	.	.	.	.	.	.	.	.	.	.	.	.	.	.	.	.	.	.	.	.
2479	1,5	ZNF45	NM_003425	chr19	49108615	49131251	-	.	.	.	.	.	.	.	.	.	.	.	.	.	.	.	.	.	.	.	.
5770	1	ZNF221	NM_013359	chr19	49147236	49163592	+	.	.	.	.	.	.	.	.	.	.	.	.	.	.	.	.	.	.	.	.
5763	1	ZNF155	NM_003445	chr19	49180161	49194317	+	AY517061.1	chr19	49184921	+	.	.	.	.	.	.	.	.	.	.	.	.	.	.	.	.
5776	1	ZNF230	NM_006300	chr19	49198916	49209912	+	.	.	.	.	.	.	.	.	.	.	.	.	.	.	.	.	.	.	.	.
5771	1	ZNF222	NM_001129996	chr19	49221333	49229102	+	.	.	.	.	.	.	.	.	.	.	.	.	.	.	.	.	.	.	.	.
5772	1	ZNF223	NM_013361	chr19	49248003	49263982	+	.	.	.	.	.	.	.	.	.	.	.	.	.	.	.	.	.	.	.	.
5782	1	ZNF284	NM_001037813	chr19	49268136	49283463	+	CL529384	chr19	49273636	+	.	.	.	.	.	.	.	.	.	.	.	.	.	.	.	.
5773	1	ZNF224	NM_013398	chr19	49290321	49304319	+	BH609821	chr19	49298252	+	.	.	.	.	.	.	.	.	.	.	.	.	.	.	.	.
5774	1	ZNF225	NM_013362	chr19	49309387	49329095	+	.	.	.	.	.	.	.	.	.	.	.	.	.	.	.	.	.	.	.	.
5777	1	ZNF234	NM_001144824	chr19	49337549	49356302	+	.	.	.	.	.	.	.	.	.	.	.	.	.	.	.	.	.	.	.	.
5775	1	ZNF226	NM_001032373	chr19	49361088	49373678	+	.	.	.	.	.	.	.	.	.	.	.	.	.	.	.	.	.	.	.	.
2473	1,5	ZNF227	NM_182490	chr19	49408530	49433261	+	.	.	.	.	.	.	.	.	.	.	.	.	.	.	.	.	.	.	.	.
2474	1,5	ZNF235	NM_004234	chr19	49482340	49501018	-	.	.	.	.	.	.	.	.	.	.	.	.	.	.	.	.	.	.	.	.
2470	1,5	ZFP112	NM_001083335	chr19	49522545	49552696	-	.	.	.	.	.	.	.	.	.	.	.	.	.	.	.	.	.	.	.	.
5766	1	ZNF180	NM_013256	chr19	49671698	49696414	-	.	.	.	.	.	.	.	.	.	.	.	.	.	.	.	.	.	.	.	.
5007	1	PVR	NM_001135770	chr19	49838937	49854202	+	.	.	.	.	.	.	.	.	.	.	.	.	.	.	.	.	.	.	.	.
3324	1	CEACAM19	NM_020219	chr19	49866563	49879467	+	.	.	.	.	.	.	.	.	.	.	.	.	.	.	.	.	.	.	.	.
237	4	BCL3	NM_005178	chr19	49943817	49955141	+	.	.	.	.	.	.	.	.	.	.	.	.	.	.	.	.	.	.	.	.
3283	1	CBLC	NM_012116	chr19	49972965	49995743	+	.	.	.	.	.	.	.	.	.	.	.	.	.	.	.	.	.	.	.	.
3130	1	BCAM	NM_001013257	chr19	50004155	50016518	+	.	.	.	.	.	.	.	.	.	.	.	.	.	.	.	.	.	.	.	.
5009	1	PVRL2	NM_001042724	chr19	50041232	50084325	+	AY517388.1	chr19	50058807	-	.	.	.	.	.	.	.	.	.	.	.	.	.	.	.	.
5522	1	TOMM40	NM_001128916	chr19	50086316	50098786	+	.	.	.	.	.	.	.	.	.	.	.	.	.	.	.	.	.	.	.	.
2002	1,5	APOE	NM_000041	chr19	50100878	50104490	+	.	.	.	.	.	.	.	.	.	.	.	.	.	.	.	.	.	.	.	.
6026	0,5	APOC2	NM_000483	chr19	50141078	50144662	+	.	.	.	.	.	.	.	.	.	.	.	.	.	.	.	.	.	.	.	.
3394	1	CLPTM1	NR_040007	chr19	50149681	50188444	+	.	.	.	.	.	.	.	.	.	.	.	.	.	.	.	.	.	.	.	.
2369	1,5	RELB	NM_006509	chr19	50196546	50233296	+	.	.	.	.	.	.	.	.	.	.	.	.	.	.	.	.	.	.	.	.
3874	1	GEMIN7	NM_024707	chr19	50274369	50286622	+	.	.	.	.	.	.	.	.	.	.	.	.	.	.	.	.	.	.	.	.
5542	1	TRAPPC6A	NM_001270891	chr19	50358025	50373341	-	.	.	.	.	.	.	.	.	.	.	.	.	.	.	.	.	.	.	.	.
3150	1	BLOC1S3	NM_212550	chr19	50373842	50376898	+	.	.	.	.	.	.	.	.	.	.	.	.	.	.	.	.	.	.	.	.
618	3	MARK4	NM_031417	chr19	50446355	50500381	+	.	.	.	.	.	.	.	.	.	.	.	.	.	.	.	.	.	.	.	.
2550	1,25	CKM	NM_001824	chr19	50501510	50518075	-	.	.	.	.	.	.	.	.	.	.	.	.	.	.	.	.	.	.	.	.
4232	1	KLC3	NM_177417	chr19	50535837	50546618	+	.	.	.	.	.	.	.	.	.	.	.	.	.	.	.	.	.	.	.	.

Table S2

tumor associated genes								HIV				MLV				MMTV				MMTV(SIN)				MMTV(SIN)arrest			
rank	score	gene symbol	RefSEq	chrom	txStart	txEnd	strand	integrant	chr	position	strand	integrant	chr	position	strand	integrant	chr	position	strand	integrant	chr	position	strand	integrant	chr	position	strand
15	9,75	ERCC2	NM_000400	chr19	50546488	50565685	-	.	.	.	.	.	.	.	.	.	.	.	.	.	.	.	.	.	.	.	.
291	4	PPP1R13L	NM_006663	chr19	50574731	50600152	-	.	.	.	.	.	.	.	.	.	.	.	.	.	.	.	.	.	.	.	.
2058	1,5	CD3EAP	NM_012099	chr19	50601306	50605864	+	.	.	.	.	.	.	.	.	.	.	.	.	.	.	.	.	.	.	.	.
208	4,25	ERCC1	NM_001983	chr19	50602430	50619017	-	.	.	.	.	.	.	.	.	.	.	.	.	.	.	.	.	.	.	.	.
6835	0,5	RTN2	NM_206900	chr19	50680385	50692153	-	.	.	.	.	.	.	.	.	.	.	.	.	.	.	.	.	.	.	.	.
7616	0,25	VASP	NM_003370	chr19	50702527	50722080	+	.	.	.	.	.	.	.	.	.	.	.	.	.	.	.	.	.	.	.	.
4664	1	OPA3	NM_025136	chr19	50741378	50779962	-	.	.	.	.	.	.	.	.	.	.	.	.	.	.	.	.	.	.	.	.
6943	0,5	SNRPD2	NM_004597	chr19	50882551	50887283	-	.	.	.	.	.	.	.	.	.	.	.	.	.	.	.	.	.	.	.	.
5248	1	SIX5	NM_175875	chr19	50959882	50964337	-	.	.	.	.	.	.	.	.	.	.	.	.	.	.	.	.	.	.	.	.
3592	1	DMPK	NM_001081560	chr19	50964815	50977655	-	.	.	.	.	.	.	.	.	.	.	.	.	.	.	.	.	.	.	.	.
6272	0,5	DMWD	NM_004943	chr19	50978103	50987900	-	.	.	.	.	.	.	.	.	.	.	.	.	.	.	.	.	.	.	.	.
2184	1,5	HIF3A	NM_152794	chr19	51498695	51538530	+	.	.	.	.	.	.	.	.	.	.	.	.	.	.	.	.	.	.	.	.
1677	2	PPP5C	NM_001204284	chr19	51542090	51586072	+	.	.	.	.	.	.	.	.	.	.	.	.	.	.	.	.	.	.	.	.
6758	0,5	PNMAL2	NM_020709	chr19	51686287	51691009	-	.	.	.	.	.	.	.	.	.	.	.	.	.	.	.	.	.	.	.	.
2047	1,5	CALM3	NM_005184	chr19	51796351	51805879	+	.	.	.	.	.	.	.	.	.	.	.	.	.	.	.	.	.	.	.	.
3926	1	GNG8	NM_033258	chr19	51829172	51829779	-	.	.	.	.	.	.	.	.	.	.	.	.	.	.	.	.	.	.	.	.
6251	0,5	DACT3	NM_145056	chr19	51842708	51856235	-	.	.	.	.	.	.	.	.	.	.	.	.	.	.	.	.	.	.	.	.
1687	2	PRKD2	NM_001079880	chr19	51869412	51912224	-	.	.	.	.	.	.	.	.	.	.	.	.	.	.	.	.	.	.	.	.
7182	0,25	BBC3	NM_014417	chr19	52415918	52426291	-	.	.	.	.	.	.	.	.	.	.	.	.	.	.	.	.	.	.	.	.
3233	1	C5AR1	NM_001736	chr19	52504943	52517167	+	.	.	.	.	.	.	.	.	.	.	.	.	.	.	.	.	.	.	.	.
6930	0,5	SLC8A2	NM_015063	chr19	52623090	52667119	-	.	.	.	.	.	.	.	.	.	.	.	.	.	.	.	.	.	.	.	.
3905	1	GLTSCR1	NM_015711	chr19	52803264	52898346	+	BH609828	chr19	52835268	-	.	.	.	.	.	.	.	.	.	.	.	.	.	.	.	.
6303	0,5	EHD2	NM_014601	chr19	52908412	52938203	+	.	.	.	.	.	.	.	.	.	.	.	.	.	.	.	.	.	.	.	.
6388	0,5	GLTSCR2	NM_015710	chr19	52940604	52952135	+	.	.	.	.	.	.	.	.	.	.	.	.	.	.	.	.	.	.	.	.
6873	0,5	SEPW1	NM_003009	chr19	52973653	52979755	+	.	.	.	.	.	.	.	.	.	.	.	.	.	.	.	.	.	.	.	.
5409	1	SULT2A1	NM_003167	chr19	53065534	53081466	-	.	.	.	.	.	.	.	.	.	.	.	.	.	.	.	.	.	.	.	.
1093	2,25	LIG1	NM_000234	chr19	53310514	53365372	-	.	.	.	.	.	.	.	.	.	.	.	.	.	.	.	.	.	.	.	.
5906	0,75	EMP3	NM_001425	chr19	53520440	53525622	+	.	.	.	.	.	.	.	.	.	.	.	.	.	.	.	.	.	.	.	.
3969	1	GRIN2D	NM_000836	chr19	53589943	53640000	+	.	.	.	.	.	.	.	.	.	.	.	.	.	.	.	.	.	.	.	.
2647	1,25	LMTK3	NM_001080434	chr19	53680339	53708258	-	.	.	.	.	.	.	.	.	.	.	.	.	.	.	.	.	.	.	.	.
1787	2	SULT2B1	NM_004605	chr19	53770804	53794496	+	.	.	.	.	.	.	.	.	.	.	.	.	.	.	.	.	.	.	.	.
5331	1	SPHK2	NM_001204159	chr19	53814601	53825475	+	.	.	.	.	.	.	.	.	.	.	.	.	.	.	.	.	.	.	.	.
3251	1	CA11	NM_001217	chr19	53833083	53841263	-	.	.	.	.	.	.	.	.	.	.	.	.	.	.	.	.	.	.	.	.
3817	1	FUT2	NM_000511	chr19	53891039	53901003	+	.	.	.	.	.	.	.	.	.	.	.	.	.	.	.	.	.	.	.	.
6798	0,5	RASIP1	NM_017805	chr19	53915653	53935782	-	.	.	.	.	.	.	.	.	.	.	.	.	.	.	.	.	.	.	.	.
577	3	FUT1	NM_000148	chr19	53943079	53950459	-	.	.	.	.	.	.	.	.	.	.	.	.	.	.	.	.	.	.	.	.
3132	1	BCAT2	NM_001190	chr19	53990130	54006132	-	.	.	.	.	.	.	.	.	.	.	.	.	.	.	.	.	.	.	.	.
6752	0,5	PLEKHA4	NM_020904	chr19	54032165	54063696	-	CL800307	chr19	54046365	-	.	.	.	.	.	.	.	.	.	.	.	.	.	.	.	.
2721	1,25	PPP1R15A	NM_014330	chr19	54067460	54071131	+	.	.	.	.	.	.	.	.	.	.	.	.	.	.	.	.	.	.	.	.
3573	1	DHDH	NM_014475	chr19	54128750	54140038	+	.	.	.	.	.	.	.	.	.	.	.	.	.	.	.	.	.	.	.	.
168	4,5	BAX	NM_004324	chr19	54149928	54156331	+	.	.	.	.	.	.	.	.	.	.	.	.	.	.	.	.	.	.	.	.
2385	1,5	RUVBL2	NM_006666	chr19	54188967	54210994	+	CL800443	chr19	54198381	+	.	.	.	.	.	.	.	.	.	.	.	.	.	.	.	.
2385	1,5	RUVBL2	NM_006666	chr19	54188967	54210994	+	CL799825	chr19	54197857	+	.	.	.	.	.	.	.	.	.	.	.	.	.	.	.	.
2549	1,25	CGB5	NM_033043	chr19	54238913	54240380	+	.	.	.	.	.	.	.	.	.	.	.	.	.	.	.	.	.	.	.	.
4897	1	PPFIA3	NM_003660	chr19	54314474	54346092	+	.	.	.	.	.	.	.	.	.	.	.	.	.	.	.	.	.	.	.	.
5960	0,75	SLC17A7	NM_020309	chr19	54624466	54636620	-	.	.	.	.	.	.	.	.	.	.	.	.	.	.	.	.	.	.	.	.
3788	1	FLT3LG	NM_001204503	chr19	54669629	54681300	+	.	.	.	.	.	.	.	.	.	.	.	.	.	.	.	.	.	.	.	.
2382	1,5	RRAS	NM_006270	chr19	54830363	54835212	-	.	.	.	.	.	.	.	.	.	.	.	.	.	.	.	.	.	.	.	.
5192	1	SCAF1	NM_021228	chr19	54837193	54853718	+	.	.	.	.	.	.	.	.	.	.	.	.	.	.	.	.	.	.	.	.
4165	1	IRF3	NM_001197128	chr19	54854637	54860944	-	.	.	.	.	.	.	.	.	.	.	.	.	.	.	.	.	.	.	.	.
7184	0,25	BCL2L12	NM_001040668	chr19	54860210	54868985	+	.	.	.	.	.	.	.	.	.	.	.	.	.	.	.	.	.	.	.	.

Table S2

tumor associated genes								HIV				MLV				MMTV				MMTV(SIN)				MMTV(SIN)arrest			
rank	score	gene symbol	RefSEq	chrom	txStart	txEnd	strand	integrant	chr	position	strand	integrant	chr	position	strand	integrant	chr	position	strand	integrant	chr	position	strand	integrant	chr	position	strand
655	3	PRMT1	NM_198318	chr19	54872220	54883519	+	.	.	.	.	.	.	.	.	.	.	.	.	.	.	.	.	.	.	.	.
3453	1	CPT1C	NM_001199753	chr19	54886176	54908800	+	.	.	.	.	.	.	.	.	.	.	.	.	.	.	.	.	.	.	.	.
6021	0,5	AP2A1	NM_014203	chr19	54961991	55002181	+	CL800705	chr19	54976909	-	.	.	.	.	.	.	.	.	.	.	.	.	.	.	.	.
4856	1	PNKP	NM_007254	chr19	55056271	55062634	-	.	.	.	.	.	.	.	.	.	.	.	.	.	.	.	.	.	.	.	.
7159	0,25	AKT1S1	NM_001098632	chr19	55064108	55072456	-	BH609830	chr19	55066614	-	.	.	.	.	.	.	.	.	.	.	.	.	.	.	.	.
6693	0,5	NUP62	NM_016553	chr19	55101893	55124800	-	CL800430	chr19	55121781	+	.	.	.	.	.	.	.	.	.	.	.	.	.	.	.	.
5681	1	VRK3	NM_016440	chr19	55171535	55220617	-	.	.	.	.	.	.	.	.	.	.	.	.	.	.	.	.	.	.	.	.
7444	0,25	NAPSA	NM_004851	chr19	55553545	55560743	-	.	.	.	.	.	.	.	.	.	.	.	.	.	.	.	.	.	.	.	.
4606	1	NR1H2	NM_007121	chr19	55571491	55578097	+	.	.	.	.	.	.	.	.	.	.	.	.	.	.	.	.	.	.	.	.
644	3	POLD1	NM_002691	chr19	55579391	55613087	+	AY517056.1	chr19	55595111	+	.	.	.	.	.	.	.	.	.	.	.	.	.	.	.	.
644	3	POLD1	NM_002691	chr19	55579391	55613087	+	BH609831	chr19	55588750	-	.	.	.	.	.	.	.	.	.	.	.	.	.	.	.	.
7002	0,5	SYT3	NM_001160328	chr19	55817045	55834904	-	.	.	.	.	.	.	.	.	.	.	.	.	.	.	.	.	.	.	.	.
2399	1,5	SHANK1	NM_016148	chr19	55856895	55912007	-	.	.	.	.	.	.	.	.	.	.	.	.	72_1_2277_9	chr19	55884382	+	.	.	.	.
7249	0,25	CLEC11A	NM_002975	chr19	55918416	55920793	+	.	.	.	.	.	.	.	.	.	.	.	.	.	.	.	.	.	.	.	.
2890	1	ACPT	NM_033068	chr19	55985483	55990293	+	.	.	.	.	.	.	.	.	.	.	.	.	.	.	.	.	.	.	.	.
4240	1	KLK15	NM_138563	chr19	56020356	56026591	-	.	.	.	.	.	.	.	.	.	.	.	.	.	.	.	.	.	.	.	.
735	2,75	KLK3	NM_001030047	chr19	56049982	56055832	+	.	.	.	.	.	.	.	.	.	.	.	.	.	.	.	.	.	.	.	.
1927	1,75	KLK5	NM_012427	chr19	56138370	56148156	-	.	.	.	.	.	.	.	.	.	.	.	.	.	.	.	.	.	.	.	.
1928	1,75	KLK6	NM_002774	chr19	56153698	56164741	-	.	.	.	.	.	.	.	.	.	.	.	.	.	.	.	.	.	.	.	.
876	2,5	KLK7	NM_001243126	chr19	56171546	56179132	-	.	.	.	.	.	.	.	.	.	.	.	.	.	.	.	.	.	.	.	.
2225	1,5	KLK8	NM_007196	chr19	56191075	56196770	-	.	.	.	.	.	.	.	.	.	.	.	.	.	.	.	.	.	.	.	.
4241	1	KLK9	NM_012315	chr19	56197580	56204702	-	.	.	.	.	.	.	.	.	.	.	.	.	.	.	.	.	.	.	.	.
1089	2,25	KLK10	NM_145888	chr19	56207811	56214766	-	.	.	.	.	.	.	.	.	.	.	.	.	.	.	.	.	.	.	.	.
2635	1,25	KLK11	NM_006853	chr19	56217298	56223102	-	.	.	.	.	.	.	.	.	.	.	.	.	.	.	.	.	.	.	.	.
4238	1	KLK13	NM_015596	chr19	56251274	56260179	-	.	.	.	.	.	.	.	.	.	.	.	.	.	.	.	.	.	.	.	.
4239	1	KLK14	NM_022046	chr19	56272965	56279314	-	.	.	.	.	.	.	.	.	.	.	.	.	.	.	.	.	.	.	.	.
6892	0,5	SIGLEC9	NM_014441	chr19	56319948	56325379	+	.	.	.	.	.	.	.	.	.	.	.	.	.	.	.	.	.	.	.	.
6891	0,5	SIGLEC7	NM_016543	chr19	56337369	56348595	+	.	.	.	.	.	.	.	.	.	.	.	.	.	.	.	.	.	.	.	.
4016	1	HAS1	NM_001523	chr19	56908176	56919033	-	.	.	.	.	.	.	.	.	.	.	.	.	.	.	.	.	.	.	.	.
3800	1	FPR3	NM_002030	chr19	56990222	57021146	+	.	.	.	.	.	.	.	.	.	.	.	.	.	.	.	.	.	.	.	.
5791	1	ZNF350	NM_021632	chr19	57159404	57181891	-	.	.	.	.	.	.	.	.	.	.	.	.	.	.	.	.	.	.	.	.
5836	1	ZNF615	NM_198480	chr19	57186398	57203295	-	.	.	.	.	.	.	.	.	.	.	.	.	.	.	.	.	.	.	.	.
5837	1	ZNF616	NM_178523	chr19	57309464	57335003	-	.	.	.	.	.	.	.	.	.	.	.	.	.	.	.	.	.	.	.	.
5865	1	ZNF836	NM_001102657	chr19	57349936	57366708	-	.	.	.	.	.	.	.	.	.	.	.	.	.	.	.	.	.	.	.	.
121	5,25	PPP2R1A	NR_033500	chr19	57384866	57421490	+	.	.	.	.	.	.	.	.	.	.	.	.	.	.	.	.	.	.	.	.
5853	1	ZNF766	NM_001010851	chr19	57464635	57487788	+	.	.	.	.	.	.	.	.	.	.	.	.	.	.	.	.	.	.	.	.
5810	1	ZNF480	NM_144684	chr19	57492237	57520986	+	.	.	.	.	.	.	.	.	.	.	.	.	.	.	.	.	.	.	.	.
5814	1	ZNF528	NM_032423	chr19	57592932	57613469	+	.	.	.	.	.	.	.	.	.	.	.	.	.	.	.	.	.	.	.	.
5828	1	ZNF578	NM_001099694	chr19	57648640	57711943	+	.	.	.	.	.	.	.	.	.	.	.	.	.	.	.	.	.	.	.	.
5860	1	ZNF808	NM_001039886	chr19	57722720	57751115	+	.	.	.	.	.	.	.	.	5_GVJ7CIC0	chr19	57722802	+	.	.	.	.	.	.	.	.
5847	1	ZNF701	NM_001172655	chr19	57765728	57782239	+	.	.	.	.	.	.	.	.	.	.	.	.	.	.	.	.	.	.	.	.
5863	1	ZNF83	NM_001242538	chr19	57807429	57885698	-	.	.	.	.	.	.	.	.	.	.	.	.	.	.	.	.	.	.	.	.
5835	1	ZNF611	NM_001161499	chr19	57897877	57930119	-	.	.	.	.	.	.	.	.	.	.	.	.	.	.	.	.	.	.	.	.
5834	1	ZNF600	NM_198457	chr19	57960559	57981846	-	.	.	.	.	.	.	.	.	.	.	.	.	.	.	.	.	.	.	.	.
5780	1	ZNF28	NR_036600	chr19	57992472	58016734	-	.	.	.	.	.	.	.	.	.	.	.	.	.	.	.	.	.	.	.	.
5807	1	ZNF468	NM_199132	chr19	58033596	58052714	-	.	.	.	.	.	.	.	.	.	.	.	.	.	.	.	.	.	.	.	.
5784	1	ZNF320	NM_207333	chr19	58071236	58086411	-	.	.	.	.	.	.	.	.	.	.	.	.	.	.	.	.	.	.	.	.
5764	1	ZNF160	NM_001102603	chr19	58261678	58298499	-	.	.	.	.	.	.	.	.	.	.	.	.	.	.	.	.	.	.	.	.
5796	1	ZNF415	NR_028343	chr19	58302943	58327985	-	.	.	.	.	.	.	.	.	.	.	.	.	.	.	.	.	.	.	.	.
5790	1	ZNF347	NM_032584	chr19	58333768	58354134	-	.	.	.	.	.	.	.	.	.	.	.	.	.	.	.	.	.	.	.	.
5843	1	ZNF665	NM_024733	chr19	58358363	58388431	-	.	.	.	.	AY516639	chr19	58379560	-	.	.	.	.	.	.	.	.	.	.	.	.

Table S2

tumor associated genes								HIV				MLV				MMTV				MMTV(SIN)				MMTV(SIN)arrest			
rank	score	gene symbol	RefSEq	chrom	txStart	txEnd	strand	integrant	chr	position	strand	integrant	chr	position	strand	integrant	chr	position	strand	integrant	chr	position	strand	integrant	chr	position	strand
5843	1	ZNF665	NM_024733	chr19	58358363	58388431	-	.	.	.	.	AY516641	chr19	58379560	-	.	.	.	.	.	.	.	.	.	.	.	.
5845	1	ZNF677	NM_182609	chr19	58430449	58449923	-	.	.	.	.	.	.	.	.	.	.	.	.	.	.	.	.	.	.	.	.
5852	1	ZNF765	NM_001040185	chr19	58590208	58607074	+	.	.	.	.	.	.	.	.	.	.	.	.	.	.	.	.	.	.	.	.
5851	1	ZNF761	NM_001008401	chr19	58627038	58653327	+	.	.	.	.	.	.	.	.	.	.	.	.	.	.	.	.	.	.	.	.
5861	1	ZNF813	NM_001004301	chr19	58662800	58689358	+	.	.	.	.	.	.	.	.	.	.	.	.	.	.	.	.	.	.	.	.
312	4	ZNF331	NM_001079907	chr19	58750372	58775335	+	.	.	.	.	.	.	.	.	.	.	.	.	.	.	.	.	.	.	.	.
4467	1	MYADM	NM_001020820	chr19	59061422	59071501	+	.	.	.	.	.	.	.	.	.	.	.	.	.	.	.	.	.	.	.	.
341	3,75	PRKCG	NM_002739	chr19	59077278	59102713	+	.	.	.	.	.	.	.	.	.	.	.	.	.	.	.	.	.	.	.	.
3267	1	CACNG7	NM_031896	chr19	59107802	59138781	+	.	.	.	.	.	.	.	.	.	.	.	.	.	.	.	.	.	.	.	.
1566	2	NDUFA3	NM_004542	chr19	59297971	59302093	+	.	.	.	.	.	.	.	.	.	.	.	.	.	.	.	.	.	.	.	.
691	3	TFPT	NM_013342	chr19	59302131	59310867	-	.	.	.	.	.	.	.	.	.	.	.	.	.	.	.	.	.	.	.	.
2347	1,5	PRPF31	NM_015629	chr19	59310601	59326962	+	.	.	.	.	.	.	.	.	.	.	.	.	.	.	.	.	.	.	.	.
3407	1	CNOT3	NM_014516	chr19	59333247	59351258	+	.	.	.	.	.	.	.	.	.	.	.	.	.	.	.	.	.	.	.	.
4276	1	LENG1	NM_024316	chr19	59351190	59355258	-	.	.	.	.	AY516023	chr19	59354497	+	.	.	.	.	.	.	.	.	.	.	.	.
4367	1	MBOAT7	NM_001146082	chr19	59373851	59385545	-	.	.	.	.	.	.	.	.	.	.	.	.	.	.	.	.	.	.	.	.
5568	1	TSEN34	NM_024075	chr19	59385930	59390206	+	.	.	.	.	.	.	.	.	.	.	.	.	.	.	.	.	.	.	.	.
6559	0,5	LILRB3	NM_001081450	chr19	59411958	59418771	-	.	.	.	.	.	.	.	.	.	.	.	.	.	.	.	.	.	.	.	.
6558	0,5	LILRB2	NM_005874	chr19	59469486	59476845	-	.	.	.	.	.	.	.	.	.	.	.	.	.	.	.	.	.	.	.	.
6545	0,5	LAIR1	NM_002287	chr19	59557046	59568533	-	.	.	.	.	.	.	.	.	.	.	.	.	.	.	.	.	.	.	.	.
7063	0,5	TTYH1	NM_001005367	chr19	59618416	59639711	+	.	.	.	.	.	.	.	.	.	.	.	.	.	.	.	.	.	.	.	.
7387	0,25	KIR3DL2	NM_001242867	chr19	60053709	60070482	+	.	.	.	.	.	.	.	.	.	.	.	.	.	.	.	.	.	.	.	.
4509	1	NCR1	NM_001242356	chr19	60109319	60116251	+	.	.	.	.	.	.	.	.	.	.	.	.	.	.	.	.	.	.	.	.
5085	1	RDH13	NM_138412	chr19	60247503	60272726	-	.	.	.	.	.	.	.	.	.	.	.	.	.	.	.	.	.	.	.	.
4910	1	PPP1R12C	NM_017607	chr19	60294092	60320739	-	.	.	.	.	.	.	.	.	.	.	.	.	.	.	.	.	.	.	.	.
7004	0,5	SYT5	NM_003180	chr19	60376280	60383532	-	.	.	.	.	.	.	.	.	.	.	.	.	.	.	.	.	.	.	.	.
1702	2	PTPRH	NM_001161440	chr19	60384426	60412686	-	.	.	.	.	.	.	.	.	.	.	.	.	.	.	.	.	.	.	.	.
5920	0,75	HSPBP1	NM_012267	chr19	60465402	60483563	-	.	.	.	.	.	.	.	.	.	.	.	.	.	.	.	.	.	.	.	.
2519	1,25	BRSK1	NM_032430	chr19	60487345	60515715	+	AY517249.1	chr19	60499709	+	.	.	.	.	.	.	.	.	.	.	.	.	.	.	.	.
5414	1	SUV420H2	NM_032701	chr19	60543032	60551301	+	.	.	.	.	.	.	.	.	.	.	.	.	.	.	.	.	.	.	.	.
3438	1	COX6B2	NM_144613	chr19	60552881	60557994	-	.	.	.	.	.	.	.	.	.	.	.	.	.	.	.	.	.	.	.	.
5608	1	UBE2S	NM_014501	chr19	60604461	60611137	-	.	.	.	.	.	.	.	.	.	.	.	.	.	.	.	.	.	.	.	.
7072	0,5	U2AF2	NM_007279	chr19	60857227	60877894	+	.	.	.	.	.	.	.	.	.	.	.	.	.	.	.	.	.	.	.	.
6316	0,5	EPN1	NM_013333	chr19	60878372	60898945	+	.	.	.	.	.	.	.	.	.	.	.	.	.	.	.	.	.	.	.	.
4587	1	NLRP4	NM_134444	chr19	61039755	61085032	+	.	.	.	.	.	.	.	.	.	.	.	.	.	.	.	.	.	.	.	.
4588	1	NLRP5	NM_153447	chr19	61202903	61264986	+	.	.	.	.	.	.	.	.	.	.	.	.	.	.	.	.	.	.	.	.
5859	1	ZNF787	NM_001002836	chr19	61290543	61324461	-	.	.	.	.	.	.	.	.	.	.	.	.	.	.	.	.	.	.	.	.
5804	1	ZNF444	NM_018337	chr19	61344346	61364074	+	.	.	.	.	.	.	.	.	.	.	.	.	.	.	.	.	.	.	.	.
5872	1	ZSCAN5A	NM_024303	chr19	61424490	61431471	-	.	.	.	.	.	.	.	.	.	.	.	.	.	.	.	.	.	.	.	.
5829	1	ZNF582	NM_144690	chr19	61586459	61596701	-	.	.	.	.	.	.	.	.	.	.	.	.	.	.	.	.	.	.	.	.
5830	1	ZNF583	NM_001159860	chr19	61607194	61628212	+	.	.	.	.	.	.	.	.	.	.	.	.	.	.	.	.	.	.	.	.
5844	1	ZNF667	NR_030740	chr19	61642504	61680582	-	.	.	.	.	.	.	.	.	.	.	.	.	.	.	.	.	.	.	.	.
5809	1	ZNF471	NM_020813	chr19	61711023	61732081	+	.	.	.	.	.	.	.	.	.	.	.	.	.	.	.	.	.	.	.	.
5742	1	ZFP28	NM_020828	chr19	61742128	61759982	+	CL799818	chr19	61754085	-	.	.	.	.	.	.	.	.	.	.	.	.	.	.	.	.
5808	1	ZNF470	NM_001001668	chr19	61770701	61786074	+	.	.	.	.	.	.	.	.	.	.	.	.	.	.	.	.	.	.	.	.
5849	1	ZNF71	NM_021216	chr19	61798475	61827356	+	.	.	.	.	.	.	.	.	.	.	.	.	.	.	.	.	.	.	.	.
5864	1	ZNF835	NM_001005850	chr19	61865831	61874935	-	.	.	.	.	.	.	.	.	.	.	.	.	.	.	.	.	.	.	.	.
5751	1	ZIM2	NM_001146326	chr19	61977739	62043909	-	.	.	.	.	.	.	.	.	.	.	.	.	.	.	.	.	.	.	.	.
915	2,5	PEG3	NM_001146186	chr19	62013256	62028186	-	.	.	.	.	.	.	.	.	.	.	.	.	.	.	.	.	.	.	.	.
2475	1,5	ZNF264	NM_003417	chr19	62394679	62426026	+	.	.	.	.	.	.	.	.	.	.	.	.	.	.	.	.	.	.	.	.
1000	2,25	AURKC	NM_003160	chr19	62434188	62438728	+	.	.	.	.	.	.	.	.	.	.	.	.	.	.	.	.	.	.	.	.
5805	1	ZNF460	NM_006635	chr19	62483664	62497248	+	BH609833	chr19	62486093	+	.	.	.	.	.	.	.	.	.	.	.	.	.	.	.	.

Table S2

tumor associated genes								HIV				MLV				MMTV				MMTV(SIN)				MMTV(SIN)arrest			
rank	score	gene symbol	RefSEq	chrom	txStart	txEnd	strand	integrant	chr	position	strand	integrant	chr	position	strand	integrant	chr	position	strand	integrant	chr	position	strand	integrant	chr	position	strand
5816	1	ZNF543	NM_213598	chr19	62523676	62533956	+	.	.	.	.	.	.	.	.	.	.	.	.	.	.	.	.	.	.	.	.
2476	1,5	ZNF304	NM_020657	chr19	62554456	62563077	+	.	.	.	.	.	.	.	.	.	.	.	.	.	.	.	.	.	.	.	.
5818	1	ZNF547	NM_173631	chr19	62566702	62582737	+	.	.	.	.	.	.	.	.	.	.	.	.	.	.	.	.	.	.	.	.
5819	1	ZNF548	NM_152909	chr19	62593029	62605731	+	.	.	.	.	.	.	.	.	.	.	.	.	.	.	.	.	.	.	.	.
5765	1	ZNF17	NM_006959	chr19	62614340	62625119	+	.	.	.	.	.	.	.	.	.	.	.	.	.	.	.	.	.	.	.	.
5850	1	ZNF749	NM_001023561	chr19	62638504	62649003	+	.	.	.	.	.	.	.	.	.	.	.	.	.	.	.	.	.	.	.	.
5676	1	VN1R1	NM_020633	chr19	62658353	62659919	-	.	.	.	.	.	.	.	.	.	.	.	.	.	.	.	.	.	.	.	.
5855	1	ZNF772	NM_001024596	chr19	62672765	62680750	-	.	.	.	.	.	.	.	.	.	.	.	.	.	.	.	.	.	.	.	.
2477	1,5	ZNF419	NM_001098493	chr19	62690890	62697860	+	.	.	.	.	.	.	.	.	.	.	.	.	.	.	.	.	.	.	.	.
5856	1	ZNF773	NM_198542	chr19	62703120	62711322	+	.	.	.	.	.	.	.	.	.	.	.	.	.	.	.	.	.	.	.	.
5820	1	ZNF549	NM_153263	chr19	62730504	62744056	+	.	.	.	.	.	.	.	.	.	.	.	.	.	.	.	.	.	.	.	.
5821	1	ZNF550	NM_001039654	chr19	62750154	62759537	-	.	.	.	.	.	.	.	.	.	.	.	.	.	.	.	.	.	.	.	.
5797	1	ZNF416	NM_017879	chr19	62774745	62782055	-	.	.	.	.	.	.	.	.	.	.	.	.	.	.	.	.	.	.	.	.
5750	1	ZIK1	NM_001010879	chr19	62787439	62795570	+	.	.	.	.	.	.	.	.	.	.	.	.	.	.	.	.	.	.	.	.
5815	1	ZNF530	NM_020880	chr19	62803064	62811449	+	.	.	.	.	.	.	.	.	.	.	.	.	.	.	.	.	.	.	.	.
2471	1,5	ZNF134	NM_003435	chr19	62817641	62825448	+	.	.	.	.	.	.	.	.	.	.	.	.	.	.	.	.	.	.	.	.
2472	1,5	ZNF211	NM_001265597	chr19	62836346	62845959	+	.	.	.	.	.	.	.	.	.	.	.	.	.	.	.	.	.	.	.	.
5822	1	ZNF551	NM_138347	chr19	62885148	62892981	+	.	.	.	.	.	.	.	.	.	.	.	.	.	.	.	.	.	.	.	.
5762	1	ZNF154	NM_001085384	chr19	62903621	62912391	-	.	.	.	.	.	.	.	.	.	.	.	.	.	.	.	.	.	.	.	.
2481	1,5	ZNF671	NM_024833	chr19	62922930	62930807	-	.	.	.	.	.	.	.	.	.	.	.	.	.	.	.	.	.	.	.	.
5857	1	ZNF776	NM_173632	chr19	62949975	62961339	+	.	.	.	.	.	.	.	.	.	.	.	.	.	.	.	.	.	.	.	.
5832	1	ZNF586	NM_017652	chr19	62972831	62983796	+	.	.	.	.	.	.	.	.	.	.	.	.	.	.	.	.	.	.	.	.
5823	1	ZNF552	NM_024762	chr19	63010261	63018093	-	.	.	.	.	.	.	.	.	.	.	.	.	.	.	.	.	.	.	.	.
5833	1	ZNF587	NM_001204817	chr19	63052992	63068303	+	.	.	.	.	.	.	.	.	.	.	.	.	.	.	.	.	.	.	.	.
5798	1	ZNF417	NM_152475	chr19	63108953	63119790	-	.	.	.	.	.	.	.	.	.	.	.	.	.	.	.	.	.	.	.	.
5799	1	ZNF418	NM_133460	chr19	63125063	63138552	-	.	.	.	.	.	.	.	.	.	.	.	.	.	.	.	.	.	.	.	.
5779	1	ZNF256	NM_005773	chr19	63144012	63150889	-	.	.	.	.	.	.	.	.	.	.	.	.	.	.	.	.	.	.	.	.
3200	1	C19orf18	NM_152474	chr19	63161616	63177714	-	.	.	.	.	.	.	.	.	.	.	.	.	.	.	.	.	.	.	.	.
2480	1,5	ZNF606	NM_025027	chr19	63180252	63206526	-	.	.	.	.	.	.	.	.	.	.	.	.	.	.	.	.	.	.	.	.
5760	1	ZNF135	NM_007134	chr19	63262418	63272922	+	.	.	.	.	.	.	.	.	.	.	.	.	.	.	.	.	.	.	.	.
5869	1	ZSCAN18	NM_023926	chr19	63287020	63301542	-	.	.	.	.	.	.	.	.	.	.	.	.	1316_92	chr19	63295809	+	.	.	.	.
5817	1	ZNF544	NM_014480	chr19	63431881	63466820	+	.	.	.	.	.	.	.	.	.	.	.	.	.	.	.	.	.	.	.	.
2482	1,5	ZNF8	NM_021089	chr19	63482129	63499066	+	.	.	.	.	.	.	.	.	.	.	.	.	.	.	.	.	.	.	.	.
5871	1	ZSCAN22	NM_181846	chr19	63530196	63545524	+	.	.	.	.	.	.	.	.	.	.	.	.	.	.	.	.	.	.	.	.
1870	2	ZNF497	NM_198458	chr19	63557534	63566026	-	.	.	.	.	.	.	.	.	.	.	.	.	.	.	.	.	.	.	.	.
5831	1	ZNF584	NM_173548	chr19	63611874	63621504	+	AY517181.1	chr19	63621200	-	.	.	.	.	.	.	.	.	.	.	.	.	.	.	.	.
5758	1	ZNF132	NM_003433	chr19	63635992	63643401	-	.	.	.	.	.	.	.	.	.	.	.	.	.	.	.	.	.	.	.	.
5787	1	ZNF324B	NM_207395	chr19	63654782	63661011	+	.	.	.	.	.	.	.	.	.	.	.	.	.	.	.	.	.	.	.	.
5786	1	ZNF324	NM_014347	chr19	63670223	63676757	+	.	.	.	.	.	.	.	.	.	.	.	.	.	.	.	.	.	.	.	.
2478	1,5	ZNF446	NM_017908	chr19	63679606	63684409	+	.	.	.	.	.	.	.	.	.	.	.	.	.	.	.	.	.	.	.	.
1761	2	SLC27A5	NM_012254	chr19	63701511	63715244	-	.	.	.	.	.	.	.	.	.	.	.	.	.	.	.	.	.	.	.	.
5733	1	ZBTB45	NM_032792	chr19	63716708	63722733	-	.	.	.	.	.	.	.	.	.	.	.	.	.	.	.	.	.	.	.	.
5553	1	TRIM28	NM_005762	chr19	63747647	63753894	+	AY517462.1	chr19	63753218	+	.	.	.	.	.	.	.	.	.	.	.	.	.	.	.	.
3355	1	CHMP2A	NM_198426	chr19	63754744	63758167	-	.	.	.	.	.	.	.	.	.	.	.	.	.	.	.	.	.	.	.	.
698	3	UBE2M	NM_003969	chr19	63758890	63762155	-	.	.	.	.	.	.	.	.	.	.	.	.	.	.	.	.	.	.	.	.
4483	1	MZF1	NM_198055	chr19	63765095	63776754	-	.	.	.	.	.	.	.	.	.	.	.	.	.	.	.	.	.	.	.	.
6611	0,5	MGC2752	NR_026052	chr19	63778577	63787574	+	.	.	.	.	.	.	.	.	.	.	.	.	.	.	.	.	.	.	.	.
3224	1	C20orf96	NM_153269	chr20	199503	219419	-	.	.	.	.	.	.	.	.	.	.	.	.	.	.	.	.	.	.	.	.
5735	1	ZCCHC3	NM_033089	chr20	226203	228963	+	.	.	.	.	.	.	.	.	.	.	.	.	.	.	.	.	.	.	.	.
2782	1,25	SOX12	NM_006943	chr20	254238	258867	+	.	.	.	.	.	.	.	.	.	.	.	.	.	.	.	.	.	.	.	.
4624	1	NRSN2	NM_024958	chr20	275369	283512	+	.	.	.	.	.	.	.	.	.	.	.	.	.	.	.	.	.	.	.	.

Table S2

tumor associated genes								HIV				MLV				MMTV				MMTV(SIN)				MMTV(SIN)arrest			
rank	score	gene symbol	RefSEq	chrom	txStart	txEnd	strand	integrant	chr	position	strand	integrant	chr	position	strand	integrant	chr	position	strand	integrant	chr	position	strand	integrant	chr	position	strand
2365	1,5	RBCK1	NM_006462	chr20	336708	359610	+	.	.	.	.	.	.	.	.	.	.	.	.	.	.	.	.	.	.	.	.
5434	1	TBC1D20	NM_144628	chr20	364123	391187	-	.	.	.	.	.	.	.	.	.	.	.	.	.	.	.	.	.	.	.	.
455	3,25	CSNK2A1	NM_177560	chr20	411337	472482	-	.	.	.	.	.	.	.	.	.	.	.	.	.	.	.	.	.	.	.	.
5355	1	SRXN1	NM_080725	chr20	575267	581890	-	.	.	.	.	.	.	.	.	.	.	.	.	.	.	.	.	.	.	.	.
7163	0,25	ANGPT4	NM_015985	chr20	801296	844960	-	.	.	.	.	.	.	.	.	.	.	.	.	.	.	.	.	.	.	.	.
4973	1	PSMF1	NM_178578	chr20	1041905	1096426	+	.	.	.	.	.	.	.	.	.	.	.	.	.	.	.	.	.	.	.	.
6942	0,5	SNPH	NM_014723	chr20	1194959	1237971	+	.	.	.	.	.	.	.	.	.	.	.	.	.	.	.	.	.	.	.	.
5201	1	SDCBP2	NM_015685	chr20	1238554	1242317	-	.	.	.	.	.	.	.	.	.	.	.	.	.	.	.	.	.	.	.	.
1405	2	FKBP1A	NM_001199786	chr20	1297620	1321816	-	.	.	.	.	.	.	.	.	.	.	.	.	.	.	.	.	.	.	.	.
4630	1	NSFL1C	NM_016143	chr20	1370806	1396337	-	.	.	.	.	.	.	.	.	.	.	.	.	.	.	.	.	.	.	.	.
6893	0,5	SIRPA	NM_080792	chr20	1823424	1868540	+	.	.	.	.	.	.	.	.	.	.	.	.	.	.	.	.	.	.	.	.
5943	0,75	PDYN	NM_024411	chr20	1907401	1922931	-	.	.	.	.	.	.	.	.	.	.	.	.	.	.	.	.	.	.	.	.
5385	1	STK35	NM_080836	chr20	2030527	2077198	+	.	.	.	.	.	.	.	.	.	.	.	.	.	.	.	.	.	.	.	.
1806	2	TGM3	NM_003245	chr20	2224612	2269725	+	.	.	.	.	.	.	.	.	.	.	.	.	.	.	.	.	.	.	.	.
4109	1	IDH3B	NM_001258384	chr20	2587660	2592865	-	.	.	.	.	.	.	.	.	.	.	.	.	.	.	.	.	.	.	.	.
7088	0,5	VPS16	NM_080413	chr20	2769372	2795378	+	CL529608	chr20	2777491	-	.	.	.	.	.	.	.	.	.	.	.	.	.	.	.	.
423	3,5	PTPRA	NM_080841	chr20	2851852	2967315	+	CL800132	chr20	2815070	+	.	.	.	.	.	.	.	.	.	.	.	.	.	.	.	.
423	3,5	PTPRA	NM_080841	chr20	2851852	2967315	+	CL529560	chr20	2856873	-	.	.	.	.	.	.	.	.	.	.	.	.	.	.	.	.
3933	1	GNRH2	NM_178331	chr20	2972267	2974391	+	.	.	.	.	.	.	.	.	.	.	.	.	.	.	.	.	.	.	.	.
2508	1,25	AVP	NM_000490	chr20	3011201	3013370	-	.	.	.	.	.	.	.	.	.	.	.	.	.	.	.	.	.	.	.	.
3742	1	FASTKD5	NM_021826	chr20	3075164	3088532	-	.	.	.	.	.	.	.	.	.	.	.	.	.	.	.	.	.	.	.	.
4181	1	ITPA	NM_033453	chr20	3138005	3152516	+	.	.	.	.	.	.	.	.	.	.	.	.	.	.	.	.	.	.	.	.
5241	1	SIGLEC1	NM_023068	chr20	3615616	3635775	-	.	.	.	.	.	.	.	.	.	.	.	.	.	.	.	.	.	.	.	.
6453	0,5	HSPA12B	NM_052970	chr20	3661316	3681758	+	.	.	.	.	.	.	.	.	.	.	.	.	.	.	.	.	.	.	.	.
3328	1	CENPB	NM_001810	chr20	3712497	3715337	-	.	.	.	.	.	.	.	.	.	.	.	.	.	.	.	.	.	.	.	.
1012	2,25	CDC25B	NM_021873	chr20	3724400	3734761	+	.	.	.	.	.	.	.	.	.	.	.	.	.	.	.	.	.	.	.	.
4694	1	PANK2	NM_024960	chr20	3817485	3852502	+	CL529200	chr20	3841318	+	.	.	.	.	.	.	.	.	.	.	.	.	.	.	.	.
4694	1	PANK2	NM_024960	chr20	3817485	3852502	+	CL800382	chr20	3832953	+	.	.	.	.	.	.	.	.	.	.	.	.	.	.	.	.
2777	1,25	SMOX	NM_175842	chr20	4077425	4116394	+	.	.	.	.	.	.	.	.	.	.	.	.	.	.	.	.	.	.	.	.
2914	1	ADRA1D	NM_000678	chr20	4149277	4177659	-	.	.	.	.	.	.	.	.	.	.	.	.	.	.	.	.	.	.	.	.
4950	1	PRNP	NM_183079	chr20	4614796	4630234	+	.	.	.	.	.	.	.	.	.	.	.	.	.	.	.	.	.	.	.	.
7502	0,25	RASSF2	NM_170774	chr20	4708668	4743769	-	.	.	.	.	.	.	.	.	.	.	.	.	.	.	.	.	.	.	.	.
187	4,5	PCNA	NM_002592	chr20	5043598	5055268	-	.	.	.	.	.	.	.	.	.	.	.	.	.	.	.	.	.	.	.	.
3323	1	CDS2	NM_003818	chr20	5055406	5126533	+	.	.	.	.	.	.	.	.	.	.	.	.	.	.	.	.	.	.	.	.
3352	1	CHGB	NM_001819	chr20	5839973	5854005	+	.	.	.	.	.	.	.	.	.	.	.	.	.	.	.	.	.	.	.	.
3462	1	CRLS1	NM_001127458	chr20	5935897	5968697	+	AY517099.1	chr20	5948205	-	.	.	.	.	.	.	.	.	.	.	.	.	.	.	.	.
2140	1,5	FERMT1	NM_017671	chr20	6003491	6052191	-	.	.	.	.	.	.	.	.	.	.	.	.	.	.	.	.	.	.	.	.
5882	0,75	BMP2	NM_001200	chr20	6696744	6708910	+	.	.	.	.	.	.	.	.	.	.	.	.	.	.	.	.	.	.	.	.
4011	1	HAO1	NM_017545	chr20	7811630	7869093	-	.	.	.	.	.	.	.	.	.	.	.	.	.	.	.	.	.	.	.	.
144	5	PLCB1	NM_182734	chr20	8061295	8813547	+	CL799745	chr20	8210356	+	.	.	.	.	.	.	.	.	.	.	.	.	.	.	.	.
415	3,5	PLCB4	NM_001172646	chr20	8997700	9409462	+	.	.	.	.	AY515993	chr20	9305239	-	.	.	.	.	.	.	.	.	.	.	.	.
287	4	PAK7	NM_020341	chr20	9466036	9767687	-	.	.	.	.	.	.	.	.	.	.	.	.	.	.	.	.	.	.	.	.
5303	1	SNAP25	NM_003081	chr20	10147476	10236066	+	.	.	.	.	.	.	.	.	.	.	.	.	.	.	.	.	.	.	.	.
4184	1	JAG1	NM_000214	chr20	10566331	10602694	-	.	.	.	.	.	.	.	.	.	.	.	.	.	.	.	.	.	.	.	.
1513	2	MACROD2	NM_080676	chr20	13924145	15981841	+	.	.	.	.	AY516798	chr20	14596333	+	.	.	.	.	1822_198	chr20	15707726	-	.	.	.	.
1513	2	MACROD2	NM_080676	chr20	13924145	15981841	+	.	.	.	.	AY516152	chr20	14313832	-	.	.	.	.	1822_198	chr20	15707726	-	.	.	.	.
6359	0,5	FLRT3	NM_013281	chr20	14252638	14266313	-	.	.	.	.	.	.	.	.	.	.	.	.	.	.	.	.	.	.	.	.
4228	1	KIF16B	NM_001199866	chr20	16295487	16502079	-	.	.	.	.	.	.	.	.	.	.	.	.	.	.	.	.	.	.	.	.
5308	1	SNRPB2	NM_003092	chr20	16658608	16670417	+	.	.	.	.	.	.	.	.	.	.	.	.	.	.	.	.	.	.	.	.
2310	1,5	PCSK2	NM_001201529	chr20	17155596	17413222	+	.	.	.	.	.	.	.	.	.	.	.	.	.	.	.	.	.	.	.	.
3611	1	DSTN	NM_001011546	chr20	17498598	17536652	+	.	.	.	.	.	.	.	.	.	.	.	.	.	.	.	.	.	.	.	.

Table S2

tumor associated genes								HIV				MLV				MMTV				MMTV(SIN)				MMTV(SIN)arrest			
rank	score	gene symbol	RefSEq	chrom	txStart	txEnd	strand	integrant	chr	position	strand	integrant	chr	position	strand	integrant	chr	position	strand	integrant	chr	position	strand	integrant	chr	position	strand
5168	1	RRBP1	NM_004587	chr20	17542322	17610928	-	.	.	.	.	.	.	.	.	.	.	.	.	.	.	.	.	.	.	.	.
5311	1	SNX5	NM_014426	chr20	17870243	17897154	-	.	.	.	.	.	.	.	.	.	.	.	.	.	.	.	.	.	.	.	.
3222	1	C20orf72	NM_052865	chr20	17897761	17919762	+	.	.	.	.	.	.	.	.	.	.	.	.	.	.	.	.	.	.	.	.
4676	1	OVOL2	NM_021220	chr20	17952795	17986521	-	.	.	.	.	.	.	.	.	.	.	.	.	.	.	.	.	.	.	.	.
3474	1	CSRP2BP	NR_028402	chr20	18073742	18117031	+	.	.	.	.	.	.	.	.	.	.	.	.	.	.	.	.	.	.	.	.
5759	1	ZNF133	NM_003434	chr20	18217120	18245640	+	.	.	.	.	.	.	.	.	.	.	.	.	.	.	.	.	.	.	.	.
1658	2	POLR3F	NM_006466	chr20	18396032	18413286	+	.	.	.	.	.	.	.	.	.	.	.	.	.	.	.	.	.	.	.	.
5071	1	RBBP9	NM_006606	chr20	18415187	18425887	-	.	.	.	.	.	.	.	.	.	.	.	.	.	.	.	.	.	.	.	.
5205	1	SEC23B	NM_006363	chr20	18436187	18490050	+	.	.	.	.	.	.	.	.	.	.	.	.	.	.	.	.	.	.	.	.
3613	1	DTD1	NM_080820	chr20	18516555	18692560	+	.	.	.	.	.	.	.	.	.	.	.	.	.	.	.	.	.	.	.	.
5261	1	SLC24A3	NM_020689	chr20	19141289	19651541	+	.	.	.	.	.	.	.	.	.	.	.	.	.	.	.	.	.	.	.	.
3463	1	CRNKL1	NM_016652	chr20	19963011	19984690	-	.	.	.	.	.	.	.	.	.	.	.	.	.	.	.	.	.	.	.	.
3218	1	C20orf26	NM_015585	chr20	19981157	20289346	+	CL529201	chr20	20122578	-	.	.	.	.	.	.	.	.	.	.	.	.	.	.	.	.
6479	0,5	INSM1	NM_002196	chr20	20296764	20299592	+	.	.	.	.	.	.	.	.	.	.	.	.	.	.	.	.	.	.	.	.
2835	1,25	XRN2	NM_012255	chr20	21231941	21318463	+	.	.	.	.	.	.	.	.	.	.	.	.	.	.	.	.	.	.	.	.
6674	0,5	NKX2-2	NM_002509	chr20	21439659	21442664	-	.	.	.	.	.	.	.	.	.	.	.	.	.	.	.	.	.	.	.	.
2588	1,25	FOXA2	NM_021784	chr20	22509641	22513101	-	.	.	.	.	.	.	.	.	.	.	.	.	.	.	.	.	.	.	.	.
967	2,5	THBD	NM_000361	chr20	22974269	22978301	-	.	.	.	.	.	.	.	.	.	.	.	.	.	.	.	.	.	.	.	.
3303	1	CD93	NM_012072	chr20	23007992	23014977	-	.	.	.	.	.	.	.	.	.	.	.	.	.	.	.	.	.	.	.	.
4651	1	NXT1	NM_013248	chr20	23279372	23283408	+	.	.	.	.	.	.	.	.	.	.	.	.	.	.	.	.	.	.	.	.
3998	1	GZF1	NM_022482	chr20	23293020	23301683	+	.	.	.	.	.	.	.	.	.	.	.	.	.	.	.	.	.	.	.	.
6647	0,5	NAPB	NM_022080	chr20	23303155	23350156	-	.	.	.	.	.	.	.	.	.	.	.	.	.	.	.	.	.	.	.	.
3479	1	CST8	NM_005492	chr20	23419765	23424655	+	.	.	.	.	.	.	.	.	.	.	.	.	.	.	.	.	.	.	.	.
1330	2	CST3	NM_000099	chr20	23562293	23566574	-	.	.	.	.	.	.	.	.	.	.	.	.	.	.	.	.	.	.	.	.
3476	1	CST4	NM_001899	chr20	23614276	23617662	-	.	.	.	.	.	.	.	.	.	.	.	.	.	.	.	.	.	.	.	.
3475	1	CST2	NM_001322	chr20	23752403	23755312	-	.	.	.	.	.	.	.	.	.	.	.	.	.	.	.	.	.	.	.	.
3477	1	CST5	NM_001900	chr20	23804571	23808380	-	.	.	.	.	.	.	.	.	.	.	.	.	.	.	.	.	.	.	.	.
3478	1	CST7	NM_003650	chr20	24877865	24888564	+	.	.	.	.	.	.	.	.	.	.	.	.	.	.	.	.	.	.	.	.
1204	2	ACSS1	NM_001252676	chr20	24934865	24961342	-	.	.	.	.	.	.	.	.	.	.	.	.	.	.	.	.	.	.	.	.
2458	1,5	VSX1	NR_045948	chr20	24999520	25011015	-	.	.	.	.	.	.	.	.	.	.	.	.	.	.	.	.	.	.	.	.
1385	2	ENTPD6	NM_001114089	chr20	25124338	25155360	+	CL529202	chr20	25129044	+	.	.	.	.	.	.	.	.	.	.	.	.	.	.	.	.
1706	2	PYGB	NM_002862	chr20	25176705	25226648	+	.	.	.	.	.	.	.	.	.	.	.	.	.	.	.	.	.	.	.	.
2859	1	ABHD12	NM_015600	chr20	25223378	25319618	-	.	.	.	.	.	.	.	.	.	.	.	.	.	.	.	.	.	.	.	.
3890	1	GINS1	NM_021067	chr20	25336322	25377191	+	.	.	.	.	.	.	.	.	.	.	.	.	.	.	.	.	.	.	.	.
4493	1	NANP	NM_152667	chr20	25541572	25552648	-	.	.	.	.	.	.	.	.	.	.	.	.	.	.	.	.	.	.	.	.
5788	1	ZNF337	NM_015655	chr20	25602850	25625469	-	.	.	.	.	.	.	.	.	.	.	.	.	.	.	.	.	.	.	.	.
5094	1	REM1	NM_014012	chr20	29526765	29536369	+	.	.	.	.	.	.	.	.	.	.	.	.	.	.	.	.	.	.	.	.
859	2,5	ID1	NM_002165	chr20	29656746	29657978	+	.	.	.	.	.	.	.	.	.	.	.	.	.	.	.	.	.	.	.	.
3434	1	COX4I2	NM_032609	chr20	29689351	29696461	+	.	.	.	.	.	.	.	.	.	.	.	.	.	.	.	.	.	.	.	.
359	3,5	BCL2L1	NM_001191	chr20	29715921	29774317	-	.	.	.	.	.	.	.	.	.	.	.	.	.	.	.	.	.	.	.	.
7600	0,25	TPX2	NM_012112	chr20	29790564	29853264	+	.	.	.	.	.	.	.	.	.	.	.	.	.	.	.	.	.	.	.	.
2266	1,5	MYLK2	NM_033118	chr20	29870838	29886161	+	.	.	.	.	.	.	.	.	.	.	.	.	.	.	.	.	.	.	.	.
3620	1	DUSP15	NM_001012644	chr20	29912530	29921462	-	.	.	.	.	.	.	.	.	.	.	.	.	.	.	.	.	.	.	.	.
6094	0,5	C20orf160	NM_080625	chr20	30061905	30083645	+	.	.	.	.	.	.	.	.	.	.	.	.	.	.	.	.	.	.	.	.
1441	2	HCK	NM_001172131	chr20	30103651	30153318	+	.	.	.	.	.	.	.	.	.	.	.	.	.	.	.	.	.	.	.	.
7589	0,25	TM9SF4	NM_014742	chr20	30160969	30218722	+	.	.	.	.	.	.	.	.	.	.	.	.	.	.	.	.	.	.	.	.
7481	0,25	PLAGL2	NM_002657	chr20	30243967	30259207	-	.	.	.	.	.	.	.	.	.	.	.	.	.	.	.	.	.	.	.	.
1136	2,25	POFUT1	NM_015352	chr20	30259356	30290128	+	.	.	.	.	.	.	.	.	.	.	.	.	.	.	.	.	.	.	.	.
6533	0,5	KIF3B	NM_004798	chr20	30329114	30386472	+	.	.	.	.	.	.	.	.	.	.	.	.	.	.	.	.	.	.	.	.
7176	0,25	ASXL1	NM_015338	chr20	30409807	30490783	+	.	.	.	.	.	.	.	.	.	.	.	.	.	.	.	.	.	.	.	.
7209	0,25	C20orf112	NM_001256798	chr20	30494522	30636536	-	.	.	.	.	.	.	.	.	.	.	.	.	.	.	.	.	.	.	.	.

Table S2

tumor associated genes								HIV				MLV				MMTV				MMTV(SIN)				MMTV(SIN)arrest			
rank	score	gene symbol	RefSEq	chrom	txStart	txEnd	strand	integrant	chr	position	strand	integrant	chr	position	strand	integrant	chr	position	strand	integrant	chr	position	strand	integrant	chr	position	strand
207	4,25	DNMT3B	NM_006892	chr20	30813851	30860823	+	.	.	.	.	.	.	.	.	.	.	.	.	.	.	.	.	.	.	.	.
5309	1	SNTA1	NM_003098	chr20	31459423	31495359	-	.	.	.	.	.	.	.	.	.	.	.	.	.	.	.	.	.	.	.	.
134	5	E2F1	NM_005225	chr20	31726952	31737871	-	.	.	.	.	.	.	.	.	.	.	.	.	.	.	.	.	.	.	.	.
6304	0,5	EIF2S2	NM_003908	chr20	32139775	32163746	-	.	.	.	.	.	.	.	.	.	.	.	.	.	.	.	.	.	.	.	.
2932	1	AHCY	NM_000687	chr20	32331731	32354876	-	.	.	.	.	AY516801	chr20	32351787	+	.	.	.	.	.	.	.	.	.	.	.	.
4172	1	ITCH	NM_001257138	chr20	32414701	32562859	+	CL800833	chr20	32469846	-	.	.	.	.	.	.	.	.	.	.	.	.	.	.	.	.
4172	1	ITCH	NM_001257138	chr20	32414701	32562859	+	CL800464	chr20	32444424	+	.	.	.	.	.	.	.	.	.	.	.	.	.	.	.	.
7294	0,25	DYNLRB1	NM_014183	chr20	32567864	32592423	+	.	.	.	.	.	.	.	.	.	.	.	.	.	.	.	.	.	.	.	.
4803	1	PIGU	NM_080476	chr20	32612006	32728750	-	CL799801	chr20	32643818	+	.	.	.	.	.	.	.	.	.	.	.	.	.	.	.	.
4803	1	PIGU	NM_080476	chr20	32612006	32728750	-	BH609840	chr20	32722585	+	.	.	.	.	.	.	.	.	.	.	.	.	.	.	.	.
903	2,5	NCOA6	NM_001242539	chr20	32766238	32877094	-	.	.	.	.	.	.	.	.	.	.	.	.	.	.	.	.	.	.	.	.
3884	1	GGT7	NM_178026	chr20	32896183	32924322	-	.	.	.	.	.	.	.	.	.	.	.	.	.	.	.	.	.	.	.	.
2892	1	ACSS2	NM_018677	chr20	32927988	32979430	+	.	.	.	.	.	.	.	.	.	.	.	.	.	.	.	.	.	.	.	.
3978	1	GSS	NM_000178	chr20	32979896	33007262	-	.	.	.	.	.	.	.	.	.	.	.	.	.	.	.	.	.	.	.	.
6768	0,5	PROCR	NM_006404	chr20	33223434	33228826	+	.	.	.	.	.	.	.	.	.	.	.	.	.	.	.	.	.	.	.	.
6380	0,5	GDF5	NM_000557	chr20	33484562	33489441	-	.	.	.	.	.	.	.	.	.	.	.	.	.	.	.	.	.	.	.	.
3447	1	CPNE1	NM_152925	chr20	33677366	33716292	-	.	.	.	.	.	.	.	.	.	.	.	.	.	.	.	.	.	.	.	.
4576	1	NFS1	NR_037570	chr20	33720023	33750701	-	.	.	.	.	.	.	.	.	.	.	.	.	.	.	.	.	.	.	.	.
5077	1	RBM39	NM_001242599	chr20	33754944	33793672	-	.	.	.	.	.	.	.	.	.	.	.	.	.	.	.	.	.	.	.	.
2710	1,25	PHF20	NM_016436	chr20	33823336	34001702	+	CL800782	chr20	33883177	-	.	.	.	.	.	.	.	.	.	.	.	.	.	.	.	.
6311	0,5	EPB41L1	NM_177996	chr20	34144045	34284135	+	.	.	.	.	.	.	.	.	.	.	.	.	.	.	.	.	.	.	.	.
2434	1,5	TGIF2	NM_001199515	chr20	34636370	34655769	+	.	.	.	.	.	.	.	.	.	.	.	.	.	.	.	.	.	.	.	.
4518	1	NDRG3	NM_022477	chr20	34713582	34807955	-	.	.	.	.	.	.	.	.	.	.	.	.	.	.	.	.	.	.	.	.
3609	1	DSN1	NM_001145317	chr20	34813607	34835644	-	.	.	.	.	.	.	.	.	.	.	.	.	.	.	.	.	.	.	.	.
1958	1,75	RBL1	NM_183404	chr20	35065390	35157824	-	.	.	.	.	.	.	.	.	.	.	.	.	.	.	.	.	.	.	.	.
1729	2	RPN2	NM_001135771	chr20	35240869	35303439	+	.	.	.	.	.	.	.	.	.	.	.	.	.	.	.	.	.	.	.	.
2157	1,5	GHRH	NM_001184731	chr20	35312903	35318713	-	.	.	.	.	.	.	.	.	.	.	.	.	.	.	.	.	.	.	.	.
195	4,5	SRC	NM_005417	chr20	35406501	35467235	+	.	.	.	.	.	.	.	.	.	.	.	.	.	.	.	.	.	.	.	.
5940	0,75	NNAT	NM_181689	chr20	35583020	35585504	+	.	.	.	.	.	.	.	.	.	.	.	.	.	.	.	.	.	.	.	.
7090	0,5	VSTM2L	NM_080607	chr20	35964912	36007161	+	.	.	.	.	.	.	.	.	.	.	.	.	.	.	.	.	.	.	.	.
2435	1,5	TGM2	NM_198951	chr20	36199764	36227114	-	.	.	.	.	.	.	.	.	.	.	.	.	.	.	.	.	.	.	.	.
6917	0,5	SLC32A1	NM_080552	chr20	36786518	36791429	+	.	.	.	.	.	.	.	.	.	.	.	.	.	.	.	.	.	.	.	.
2337	1,5	PPP1R16B	NM_015568	chr20	36867761	36985081	+	.	.	.	.	.	.	.	.	.	.	.	.	.	.	.	.	.	.	.	.
6343	0,5	FAM83D	NM_030919	chr20	36988368	37015117	+	.	.	.	.	.	.	.	.	.	.	.	.	.	.	.	.	.	.	.	.
3580	1	DHX35	NM_001190809	chr20	37024394	37101780	+	.	.	.	.	.	.	.	.	.	.	.	.	.	.	.	.	.	.	.	.
612	3	MAFB	NM_005461	chr20	38747930	38751290	-	.	.	.	.	.	.	.	.	.	.	.	.	.	.	.	.	.	.	.	.
435	3,5	TOP1	NM_003286	chr20	39090875	39186540	+	AY517126.1	chr20	39098231	-	.	.	.	.	.	.	.	.	.	.	.	.	.	.	.	.
37	8	PLCG1	NM_002660	chr20	39199574	39237771	+	.	.	.	.	.	.	.	.	.	.	.	.	.	.	.	.	.	.	.	.
3348	1	CHD6	NM_032221	chr20	39464583	39680547	-	CL529204	chr20	39469720	+	.	.	.	.	.	.	.	.	.	.	.	.	.	.	.	.
749	2,75	PTPRT	NM_133170	chr20	40134805	41251971	-	.	.	.	.	.	.	.	.	.	.	.	.	.	.	.	.	434_104_1	chr20	40244924	+
5223	1	SGK2	NM_001199264	chr20	41627168	41647687	+	.	.	.	.	.	.	.	.	.	.	.	.	.	.	.	.	.	.	.	.
4468	1	MYBL2	NM_002466	chr20	41729122	41778536	+	.	.	.	.	.	.	.	.	.	.	.	.	.	.	.	.	.	.	.	.
6378	0,5	GDAP1L1	NM_024034	chr20	42309311	42342971	+	.	.	.	.	.	.	.	.	.	.	.	.	.	.	.	.	.	.	.	.
4063	1	HNF4A	NM_001030003	chr20	42417854	42494899	+	.	.	.	.	.	.	.	.	.	.	.	.	.	.	.	.	.	.	.	.
7536	0,25	SERINC3	NM_198941	chr20	42558277	42584140	-	.	.	.	.	.	.	.	.	.	.	.	.	.	.	.	.	.	.	.	.
4819	1	PKIG	NM_181805	chr20	42593849	42681092	+	.	.	.	.	.	.	.	.	.	.	.	.	.	.	.	.	.	.	.	.
353	3,5	ADA	NM_000022	chr20	42681576	42713790	-	.	.	.	.	.	.	.	.	.	.	.	.	.	.	.	.	.	.	.	.
1856	2	WISP2	NM_003881	chr20	42777298	42789866	+	.	.	.	.	.	.	.	.	.	.	.	.	.	.	.	.	.	.	.	.
5389	1	STK4	NM_006282	chr20	43028533	43142007	+	BH609848	chr20	43116128	-	.	.	.	.	.	.	.	.	.	.	.	.	.	.	.	.
5389	1	STK4	NM_006282	chr20	43028533	43142007	+	CL800296	chr20	43075045	+	.	.	.	.	.	.	.	.	.	.	.	.	.	.	.	.
5389	1	STK4	NM_006282	chr20	43028533	43142007	+	CL529633	chr20	43120289	+	.	.	.	.	.	.	.	.	.	.	.	.	.	.	.	.

Table S2

tumor associated genes								HIV				MLV				MMTV				MMTV(SIN)				MMTV(SIN)arrest			
rank	score	gene symbol	RefSEq	chrom	txStart	txEnd	strand	integrant	chr	position	strand	integrant	chr	position	strand	integrant	chr	position	strand	integrant	chr	position	strand	integrant	chr	position	strand
5389	1	STK4	NM_006282	chr20	43028533	43142007	+	CL800043	chr20	43093720	-	.	.	.	.	.	.	.	.	.	.	.	.	.	.	.	.
4204	1	KCNS1	NM_002251	chr20	43154363	43163167	-	.	.	.	.	.	.	.	.	.	.	.	.	.	.	.	.	.	.	.	.
7476	0,25	PI3	NM_002638	chr20	43236953	43238599	+	.	.	.	.	.	.	.	.	.	.	.	.	.	.	.	.	.	.	.	.
7534	0,25	SEMG1	NM_003007	chr20	43269051	43271828	+	.	.	.	.	.	.	.	.	.	.	.	.	.	.	.	.	.	.	.	.
7535	0,25	SEMG2	NM_003008	chr20	43283423	43286513	+	.	.	.	.	.	.	.	.	.	.	.	.	.	.	.	.	.	.	.	.
1762	2	SLPI	NM_003064	chr20	43314292	43316620	-	.	.	.	.	.	.	.	.	.	.	.	.	.	.	.	.	.	.	.	.
6861	0,5	SDC4	NM_002999	chr20	43387342	43410478	-	.	.	.	.	.	.	.	.	.	.	.	.	.	.	.	.	.	.	.	.
7042	0,5	TP53TG5	NM_014477	chr20	43435933	43440371	-	.	.	.	.	.	.	.	.	.	.	.	.	.	.	.	.	.	.	.	.
7268	0,25	DBNDD2	NM_001197140	chr20	43468234	43472664	+	.	.	.	.	.	.	.	.	.	.	.	.	.	.	.	.	.	.	.	.
4802	1	PIGT	NR_047693	chr20	43478120	43488299	+	.	.	.	.	.	.	.	.	.	.	.	.	.	.	.	.	.	.	.	.
2827	1,25	WFDC2	NM_006103	chr20	43531807	43543586	+	.	.	.	.	.	.	.	.	.	.	.	.	.	.	.	.	.	.	.	.
438	3,5	UBE2C	NM_181803	chr20	43874661	43879003	+	.	.	.	.	.	.	.	.	.	.	.	.	.	.	.	.	.	.	.	.
7039	0,5	TNNC2	NM_003279	chr20	43885261	43889360	-	.	.	.	.	.	.	.	.	.	.	.	.	.	.	.	.	.	.	.	.
2100	1,5	CTSA	NM_000308	chr20	43952997	43960865	+	.	.	.	.	.	.	.	.	.	.	.	.	.	.	.	.	.	.	.	.
400	3,5	MMP9	NM_004994	chr20	44070953	44078607	+	.	.	.	.	.	.	.	.	.	.	.	.	.	.	.	.	.	.	.	.
5253	1	SLC12A5	NM_001134771	chr20	44083735	44122196	+	.	.	.	.	.	.	.	.	.	.	.	.	.	.	.	.	.	.	.	.
1010	2,25	CD40	NM_152854	chr20	44180312	44191791	+	.	.	.	.	.	.	.	.	.	.	.	.	.	.	.	.	.	.	.	.
6163	0,5	CDH22	NM_021248	chr20	44235782	44313741	-	.	.	.	.	.	.	.	.	.	.	.	.	.	.	.	.	.	.	.	.
6899	0,5	SLC13A3	NM_001193340	chr20	44619868	44746531	-	.	.	.	.	.	.	.	.	.	.	.	.	.	.	.	.	.	.	.	.
5527	1	TP53RK	NM_033550	chr20	44746410	44751683	-	.	.	.	.	.	.	.	.	.	.	.	.	.	.	.	.	.	.	.	.
6914	0,5	SLC2A10	NM_030777	chr20	44771685	44798392	+	.	.	.	.	.	.	.	.	.	.	.	.	.	.	.	.	.	.	.	.
570	3	EYA2	NM_005244	chr20	44956669	45250899	+	AY517264.1	chr20	45030505	+	.	.	.	.	.	.	.	.	.	.	.	.	.	.	.	.
5755	1	ZMYND8	NM_012408	chr20	45271787	45418881	-	.	.	.	.	AY515860	chr20	45417709	-	.	.	.	.	.	.	.	.	.	.	.	.
626	3	NCOA3	NM_006534	chr20	45564007	45719028	+	CL800590	chr20	45639476	+	.	.	.	.	.	.	.	.	.	.	.	.	.	.	.	.
626	3	NCOA3	NM_006534	chr20	45564007	45719028	+	CL529540	chr20	45664615	+	.	.	.	.	.	.	.	.	.	.	.	.	.	.	.	.
5401	1	SULF2	NM_001161841	chr20	45719556	45848767	-	.	.	.	.	.	.	.	.	.	.	.	.	.	.	.	.	.	.	.	.
3021	1	ARFGEF2	NM_006420	chr20	46971681	47086637	+	.	.	.	.	.	.	.	.	.	.	.	.	.	.	.	.	.	.	.	.
3466	1	CSE1L	NM_001256135	chr20	47096189	47146904	+	CL529206	chr20	47125354	-	.	.	.	.	.	.	.	.	.	.	.	.	.	.	.	.
3466	1	CSE1L	NM_001256135	chr20	47096189	47146904	+	CL800151	chr20	47137912	-	.	.	.	.	.	.	.	.	.	.	.	.	.	.	.	.
870	2,5	KCNB1	NM_004975	chr20	47421911	47532588	-	.	.	.	.	.	.	.	.	.	.	.	.	.	.	.	.	.	.	.	.
4984	1	PTGIS	NM_000961	chr20	47553817	47618114	-	.	.	.	.	.	.	.	.	.	.	.	.	.	.	.	.	.	.	.	.
3121	1	B4GALT5	NM_004776	chr20	47682889	47763828	-	CL800769	chr20	47739392	-	.	.	.	.	.	.	.	.	.	.	.	.	.	.	.	.
2069	1,5	CEBPB	NM_005194	chr20	48240526	48242634	+	.	.	.	.	.	.	.	.	.	.	.	.	.	.	.	.	.	.	.	.
498	3,25	PTPN1	NM_002827	chr20	48560297	48634493	+	CL529207	chr20	48573623	+	.	.	.	.	.	.	.	.	.	.	.	.	.	.	.	.
6494	0,5	KCNG1	NM_002237	chr20	49053599	49073082	-	.	.	.	.	.	.	.	.	.	.	.	.	.	.	.	.	.	.	.	.
6666	0,5	NFATC2	NM_173091	chr20	49436900	49592665	-	.	.	.	.	.	.	.	.	.	.	.	.	.	.	.	.	.	.	.	.
1866	2	ZFP64	NM_199427	chr20	50133956	50241931	-	.	.	.	.	.	.	.	.	.	.	.	.	.	.	.	.	.	.	.	.
982	2,5	ZNF217	NM_006526	chr20	51617016	51633043	-	.	.	.	.	.	.	.	.	.	.	.	.	.	.	.	.	.	.	.	.
1275	2	BCAS1	NM_003657	chr20	51993485	52120711	-	.	.	.	.	.	.	.	.	.	.	.	.	.	.	.	.	.	.	.	.
1024	2,25	CYP24A1	NM_001128915	chr20	52203394	52223923	-	.	.	.	.	.	.	.	.	.	.	.	.	.	.	.	.	.	.	.	.
201	4,25	AURKA	NM_198434	chr20	54377851	54400758	-	.	.	.	.	.	.	.	.	.	.	.	.	.	.	.	.	.	.	.	.
7015	0,5	TFAP2C	NM_003222	chr20	54637764	54647745	+	.	.	.	.	.	.	.	.	.	.	.	.	.	.	.	.	.	.	.	.
6061	0,5	BMP7	NM_001719	chr20	55177215	55275114	-	.	.	.	.	.	.	.	.	.	.	.	.	.	.	.	.	.	.	.	.
1771	2	SPO11	NM_012444	chr20	55338237	55352456	+	.	.	.	.	.	.	.	.	.	.	.	.	.	.	.	.	.	.	.	.
2361	1,5	RAE1	NM_001015885	chr20	55360024	55386926	+	.	.	.	.	.	.	.	.	.	.	.	.	.	.	.	.	.	.	.	.
7260	0,25	CTCFL	NM_001269050	chr20	55515203	55533569	-	.	.	.	.	AY516174	chr20	55513697	+	.	.	.	.	.	.	.	.	.	.	.	.
4716	1	PCK1	NM_002591	chr20	55569542	55574919	+	.	.	.	.	.	.	.	.	.	.	.	.	.	.	.	.	.	.	.	.
4850	1	PMEPA1	NM_199170	chr20	55656853	55719076	-	.	.	.	.	.	.	.	.	.	.	.	.	.	.	.	.	.	.	.	.
5024	1	RAB22A	NM_020673	chr20	56318176	56375969	+	.	.	.	.	.	.	.	.	.	.	.	.	.	.	.	.	.	.	.	.
2999	1	APCDD1L	NM_153360	chr20	56467831	56523355	-	.	.	.	.	.	.	.	.	.	.	.	.	.	.	.	.	.	.	.	.
6680	0,5	NPEPL1	NM_001204872	chr20	56697592	56724307	+	.	.	.	.	.	.	.	.	.	.	.	.	.	.	.	.	.	.	.	.

Table S2

tumor associated genes								HIV				MLV				MMTV				MMTV(SIN)				MMTV(SIN)arrest			
rank	score	gene symbol	RefSEq	chrom	txStart	txEnd	strand	integrant	chr	position	strand	integrant	chr	position	strand	integrant	chr	position	strand	integrant	chr	position	strand	integrant	chr	position	strand
106	5,5	GNAS	NM_080425	chr20	56861430	56919645	+	.	.	.	.	.	.	.	.	.	.	.	.	.	.	.	.	.	.	.	.
5465	1	TH1L	NM_198976	chr20	56989657	57003583	+	.	.	.	.	.	.	.	.	.	.	.	.	.	.	.	.	.	.	.	.
6229	0,5	CTSZ	NM_001336	chr20	57003636	57015704	-	.	.	.	.	.	.	.	.	.	.	.	.	.	.	.	.	.	.	.	.
3074	1	ATP5E	NM_006886	chr20	57037127	57040817	-	.	.	.	.	.	.	.	.	.	.	.	.	.	.	.	.	.	.	.	.
7143	0,5	ZNF831	NM_178457	chr20	57199469	57267562	+	.	.	.	.	.	.	.	.	.	.	.	.	.	.	.	.	.	.	.	.
2320	1,5	PHACTR3	NM_183246	chr20	57729659	57856161	+	.	.	.	.	.	.	.	.	.	.	.	.	.	.	.	.	.	.	.	.
4918	1	PPP1R3D	NM_006242	chr20	57945281	57948747	-	.	.	.	.	.	.	.	.	.	.	.	.	.	.	.	.	.	.	.	.
805	2,5	CDH4	NM_001252339	chr20	59608211	59949068	+	.	.	.	.	.	.	.	.	.	.	.	.	.	.	.	.	.	.	.	.
1776	2	SS18L1	NM_198935	chr20	60152216	60190961	+	.	.	.	.	.	.	.	.	.	.	.	.	.	.	.	.	.	.	.	.
6451	0,5	HRH3	NM_007232	chr20	60223411	60228718	-	.	.	.	.	.	.	.	.	.	.	.	.	.	.	.	.	.	.	.	.
736	2,75	LAMA5	NM_005560	chr20	60317515	60375763	-	.	.	.	.	.	.	.	.	.	.	.	.	.	.	.	.	.	.	.	.
2599	1,25	GATA5	NM_080473	chr20	60471947	60484421	-	.	.	.	.	.	.	.	.	.	.	.	.	.	.	.	.	.	.	.	.
1587	2	NTSR1	NM_002531	chr20	60810633	60864568	+	.	.	.	.	.	.	.	.	.	.	.	.	.	.	.	.	.	.	.	.
2701	1,25	OGFR	NM_007346	chr20	60906621	60915797	+	.	.	.	.	.	.	.	.	.	.	.	.	.	.	.	.	.	.	.	.
1896	1,75	COL9A3	NM_001853	chr20	60918858	60942956	+	.	.	.	.	AY516821	chr20	60936510	-	.	.	.	.	.	.	.	.	.	.	.	.
7581	0,25	TCFL5	NM_006602	chr20	60942911	60963560	-	.	.	.	.	.	.	.	.	.	.	.	.	.	.	.	.	.	.	.	.
6422	0,5	HAR1A	NR_003244	chr20	61203088	61206182	+	.	.	.	.	.	.	.	.	.	.	.	.	.	.	.	.	.	.	.	.
7190	0,25	BIRC7	NM_022161	chr20	61337720	61342299	+	.	.	.	.	.	.	.	.	.	.	.	.	.	.	.	.	.	.	.	.
6200	0,5	COL20A1	NM_020882	chr20	61394982	61432729	+	.	.	.	.	.	.	.	.	.	.	.	.	.	.	.	.	.	.	.	.
6507	0,5	KCNQ2	NM_172109	chr20	61535475	61574437	-	.	.	.	.	.	.	.	.	.	.	.	.	.	.	.	.	.	.	.	.
1376	2	EEF1A2	NM_001958	chr20	61589808	61601112	-	.	.	.	.	.	.	.	.	.	.	.	.	.	.	.	.	.	.	.	.
4987	1	PTK6	NM_001256358	chr20	61630219	61639167	-	.	.	.	.	.	.	.	.	.	.	.	.	.	.	.	.	.	.	.	.
5351	1	SRMS	NM_080823	chr20	61642606	61649301	-	.	.	.	.	.	.	.	.	.	.	.	.	.	.	.	.	.	.	.	.
2448	1,5	TNFRSF6B	NM_003823	chr20	61798447	61800495	+	.	.	.	.	.	.	.	.	.	.	.	.	.	.	.	.	.	.	.	.
3022	1	ARFRP1	NM_001267544	chr20	61800438	61809809	-	.	.	.	.	.	.	.	.	.	.	.	.	.	.	.	.	.	.	.	.
5529	1	TPD52L2	NM_199360	chr20	61967024	61993342	+	CL799601	chr20	61977896	+	.	.	.	.	.	.	.	.	.	.	.	.	.	.	.	.
6957	0,5	SOX18	NM_018419	chr20	62149522	62151423	-	.	.	.	.	.	.	.	.	.	.	.	.	.	.	.	.	.	.	.	.
1942	1,75	MYT1	NM_004535	chr20	62266270	62344050	+	.	.	.	.	.	.	.	.	.	.	.	.	.	.	.	.	.	.	.	.
5536	1	TPTE	NM_199261	chr21	9928613	10012791	-	.	.	.	.	.	.	.	.	.	.	.	.	.	.	.	.	.	.	.	.
2509	1,25	BAGE	NM_001187	chr21	10079666	10120808	-	.	.	.	.	.	.	.	.	.	.	.	.	.	.	.	.	.	.	.	.
4882	1	POTED	NM_174981	chr21	13904368	13935777	+	.	.	.	.	.	.	.	.	.	.	.	.	.	.	.	.	.	.	.	.
5072	1	RBM11	NM_144770	chr21	14510336	14522564	+	.	.	.	.	.	.	.	.	.	.	.	.	.	.	.	.	.	.	.	.
6847	0,5	SAMSN1	NM_001256370	chr21	14779419	14877594	-	.	.	.	.	AY516622	chr21	14830690	+	.	.	.	.	.	.	.	.	.	.	.	.
4622	1	NRIP1	NM_003489	chr21	15255426	15358997	-	.	.	.	.	.	.	.	.	.	.	.	.	.	.	.	.	.	.	.	.
3491	1	CXADR	NM_001207066	chr21	17807094	17887768	+	.	.	.	.	.	.	.	.	.	.	.	.	.	.	.	.	.	.	.	.
7199	0,25	BTG3	NM_001130914	chr21	17887838	17907139	-	CL529386	chr21	17902691	-	.	.	.	.	.	.	.	.	.	.	.	.	.	.	.	.
3356	1	CHODL	NM_024944	chr21	18539020	18561558	+	.	.	.	.	.	.	.	.	.	.	.	.	.	.	.	.	.	.	.	.
3080	1	ATP5J	NM_001003703	chr21	26018661	26029836	-	.	.	.	.	.	.	.	.	.	.	.	.	.	.	.	.	.	.	.	.
7167	0,25	APP	NM_001204303	chr21	26174731	26465009	-	CL529208	chr21	26419253	+	.	.	.	.	.	.	.	.	.	.	.	.	.	.	.	.
7167	0,25	APP	NM_001204303	chr21	26174731	26465009	-	CL529500	chr21	26302418	-	.	.	.	.	.	.	.	.	.	.	.	.	.	.	.	.
1988	1,5	ADAMTS1	NM_006988	chr21	27130476	27139599	-	.	.	.	.	.	.	.	.	.	.	.	.	.	.	.	.	.	.	.	.
6146	0,5	CCT8	NM_006585	chr21	29350518	29367881	-	.	.	.	.	.	.	.	.	.	.	.	.	.	.	.	.	.	.	.	.
6097	0,5	C21orf7	NM_020152	chr21	29374743	29470073	+	.	.	.	.	.	.	.	.	.	.	.	.	.	.	.	.	.	.	.	.
3123	1	BACH1	NM_001186	chr21	29593090	29640340	+	.	.	.	.	.	.	.	.	.	.	.	.	.	.	.	.	.	.	.	.
1432	2	GRIK1	NM_175611	chr21	29831124	30234153	-	.	.	.	.	.	.	.	.	.	.	.	.	.	.	.	.	.	.	.	.
510	3,25	TIAM1	NM_003253	chr21	31412606	31853161	-	.	.	.	.	.	.	.	.	.	.	.	.	.	.	.	.	.	.	.	.
4101	1	HUNK	NM_014586	chr21	32167498	32298248	+	.	.	.	.	.	.	.	.	.	.	.	.	.	.	.	.	.	.	.	.
2426	1,5	SYNJ1	NM_203446	chr21	32922939	33022222	-	.	.	.	.	.	.	.	.	.	.	.	.	.	.	.	.	.	.	.	.
6096	0,5	C21orf62	NM_019596	chr21	33084854	33107923	-	.	.	.	.	.	.	.	.	.	.	.	.	.	.	.	.	.	.	.	.
632	3	OLIG2	NM_005806	chr21	33320085	33323373	+	.	.	.	.	.	.	.	.	.	.	.	.	.	.	.	.	.	.	.	.
743	2,75	OLIG1	NM_138983	chr21	33364319	33366598	+	.	.	.	.	.	.	.	.	.	.	.	.	.	.	.	.	.	.	.	.

Table S2

tumor associated genes								HIV				MLV				MMTV				MMTV(SIN)				MMTV(SIN)arrest			
rank	score	gene symbol	RefSEq	chrom	txStart	txEnd	strand	integrant	chr	position	strand	integrant	chr	position	strand	integrant	chr	position	strand	integrant	chr	position	strand	integrant	chr	position	strand
4119	1	IFNAR2	NM_000874	chr21	33524100	33556932	+	.	.	.	.	.	.	.	.	.	.	.	.	.	.	.	.	.	.	.	.
1465	2	IFNAR1	NM_000629	chr21	33619083	33653998	+	.	.	.	.	.	.	.	.	.	.	.	.	.	.	.	.	.	.	.	.
6467	0,5	IFNGR2	NM_005534	chr21	33697071	33731698	+	.	.	.	.	.	.	.	.	.	.	.	.	.	.	.	.	.	.	.	.
3858	1	GART	NM_001136006	chr21	33798107	33837068	-	.	.	.	.	.	.	.	.	.	.	.	.	.	.	.	.	.	.	.	.
6221	0,5	CRYZL1	NM_145858	chr21	33883517	33936030	-	.	.	.	.	.	.	.	.	.	.	.	.	.	.	.	.	.	.	.	.
3081	1	ATP5O	NM_001697	chr21	34197626	34210028	-	.	.	.	.	.	.	.	.	.	.	.	.	.	.	.	.	.	.	.	.
3388	1	CLIC6	NM_053277	chr21	34963557	35012389	+	.	.	.	.	.	.	.	.	.	.	.	.	.	.	.	.	.	.	.	.
69	6,5	RUNX1	NM_001001890	chr21	35081967	35182857	-	.	.	.	.	.	.	.	.	.	.	.	.	.	.	.	.	.	.	.	.
7226	0,25	CBR1	NM_001757	chr21	36364154	36367332	+	.	.	.	.	.	.	.	.	.	.	.	.	.	.	.	.	.	.	.	.
3347	1	CHAF1B	NM_005441	chr21	36679558	36710995	+	.	.	.	.	.	.	.	.	.	.	.	.	.	.	.	.	.	.	.	.
5243	1	SIM2	NM_005069	chr21	36993860	37044380	+	.	.	.	.	.	.	.	.	.	.	.	.	.	.	.	.	.	.	.	.
4055	1	HLCS	NM_001242784	chr21	37045058	37260826	-	.	.	.	.	AY516660	chr21	37205151	+	.	.	.	.	.	.	.	.	1688_160_1	chr21	37133387	-
2322	1,5	PIGP	NM_153682	chr21	37359533	37367328	-	.	.	.	.	.	.	.	.	.	.	.	.	.	.	.	.	.	.	.	.
7060	0,5	TTC3	NM_001001894	chr21	37367440	37497278	+	BH609853	chr21	37391403	-	.	.	.	.	.	.	.	.	.	.	.	.	.	.	.	.
3633	1	DYRK1A	NM_101395	chr21	37661728	37809549	+	BH609854	chr21	37713528	+	.	.	.	.	.	.	.	.	.	.	.	.	.	.	.	.
3633	1	DYRK1A	NM_101395	chr21	37661728	37809549	+	BH609849	chr21	37713528	+	.	.	.	.	.	.	.	.	.	.	.	.	.	.	.	.
6502	0,5	KCNJ6	NM_002240	chr21	37918655	38210611	-	.	.	.	.	.	.	.	.	.	.	.	.	.	.	.	.	.	.	.	.
254	4	ERG	NM_004449	chr21	38673819	38955574	-	.	.	.	.	.	.	.	.	.	.	.	.	.	.	.	.	.	.	.	.
325	3,75	ETS2	NM_001256295	chr21	39099100	39118748	+	.	.	.	.	.	.	.	.	.	.	.	.	.	.	.	.	.	.	.	.
7351	0,25	HMGN1	NM_004965	chr21	39636110	39642917	-	.	.	.	.	.	.	.	.	.	.	.	.	.	.	.	.	.	.	.	.
7211	0,25	C21orf88	NR_026542	chr21	39890944	39906619	-	.	.	.	.	.	.	.	.	.	.	.	.	.	.	.	.	.	.	.	.
3108	1	B3GALT5	NM_033170	chr21	39951123	39956685	+	.	.	.	.	.	.	.	.	.	.	.	.	.	.	.	.	.	.	.	.
2706	1,25	PCP4	NM_006198	chr21	40161216	40223192	+	.	.	.	.	.	.	.	.	_310_1_197	chr21	40171942	+	.	.	.	.	.	.	.	.
4465	1	MX2	NM_002463	chr21	41655819	41702739	+	.	.	.	.	AY516701	chr21	41685747	-	.	.	.	.	.	.	.	.	.	.	.	.
6636	0,5	MX1	NM_001144925	chr21	41714389	41753011	+	.	.	.	.	.	.	.	.	.	.	.	.	.	.	.	.	.	.	.	.
693	3	TMPRSS2	NM_001135099	chr21	41758347	41801862	-	.	.	.	.	.	.	.	.	.	.	.	.	.	.	.	.	.	.	.	.
2753	1,25	RIPK4	NM_020639	chr21	42032597	42060318	-	.	.	.	.	.	.	.	.	.	.	.	.	.	.	.	.	.	.	.	.
3225	1	C21orf128	NR_027243	chr21	42395312	42401713	-	.	.	.	.	.	.	.	.	.	.	.	.	.	.	.	.	.	.	.	.
2797	1,25	TFF1	NM_003225	chr21	42655459	42659713	-	.	.	.	.	.	.	.	.	.	.	.	.	.	.	.	.	.	.	.	.
5503	1	TMPRSS3	NR_046020	chr21	42665064	42690024	-	.	.	.	.	.	.	.	.	.	.	.	.	.	.	.	.	.	.	.	.
4740	1	PDE9A	NM_001001568	chr21	42946930	43068687	+	.	.	.	.	.	.	.	.	.	.	.	.	.	.	.	.	.	.	.	.
4554	1	NDUFV3	NM_001001503	chr21	43186446	43202842	+	.	.	.	.	.	.	.	.	.	.	.	.	.	.	.	.	.	.	.	.
4823	1	PKNOX1	NM_004571	chr21	43267711	43326757	+	.	.	.	.	AY515966	chr21	43268691	+	.	.	.	.	.	.	.	.	.	.	.	.
3284	1	CBS	NM_001178008	chr21	43346369	43369541	-	.	.	.	.	.	.	.	.	.	.	.	.	.	.	.	.	.	.	.	.
7071	0,5	U2AF1	NM_001025204	chr21	43386134	43400757	-	.	.	.	.	.	.	.	.	.	.	.	.	.	.	.	.	.	.	.	.
1757	2	SIK1	NM_173354	chr21	43658826	43671430	-	.	.	.	.	.	.	.	.	.	.	.	.	.	.	.	.	.	.	.	.
6833	0,5	RRP1B	NM_015056	chr21	43903859	43940388	+	.	.	.	.	.	.	.	.	.	.	.	.	.	.	.	.	.	.	.	.
2316	1,5	PDXK	NM_003681	chr21	43963405	44006616	+	.	.	.	.	.	.	.	.	.	.	.	.	.	.	.	.	.	.	.	.
2557	1,25	CSTB	NM_000100	chr21	44017973	44020684	-	.	.	.	.	.	.	.	.	.	.	.	.	.	.	.	.	.	.	.	.
2926	1	AGPAT3	NM_020132	chr21	44109543	44231903	+	.	.	.	.	.	.	.	.	.	.	.	.	.	.	.	.	.	.	.	.
4761	1	PFKL	NM_002626	chr21	44544357	44571684	+	.	.	.	.	.	.	.	.	.	.	.	.	.	.	.	.	.	.	.	.
7050	0,5	TRPM2	NR_038257	chr21	44597911	44687392	+	.	.	.	.	.	.	.	.	.	.	.	.	.	.	.	.	.	.	.	.
5602	1	UBE2G2	NM_003343	chr21	45012922	45046179	-	.	.	.	.	.	.	.	.	.	.	.	.	.	.	.	.	.	.	.	.
733	2,75	ITGB2	NM_001127491	chr21	45130296	45173181	-	.	.	.	.	.	.	.	.	.	.	.	.	.	.	.	.	.	.	.	.
1015	2,25	COL18A1	NM_130445	chr21	45649524	45758062	+	.	.	.	.	.	.	.	.	.	.	.	.	.	.	.	.	.	.	.	.
6202	0,5	COL6A1	NM_001848	chr21	46226090	46249391	+	.	.	.	.	.	.	.	.	.	.	.	.	.	.	.	.	.	.	.	.
3419	1	COL6A2	NM_058174	chr21	46342460	46374147	+	.	.	.	.	.	.	.	.	.	.	.	.	.	.	.	.	.	.	.	.
3809	1	FTCD	NM_006657	chr21	46380603	46399909	-	.	.	.	.	.	.	.	.	.	.	.	.	.	.	.	.	.	.	.	.
4313	1	LSS	NM_001145436	chr21	46433465	46473166	-	.	.	.	.	.	.	.	.	.	.	.	.	.	.	.	.	.	.	.	.
1528	2	MCM3AP	NM_003906	chr21	46479475	46529664	-	CL529484	chr21	46524888	-	.	.	.	.	.	.	.	.	.	.	.	.	.	.	.	.
2761	1,25	S100B	NM_006272	chr21	46842958	46849463	-	.	.	.	.	.	.	.	.	.	.	.	.	.	.	.	.	.	.	.	.

Table S2

tumor associated genes								HIV				MLV				MMTV				MMTV(SIN)				MMTV(SIN)arrest			
rank	score	gene symbol	RefSEq	chrom	txStart	txEnd	strand	integrant	chr	position	strand	integrant	chr	position	strand	integrant	chr	position	strand	integrant	chr	position	strand	integrant	chr	position	strand
1688	2	PRMT2	NM_206962	chr21	46879934	46909583	+	.	.	.	.	.	.	.	.	.	.	.	.	.	.	.	.	.	.	.	.
2070	1,5	CECR6	NM_001163079	chr22	15977188	15982257	-	.	.	.	.	.	.	.	.	.	.	.	.	.	.	.	.	.	.	.	.
2546	1,25	CECR5	NM_033070	chr22	15998409	16020169	-	.	.	.	.	.	.	.	.	.	.	.	.	.	.	.	.	.	.	.	.
3325	1	CECR2	NM_031413	chr22	16336627	16413845	+	.	.	.	.	.	.	.	.	.	.	.	.	.	.	.	.	.	.	.	.
2403	1,5	SLC25A18	NM_031481	chr22	16423182	16453647	+	.	.	.	.	.	.	.	.	.	.	.	.	.	.	.	.	.	.	.	.
790	2,5	ATP6V1E1	NM_001696	chr22	16454902	16491588	-	.	.	.	.	.	.	.	.	.	.	.	.	.	.	.	.	.	.	.	.
2024	1,5	BCL2L13	NM_001270729	chr22	16501349	16593621	+	.	.	.	.	.	.	.	.	.	.	.	.	.	.	.	.	.	.	.	.
1003	2,25	BID	NM_197967	chr22	16596905	16637261	-	.	.	.	.	.	.	.	.	.	.	.	.	.	.	.	.	.	.	.	.
4396	1	MICAL3	NM_001136004	chr22	16722920	16887325	-	.	.	.	.	.	.	.	.	.	.	.	.	.	.	.	.	2061_180_1	chr22	16687088	+
5588	1	TUBA8	NM_018943	chr22	16973452	16994498	+	.	.	.	.	.	.	.	.	.	.	.	.	.	.	.	.	.	.	.	.
4951	1	PRODH	NM_001195226	chr22	17280286	17303964	-	.	.	.	.	.	.	.	.	.	.	.	.	.	.	.	.	.	.	.	.
3567	1	DGCR2	NR_033674	chr22	17403794	17489967	-	CL799734	chr22	17483562	+	.	.	.	.	.	.	.	.	.	.	.	.	.	.	.	.
3566	1	DGCR14	NM_022719	chr22	17497791	17512190	-	.	.	.	.	.	.	.	.	.	.	.	.	.	.	.	.	.	.	.	.
5575	1	TSSK2	NM_053006	chr22	17498320	17500136	+	.	.	.	.	.	.	.	.	.	.	.	.	.	.	.	.	.	.	.	.
5262	1	SLC25A1	NM_001256534	chr22	17543087	17546018	-	.	.	.	.	.	.	.	.	.	.	.	.	.	.	.	.	.	.	.	.
546	3	CLTCL1	NM_001835	chr22	17546986	17659239	-	.	.	.	.	.	.	.	.	.	.	.	.	.	.	.	.	.	.	.	.
1448	2	HIRA	NM_003325	chr22	17698223	17799219	-	.	.	.	.	AY516740	chr22	17795908	-	.	.	.	.	.	.	.	.	3239_324_1	chr22	17723772	+
4439	1	MRPL40	NM_003776	chr22	17800035	17803596	+	.	.	.	.	.	.	.	.	.	.	.	.	.	.	.	.	.	.	.	.
5620	1	UFD1L	NM_001035247	chr22	17817463	17846738	-	.	.	.	.	.	.	.	.	.	.	.	.	.	.	.	.	.	.	.	.
2551	1,25	CLDN5	NM_003277	chr22	17890546	17892860	-	.	.	.	.	.	.	.	.	.	.	.	.	.	.	.	.	.	.	.	.
3936	1	GP1BB	NM_000407	chr22	18091065	18092297	+	.	.	.	.	.	.	.	.	.	.	.	.	.	.	.	.	.	.	.	.
5438	1	TBX1	NM_080646	chr22	18124225	18147068	+	.	.	.	.	.	.	.	.	.	.	.	.	.	.	.	.	.	.	.	.
3921	1	GNB1L	NM_053004	chr22	18155933	18222462	-	CL800680	chr22	18203745	-	.	.	.	.	.	.	.	.	.	.	.	.	.	.	.	.
3921	1	GNB1L	NM_053004	chr22	18155933	18222462	-	BH609857	chr22	18204694	-	.	.	.	.	.	.	.	.	.	.	.	.	.	.	.	.
3228	1	C22orf29	NM_024627	chr22	18213660	18222371	-	.	.	.	.	.	.	.	.	.	.	.	.	.	.	.	.	.	.	.	.
1829	2	TXNRD2	NM_006440	chr22	18243039	18309359	-	.	.	.	.	.	.	.	.	.	.	.	.	.	.	.	.	.	.	.	.
549	3	COMT	NM_001135161	chr22	18318459	18337498	+	.	.	.	.	.	.	.	.	.	.	.	.	.	.	.	.	.	.	.	.
3050	1	ARVCF	NM_001670	chr22	18337401	18384309	-	.	.	.	.	.	.	.	.	.	.	.	.	.	.	.	.	.	.	.	.
3227	1	C22orf25	NM_152906	chr22	18388630	18433447	+	.	.	.	.	.	.	.	.	.	.	.	.	.	.	.	.	.	.	.	.
3569	1	DGCR8	NM_001190326	chr22	18447754	18479400	+	BH609858	chr22	18466044	-	.	.	.	.	.	.	.	.	.	.	.	.	.	.	.	.
5560	1	TRMT2A	NM_182984	chr22	18479388	18484818	-	.	.	.	.	.	.	.	.	.	.	.	.	.	.	.	.	.	.	.	.
5058	1	RANBP1	NM_002882	chr22	18485023	18494704	+	.	.	.	.	.	.	.	.	.	.	.	.	.	.	.	.	.	.	.	.
1736	2	RTN4R	NM_023004	chr22	18608937	18635816	-	.	.	.	.	.	.	.	.	.	.	.	.	.	.	.	.	.	.	.	.
3568	1	DGCR6L	NM_033257	chr22	18681760	18687628	-	.	.	.	.	.	.	.	.	.	.	.	.	.	.	.	.	.	.	.	.
411	3,5	PI4KA	NM_058004	chr22	19391978	19543100	-	CL800733	chr22	19477502	+	.	.	.	.	.	.	.	.	.	.	.	.	.	.	.	.
1325	2	CRKL	NM_005207	chr22	19601713	19638037	+	.	.	.	.	.	.	.	.	.	.	.	.	.	.	.	.	1570_154_1	chr22	19636699	+
6002	0,5	AIFM3	NR_027464	chr22	19651446	19665649	+	.	.	.	.	.	.	.	.	.	.	.	.	.	.	.	.	.	.	.	.
4324	1	LZTR1	NM_006767	chr22	19666557	19683326	+	.	.	.	.	.	.	.	.	.	.	.	.	.	.	.	.	.	.	.	.
6928	0,5	SLC7A4	NM_004173	chr22	19713006	19716847	-	.	.	.	.	.	.	.	.	.	.	.	.	.	.	.	.	.	.	.	.
7345	0,25	HIC2	NM_015094	chr22	20101692	20135750	+	.	.	.	.	.	.	.	.	.	.	.	.	.	.	.	.	.	.	.	.
5605	1	UBE2L3	NM_003347	chr22	20251835	20308323	+	CL800825	chr22	20268086	-	.	.	.	.	.	.	.	.	.	.	.	.	.	.	.	.
5605	1	UBE2L3	NM_003347	chr22	20251835	20308323	+	CL529211	chr22	20303952	+	.	.	.	.	.	.	.	.	.	.	.	.	.	.	.	.
5605	1	UBE2L3	NM_003347	chr22	20251835	20308323	+	BH609860	chr22	20290212	-	.	.	.	.	.	.	.	.	.	.	.	.	.	.	.	.
7109	0,5	YPEL1	NM_013313	chr22	20381825	20420071	-	.	.	.	.	.	.	.	.	.	.	.	.	.	.	.	.	.	.	.	.
142	5	MAPK1	NM_138957	chr22	20453318	20551970	-	CL799808	chr22	20544760	-	.	.	.	.	.	.	.	.	.	.	.	.	.	.	.	.
142	5	MAPK1	NM_138957	chr22	20453318	20551970	-	BH609861	chr22	20530551	+	.	.	.	.	.	.	.	.	.	.	.	.	.	.	.	.
4899	1	PPM1F	NM_014634	chr22	20603791	20637250	-	.	.	.	.	.	.	.	.	.	.	.	.	.	.	.	.	.	.	.	.
7598	0,25	TOP3B	NM_003935	chr22	20641402	20667147	-	.	.	.	.	.	.	.	.	.	.	.	.	.	.	.	.	.	.	.	.
4932	1	PRAME	NM_206955	chr22	21220122	21231550	-	.	.	.	.	.	.	.	.	.	.	.	.	.	.	.	.	.	.	.	.
3885	1	GGTLC2	NM_199127	chr22	21318781	21320368	+	.	.	.	.	.	.	.	.	.	.	.	.	.	.	.	.	.	.	.	.
7523	0,25	RTDR1	NM_014433	chr22	21731592	21814241	-	.	.	.	.	.	.	.	.	.	.	.	.	.	.	.	.	.	.	.	.

Table S2

tumor associated genes								HIV				MLV				MMTV				MMTV(SIN)				MMTV(SIN)arrest			
rank	score	gene symbol	RefSEq	chrom	txStart	txEnd	strand	integrant	chr	position	strand	integrant	chr	position	strand	integrant	chr	position	strand	integrant	chr	position	strand	integrant	chr	position	strand
1914	1,75	GNAZ	NM_002073	chr22	21742668	21797221	+	.	.	.	.	.	.	.	.	.	.	.	.	.	.	.	.	.	.	.	.
5032	1	RAB36	NM_004914	chr22	21817512	21836531	+	.	.	.	.	.	.	.	.	.	.	.	.	.	.	.	.	.	.	.	.
170	4,5	BCR	NM_021574	chr22	21852551	21990224	+	.	.	.	.	.	.	.	.	.	.	.	.	.	.	.	.	.	.	.	.
4414	1	MMP11	NM_005940	chr22	22445035	22456503	+	.	.	.	.	.	.	.	.	.	.	.	.	.	.	.	.	.	.	.	.
146	5	SMARCB1	NM_001007468	chr22	22459149	22506705	+	.	.	.	.	.	.	.	.	.	.	.	.	.	.	.	.	.	.	.	.
3560	1	DERL3	NM_001002862	chr22	22506689	22511199	-	.	.	.	.	.	.	.	.	.	.	.	.	.	.	.	.	.	.	.	.
399	3,5	MIF	NM_002415	chr22	22566564	22567409	+	.	.	.	.	.	.	.	.	.	.	.	.	.	.	.	.	.	.	.	.
3983	1	GSTT2	NM_000854	chr22	22629600	22633393	-	.	.	.	.	.	.	.	.	.	.	.	.	.	.	.	.	.	.	.	.
328	3,75	GSTT1	NM_000853	chr22	22706138	22714284	-	.	.	.	.	.	.	.	.	.	.	.	.	.	.	.	.	.	.	.	.
3255	1	CABIN1	NM_012295	chr22	22737764	22904596	+	.	.	.	.	.	.	.	.	.	.	.	.	.	.	.	.	.	.	.	.
7327	0,25	GGT5	NM_001099781	chr22	22945621	22971110	-	.	.	.	.	.	.	.	.	.	.	.	.	.	.	.	.	.	.	.	.
5643	1	UPB1	NM_016327	chr22	23221250	23252553	+	.	.	.	.	.	.	.	.	.	.	.	.	.	.	.	.	.	.	.	.
1055	2,25	GGT1	NM_001032364	chr22	23329123	23354972	+	.	.	.	.	.	.	.	.	.	.	.	.	.	.	.	.	.	.	.	.
2916	1	ADRBK2	NM_005160	chr22	24290860	24455258	+	.	.	.	.	.	.	.	.	.	.	.	.	.	.	.	.	.	.	.	.
485	3,25	MYO18B	NM_032608	chr22	24468119	24757007	+	.	.	.	.	.	.	.	.	.	.	.	.	.	.	.	.	.	.	.	.
2767	1,25	SEZ6L	NM_001184774	chr22	24895439	25109563	+	.	.	.	.	.	.	.	.	.	.	.	.	.	.	.	.	.	.	.	.
897	2,5	MN1	NM_002430	chr22	26474264	26527486	-	.	.	.	.	AY516068	chr22	26518512	-	.	.	.	.	.	.	.	.	.	.	.	.
7059	0,5	TTC28	NM_001145418	chr22	26704001	27405853	-	.	.	.	.	.	.	.	.	.	.	.	.	.	.	.	.	.	.	.	.
76	6,25	CHEK2	NM_007194	chr22	27413730	27467822	-	.	.	.	.	AY516248	chr22	27438106	-	.	.	.	.	.	.	.	.	.	.	.	.
2833	1,25	XBP1	NM_005080	chr22	27520547	27526560	-	.	.	.	.	.	.	.	.	.	.	.	.	.	.	.	.	.	.	.	.
156	4,75	EWSR1	NM_013986	chr22	27993997	28026515	+	.	.	.	.	.	.	.	.	.	.	.	.	.	.	.	.	.	.	.	.
2364	1,5	RASL10A	NM_006477	chr22	28038921	28041745	-	.	.	.	.	.	.	.	.	.	.	.	.	.	.	.	.	.	.	.	.
6810	0,5	RFPL1	NM_021026	chr22	28164571	28168444	+	.	.	.	.	.	.	.	.	.	.	.	.	.	.	.	.	.	.	.	.
6660	0,5	NEFH	NM_021076	chr22	28206180	28217277	+	.	.	.	.	.	.	.	.	.	.	.	.	.	.	.	.	.	.	.	.
5467	1	THOC5	NM_001002877	chr22	28234155	28279736	-	.	.	.	.	.	.	.	.	.	.	.	.	.	.	.	.	.	.	.	.
49	7,25	NF2	NM_016418	chr22	28329544	28424589	+	.	.	.	.	.	.	.	.	.	.	.	.	.	.	.	.	.	.	.	.
7439	0,25	MTMR3	NM_153050	chr22	28609157	28756857	+	BH609863	chr22	28678909	+	.	.	.	.	.	.	.	.	.	.	.	.	.	.	.	.
1503	2	LIF	NM_001257135	chr22	28966435	28972840	-	.	.	.	.	.	.	.	.	.	.	.	.	.	.	.	.	.	.	.	.
2300	1,5	OSM	NM_020530	chr22	28988818	28992829	-	.	.	.	.	.	.	.	.	.	.	.	.	.	.	.	.	.	.	.	.
3836	1	GAL3ST1	NM_004861	chr22	29280623	29290876	-	.	.	.	.	.	.	.	.	.	.	.	.	.	.	.	.	.	.	.	.
3622	1	DUSP18	NM_152511	chr22	29388038	29393872	-	.	.	.	.	.	.	.	.	.	.	.	.	.	.	.	.	.	.	.	.
6622	0,5	MORC2	NM_014941	chr22	29652599	29694187	-	.	.	.	.	.	.	.	.	.	.	.	.	.	.	.	.	.	.	.	.
5299	1	SMTN	NM_134270	chr22	29807281	29830610	+	.	.	.	.	.	.	.	.	.	.	.	.	.	.	.	.	.	.	.	.
604	3	INPP5J	NM_001002837	chr22	29848960	29860683	+	.	.	.	.	.	.	.	.	.	.	.	.	.	.	.	.	.	.	.	.
4832	1	PLA2G3	NM_015715	chr22	29860792	29866469	-	.	.	.	.	.	.	.	.	.	.	.	.	.	.	.	.	.	.	.	.
4284	1	LIMK2	NM_001031801	chr22	29974347	30003624	+	.	.	.	.	.	.	.	.	.	.	.	.	.	.	.	.	.	.	.	.
4808	1	PIK3IP1	NM_052880	chr22	30007578	30018520	-	.	.	.	.	.	.	.	.	.	.	.	.	.	.	.	.	.	.	.	.
1596	2	PATZ1	NM_014323	chr22	30051789	30072249	-	.	.	.	.	.	.	.	.	.	.	.	.	.	.	.	.	.	.	.	.
4815	1	PISD	NM_014338	chr22	30344478	30356810	-	.	.	.	.	.	.	.	.	.	.	.	.	.	.	.	.	.	.	.	.
7274	0,25	DEPDC5	NM_001136029	chr22	30480847	30633020	+	CL799677	chr22	30622795	-	.	.	.	.	.	.	.	.	.	.	.	.	.	.	.	.
7212	0,25	C22orf24	NM_015372	chr22	30659506	30671336	-	.	.	.	.	.	.	.	.	.	.	.	.	.	.	.	.	.	.	.	.
1186	2,25	YWHAH	NM_003405	chr22	30670478	30683590	+	.	.	.	.	.	.	.	.	.	.	.	.	.	.	.	.	.	.	.	.
7558	0,25	SLC5A1	NM_001256314	chr22	30785156	30839011	+	.	.	.	.	.	.	.	.	.	.	.	.	.	.	.	.	.	.	.	.
5105	1	RFPL2	NM_001159546	chr22	30916421	30928537	-	.	.	.	.	.	.	.	.	.	.	.	.	.	.	.	.	.	.	.	.
5106	1	RFPL3	NM_001098535	chr22	31083853	31087148	+	.	.	.	.	.	.	.	.	.	.	.	.	.	.	.	.	.	.	.	.
3760	1	FBXO7	NM_001033024	chr22	31201223	31224818	+	.	.	.	.	.	.	.	.	.	.	.	.	.	.	.	.	.	.	.	.
1810	2	TIMP3	NM_000362	chr22	31526801	31589028	+	.	.	.	.	.	.	.	.	.	.	.	.	.	.	.	.	.	.	.	.
2188	1,5	HMOX1	NM_002133	chr22	34107059	34120207	+	.	.	.	.	.	.	.	.	.	.	.	.	.	.	.	.	.	.	.	.
5067	1	RASD2	NM_014310	chr22	34267297	34279991	+	.	.	.	.	.	.	.	.	.	.	.	.	.	.	.	.	.	.	.	.
185	4,5	MYH9	NM_002473	chr22	35007268	35114009	-	.	.	.	.	.	.	.	.	.	.	.	.	.	.	.	.	.	.	.	.
6651	0,5	NCF4	NM_000631	chr22	35586975	35604005	+	.	.	.	.	.	.	.	.	.	.	.	.	.	.	.	.	.	.	.	.

Table S2

tumor associated genes								HIV				MLV				MMTV				MMTV(SIN)				MMTV(SIN)arrest			
rank	score	gene symbol	RefSEq	chrom	txStart	txEnd	strand	integrant	chr	position	strand	integrant	chr	position	strand	integrant	chr	position	strand	integrant	chr	position	strand	integrant	chr	position	strand
2095	1,5	CSF2RB	NM_000395	chr22	35639620	35666425	+	.	.	.	.	.	.	.	.	.	.	.	.	.	.	.	.	.	.	.	.
4433	1	MPST	NM_001013436	chr22	35745628	35755809	+	.	.	.	.	.	.	.	.	.	.	.	.	.	.	.	.	.	.	.	.
5504	1	TMPRSS6	NM_153609	chr22	35791424	35829639	-	.	.	.	.	.	.	.	.	.	.	.	.	.	.	.	.	.	.	.	.
6473	0,5	IL2RB	NM_000878	chr22	35851825	35875908	-	.	.	.	.	.	.	.	.	.	.	.	.	.	.	.	.	.	.	.	.
941	2,5	RAC2	NM_002872	chr22	35951255	35970251	-	.	.	.	.	.	.	.	.	.	.	.	.	.	.	.	.	.	.	.	.
6244	0,5	CYTH4	NM_013385	chr22	36008440	36041335	+	.	.	.	.	AY516584	chr22	36030748	+	.	.	.	.	.	.	.	.	.	.	.	.
4388	1	MFNG	NM_001166343	chr22	36195046	36212371	-	.	.	.	.	.	.	.	.	.	.	.	.	.	.	.	.	.	.	.	.
3880	1	GGA1	NM_001001561	chr22	36334426	36344073	+	.	.	.	.	.	.	.	.	.	.	.	.	.	.	.	.	.	.	.	.
5231	1	SH3BP1	NM_018957	chr22	36365629	36381996	+	.	.	.	.	.	.	.	.	.	.	.	.	.	.	.	.	.	.	.	.
4747	1	PDXP	NM_020315	chr22	36384682	36392885	+	.	.	.	.	.	.	.	.	.	.	.	.	.	.	.	.	.	.	.	.
1501	2	LGALS1	NM_002305	chr22	36401558	36405755	+	.	.	.	.	.	.	.	.	.	.	.	.	.	.	.	.	.	.	.	.
5559	1	TRIOBP	NM_007032	chr22	36472186	36502509	+	CL800565	chr22	36446965	-	.	.	.	.	.	.	.	.	.	.	.	.	.	.	.	.
3866	1	GCAT	NM_001171690	chr22	36533857	36543129	+	.	.	.	.	.	.	.	.	.	.	.	.	.	.	.	.	.	.	.	.
924	2,5	POLR2F	NM_021974	chr22	36679619	36695265	+	.	.	.	.	.	.	.	.	.	.	.	.	.	.	.	.	.	.	.	.
5966	0,75	SOX10	NM_006941	chr22	36698264	36710485	-	.	.	.	.	.	.	.	.	.	.	.	.	.	.	.	.	.	.	.	.
6904	0,5	SLC16A8	NM_013356	chr22	36804089	36809116	-	.	.	.	.	.	.	.	.	.	.	.	.	.	.	.	.	.	.	.	.
1134	2,25	PLA2G6	NM_001199562	chr22	36837447	36907782	-	.	.	.	.	.	.	.	.	.	.	.	.	.	.	.	.	.	.	.	.
1329	2	CSNK1E	NM_152221	chr22	37016642	37043359	-	.	.	.	.	.	.	.	.	.	.	.	.	.	.	.	.	.	.	.	.
3546	1	DDX17	NM_006386	chr22	37209388	37232291	-	.	.	.	.	.	.	.	.	.	.	.	.	.	.	.	.	.	.	.	.
3590	1	DMC1	NM_007068	chr22	37244899	37296135	-	CL529645	chr22	37249168	-	.	.	.	.	.	.	.	.	.	.	.	.	.	.	.	.
6682	0,5	NPTXR	NM_014293	chr22	37544401	37569963	-	.	.	.	.	.	.	.	.	.	.	.	.	.	.	.	.	.	.	.	.
2501	1,25	APOBEC3A	NM_001270406	chr22	37683472	37689134	+	.	.	.	.	.	.	.	.	.	.	.	.	.	.	.	.	.	.	.	.
3006	1	APOBEC3B	NM_004900	chr22	37708349	37718730	+	.	.	.	.	AY516683	chr22	37709488	-	.	.	.	.	.	.	.	.	.	.	.	.
3007	1	APOBEC3C	NM_014508	chr22	37740210	37744771	+	.	.	.	.	.	.	.	.	.	.	.	.	.	.	.	.	.	.	.	.
3008	1	APOBEC3F	NM_145298	chr22	37766618	37781921	+	.	.	.	.	.	.	.	.	.	.	.	.	.	.	.	.	.	.	.	.
3009	1	APOBEC3G	NM_021822	chr22	37802955	37813694	+	.	.	.	.	.	.	.	.	.	.	.	.	.	.	.	.	.	.	.	.
3286	1	CBX7	NM_175709	chr22	37856724	37878484	-	.	.	.	.	.	.	.	.	.	.	.	.	.	.	.	.	.	.	.	.
91	6	PDGFB	NM_002608	chr22	37949630	37970903	-	.	.	.	.	.	.	.	.	.	.	.	.	.	.	.	.	.	.	.	.
4392	1	MGAT3	NM_001098270	chr22	38213174	38218145	+	.	.	.	.	.	.	.	.	.	.	.	.	.	.	.	.	.	.	.	.
2044	1,5	CACNA1I	NM_001003406	chr22	38296703	38415687	+	.	.	.	.	.	.	.	.	.	.	.	.	.	.	.	.	.	.	.	.
2174	1,5	GRAP2	NM_004810	chr22	38627031	38697330	+	AY517120.1	chr22	38633230	-	.	.	.	.	.	.	.	.	.	.	.	.	.	.	.	.
2174	1,5	GRAP2	NM_004810	chr22	38627031	38697330	+	CL529522	chr22	38672758	+	.	.	.	.	.	.	.	.	.	.	.	.	.	.	.	.
2492	1,25	ADSL	NM_000026	chr22	39072449	39092521	+	.	.	.	.	.	.	.	.	.	.	.	.	.	.	.	.	.	.	.	.
7544	0,25	SGSM3	NM_015705	chr22	39096540	39136239	+	CL800440	chr22	39100958	-	.	.	.	.	.	.	.	.	.	.	.	.	.	.	.	.
1111	2,25	MKL1	NM_020831	chr22	39136237	39362636	-	CL799746	chr22	39194704	+	AY516429	chr22	39260912	+	.	.	.	.	.	.	.	.	.	.	.	.
1111	2,25	MKL1	NM_020831	chr22	39136237	39362636	-	CL800449	chr22	39311675	+	AY516429	chr22	39260912	+	.	.	.	.	.	.	.	.	.	.	.	.
14	9,75	EP300	NM_001429	chr22	39818559	39906027	+	CL529213	chr22	39849131	-	.	.	.	.	.	.	.	.	.	.	.	.	.	.	.	.
14	9,75	EP300	NM_001429	chr22	39818559	39906027	+	AY516950.1	chr22	39823641	+	.	.	.	.	.	.	.	.	.	.	.	.	.	.	.	.
14	9,75	EP300	NM_001429	chr22	39818559	39906027	+	CL529557	chr22	39887599	+	.	.	.	.	.	.	.	.	.	.	.	.	.	.	.	.
5060	1	RANGAP1	NM_002883	chr22	39971560	40012162	-	.	.	.	.	.	.	.	.	.	.	.	.	.	.	.	.	.	.	.	.
1201	2	ACO2	NM_001098	chr22	40195074	40254939	+	.	.	.	.	.	.	.	.	.	.	.	.	.	.	.	.	.	.	.	.
1660	2	POLR3H	NM_001018052	chr22	40251751	40270425	-	.	.	.	.	.	.	.	.	.	.	.	.	.	.	.	.	.	.	.	.
4851	1	PMM1	NM_002676	chr22	40302835	40315817	-	.	.	.	.	.	.	.	.	.	.	.	.	.	.	.	.	.	.	.	.
768	2,75	XRCC6	NM_001469	chr22	40347240	40389998	+	.	.	.	.	.	.	.	.	.	.	.	.	.	.	.	.	.	.	.	.
5349	1	SREBF2	NM_004599	chr22	40559051	40632321	+	.	.	.	.	.	.	.	.	.	.	.	.	.	.	.	.	.	.	.	.
3334	1	CENPM	NM_024053	chr22	40664686	40673094	-	.	.	.	.	.	.	.	.	.	.	.	.	.	.	.	.	.	.	.	.
4489	1	NAGA	NM_000262	chr22	40784283	40796792	-	.	.	.	.	.	.	.	.	.	.	.	.	.	.	.	.	.	.	.	.
4529	1	NDUFA6	NM_002490	chr22	40811475	40816834	-	.	.	.	.	.	.	.	.	.	.	.	.	.	.	.	.	.	.	.	.
4862	1	POLDIP3	NM_032311	chr22	41309670	41340906	-	.	.	.	.	.	.	.	.	.	.	.	.	.	.	.	.	.	.	.	.
1334	2	CYB5R3	NM_001171660	chr22	41343789	41370584	-	.	.	.	.	.	.	.	.	.	.	.	.	.	.	.	.	.	.	.	.
1189	2	A4GALT	NM_017436	chr22	41418070	41446820	-	.	.	.	.	.	.	.	.	.	.	.	.	.	.	.	.	.	.	.	.

Table S2

tumor associated genes								HIV				MLV				MMTV				MMTV(SIN)				MMTV(SIN)arrest			
rank	score	gene symbol	RefSEq	chrom	txStart	txEnd	strand	integrant	chr	position	strand	integrant	chr	position	strand	integrant	chr	position	strand	integrant	chr	position	strand	integrant	chr	position	strand
3020	1	ARFGAP3	NM_014570	chr22	41522475	41583352	-	CL529434	chr22	41561706	-	.	.	.	.	.	.	.	.	.	.	.	.	.	.	.	.
4684	1	PACSIN2	NM_001184970	chr22	41595715	41741128	-	.	.	.	.	.	.	.	.	.	.	.	.	.	.	.	.	.	.	.	.
5584	1	TTLL1	NM_012263	chr22	41765466	41815378	-	.	.	.	.	.	.	.	.	.	.	.	.	.	.	.	.	.	.	.	.
1004	2,25	BIK	NM_001197	chr22	41836697	41855662	+	.	.	.	.	.	.	.	.	.	.	.	.	.	.	.	.	.	.	.	.
1527	2	MCAT	NM_173467	chr22	41858155	41869347	-	.	.	.	.	.	.	.	.	.	.	.	.	.	.	.	.	.	.	.	.
5573	1	TSPO	NM_001256530	chr22	41877884	41889192	+	.	.	.	.	.	.	.	.	.	.	.	.	.	.	.	.	.	.	.	.
5585	1	TTLL12	NM_015140	chr22	41892571	41913081	-	.	.	.	.	.	.	.	.	.	.	.	.	.	.	.	.	.	.	.	.
6625	0,5	MPPED1	NM_001044370	chr22	42137963	42234129	+	.	.	.	.	.	.	.	.	.	.	.	.	.	.	.	.	.	.	.	.
1788	2	SULT4A1	NM_014351	chr22	42551719	42589711	-	.	.	.	.	.	.	.	.	.	.	.	.	.	.	.	.	.	.	.	.
1640	2	PNPLA3	NM_025225	chr22	42650951	42674781	+	.	.	.	.	.	.	.	.	.	.	.	.	.	.	.	.	.	.	.	.
5178	1	SAMM50	NM_015380	chr22	42682593	42723745	+	.	.	.	.	.	.	.	.	.	.	.	.	.	.	.	.	.	.	.	.
4702	1	PARVB	NM_013327	chr22	42751489	42896445	+	CL800457	chr22	42763137	-	.	.	.	.	.	.	.	.	.	.	.	.	.	.	.	.
4702	1	PARVB	NM_013327	chr22	42751489	42896445	+	CL800564	chr22	42727190	-	.	.	.	.	.	.	.	.	.	.	.	.	.	.	.	.
6527	0,5	KIAA1644	NM_001099294	chr22	42970889	43040064	-	.	.	.	.	.	.	.	.	.	.	.	.	.	.	.	.	.	.	.	.
4274	1	LDOC1L	NM_032287	chr22	43267113	43272669	-	.	.	.	.	.	.	.	.	.	.	.	.	.	.	.	.	.	.	.	.
4959	1	PRR5	NM_001017530	chr22	43451351	43512225	+	.	.	.	.	.	.	.	.	.	.	.	.	.	.	.	.	.	.	.	.
4782	1	PHF21B	NM_001135862	chr22	43655706	43784473	-	.	.	.	.	.	.	.	.	.	.	.	.	.	.	.	.	.	.	.	.
2296	1,5	NUP50	NM_153645	chr22	43939215	43962554	+	.	.	.	.	.	.	.	.	.	.	.	.	.	.	.	.	.	.	.	.
3717	1	FAM118A	NM_001104595	chr22	44083744	44116500	+	.	.	.	.	.	.	.	.	.	.	.	.	.	.	.	.	.	.	.	.
5296	1	SMC1B	NM_148674	chr22	44118608	44188164	-	.	.	.	.	.	.	.	.	.	.	.	.	.	.	.	.	.	.	.	.
373	3,5	FBLN1	NM_006485	chr22	44277382	44340245	+	.	.	.	.	.	.	.	.	.	.	.	.	.	.	.	.	.	.	.	.
3101	1	ATXN10	NM_001167621	chr22	44446341	44619851	+	CL529444	chr22	44467065	+	.	.	.	.	.	.	.	.	.	.	.	.	.	.	.	.
3101	1	ATXN10	NM_001167621	chr22	44446341	44619851	+	CL799970	chr22	44554880	-	.	.	.	.	.	.	.	.	.	.	.	.	.	.	.	.
2831	1,25	WNT7B	NM_058238	chr22	44694911	44751672	-	.	.	.	.	.	.	.	.	.	.	.	.	.	.	.	.	.	.	.	.
340	3,75	PPARA	NM_005036	chr22	44925162	45018317	+	.	.	.	.	.	.	.	.	.	.	.	.	.	.	.	.	.	.	.	.
5581	1	TTC38	NM_017931	chr22	45042524	45068569	+	.	.	.	.	.	.	.	.	.	.	.	.	.	.	.	.	.	.	.	.
3993	1	GTSE1	NM_016426	chr22	45071301	45105371	+	.	.	.	.	.	.	.	.	.	.	.	.	.	.	.	.	.	.	.	.
5562	1	TRMU	NM_018006	chr22	45109961	45131901	+	.	.	.	.	.	.	.	.	.	.	.	.	.	.	.	.	.	.	.	.
3963	1	GRAMD4	NM_015124	chr22	45401321	45454352	+	.	.	.	.	.	.	.	.	.	.	.	.	.	.	.	.	.	.	.	.
132	5	CERK	NM_022766	chr22	45458970	45512816	-	.	.	.	.	.	.	.	.	.	.	.	.	.	.	.	.	.	.	.	.
5435	1	TBC1D22A	NM_014346	chr22	45537181	45950006	+	.	.	.	.	.	.	.	.	.	.	.	.	.	.	.	.	.	.	.	.
1393	2	FAM19A5	NM_001082967	chr22	47263951	47533750	+	.	.	.	.	.	.	.	.	.	.	.	.	s716_133	chr22	47456715	+	.	.	.	.
3159	1	BRD1	NM_014577	chr22	48552940	48604456	-	.	.	.	.	.	.	.	.	.	.	.	.	.	.	.	.	.	.	.	.
5729	1	ZBED4	NM_014838	chr22	48633500	48669730	+	CL800819	chr22	48648123	-	.	.	.	.	.	.	.	.	.	.	.	.	.	.	.	.
1240	2	ALG12	NM_024105	chr22	48682856	48698110	-	.	.	.	.	.	.	.	.	.	.	.	.	.	.	.	.	.	.	.	.
3457	1	CRELD2	NM_001135101	chr22	48698286	48707190	+	.	.	.	.	.	.	.	.	.	.	.	.	.	.	.	.	.	.	.	.
4812	1	PIM3	NM_001001852	chr22	48740146	48743724	+	.	.	.	.	.	.	.	.	.	.	.	.	.	.	.	.	.	.	.	.
2258	1,5	MLC1	NM_015166	chr22	48839946	48866485	-	.	.	.	.	.	.	.	.	.	.	.	.	.	.	.	.	.	.	.	.
1543	2	MOV10L1	NM_001164106	chr22	48927610	48942243	+	CL800525	chr22	48903011	-	.	.	.	.	.	.	.	.	.	.	.	.	.	.	.	.
5538	1	TRABD	NM_025204	chr22	48966486	48980154	+	BH609868	chr22	48968870	+	.	.	.	.	.	.	.	.	.	.	.	.	.	.	.	.
5591	1	TUBGCP6	NM_020461	chr22	48998244	49025527	-	.	.	.	.	.	.	.	.	.	.	.	.	.	.	.	.	.	.	.	.
1442	2	HDAC10	NM_001159286	chr22	49025739	49031961	-	.	.	.	.	.	.	.	.	.	.	.	.	.	.	.	.	.	.	.	.
1102	2,25	MAPK12	NM_002969	chr22	49033457	49042216	-	.	.	.	.	.	.	.	.	.	.	.	.	.	.	.	.	.	.	.	.
1101	2,25	MAPK11	NM_002751	chr22	49044268	49050906	-	.	.	.	.	.	.	.	.	.	.	.	.	.	.	.	.	.	.	.	.
4849	1	PLXNB2	NM_012401	chr22	49055534	49075336	-	.	.	.	.	.	.	.	.	.	.	.	.	.	.	.	.	.	.	.	.
5186	1	SBF1	NM_002972	chr22	49230296	49260330	-	.	.	.	.	.	.	.	.	.	.	.	.	.	.	.	.	.	.	.	.
2913	1	ADM2	NM_001253845	chr22	49267018	49271732	+	.	.	.	.	.	.	.	.	.	.	.	.	.	.	.	.	.	.	.	.
4402	1	MIOX	NM_017584	chr22	49272078	49275616	+	.	.	.	.	.	.	.	.	.	.	.	.	.	.	.	.	.	.	.	.
4290	1	LMF2	NM_033200	chr22	49288241	49293001	-	.	.	.	.	.	.	.	.	.	.	.	.	.	.	.	.	.	.	.	.
4504	1	NCAPH2	NM_001185011	chr22	49293510	49310075	+	CL799953	chr22	49296499	-	.	.	.	.	.	.	.	.	.	.	.	.	.	.	.	.
5200	1	SCO2	NM_005138	chr22	49308862	49310900	-	.	.	.	.	.	.	.	.	.	.	.	.	.	.	.	.	.	.	.	.

Table S2

tumor associated genes								HIV				MLV				MMTV				MMTV(SIN)				MMTV(SIN)arrest			
rank	score	gene symbol	RefSEq	chrom	txStart	txEnd	strand	integrant	chr	position	strand	integrant	chr	position	strand	integrant	chr	position	strand	integrant	chr	position	strand	integrant	chr	position	strand
766	2,75	TYMP	NM_001953	chr22	49311047	49315380	-	.	.	.	.	.	.	.	.	.	.	.	.	.	.	.	.	.	.	.	.
551	3	CPT1B	NM_001145137	chr22	49354155	49363372	-	.	.	.	.	.	.	.	.	.	.	.	.	.	.	.	.	.	.	.	.
1310	2	CHKB	NM_005198	chr22	49364252	49368294	-	.	.	.	.	.	.	.	.	.	.	.	.	.	.	.	.	.	.	.	.
276	4	MAPK8IP2	NM_012324	chr22	49385996	49396845	+	.	.	.	.	.	.	.	.	.	.	.	.	.	.	.	.	.	.	.	.
2503	1,25	ARSA	NM_000487	chr22	49408047	49413467	-	.	.	.	.	.	.	.	.	.	.	.	.	.	.	.	.	.	.	.	.
1712	2	RABL2B	NM_007081	chr22	49552785	49568953	-	.	.	.	.	.	.	.	.	.	.	.	.	.	.	.	.	.	.	.	.
2722	1,25	PPP2R3B	NM_013239	chrX	214667	267690	-	.	.	.	.	.	.	.	.	.	.	.	.	.	.	.	.	.	.	.	.
4139	1	IL3RA	NM_001267713	chrX	1415508	1461582	+	.	.	.	.	.	.	.	.	.	.	.	.	.	.	.	.	.	.	.	.
530	3	ASMT	NM_001171038	chrX	1693940	1721974	+	.	.	.	.	.	.	.	.	.	.	.	.	.	.	.	.	.	.	.	.
3578	1	DHRSX	NM_145177	chrX	2147552	2429015	-	.	.	.	.	.	.	.	.	.	.	.	.	.	.	.	.	.	.	.	.
1011	2,25	CD99	NM_001122898	chrX	2619227	2669350	+	.	.	.	.	.	.	.	.	.	.	.	.	.	.	.	.	.	.	.	.
1256	2	ARSD	NM_001669	chrX	2832010	2857416	-	.	.	.	.	.	.	.	.	.	.	.	.	.	.	.	.	.	.	.	.
3047	1	ARSE	NM_000047	chrX	2862672	2892494	-	.	.	.	.	.	.	.	.	.	.	.	.	.	.	.	.	.	.	.	.
3048	1	ARSH	NM_001011719	chrX	2934653	2961426	+	.	.	.	.	.	.	.	.	.	.	.	.	.	.	.	.	.	.	.	.
2265	1,5	MXRA5	NM_015419	chrX	3236607	3274684	-	.	.	.	.	.	.	.	.	.	.	.	.	.	.	.	.	.	.	.	.
4947	1	PRKX	NM_005044	chrX	3532383	3641675	-	.	.	.	.	.	.	.	.	.	.	.	.	.	.	.	.	.	.	.	.
4585	1	NLGN4X	NM_181332	chrX	5818082	6156706	-	.	.	.	.	.	.	.	.	.	.	.	.	.	.	.	.	.	.	.	.
1785	2	STS	NM_000351	chrX	7147471	7282682	+	CL529216	chrX	7254942	+	.	.	.	.	.	.	.	.	.	.	.	.	.	.	.	.
6760	0,5	PNPLA4	NM_004650	chrX	7826803	7855475	-	.	.	.	.	.	.	.	.	.	.	.	.	.	.	.	.	.	.	.	.
2400	1,5	SHROOM2	NM_001649	chrX	9714495	9877481	+	.	.	.	.	.	.	.	.	.	.	.	.	.	.	.	.	.	.	.	.
3380	1	CLCN4	NM_001256944	chrX	10084984	10165699	+	.	.	.	.	.	.	.	.	.	.	.	.	.	.	.	.	.	.	.	.
4398	1	MID1	NM_000381	chrX	10373349	10548674	-	.	.	.	.	.	.	.	.	.	.	.	.	.	.	.	.	.	.	.	.
4023	1	HCCS	NM_005333	chrX	11039326	11051125	+	.	.	.	.	.	.	.	.	.	.	.	.	.	.	.	.	.	.	.	.
835	2,5	FRMPD4	NM_014728	chrX	12066505	12652563	+	.	.	.	.	.	.	.	.	.	.	.	.	.	.	.	.	.	.	.	.
4956	1	PRPS2	NM_002765	chrX	12719394	12752267	+	CL529594	chrX	12729654	-	.	.	.	.	.	.	.	.	.	.	.	.	.	.	.	.
5478	1	TLR7	NM_016562	chrX	12795122	12818401	+	.	.	.	.	.	.	.	.	.	.	.	.	.	.	.	.	.	.	.	.
3103	1	ATXN3L	NM_001135995	chrX	13246688	13248439	-	.	.	.	.	.	.	.	.	.	.	.	.	.	.	.	.	.	.	.	.
5046	1	RAB9A	NM_004251	chrX	13617160	13637866	+	CL529218	chrX	13630872	+	.	.	.	.	.	.	.	.	.	.	.	.	.	.	.	.
5046	1	RAB9A	NM_004251	chrX	13617160	13637866	+	CL800630	chrX	13629233	+	.	.	.	.	.	.	.	.	.	.	.	.	.	.	.	.
3903	1	GLRA2	NM_002063	chrX	14457340	14659854	+	.	.	.	.	.	.	.	.	.	.	.	.	.	.	.	.	.	.	.	.
3733	1	FANCB	NM_001018113	chrX	14771449	14801105	-	.	.	.	.	AY516189	chrX	14800821	+	.	.	.	.	.	.	.	.	.	.	.	.
4425	1	MOSPD2	NM_152581	chrX	14801447	14849380	+	.	.	.	.	.	.	.	.	.	.	.	.	.	.	.	.	.	.	.	.
4795	1	PIGA	NR_033836	chrX	15247493	15263597	-	.	.	.	.	.	.	.	.	.	.	.	.	.	.	.	.	.	.	.	.
59	6,75	FIGF	NM_004469	chrX	15273633	15312456	-	.	.	.	.	.	.	.	.	.	.	.	.	.	.	.	.	.	.	.	.
6744	0,5	PIR	NM_003662	chrX	15312844	15421632	-	.	.	.	.	.	.	.	.	.	.	.	.	.	.	.	.	.	.	.	.
3154	1	BMX	NM_001721	chrX	15435351	15484573	+	.	.	.	.	.	.	.	.	.	.	.	.	.	.	.	.	.	.	.	.
3254	1	CA5B	NM_007220	chrX	15666332	15715669	+	.	.	.	.	.	.	.	.	.	.	.	.	.	.	.	.	.	.	.	.
2608	1,25	GRPR	NM_005314	chrX	16051344	16081562	+	.	.	.	.	.	.	.	.	.	.	.	.	.	.	.	.	.	.	.	.
3485	1	CTPS2	NM_175859	chrX	16516042	16640696	-	CL529220	chrX	16520832	-	.	.	.	.	.	.	.	.	.	.	.	.	.	.	.	.
2371	1,5	REPS2	NM_001080975	chrX	16874734	17081324	+	.	.	.	.	.	.	.	.	.	.	.	.	.	.	.	.	.	.	.	.
5056	1	RAI2	NR_033349	chrX	17728089	17788868	-	.	.	.	.	.	.	.	.	.	.	.	.	.	.	.	.	.	.	.	.
3142	1	BEND2	NM_001184767	chrX	18098977	18148945	-	.	.	.	.	.	.	.	.	.	.	.	.	.	.	.	.	.	.	.	.
806	2,5	CDKL5	NM_003159	chrX	18353645	18581670	+	AY517434.1	chrX	18417780	-	.	.	.	.	.	.	.	.	.	.	.	.	.	.	.	.
4894	1	PPEF1	NM_152224	chrX	18618965	18755955	+	.	.	.	.	.	.	.	.	.	.	.	.	.	.	.	.	.	.	.	.
4785	1	PHKA2	NM_000292	chrX	18820336	18912401	-	.	.	.	.	.	.	.	.	.	.	.	.	.	.	.	.	.	.	.	.
4741	1	PDHA1	NM_001173456	chrX	19271931	19289746	+	.	.	.	.	.	.	.	.	.	.	.	.	.	.	.	.	.	.	.	.
1515	2	MAP3K15	NM_001001671	chrX	19288096	19443300	-	.	.	.	.	.	.	.	.	.	.	.	.	.	.	.	.	.	.	.	.
1755	2	SH3KBP1	NM_001184960	chrX	19462003	19599040	-	CL529462	chrX	19736234	-	.	.	.	.	.	.	.	.	.	.	.	.	.	.	.	.
3497	1	CXorf23	NM_198279	chrX	19840900	19898303	-	.	.	.	.	.	.	.	.	.	.	.	.	.	.	.	.	.	.	.	.
4353	1	MAP7D2	NM_152780	chrX	19934751	20045035	-	.	.	.	.	.	.	.	.	.	.	.	.	.	.	.	.	.	.	.	.
949	2,5	RPS6KA3	NM_004586	chrX	20077949	20194671	-	CL800236	chrX	20175122	+	.	.	.	.	.	.	.	.	.	.	.	.	.	.	.	.

Table S2

tumor associated genes								HIV				MLV				MMTV				MMTV(SIN)				MMTV(SIN)arrest			
rank	score	gene symbol	RefSEq	chrom	txStart	txEnd	strand	integrant	chr	position	strand	integrant	chr	position	strand	integrant	chr	position	strand	integrant	chr	position	strand	integrant	chr	position	strand
2080	1,5	CNKSR2	NM_001168649	chrX	21302456	21570901	+	.	.	.	.	.	.	.	.	.	.	.	.	s1572_239	chrX	21445410	-	.	.	.	.
5297	1	SMPX	NM_014332	chrX	21634010	21686199	-	.	.	.	.	.	.	.	.	.	.	.	.	.	.	.	.	.	.	.	.
5728	1	YY2	NM_206923	chrX	21784025	21786766	+	.	.	.	.	.	.	.	.	.	.	.	.	.	.	.	.	.	.	.	.
5298	1	SMS	NM_001258423	chrX	21868611	21922876	+	.	.	.	.	.	.	.	.	.	.	.	.	.	.	.	.	.	.	.	.
4780	1	PHEX	NM_000444	chrX	21960841	22176399	+	.	.	.	.	.	.	.	.	.	.	.	.	.	.	.	.	.	.	.	.
5839	1	ZNF645	NM_152577	chrX	22200950	22202497	+	.	.	.	.	.	.	.	.	.	.	.	.	.	.	.	.	.	.	.	.
4979	1	PTCHD1	NM_173495	chrX	23262905	23324839	+	.	.	.	.	.	.	.	.	.	.	.	.	.	.	.	.	.	.	.	.
5950	0,75	PRDX4	NM_006406	chrX	23595565	23614435	+	.	.	.	.	.	.	.	.	.	.	.	.	.	.	.	.	.	.	.	.
425	3,5	SAT1	NM_002970	chrX	23711195	23714248	+	.	.	.	.	.	.	.	.	.	.	.	.	.	.	.	.	.	.	.	.
1380	2	EIF2S3	NM_001415	chrX	23982985	24006851	+	.	.	.	.	.	.	.	.	.	.	.	.	.	.	.	.	.	.	.	.
3729	1	FAM48B1	NM_001136234	chrX	24290798	24293462	+	.	.	.	.	.	.	.	.	.	.	.	.	.	.	.	.	.	.	.	.
4726	1	PCYT1B	NM_004845	chrX	24486124	24575376	-	.	.	.	.	.	.	.	.	.	.	.	.	.	.	.	.	.	.	.	.
921	2,5	POLA1	NM_016937	chrX	24621984	24925023	+	CL529221	chrX	24763653	+	.	.	.	.	.	.	.	.	.	.	.	.	.	.	.	.
6590	0,5	MAGEB18	NM_173699	chrX	26066380	26068774	+	.	.	.	.	.	.	.	.	.	.	.	.	.	.	.	.	.	.	.	.
4329	1	MAGEB10	NM_182506	chrX	27736027	27751052	+	.	.	.	.	.	.	.	.	.	.	.	.	.	.	.	.	.	.	.	.
1469	2	IL1RAPL1	NM_014271	chrX	28515601	29883938	+	.	.	.	.	.	.	.	.	.	.	.	.	.	.	.	.	.	.	.	.
7411	0,25	MAGEB2	NM_002364	chrX	30143595	30148127	+	.	.	.	.	.	.	.	.	.	.	.	.	.	.	.	.	.	.	.	.
7410	0,25	MAGEB1	NM_002363	chrX	30171768	30180076	+	.	.	.	.	.	.	.	.	.	.	.	.	.	.	.	.	.	.	.	.
406	3,5	NR0B1	NM_000475	chrX	30232459	30237416	-	.	.	.	.	.	.	.	.	.	.	.	.	.	.	.	.	.	.	.	.
3894	1	GK	NM_001205019	chrX	30581396	30659498	+	.	.	.	.	.	.	.	.	.	.	.	.	.	.	.	.	.	.	.	.
5497	1	TMEM47	NM_031442	chrX	34555101	34585326	-	.	.	.	.	.	.	.	.	.	.	.	.	.	.	.	.	.	.	.	.
3496	1	CXorf22	NM_152632	chrX	35847771	35918190	+	.	.	.	.	.	.	.	.	.	.	.	.	.	.	.	.	.	.	.	.
7105	0,5	XK	NM_021083	chrX	37430051	37476322	+	.	.	.	.	.	.	.	.	.	.	.	.	.	.	.	.	.	.	.	.
3501	1	CYBB	NM_000397	chrX	37524213	37557658	+	.	.	.	.	.	.	.	.	.	.	.	.	.	.	.	.	.	.	.	.
5353	1	SRPX	NM_006307	chrX	37893531	37965121	-	.	.	.	.	.	.	.	.	.	.	.	.	.	.	.	.	.	.	.	.
4673	1	OTC	NM_000531	chrX	38096679	38165647	+	.	.	.	.	.	.	.	.	.	.	.	.	.	.	.	.	.	.	.	.
6055	0,5	BCOR	NM_001123383	chrX	39795442	39921526	-	.	.	.	.	.	.	.	.	.	.	.	.	.	.	.	.	.	.	.	.
7271	0,25	DDX3X	NM_001193417	chrX	41077594	41094468	+	.	.	.	.	.	.	.	.	.	.	.	.	.	.	.	.	.	.	.	.
4652	1	NYX	NM_022567	chrX	41191656	41219849	+	.	.	.	.	.	.	.	.	.	.	.	.	.	.	.	.	.	.	.	.
1289	2	CASK	NM_001126055	chrX	41259132	41667231	-	CL800661	chrX	41581044	+	.	.	.	.	.	.	.	.	.	.	.	.	.	.	.	.
615	3	MAOA	NM_001270458	chrX	43399098	43491015	+	.	.	.	.	.	.	.	.	.	.	.	.	.	.	.	.	.	.	.	.
1934	1,75	MAOB	NM_000898	chrX	43510800	43626665	-	.	.	.	.	.	.	.	.	.	.	.	.	.	.	.	.	.	.	.	.
6655	0,5	NDP	NM_000266	chrX	43692967	43717865	-	.	.	.	.	.	.	.	.	.	.	.	.	.	.	.	.	.	.	.	.
3624	1	DUSP21	NM_022076	chrX	44588192	44589078	+	.	.	.	.	.	.	.	.	.	.	.	.	.	.	.	.	.	.	.	.
3498	1	CXorf36	NM_024689	chrX	44906499	44945090	-	.	.	.	.	.	.	.	.	.	.	.	.	.	.	.	.	.	.	.	.
3366	1	CHST7	NM_019886	chrX	46318135	46342782	+	.	.	.	.	.	.	.	.	.	.	.	.	.	.	.	.	.	.	.	.
5153	1	RP2	NM_006915	chrX	46581290	46626735	+	.	.	.	.	.	.	.	.	.	.	.	.	.	.	.	.	.	.	.	.
6740	0,5	PHF16	NM_001077445	chrX	46656679	46805585	+	.	.	.	.	.	.	.	.	.	.	.	.	.	.	.	.	.	.	.	.
5114	1	RGN	NM_152869	chrX	46822718	46837656	+	.	.	.	.	.	.	.	.	.	.	.	.	.	.	.	.	.	.	.	.
4537	1	NDUFB11	NM_001135998	chrX	46886558	46889553	-	.	.	.	.	.	.	.	.	.	.	.	.	.	.	.	.	.	.	.	.
2367	1,5	RBM10	NM_001204466	chrX	46889560	46931158	+	.	.	.	.	.	.	.	.	.	.	.	.	.	.	.	.	.	.	.	.
997	2,25	ARAF	NM_001654	chrX	47305442	47316264	+	.	.	.	.	.	.	.	.	.	.	.	.	.	.	.	.	.	.	.	.
1809	2	TIMP1	NM_003254	chrX	47326633	47331134	+	BH609626	chrX	47328401	+	.	.	.	.	.	.	.	.	.	.	.	.	.	.	.	.
1307	2	CFP	NM_002621	chrX	47368555	47374648	-	.	.	.	.	.	.	.	.	.	.	.	.	.	.	.	.	.	.	.	.
566	3	ELK1	NM_001114123	chrX	47379862	47394947	-	.	.	.	.	.	.	.	.	.	.	.	.	.	.	.	.	.	.	.	.
5767	1	ZNF182	NM_001178099	chrX	47719193	47748338	-	.	.	.	.	.	.	.	.	.	.	.	.	.	.	.	.	.	.	.	.
5838	1	ZNF630	NM_001037735	chrX	47802510	47815452	-	.	.	.	.	.	.	.	.	.	.	.	.	.	.	.	.	.	.	.	.
1777	2	SSX1	NM_005635	chrX	47999740	48011823	+	.	.	.	.	.	.	.	.	.	.	.	.	.	.	.	.	.	.	.	.
506	3,25	SSX4	NM_175729	chrX	48146467	48156288	-	.	.	.	.	.	.	.	.	.	.	.	.	.	.	.	.	.	.	.	.
4880	1	PORCN	NM_203474	chrX	48253115	48264146	+	.	.	.	.	.	.	.	.	.	.	.	.	.	.	.	.	.	.	.	.
3641	1	EBP	NM_006579	chrX	48265107	48272048	+	.	.	.	.	.	.	.	.	.	.	.	.	.	.	.	.	.	.	.	.

Table S2

tumor associated genes								HIV				MLV				MMTV				MMTV(SIN)				MMTV(SIN)arrest			
rank	score	gene symbol	RefSEq	chrom	txStart	txEnd	strand	integrant	chr	position	strand	integrant	chr	position	strand	integrant	chr	position	strand	integrant	chr	position	strand	integrant	chr	position	strand
1852	2	WAS	NM_000377	chrX	48427129	48434761	+	.	.	.	.	.	.	.	.	.	.	.	.	.	.	.	.	.	.	.	.
962	2,5	SUV39H1	NM_003173	chrX	48440074	48452350	+	.	.	.	.	.	.	.	.	.	.	.	.	.	.	.	.	.	.	.	.
259	4	GATA1	NM_002049	chrX	48529925	48537661	+	.	.	.	.	.	.	.	.	.	.	.	.	.	.	.	.	.	.	.	.
378	3,5	HDAC6	NM_006044	chrX	48545430	48568324	+	.	.	.	.	.	.	.	.	.	.	.	.	.	.	.	.	.	.	.	.
7302	0,25	ERAS	NM_181532	chrX	48572226	48573492	+	.	.	.	.	.	.	.	.	.	.	.	.	.	.	.	.	.	.	.	.
308	4	TFE3	NM_006521	chrX	48771185	48787934	-	.	.	.	.	.	.	.	.	.	.	.	.	.	.	.	.	.	.	.	.
6755	0,5	PLP2	NM_002668	chrX	48915127	48918412	+	.	.	.	.	.	.	.	.	.	.	.	.	.	.	.	.	.	.	.	.
759	2,75	SYP	NM_003179	chrX	48931208	48943605	-	.	.	.	.	.	.	.	.	.	.	.	.	.	.	.	.	.	.	.	.
360	3,5	CACNA1F	NM_001256789	chrX	48948466	48976777	-	.	.	.	.	.	.	.	.	.	.	.	.	.	.	.	.	.	.	.	.
2339	1,5	PPP1R3F	NM_001184745	chrX	49013249	49031499	+	.	.	.	.	.	.	.	.	.	.	.	.	.	.	.	.	.	.	.	.
7324	0,25	GAGE2C	NM_001472	chrX	49103591	49181542	+	.	.	.	.	.	.	.	.	.	.	.	.	.	.	.	.	.	.	.	.
7323	0,25	GAGE1	NM_001040663	chrX	49250559	49260086	+	.	.	.	.	.	.	.	.	.	.	.	.	.	.	.	.	.	.	.	.
4686	1	PAGE1	NM_003785	chrX	49339007	49347307	-	.	.	.	.	.	.	.	.	.	.	.	.	.	.	.	.	.	.	.	.
3381	1	CLCN5	NM_001127899	chrX	49573964	49750632	+	.	.	.	.	.	.	.	.	.	.	.	.	.	.	.	.	.	.	.	.
1233	2	AKAP4	NM_003886	chrX	49842150	49852404	-	.	.	.	.	.	.	.	.	.	.	.	.	.	.	.	.	.	.	.	.
3288	1	CCNB3	NM_033670	chrX	50044279	50111651	+	.	.	.	.	.	.	.	.	.	.	.	.	.	.	.	.	.	.	.	.
3570	1	DGKK	NM_001013742	chrX	50128618	50230461	-	.	.	.	.	.	.	.	.	.	.	.	.	.	.	.	.	.	.	.	.
6060	0,5	BMP15	NM_005448	chrX	50670474	50676381	+	.	.	.	.	.	.	.	.	.	.	.	.	.	.	.	.	.	.	.	.
7413	0,25	MAGED4	NM_001098800	chrX	51944747	51952104	+	.	.	.	.	.	.	.	.	.	.	.	.	.	.	.	.	.	.	.	.
2832	1,25	XAGE1A	NM_001097592	chrX	52557778	52562922	-	.	.	.	.	.	.	.	.	.	.	.	.	.	.	.	.	.	.	.	.
7619	0,25	XAGE1B	NM_001097596	chrX	52557778	52562769	-	.	.	.	.	.	.	.	.	.	.	.	.	.	.	.	.	.	.	.	.
7620	0,25	XAGE1D	NM_020411	chrX	52557778	52562922	-	.	.	.	.	.	.	.	.	.	.	.	.	.	.	.	.	.	.	.	.
685	3	SSX2	NM_175698	chrX	52797032	52807342	+	.	.	.	.	.	.	.	.	.	.	.	.	.	.	.	.	.	.	.	.
3952	1	GPR173	NM_018969	chrX	53095230	53126521	+	.	.	.	.	.	.	.	.	.	.	.	.	.	.	.	.	.	.	.	.
2812	1,25	TSPYL2	NM_022117	chrX	53128266	53134453	+	.	.	.	.	.	.	.	.	.	.	.	.	.	.	.	.	.	.	.	.
4211	1	KDM5C	NM_001146702	chrX	53237227	53271329	-	.	.	.	.	.	.	.	.	.	.	.	.	.	.	.	.	.	.	.	.
6482	0,5	IQSEC2	NM_001111125	chrX	53278782	53367247	-	.	.	.	.	.	.	.	.	.	.	.	.	.	.	.	.	.	.	.	.
2408	1,5	SMC1A	NM_006306	chrX	53417794	53466343	-	.	.	.	.	.	.	.	.	.	.	.	.	.	.	.	.	.	.	.	.
4088	1	HSD17B10	NM_004493	chrX	53474930	53478048	-	.	.	.	.	.	.	.	.	.	.	.	.	.	.	.	.	.	.	.	.
593	3	HUWE1	NM_031407	chrX	53575781	53730422	-	AY516978.1	chrX	53688318	+	.	.	.	.	.	.	.	.	.	.	.	.	.	.	.	.
5702	1	WNK3	NM_001002838	chrX	54235980	54401163	-	.	.	.	.	.	.	.	.	.	.	.	.	.	.	.	.	.	.	.	.
4757	1	PFKFB1	NM_002625	chrX	54976314	55037236	-	.	.	.	.	.	.	.	.	.	.	.	.	.	.	.	.	.	.	.	.
3000	1	APEX2	NM_014481	chrX	55043504	55050937	+	.	.	.	.	.	.	.	.	.	.	.	.	.	.	.	.	.	.	.	.
2945	1	ALAS2	NM_000032	chrX	55052212	55074222	-	.	.	.	.	.	.	.	.	.	.	.	.	.	.	.	.	.	.	.	.
2236	1,5	MAGEH1	NM_014061	chrX	55495262	55496724	+	.	.	.	.	.	.	.	.	.	.	.	.	.	.	.	.	.	.	.	.
5613	1	UBQLN2	NM_013444	chrX	56606750	56610168	+	.	.	.	.	.	.	.	.	.	.	.	.	.	.	.	.	.	.	.	.
6031	0,5	ARHGEF9	NM_001173480	chrX	62771572	62891756	-	CL799573	chrX	62856639	+	.	.	.	.	.	.	.	.	.	.	.	.	.	.	.	.
572	3	FAM123B	NM_152424	chrX	63321721	63342349	-	.	.	.	.	.	.	.	.	.	.	.	.	.	.	.	.	.	.	.	.
7116	0,5	ZC4H2	NM_001243804	chrX	64052406	64171349	-	.	.	.	.	.	.	.	.	.	.	.	.	.	.	.	.	.	.	.	.
5734	1	ZC3H12B	NM_001010888	chrX	64625339	64644492	+	.	.	.	.	.	.	.	.	.	.	.	.	.	.	.	.	.	.	.	.
401	3,5	MSN	NM_002444	chrX	64804235	64878518	+	.	.	.	.	.	.	.	.	.	.	.	.	.	.	.	.	.	.	.	.
2457	1,5	VSIG4	NM_001184830	chrX	65158304	65176692	-	.	.	.	.	.	.	.	.	.	.	.	.	.	.	.	.	.	.	.	.
855	2,5	HEPH	NM_001130860	chrX	65300803	65403955	+	.	.	.	.	.	.	.	.	.	.	.	.	.	.	.	.	.	.	.	.
73	6,25	AR	NM_000044	chrX	66680598	66867186	+	.	.	.	.	AY516061	chrX	66701240	+	.	.	.	.	.	.	.	.	.	.	.	.
73	6,25	AR	NM_000044	chrX	66680598	66867186	+	.	.	.	.	AY516824	chrX	66700477	+	.	.	.	.	.	.	.	.	.	.	.	.
1779	2	STARD8	NM_001142504	chrX	67830206	67862409	+	.	.	.	.	.	.	.	.	.	.	.	.	.	.	.	.	.	.	.	.
3655	1	EFNB1	NM_004429	chrX	67965564	67978731	+	.	.	.	.	.	.	.	.	.	.	.	.	.	.	.	.	.	.	.	.
3644	1	EDA	NM_001005609	chrX	68752635	69176046	+	.	.	.	.	.	.	.	.	.	.	.	.	.	.	.	.	.	.	.	.
5460	1	TEX11	NM_001003811	chrX	69665514	70045292	-	.	.	.	.	.	.	.	.	.	.	.	.	.	.	.	.	.	.	.	.
834	2,5	FOXO4	NM_001170931	chrX	70232723	70240109	+	.	.	.	.	.	.	.	.	.	.	.	.	.	.	.	.	.	.	.	.
2201	1,5	IL2RG	NM_000206	chrX	70243978	70248206	-	.	.	.	.	.	.	.	.	.	.	.	.	.	.	.	.	.	.	.	.

Table S2

tumor associated genes								HIV				MLV				MMTV				MMTV(SIN)				MMTV(SIN)arrest			
rank	score	gene symbol	RefSEq	chrom	txStart	txEnd	strand	integrant	chr	position	strand	integrant	chr	position	strand	integrant	chr	position	strand	integrant	chr	position	strand	integrant	chr	position	strand
6604	0,5	MED12	NM_005120	chrX	70255130	70279029	+	.	.	.	.	.	.	.	.	.	.	.	.	.	.	.	.	.	.	.	.
6675	0,5	NLGN3	NM_018977	chrX	70281405	70307776	+	.	.	.	.	.	.	.	.	.	.	.	.	.	.	.	.	.	.	.	.
1580	2	NONO	NM_007363	chrX	70419766	70437743	+	.	.	.	.	.	.	.	.	.	.	.	.	.	.	.	.	.	.	.	.
432	3,5	TAF1	NM_138923	chrX	70502838	70602580	+	AY517054.1	chrX	70567274	+	.	.	.	.	.	.	.	.	.	.	.	.	.	.	.	.
432	3,5	TAF1	NM_138923	chrX	70502838	70602580	+	CL529260	chrX	70559522	-	.	.	.	.	.	.	.	.	.	.	.	.	.	.	.	.
4660	1	OGT	NM_181673	chrX	70669636	70712472	+	.	.	.	.	.	.	.	.	.	.	.	.	.	.	.	.	.	.	.	.
3493	1	CXCR3	NM_001504	chrX	70752490	70755092	-	.	.	.	.	.	.	.	.	.	.	.	.	.	.	.	.	.	.	.	.
5110	1	RGAG4	NM_001024455	chrX	71263685	71268476	-	.	.	.	.	.	.	.	.	.	.	.	.	.	.	.	.	.	.	.	.
3786	1	FLJ44635	NM_207422	chrX	71280758	71298325	+	.	.	.	.	.	.	.	.	.	.	.	.	.	.	.	.	.	.	.	.
6183	0,5	CITED1	NM_004143	chrX	71438212	71443562	-	.	.	.	.	.	.	.	.	.	.	.	.	.	.	.	.	.	.	.	.
1444	2	HDAC8	NM_018486	chrX	71466090	71709678	-	.	.	.	.	.	.	.	.	.	.	.	.	.	.	.	.	2186_190_5	chrX	71598419	+
4784	1	PHKA1	NM_001172436	chrX	71715388	71850754	-	.	.	.	.	.	.	.	.	.	.	.	.	.	.	.	.	.	.	.	.
6271	0,5	DMRTC1	NM_033053	chrX	72008583	72012347	-	.	.	.	.	.	.	.	.	.	.	.	.	.	.	.	.	.	.	.	.
6713	0,5	PABPC1L2B	NM_001042506	chrX	72140076	72142276	+	.	.	.	.	.	.	.	.	.	.	.	.	.	.	.	.	.	.	.	.
1556	2	NAP1L2	NM_021963	chrX	72348861	72351435	-	.	.	.	.	.	.	.	.	.	.	.	.	.	.	.	.	.	.	.	.
6175	0,5	CHIC1	NM_001039840	chrX	72699708	72823662	+	AY517373.1	chrX	72715346	+	.	.	.	.	.	.	.	.	.	.	.	.	.	.	.	.
5713	1	XIST	NR_001564	chrX	72957210	72989313	-	.	.	.	.	.	.	.	.	.	.	.	.	.	.	.	.	.	.	.	.
5255	1	SLC16A2	NM_006517	chrX	73558052	73670489	+	.	.	.	.	.	.	.	.	.	.	.	.	.	.	.	.	.	.	.	.
1493	2	KIAA2022	NM_001008537	chrX	73869415	74062012	-	.	.	.	.	.	.	.	.	.	.	.	.	.	.	.	.	.	.	.	.
5979	0,5	ABCB7	NM_004299	chrX	74189829	74292857	-	.	.	.	.	.	.	.	.	.	.	.	.	.	.	.	.	.	.	.	.
5645	1	UPRT	NM_145052	chrX	74410618	74441457	+	.	.	.	.	.	.	.	.	.	.	.	.	.	.	.	.	.	.	.	.
888	2,5	MAGEE1	NM_020932	chrX	75564449	75568150	+	.	.	.	.	.	.	.	.	.	.	.	.	.	.	.	.	.	.	.	.
532	3	ATRX	NM_138270	chrX	76647011	76928375	-	BH609629	chrX	76843688	+	.	.	.	.	.	.	.	.	.	.	.	.	.	.	.	.
3440	1	COX7B	NM_001866	chrX	77041616	77047537	+	.	.	.	.	.	.	.	.	.	.	.	.	.	.	.	.	.	.	.	.
3095	1	ATP7A	NM_000052	chrX	77052849	77192548	+	.	.	.	.	.	.	.	.	.	.	.	.	.	.	.	.	.	.	.	.
4765	1	PGAM4	NM_001029891	chrX	77110113	77111791	-	.	.	.	.	.	.	.	.	.	.	.	.	.	.	.	.	.	.	.	.
491	3,25	PGK1	NM_000291	chrX	77246321	77268980	+	.	.	.	.	.	.	.	.	.	.	.	.	.	.	.	.	.	.	.	.
6568	0,5	LPAR4	NM_005296	chrX	77889861	77899234	+	.	.	.	.	.	.	.	.	.	.	.	.	.	.	.	.	.	.	.	.
4680	1	P2RY10	NM_198333	chrX	78087484	78104094	+	.	.	.	.	.	.	.	.	.	.	.	.	.	.	.	.	.	.	.	.
3953	1	GPR174	NM_032553	chrX	78313124	78314382	+	.	.	.	.	.	.	.	.	.	.	.	.	.	.	.	.	.	.	.	.
1481	2	ITM2A	NM_004867	chrX	78502536	78509705	-	.	.	.	.	.	.	.	.	.	.	.	.	.	.	.	.	.	.	.	.
1795	2	TBX22	NM_001109879	chrX	79156910	79173924	+	.	.	.	.	.	.	.	.	.	.	.	.	.	.	.	.	.	.	.	.
3728	1	FAM46D	NM_001170574	chrX	79477658	79587466	+	.	.	.	.	.	.	.	.	.	.	.	.	.	.	.	.	.	.	.	.
3503	1	CYLC1	NM_021118	chrX	83002825	83028282	+	.	.	.	.	.	.	.	.	.	.	.	.	.	.	.	.	.	.	.	.
5165	1	RPS6KA6	NM_014496	chrX	83200009	83329599	-	.	.	.	.	.	.	.	.	.	.	.	.	.	.	.	.	.	.	.	.
5184	1	SATL1	NM_001012980	chrX	84233947	84250630	-	.	.	.	.	.	.	.	.	.	.	.	.	.	.	.	.	.	.	.	.
7140	0,5	ZNF711	NM_021998	chrX	84385652	84415025	+	CL799553	chrX	84406941	-	.	.	.	.	.	.	.	.	.	.	.	.	.	.	.	.
6250	0,5	DACH2	NM_001139514	chrX	85290110	85974261	+	.	.	.	.	.	.	.	.	.	.	.	.	.	.	.	.	.	.	.	.
1494	2	KLHL4	NM_019117	chrX	86659370	86811706	+	.	.	.	.	.	.	.	.	.	.	.	.	.	.	.	.	.	.	.	.
4683	1	PABPC5	NM_080832	chrX	90576252	90580239	+	.	.	.	.	.	.	.	.	.	.	.	.	.	.	.	.	.	.	.	.
2307	1,5	PCDH11X	NM_001168362	chrX	90977115	91764884	+	BH609624	chrX	91198244	+	.	.	.	.	.	.	.	.	.	.	.	.	46_69_1	chrX	91533674	-
1557	2	NAP1L3	NM_004538	chrX	92812580	92815338	-	.	.	.	.	.	.	.	.	.	.	.	.	.	.	.	.	.	.	.	.
3582	1	DIAPH2	NM_007309	chrX	95826317	96611493	+	AY517263.1	chrX	96002088	+	.	.	.	.	.	.	.	.	30_2_1591_5	chrX	96377292	+	.	.	.	.
5156	1	RPA4	NM_013347	chrX	96025562	96027106	+	.	.	.	.	.	.	.	.	.	.	.	.	.	.	.	.	.	.	.	.
4708	1	PCDH19	NM_001184880	chrX	99433297	99551927	-	.	.	.	.	.	.	.	.	.	.	.	.	.	.	.	.	.	.	.	.
5354	1	SRPX2	NM_014467	chrX	99785818	99812952	+	.	.	.	.	.	.	.	.	.	.	.	.	.	.	.	.	.	.	.	.
5714	1	XKRX	NM_212559	chrX	100055086	100070554	-	.	.	.	.	.	.	.	.	.	.	.	.	.	.	.	.	.	.	.	.
5496	1	TMEM35	NM_021637	chrX	100220491	100238011	+	.	.	.	.	.	.	.	.	.	.	.	.	.	.	.	.	.	.	.	.
1305	2	CENPI	NM_006733	chrX	100241453	100304634	+	.	.	.	.	.	.	.	.	.	.	.	.	.	.	.	.	.	.	.	.
5428	1	TAF7L	NM_024885	chrX	100409896	100434715	-	.	.	.	.	.	.	.	.	.	.	.	.	.	.	.	.	.	.	.	.
2031	1,5	BTK	NM_000061	chrX	100491090	100527868	-	.	.	.	.	.	.	.	.	.	.	.	.	.	.	.	.	.	.	.	.

Table S2

tumor associated genes								HIV				MLV				MMTV				MMTV(SIN)				MMTV(SIN)arrest			
rank	score	gene symbol	RefSEq	chrom	txStart	txEnd	strand	integrant	chr	position	strand	integrant	chr	position	strand	integrant	chr	position	strand	integrant	chr	position	strand	integrant	chr	position	strand
3898	1	GLA	NM_000169	chrX	100539434	100549657	-	.	.	.	.	.	.	.	.	.	.	.	.	.	.	.	.	.	.	.	.
3042	1	ARMCX1	NM_016608	chrX	100692169	100696331	+	.	.	.	.	.	.	.	.	.	.	.	.	.	.	.	.	.	.	.	.
3044	1	ARMCX3	NM_177948	chrX	100765006	100769487	+	.	.	.	.	.	.	.	.	.	.	.	.	.	.	.	.	.	.	.	.
3043	1	ARMCX2	NM_014782	chrX	100796925	100801519	-	.	.	.	.	.	.	.	.	.	.	.	.	.	.	.	.	.	.	.	.
5442	1	TCEAL2	NM_080390	chrX	101267315	101269340	+	.	.	.	.	.	.	.	.	.	.	.	.	.	.	.	.	.	.	.	.
5505	1	TMSB15A	NM_021992	chrX	101655265	101658355	-	.	.	.	.	.	.	.	.	.	.	.	.	.	.	.	.	.	.	.	.
2172	1,5	GPRASP1	NM_001099410	chrX	101792949	101800666	+	.	.	.	.	.	.	.	.	.	.	.	.	.	.	.	.	.	.	.	.
6405	0,5	GPRASP2	NM_001184876	chrX	101853759	101859317	+	.	.	.	.	.	.	.	.	.	.	.	.	.	.	.	.	.	.	.	.
3143	1	BEX1	NM_018476	chrX	102204236	102205824	-	.	.	.	.	.	.	.	.	.	.	.	.	.	.	.	.	.	.	.	.
5443	1	TCEAL5	NM_001012979	chrX	102415273	102418453	-	.	.	.	.	.	.	.	.	.	.	.	.	.	.	.	.	.	.	.	.
6058	0,5	BEX2	NM_001168399	chrX	102450929	102452539	-	.	.	.	.	.	.	.	.	.	.	.	.	.	.	.	.	.	.	.	.
5444	1	TCEAL7	NM_152278	chrX	102471769	102473907	+	.	.	.	.	.	.	.	.	.	.	.	.	.	.	.	.	.	.	.	.
7012	0,5	TCEAL1	NM_001006640	chrX	102770303	102772532	+	.	.	.	.	.	.	.	.	.	.	.	.	.	.	.	.	.	.	.	.
4424	1	MORF4L2	NM_001142430	chrX	102817081	102828402	-	.	.	.	.	.	.	.	.	.	.	.	.	.	.	.	.	.	.	.	.
2715	1,25	PLP1	NM_000533	chrX	102918409	102934203	+	.	.	.	.	.	.	.	.	.	.	.	.	.	.	.	.	.	.	.	.
5506	1	TMSB15B	NM_194324	chrX	103103855	103107219	+	.	.	.	.	.	.	.	.	.	.	.	.	.	.	.	.	.	.	.	.
4137	1	IL1RAPL2	NM_017416	chrX	103697651	104898478	+	.	.	.	.	.	.	.	.	.	.	.	.	.	.	.	.	646_114_3	chrX	104243619	-
4623	1	NRK	NM_198465	chrX	104953191	105089258	+	.	.	.	.	.	.	.	.	.	.	.	.	.	.	.	.	.	.	.	.
2264	1,5	MUM1L1	NM_152423	chrX	105298953	105339605	+	.	.	.	.	.	.	.	.	.	.	.	.	.	.	.	.	.	.	.	.
2101	1,5	CXorf57	NM_001184782	chrX	105741815	105809329	+	CL800245	chrX	105759739	-	.	.	.	.	.	.	.	.	.	.	.	.	.	.	.	.
6824	0,5	RNF128	NM_024539	chrX	105823723	105926902	+	.	.	.	.	.	.	.	.	.	.	.	.	.	.	.	.	.	.	.	.
656	3	PRPS1	NM_001204402	chrX	106758309	106780912	+	.	.	.	.	.	.	.	.	.	.	.	.	.	.	.	.	.	.	.	.
6780	0,5	PSMD10	NM_170750	chrX	107214090	107221530	-	.	.	.	.	.	.	.	.	.	.	.	.	.	.	.	.	.	.	.	.
1320	2	COL4A6	NM_033641	chrX	107285492	107568314	-	AY517310.1	chrX	107535401	+	.	.	.	.	.	.	.	.	485_295	chrX	107542059	+	.	.	.	.
1084	2,25	IRS4	NM_003604	chrX	107862382	107866263	-	.	.	.	.	.	.	.	.	.	.	.	.	.	.	.	.	.	.	.	.
584	3	GUCY2F	NM_001522	chrX	108502790	108611941	-	.	.	.	.	.	.	.	.	.	.	.	.	.	.	.	.	.	.	.	.
1986	1,5	ACSL4	NM_022977	chrX	108771219	108863277	-	AY516932.1	chrX	108794600	+	.	.	.	.	.	.	.	.	.	.	.	.	.	.	.	.
5491	1	TMEM164	NM_032227	chrX	109132997	109307672	+	CL800090	chrX	109229297	-	.	.	.	.	.	.	.	.	.	.	.	.	.	.	.	.
5109	1	RGAG1	NM_020769	chrX	109548940	109586218	+	.	.	.	.	.	.	.	.	.	.	.	.	.	.	.	.	.	.	.	.
6177	0,5	CHRDL1	NM_145234	chrX	109803739	109925732	-	.	.	.	.	.	.	.	.	.	.	.	.	.	.	.	.	.	.	.	.
634	3	PAK3	NM_001128166	chrX	110074168	110350829	+	.	.	.	.	.	.	.	.	.	.	.	.	.	.	.	.	.	.	.	.
3274	1	CAPN6	NM_014289	chrX	110374982	110400430	-	.	.	.	.	.	.	.	.	.	.	.	.	.	.	.	.	.	.	.	.
1030	2,25	DCX	NM_178152	chrX	110423662	110542116	-	.	.	.	.	.	.	.	.	.	.	.	.	.	.	.	.	.	.	.	.
2957	1	ALG13	NM_001039210	chrX	110811001	110820279	+	.	.	.	.	.	.	.	.	.	.	.	.	.	.	.	.	.	.	.	.
5564	1	TRPC5	NM_012471	chrX	110904197	111212660	-	.	.	.	.	.	.	.	.	.	.	.	.	.	.	.	.	.	.	.	.
6008	0,5	AMOT	NM_001113490	chrX	111904760	111953010	-	.	.	.	.	.	.	.	.	.	.	.	.	.	.	.	.	2966_270_1	chrX	111950310	-
6458	0,5	HTR2C	NM_000868	chrX	113724806	114050880	+	.	.	.	.	.	.	.	.	.	.	.	.	.	.	.	.	.	.	.	.
864	2,5	IL13RA2	NM_000640	chrX	114144793	114158463	-	.	.	.	.	.	.	.	.	.	.	.	.	.	.	.	.	.	.	.	.
919	2,5	PLS3	NM_001172335	chrX	114734074	114791435	+	CL529232	chrX	114753211	+	.	.	.	.	.	.	.	.	.	.	.	.	.	.	.	.
7156	0,25	AGTR2	NM_000686	chrX	115215985	115220253	+	.	.	.	.	.	.	.	.	.	.	.	.	.	.	.	.	.	.	.	.
7264	0,25	CXorf61	NM_001017978	chrX	115506879	115508165	-	.	.	.	.	.	.	.	.	.	.	.	.	.	.	.	.	.	.	.	.
7389	0,25	KLHL13	NM_001168301	chrX	116915803	117135331	-	.	.	.	.	.	.	.	.	.	.	.	.	.	.	.	.	.	.	.	.
5696	1	WDR44	NM_019045	chrX	117364063	117467951	+	.	.	.	.	.	.	.	.	.	.	.	.	.	.	.	.	.	.	.	.
7370	0,25	IL13RA1	NM_001560	chrX	117745586	117812524	+	.	.	.	.	.	.	.	.	.	.	.	.	.	.	.	.	.	.	.	.
4225	1	KIAA1210	NM_020721	chrX	118096625	118168570	-	.	.	.	.	.	.	.	.	.	.	.	.	.	.	.	.	.	.	.	.
1612	2	PGRMC1	NM_006667	chrX	118254238	118262457	+	.	.	.	.	.	.	.	.	.	.	.	.	.	.	.	.	.	.	.	.
5266	1	SLC25A43	NM_145305	chrX	118417285	118472465	+	.	.	.	.	.	.	.	.	.	.	.	.	.	.	.	.	.	.	.	.
2452	1,5	UBE2A	NM_003336	chrX	118592526	118602407	+	.	.	.	.	.	.	.	.	.	.	.	.	.	.	.	.	.	.	.	.
6673	0,5	NKRF	NM_017544	chrX	118606327	118623874	-	.	.	.	.	.	.	.	.	.	.	.	.	.	.	.	.	.	.	.	.
5134	1	RNF113A	NM_006978	chrX	118888522	118889819	-	.	.	.	.	.	.	.	.	.	.	.	.	.	.	.	.	.	.	.	.
4523	1	NDUFA1	NM_004541	chrX	118889761	118894657	+	.	.	.	.	.	.	.	.	.	.	.	.	.	.	.	.	.	.	.	.

Table S2

tumor associated genes								HIV				MLV				MMTV				MMTV(SIN)				MMTV(SIN)arrest			
rank	score	gene symbol	RefSEq	chrom	txStart	txEnd	strand	integrant	chr	position	strand	integrant	chr	position	strand	integrant	chr	position	strand	integrant	chr	position	strand	integrant	chr	position	strand
5732	1	ZBTB33	NM_001184742	chrX	119268637	119276279	+	.	.	.	.	.	.	.	.	.	.	.	.	.	.	.	.	.	.	.	.
3730	1	FAM70A	NM_001104544	chrX	119276532	119329419	-	.	.	.	.	.	.	.	.	.	.	.	.	.	.	.	.	.	.	.	.
3067	1	ATP1B4	NM_012069	chrX	119379967	119401132	+	.	.	.	.	.	.	.	.	.	.	.	.	.	.	.	.	.	.	.	.
4257	1	LAMP2	NM_001122606	chrX	119444030	119487232	-	.	.	.	.	.	.	.	.	.	.	.	.	.	.	.	.	.	.	.	.
3488	1	CUL4B	NM_001079872	chrX	119542473	119578845	-	CL529233	chrX	119564599	-	.	.	.	.	.	.	.	.	.	.	.	.	.	.	.	.
3214	1	C1GALT1C1	NM_001011551	chrX	119643556	119648033	-	.	.	.	.	.	.	.	.	.	.	.	.	.	.	.	.	.	.	.	.
7258	0,25	CT47A11	NM_173571	chrX	119900236	119903554	-	.	.	.	.	.	.	.	.	.	.	.	.	.	.	.	.	.	.	.	.
3907	1	GLUD2	NM_012084	chrX	120009142	120011477	+	.	.	.	.	.	.	.	.	.	.	.	.	.	.	.	.	.	.	.	.
7019	0,5	THOC2	NM_001081550	chrX	122562092	122694585	-	.	.	.	.	.	.	.	.	.	.	.	.	.	.	.	.	.	.	.	.
981	2,5	XIAP	NM_001167	chrX	122821697	122875510	+	.	.	.	.	.	.	.	.	.	.	.	.	.	.	.	.	.	.	.	.
686	3	STAG2	NM_006603	chrX	122923236	123064186	+	CL529234	chrX	123041009	+	.	.	.	.	.	.	.	.	.	.	.	.	.	.	.	.
686	3	STAG2	NM_006603	chrX	122923236	123064186	+	BH609631	chrX	123007838	-	.	.	.	.	.	.	.	.	.	.	.	.	.	.	.	.
686	3	STAG2	NM_006603	chrX	122923236	123064186	+	BH609630	chrX	123056442	+	.	.	.	.	.	.	.	.	.	.	.	.	.	.	.	.
5229	1	SH2D1A	NM_001114937	chrX	123307812	123334691	+	.	.	.	.	.	.	.	.	.	.	.	.	.	.	.	.	.	.	.	.
1589	2	ODZ1	NM_014253	chrX	123337436	123925347	-	.	.	.	.	.	.	.	.	.	.	.	.	.	.	.	.	.	.	.	.
1588	2	OCRL	NM_000276	chrX	128501932	128554211	+	.	.	.	.	.	.	.	.	.	.	.	.	.	.	.	.	.	.	.	.
823	2,5	ELF4	NM_001421	chrX	129026575	129072156	-	.	.	.	.	.	.	.	.	.	.	.	.	.	.	.	.	.	.	.	.
5877	0,75	AIFM1	NM_001130847	chrX	129091018	129127542	-	.	.	.	.	.	.	.	.	.	.	.	.	.	.	.	.	.	.	.	.
1709	2	RAB33A	NM_004794	chrX	129133453	129146525	+	.	.	.	.	.	.	.	.	.	.	.	.	.	.	.	.	.	.	.	.
4124	1	IGSF1	NM_001170962	chrX	130235162	130251084	-	.	.	.	.	.	.	.	.	.	.	.	.	.	.	.	.	.	.	.	.
4086	1	HS6ST2	NM_147175	chrX	131587718	131923093	-	.	.	.	.	.	.	.	.	.	.	.	.	.	.	.	.	.	.	.	.
261	4	GPC3	NM_001164618	chrX	132497441	132947339	-	.	.	.	.	.	.	.	.	.	.	.	.	.	.	.	.	1088_129_1	chrX	132838309	+
2193	1,5	HPRT1	NM_000194	chrX	133421840	133462364	+	CL799719	chrX	133454926	+	.	.	.	.	.	.	.	.	.	.	.	.	.	.	.	.
3718	1	FAM127C	NM_001078173	chrX	133982199	133984232	-	.	.	.	.	.	.	.	.	.	.	.	.	.	.	.	.	.	.	.	.
3778	1	FHL1	NM_001159702	chrX	135057224	135121184	+	.	.	.	.	.	.	.	.	.	.	.	.	.	.	.	.	.	.	.	.
4354	1	MAP7D3	NM_024597	chrX	135126640	135161404	-	.	.	.	.	.	.	.	.	.	.	.	.	.	.	.	.	.	.	.	.
1430	2	GPR112	NM_153834	chrX	135210787	135326713	+	.	.	.	.	.	.	.	.	.	.	.	.	.	.	.	.	.	.	.	.
2518	1,25	BRS3	NM_001727	chrX	135397790	135402264	+	.	.	.	.	.	.	.	.	.	.	.	.	.	.	.	.	.	.	.	.
4099	1	HTATSF1	NM_014500	chrX	135407336	135422169	+	.	.	.	.	.	.	.	.	.	.	.	.	.	.	.	.	.	.	.	.
203	4,25	CD40LG	NM_000074	chrX	135558001	135570215	+	.	.	.	.	.	.	.	.	.	.	.	.	.	.	.	.	.	.	.	.
6030	0,5	ARHGEF6	NM_004840	chrX	135575376	135691169	-	CL529727	chrX	135588229	-	.	.	.	.	.	.	.	.	.	.	.	.	.	.	.	.
6804	0,5	RBMX	NR_028477	chrX	135783271	135790605	-	.	.	.	.	.	.	.	.	.	.	.	.	.	.	.	.	.	.	.	.
3945	1	GPR101	NM_054021	chrX	135939972	135941499	-	.	.	.	.	.	.	.	.	.	.	.	.	.	.	.	.	.	.	.	.
574	3	FGF13	NM_004114	chrX	137541399	137621493	-	.	.	.	.	.	.	.	.	.	.	.	.	.	.	.	.	.	.	.	.
6602	0,5	MCF2	NM_001099855	chrX	138491595	138618047	-	.	.	.	.	.	.	.	.	.	.	.	.	.	.	.	.	.	.	.	.
3062	1	ATP11C	NM_173694	chrX	138636170	138742113	-	CL800555	chrX	138653818	-	.	.	.	.	.	.	.	.	.	.	.	.	.	.	.	.
7565	0,25	SOX3	NM_005634	chrX	139412817	139414891	-	.	.	.	.	.	.	.	.	.	.	.	.	.	.	.	.	.	.	.	.
2067	1,5	CDR1	NM_004065	chrX	139693090	139694389	-	.	.	.	.	.	.	.	.	.	.	.	.	.	.	.	.	.	.	.	.
7568	0,25	SPANXA1	NM_013453	chrX	140499471	140500502	-	.	.	.	.	.	.	.	.	.	.	.	.	.	.	.	.	.	.	.	.
7569	0,25	SPANXA2	NM_145662	chrX	140505500	140506565	+	.	.	.	.	.	.	.	.	.	.	.	.	.	.	.	.	.	.	.	.
2235	1,5	MAGEC3	NM_177456	chrX	140810117	140813284	+	.	.	.	.	.	.	.	.	.	.	.	.	.	.	.	.	.	.	.	.
7412	0,25	MAGEC1	NM_005462	chrX	140819307	140824853	+	.	.	.	.	.	.	.	.	.	.	.	.	.	.	.	.	.	.	.	.
2653	1,25	MAGEC2	NM_016249	chrX	141117793	141120742	-	.	.	.	.	.	.	.	.	.	.	.	.	.	.	.	.	.	.	.	.
2407	1,5	SLITRK4	NM_001184750	chrX	142538260	142549985	-	.	.	.	.	.	.	.	.	.	.	.	.	.	.	.	.	.	.	.	.
7315	0,25	FMR1NB	NM_152578	chrX	146870540	146915879	+	.	.	.	.	.	.	.	.	.	.	.	.	.	.	.	.	.	.	.	.
4113	1	IDS	NM_000202	chrX	148368200	148394789	-	.	.	.	.	.	.	.	.	.	.	.	.	.	.	.	.	.	.	.	.
6589	0,5	MAGEA11	NM_005366	chrX	148575476	148580667	-	.	.	.	.	.	.	.	.	.	.	.	.	.	.	.	.	.	.	.	.
4457	1	MTMR1	NM_003828	chrX	149612526	149684233	+	.	.	.	.	.	.	.	.	.	.	.	.	.	.	.	.	.	.	.	.
6157	0,5	CD99L2	NM_134445	chrX	149685466	149817947	-	.	.	.	.	.	.	.	.	.	.	.	.	.	.	.	.	.	.	.	.
5916	0,75	HMGB3	NM_005342	chrX	149902420	149909906	+	.	.	.	.	.	.	.	.	.	.	.	.	.	.	.	.	.	.	.	.
3955	1	GPR50	NM_004224	chrX	150095713	150100595	+	.	.	.	.	.	.	.	.	.	.	.	.	.	.	.	.	.	.	.	.

Table S2

tumor associated genes								HIV				MLV				MMTV				MMTV(SIN)				MMTV(SIN)arrest			
rank	score	gene symbol	RefSEq	chrom	txStart	txEnd	strand	integrant	chr	position	strand	integrant	chr	position	strand	integrant	chr	position	strand	integrant	chr	position	strand	integrant	chr	position	strand
3403	1	CNGA2	NM_005140	chrX	150653873	150664692	+	.	.	.	.	.	.	.	.	.	.	.	.	.	.	.	.	.	.	.	.
2652	1,25	MAGEA4	NM_001011548	chrX	150832016	150844298	+	.	.	.	.	.	.	.	.	.	.	.	.	.	.	.	.	.	.	.	.
7409	0,25	MAGEA10	NM_021048	chrX	151052438	151057706	-	.	.	.	.	.	.	.	.	.	.	.	.	.	.	.	.	.	.	.	.
6373	0,5	GABRA3	NM_000808	chrX	151086289	151370487	-	.	.	.	.	.	.	.	.	.	.	.	.	.	.	.	.	.	.	.	.
4328	1	MAGEA6	NM_175868	chrX	151617900	151621470	+	.	.	.	.	.	.	.	.	.	.	.	.	.	.	.	.	.	.	.	.
2650	1,25	MAGEA2	NM_175743	chrX	151633774	151637751	+	.	.	.	.	.	.	.	.	.	.	.	.	.	.	.	.	.	.	.	.
2651	1,25	MAGEA2B	NM_153488	chrX	151636039	151637735	+	.	.	.	.	.	.	.	.	.	.	.	.	.	.	.	.	.	.	.	.
2555	1,25	CSAG2	NM_001080848	chrX	151678389	151679394	+	.	.	.	.	.	.	.	.	.	.	.	.	.	.	.	.	.	.	.	.
3344	1	CETN2	NM_004344	chrX	151746526	151749957	-	.	.	.	.	.	.	.	.	.	.	.	.	.	.	.	.	.	.	.	.
4628	1	NSDHL	NM_001129765	chrX	151750166	151788563	+	.	.	.	.	.	.	.	.	.	.	.	.	.	.	.	.	.	.	.	.
2328	1,5	PNMA5	NM_001103151	chrX	151908023	151911417	-	.	.	.	.	.	.	.	.	.	.	.	.	.	.	.	.	.	.	.	.
5948	0,75	PNMA3	NM_013364	chrX	151975421	151979483	+	.	.	.	.	.	.	.	.	.	.	.	.	.	.	.	.	.	.	.	.
738	2,75	MAGEA1	NM_004988	chrX	152134715	152139310	-	.	.	.	.	.	.	.	.	.	.	.	.	.	.	.	.	.	.	.	.
5548	1	TREX2	NM_080701	chrX	152363371	152365139	-	.	.	.	.	.	.	.	.	.	.	.	.	.	.	.	.	.	.	.	.
3144	1	BGN	NM_001711	chrX	152413540	152428198	+	.	.	.	.	.	.	.	.	.	.	.	.	.	.	.	.	.	.	.	.
3068	1	ATP2B3	NM_001001344	chrX	152454773	152501581	+	.	.	.	.	.	.	.	.	.	.	.	.	.	.	.	.	.	.	.	.
1370	2	DUSP9	NM_001395	chrX	152561090	152569975	+	.	.	.	.	.	.	.	.	.	.	.	.	.	.	.	.	.	.	.	.
2327	1,5	PNCK	NM_001135740	chrX	152588381	152591937	-	.	.	.	.	.	.	.	.	.	.	.	.	.	.	.	.	.	.	.	.
2856	1	ABCD1	NM_000033	chrX	152643516	152663410	+	.	.	.	.	.	.	.	.	.	.	.	.	.	.	.	.	.	.	.	.
1639	2	PLXNB3	NM_001163257	chrX	152682844	152697995	+	.	.	.	.	.	.	.	.	.	.	.	.	.	.	.	.	.	.	.	.
1775	2	SRPK3	NM_001170760	chrX	152699649	152704381	+	.	.	.	.	.	.	.	.	.	.	.	.	.	.	.	.	.	.	.	.
4110	1	IDH3G	NM_004135	chrX	152704414	152713172	-	BH609633	chrX	152711051	+	.	.	.	.	.	.	.	.	.	.	.	.	.	.	.	.
1090	2,25	L1CAM	NM_000425	chrX	152780164	152794593	-	.	.	.	.	.	.	.	.	.	.	.	.	.	.	.	.	.	.	.	.
7180	0,25	AVPR2	NM_001146151	chrX	152823722	152825814	+	.	.	.	.	.	.	.	.	.	.	.	.	.	.	.	.	.	.	.	.
6424	0,5	HCFC1	NM_005334	chrX	152866201	152890013	-	.	.	.	.	.	.	.	.	.	.	.	.	.	.	.	.	.	.	.	.
1477	2	IRAK1	NM_001025243	chrX	152929150	152938536	-	.	.	.	.	.	.	.	.	.	.	.	.	.	.	.	.	.	.	.	.
2672	1,25	MECP2	NM_001110792	chrX	152948879	153016382	-	CL529634	chrX	153000470	+	.	.	.	.	.	.	.	.	.	.	.	.	.	.	.	.
2672	1,25	MECP2	NM_001110792	chrX	152948879	153016382	-	BH609634	chrX	152985635	+	.	.	.	.	.	.	.	.	.	.	.	.	.	.	.	.
2672	1,25	MECP2	NM_001110792	chrX	152948879	153016382	-	CL800363	chrX	153003755	-	.	.	.	.	.	.	.	.	.	.	.	.	.	.	.	.
2672	1,25	MECP2	NM_001110792	chrX	152948879	153016382	-	CL800589	chrX	152963356	-	.	.	.	.	.	.	.	.	.	.	.	.	.	.	.	.
5472	1	TKTL1	NM_001145934	chrX	153186602	153211907	+	.	.	.	.	.	.	.	.	.	.	.	.	.	.	.	.	.	.	.	.
727	2,75	FLNA	NM_001110556	chrX	153230093	153256200	-	CL800134	chrX	153247748	+	.	.	.	.	.	.	.	.	.	.	.	.	.	.	.	.
7287	0,25	DNASE1L1	NM_006730	chrX	153282772	153290769	-	.	.	.	.	.	.	.	.	.	.	.	.	.	.	.	.	.	.	.	.
2506	1,25	ATP6AP1	NM_001183	chrX	153310171	153318056	+	.	.	.	.	.	.	.	.	.	.	.	.	.	.	.	.	.	.	.	.
6896	0,5	SLC10A3	NM_001142392	chrX	153368843	153372196	-	.	.	.	.	.	.	.	.	.	.	.	.	.	.	.	.	.	.	.	.
1417	2	G6PD	NM_000402	chrX	153412799	153428427	-	.	.	.	.	.	.	.	.	.	.	.	.	.	.	.	.	.	.	.	.
4127	1	IKBKG	NM_001099857	chrX	153428755	153446455	+	.	.	.	.	.	.	.	.	.	.	.	.	.	.	.	.	.	.	.	.
2558	1,25	CTAG1B	NM_001327	chrX	153466611	153468269	+	.	.	.	.	.	.	.	.	.	.	.	.	.	.	.	.	.	.	.	.
2559	1,25	CTAG2	NM_020994	chrX	153533439	153535047	-	.	.	.	.	.	.	.	.	.	.	.	.	.	.	.	.	.	.	.	.
571	3	F8	NM_019863	chrX	153717257	153767771	-	.	.	.	.	.	.	.	.	.	.	.	.	.	.	.	.	.	.	.	.
1546	2	MTCP1	NM_001018025	chrX	153945502	153952741	-	.	.	.	.	.	.	.	.	.	.	.	.	.	.	.	.	.	.	.	.
1277	2	BRCC3	NM_024332	chrX	153952888	154004543	+	CL529606	chrX	153999724	-	.	.	.	.	.	.	.	.	.	.	.	.	.	.	.	.
3387	1	CLIC2	NM_001289	chrX	154158693	154217180	-	.	.	.	.	.	.	.	.	.	.	.	.	.	.	.	.	.	.	.	.
5502	1	TMLHE	NM_018196	chrX	154371866	154495816	-	.	.	.	.	.	.	.	.	.	.	.	.	.	.	.	.	.	.	.	.
6222	0,5	CSF2RA	NM_172249	chrY	1347692	1388828	+	.	.	.	.	.	.	.	.	.	.	.	.	.	.	.	.	.	.	.	.
5954	0,75	RPS4Y1	NM_001008	chrY	2769622	2794997	+	.	.	.	.	.	.	.	.	.	.	.	.	.	.	.	.	.	.	.	.
4705	1	PCDH11Y	NM_032971	chrY	4928266	5033485	+	.	.	.	.	.	.	.	.	.	.	.	.	.	.	.	.	.	.	.	.
4948	1	PRKY	NR_028062	chrY	7202012	7309588	+	.	.	.	.	.	.	.	.	.	.	.	.	.	.	.	.	.	.	.	.
7083	0,5	USP9Y	NM_004654	chrY	13322553	13482162	+	CL799559	chrY	13380222	+	.	.	.	.	.	.	.	.	.	.	.	.	.	.	.	.
6261	0,5	DDX3Y	NM_001122665	chrY	13525412	13541784	+	.	.	.	.	.	.	.	.	.	.	.	.	.	.	.	.	.	.	.	.
6676	0,5	NLGN4Y	NR_028319	chrY	15145847	15465242	+	.	.	.	.	.	.	.	.	.	.	.	.	.	.	.	.	.	.	.	.

Table S2

tumor associated genes								HIV				MLV				MMTV				MMTV(SIN)				MMTV(SIN)arrest			
rank	score	gene symbol	RefSEq	chrom	txStart	txEnd	strand	integrant	chr	position	strand	integrant	chr	position	strand	integrant	chr	position	strand	integrant	chr	position	strand	integrant	chr	position	strand
7236	0,25	CD24	NM_013230	chrY	19611913	19614093	-	.	.	.	.	.	.	.	.	.	.	.	.	.	.	.	.	.	.	.	.
1926	1,75	KDM5D	NM_001146706	chrY	20326688	20366213	-	.	.	.	.	.	.	.	.	.	.	.	.	.	.	.	.	.	.	.	.
5904	0,75	EIF1AY	NM_004681	chrY	21146998	21164428	+	.	.	.	.	.	.	.	.	.	.	.	.	.	.	.	.	.	.	.	.
6253	0,5	DAZ1	NM_004081	chrY	23684889	23754627	-	.	.	.	.	.	.	.	.	.	.	.	.	.	.	.	.	.	.	.	.
7266	0,25	DAZ2	NM_001005785	chrY	23774991	23846891	+	.	.	.	.	.	.	.	.	.	.	.	.	.	.	.	.	.	.	.	.
6254	0,5	DAZ4	NM_001005375	chrY	25389354	25462575	+	.	.	.	.	.	.	.	.	.	.	.	.	.	.	.	.	.	.	.	.
7267	0,25	DAZ3	NM_020364	chrY	25389354	25462575	+	.	.	.	.	.	.	.	.	.	.	.	.	.	.	.	.	.	.	.	.
